# Harnessing Bifunctional *N*‐Benzoyloxyamides for Photoredox Amidative Dual Functionalizations of Alkenes

**DOI:** 10.1002/anie.202506290

**Published:** 2025-06-16

**Authors:** Harin Ryoo, Hyeyun Keum, Dongwook Kim, Sukbok Chang

**Affiliations:** ^1^ Department of Chemistry Korea Advanced Institute of Science and Technology (KAIST) Daejeon 34141 Republic of Korea; ^2^ Center for Catalytic Hydrocarbon Functionalizations Institute for Basic Science (IBS) Daejeon 34141 Republic of Korea

**Keywords:** Amidyl radical, Bifunctional N─O reagent, Olefin amido‐functionalizations, Photocatalysis, Radical reactions

## Abstract

Here, we present a photocatalytic strategy for the intermolecular dual functionalizations of alkenes using N─O bifunctional reagents in an atom‐economical fashion. By leveraging *N*‐benzoyloxyamides as bifunctional precursors for generating both amidyl radical and internal O‐nucleophiles, this approach achieves chemoselective olefin amidation with simultaneous incorporation of additional functional groups. The current method readily accesses a range of doubly functionalized amino products through 1,2‐amidooxygenation, amidoazidation, and formal *anti*‐Markovnikov hydroamidation. Mechanistic studies revealed that the selective interplay of radical and ionic pathways is operative to enable a unified platform for this olefin dual functionalization.

## Graphical abstract

A novel photocatalytic strategy is presented for intermolecular amidative dual functionalization of alkenes using *N*‐benzoyloxyamides as bifunctional N─O reagents. The method enables efficient olefin 1,2‐amidooxygenation and significantly extends its reactivity to encompass amidoazidation, diamination, and formal *anti*‐Markovnikov hydroamidation. By harnessing the reactivity of both amidyl radicals and O‐nucleophiles via mesolytic N─O bond cleavage, this approach demonstrates a unified platform for dual amido‐functionalization of olefins utilizing both radical and ionic reaction pathways.

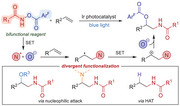

Given the abundance of alkenes as readily available feedstocks, selective olefin functionalization represents a fundamental transformation in synthetic, medicinal, and polymer chemistry.^[^ ^1^, ^2^, ^3^ ^]^ Among these, intermolecular amination with concurrent incorporation of additional functional groups across the alkene double bonds represents a valuable route to nitrogen‐containing compounds with potential further transformations.^[^ ^4^, ^5^, ^6^, ^7^, ^8^, ^9^, ^10^ ^]^ In this context, olefin dual functionalization via radical addition using photoactive bifunctional reagents has proven to be an efficient protocol for installing both C─N and C─X bonds (X = C, O, N, halides, etc.) in a step‐economical manner.^[^ ^11^, ^12^, ^13^, ^14^, ^15^, ^16^, ^17^, ^18^, ^19^, ^20^ ^]^

Recent advances in photoredox chemistry have led to several elegant examples of olefin amino‐functionalizations using bifunctional reagents, especially showing notable reactivity in carboamination and chloroamination (Scheme 1a). For instance, Hong and Powers groups independently demonstrated the utilization of *N*‐aminopyridinium ylides for the *ortho*‐selective aminopyridylation of alkenes.^[^ ^21^, ^22^ ^]^ In this approach, the photocatalytic single‐electron oxidation of those reagents was proposed to generate radical cations that undergo 1,3‐cycloaddition with alkenes. Additionally, Hong and co‐workers reported a C4‐selective aminopyridylation of alkenes using an analogous aminating reagent, enabling simultaneous incorporation of aminyl radical and pyridyl group into olefins.^[^ ^23^ ^]^ Notably, Stephenson and Nevado groups have respectively developed photocatalytic strategies for the aminoarylation of alkenes using sulfonyl bifunctional reagents as amine and arene donors.^[^ ^19^, ^20^, ^24^, ^25^, ^26^ ^]^ Moreover, *N*‐chlorosulfonamides and oxime‐contained substrates have also been employed as bifunctional reagents to achieve olefin aminochlorination.^[^ ^27^, ^28^ ^]^

**Scheme 1:**
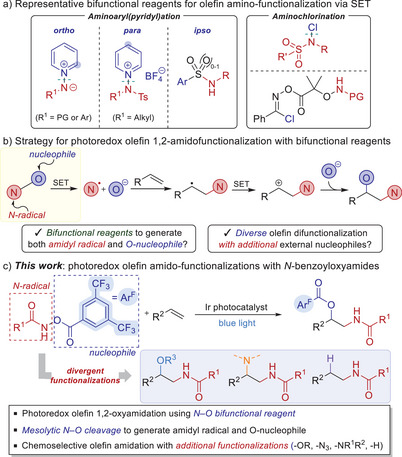
Utility of bifunctional reagents in photocatalytic olefin amino‐functionalizations.

Despite these significant advances, achieving photoredox‐mediated 1,2‐amidooxygenation of olefins using bifunctional oxyaminating reagents remains an important challenge. While N‐radical generation via N─O bond cleavage of such precursors has been well documented, these reagents usually provide O‐anion fragments, which merely function either as an electron transfer mediator^[^ ^5^, ^29^, ^30^, ^31^, ^32^, ^33^, ^34^, ^35^, ^36^, ^37^, ^38^ ^]^ or as an internal base.^[^ ^39^, ^40^ ^]^ Based on our efforts to harness the reactivity of photogenerated N‐centered intermediates for the C─N bond‐forming processes,^[^ ^40^, ^41^, ^42^, ^43^, ^44^ ^]^ we sought to propose N─O bifunctional reagents capable of delivering amidyl radicals and O‐nucleophiles, both of which may add into alkenes (Scheme 1b). In this approach, amidyl radicals, generated via single‐electron reduction of the N─O reagents, would react regioselectively with alkenes to form a transient C‐radical. This species may undergo radical‐polar crossover (RPC) upon single‐electron oxidation, leading to a carbocation intermediate, which will eventually undergo nucleophilic addition with the released O‐anions.

Building on this design, herein, we report a photocatalytic 1,2‐amidooxygenation of olefins enabled by the dual reactivity of bifunctional *N*‐benzoyloxyamide reagents, which generate both amidyl radical and O‐nucleophiles via mesolytic N─O bond cleavage (Scheme 1c). Moreover, this approach was found to enable the incorporation of additional external nucleophiles such as alcohols, azides, and heterocycles, while also facilitating hydroamidation via hydrogen atom transfer (HAT) catalysis.

To evaluate the feasibility of the proposed intermolecular 1,2‐amidooxygenation using bifunctional reagents, we initially focused on the selective introduction of both C─N and C─O bonds onto olefins. Readily accessible and bench‐stable *N*‐[{3,5‐bis(trifluoromethyl)benzoyl}oxy]benzamide (**1a**, 1.5 equiv.) was initially chosen as a bifunctional N─O reagent to serve as both nitrogen and oxygen source (Table 1). For this, Ir(ppy)_3_ was examined as the photocatalyst and *p*‐methoxystyrene (**2a**, 1 equiv.) as the alkene substrate under 427 nm blue light irradiation. To our delight, the desired aminooxygenated product **3a** was obtained in 64% ^1^H NMR yield in dichloromethane (DCM) after 16 h at room temperature (entry 1). The reaction efficiency was influenced to some extents by the solution concentrations, as shown in entries 2 and 3. It should be mentioned that the reaction was highly selective in that no regioisomeric aminooxygenated product was detected.

**Table 1:** Optimization of reaction parameters in olefin 1,2‐amidooxygenation.^a)^

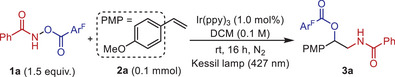		

Entry	Variations from the standard conditions	3a (%)
1	None	64
2	DCM (0.05 M)	61
3	DCM (0.14 M)	**75** (67)^b)^
*Variation from entry 3*		
4	**1b** instead of **1a**	8
5	**1c** instead of **1a**	n.d.
6	**1d** instead of **1a**	n.d.
7	3DPAFIPN instead of Ir(ppy)_3_	35
8	**1a** (1.0 equiv.)	49
9	3 h	55
10	Ir(ppy)_3_ (2.0 mol%)	75
11	without light	n.d.
12	without photocatalyst	n.d.
13	under air	14
14	1,1,2,2‐TCE	56
15	EtOAc	37
16	DMF	6
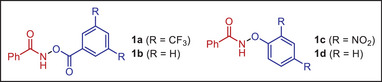		

1,1,2,2‐TCE, 1,1,2,2,‐tetrachloroethane; DCM, dichloromethane.

^a)^ Yields based on ^1^H NMR analysis of the reaction mixture (internal standard: dibromomethane).

^b)^ Isolated yield.

Importantly, the structural nature of the bifunctional reagents was found to be critical to the reaction progress. For instance, **1b** bearing a *N*‐benzoyloxy moiety (R = H) reacted with significantly lower yield (entry 4). Moreover, *O*‐arylhydroxamates (**1c** and **1d**), previously utilized as an exclusive source of N‐radicals upon photocatalytic N─O bond cleavage,^[^ ^29^, ^36^, ^37^ ^]^ failed to deliver the desired olefin dual functionalization (entries 5 and 6). The organophotocatalyst 3DPAFIPN was also examined and found to promote the reaction with diminished reactivity compared to the optimized iridium catalyst (entry 7).

The robustness of this protocol using **1a** was further demonstrated by observing its efficiency under the decreased equivalent of **1a** or reduced reaction time (entries 8 and 9). Additionally, we found that it proceeded efficiently with only 1.0 mol% of photocatalyst while no benefit was observed at higher catalyst loading (entry 10). Control experiments confirmed that both the photocatalyst and visible‐light irradiation were essential for this reaction (entries 11 and 12) and that maintaining an inert atmosphere was also necessary (entry 13). On the other hand, the reaction turned out to be sensitive to the choice of solvents: while the use of chlorinated solvents such as 1,1,2,2,‐tetrachloroethane (TCE) was still efficient albeit slightly decreased yield (entry 14), the use of polar media was less effective (entries 15 and 16).

With the optimized conditions in hand, we next explored the scope of the current photocatalytic 1,2‐amidooxygenation of alkenes using bifunctional N─O reagent **1a** (Scheme 2, top). Styrene analogues bearing electron‐donating substituents at the *para*‐position, including methoxy (**3a**), ethoxy (**3b**), *tert*‐butoxy (**3c**), and phenoxy (**3d**), afforded the desired products in moderate to good yields. The synthetic utility of the 1,2‐amidooxygenation was further demonstrated on a gram‐scale reaction. An *ortho*‐substituted styrene (**3e**) was also compatible albeit with slightly lower yield, which may be attributed to the reduced electronic effect compared to *para*‐substitution, in addition to the steric influence.^[^ ^45^ ^]^ In addition, styrenes featuring di‐substituted (**3f**) or fused skeleton (**3** **g**) participated in the difunctionalization. Internal olefins such as *β*‐methylstyrene (**3** **h**) also underwent 1,2‐amidooxygenation, delivering the desired product in moderate yield. Notably, this protocol was amenable to vinyl thioether, yielding **3i** under the standard conditions.

**Scheme 2:**
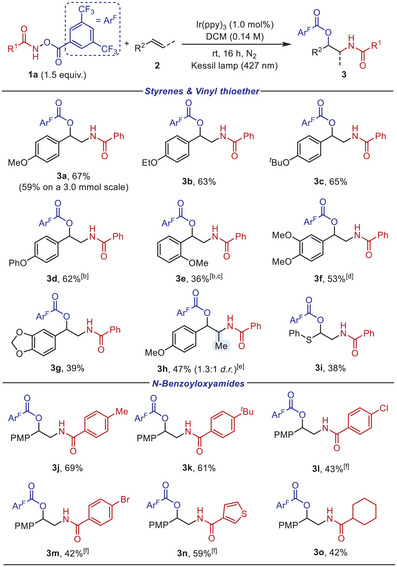
Olefin 1,2‐amidooxygenation with bifunctional *N*‐benzoyloxyamide.^a) a)^ Conditions: 1a (1.5 equiv.), 2 (0.1 mmol, 1.0 equiv.), and Ir(ppy)_3_ (1.0 mol%) in DCM (0.14 M) at room temperature for 16 h under 427 nm blue light irradiation. ^b)^ Run at 60 °C. ^c)^ Ir(ppy)_3_ (2.0 mol%). ^d)^ 1a (2.0 equiv.). ^e)^ Diastereoisomeric ratio determined by ^1^H NMR analysis of crude mixture. ^f)^ Ir(ppy)_3_ (5.0 mol%).

While this method represents the first example of a photoredox 1,2‐amidooxygenation using bifunctional N─O reagents, its applicability to substrates with electron‐withdrawing properties remained limited, suggesting an intrinsic electronic influence on the reaction efficiency (see the Supporting Information for details). However, the scope of the bifunctional N─O reagents proved highly versatile, accommodating a diverse range of amide derivatives with varying electronic properties (Scheme 2, bottom). *N*‐Benzoyloxyamides bearing *para*‐substituents on the phenyl ring (**3j**–**3m**), as well as thiophenyl (**3n**) or cyclohexyl (**3o**), were well viable, thereby affording the desired products in good yields.

Given the applicability of *N*‐benzoyloxyamide as a bifunctional reagent, we questioned whether our reaction protocol could be further extended to introduce, in addition to C─N bond formation, a different type of C─O bond such as an ether linkage by utilizing external alcohol nucleophiles (Scheme 3, top). Notably, during the reaction optimization (Tables S3−S5), the DCM/O‐nucleophile (2:3) ratio was found to afford the highest product yields, likely due to a synergistic effect between competent radical reactivity and sufficient O‐nucleophile concentration. In this case, a variety of *para*‐substituted styrenes could be successfully converted to 1,2‐amidooxygenated products (**4a**–**4f**), regardless of the electronic property on the aryl moiety, well accommodating styrene derivatives bearing even electron‐withdrawing substituents.

**Scheme 3:**
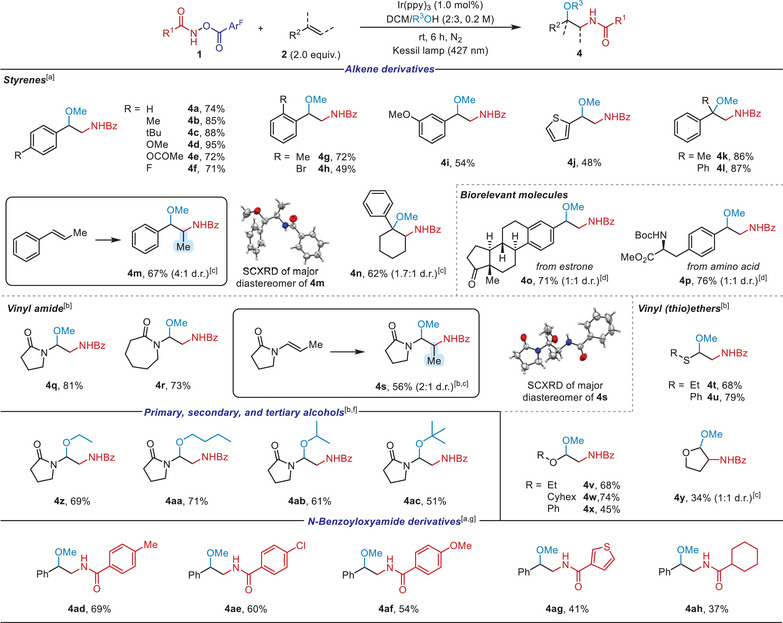
Intermolecular 1,2‐amidooxyggenation of alkenes using external alcohol nucleophiles.^a,e) a)^ Conditions: 1 (0.1 mmol), 2 (2.0 equiv.) and Ir(ppy)_3_ (1.0 mol%) in DCM/R^3^OH (2:3, 0.2 M) at room temperature for 6 h under 427 nm blue light irradiation. ^b)^ Reactions performed for 1 h. ^c)^ Diastereoisomeric ratio was determined by ^1^H NMR analysis of the crude mixture. ^d)^ Diastereoisomeric ratio was determined by ^13^C NMR analysis of isolated product. ^e)^ R_1_ = Ph; R_3_ = Me. ^f)^ R_1_ = Ph; R_2_ = *N*‐pyrrolidin‐2‐one. ^g)^ R_2_ = Ph; R_3_ = Me.

Moreover, the position of substituents turned out to be also flexible in that substrates bearing *ortho*‐ (**4** **g** and **4** **h**) and *meta*‐substituent (**4i**) as well as vinyl thiophene (**4j**) were readily amenable. 1,1‐Disubstituted alkenes were also viable, affording the corresponding products in high yields, featuring a quaternary carbon center (**4k** and **4l**). The evaluation of *β*‐methylstyrene also resulted in the desired product (**4m**) in satisfactory yield with moderate diastereoselectivity, and the structure of the major isomer was confirmed by an X‐ray diffraction (SCXRD) analysis. Additionally, a trisubstituted cyclic olefin smoothly underwent the reaction, yielding **4n** under the standard conditions. To further highlight the synthetic utility of the current transformation, alkenes derived from biorelevant molecules, such as estrone or amino acid derivatives, were briefly examined to afford the desired products in good yields (**4o** and **4p**, respectively).

Significantly, this protocol was not limited to styrene analogs but was also applicable to heteroatom‐substituted alkenes, including enamides (**4q**–**4s**), vinyl thioethers (**4t** and **4u**), and vinyl ethers (**4v**–**4y**), affording the desired products with excellent regioselectivity. It needs to be emphasized that this photoredox amidooxygenation approach employing external O‐nucleophiles displayed broader applicability across various olefinic classes, featuring the advantage of the current amidyl radical‐mediated dual functionalization of alkenes.

Next, we sought to expand this amidooxygenation by employing additional alcohols in reaction with 1‐vinylpyrrolidine‐2‐one (Scheme 3, middle). Various types of alcohols, including ethanol (**4z**), *n*‐butanol (**4aa**), isopropanol (**4ab**), and *tert*‐butanol (**4ac**), were all successfully incorporated, providing the desired products in moderate to good yield. Furthermore, we investigated the flexibility of various *N*‐benzoyloxyamides and found that both aryl‐ and alkyl‐substituted derivatives were suitable for delivering the corresponding amidooxygenation products (**4ad**–**4ah**). Notably, the facile incorporation of alcohol nucleophiles as proved above supports the involvement of the proposed carbocation intermediate in our strategy (Scheme 1b).

To further demonstrate the potential of our developed protocol as a platform for olefin amidative dual functionalization, we aimed to further introduce unsymmetrical diamino functionalities, which are highly valuable in organic synthesis^[^ ^14^, ^46^, ^47^, ^48^ ^]^ (Scheme 4). It should be mentioned that Waser and co‐workers recently reported an interesting photocatalytic dual 1,2‐azidofunctinalization of alkenes via RPC using a hypervalent cyclic iodine azide precursor.^[^ ^49^ ^]^ However, a general protocol for the synthetically versatile 1,2‐diamination of alkenes is still highly sought after.^[^ ^47^, ^49^, ^50^, ^51^ ^]^ We were pleased to find that 1,2‐amidoazidation could be readily achieved under our catalytic platform. Upon optimization of parameters (see the Supporting Information for details), the reaction of styrene with TMSN_3_ (1.2 equiv.) as an external N‐nucleophile afforded the azidoamidated product **5a** in 62% yield. Various styrene derivatives with either electron‐donating or withdrawing substituents underwent the 1,2‐amidoazidation smoothly, giving rise to the corresponding products in moderate to good yields (**5a**–**5f**). In addition, this protocol was amenable to vinyl amide, affording the desired product **5** **g**.

**Scheme 4:**
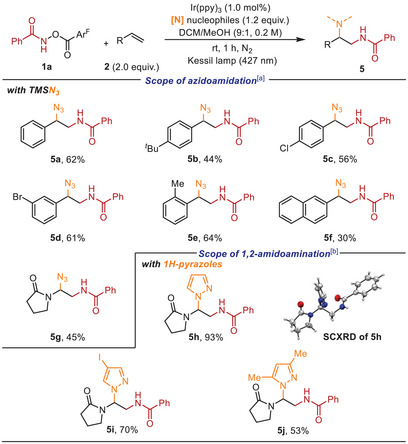
Intermolecular diamination of alkenes. ^a)^ Conditions: 1a (0.1 mmol, 1.0 equiv.), 2 (2.0 equiv.), TMSN_3_ (1.2 equiv.), and Ir(ppy)_3_ (1.0 mol%) in DCM/MeOH (9:1, 0.2 M) at room temperature for 1 h under 427 nm blue light irradiation. ^b)^ Conditions: *1H*‐pyrazole derivatives instead of TMSN_3_ and DCM (0.2 M) instead of DCM/MeOH (9:1, 0.2 M).

Furthermore, we briefly examined olefin diamination using heterocycles as an external N‐nucleophile, which may have potential applicability in medicinal chemistry.^[^ ^52^, ^53^, ^54^ ^]^ *1H*‐Pyrazoles functioned as effective N‐nucleophiles in this transformation, selectively enabling the exclusive formation of the regioisomeric 1,2‐amidoamination products (**5h**–**5j**). The structure of **5** **h** was unambiguously confirmed by SCXRD analysis.

To delineate mechanistic pathways of the current dual functionalization of alkenes using a bifunctional N─O reagent, we performed combined experimental and DFT (density functional theory) studies (Figure 1a). The measured reduction potential of *N*‐benzoyloxyamide **1a** (E*_p,red_* = –1.54 V vs. SCE in MeCN; Standard Calomel Electrode) indicates that it can be effectively quenched by excited photocatalyst Ir^*III^(ppy)_3_ (E*_1/2,ox_ = –1.73 V vs. SCE in MeCN),^[^ ^55^, ^56^, ^57^ ^]^ thus generating an anionic radical species **1a’** (see the Supporting Information for cyclic voltammetry measurements). Subsequently, **1a’** was calculated to undergo the N─O bond cleavage to afford an amidyl radical **A** and benzoate anion **B** with a low activation barrier (11.10 kcal mol^−1^). To gain more insights into the following regioselective radical addition into olefin, we compared two possible isomeric pathways through computational analysis. The energy barrier for the amidyl radical addition to the terminal position of styrene **2a** (**TS‐I**) was found to be lower by 4.05 kcal mol^−1^ than that for the addition at the internal carbon center (**TS‐I’**), thus in agreement with the observed regioselective outcome. The carbon‐centered radical **I** (E*_calc,ox_* = + 0.49 V vs. SCE) would be then readily oxidized by the resultant Ir^IV^(ppy)_3_ (E*_1/2,red_* = + 0.77 V vs. SCE in MeCN),^[^ ^55^, ^56^, ^57^ ^]^ giving rise to a carbocation intermediate **II**, which ultimately undergoes a nucleophilic attack by the benzoate anion **B** to afford the regioselective 1,2‐aminooxygenated product **3a**.

**Figure 1:**
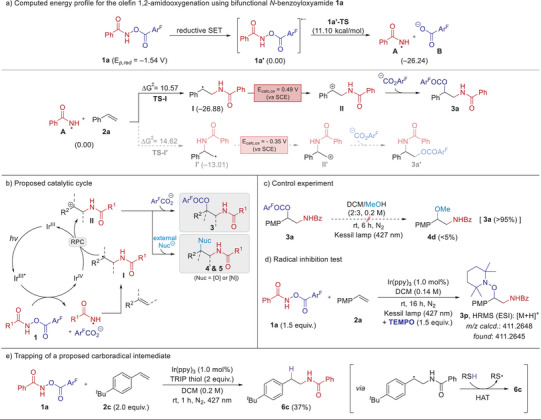
Mechanistic investigations on the photocatalytic olefin amido‐functionalizations with bifunctional N─O reagents. The Gaussian 09 level of M06‐2X/6–311 + G**(SMD, dichloromethane)//M06‐2X/6–31G** was used (values given in units of kcal mol^−1^).

Based on the above olefin amido‐functionalization results and computed energy profile, we propose a catalytic cycle depicted in Figure 1b. To validate this pathway, we first conducted a control experiment to investigate the observed chemoselectivity. The 1,2‐amidooxygenation product **3a** remained predominantly unreacted with methanol even under photoirradiation conditions, with only a minor amount of alcohol‐substituted compound **4d** (<5%) formed (Figure 1c). Moreover, even upon the addition of N‐nucleophiles, **3a** remained intact, and no 1,2‐diamination products were formed (see the Supporting Information for details). Additionally, a Stern‐Volmer experiment showed that the excited iridium photocatalyst is effectively quenched by *N*‐benzoyloxyamide. Furthermore, the low quantum yield (Φ *=* 0.034) observed for the 1,2‐amidooxygenation indicates that the reaction likely proceeds through a closed photoredox catalytic cycle (see the Supporting Information for details).

When the olefin amido‐functionalization was carried out in the presence of 2,2,6,6‐tetramethyl‐1‐piperidinyloxy (TEMPO), a TEMPO adduct **3p** was detected by HRMS with the complete suppression to form an amidooxygenation product **3a** (Figure 1d). This result suggests an intermediacy of a carboradical **I** in the present reaction progress. Indeed, this putative radical species could be trapped by using 2,4,6‐triisopropylbenzenethiol (TRIP thiol), presumably via hydrogen‐atom transfer (HAT), eventually leading to a formal *anti*‐Markovnikov hydroamidation product **6c** (Figure 1e). This radical trapping experiment not only supports the mechanistic pathway involving a carboradical intermediate but also underscores the potential of our system for developing a formal hydroamidation protocol.

Inspired by recent advances in photocatalytic intermolecular radical‐radical cross‐coupling reactions,^[^ ^31^, ^58^, ^59^, ^60^, ^61^, ^62^, ^63^, ^64^ ^]^ we further examined a range of HAT catalysts as well as potential hydrogen donors. Notably, we found that the *anti*‐Markovnikov hydroamidated product **6a** could be more efficiently obtained upon further optimizations (see the Supporting Information for details). The reaction proceeded smoothly at room temperature within 1 h in the presence of Ir(ppy)_3_ (1 mol%), TRIP thiol (50 mol%) as the HAT catalyst, and γ‐terpinene (1.0 equiv.) as the hydrogen donor. Control experiments showed that H donor, HAT catalyst, photocatalyst, and blue light irradiation each played a crucial role in the reaction.

With the optimized conditions in hand, we subsequently evaluated the substrate scope of this formal *anti*‐Markovnikov hydroamidation (Scheme 5). The reaction proved effective for various styrene derivatives bearing substituents such as methyl (**6b**), *tert*‐butyl (**6c**), chloro (**6d**), and methoxy (**6e**) groups. Additionally, a double bond containing 1,3‐benzodioxole moiety (**6f**) or 2‐methylstyrene (**6** **g**) underwent regioselective amidation. Notably, a disubstituted olefin was also amenable to afford **6** **h**, and its structure was confirmed by SCXRD analysis. Furthermore, the protocol was shown to be compatible with heteroatom‐containing olefins, such as vinyl thioethers (**6i** and **6j**) and vinyl sulfone (**6k**), furnishing the corresponding products in good to excellent yields.

**Scheme 5:**
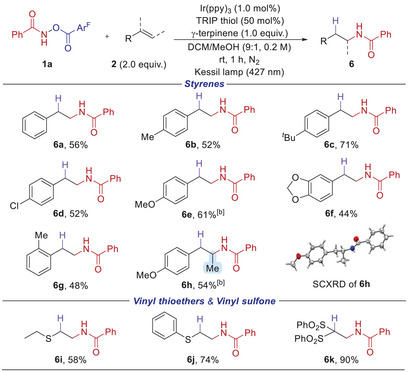
Intermolecular formal *anti*‐Markovnikov hydroamidation of alkenes.^a) a)^ Conditions: 1a (0.1 mmol), 2 (2.0 equiv.), TRIP thiol (50 mol%), γ‐terpinene (1.0 equiv.), and Ir(ppy)_3_ (1.0 mol%) in DCM/MeOH (9:1, 0.2 M) at room temperature for 1 h under 427 nm irradiation. ^b)^ DCM/*^t^* BuOH (9:1, 0.2 M).

In summary, we have developed photoredox intermolecular olefin 1,2‐amidooxygenation, utilizing *N*‐benzoyloxyamides as bifunctional reagents for the first time. This approach operates through mesolytic N─O bond cleavage, allowing the simultaneous generation of an amidyl radical along with anionic O‐nucleophiles, which sequentially add to olefinic double bonds. Beyond 1,2‐amidooxygenation, this method also enables an additional range of olefin amidative dual functionalizations, including 1,2‐amidooxygenation with alcohols, 1,2‐amidoazidation with TMSN_3_, 1,2‐diamination using pyrazoles, and formal *anti*‐Markovnikov hydroamidation. Our work demonstrates the potential utility of bifunctional N─O reagents in a modular approach for dual amido‐functionalization of olefins by integrating radical and ionic pathways within a unified catalytic system.

## Conflict of Interests

The authors declare no conflict of interest.

## Supporting information



Supporting Information

Supporting Information

## Data Availability

The data that support the findings of this study are available in the Supporting Information of this article.
