# Supplementary material for: Harnessing Bifunctional N‐Benzoyloxyamides for Photoredox Amidative Dual Functionalizations of Alkenes
Source: Angew Chem Int Ed Engl. 2025 Jun 16;64(32):e202506290. doi: 10.1002/anie.202506290 (PMC12322644; doi:10.1002/anie.202506290)
Supplement: Supplementary file 1 — Supporting Information [file ANIE-64-e202506290-s002.pdf]

Table of Contents

I. General Considerations S2
II. Procedures for the Preparation of Starting Materials S3
III. Optimization of Reaction Parameters S9
IV. Procedures of Intermolecular Olefin Amidative Dual Functionalizations S14
V. Procedures for the Experimental Mechanistic Studies S40
VI. Computational Studies S47
VII. ^{1}H, ^{13}C and ^{19}F NMR Spectra S53
VIII. Crystallographic Data S162
IX. References S166

I. General Considerations

Reagent preparation: Unless otherwise stated, all commercial reagents and solvents were used without additional purification. $Ir(ppy)_3$, $Ir(4'\text{-}F\text{-}ppy)_3$, $Ir(2',4'\text{-}dF\text{-}ppy)_3$ were purchased from TCI, Aldrich, or Strem chemical company. All photocatalysts were used without further purification. Flash column chromatography was undertaken using Combi*Flash*® R_f+ system with Redi*Sep*® R_f silica columns or alumina neutral columns using conditions noted in the procedure.

Product characterization: ^1H NMR spectra were recorded on Agilent Technologies DD2 (600 MHz), Bruker AV-500 (500 MHz), Bruker IBS-500 (500 MHz), Bruker AVHD-400 (400 MHz), or Bruker AS-400 (400 MHz), at room temperature unless otherwise noted. Chemical shifts were quoted in parts per million (ppm) referenced to the residual solvent peak (e.g. $CHCl_3$ in $CDCl_3$: 7.26 ppm). The following abbreviations were used to describe peak splitting patterns when appropriate: s = singlet, d = doublet, dd = doublet of doublet, ddd = doublet of doublet of doublet, dt = doublet of triplet, t = triplet, td = triplet of doublet, tt = triplet of triplet, tq = triplet of quartet, q = quartet, p = pentet, hept = heptet, and m = multiplet. Coupling constants, *J*, were reported in hertz (Hz). ^{13}C NMR spectra were obtained on Agilent Technologies DD2 (151 MHz), Bruker AV-500 (126 MHz), Bruker IBS-500 (126 MHz), Bruker AS-400 (101 MHz), or Bruker AVNEO-400 (101 MHz) and were fully decoupled by broad band proton decoupling. Chemical shifts were reported in ppm referenced to the residual solvent peak. ^{19}F NMR spectra were recorded on Bruker AV-500 (471 MHz), Bruker IBS-500 (471 MHz), Bruker AVNEO-400 (377 MHz), Bruker AVHD-400 (376 MHz), and Bruker AS-400 (376 MHz). Chemical shifts were reported in ppm referenced to external α,α,α-trifluorotoluene as -63.72 ppm. Infrared (IR) spectra were recorded on Bruker Alpha FT-IR Spectrometer. Frequencies are given in wave numbers (cm^{-1}) and only selected peaks were reported. High resolution mass spectra were obtained from Korea Basic Science Institute (KBSI, Daegu) by using EI (or FAB) method or KAIST Analysis Center for Research Advancement (KARA, Daejeon) by using ESI method. X-ray diffraction data was collected on a Bruker SMART APEX III or PAL beamline coated with Paraton-*N* oil under a stream of N_2 (g) at 133 K. Melting point was measured with Buchi Melting Point M-565.

Analytical method: Photoluminescence was taken using the Shimadzu RF-6000 Spectrofluorophotometer. Electrochemical data were obtained on a Bio-Logic Science VSP potentiostat. UV–Vis spectra were measured using a Shimadzu UV-2600 spectrophotometer.

II. Procedures for the Preparation of Starting Materials

Unless otherwise stated, all reagents were purchased from Sigma-Aldrich, Alfa, TCI, Strem, BLD pharm, Chemscene, Angene, or Enamine chemical company, and used as received without further purification.

1. Preparation of hydroxamic acids from carboxylic acids

Following chemicals were synthesized according to previously reported methods; 4-methyl-*N*-hydroxybenzamide,[1] 4-(*tert*-butyl)-*N*-hydroxybenzamide,[2] 4-chloro-*N*-hydroxybenzamide,[1] 4-bromo-*N*-hydroxybenzamide,[2] *N*-hydroxycyclohexanecarboxamide,[1] and *N*-hydroxy-4-methoxybenzamide.[1]

General Procedure for SM-A[3]

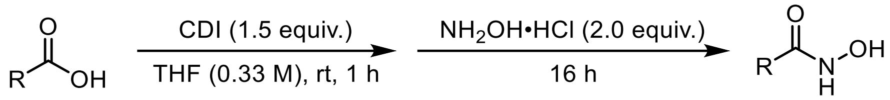

1,1'-Carbonyldiimidazole (CDI, 1.5 equiv.) was added to a solution of carboxylic acid in dry tetrahydrofuran (THF, 0.33 M). The reaction mixture was stirred at room temperature for 1 h, and powdered hydroxylamine hydrochloride (2.0 equiv.) was added. After stirring 16 h, the resulting solution was diluted with 5% aqueous $KHSO_4$ and extracted twice with ethyl acetate (EtOAc). The combined organic phase was washed with brine and dried over $MgSO_4$. The extract was filtered and concentrated under reduced pressure. Then, the crude product was purified by recrystallization (dichloromethane, DCM + few drops of methanol/*n*-pentane).

_N_-Hydroxythiophene-3-carboxamide

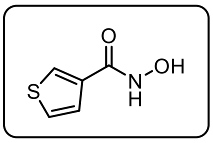

Colorless solid (314.2 mg, 73%, 3.0 mmol scale); **m.p.** 157–159 °C; **^{1}H NMR** (400 MHz, DMSO-*d$_6$*) δ 11.04 (s, 1H), 9.00 (s, 1H), 8.03 (d, *J* = 2.9 Hz, 1H), 7.59 (dd, *J* = 5.0, 3.0 Hz, 1H), 7.42 (dd, *J* = 5.0, 1.3 Hz, 1H); **^{13}C NMR** (101 MHz, DMSO-*d$_6$*) δ 160.3, 135.3, 128.2, 126.9, 126.2; **IR** (cm^{-1}) 3272, 2704, 1557, 1411, 1303, 845, 804, 725, 589, 520; **HRMS** (EI) m/z calcd. for $C_5H_5NO_2S$ $[M]^+$: 143.0041, found: 143.0043.

2. Preparation of *N*-[{3,5-bis(trifluoromethyl)benzoyl}oxy]amides

N-[{3,5-Bis(trifluoromethyl)benzoyl}oxy]benzamide was prepared according to the previously known method.[4,5]

General Procedure for SM-B[4]

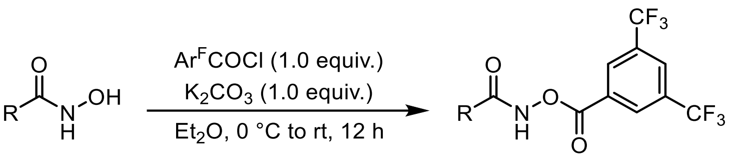

3,5-Bis(trifluoromethyl)benzoyl chloride (Ar^FCOCl, 1.0 equiv.) was added dropwise to a solution of hydroxamic acid and potassium carbonate (1.0 equiv.) in diethyl ether (Et_2O) at 0 °C, and the reaction mixture was warmed up to room temperature. After stirring 12 h, the resulting solution was diluted with water and extracted twice with EtOAc. The combined organic phase was dried over $MgSO_4$. The extract was filtered and concentrated under reduced pressure. Then, the crude product was purified by recrystallization (DCM + few drops of methanol/*n*-pentane) or silica gel column chromatography (*n*-hexane/EtOAc, 3:1).

N-[{3,5-Bis(trifluoromethyl)benzoyl}oxy]-4-methylbenzamide

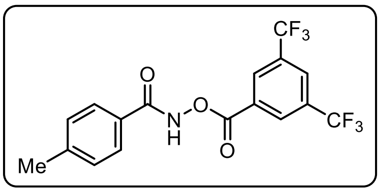

Colorless solid (592.0 mg, 89%, 1.7 mmol scale); **m.p.** 161–163 °C; **^{1}H NMR** (500 MHz, CD_2Cl_2) δ 9.56 (s, 1H), 8.62 (s, 2H), 8.20 (s, 1H), 7.77 (d, *J* = 8.2 Hz, 2H), 7.33 (d, *J* = 7.9 Hz, 2H), 2.44 (s, 3H); **^{13}C NMR** (101 MHz, CD_3OD) δ 168.2, 163.7, 144.8, 133.6 (q, *J* = 34.1 Hz), 131.3, 131.3 – 131.1 (m), 130.5, 129.1, 128.7, 128.6 – 128.4 (m), 124.3 (q, *J* = 272.2 Hz), 21.6; **^{19}F NMR** (377 MHz, CD_3OD) δ -64.5; **IR** (cm^{-1}) 3126, 2956, 1785, 1646, 1531, 1278, 1215, 1177, 1137, 749, 681; **HRMS** (EI) m/z calcd. for $C_{17}H_{11}F_6NO_3$ $[M]^+$: 391.0643, found: 391.0641.

N-[{3,5-Bis(trifluoromethyl)benzoyl}oxy]-4-(*tert*-butyl)benzamide

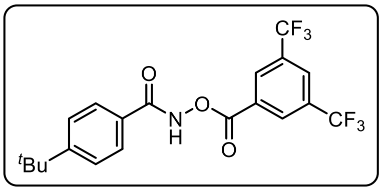

Colorless solid (820.4 mg, 95%, 2.0 mmol scale); **m.p.** 146–148 °C; **^{1}H NMR** (500 MHz, CD_2Cl_2) δ 10.08 (s, 1H), 8.55 (s, 2H), 8.13 (s, 1H), 7.78 (d, *J* = 7.8 Hz, 2H), 7.45 (d, *J* = 7.8 Hz, 2H), 1.32 (s, 9H); **^{13}C NMR** (126 MHz, $CDCl_3$) δ 166.9, 163.0, 157.1, 132.7 (q, *J* = 34.4 Hz), 130.4 – 130.0 (m), 129.2, 127.7 – 127.5 (m), 127.6, 127.2, 126.0, 122.7 (q, *J* = 274.1 Hz), 35.2, 31.1; **^{19}F NMR** (471 MHz, $CDCl_3$) δ -63.1; **IR** (cm^{-1}) 3161, 2970, 1778, 1656, 1498, 1382, 1280, 1216, 1177, 1135, 913, 681; **HRMS** (ESI) m/z calcd. for $C_{20}H_{17}F_6NO_3Na$ $[M+Na]^+$: 456.1010, found: 456.1006.

N-[{3,5-Bis(trifluoromethyl)benzoyl}oxy]-4-chlorobenzamide

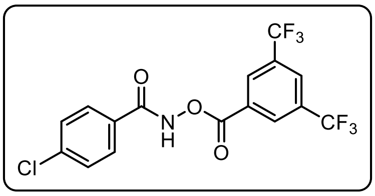

Colorless solid (704.5 mg, 86%, 2.0 mmol scale); **m.p.** 219–221 °C; **^{1}H NMR** (400 MHz, CD_2Cl_2) δ 9.73 (s, 1H), 8.61 (s, 2H), 8.21 (s, 1H), 7.88 – 7.78 (m, 2H), 7.55 – 7.46 (m, 2H); **^{13}C NMR** (101 MHz, CD_3OD) δ 167.1, 163.8, 139.7, 133.6 (q, J = 34.2 Hz), 131.5, 131.3 – 131.0 (m), 130.4, 130.1, 129.8, 128.6 – 128.4 (m), 124.3 (q, J = 272.2 Hz); **^{19}F NMR** (376 MHz, $CDCl_3$) δ -63.0; **IR** (cm^{-1}) 3116, 2974, 1787, 1646, 1277, 1215, 1182, 1149, 1134, 846, 681; **HRMS** (EI) m/z calcd. for $C_{16}H_8ClF_6NO_3$ $[M]^+$: 411.0097, found: 411.0100.

N-[{3,5-Bis(trifluoromethyl)benzoyl}oxy]-4-bromobenzamide

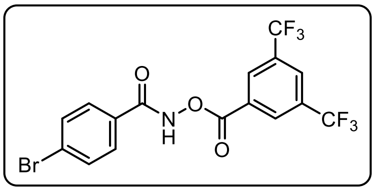

Colorless solid (372.6 mg, 82%, 1.0 mmol scale); **m.p.** 256–258 °C; **^{1}H NMR** (400 MHz, $CDCl_3$) δ 9.53 (s, 1H), 8.60 (s, 2H), 8.17 (s, 1H), 7.80 – 7.72 (m, 2H), 7.70 – 7.63 (m, 2H); **^{13}C NMR** (101 MHz, $CDCl_3$) δ 166.1, 163.1, 132.9 (q, J = 34.4 Hz), 132.5, 130.6 – 130.1 (m), 129.3, 129.1, 128.9, 128.5, 128.0 – 127.8 (m), 122.8 (q, J = 273.8 Hz); **^{19}F NMR** (377 MHz, CD_3OD) δ -64.5; **IR** (cm^{-1}) 3139, 2948, 1783, 1654, 1592, 1275, 1181, 1131, 842, 680.

N-[{3,5-Bis(trifluoromethyl)benzoyl}oxy]cyclohexanecarboxamide

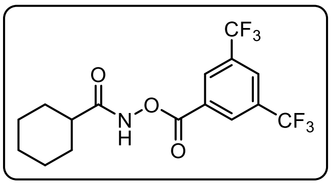

Colorless solid (571.7 mg, 83%, 1.8 mmol scale); **m.p.** 143–145 °C; **^{1}H NMR** (500 MHz, $CDCl_3$) δ 8.98 (s, 1H), 8.55 (s, 2H), 8.14 (s, 1H), 2.32 (tt, J = 11.6, 3.4 Hz, 1H), 1.99 – 1.89 (m, 2H), 1.89 – 1.81 (m, 2H), 1.71 (dd, J = 9.8, 3.9 Hz, 1H), 1.65 – 1.54 (m, 2H), 1.39 – 1.21 (m, 3H); **^{13}C NMR** (101 MHz, $CDCl_3$) δ 175.0, 162.8, 132.8 (q, J = 34.4 Hz), 130.2 (d, J = 4.0 Hz), 129.2, 127.6 (q, J = 3.7 Hz), 122.7 (q, J = 273.2 Hz), 42.5, 29.3, 25.6, 25.5; **^{19}F NMR** (376 MHz, $CDCl_3$) δ -63.0; **IR** (cm^{-1}) 3164, 2930, 2859, 1779, 1680, 1290, 1221, 1172, 1135, 701, 682; **HRMS** (FAB) m/z calcd. for $C_{16}H_{16}F_6NO_3$ $[M+H]^+$: 384.1034, found: 384.1030.

N-[{3,5-Bis(trifluoromethyl)benzoyl}oxy]thiophene-3-carboxamide

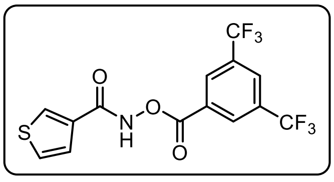

Colorless solid (658.6 mg, 86%, 2.0 mmol scale); **m.p.** 158–160 °C; **^{1}H NMR** (500 MHz, $CDCl_3$) δ 9.43 (s, 1H), 8.60 (s, 2H), 8.16 (s, 1H), 8.10 (dd, J = 2.9, 1.3 Hz, 1H), 7.50 (dd, J = 5.1, 1.3 Hz, 1H), 7.45 (dd, J = 5.1, 3.0 Hz, 1H); **^{13}C NMR** (101 MHz, $CDCl_3$) δ 163.1, 162.6, 132.8 (q, J = 34.5 Hz), 132.4, 131.0, 130.3 (q, J = 3.9 Hz), 129.1, 127.9 – 127.7 (m), 127.5, 126.2, 122.9 (q, J = 273.7 Hz); **^{19}F NMR** (376 MHz, $CDCl_3$) δ -63.0; **IR** (cm^{-1}) 1789, 1645, 1540, 1282, 1215, 1180, 1136, 745, 700, 681; **HRMS** (EI) m/z calcd. for $C_{14}H_7F_6NO_3S$ $[M]^+$: 383.0051, found: 383.0052.

N-[{3,5-Bis(trifluoromethyl)benzoyl}oxy]-4-methoxybenzamide

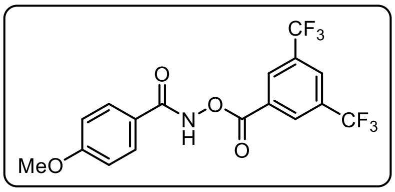

Colorless solid (891.2 mg, 88%, 2.5 mmol scale); **m.p.** 148–150 °C; **^{1}H NMR** (600 MHz, $CDCl_3$) δ 9.59 (s, 1H), 8.59 (s, 2H), 8.15 (s, 1H), 7.88 – 7.83 (m, 2H), 7.00 – 6.95 (m, 2H), 3.88 (s, 3H); **^{13}C NMR** (101 MHz, $CDCl_3$) δ 166.8, 163.8, 163.3, 132.8 (q, J = 34.5 Hz), 130.4 – 130.2 (m), 129.8, 129.3, 127.9 – 127.6 (m), 122.8 (q, J = 272.8 Hz), 122.4, 114.4, 55.7; **^{19}F NMR** (376 MHz, $CDCl_3$) δ -63.0; **IR** (cm^{-1}) 1782, 1645, 1503, 1278, 1217, 1136, 846, 682; **HRMS** (EI) m/z calcd. for $C_{17}H_{11}F_6NO_4$ $[M]^+$: 407.0592, found: 407.0594.

N-[{3,5-Bis(trifluoromethyl)benzoyl}oxy]acetamide (1e)

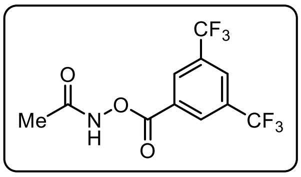

Colorless solid (953.6 mg, 61%, 5.0 mmol scale); **m.p.** 136–138 °C; **^{1}H NMR** (400 MHz, $CDCl_3$) δ 9.00 (s, 1H), 8.54 (s, 2H), 8.15 (s, 1H), 2.17 (s, 3H); **^{13}C NMR** (101 MHz, $CDCl_3$) δ 168.5, 162.7, 132.8 (q, J = 34.3 Hz), 130.4 – 130.0 (m), 129.0, 128.0 – 127.6 (m), 122.7 (q, J = 273.1 Hz), 19.9; **^{19}F NMR** (376 MHz, $CDCl_3$) δ -63.0; **IR** (cm^{-1}) 3098, 2917, 2808, 1769, 1660, 1279, 1113, 914, 682; **HRMS** (FAB) m/z calcd. for $C_{11}H_8F_6NO_3$ $[M+H]^+$: 316.0408, found: 316.0411.

3. Preparation of *N*-phenoxybenzamide

N-Phenoxybenzamide was prepared according to the previously known method.[6]

General Procedure for SM-C[6]

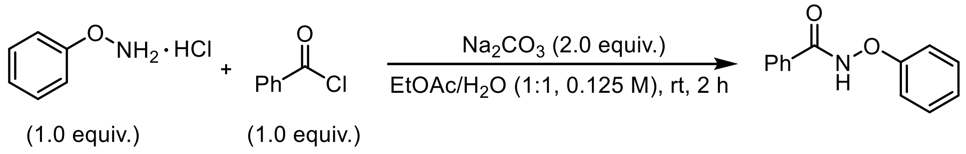

Benzoyl chloride (1.0 equiv.) was added dropwise to a solution of *O*-phenylhydroxylamine hydrochloride (1.0 equiv.) and sodium carbonate (2.0 equiv.) in EtOAc/H_2O (1:1, 0.125 M) at room temperature. After stirring for 2 h, the separated organic phase was washed with brine, dried over $MgSO_4$, filtered and concentrated under reduced pressure. The crude product was purified by column chromatography (*n*-hexane/EtOAc, 9:1).

General Procedure for SM-D[7]

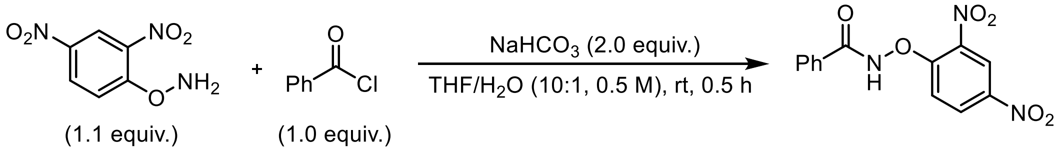

Benzoyl chloride (1.0 equiv.) was added dropwise over 20 min to a solution of *O*-(2,4-dinitrophenyl)hydroxylamine hydrochloride (1.1 equiv.) and sodium bicarbonate (2.0 equiv.) in THF/H_2O (10:1, 0.5 M) at room temperature. After stirring for 30 min, the reaction mixture was diluted with water and extracted twice with EtOAc. Then, the combined organic phase was washed with brine, dried over $MgSO_4$, filtered and concentrated under reduced pressure. The crude product was purified by column chromatography (*n*-hexane/EtOAc, 9:1).

N-(2,4-Dinitrophenoxy)benzamide (1d)

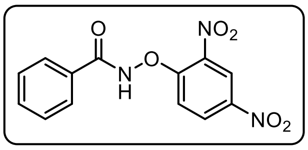

Colorless solid (87.1 mg, 38%, 0.75 mmol scale); **m.p.** 145–147 °C; **^{1}H NMR** (500 MHz, $CDCl_3$) δ 9.03 (d, *J* = 2.7 Hz, 1H), 8.53 (dd, *J* = 9.3, 2.7 Hz, 1H), 8.18 (d, *J* = 9.3 Hz, 1H), 8.06 (s, 1H), 7.99 (d, *J* = 7.4 Hz, 2H), 7.58 (t, *J* = 7.4 Hz, 1H), 7.50 (t, *J* = 7.7 Hz, 2H); **^{13}C NMR** (126 MHz, $CDCl_3$) δ 161.0, 156.7, 145.8, 145.2, 141.3, 132.5, 130.3, 128.9, 126.9, 122.6, 117.7; **IR** (cm^{-1}) 3121, 2924, 1666, 1608, 1518, 1345, 1262, 1155, 1067, 1012, 927, 828, 713, 661, 510; **HRMS** (ESI) m/z calcd. for $C_{13}H_9N_3O_6Na$ $[M+Na]^+$: 326.0389, found: 326.0389.

4. Preparation of olefins

Following chemicals were was prepared according to the previously known method: (8*R*,9*S*,13*S*,14*S*)-13-methyl-3-vinyl-6,7,8,9,11,12,13,14,15,16-decahydro-17*H*-cyclopenta[*a*]phenanthrene-17-one,[8] methyl (*S*)-2-{(*tert*-butoxycarbonyl)amino}-3-(4-vinylphenyl)propanoate[9,10] and (*E*)-1-(prop-1-en-1-yl)pyrrolidin-2-one.[11,12]

General Procedure for SM-E[8–10]

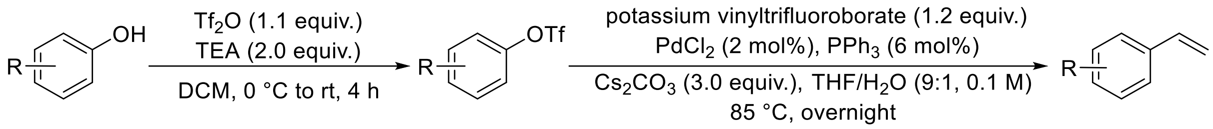

A flame-dried flask was charged with phenol (1.0 equiv.), triethylamine (2.0 equiv.), and DCM (0.1 M). The mixture was cooled down to 0 °C and Tf_2O (1.1 equiv.) was added dropwise. The crude mixture was warmed up to room temperature and stirred under Ar for 6 h. The resulting mixture was diluted with DCM, washed with saturated NH_4Cl and the aqueous layer was extracted with DCM. The combined organic layer was dried over anhydrous $MgSO_4$, filtered and evaporated under reduced pressure. The triflate product was obtained by column chromatography.

A flame-dried flask was charged with triflate (1.0 equiv.), potassium vinyltrifluoroborate (1.2 equiv.), $PdCl_2$ (2 mol%), PPh_3 (6 mol%), Cs_2CO_3 (3.0 equiv.) in THF/H_2O (9:1, 0.1 M). The mixture was stirred at 85 °C under Ar for overnight. The crude mixture was allowed to cool down to room temperature, diluted with DCM, washed with water, and the aqueous layer was extracted with dichloromethane. The combined organic layer was dried over anhydrous $MgSO_4$, filtered and evaporated under reduced pressure. The desired product was obtained by column chromatography.

General Procedure for SM-F[11]

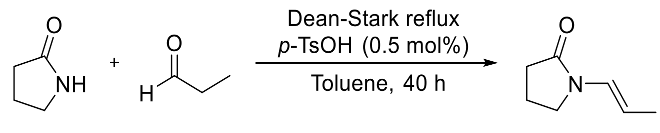

A flame-dried flask was charged with lactam (1.0 equiv.), aldehyde (1.0 equiv.), *p*-toluenesulfonic acid (0.5 mol%) in dry toluene. The mixture was heated at reflux by a Dean-Stark trap and stirred for 40 h until no more water was collected. The resulting mixture was cooled down to room temperature and washed with EtOAc. The combined organic layer was dried over anhydrous $MgSO_4$, filtered and evaporated under reduced pressure. The desired product was obtained by column chromatography.

III. Optimization of Reaction Parameters

1. Optimization of olefin 1,2-amidooxygenation using bifunctional *N*-benzoyloxyamide

To an oven-dried 4 mL vial with a stir bar were added Ir(ppy)$_3$ (1.0 mol%), *N*-[{3,5-bis(trifluoromethyl)benzoyl}oxy]benzamide (**1a**, 0.15 mmol, 1.5 equiv.), *p*-methoxystyrene (**2a**, 0.1 mmol, 1.0 equiv.) and anhydrous DCM (0.14 M) under N_2 atmosphere. The vial was stirred at room temperature for the indicated time under irradiation with a Kessil lamp (427 nm, maximum intensity), which was positioned 3~5 cm away from the reaction vial, and regular fans were equipped to maintain the temperature. Solvent was removed under reduced pressure and the crude product yield was determined by using ^{1}H NMR spectroscopy (dibromomethane as an internal standard in $CDCl_3$).

Table S1. Screening of photocatalysts

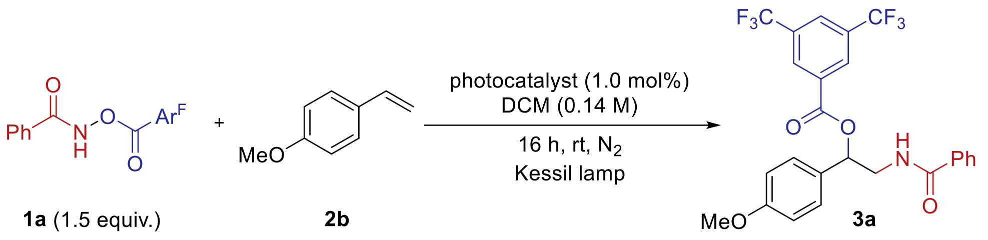

Entry	photocatalyst	Light wavelength (nm)	Yield (**3a**, %)
1	Ir(ppy)$_3$	427	75
2	3DPAFIPN	427	35
3	Thioxanthone	370	11
4	Xanthone	370	n.d.
5	Eosin Y	525	n.d.

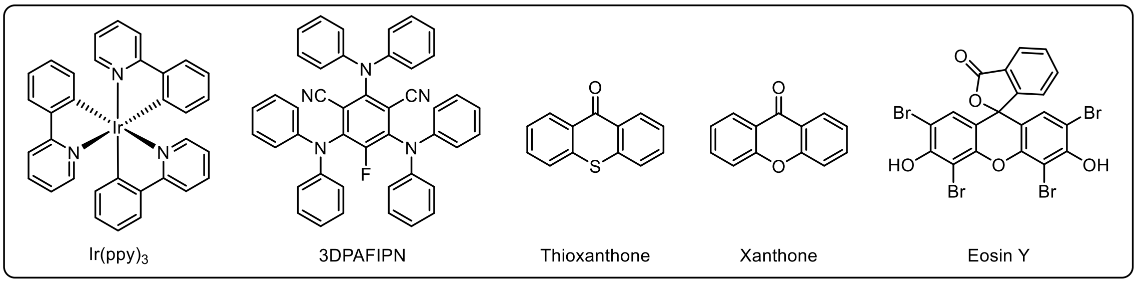

Table S2. Reaction time test

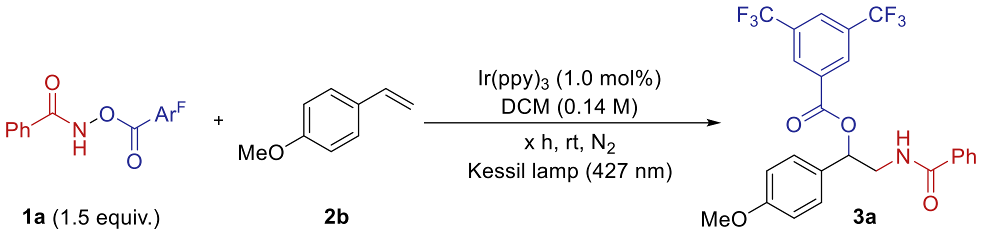

Entry	Time (h)	Yield (**3a**, %)
1	3	55
2	6	60
3	12	68
4	16	75
5	24	73

2. Optimization study for olefin amidooxygenation using alcohol as external nucleophile

To an oven-dried 4 mL vial with a stir bar were added Ir(ppy)$_3$ (1.0 mol%), *N*-[{3,5-bis(trifluoromethyl)benzoyl}oxy]benzamide (**1a**, 0.1 mmol, 1.0 equiv.), styrene (**2b**, 0.2 mmol, 2.0 equiv.) and anhydrous DCM/MeOH (2:3, 0.2 M) under N_2 atmosphere. The vial was stirred at room temperature for 6 h under irradiation with a Kessil lamp (427 nm, maximum intensity), which was positioned 3~5 cm away from the reaction vial. Regular fans were equipped to maintain the temperature. Solvent was removed under reduced pressure and the crude product yield was determined by using ^{1}H NMR spectroscopy (dibromomethane as an internal standard in $CDCl_3$).

Table S3. Screening of photocatalysts

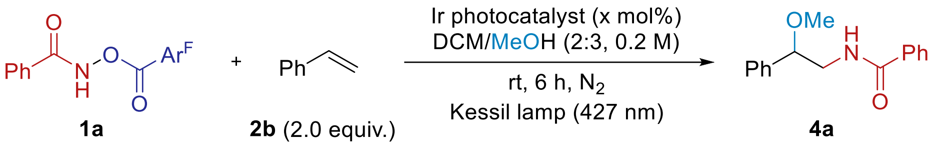

Entry	Ir photocatalyst	mol%	Yield (**4a**, %)
1	Ir(ppy)$_3$	1.0	80
2	Ir(4'-F-ppy)$_3$	1.0	57
3	Ir(2',4'-dF-ppy)$_3$	1.0	21
4	Ir(ppy)$_3$	2.0	71

Table S4. Stoichiometry test of two reactants

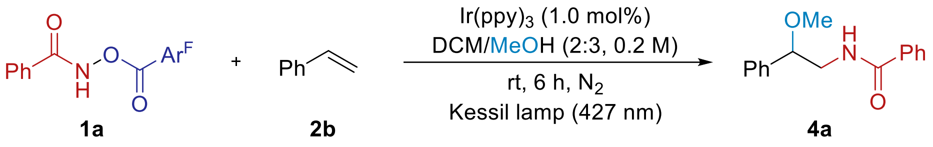

Entry	**1a** (equiv.)	**2b** (equiv.)	Yield (**4a**, %)
1	1.0	2.0	80
2	1.0	1.5	75
3	1.0	1.0	49
4	1.5	1.0	74

Table S5. Concentration and ratio of co-solvent

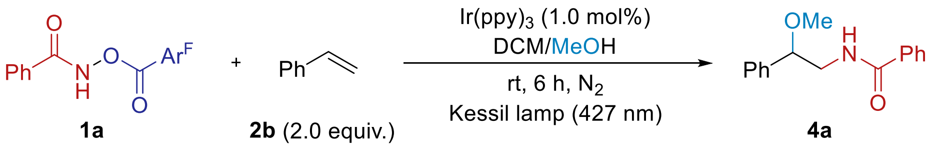

Entry	Concentration (M)	DCM/MeOH ratio	Yield (**4a**, %)
1	0.2	1:4	72
2	0.2	2:3	80
3	0.2	1:1	66
4	0.1	2:3	67

3. Optimization study for olefin amidoazidation

To an oven-dried 4 mL vial with a stir bar were added Ir(ppy)$_3$ (1.0 mol%), *N*-[{3,5-bis(trifluoromethyl)benzoyl}oxy]benzamide (**1a**, 0.1 mmol, 1.0 equiv.), styrene (**2b**, 0.2 mmol, 2.0 equiv.), trimethylsilyl azide (0.12 mmol, 1.2 equiv.) and anhydrous DCM/MeOH (9:1, 0.2 M) under N_2 atmosphere. The vial was stirred at room temperature for 1 h under irradiation with a Kessil lamp (427 nm, maximum intensity), which was positioned 3~5 cm away from the reaction vials. Regular fans were equipped to maintain the temperature. Solvent was removed under reduced pressure and the crude product yield was determined by using ^{1}H NMR spectroscopy (dibromomethane as an internal standard in $CDCl_3$).

Table S6. Optimization of amidoazidation reaction

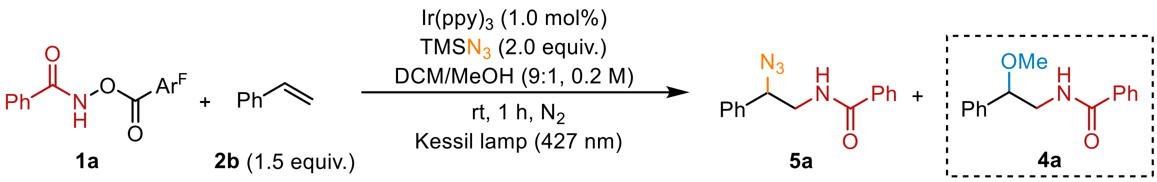

Entry	Variations from the standard conditions	Yield (**5a**, %)	**4a** (%)
1	None	55	<5
2	DCM/MeOH (4:1, 0.2 M)	35	5
3	DCM/MeOH (1:1, 0.2 M)	46	11
4	DCM/MeOH (1:4, 0.2 M)	22	15
5	DCM (0.2 M)	25	n.d.
6	$TMSN_3$ (1.2 equiv.)	68	<5
7	$TMSN_3$ (1.5 equiv.)	60	<5
8	$TMSN_3$ (1.2 equiv.), **2b** (2.0 equiv.)	70	<5

4. Optimization study for formal olefin hydroamidation

To an oven-dried 4 mL vial with a stir bar were added Ir(ppy)$_3$ (1.0 mol%), *N*-[{3,5-bis(trifluoromethyl)benzoyl}oxy]benzamide (**1a**, 0.1 mmol, 1.0 equiv.), 4-*tert*-butylstyrene (**2c**, 0.2 mmol, 2.0 equiv.), 2,4,6-triisopropylbenzenethiol (TRIP thiol, 50 mol%), γ-terpinene (0.1 mmol, 1.0 equiv.) and anhydrous DCM/MeOH (9:1, 0.2 M) under N_2 atmosphere. The vial was stirred at room temperature for 1 h under irradiation with a Kessil lamp (427 nm, maximum intensity), which was positioned 3~5 cm away from the reaction vial. Regular fans were equipped to maintain the temperature. Solvent was removed under reduced pressure and the crude product yield was determined by using ^{1}H NMR spectroscopy (dibromomethane as an internal standard in $CDCl_3$).

Table S7. Optimization of formal *anti*-Markovnikov hydroamidation reaction

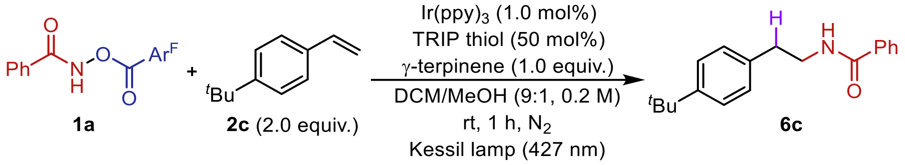

Entry	Variations from the standard conditions	Yield (**6c**, %)
1	None	**72**
2	3DPAFIPN instead of Ir(ppy)$_3$	30
3	TRIP thiol (20 mol%) instead of TRIP thiol (50 mol%)	55
4	Cyclohexa-1,4-diene instead of γ-terpinene	62
5	DCM instead of DCM/MeOH (9:1)	31
6	w/o γ-terpinene	27
7	w/o TRIP thiol	<5
8	w/o Ir(ppy)$_3$	<5
9	In dark conditions	<5

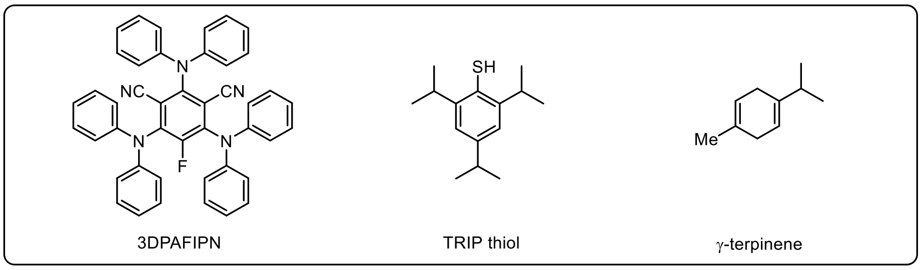

IV. Procedures of Intermolecular Olefin Amidative Dual Functionalizations

1. 1,2-Amidooxygenation of alkenes using bifunctional *N*-benzoyloxyamides

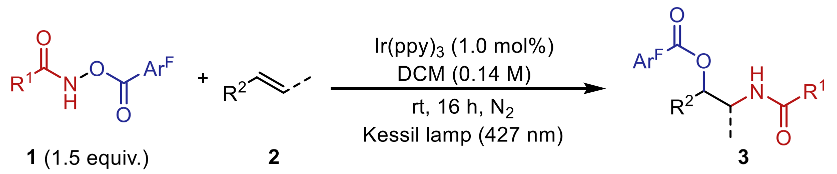

General Procedure A. To an oven-dried 4 mL vial with a stir bar were added Ir(ppy)$_3$ (1.0 mol%), *N*-benzoyloxyamide (**1**, 0.15 mmol, 1.5 equiv.), alkene substrate (**2**, 0.1 mmol, 1.0 equiv.) and anhydrous DCM (0.14 M) under N_2 atmosphere. The vial was stirred at room temperature for 16 h under irradiation with a Kessil lamp (427 nm, maximum intensity), which was positioned 3~5 cm away from the reaction vial. Regular fans were equipped to maintain the temperature. The reaction mixture was poured into saturated $NaHCO_3$ aqueous solution (10 mL) and extracted with DCM (10 mL x 3). The combined organic layer was dried over anhydrous $MgSO_4$, filtered and evaporated under reduced pressure. Desired product was obtained by silica gel or neutral alumina column chromatography (*n*-hexane/EtOAc, 9:1 ~ 4:1).

2. 1,2-Amidooxygenation of alkenes using external alcohols

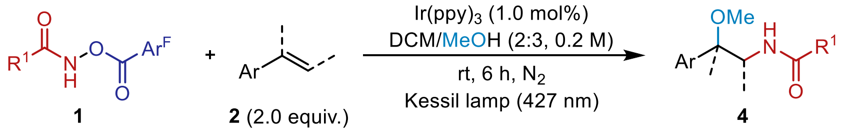

General Procedure B1 *for styrenes or vinyl thiophene.* To an oven-dried 4 mL vial with a stir bar were added Ir(ppy)$_3$ (1.0 mol%), *N*-benzoyloxyamide (**1**, 0.1 mmol, 1.0 equiv.), alkene substrate (**2**, 0.2 mmol, 2.0 equiv.) and anhydrous DCM/MeOH (2:3, 0.2 M) under N_2 atmosphere. The vial was stirred at room temperature for 6 h under irradiation with a Kessil lamp (427 nm, maximum intensity), which was positioned 3~5 cm away from the reaction vial. Regular fans were equipped to maintain the room temperature. The reaction mixture was poured into saturated $NaHCO_3$ aqueous solution (10 mL) and extracted with DCM (10 mL x 3). The combined organic layer was dried over anhydrous $MgSO_4$, filtered and evaporated under reduced pressure. Desired product was obtained by silica gel column chromatography (Pet Ether/EtOAc, 9:1 ~ 1:1).

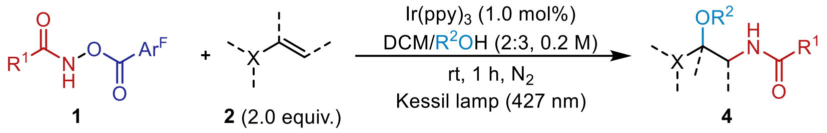

General Procedure B2 ***for vinyl (thio)ethers or vinyl amides*****.** To an oven-dried 4 mL vial with a stir bar were added Ir(ppy)$_3$ (1.0 mol%), *N*-benzoyloxyamide (**1**, 0.1 mmol, 1.0 equiv.), alkene substrate (**2**, 0.2 mmol, 2.0 equiv.) and anhydrous DCM/R^{2}OH (2:3, 0.2 M) under N_2 atmosphere. The vial was stirred at room temperature for 1 h under irradiation with a Kessil lamp (427 nm, maximum intensity), which was positioned 3~5 cm away from the reaction vial. Regular fans were equipped to maintain the temperature. The reaction mixture was poured into saturated $NaHCO_3$ aqueous solution (10 mL) and extracted with DCM (10 mL x 3). The combined organic layer was dried over anhydrous $MgSO_4$, filtered and evaporated under reduced pressure. Desired product was obtained by silica gel or neutral alumina column chromatography (Pet Ether/EtOAc, 9:1 ~ 1:1).

3. 1,2-Amidoaziation and 1,2-amidoamination of alkenes using external N-nucleophiles

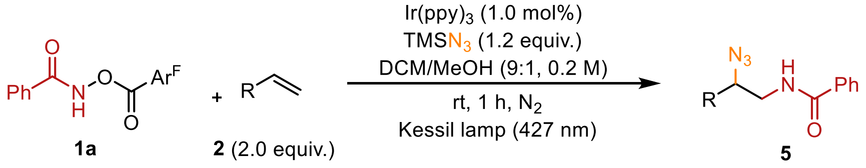

General Procedure C1 (Amidoazidation) ***using TMSN$_3$*****.** To an oven-dried 4 mL vial with a stir bar were added Ir(ppy)$_3$ (1.0 mol%), *N*-[{3,5-bis(trifluoromethyl)benzoyl}oxy]benzamide (**1a**, 0.1 mmol, 1.0 equiv.), alkene substrate (**2**, 0.2 mmol, 2.0 equiv.), TMSN$_3$ (0.12 mmol, 1.2 equiv.) and anhydrous DCM/MeOH (9:1, 0.2 M) under N_2 atmosphere. The vial was stirred under irradiation at room temperature for 1 h with a Kessil lamp (427 nm, maximum intensity), which was positioned 3~5 cm away from the reaction vial. Regular fans were equipped to maintain the temperature. The reaction mixture was poured into saturated $NaHCO_3$ aqueous solution (10 mL) and extracted with DCM (10 mL x 3). The combined organic layer was dried over anhydrous $MgSO_4$, filtered and evaporated under reduced pressure. Desired product was obtained by silica gel or neutral alumina column chromatography (Pet Ether/EtOAc, 9:1 ~ 1:1).

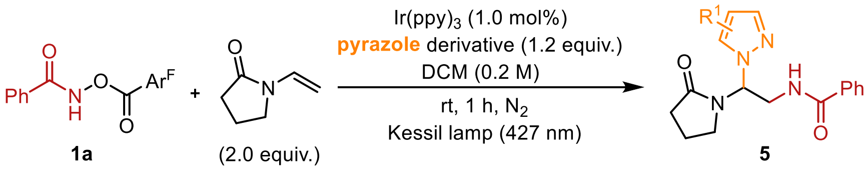

General Procedure C2 (1,2-Amidoamination) ***using pyrazoles*****.** To an oven-dried 4 mL vial with a stir bar were added Ir(ppy)$_3$ (1.0 mol%), *N*-[{3,5-bis(trifluoromethyl)benzoyl}oxy] benzamide (**1a**, 0.1

mmol, 1.0 equiv.), 1-vinylpyrrolidin-2-one (0.2 mmol, 2.0 equiv.), pyrazole derivative (0.12 mmol, 1.2 equiv.) and anhydrous DCM (0.2 M) under N_2 atmosphere. The vial was stirred at room temperature for 1 h under irradiation with a Kessil lamp (427 nm, maximum intensity), which was positioned 3~5 cm away from the reaction vial. Regular fans were equipped to maintain the temperature. The reaction mixture was poured into saturated $NaHCO_3$ aqueous solution (10 mL) and extracted with DCM (10 mL x 3). The combined organic layer was dried over anhydrous $MgSO_4$, filtered and evaporated under reduced pressure. Desired product was obtained by neutral alumina column chromatography (*n*-hexane/EtOAc, 1:1 ~ 1:3).

4. Formal *anti*-Markovnikov hydroamidation of alkenes

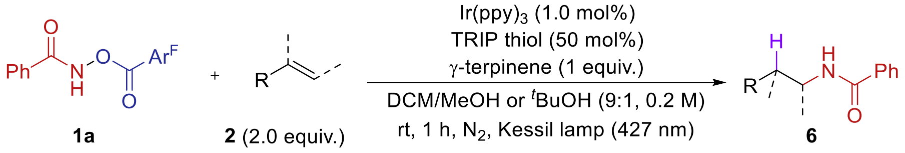

General Procedure D. To an oven-dried 4 mL vial with a stir bar were added $Ir(ppy)_3$ (1.0 mol%), *N*-[{3,5-bis(trifluoromethyl)benzoyl}oxy]benzamide (**1a**, 0.1 mmol, 1.0 equiv.), alkene substrate (**2**, 0.2 mmol, 2.0 equiv.), 2,4,6-triisopropylbenzenethiol (TRIP thiol, 50 mol%), γ-terpinene (0.1 mmol, 1.0 equiv.) and anhydrous DCM/MeOH or DCM/^{t}BuOH (9:1, 0.2 M) under N_2 atmosphere. The vial was stirred at room temperature for 1 h under irradiation with a Kessil lamp (427 nm, maximum intensity), which was positioned 3~5 cm away from the reaction vial. Regular fans were equipped to maintain the temperature. The reaction mixture was poured into saturated $NaHCO_3$ aqueous solution (10 mL) and extracted with DCM (10 mL x 3). The combined organic layer was dried over anhydrous $MgSO_4$, filtered and evaporated under reduced pressure. Desired product was obtained by silica gel column chromatography (Pet Ether/EtOAc, 4:1 ~ 1:1).

2-Benzamido-1-(4-methoxyphenyl)ethyl 3,5-bis(trifluoromethyl)benzoate (3a)

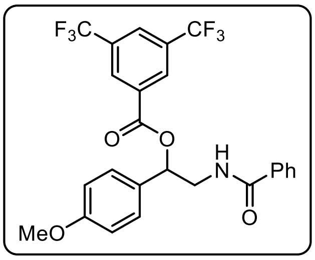

Synthesized according to the General Procedure A; colorless solid (34.1 mg, 67%); **m.p.** 137–139 °C; **^{1}H NMR** (500 MHz, $CDCl_3$) δ 8.48 (s, 2H), 8.05 (s, 1H), 7.73 – 7.67 (m, 2H), 7.52 – 7.45 (m, 1H), 7.45 – 7.35 (m, 4H), 6.97 – 6.91 (m, 2H), 6.50 (t, *J* = 6.0 Hz, 1H), 6.22 (dd, *J* = 8.2, 4.4 Hz, 1H), 4.08 (ddd, *J* = 14.3, 8.2, 6.1 Hz, 1H), 3.98 (ddd, *J* = 14.3, 5.9, 4.5 Hz, 1H), 3.81 (s, 3H); **^{13}C NMR** (126 MHz, $CDCl_3$) δ 167.8, 163.7, 160.2, 134.2, 132.4 (q, *J* = 34.0 Hz), 132.3, 131.9, 130.1 – 129.8 (m), 129.0, 128.8, 128.3, 127.0, 126.8 – 126.5 (m), 122.9 (q, *J* = 272.8 Hz), 114.5, 76.6, 55.5, 44.7; **^{19}F NMR** (471 MHz, $CDCl_3$) δ -62.9; **IR** (cm^{-1}) 3313, 1729, 1637, 1514, 1280, 1240, 1126, 1028, 914, 697, 681; **HRMS** (EI) m/z calcd. for $C_{25}H_{19}F_6NO_4$ $[M]^+$: 511.1218, found: 511.1216.

2-Benzamido-1-(4-ethoxyphenyl)ethyl 3,5-bis(trifluoromethyl)benzoate (3b)

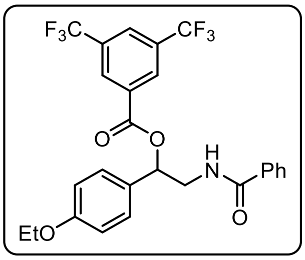

Synthesized according to the General Procedure A; colorless solid (33.1 mg, 63%); **m.p.** 159–161 °C; **^{1}H NMR** (600 MHz, $CDCl_3$) δ 8.48 (s, 2H), 8.05 (s, 1H), 7.70 (d, *J* = 7.9 Hz, 2H), 7.51 – 7.45 (m, 1H), 7.44 – 7.36 (m, 4H), 6.93 (d, *J* = 8.7 Hz, 2H), 6.49 (t, *J* = 4.8 Hz, 1H), 6.21 (dd, *J* = 8.1, 4.5 Hz, 1H), 4.12 – 3.94 (m, 4H), 1.41 (t, *J* = 7.0 Hz, 3H); **^{13}C NMR** (101 MHz, $CDCl_3$) δ 167.8, 163.7, 159.6, 134.2, 132.4 (q, *J* = 34.1 Hz), 132.3, 131.9, 130.0 (d, *J* = 3.0 Hz), 128.8(1), 128.7(7), 128.3, 127.0, 126.8 – 126.5 (m), 122.9 (q, *J* = 273.5 Hz), 115.0, 76.6, 63.7, 44.7, 14.9; **^{19}F NMR** (471 MHz, $CDCl_3$) δ -62.9; **IR** (cm^{-1}) 3313, 2916, 1727, 1639, 1516, 1282, 1242, 1172, 1129, 911, 697; **HRMS** (EI) m/z calcd. for $C_{26}H_{21}F_6NO_4$ $[M]^+$: 525.1375, found: 525.1378.

2-Benzamido-1-{4-(*tert*-butoxy)phenyl}ethyl 3,5-bis(trifluoromethyl)benzoate (3c)

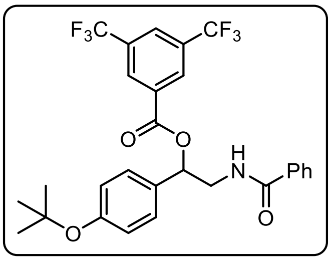

Synthesized according to the General Procedure A; colorless solid (35.8 mg, 65%); **m.p.** 130–132 °C; **^{1}H NMR** (600 MHz, $CDCl_3$) δ 8.50 (s, 2H), 8.06 (s, 1H), 7.68 (d, *J* = 8.0 Hz, 2H), 7.48 (t, *J* = 7.4 Hz, 1H), 7.39 (t, *J* = 7.9 Hz, 4H), 7.03 (d, *J* = 8.5 Hz, 2H), 6.44 (t, *J* = 5.4 Hz, 1H), 6.25 (dd, *J* = 8.0, 4.4 Hz, 1H), 4.06 (dt, *J* = 14.1, 7.0 Hz, 1H), 3.99 (dt, *J* = 14.3, 5.2 Hz, 1H), 1.36 (s, 9H); **^{13}C NMR** (101 MHz, $CDCl_3$) δ 167.8, 163.7, 156.3, 134.2, 132.4 (q, *J* = 34.1 Hz), 132.3, 131.9, 131.5, 130.0 (d, *J* = 3.0 Hz), 128.8, 127.5, 127.0, 126.8 – 126.6 (m), 124.4, 122.9 (q, *J* = 274.0 Hz), 79.1, 76.5, 44.8, 29.0; **^{19}F NMR** (376 MHz, $CDCl_3$) δ -62.9; **IR** (cm^{-1}) 3320, 2984, 2932, 1727, 1633, 1535, 1258, 1130, 901, 769, 680, 555; **HRMS** (EI) m/z calcd. for $C_{28}H_{25}F_6NO_4$ $[M]^+$: 553.1688, found: 553.1690.

2-Benzamido-1-(4-phenoxyphenyl)ethyl 3,5-bis(trifluoromethyl)benzoate (3d)

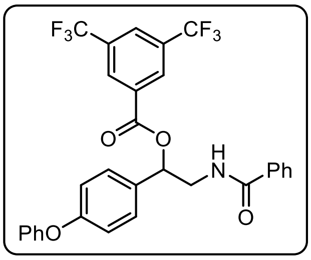

Synthesized according to the General Procedure A at 60 °C*;* colorless solid (35.7 mg, 62%); **m.p.** 143–145 °C; **^{1}H NMR** (600 MHz, $CDCl_3$) δ 8.50 (s, 2H), 8.06 (s, 1H), 7.71 (d, *J* = 8.1 Hz, 2H), 7.49 (t, *J* = 7.4 Hz, 1H), 7.45 (d, *J* = 8.4 Hz, 2H), 7.40 (t, *J* = 7.6 Hz, 2H), 7.35 (t, *J* = 7.6 Hz, 2H), 7.17 – 7.11 (m, 1H), 7.05 – 7.01 (m, 4H), 6.56 (t, *J* = 5.3 Hz, 1H), 6.25 (dd, *J* = 8.1, 4.4 Hz, 1H), 4.12 – 4.04 (m, 1H), 4.04 – 3.97 (m, 1H); **^{13}C NMR** (101 MHz, $CDCl_3$) δ 167.8, 163.7, 158.3, 156.6, 134.1, 132.4 (q, *J* = 34.3 Hz), 132.2, 132.0, 131.4, 130.1 – 129.9 (m), 129.4, 128.8, 128.4, 127.0, 126.9 – 126.6 (m), 124.0, 122.9 (q, *J* = 272.9 Hz), 119.6, 118.9, 76.4, 44.8; **^{19}F NMR** (376 MHz, $CDCl_3$) δ -62.9; **IR** (cm^{-1}) 3285, 1731, 1629, 1488, 1282, 1239, 1127, 914, 700, 532; **HRMS** (EI) m/z calcd. for $C_{30}H_{21}F_6NO_4$ [M]$^+$: 573.1375, found: 573.1372.

2-Benzamido-1-(2-methoxyphenyl)ethyl 3,5-bis(trifluoromethyl)benzoate (3e)

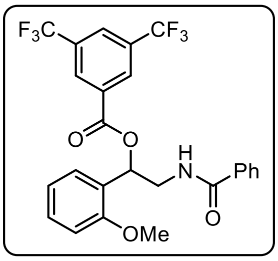

Synthesized according to the General Procedure A at 60 °C with 2.0 mol% of Ir(ppy)$_3$; colorless solid (18.4 mg, 36%); **m.p.** 160–162 °C; **^{1}H NMR** (500 MHz, $CDCl_3$) δ 8.52 (s, 2H), 8.06 (s, 1H), 7.73 – 7.67 (m, 2H), 7.52 – 7.46 (m, 1H), 7.46 – 7.38 (m, 3H), 7.35 (td, *J* = 8.3, 1.7 Hz, 1H), 7.05 – 6.99 (m, 1H), 6.96 (d, *J* = 8.2 Hz, 1H), 6.64 (dd, *J* = 7.0, 4.3 Hz, 1H), 6.48 (t, *J* = 5.3 Hz, 1H), 4.12 – 3.98 (m, 2H), 3.91 (s, 3H); **^{13}C NMR** (101 MHz, $CDCl_3$) δ 167.7, 163.7, 156.4, 134.4, 132.4(2), 132.3(8) (q, *J* = 34.1 Hz), 131.7, 130.0(9), 130.0(5), 128.7, 127.0, 126.8, 126.8 – 126.5 (m), 125.3, 123.0 (q, *J* = 273.0 Hz), 121.1, 111.0, 72.0, 55.7, 43.7; **^{19}F NMR** (471 MHz, $CDCl_3$) δ -62.9; **IR** (cm^{-1}) 3287, 1723, 1635, 1492, 1279, 1239, 1129, 1032, 912, 681; **HRMS** (EI) m/z calcd. for $C_{25}H_{19}F_6NO_4$ [M]$^+$: 511.1218, found: 511.1221.

2-Benzamido-1-(3,4-dimethoxyphenyl)ethyl 3,5-bis(trifluoromethyl)benzoate (3f)

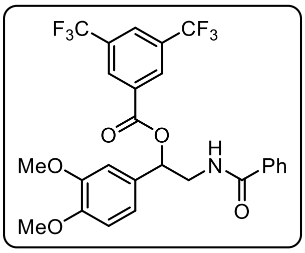

Synthesized according to the General Procedure A with 2.0 equiv. of **1a**; pale yellow solid (28.7 mg, 53%); **m.p.** 117–119 °C; **^{1}H NMR** (400 MHz, $CDCl_3$) δ 8.49 (s, 2H), 8.06 (s, 1H), 7.74 – 7.67 (m, 2H), 7.53 – 7.46 (m, 1H), 7.41 (t, *J* = 7.5 Hz, 2H), 7.05 (dd, *J* = 8.2, 2.0 Hz, 1H), 7.00 (d, *J* = 1.9 Hz, 1H), 6.90 (d, *J* = 8.3 Hz, 1H), 6.43 (t, *J* = 5.8 Hz, 1H), 6.21 (dd, *J* = 8.1, 4.6 Hz, 1H), 4.10 (ddd, *J* = 14.3, 8.1, 6.2 Hz, 1H), 3.99 (ddd, *J* = 14.3, 5.8, 4.7 Hz, 1H), 3.89 (d, *J* = 1.8 Hz, 6H); **^{13}C NMR** (101 MHz, $CDCl_3$) δ 167.8, 163.7, 149.7, 149.4, 134.1, 132.4 (q, *J* = 34.0 Hz), 132.3, 131.9, 130.0 (d, *J* = 2.8 Hz), 129.4, 128.8, 127.0, 126.8 – 126.6 (m), 122.9 (q, *J* = 274.1 Hz), 119.3, 111.5, 110.0, 76.7, 56.1(0), 56.0(7), 44.7; **^{19}F NMR** (471 MHz, $CDCl_3$) δ -62.9; **IR** (cm^{-1}) 3296, 2920, 2849, 1729, 1518, 1278, 1240, 1127, 1024, 911, 765, 681; **HRMS** (ESI) m/z calcd. for $C_{26}H_{21}F_6NO_5Na$ [M+Na]$^+$: 564.1221, found: 564.1220.

2-Benzamido-1-(benzo[*d*][1,3]dioxol-5-yl)ethyl 3,5-bis(trifluoromethyl)benzoate (3g)

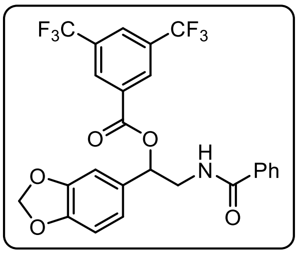

Synthesized according to the General Procedure A; colorless solid (20.7 mg, 39%); **m.p.** 170–172 °C; **^{1}H NMR** (500 MHz, $CDCl_3$) δ 8.48 (s, 2H), 8.06 (s, 1H), 7.73 – 7.67 (m, 2H), 7.52 – 7.45 (m, 1H), 7.44 – 7.37 (m, 2H), 7.01 – 6.93 (m, 2H), 6.87 – 6.80 (m, 1H), 6.44 (t, *J* = 6.0 Hz, 1H), 6.16 (dd, *J* = 8.1, 4.6 Hz, 1H), 5.98 (q, *J* = 1.5 Hz, 2H), 4.05 (ddd, *J* = 14.2, 8.1, 6.1 Hz, 1H), 3.96 (ddd, *J* = 14.2, 6.0, 4.6 Hz, 1H); **^{13}C NMR** (101 MHz, $CDCl_3$) δ 167.8, 163.6, 148.4(8), 148.4(5), 134.1, 132.4 (q, *J* = 34.2 Hz), 132.2, 131.9, 130.8, 130.2 – 129.7 (m), 128.8, 127.0, 126.9 – 126.6 (m), 122.9 (q, *J* = 274.0 Hz), 120.8, 108.8, 107.1, 101.6, 76.7, 44.8; **^{19}F NMR** (471 MHz, $CDCl_3$) δ -62.9; **IR** (cm^{-1}) 3268, 1733, 1628, 1488, 1278, 1243, 1124, 1031, 910, 696, 681; **HRMS** (EI) m/z calcd. for $C_{25}H_{17}F_6NO_5$ $[M]^+$: 525.1011, found: 525.1014.

2-Benzamido-1-(4-methoxyphenyl)propyl 3,5-bis(trifluoromethyl)benzoate (3h + 3h')

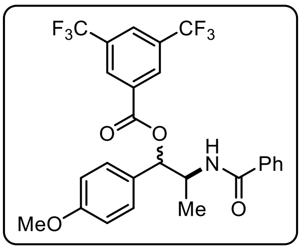

Synthesized according to the General Procedure A; colorless solid (24.6 mg, 47%); **m.p.** 76–78 °C; A 1.3:1 mixture of diastereomer was obtained and the ratio was determined by ^{1}H NMR spectroscopic analysis of the unpurified reaction mixture; **^{1}H NMR** (500 MHz, $CDCl_3$) δ 8.51 (s, 2H), 8.44 (s, 2.2H), 8.06 (s, 1H), 8.00 (s, 1.1H), 7.71 (d, *J* = 7.3 Hz, 2H), 7.67 (d, *J* = 7.3 Hz, 2.2H), 7.51 (t, *J* = 7.3 Hz, 1.1H), 7.48 – 7.40 (m, 5.2H), 7.40 – 7.33 (m, 4.2H), 6.97 – 6.91 (m, 4.2H), 6.24 (d, *J* = 9.0 Hz, 1.1H), 6.15 (d, *J* = 3.9 Hz, 1H), 6.08 (d, *J* = 8.9 Hz, 1H), 5.94 (d, *J* = 8.9 Hz, 1.1H), 4.96 – 4.84 (m, 2.1H), 3.82 (s, 3.3H), 3.81 (s, 3H), 1.29 (d, *J* = 6.9 Hz, 3H), 1.19 (d, *J* = 6.8 Hz, 3.3H); **^{13}C NMR** (126 MHz, $CDCl_3$) δ 167.1, 167.0, 164.1, 163.3, 160.3, 160.0, 134.4, 134.2, 132.5, 132.4 (q, *J* = 34.0 Hz), 132.3 (q, *J* = 34.3 Hz), 132.2, 131.8(5), 131.8(0), 130.1 – 129.8 (m, 2C), 129.1, 128.8(3), 128.7(5), 128.6, 128.3, 127.8, 126.9(4), 126.8(6), 126.8 – 126.5 (m, 2C), 123.0 (q, *J* = 273.8 Hz), 122.9 (q, *J* = 273.0 Hz), 114.4, 114.3, 80.6, 79.6, 55.5 (2C), 49.5, 48.6, 17.6, 16.6; **^{19}F NMR** (471 MHz, $CDCl_3$) δ -62.9, 63.0; **IR** (cm^{-1}) 3329, 1996, 1728, 1514, 1278, 1240, 1174, 1130, 911, 681; **HRMS** (EI) m/z calcd. for $C_{26}H_{21}F_6NO_4$ $[M]^+$: 525.1375, found: 525.1378.

2-Benzamido-1-(phenylthio)ethyl 3,5-bis(trifluoromethyl)benzoate (3i)

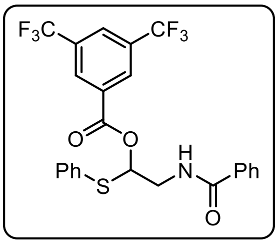

Synthesized according to the General Procedure A; yellow sticky oil (18.8 mg, 36%); **^{1}H NMR** (500 MHz, $CDCl_3$) δ 8.44 (s, 2H), 8.09 (s, 1H), 7.75 – 7.69 (m, 2H), 7.58 – 7.52 (m, 2H), 7.51 (tt, *J* = 6.7, 1.2 Hz, 1H), 7.43 (t, *J* = 7.6 Hz, 2H), 7.40 – 7.31 (m, 3H), 6.51 – 6.45 (m, 1H), 6.45 (t, *J* = 5.8 Hz, 1H), 4.04 – 3.91 (m, 2H); **^{13}C NMR** (101 MHz, $CDCl_3$) δ 167.7, 162.9, 134.8, 133.9, 132.5 (q, *J* = 34.4 Hz), 132.0, 131.8, 130.2 – 129.9 (m), 129.8, 129.5(3), 129.5(1), 128.9, 127.1, 127.3 – 126.7 (m), 122.9 (q, *J* = 274.2 Hz), 80.7, 43.1; **^{19}F NMR** (376 MHz, $CDCl_3$) δ -63.0; **IR** (cm^{-1})

3316, 3064, 2926, 1734, 1640, 1533, 1277, 1132, 911, 691; **HRMS** (EI) m/z calcd. for $C_{24}H_{17}F_6NO_3S$ $[M]^+$: 513.0833, found: 513.0829.

1-(4-Methoxyphenyl)-2-(4-methylbenzamido)ethyl 3,5-bis(trifluoromethyl)benzoate (3j)

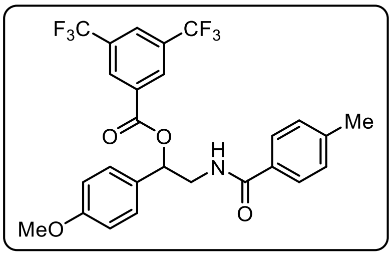

Synthesized according to the General Procedure A; colorless solid (36.2 mg, 69%); **m.p.** 162–164 °C; **^{1}H NMR** (500 MHz, $CDCl_3$) δ 8.48 (s, 2H), 8.05 (s, 1H), 7.60 (d, *J* = 8.0 Hz, 2H), 7.42 (d, *J* = 8.7 Hz, 2H), 7.19 (d, *J* = 7.9 Hz, 2H), 6.94 (d, *J* = 8.7 Hz, 2H), 6.45 (t, *J* = 6.1 Hz, 1H), 6.20 (dd, *J* = 8.2, 4.5 Hz, 1H), 4.07 (ddd, *J* = 14.3, 8.2, 6.1 Hz, 1H), 3.96 (ddd, *J* = 14.3, 5.9, 4.5 Hz, 1H), 3.81 (s, 3H), 2.37 (s, 3H); **^{13}C NMR** (101 MHz, $CDCl_3$) δ 167.7, 163.7, 160.2, 142.3, 132.4, 132.3 (q, *J* = 34.1 Hz), 131.3, 130.0 (d, *J* = 3.1 Hz), 129.4, 129.1, 128.3, 127.0, 126.8 – 126.5 (m), 122.9 (q, *J* = 274.0 Hz), 114.5, 76.6, 55.4, 44.7, 21.5; **^{19}F NMR** (471 MHz, $CDCl_3$) δ -62.9; **IR** (cm^{-1}) 3321, 2938, 1732, 1635, 1516, 1284, 1255, 1178, 1132, 1032, 915, 681; **HRMS** (EI) m/z calcd. for $C_{26}H_{21}F_6NO_4$ $[M]^+$: 525.1375, found: 525.1377.

2-{4-(*tert*-Butyl)benzamido}-1-(4-methoxyphenyl)ethyl 3,5-bis(trifluoromethyl)benzoate (3k)

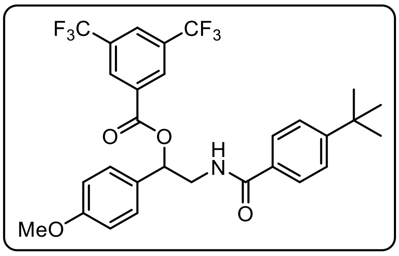

Synthesized according to the General Procedure A; colorless solid (34.8 mg, 61%); **m.p.** 146–148 °C; **^{1}H NMR** (400 MHz, $CDCl_3$) δ 8.48 (s, 2H), 8.05 (s, 1H), 7.68 – 7.60 (m, 2H), 7.47 – 7.38 (m, 4H), 6.98 – 6.88 (m, 2H), 6.40 (s, 1H), 6.21 (dd, *J* = 8.1, 4.6 Hz, 1H), 4.08 (ddd, *J* = 14.3, 8.1, 6.1 Hz, 1H), 4.02 – 3.92 (m, 1H), 3.82 (s, 3H), 1.31 (s, 9H); **^{13}C NMR** (101 MHz, $CDCl_3$) δ 167.7, 163.7, 160.2, 155.4, 132.36(8), 132.36(7) (q, *J* = 34.1 Hz), 131.3, 130.2 – 129.8 (m), 129.1, 128.3, 126.8, 126.8 – 126.5 (m), 125.7, 123.0 (q, *J* = 272.7 Hz), 114.5, 76.6, 55.5, 44.7, 35.1, 31.2; **^{19}F NMR** (376 MHz, $CDCl_3$) δ -62.9; **IR** (cm^{-1}) 3291, 2963, 1726, 1636, 1515, 1280, 1248, 1170, 1129, 913, 680; **HRMS** (ESI) m/z calcd. for $C_{29}H_{27}F_6NO_4Na$ $[M+Na]^+$: 590.1742, found: 590.1745.

2-(4-Chlorobenzamido)-1-(4-methoxyphenyl)ethyl 3,5-bis(trifluoromethyl)benzoate (3l)

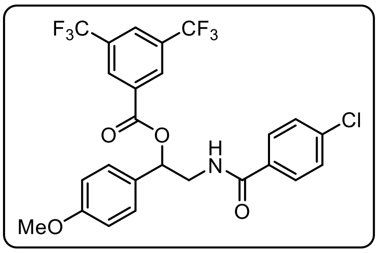

Synthesized according to the General Procedure A with 5 mol% of $Ir(ppy)_3$; pale yellow solid (23.5 mg, 43%); **m.p.** 143–145 °C; **^{1}H NMR** (400 MHz, $CDCl_3$) δ 8.47 (s, 2H), 8.06 (s, 1H), 7.63 (d, *J* = 8.1 Hz, 2H), 7.39 (dd, *J* = 16.2, 8.2 Hz, 4H), 6.94 (d, *J* = 8.3 Hz, 2H), 6.43 (t, *J* = 6.0 Hz, 1H), 6.20 (dd, *J* = 8.2, 4.5 Hz, 1H), 4.13 – 4.01 (m, 1H), 3.96 (dt, *J* = 14.3, 5.3 Hz, 1H), 3.82 (d, *J* = 1.3 Hz, 3H); **^{13}C NMR** (101 MHz, $CDCl_3$) δ 166.7, 163.7, 160.3, 138.2, 132.47(2), 132.47(0) (q, *J* = 34.3 Hz), 132.2, 130.2 – 129.8 (m), 129.1, 128.9, 128.4, 128.3, 126.9 – 126.6 (m), 122.9 (q, *J* = 274.0 Hz), 114.6, 76.5, 55.5, 44.9; **^{19}F NMR** (376 MHz, $CDCl_3$)

δ -62.9; **IR** (cm^{-1}) 3297, 1733, 1631, 1516, 1282, 1240, 1128, 916, 681, 507; **HRMS** (EI) m/z calcd. for $C_{25}H_{18}ClF_6NO_4$ $[M]^+$: 545.0829, found: 545.0832.

2-(4-Bromobenzamido)-1-(4-methoxyphenyl)ethyl 3,5-bis(trifluoromethyl)benzoate (3m)

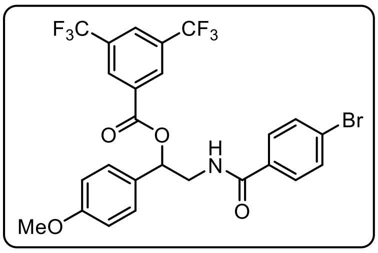

Synthesized according to the General Procedure A with 5 mol% of Ir(ppy)$_3$; pale yellow solid (24.9 mg, 42%); **m.p.** 149–151 °C; **^{1}H NMR** (500 MHz, $CDCl_3$) δ 8.47 (s, 2H), 8.06 (s, 1H), 7.55 (q, *J* = 8.7 Hz, 4H), 7.44 – 7.38 (m, 2H), 6.99 – 6.89 (m, 2H), 6.46 (t, *J* = 5.8 Hz, 1H), 6.20 (dd, *J* = 8.2, 4.4 Hz, 1H), 4.07 (ddd, *J* = 14.3, 8.2, 6.1 Hz, 1H), 3.95 (ddd, *J* = 14.3, 5.7, 4.6 Hz, 1H), 3.81 (s, 3H); **^{13}C NMR** (101 MHz, $CDCl_3$) δ 166.8, 163.7, 160.3, 132.9, 133.0 – 131.8 (m), 132.2, 132.0, 130.1 – 129.9 (m), 128.8, 128.6, 128.2, 126.9 – 126.6 (m), 126.6, 122.9 (q, *J* = 272.9 Hz), 114.6, 76.5, 55.5, 44.9; **^{19}F NMR** (471MHz, $CDCl_3$) δ -62.9; **IR** (cm^{-1}) 3292, 2158, 1731, 1631, 1515, 1283, 1242, 1129, 916, 846, 681; **HRMS** (ESI) m/z calcd. for $C_{25}H_{18}BrF_6NO_4Na$ $[M+Na]^+$: 612.0221, found: 612.0220.

1-(4-Methoxyphenyl)-2-(thiophene-3-carboxamido)ethyl 3,5-bis(trifluoromethyl)benzoate (3n)

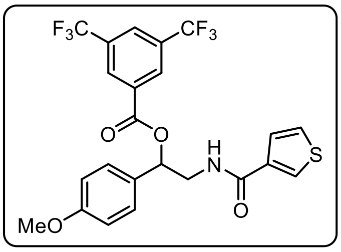

Synthesized according to the General Procedure A with 5 mol% of Ir(ppy)$_3$; pale yellow solid (30.6 mg, 59%); **m.p.** 119–121 °C; **^{1}H NMR** (600 MHz, $CDCl_3$) δ 8.48 (s, 2H), 8.05 (s, 1H), 7.83 – 7.79 (m, 1H), 7.44 – 7.39 (m, 2H), 7.34 – 7.29 (m, 2H), 6.97 – 6.92 (m, 2H), 6.24 (s, 1H), 6.19 (dd, *J* = 8.3, 4.5 Hz, 1H), 4.05 (ddd, *J* = 14.4, 8.2, 6.1 Hz, 1H), 3.94 (ddd, *J* = 14.4, 5.9, 4.5 Hz, 1H), 3.82 (s, 3H); **^{13}C NMR** (126 MHz, $CDCl_3$) δ 163.7, 163.3, 160.3, 137.1, 132.4 (q, *J* = 34.3 Hz), 132.3, 130.3 – 129.7 (m), 129.0, 128.6, 128.3, 126.9, 126.8 – 126.6 (m), 126.0, 122.9 (q, *J* = 273.4 Hz), 114.5, 76.6, 55.5, 44.5; **^{19}F NMR** (471 MHz, $CDCl_3$) δ -62.9; **IR** (cm^{-1}) 3314, 2940, 1728, 1630, 1512, 1280, 1236, 1124, 1023, 915, 821, 680, 536; **HRMS** (EI) m/z calcd. for $C_{23}H_{17}F_6NO_4S$ $[M]^+$: 517.0782, found: 517.0787.

2-(Cyclohexanecarboxamido)-1-(4-methoxyphenyl)ethyl 3,5-bis(trifluoromethyl)benzoate (3o)

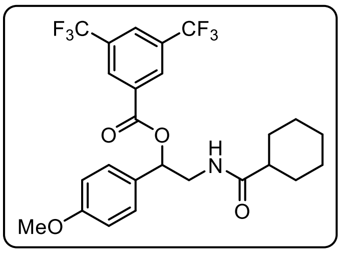

Synthesized according to the General Procedure A; colorless solid (24.4 mg, 47%); **m.p.** 150–152 °C; **^{1}H NMR** (500 MHz, $CDCl_3$) δ 8.48 (s, 2H), 8.07 (s, 1H), 7.37 (d, *J* = 8.7 Hz, 2H), 6.92 (d, *J* = 8.7 Hz, 2H), 6.09 (dd, *J* = 7.5, 5.0 Hz, 1H), 5.66 (t, *J* = 5.8 Hz, 1H), 3.88 – 3.76 (m, 5H), 2.03 (tt, *J* = 11.7, 3.1 Hz, 1H), 1.81 – 1.70 (m, 4H), 1.67 – 1.60 (m, 1H), 1.41 – 1.32 (m, 2H), 1.26 – 1.15 (m, 3H); **^{13}C NMR** (101 MHz, CD_3OD) δ 179.5, 164.5, 161.5, 134.1, 133.3 (q, *J* = 34.0 Hz), 131.1 – 130.8 (m), 130.7, 129.2, 127.8 – 127.5 (m), 124.4 (q, *J* = 271.9 Hz), 115.1, 77.7, 55.7, 46.3, 44.8, 30.7, 30.6, 26.8, 26.7(0), 26.6(8); **^{19}F NMR** (376 MHz, $CDCl_3$) δ -62.9;

IR (cm^{-1}) 2938, 2851, 2464, 1725, 1633, 1454, 1288, 1241, 1128, 914, 682, 534; **HRMS** (EI) m/z calcd. for $C_{25}H_{25}F_6NO_4$ [M]$^+$: 517.1688, found: 517.1685.

***N*-(2-Methoxy-2-phenylethyl)benzamide (4a)**

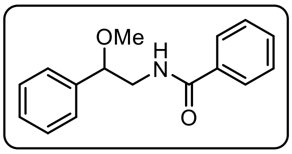

Synthesized according to the General Procedure B1; colorless solid (19.0 mg, 74%); **m.p.** 90–92 °C; **^{1}H NMR** (500 MHz, $CDCl_3$) δ 7.80 – 7.74 (m, 2H), 7.54 – 7.47 (m, 1H), 7.48 – 7.41 (m, 2H), 7.43 – 7.30 (m, 5H), 6.60 (s, 1H), 4.39 (dd, *J* = 8.8, 3.9 Hz, 1H), 3.94 (ddd, *J* = 13.9, 7.7, 3.9 Hz, 1H), 3.39 (ddd, *J* = 13.9, 8.8, 4.0 Hz, 1H), 3.29 (s, 3H); **^{13}C NMR** (101 MHz, $CDCl_3$) δ 167.5, 139.2, 134.7, 131.6, 128.8, 128.7, 128.4, 127.1, 126.8, 82.5, 57.1, 46.2; **IR** (cm^{-1}) 3328, 2923, 1632, 1538, 1451, 1113, 1022, 687, 556; **HRMS** (EI) m/z calcd. for $C_{16}H_{17}NO_2$ [M]$^+$: 255.1259, found: 255.1255.

***N*-{2-Methoxy-2-(*p*-tolyl)ethyl}benzamide (4b)**

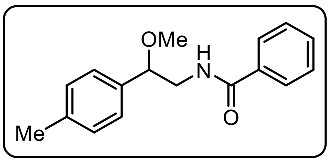

Synthesized according to the General Procedure B1; colorless solid (22.8 mg, 85%); **m.p.** 79–81 °C; **^{1}H NMR** (600 MHz, $CDCl_3$) δ 7.77 (d, *J* = 7.1 Hz, 2H), 7.50 (t, *J* = 7.4 Hz, 1H), 7.44 (t, *J* = 7.6 Hz, 2H), 7.24 (d, *J* = 7.8 Hz, 2H), 7.19 (d, *J* = 7.8 Hz, 2H), 6.61 (s, 1H), 4.35 (dd, *J* = 8.8, 4.0 Hz, 1H), 3.91 (ddd, *J* = 13.9, 7.7, 4.0 Hz, 1H), 3.39 (ddd, *J* = 13.2, 8.8, 3.9 Hz, 1H), 3.27 (s, 3H), 2.36 (s, 3H); **^{13}C NMR** (151 MHz, $CDCl_3$) δ 167.5, 138.1, 136.1, 134.8, 131.6, 129.5, 128.7, 127.1, 126.8, 82.3, 56.9, 46.2, 21.3; **IR** (cm^{-1}) 3250, 2923, 1629, 1545, 1261, 1110, 1029, 802, 697, 559; **HRMS** (EI) m/z calcd. for $C_{17}H_{19}NO_2$ [M]$^+$: 269.1416, found: 269.1417.

***N*-[2-{4-(*tert*-Butyl)phenyl}-2-methoxyethyl]benzamide (4c)**

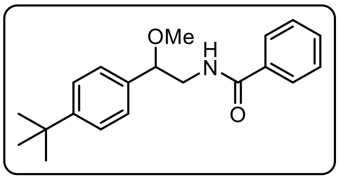

Synthesized according to the General Procedure B1; colorless solid (27.4 mg, 88%); **m.p.** 86–88 °C; **^{1}H NMR** (600 MHz, $CDCl_3$) δ 7.80 – 7.75 (m, 2H), 7.53 – 7.47 (m, 1H), 7.44 (t, *J* = 7.6 Hz, 2H), 7.40 (d, *J* = 8.3 Hz, 2H), 7.28 (d, *J* = 8.3 Hz, 2H), 6.61 (s, 1H), 4.36 (dd, *J* = 8.9, 3.9 Hz, 1H), 3.93 (ddd, *J* = 13.9, 7.7, 3.9 Hz, 1H), 3.38 (ddd, *J* = 14.0, 8.9, 3.9 Hz, 1H), 3.28 (s, 3H), 1.33 (s, 9H); **^{13}C NMR** (126 MHz, $CDCl_3$) δ 167.5, 151.3, 136.0, 134.7, 131.6, 128.7, 127.1, 126.5, 125.7, 82.2, 57.0, 46.2, 34.7, 31.5; **IR** (cm^{-1}) 3325, 2948, 1636, 1540, 1271, 1107, 827, 690, 580; **HRMS** (EI) m/z calcd. for $C_{20}H_{25}NO_2$ [M]$^+$: 311.1885, found: 311.1888.

***N*-{2-Methoxy-2-(4-methoxyphenyl)ethyl}benzamide (4d)**[13]

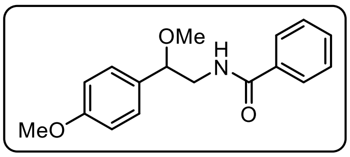

Synthesized according to the General Procedure B1; colorless solid (27.0 mg, 95%); **m.p.** 93–95 °C; **^{1}H NMR** (400 MHz, $CDCl_3$) δ 7.80 – 7.73 (m, 2H), 7.54 – 7.47 (m, 1H), 7.43 (tt, *J* = 6.8, 1.1 Hz, 2H), 7.30 – 7.24 (m, 2H), 6.96 – 6.87 (m, 2H), 6.60 (s, 1H), 4.33 (dd, *J* = 8.8, 4.0 Hz, 1H), 3.89 (ddd, *J* = 13.9, 7.7, 4.1 Hz, 1H), 3.82 (s, 3H), 3.45 – 3.33 (m, 1H), 3.25 (s, 3H); **^{13}C NMR** (101 MHz, $CDCl_3$) δ 167.4, 159.7, 134.7, 131.6, 131.1, 128.7, 128.1, 127.1, 114.2, 82.0, 56.8, 55.4, 46.2; **IR** (cm^{-1}) 3307, 2030, 1969, 1638, 1509, 1238, 1031, 693; **HRMS** (EI) m/z calcd. for $C_{17}H_{19}NO_3$ $[M]^+$: 285.1365, found: 285.1364.

4-(2-Benzamido-1-methoxyethyl)phenyl acetate (4e)

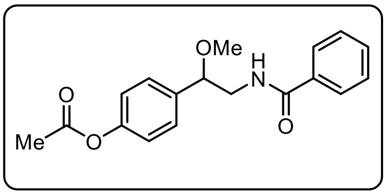

Synthesized according to the General Procedure B1; colorless solid (22.6 mg, 72%); **m.p.** 97–99 °C; **^{1}H NMR** (600 MHz, $CDCl_3$) δ 7.79 – 7.74 (m, 2H), 7.53 – 7.47 (m, 1H), 7.44 (t, *J* = 7.5 Hz, 2H), 7.37 (d, *J* = 8.5 Hz, 2H), 7.11 (d, *J* = 8.5 Hz, 2H), 6.61 (s, 1H), 4.39 (dd, *J* = 8.8, 3.9 Hz, 1H), 3.90 (ddd, J = 13.9, 7.7, 4.0 Hz, 1H), 3.36 (ddd, J = 14.1, 8.8, 4.0 Hz, 1H), 3.28 (s, 3H), 2.30 (s, 3H); **^{13}C NMR** (151 MHz, $CDCl_3$) δ 169.5, 167.5, 150.6, 136.8, 134.6, 131.6, 128.7, 127.8, 127.1, 121.9, 81.9, 57.1, 46.3, 21.3; **IR** (cm^{-1}) 3326, 2924, 2852, 1753, 1632, 1537, 1197, 1112, 1013, 911, 691, 555; **HRMS** (EI) m/z calcd. for $C_{18}H_{19}NO_4$ $[M]^+$: 313.1314, found: 313.1314.

***N*-{2-(4-Fluorophenyl)-2-methoxyethyl}benzamide (4f)**

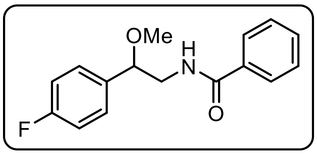

Synthesized according to the General Procedure B1; colorless solid (19.3 mg, 71%); **m.p.** 97–99 °C; **^{1}H NMR** (400 MHz, $CDCl_3$) δ 7.81 – 7.73 (m, 2H), 7.55 – 7.46 (m, 1H), 7.48 – 7.39 (m, 2H), 7.37 – 7.28 (m, 2H), 7.07 (tt, *J* = 8.7, 2.5 Hz, 2H), 6.59 (s, 1H), 4.37 (dd, *J* = 8.9, 3.7 Hz, 1H), 3.90 (ddd, *J* = 13.9, 7.7, 3.9 Hz, 1H), 3.36 (ddd, *J* = 13.9, 8.8, 4.0 Hz, 1H), 3.26 (s, 3H); **^{13}C NMR** (101 MHz, $CDCl_3$) δ 167.5, 162.7 (d, *J* = 246.3 Hz), 135.0 (d, *J* = 3.2 Hz), 134.6, 131.7, 128.7, 128.5 (d, *J* = 8.2 Hz), 127.1, 115.7 (d, *J* = 21.5 Hz), 81.8, 57.0, 46.2; **^{19}F NMR** (376 MHz, $CDCl_3$) δ -114.0; **IR** (cm^{-1}) 3336, 2923, 1632, 1536, 1506, 1221, 1114, 1028, 834, 687, 553; **HRMS** (EI) m/z calcd. for $C_{16}H_{16}FNO_2$ $[M]^+$: 273.1165, found: 273.1169.

***N*-{2-Methoxy-2-(*o*-tolyl)ethyl}benzamide (4g)**

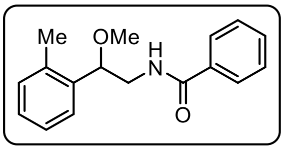

Synthesized according to the General Procedure B1; colorless solid (19.3 mg, 72%); **m.p.** 87–89 °C; **^{1}H NMR** (400 MHz, $CDCl_3$) δ 7.80 (dd, *J* = 6.9, 1.7 Hz, 2H), 7.56 – 7.48 (m, 1H), 7.48 – 7.41 (m, 2H), 7.42 – 7.36 (m, 1H), 7.29 – 7.15 (m, 3H), 6.70 (s, 1H), 4.66 (dd, *J* = 9.0, 3.4 Hz, 1H), 3.97 (ddd, *J* = 14.1, 7.9, 3.4 Hz, 1H), 3.27

(s, 3H), 3.27 – 3.20 (m, 1H), 2.43 (s, 3H); **^{13}C NMR** (101 MHz, $CDCl_3$) δ 167.5, 137.1, 136.2, 134.6, 131.6, 130.8, 128.7, 127.9, 127.1, 126.4, 125.7, 79.4, 57.0, 45.4, 19.1; **IR** (cm^{-1}) 3288, 2922, 1737, 1628, 1542, 1260, 1108, 1025, 802, 760, 692, 569; **HRMS** (EI) m/z calcd. for $C_{17}H_{19}NO_2$ $[M]^+$: 269.1416, found: 269.1420.

***N*-{2-(2-Bromophenyl)-2-methoxyethyl}benzamide (4h)**

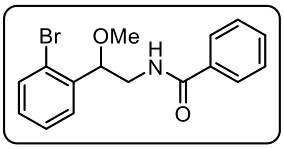

Synthesized according to the General Procedure B1; colorless solid (16.5 mg, 49%); **m.p.** 119–121 °C; **^{1}H NMR** (500 MHz, $CDCl_3$) δ 7.81 – 7.75 (m, 2H), 7.58 (dd, *J* = 8.0, 1.2 Hz, 1H), 7.53 – 7.48 (m, 1H), 7.49 – 7.41 (m, 3H), 7.36 (td, *J* = 7.5, 1.2 Hz, 1H), 7.19 (td, *J* = 7.6, 1.8 Hz, 1H), 6.54 (s, 1H), 4.83 (dd, *J* = 8.1, 3.9 Hz, 1H), 4.03 (ddd, *J* = 13.9, 7.3, 3.9 Hz, 1H), 3.39 (ddd, *J* = 13.9, 8.1, 4.2 Hz, 1H), 3.32 (s, 3H); **^{13}C NMR** (126 MHz, $CDCl_3$) δ 167.4, 138.2, 134.7, 133.3, 131.6, 129.7, 128.7, 127.9, 127.8, 127.1, 123.5, 81.2, 57.3, 44.6; **IR** (cm^{-1}) 3323, 2922, 1628, 1541, 1115, 1021, 759, 718, 565; **HRMS** (EI) m/z calcd. for $C_{16}H_{16}BrNO_2$ $[M]^+$: 333.0364, found: 333.0361.

***N*-{2-Methoxy-2-(3-methoxyphenyl)ethyl}benzamide (4i)**

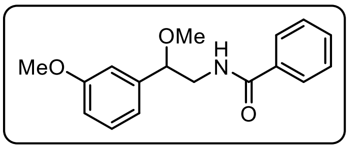

Synthesized according to the General Procedure B1; yellow oil (15.3 mg, 54%); **^{1}H NMR** (500 MHz, $CDCl_3$) δ 7.80 – 7.74 (m, 2H), 7.54 – 7.45 (m, 1H), 7.44 (td, *J* = 7.6, 7.1, 1.8 Hz, 2H), 7.30 (t, *J* = 7.8 Hz, 1H), 6.97 – 6.84 (m, 3H), 6.60 (s, 1H), 4.36 (dd, *J* = 8.8, 3.9 Hz, 1H), 3.93 (ddd, *J* = 13.9, 7.7, 4.0 Hz, 1H), 3.81 (s, 3H), 3.38 (ddd, *J* = 13.9, 8.8, 4.0 Hz, 1H), 3.29 (s, 3H); **^{13}C NMR** (126 MHz, $CDCl_3$) δ 167.5, 160.1, 140.9, 134.7, 131.6, 129.9, 128.7, 127.1, 119.2, 113.9, 112.0, 82.4, 57.1, 55.4, 46.2; **IR** (cm^{-1}) 3331, 2932, 2832, 1638, 1533, 1486, 1259, 1107, 1040, 785, 695; **HRMS** (EI) m/z calcd. for $C_{17}H_{19}NO_3$ $[M]^+$: 285.1365, found: 285.1366.

***N*-{2-Methoxy-2-(thiophen-2-yl)ethyl}benzamide (4j)**

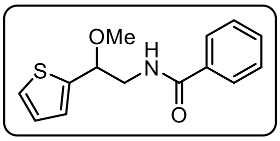

Synthesized according to the General Procedure B1; colorless oil (12.5 mg, 48%); **^{1}H NMR** (400 MHz, $CDCl_3$) δ 7.77 (d, *J* = 8.1 Hz, 2H), 7.51 (t, *J* = 7.3 Hz, 1H), 7.44 (t, *J* = 7.2 Hz, 2H), 7.32 (d, *J* = 4.9 Hz, 1H), 7.06 (d, *J* = 3.2 Hz, 1H), 7.03 – 6.98 (m, 1H), 6.58 (s, 1H), 4.67 (dd, *J* = 8.0, 4.3 Hz, 1H), 3.96 (ddd, *J* = 13.7, 7.1, 4.3 Hz, 1H), 3.60 (ddd, *J* = 12.8, 8.0, 4.6 Hz, 1H), 3.33 (s, 3H); **^{13}C NMR** (101 MHz, $CDCl_3$) δ 167.5, 142.6, 134.6, 131.7, 128.7, 127.1, 126.9, 126.3, 125.8, 78.0, 57.0, 46.1; **IR** (cm^{-1}) 3309, 2929, 2824, 1638, 1532, 1284, 1103, 1080, 692; **HRMS** (EI) m/z calcd. for $C_{14}H_{15}NO_2S$ $[M]^+$: 261.0823, found: 261.0827.

N-(2-Methoxy-2-phenylpropyl)benzamide (4k)

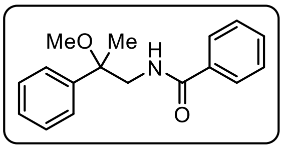

Synthesized according to the General Procedure B1; colorless oil (23.2 mg, 86%); **^{1}H NMR** (500 MHz, $CDCl_3$) δ 7.77 – 7.69 (m, 2H), 7.53 – 7.46 (m, 1H), 7.46 – 7.35 (m, 6H), 7.34 – 7.28 (m, 1H), 6.47 (s, 1H), 3.88 (dd, *J* = 13.7, 7.2 Hz, 1H), 3.53 (dd, *J* = 13.7, 4.3 Hz, 1H), 3.16 (s, 3H), 1.62 (s, 3H); **^{13}C NMR** (126 MHz, $CDCl_3$) δ 167.6, 142.6, 134.8, 131.5, 128.7, 128.7, 127.7, 127.0, 126.3, 79.0, 50.7, 49.8, 21.0; **IR** (cm^{-1}) 3322, 2935, 2826, 1643, 1523, 1487, 1070, 763, 698, 564; **HRMS** (EI) m/z calcd. for $C_{17}H_{19}NO_2$ $[M]^+$: 269.1416, found: 269.1419.

N-(2-Methoxy-2,2-diphenylethyl)benzamide (4l)

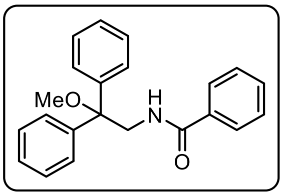

Synthesized according to the General Procedure B1; colorless solid (28.9 mg, 87%); **m.p.** 139–141 °C; **^{1}H NMR** (500 MHz, CD_2Cl_2) δ 7.58 – 7.52 (m, 2H), 7.50 – 7.41 (m, 5H), 7.41 – 7.32 (m, 6H), 7.30 – 7.21 (m, 2H), 6.14 (s, 1H), 4.37 (d, *J* = 5.0 Hz, 2H), 3.16 (s, 3H); **^{13}C NMR** (101 MHz, $CDCl_3$) δ 167.7, 142.9, 134.7, 131.5, 128.6, 128.5, 127.7, 126.9, 126.9, 81.7, 50.8, 43.5; **IR** (cm^{-1}) 3421, 1651, 1479, 1072, 751, 699, 534; **HRMS** (EI) m/z calcd. for $C_{22}H_{21}NO_2$ $[M]^+$: 331.1572, found: 331.1570.

N-(1-Methoxy-1-phenylpropan-2-yl)benzamide (4m + 4m')

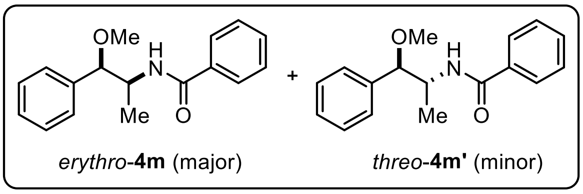

Synthesized according to the General Procedure B1; a 4:1 mixture of *erythro*-**4m** and *threo*-**4m'** was obtained and the ratio was determined by ^{1}H NMR spectroscopic analysis of the unpurified reaction mixture; Colorless solid (18.0 mg, 67%);

Major diastereomer (*erythro*): **m.p.** 119–121 °C; **^{1}H NMR** (400 MHz, $CDCl_3$) δ 7.82 – 7.77 (m, 2H), 7.54 – 7.49 (m, 1H), 7.48 – 7.42 (m, 2H), 7.42 – 7.34 (m, 4H), 7.34 – 7.28 (m, 1H), 6.51 (d, *J* = 8.9 Hz, 1H), 4.47 (d, *J* = 3.3 Hz, 1H), 4.46 – 4.37 (m, 1H), 3.36 (s, 3H), 1.07 (d, *J* = 6.8 Hz, 3H); **^{13}C NMR** (101 MHz, $CDCl_3$) δ 166.7, 138.6, 135.0, 131.5, 128.7, 128.6, 127.8, 127.1, 126.9, 85.4, 57.9, 50.4, 14.0; **IR** (cm^{-1}) 3333, 2923, 1634, 1525, 1453, 1091, 689, 634, 567; **HRMS** (EI) m/z calcd. for $C_{17}H_{19}NO_2$ $[M]^+$: 269.1416, found: 269.1417;

Minor diastereomer (*threo*): **m.p.** 147–149 °C; **^{1}H NMR** (400 MHz, $CDCl_3$) δ 7.72 – 7.67 (m, 2H), 7.52 – 7.44 (m, 1H), 7.45 – 7.37 (m, 2H), 7.39 – 7.31 (m, 2H), 7.31 – 7.27 (m, 3H), 6.22 (d, *J* = 8.3 Hz, 1H), 4.49 – 4.37 (m, 1H), 4.32 (d, *J* = 4.0 Hz, 1H), 3.34 (s, 3H), 1.28 (d, *J* = 6.8 Hz, 3H); **^{13}C NMR** (101 MHz, $CDCl_3$) δ 166.9, 138.7, 135.0, 131.4, 128.7, 128.5, 128.1, 127.2, 127.0, 85.1, 57.6, 50.4, 17.5; **IR** (cm^{-1}) 3320, 2920, 2851, 1630, 1543, 1259, 1091, 1026, 799, 699, 567; **HRMS** (FAB) m/z calcd. for $C_{17}H_{20}NO_2$ $[M+H]^+$: 270.1494, found: 270.1498.

The structure of ***erythro*-4m** was assigned by an X-ray crystallography (SCXRD) analysis.

***N*-(2-Methoxy-2-phenylcyclohexyl)benzamide (4n + 4n')**

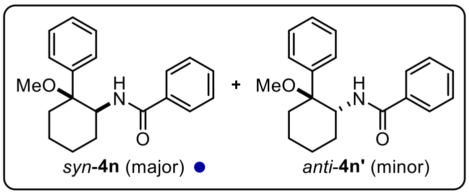

Synthesized according to the General Procedure B1; colorless solid (19.5 mg, 63%); **m.p.** 138–140 °C; an inseparable mixture of *syn*-**4n** and *anti*-**4n'** (1.3:1) was obtained and the ratio was determined by ^{1}H NMR spectroscopic analysis of the unpurified reaction mixture; **^{1}H NMR** (400 MHz, $CDCl_3$) δ 7.59 – 7.52 (m, 2.8H), 7.46 (t, *J* = 6.3 Hz, 2H), 7.43 – 7.24 (m, 16.8H), 7.23 (t, *J* = 7.3 Hz, 1H), 7.18 (t, *J* = 7.3 Hz, 1.4H), 6.48 (d, *J* = 9.1 Hz, 1.4H), 5.83 (d, *J* = 8.3 Hz, 1H), 4.51 (dd, *J* = 8.6, 4.2 Hz, 1H), 4.06 (ddd, *J* = 11.5, 9.4, 4.5 Hz, 1.4H), 3.26 (s, 4.2H), 2.97 (s, 3H), 2.33 – 2.13 (m, 3.4H), 2.14 – 2.02 (m, 1.4H), 1.96 – 1.47 (m, 13.4H), 1.44 – 1.30 (m, 1H); **^{13}C NMR** (151 MHz, $CDCl_3$) δ 166.6, 166.3, 142.2, 142.0, 135.4, 135.3, 131.2, 131.0, 128.5(3), 128.4(5), 128.2, 127.9, 127.2, 126.9(5), 126.8(6), 126.7, 126.5, 80.2, 78.6, 56.9, 53.6, 50.1, 49.7, 31.0, 29.8, 29.3, 26.8, 26.2, 25.1, 21.1, 20.8, 20.4; **IR** (cm^{-1}) 3330, 3061, 2934, 2850, 1632, 1522, 1488, 1262, 1077, 1019, 690, 565; **HRMS** (EI) m/z calcd. for $C_{20}H_{23}NO_2$ $[M]^+$: 309.1729, found: 309.1732.

***N*-[2-Methoxy-2-{(8*R*,9*S*,13*S*,14*S*)-13-methyl-17-oxo-7,8,9,11,12,13,14,15,16,17-decahydro-6*H*-cyclopenta[*a*]phenanthren-3-yl}ethyl]benzamide (4o)**

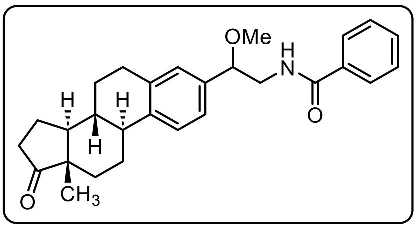

Synthesized according to the General Procedure B1; an inseparable equal mixture of two diastereomers obtained, determined by DEPT-45 ^{13}C NMR spectroscopic analysis of the isolated product; colorless solid (30.6 mg, 71%); **m.p.** 72–74 °C; **^{1}H NMR** (600 MHz, $CDCl_3$) δ 7.80 – 7.76 (m, 2H), 7.51 (t, *J* = 7.4 Hz, 1H), 7.44 (t, *J* = 7.6 Hz, 2H), 7.33 – 7.28 (m, 1H), 7.14 (dt, *J* = 8.0, 2.2 Hz, 1H), 7.08 (s, 1H), 6.61 (s, 1H), 4.33 (ddd, *J* = 8.8, 3.9, 2.1 Hz, 1H), 3.93 (m, 1H), 3.39 – 3.31 (m, 1H), 3.29 (s, 3H), 2.93 (dd, *J* = 9.2, 4.3 Hz, 2H), 2.51 (dd, *J* = 19.0, 8.8 Hz, 1H), 2.47 – 2.40 (m, 1H), 2.32 (td, *J* = 11.0, 4.2 Hz, 1H), 2.15 (dt, *J* = 18.6, 8.9 Hz, 1H), 2.11 – 2.00 (m, 2H), 2.01 – 1.95 (m, 1 H), 1.69 – 1.58 (m, 2H), 1.59 – 1.42 (m, 4H), 0.92 (s, 3H); **^{13}C NMR** (151 MHz, $CDCl_3$) δ 220.9, 167.5, 139.9, 136.9(8)[a], 136.9(6)[a], 136.6, 134.8, 131.6, 128.7, 127.5[a], 127.4[a], 127.1, 125.8, 124.2[a], 124.1[a], 82.4[a], 82.3[a], 57.1, 50.7, 48.1, 46.2(9)[a], 46.2(5)[a], 44.6, 38.2, 36.0, 31.8, 29.6[a], 29.5[a], 26.6(3)[a], 26.6(1)[a], 25.8(5)[a], 25.8(1)[a], 21.7, 14.0; **IR** (cm^{-1}) 2961, 2923, 2854, 1734, 1639, 1532, 1259, 1083, 1023, 797, 710; **HRMS** (FAB) m/z calcd. for $C_{28}H_{34}NO_3$ $[M+H]^+$: 432.2539, found: 432.2537.

[a]Distinct two signals were shown for specific carbons due to the presence of diastereomers.

Methyl (2*S*)-3-{4-(2-benzamido-1-methoxyethyl)phenyl}-2-{(*tert*-butoxycarbonyl)amino} propanoate (4p)

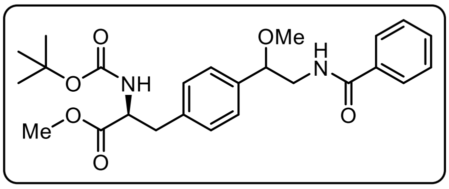

Synthesized according to the General Procedure B1; an inseparable equal mixture of two diastsreomers obtained, determined by ^{13}C NMR spectroscopic analysis of the isolated product; colorless solid (34.8 mg, 76%); **m.p.** 108–110 °C; **^{1}H NMR** (400 MHz, $CDCl_3$) δ 7.80 – 7.73 (m, 2H), 7.55 – 7.46 (m, 1H), 7.47 – 7.39 (m, 2H), 7.28 (d, *J* = 7.7 Hz, 2H), 7.15 (d, *J* = 7.6 Hz, 2H), 6.60 (s, 1H), 5.01 (s, 1H), 4.59 (q, *J* = 7.0 Hz, 1H), 4.36 (dd, *J* = 8.8, 3.9 Hz, 1H), 3.96 – 3.84 (m, 1H), 3.70 (s, 3H), 3.42 – 3.29 (m, 1H), 3.26 (s, 3H), 3.18 – 2.96 (m, 2H), 1.40 (s, 9H); **^{13}C NMR** (101 MHz, $CDCl_3$) δ 172.4(2)[a], 172.3(9)[a], 167.4, 155.2, 137.9, 136.2, 134.6, 131.6, 129.7, 128.7, 127.1, 127.0, 82.2, 80.1, 57.0, 54.5, 52.4, 46.2, 38.3, 28.4; **IR** (cm^{-1}) 3359, 2933, 1749, 1686, 1526, 1167, 1107, 1053, 694, 562; **HRMS** (EI) m/z calcd. for $C_{25}H_{32}N_2O_6$ $[M]^+$: 456.2260, found: 456.2263.

[a]Distinct two signals were shown for specific carbons due to the presence of diastereomers.

***N*-{2-Methoxy-2-(2-oxopyrrolidin-1-yl)ethyl}benzamide (4q)**

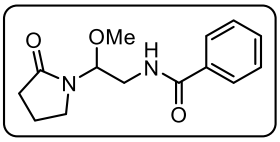

Synthesized according to the General Procedure B2; yellow solid (21.2 mg, 81%); **m.p.** 100–102 °C; **^{1}H NMR** (400 MHz, $CDCl_3$) δ 7.76 – 7.69 (m, 2H), 7.52 – 7.43 (m, 1H), 7.43 – 7.36 (m, 2H), 6.61 (s, 1H), 5.32 (dd, *J* = 7.3, 5.7 Hz, 1H), 3.95 (dt, *J* = 13.7, 7.6 Hz, 1H), 3.56 – 3.32 (m, 3H), 3.29 (s, 3H), 2.51 – 2.28 (m, 2H), 2.01 (p, *J* = 7.1 Hz, 2H); **^{13}C NMR** (101 MHz, $CDCl_3$) δ 177.1, 167.7, 134.4, 131.7, 128.7, 127.1, 81.5, 56.1, 41.7, 41.0, 31.7, 18.5; **IR** (cm^{-1}) 3335, 2928, 1669, 1535, 1260, 1081, 1023, 799, 694, 630; **HRMS** (EI) m/z calcd. for $C_{14}H_{18}N_2O_3$ $[M]^+$: 262.1317, found: 262.1315.

***N*-{2-Methoxy-2-(2-oxoazepan-1-yl)ethyl}benzamide (4r)**

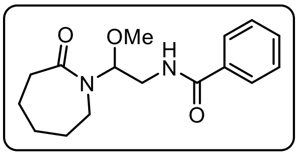

Synthesized according to the General Procedure B2; colorless oil (21.3 mg, 73%); **^{1}H NMR** (500 MHz, CD_2Cl_2) δ 7.77 – 7.71 (m, 2H), 7.53 – 7.46 (m, 1H), 7.47 – 7.39 (m, 2H), 6.66 (s, 1H), 5.73 (dd, *J* = 7.1, 6.4 Hz, 1H), 3.74 (dt, *J* = 13.4, 7.3 Hz, 1H), 3.42 – 3.31 (m, 2H), 3.32 – 3.23 (m, 4H), 2.58 (ddd, *J* = 13.7, 10.3, 1.6 Hz, 1H), 2.53 – 2.43 (m, 1H), 1.78 – 1.64 (m, 4H), 1.56 (m, 2H); **^{13}C NMR** (151 MHz, $CDCl_3$) δ 178.5, 167.5, 134.3, 131.6, 128.7, 127.1, 83.3, 56.6, 42.1, 41.4, 37.8, 30.1, 29.2, 23.7; **IR** (cm^{-1}) 3327, 2927, 2854, 1627, 1538, 1184, 1077, 694, 567; **HRMS** (FAB) m/z calcd. for $C_{16}H_{23}N_2O_3$ $[M+H]^+$: 291.1709, found: 291.1706.

N-{1-Methoxy-1-(2-oxopyrrolidin-1-yl)propan-2-yl}benzamide (4s + 4s')

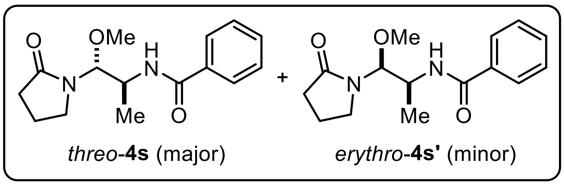

Synthesized according to the General Procedure B2; an inseparable mixture of *threo*-**4s** and *erythro*-**4s'** (2:1) was obtained, and the ratio was determined by ^{1}H NMR spectroscopic analysis of the unpurified reaction mixture;

Major diastereomer (*threo*): Colorless solid (11.7 mg, 42%); **m.p.** 166–168 °C; **^{1}H NMR** (400 MHz, $CDCl_3$) δ 7.81 – 7.77 (m, 2H), 7.54 – 7.48 (m, 1H), 7.47 – 7.41 (m, 2H), 6.68 (d, *J* = 8.7 Hz, 1H), 5.19 (d, *J* = 4.0 Hz, 1H), 4.42 – 4.33 (m, 1H), 3.45 – 3.33 (m, 2H), 3.32 (s, 3H), 2.49 – 2.40 (m, 2H), 2.08 – 1.94 (m, 2H), 1.28 (d, *J* = 6.7 Hz, 3H); **^{13}C NMR** (101 MHz, $CDCl_3$, *one carbon merged to others*) δ 176.8, 166.7, 134.7, 131.7, 128.7, 127.1, 84.3, 56.4, 48.2, 42.2, 31.3, 18.7 (2C); **IR** (cm^{-1}) 3334, 2926, 1680, 1650, 1538, 1287, 1086, 690, 580; **HRMS** (FAB) m/z calcd. for $C_{15}H_{21}N_2O_3$ $[M+H]^+$: 277.1552, found: 277.1554;

Minor diastereomer (*erythro*): Colorless solid (4.0 mg, 14%); **m.p.** 130–132 °C; **^{1}H NMR** (400 MHz, $CDCl_3$) δ 7.73 – 7.67 (m, 2H), 7.51 – 7.45 (m, 1H), 7.44 – 7.37 (m, 2H), 6.21 (d, *J* = 9.7 Hz, 1H), 4.95 (d, *J* = 9.5 Hz, 1H), 4.45 (tq, *J* = 9.6, 6.6 Hz, 1H), 3.60 – 3.50 (m, 1H), 3.39 – 3.30 (m, 4H), 2.42 (ddd, *J* = 17.0, 8.6, 6.7 Hz, 1H), 2.24 (ddd, *J* = 16.9, 9.2, 7.5 Hz, 1H), 2.01 – 1.90 (m, 2H), 1.35 (d, *J* = 6.6 Hz, 3H); **^{13}C NMR** (101 MHz, $CDCl_3$) δ 177.7, 167.2, 134.5, 131.7, 128.8, 127.0, 86.6, 56.6, 46.4, 42.0, 32.0, 18.6, 18.1; **IR** (cm^{-1}) 3314, 2925, 1673, 1650, 1545, 1268, 1087, 694, 640; **HRMS** (EI) m/z calcd. for $C_{15}H_{20}N_2O_3$ $[M]^+$: 276.1474, found: 276.1476.

The structure of *threo*-**4s** was assigned by X-ray crystallography analysis.

N-{2-(Ethylthio)-2-methoxyethyl}benzamide (4t)

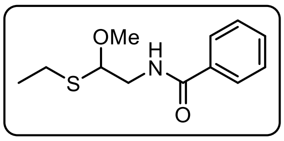

Synthesized according to the General Procedure B2; colorless solid (16.3 mg, 68%); **m.p.** 39–41 °C; **^{1}H NMR** (600 MHz, $CDCl_3$ δ 7.81 – 7.76 (m, 2H), 7.54 – 7.48 (m, 1H), 7.46 – 7.42 (m, 2H), 6.54 (s, 1H), 4.51 (dd, *J* = 7.8, 5.6 Hz, 1H), 3.85 (ddd, *J* = 13.9, 6.4, 5.5 Hz, 1H), 3.71 (ddd, *J* = 13.9, 7.8, 5.3 Hz, 1H), 3.45 (s, 3H), 2.63 (q, *J* = 7.5 Hz, 2H), 1.29 (t, *J* = 7.5 Hz, 3H); **^{13}C NMR** (151 MHz, $CDCl_3$) δ 167.5, 134.5, 131.7, 128.7, 127.1, 86.0, 56.1, 44.1, 22.7, 15.7; **IR** (cm^{-1}) 3325, 2927, 2822, 1633, 1530, 1313, 1110, 1075, 692, 663; **HRMS** (EI) m/z calcd. for $C_{12}H_{17}NO_2S$ $[M]^+$: 239.0980, found: 239.0978.

N-{2-Methoxy-2-(phenylthio)ethyl}benzamide (4u)

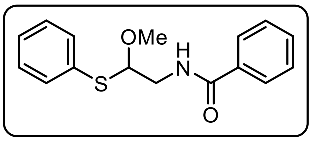

Synthesized according to the General Procedure B2; colorless oil (22.7 mg, 79%); **^{1}H NMR** (500 MHz, $CDCl_3$) δ 7.74 (d, *J* = 7.4 Hz, 2H), 7.55 – 7.47 (m, 3H), 7.43 (t, *J* = 7.7 Hz, 2H), 7.36 – 7.28 (m, 3H), 6.49 (s, 1H), 4.74 (dd, *J* = 7.7, 5.1 Hz, 1H), 3.88 (ddd, *J* = 13.8, 6.7, 5.0 Hz, 1H), 3.59 – 3.50 (m, 4H); **^{13}C NMR** (101

MHz, $CDCl_3$) δ 167.5, 134.4(2), 134.3(8), 131.7, 131.6, 129.2, 128.7, 128.3, 127.1, 88.7, 56.7, 44.1; **IR** (cm^{-1}) 3314, 3060, 2927, 1639, 1532, 1286, 1112, 1070, 690; **HRMS** (FAB) m/z calcd. for $C_{16}H_{18}NO_2S$ $[M+H]^+$: 288.1058, found: 288.1056.

***N*-(2-Ethoxy-2-methoxyethyl)benzamide (4v)**

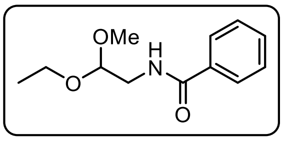

Synthesized according to the General Procedure B2; colorless oil (15.1 mg, 68%); **^{1}H NMR** (400 MHz, $CDCl_3$) δ 7.81 – 7.74 (m, 2H), 7.53 – 7.47 (m, 1H), 7.4 8 – 7.39 (m, 2H), 6.40 (s, 1H), 4.56 (t, *J* = 5.3 Hz, 1H), 3.75 (dq, *J* = 9.5, 7.0 Hz, 1H), 3.68 – 3.52 (m, 3H), 3.43 (s, 3H), 1.24 (t, *J* = 7.0 Hz, 3H); **^{13}C NMR** (126 MHz, $CDCl_3$) δ 167.7, 134.5, 131.7, 128.7, 127.1, 101.9, 63.4, 54.6, 42.1, 15.5; **IR** (cm^{-1}) 3329, 2932, 1639, 1537, 1291, 1125, 1061, 693, 535; **HRMS** (EI) m/z calcd. for $C_{12}H_{17}NO_3$ $[M]^+$: 223.1208, found: 223.1205.

***N*-{2-(Cyclohexyloxy)-2-methoxyethyl}benzamide (4w)**

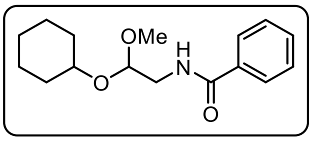

Synthesized according to the General Procedure B2; colorless solid (20.5 mg, 74%); **m.p.** 66–68 °C; **^{1}H NMR** (500 MHz, $CDCl_3$) δ 7.80 – 7.74 (m, 2H), 7.54 – 7.47 (m, 1H), 7.46 – 7.41 (m, 2H), 6.39 (s, 1H), 4.67 – 4.61 (m, 1H), 3.62 (ddd, *J* = 13.8, 6.2, 4.9 Hz, 1H), 3.58 – 3.49 (m, 2H), 3.40 (s, 3H), 1.98 – 1.86 (m, 2H), 1.81 – 1.69 (m, 2H), 1.58 – 1.49 (m, 1H), 1.40 (m, 1H), 1.36 – 1.14 (m, 4H); **^{13}C NMR** (126 MHz, $CDCl_3$) δ 167.6, 134.5, 131.7, 128.7, 127.0, 100.4, 76.5, 54.1, 42.9, 33.2, 32.9, 25.6, 24.4, 24.2; **IR** (cm^{-1}) 3331, 2925, 2855, 1636, 1541, 1294, 1127, 1055, 719, 696; **HRMS** (FAB) m/z calcd. for $C_{15}H_{20}NO_2$ $[M-CH_3OH+H]^+$: 246.1494, found: 246.1492.

***N*-(2-Methoxy-2-phenoxyethyl)benzamide (4x)**

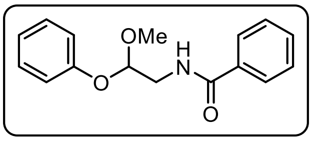

Synthesized according to the General Procedure B2; colorless oil (12.3 mg, 45%); **^{1}H NMR** (500 MHz, $CDCl_3$) δ 7.81 – 7.75 (m, 2H), 7.54 – 7.49 (m, 1H), 7.47 – 7.41 (m, 2H), 7.35 – 7.28 (m, 2H), 7.11 – 7.05 (m, 2H), 7.07 – 7.00 (m, 1H), 6.48 (s, 1H), 5.35 – 5.28 (m, 1H), 3.89 (ddd, *J* = 14.0, 6.4, 4.9 Hz, 1H), 3.72 (dt, *J* = 14.0, 5.8 Hz, 1H), 3.47 (s, 3H); **^{13}C NMR** (126 MHz, $CDCl_3$) δ 167.9, 157.3, 134.2, 131.9, 129.9, 128.8, 127.1, 122.7, 117.3, 101.4, 55.2, 42.6; **IR** (cm^{-1}) 3313, 3062, 2936, 2838, 1638, 1535, 1488, 1226, 1031, 690; **HRMS** (FAB) m/z calcd. for $C_{16}H_{18}NO_3$ $[M+H]^+$: 272.1287, found: 272.1288.

N-(2-Methoxytetrahydrofuran-3-yl)benzamide (4y + 4y’)

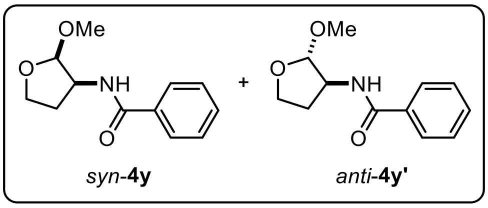

Synthesized according to the General Procedure B2; colorless solid (7.6 mg, 34%); **m.p.** 148–150 °C; an inseparable mixture of *syn*-**4y** and *anti*-**4y’** (1:1) was obtained and the ratio was determined by ^{1}H NMR spectroscopic analysis of the unpurified reaction mixture; **^{1}H NMR** (500 MHz, $CDCl_3$) δ 7.81 – 7.77 (m, 2H), 7.76 – 7.73 (m, 2H), 7.53 – 7.48 (m, 2H), 7.47 – 7.41 (m, 4H), 6.64 (d, *J* = 6.6 Hz, 1H), 6.08 (d, *J* = 7.0 Hz, 1H), 4.93 – 4.88 (m, 2H), 4.67 – 4.56 (m, 2H), 4.11 – 3.93 (m, 4H), 3.43 (s, 3H), 3.37 (s, 3H), 2.56 – 2.42 (m, 2H), 1.92 – 1.79 (m, 2H); **^{13}C NMR** (126 MHz, $CDCl_3$) δ 167.2(4), 167.1(6), 134.4, 134.3, 131.8, 131.7, 128.8, 128.7, 127.1(2), 127.0(6), 107.8, 101.7, 66.1, 65.9, 55.4, 54.9, 54.7, 52.4, 30.5, 29.9; **IR** (cm^{-1}) 3319, 2961, 2922, 1733, 1636, 1527, 1488, 1259, 1019, 799, 692; **HRMS** (EI) m/z calcd. for $C_{12}H_{15}NO_3$ $[M]^+$: 221.1052, found: 221.1047.

N-{2-Ethoxy-2-(2-oxopyrrolidin-1-yl)ethyl}benzamide (4z)

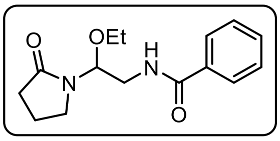

Synthesized according to the General Procedure B2; yellow solid (19.1 mg, 69%); **m.p.** 142–144 °C; **^{1}H NMR** (400 MHz, $CDCl_3$) δ 7.77 – 7.68 (m, 2H), 7.51 – 7.44 (m, 1H), 7.44 – 7.35 (m, 2H), 6.59 (s, 1H), 5.42 (dd, *J* = 7.6, 5.6 Hz, 1H), 3.98 (dt, *J* = 13.7, 7.8 Hz, 1H), 3.57 – 3.33 (m, 5H), 2.48 – 2.28 (m, 2H), 2.07 – 1.90 (m, 2H), 1.20 (t, *J* = 7.0 Hz, 3H); **^{13}C NMR** (101 MHz, $CDCl_3$) δ 177.0, 167.7, 134.4, 131.7, 128.7, 127.1, 79.9, 64.0, 41.8, 41.1, 31.8, 18.5, 15.0; **IR** (cm^{-1}) 3370, 2973, 2894, 1662, 1533, 1427, 1290, 1091, 1070, 720, 563; **HRMS** (EI) m/z calcd. for $C_{15}H_{20}N_2O_3$ $[M]^+$: 276.1474, found: 276.1477.

N-{2-Butoxy-2-(2-oxopyrrolidin-1-yl)ethyl}benzamide (4aa)

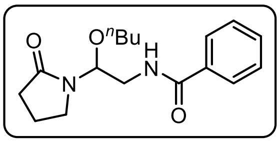

Synthesized according to the General Procedure B2; yellow sticky oil (21.7 mg, 71%); **m.p.** 114–116 °C; **^{1}H NMR** (500 MHz, $CDCl_3$) δ 7.75 – 7.70 (m, 2H), 7.51 – 7.46 (m, 1H), 7.44 – 7.39 (m, 2H), 6.44 (d, *J* = 4.5 Hz, 1H), 5.40 (dd, *J* = 7.5, 5.7 Hz, 1H), 3.98 (dt, *J* = 13.7, 7.7 Hz, 1H), 3.54 – 3.34 (m, 5H), 2.48 – 2.30 (m, 2H), 2.07 – 1.94 (m, 2H), 1.60 – 1.52 (m, 2H), 1.44 – 1.31 (m, 2H), 0.92 (t, *J* = 7.4 Hz, 3H); **^{13}C NMR** (126 MHz, $CDCl_3$) δ 176.9, 167.7, 134.5, 131.7, 128.8, 127.0, 80.1, 68.4, 41.8, 41.2, 31.8, 31.6, 19.5, 18.6, 14.0; **IR** (cm^{-1}) 3312, 2930, 2871, 1669, 1539, 1421, 1287, 1087, 695; **HRMS** (FAB) m/z calcd. for $C_{17}H_{25}N_2O_3$ $[M+H]^+$: 305.1865, found: 305.1861.

N-{2-Isopropoxy-2-(2-oxopyrrolidin-1-yl)ethyl}benzamide (4ab)

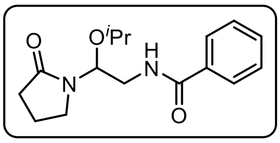

Synthesized according to the General Procedure B2; colorless solid (17.6 mg, 61%); **m.p.** 116–118 °C; **^{1}H NMR** (500 MHz, $CDCl_3$) δ 7.77 – 7.68 (m, 2H), 7.52 – 7.45 (m, 1H), 7.45 – 7.38 (m, 2H), 6.47 (s, 1H), 5.51 (dd, *J* = 7.5, 5.8

Hz, 1H), 3.97 (dt, *J* = 13.6, 7.8 Hz, 1H), 3.68 (hept, *J* = 6.2 Hz, 1H), 3.55 – 3.46 (m, 1H), 3.42 (ddd, *J* = 9.8, 7.8, 5.7 Hz, 1H), 3.36 (ddd, *J* = 13.7, 5.8, 3.7 Hz, 1H), 2.45 – 2.29 (m, 2H), 2.05 – 1.94 (m, 2H), 1.18 (dd, *J* = 16.4, 6.2 Hz, 6H); **^{13}C NMR** (151 MHz, $CDCl_3$) δ 176.8, 167.7, 134.5, 131.7, 128.8, 127.0, 77.8, 69.7, 41.9, 41.4, 31.8, 23.1, 21.6, 18.6; **IR** (cm^{-1}) 3330, 2974, 1649, 1545, 1434, 1288, 1080, 714, 693; **HRMS** (FAB) m/z calcd. for $C_{16}H_{23}N_2O_3$ $[M+H]^+$: 291.1709, found: 291.1705.

***N*-{2-(*tert*-Butoxy)-2-(2-oxopyrrolidin-1-yl)ethyl}benzamide (4ac)**

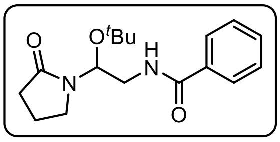

Synthesized according to the General Procedure B2; colorless solid (15.5 mg, 51%); **m.p.** 158–160 °C; **^{1}H NMR** (500 MHz, $CDCl_3$) δ 7.74 – 7.69 (m, 2H), 7.51 – 7.44 (m, 1H), 7.41 (t, *J* = 7.6 Hz, 2H), 6.42 (s, 1H), 5.61 (dd, *J* = 8.2, 5.5 Hz, 1H), 3.92 (dt, *J* = 13.6, 8.1 Hz, 1H), 3.56 – 3.44 (m, 2H), 3.27 (ddd, *J* = 13.6, 5.6, 3.8 Hz, 1H), 2.30 (t, *J* = 8.1 Hz, 2H), 2.01 – 1.92 (m, 2H), 1.23 (s, 9H); **^{13}C NMR** (151 MHz, $CDCl_3$) δ 175.5, 167.6, 134.6, 131.7, 128.8, 127.0, 75.6, 74.4, 42.3, 41.9, 32.1, 28.2, 18.6; **IR** (cm^{-1}) 3348, 2971, 2914, 2857, 1650, 1550, 1437, 1291, 1086, 695; **HRMS** (FAB) m/z calcd. for $C_{17}H_{25}N_2O_3$ $[M+H]^+$: 305.1865, found: 305.1863.

***N*-(2-Methoxy-2-phenylethyl)-4-methylbenzamide (4ad)**

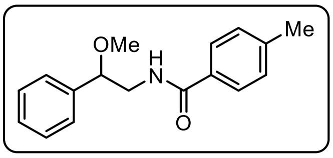

Synthesized according to the General Procedure B1; colorless solid (18.5 mg, 69%); **m.p.** 118–120 °C; **^{1}H NMR** (600 MHz, $CDCl_3$) δ 7.67 (d, *J* = 7.9 Hz, 2H), 7.41 – 7.30 (m, 5H), 7.24 (d, *J* = 7.8 Hz, 2H), 6.57 (s, 1H), 4.38 (dd, *J* = 8.7, 3.8 Hz, 1H), 3.92 (ddd, *J* = 13.6, 7.6, 3.9 Hz, 1H), 3.38 (ddd, *J* = 13.3, 8.8, 3.9 Hz, 1H), 3.28 (s, 3H), 2.40 (s, 3H); **^{13}C NMR** (151 MHz, $CDCl_3$) δ 167.4, 142.0, 139.3, 131.9, 129.3, 128.8, 128.3, 127.1, 126.8, 82.5, 57.0, 46.2, 21.6; **IR** (cm^{-1}) 3258, 2924, 2814, 1624, 1540, 1504, 1453, 1114, 700, 561; **HRMS** (ESI) m/z calcd. for $C_{17}H_{19}NO_2Na$ $[M+Na]^+$: 292.1313, found: 292.1309.

4-Chloro-*N*-(2-methoxy-2-phenylethyl)benzamide (4ae)

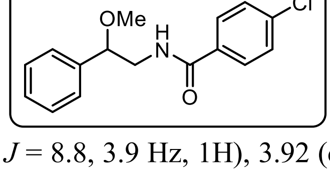

Synthesized according to the General Procedure B1; colorless solid (17.5 mg, 60%); **m.p.** 119–121 °C; **^{1}H NMR** (500 MHz, $CDCl_3$) δ 7.75 – 7.68 (m, 2H), 7.44 – 7.36 (m, 4H), 7.37 – 7.29 (m, 3H), 6.56 (s, 1H), 4.38 (dd, *J* = 8.8, 3.9 Hz, 1H), 3.92 (ddd, *J* = 13.9, 7.7, 3.9 Hz, 1H), 3.38 (ddd, *J* = 14.0, 8.8, 3.9 Hz, 1H), 3.28 (s, 3H); **^{13}C NMR** (126 MHz, $CDCl_3$) δ 166.4, 139.0, 137.8, 133.0, 129.0, 128.8, 128.5, 128.4, 126.8, 82.4, 57.0, 46.3; **IR** (cm^{-1}) 3246, 2924, 2852, 2174, 1628, 1542, 1485, 1261, 1090, 1014, 756, 699, 563; **HRMS** (EI) m/z calcd. for $C_{16}H_{16}ClNO_2$ $[M]^+$: 289.0870, found: 289.0871.

4-Methoxy-*N*-(2-methoxy-2-phenylethyl)benzamide (4af)[13]

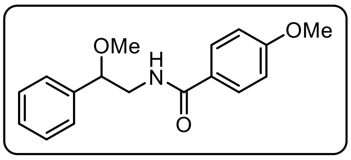

Synthesized according to the General Procedure B1; yellow solid (15.4 mg, 54%); **m.p.** 83–85 °C; **^{1}H NMR** (600 MHz, $CDCl_3$) δ 7.75 – 7.72 (m, 2H), 7.41 – 7.30 (m, 5H), 6.96 – 6.90 (m, 2H), 6.50 (s, 1H), 4.38 (dd, *J* = 8.8, 4.0 Hz, 1H), 3.91 (ddd, *J* = 13.9, 7.7, 4.0 Hz, 1H), 3.85 (s, 3H), 3.37 (ddd, *J* = 14.0, 8.8, 4.0 Hz, 1H), 3.28 (s, 3H); **^{13}C NMR** (126 MHz, $CDCl_3$) δ 167.0, 162.3, 139.3, 128.9, 128.8, 128.3, 127.0, 126.8, 113.9, 82.6, 57.0, 55.5, 46.2; **IR** (cm^{-1}) 3313, 2927, 1629, 1505, 1254, 1108, 1024, 841, 698, 553; **HRMS** (EI) m/z calcd. for $C_{17}H_{19}NO_3$ $[M]^+$: 285.1365, found: 285.1367.

***N*-(2-Methoxy-2-phenylethyl)thiophene-3-carboxamide (4ag)**

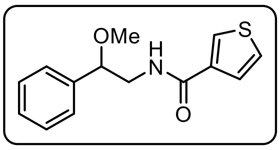

Synthesized according to the General Procedure B1; colorless solid (10.6 mg, 41%); **m.p.** 106–108 °C; **^{1}H NMR** (500 MHz, $CDCl_3$) δ 7.86 (dd, *J* = 2.9, 1.2 Hz, 1H), 7.42 – 7.30 (m, 7H), 6.41 (s, 1H), 4.37 (dd, *J* = 8.8, 3.9 Hz, 1H), 3.90 (ddd, *J* = 13.9, 7.8, 3.9 Hz, 1H), 3.34 (ddd, *J* = 13.8, 8.9, 4.0 Hz, 1H), 3.28 (s, 3H); **^{13}C NMR** (126 MHz, $CDCl_3$) δ 163.1, 139.2, 137.7, 128.8, 128.4, 128.3, 126.8, 126.6, 126.2, 82.5, 57.0, 46.0; **IR** (cm^{-1}) 3301, 3081, 2920, 1736, 1621, 1551, 1262, 1111, 1026, 748, 698, 558; **HRMS** (ESI) m/z calcd. for $C_{14}H_{15}NO_2SNa$ $[M+Na]^+$: 284.0721, found: 284.0727.

***N*-(2-Methoxy-2-phenylethyl)cyclohexanecarboxamide (4ah)**

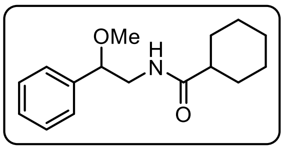

Synthesized according to the General Procedure B1; colorless solid (9.6 mg, 37%); **m.p.** 54–56 °C; **^{1}H NMR** (500 MHz, $CDCl_3$) δ 7.40 – 7.33 (m, 2H), 7.33 – 7.27 (m, 3H), 5.87 (s, 1H), 4.25 (dd, *J* = 8.6, 4.1 Hz, 1H), 3.68 (ddd, *J* = 13.9, 7.6, 4.0 Hz, 1H), 3.26 (s, 3H), 3.20 (ddd, *J* = 13.8, 8.7, 4.1 Hz, 1H), 2.07 (tt, *J* = 11.8, 3.5 Hz, 1H), 1.90 – 1.75 (m, 4H), 1.49 – 1.35 (m, 2H), 1.33 – 1.15 (m, 4H); **^{13}C NMR** (126 MHz, $CDCl_3$) δ 176.2, 139.3, 128.7, 128.3, 126.8, 82.5, 57.0, 45.7, 45.5, 29.8(0), 29.7(7), 25.9; **IR** (cm^{-1}) 3311, 2928, 2849, 1637, 1546, 1257, 1087, 1027, 800, 698; **HRMS** (EI) m/z calcd. for $C_{16}H_{23}NO_2$ $[M]^+$: 261.1729, found: 261.1732.

***N*-(2-Azido-2-phenylethyl)benzamide (5a)**

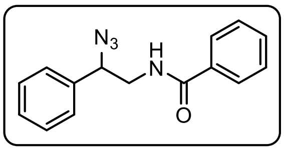

Synthesized according to the General Procedure C1; colorless solid (16.5 mg, 62%); **m.p.** 75–77 °C; **^{1}H NMR** (400 MHz, $CDCl_3$) δ 7.80 – 7.72 (m, 2H), 7.57 – 7.48 (m, 1H), 7.48 – 7.33 (m, 7H), 6.47 (s, 1H), 4.85 (dd, *J* = 9.0, 4.8 Hz, 1H), 3.90 (ddd, *J* = 13.9, 7.1, 4.9 Hz, 1H), 3.48 (ddd, *J* = 13.9, 9.0, 5.1 Hz, 1H); **^{13}C NMR** (101 MHz, $CDCl_3$) δ 167.7, 137.1, 134.2, 131.9, 129.2, 129.0, 128.8, 127.1(0), 127.0(8), 65.4, 45.4; **IR** (cm^{-1}) 3316, 2922, 2091, 1639, 1528, 1487, 1243, 690, 662; **HRMS** (FAB) m/z calcd. for $C_{15}H_{15}N_4O$ $[M+H]^+$: 267.1246, found: 267.1248.

_N_-[2-Azido-2-{4-(_tert_-butyl)phenyl}ethyl]benzamide (5b)

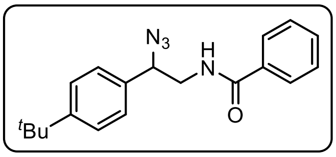

Synthesized according to the General Procedure C1; colorless sticky oil (14.2 mg, 44%); **^{1}H NMR** (500 MHz, $CDCl_3$) δ 7.78 – 7.74 (m, 2H), 7.55 – 7.48 (m, 1H), 7.48 – 7.40 (m, 4H), 7.33 – 7.29 (m, 2H), 6.49 (s, 1H), 4.82 (dd, *J* = 9.1, 4.7 Hz, 1H), 3.90 (ddd, *J* = 13.9, 7.2, 4.8 Hz, 1H), 3.46 (ddd, *J* = 13.9, 9.1, 5.0 Hz, 1H), 1.33 (s, 9H); **^{13}C NMR** (126 MHz, $CDCl_3$) δ 167.8, 152.0, 134.3, 134.1, 131.9, 128.8, 127.1, 126.8, 126.1, 65.2, 45.4, 34.8, 31.4; **IR** (cm^{-1}) 3309, 2961, 2927, 2099, 1638, 1533, 1279, 1242, 829, 692, 572; **HRMS** (FAB) m/z calcd. for $C_{19}H_{23}N_4O$ $[M+H]^+$: 323.1872, found: 323.1875.

_N_-{2-Azido-2-(4-chlorophenyl)ethyl}benzamide (5c)

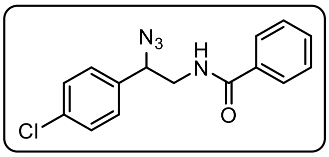

Synthesized according to the General Procedure C1; colorless solid (16.7 mg, 56%); **m.p.** 151–153 °C; **^{1}H NMR** (400 MHz, $CDCl_3$) δ 7.82 – 7.75 (m, 2H), 7.60 – 7.51 (m, 1H), 7.50 – 7.44 (m, 2H), 7.44 – 7.39 (m, 2H), 7.37 – 7.31 (m, 2H), 6.52 (s, 1H), 4.87 (dd, *J* = 8.9, 4.7 Hz, 1H), 3.89 (ddd, *J* = 13.8, 7.0, 4.7 Hz, 1H), 3.45 (ddd, *J* = 14.0, 8.9, 5.1 Hz, 1H); **^{13}C NMR** (101 MHz, CDCl3) δ 167.8, 135.7, 134.8, 134.1, 132.0, 129.4, 128.9, 128.4, 127.1, 64.8, 45.5; **IR** (cm^{-1}) 3302, 2103, 1637, 1530, 1311, 1260, 1092, 819, 693, 518; **HRMS** (FAB) m/z calcd. for $C_{15}H_{14}ClN_4O$ $[M+H]^+$: 301.0856, found: 301.0859.

_N_-{2-Azido-2-(3-bromophenyl)ethyl}benzamide (5d)

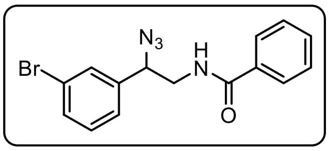

Synthesized according to the General Procedure C1; colorless solid (21.9 mg, 63%); **m.p.** 102–104 °C; **^{1}H NMR** (600 MHz, $CDCl_3$) δ 7.78 – 7.74 (m, 2H), 7.57 – 7.48 (m, 3H), 7.45 (t, *J* = 7.7 Hz, 2H), 7.34 – 7.26 (m, 2H), 6.48 (s, 1H), 4.84 (dd, *J* = 9.0, 4.6 Hz, 1H), 3.88 (ddd, *J* = 13.8, 7.0, 4.6 Hz, 1H), 3.41 (ddd, *J* = 14.0, 9.0, 5.1 Hz, 1H); **^{13}C NMR** (126 MHz, $CDCl_3$) δ 167.8, 139.6, 134.0, 132.1, 132.0, 130.8, 130.1, 128.9, 127.1, 125.8, 123.3, 64.8, 45.6; **IR** (cm^{-1}) 3322, 2096, 1639, 1530, 1314, 1261, 786, 691; **HRMS** (FAB) m/z calcd. for $C_{15}H_{14}BrN_4O$ $[M+H]^+$: 345.0351, found: 345.0354.

_N_- {2-Azido-2-(_o_-tolyl)ethyl}benzamide (5e)

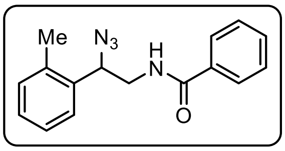

Synthesized according to the General Procedure C1; colorless oil (17.9 mg, 64%); **^{1}H NMR** (400 MHz, $CDCl_3$) δ 7.84 – 7.78 (m, 2H), 7.59 – 7.50 (m, 1H), 7.51 – 7.43 (m, 2H), 7.45 – 7.38 (m, 1H), 7.34 – 7.20 (m, 3H), 6.61 (s, 1H), 5.16 (dd, *J* = 9.4, 4.1 Hz, 1H), 3.95 (ddd, *J* = 14.0, 7.1, 4.1 Hz, 1H), 3.35 (ddd, *J* = 14.1, 9.3, 4.9 Hz, 1H), 2.50 (s, 3H); **^{13}C NMR** (101 MHz, $CDCl_3$) δ 167.9, 136.1, 135.3, 134.2, 131.9, 131.2, 128.8, 128.6, 127.1, 126.8, 126.2, 62.2, 44.9, 19.4; **IR** (cm^{-1}) 3313, 2925, 2097, 1636, 1530, 1241, 692; **HRMS** (FAB) m/z calcd. for $C_{16}H_{17}N_4O$ $[M+H]^+$: 281.1402, found: 281.1401.

N-{2-Azido-2-(naphthalen-2-yl)ethyl}benzamide (5f)

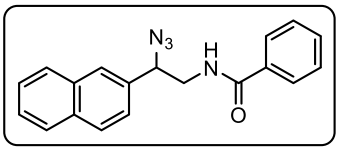

Synthesized according to the General Procedure C1; colorless solid (9.5 mg, 30%); **m.p.** 109–111 °C; **^{1}H NMR** (500 MHz, $CDCl_3$) δ 7.92 (d, *J* = 8.5 Hz, 1H), 7.89 – 7.84 (m, 3H), 7.79 – 7.74 (m, 2H), 7.56 – 7.48 (m, 4H), 7.47 – 7.42 (m, 2H), 6.46 (s, 1H), 5.03 (dd, *J* = 8.9, 4.9 Hz, 1H), 3.99 (ddd, *J* = 13.9, 7.0, 4.9 Hz, 1H), 3.57 (ddd, *J* = 14.0, 9.0, 5.1 Hz, 1H); **^{13}C NMR** (126 MHz, $CDCl_3$) δ 167.8, 134.5, 134.2, 133.5, 133.3, 131.9, 129.3, 128.8, 128.2, 127.9, 127.1, 126.8(2), 126.7(6), 126.6, 124.3, 65.6, 45.4; **IR** (cm^{-1}) 3301, 2090, 1990, 1637, 1532, 1233, 1016, 691; **HRMS** (FAB) m/z calcd. for $C_{19}H_{17}N_4O$ $[M+H]^+$: 317.1402, found: 317.1404.

N-{2-Azido-2-(2-oxopyrrolidin-1-yl)ethyl}benzamide (5g)

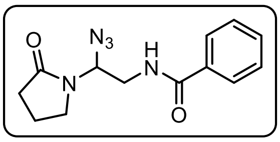

Synthesized according to the General Procedure C1 in DCM (0.2 M); yellow solid (12.2 mg, 45%); **^{1}H NMR** (500 MHz, $CDCl_3$) δ 7.78 – 7.73 (m, 2H), 7.54 (t, *J* = 7.4 Hz, 1H), 7.46 (t, *J* = 7.6 Hz, 2H), 6.39 (s, 1H), 5.86 (dd, *J* = 8.1, 5.8 Hz, 1H), 4.00 (dt, *J* = 14.0, 8.0 Hz, 1H), 3.67 – 3.57 (m, 1H), 3.57 – 3.43 (m, 2H), 2.54 – 2.35 (m, 2H), 2.18 – 2.01 (m, 2H); **^{13}C NMR** (126 MHz, CD_2Cl_2) δ 176.8, 167.7, 134.5, 132.1, 129.0, 127.3, 67.9, 42.9, 41.0, 31.3, 18.7; **IR** (cm^{-1}) 3314, 2922, 2105, 1641, 1532, 1259, 1021, 797, 693; **HRMS** (ESI) m/z calcd. for $C_{13}H_{15}N_5O_2Na$ $[M+Na]^+$: 296.1123, found: 296.1118.

N-{2-(2-Oxopyrrolidin-1-yl)-2-(1*H*-pyrazol-1-yl)ethyl}benzamide (5h)

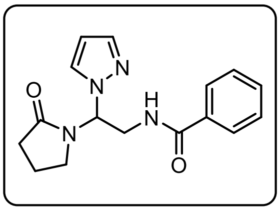

Synthesized according to the General Procedure C2; yellow solid (27.7 mg, 93%); **m.p.** 178–180 °C; **^{1}H NMR** (400 MHz, $CDCl_3$) δ 7.72 – 7.67 (m, 2H), 7.62 – 7.57 (m, 2H), 7.54 – 7.46 (m, 1H), 7.46 – 7.37 (m, 2H), 6.80 (s, 1H), 6.46 (t, *J* = 6.7 Hz, 1H), 6.31 (t, *J* = 2.1 Hz, 1H), 4.45 (ddd, *J* = 13.7, 7.2, 6.4 Hz, 1H), 4.14 (ddd, *J* = 13.9, 7.1, 5.1 Hz, 1H), 3.63 (ddd, *J* = 9.5, 8.3, 5.7 Hz, 1H), 3.24 (ddd, *J* = 9.5, 8.4, 5.9 Hz, 1H), 2.47 – 2.26 (m, 2H), 2.08 – 1.87 (m, 2H); **^{13}C NMR** (101 MHz, $CDCl_3$) δ 176.0, 167.6, 140.3, 134.1, 131.9, 130.6, 128.8, 127.1, 106.5, 65.1, 43.6, 40.2, 31.0, 18.3; **IR** (cm^{-1}) 3359, 2921, 2851, 1679, 1650, 1536, 1260, 1095, 1022, 798, 718, 612; **HRMS** (EI) m/z calcd. for $C_{16}H_{18}N_4O_2$ $[M]^+$: 298.1430, found: 298.1427.

N-{2-(4-Iodo-1*H*-pyrazol-1-yl)-2-(2-oxopyrrolidin-1-yl)ethyl}benzamide (5i)

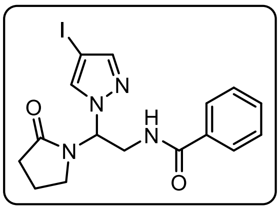

Synthesized according to the General Procedure C2; Colorless solid (29.5 mg, 70%); **m.p.** 190–192 °C; **^{1}H NMR** (500 MHz, $CDCl_3$) δ 7.73 – 7.70 (m, 2H), 7.68 (s, 1H), 7.60 (s, 1H), 7.53 (tt, *J* = 6.8, 1.2 Hz, 1H), 7.45 (t, *J* = 7.6 Hz, 2H), 6.74 – 6.67 (m, 1H), 6.46 (t, *J* = 6.8 Hz, 1H), 4.47 (dt, *J* = 13.9, 6.9 Hz, 1H), 4.12 (ddd, *J* = 13.9, 7.3, 5.1 Hz, 1H), 3.64 (td, *J* = 8.6, 5.7 Hz, 1H), 3.28 (td, *J* = 8.8, 5.9 Hz, 1H),

2.48 – 2.32 (m, 2H), 2.10 – 1.92 (m, 2H); **^{13}C NMR** (126 MHz, $CDCl_3$) δ 176.1, 167.7, 145.3, 134.8, 134.0, 131.9, 128.8, 127.1, 65.5, 57.7, 43.5, 39.8, 30.9, 18.2; **IR** (cm^{-1}) 3312, 2924, 2853, 1682, 1628, 1260, 1020, 794, 697, 667, 607; **HRMS** (EI) m/z calcd. for $C_{16}H_{17}N_4O_2I$ [M]$^+$: 424.0396, found: 424.0399.

***N*-{2-(3,5-Dimethyl-1*H*-pyrazol-1-yl)-2-(2-oxopyrrolidin-1-yl)ethyl}benzamide (5j)**

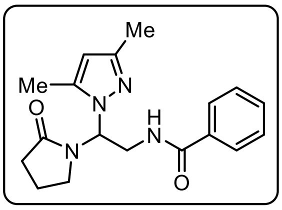

Synthesized according to the General Procedure C2; colorless solid (17.3 mg, 53%); **m.p.** 206–208 °C; **^{1}H NMR** (500 MHz, Acetone-*d*$_6$) δ 7.89 (s, 1H), 7.83 – 7.78 (m, 2H), 7.54 – 7.48 (m, 1H), 7.46 – 7.41 (m, 2H), 6.45 (dd, *J* = 8.6, 5.6 Hz, 1H), 5.80 (s, 1H), 4.39 (ddd, *J* = 13.7, 8.6, 7.1 Hz, 1H), 3.81 (dt, *J* = 13.7, 5.4 Hz, 1H), 3.75 (ddd, *J* = 9.4, 8.2, 5.4 Hz, 1H), 3.32 (ddd, *J* = 9.4, 8.2, 6.2 Hz, 1H), 2.25 (s, 3H), 2.22 – 2.17 (m, 2H), 2.14 (s, 3H), 1.97 – 1.84 (m, 2H); **^{13}C NMR** (126 MHz, Acetone-*d*$_6$) δ 175.5, 167.9, 148.3, 140.6, 135.8, 132.1, 129.2, 128.0, 105.8, 61.7, 43.4, 40.9, 31.3, 18.9, 13.8, 10.7; **IR** (cm^{-1}) 3282, 3065, 2941, 1675, 1546, 1419, 1469, 1023, 788, 687; **HRMS** (ESI) m/z calcd. for $C_{18}H_{22}N_4O_2Na$ [M+Na]$^+$: 349.1640, found: 349.1648.

***N*-Phenethylbenzamide (6a)[14]**

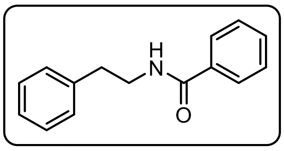

Synthesized according to the General Procedure D in DCM/MeOH (9:1, 0.2 M); colorless solid (12.6 mg, 56%); **m.p.** 115–117 °C; **^{1}H NMR** (500 MHz, $CDCl_3$) δ 7.71 – 7.66 (m, 2H), 7.52 – 7.45 (m, 1H), 7.44 – 7.37 (m, 2H), 7.38 – 7.30 (m, 2H), 7.29 – 7.22 (m, 3H), 6.14 (s, 1H), 3.72 (q, 2H), 2.94 (t, *J* = 6.9 Hz, 2H); **^{13}C NMR** (126 MHz, $CDCl_3$) δ 167.6, 139.0, 134.8, 131.6, 129.0, 128.9, 128.7, 126.9, 126.8, 41.3, 35.8; **IR** (cm^{-1}) 3340, 2923, 2851, 1637, 1541, 1454, 1295, 693, 668, 500; **HRMS** (EI) m/z calcd. for $C_{15}H_{15}NO$ [M]$^+$: 225.1154, found: 225.1157.

***N*-(4-Methylphenethyl)benzamide (6b)[15]**

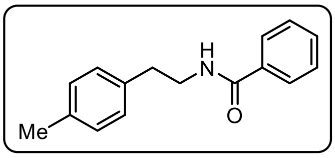

Synthesized according to the General Procedure D in DCM/MeOH (9:1, 0.2 M); Colorless solid (12.4 mg, 52%); **^{1}H NMR** (500 MHz, $CDCl_3$) δ 7.72 – 7.66 (m, 2H), 7.52 – 7.44 (m, 1H), 7.44 – 7.37 (m, 2H), 7.17 – 7.11 (m, 4H), 6.15 (s, 1H), 3.70 (q, *J* = 6.9 Hz, 2H), 2.90 (t, *J* = 6.9 Hz, 2H), 2.34 (s, 3H); **^{13}C NMR** (126 MHz, $CDCl_3$) δ 167.6, 136.3, 135.9, 134.8, 131.5, 129.5, 128.8, 128.7, 126.9, 41.3, 35.4, 21.2; **IR** (cm^{-1}) 3299, 3306, 2938, 1630, 1529, 1485, 1306, 693, 659, 453; **HRMS** (EI) m/z calcd. for $C_{16}H_{17}NO$ [M]$^+$: 239.1310, found: 239.1308.

***N*-{4-(*tert*-Butyl)phenethyl}benzamide (6c)**[15]

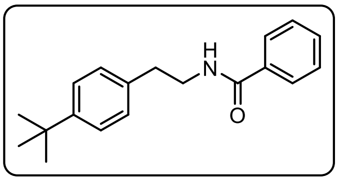

Synthesized according to the General Procedure D in DCM/MeOH (9:1, 0.2 M); Colorless solid (20.1 mg, 71%); **m.p.** 136–138 °C; **^{1}H NMR** (500 MHz, $CDCl_3$) δ 7.71 – 7.67 (m, 2H), 7.51 – 7.46 (m, 1H), 7.44 – 7.39 (m, 2H), 7.37 – 7.34 (m, 2H), 7.21 – 7.16 (m, 2H), 6.14 (s, 1H), 3.72 (q, *J* = 6.7 Hz, 2H), 2.91 (t, *J* = 6.9 Hz, 2H), 1.32 (s, 9H); **^{13}C NMR** (101 MHz, $CDCl_3$) δ 167.6, 149.6, 135.9, 134.9, 131.5, 128.7, 128.6, 127.0, 125.8, 41.3, 35.3, 34.6, 31.5; **IR** (cm^{-1}) 3314, 2958, 1635, 1546, 1491, 1313, 693, 572; **HRMS** (EI) m/z calcd. for $C_{19}H_{23}NO$ $[M]^+$: 281.1780, found: 281.1780.

***N*-(4-Chlorophenethyl)benzamide (6d)**[15]

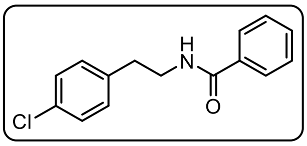

Synthesized according to the General Procedure D in DCM/MeOH (9:1, 0.2 M); colorless solid (13.5 mg, 52%); **m.p.** 134–136 °C; **^{1}H NMR** (500 MHz, $CDCl_3$) δ 7.72 – 7.66 (m, 2H), 7.53 – 7.46 (m, 1H), 7.45 – 7.39 (m, 2H), 7.32 – 7.26 (m, 2H), 7.21 – 7.14 (m, 2H), 6.11 (s, 1H), 3.70 (q, *J* = 6.9 Hz, 2H), 2.92 (t, *J* = 6.9 Hz, 2H); **^{13}C NMR** (101 MHz, $CDCl_3$) δ 167.7, 137.5, 134.6, 132.6, 131.7, 130.3, 129.0, 128.8, 126.9, 41.2, 35.2; **IR** (cm^{-1}) 3348, 2921, 1639, 1535, 1487, 1090, 1013, 692, 508; **HRMS** (EI) m/z calcd. for $C_{15}H_{14}ClNO$ $[M]^+$: 259.0764, found: 259.0766.

***N*-(4-Methoxyphenethyl)benzamide (6e)**[15]

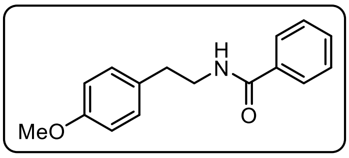

Synthesized according to the General Procedure D in DCM/^{t}BuOH (9:1, 0.2 M); colorless solid (15.6 mg, 61%); **m.p.** 117–119 °C; **^{1}H NMR** (600 MHz, $CDCl_3$) δ 7.71 – 7.66 (m, 2H), 7.48 (t, *J* = 7.4 Hz, 1H), 7.40 (t, *J* = 7.6 Hz, 2H), 7.18 – 7.13 (m, 2H), 6.89 – 6.85 (m, 2H), 6.13 (s, 1H), 3.80 (s, 3H), 3.69 (q, *J* = 6.7 Hz, 2H), 2.88 (t, *J* = 6.9 Hz, 2H); **^{13}C NMR** (101 MHz, $CDCl_3$) δ 167.6, 158.5, 134.8, 131.5, 131.0, 129.9, 128.7, 126.9, 114.3, 55.4, 41.4, 34.9; **IR** (cm^{-1}) 3318, 2922, 1634, 1536, 1510, 1240, 1032, 799, 692, 666; **HRMS** (EI) m/z calcd. for $C_{16}H_{17}NO_2$ $[M]^+$: 255.1259, found: 255.1256.

***N*-{2-(Benzo[*d*][1,3]dioxol-5-yl)ethyl}benzamide (6f)**[16]

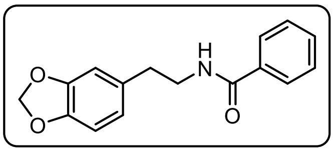

Synthesized according to the General Procedure D in DCM/MeOH (9:1, 0.2 M); colorless solid (11.8 mg, 44%); **m.p.** 104–106 °C; **^{1}H NMR** (500 MHz, $CDCl_3$) δ 7.72 – 7.67 (m, 2H), 7.52 – 7.45 (m, 1H), 7.45 – 7.38 (m, 2H), 6.78 – 6.75 (m, 1H), 6.75 – 6.71 (m, 1H), 6.71 – 6.65 (m, 1H), 6.14 (s, 1H), 5.94 (s, 2H), 3.67 (q, *J* = 6.8 Hz, 2H), 2.85 (*t*, J = 6.9 Hz, 2H); **^{13}C NMR** (126 MHz, $CDCl_3$, *one carbon merged to others*) δ 167.6, 146.4, 134.7, 132.7, 131.6, 128.7, 126.9, 121.8, 109.2, 108.6, 101.1, 41.4, 35.6; **IR** (cm^{-1}) 3378,

2918, 1638, 1536, 1484, 1243, 1036, 706, 688; **HRMS** (EI) m/z calcd. for $C_{16}H_{15}NO_3$ $[M]^+$: 269.1052, found: 269.1053.

N-(2-Methylphenethyl)benzamide (6g)[15]

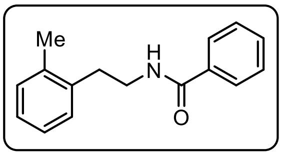

Synthesized according to the General Procedure D in DCM/MeOH (9:1, 0.2 M); colorless solid (11.5 mg, 48%); **m.p.** 91–93 °C; **^{1}H NMR** (500 MHz, $CDCl_3$) δ 7.77 – 7.71 (m, 2H), 7.55 – 7.49 (m, 1H), 7.48 – 7.41 (m, 2H), 7.24 – 7.14 (m, 4H), 6.17 (s, 1H), 3.72 (td, *J* = 7.1, 5.9 Hz, 2H), 2.99 (t, *J* = 7.1 Hz, 2H), 2.40 (s, 3H); **^{13}C NMR** (101 MHz, $CDCl_3$) δ 167.7, 137.1, 136.6, 134.7, 131.6, 130.7, 129.5, 128.7, 126.9(4), 126.8(8), 126.3, 40.1, 33.2, 19.5; **IR** (cm^{-1}) 3321, 2918, 1638, 1539, 1514, 1312, 1293, 691, 667, 489; **HRMS** (EI) m/z calcd. for $C_{16}H_{17}NO$ $[M]^+$: 239.1310, found: 239.1312.

N-{1-(4-Methoxyphenyl)propan-2-yl}benzamide (6h)

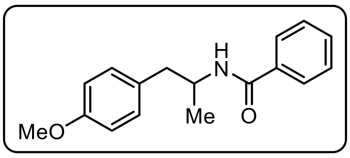

Synthesized according to the General Procedure D in CH_2Cl_2/^{t}BuOH (9:1, 0.2 M); colorless solid (14.5 mg, 54%); **m.p.** 87–89 °C; **^{1}H NMR** (500 MHz, $CDCl_3$) δ 7.72 – 7.67 (m, 2H), 7.52 – 7.45 (m, 1H), 7.45 – 7.38 (m, 2H), 7.17 – 7.11 (m, 2H), 6.88 – 6.82 (m, 2H), 5.89 (d, *J* = 7.5 Hz, 1H), 4.49 – 4.38 (m, 1H), 3.79 (s, 3H), 2.88 (dd, *J* = 13.7, 5.5 Hz, 1H), 2.81 (dd, *J* = 13.7, 6.9 Hz, 1H), 1.21 (d, *J* = 6.7 Hz, 3H); **^{13}C NMR** (126 MHz, $CDCl_3$) δ 166.9, 158.5, 135.0, 131.5, 130.7, 129.9, 128.7, 126.9, 114.0, 55.4, 46.7, 41.6, 20.1; **IR** (cm^{-1}) 2960, 2918, 2850, 1510, 1246, 1025, 798, 691; **HRMS** (EI) m/z calcd. for $C_{17}H_{19}NO_2$ $[M]^+$: 269.1416, found: 269.1413.

N-{2-(Ethylthio)ethyl}benzamide (6i)

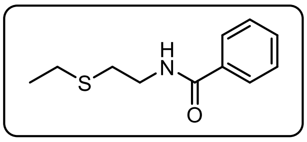

Synthesized according to the General Procedure D in DCM/MeOH (9:1, 0.2 M); colorless liquid (12.1 mg, 58%); **^{1}H NMR** (400 MHz, $CDCl_3$) δ 7.82 – 7.75 (m, 2H), 7.55 – 7.46 (m, 1H), 7.49 – 7.40 (m, 2H), 6.61 (s, 1H), 3.66 (q, *J* = 5.9 Hz, 2H), 2.80 (t, *J* = 6.4 Hz, 2H), 2.59 (q, *J* = 7.4 Hz, 2H), 1.29 (t, *J* = 7.4 Hz, 3H); **^{13}C NMR** (101 MHz, $CDCl_3$) δ 167.6, 134.6, 131.7, 128.8, 127.1, 38.7, 31.6, 25.7, 14.9; **IR** (cm^{-1}) 3306, 2926, 1636, 1532, 1488, 1305, 1291, 693; **HRMS** (EI) m/z calcd. for $C_{11}H_{15}NOS$ $[M]^+$: 209.0874, found: 209.0876.

N-{2-(Phenylthio)ethyl}benzamide (6j)[17]

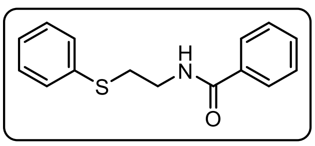

Synthesized according to the General Procedure D in DCM/MeOH (9:1, 0.2 M); colorless solid (19.0 mg, 74%); **m.p.** 84–86 °C; **^{1}H NMR** (500 MHz, $CDCl_3$) δ 7.72 – 7.67 (m, 2H), 7.53 – 7.46 (m, 1H), 7.45 – 7.38 (m, 4H), 7.34 – 7.26 (m, 2H), 7.24 – 7.18 (m, 1H), 6.52 (s, 1H), 3.69 (q, *J* = 6.0 Hz, 2H), 3.19 (t, *J* = 6.3

Hz, 2H); **^{13}C NMR** (101 MHz, $CDCl_3$) δ 167.6, 135.0, 134.4, 131.7, 130.1, 129.4, 128.7, 127.0, 126.8, 39.3, 33.9; **IR** (cm^{-1}) 3363, 3313, 2925, 1639, 1537, 1478, 1023, 686, 672; **HRMS** (EI) m/z calcd. for $C_{15}H_{15}NOS$ $[M]^+$: 257.0874, found: 257.0872.

***N*-{2,2-Bis(phenylsulfonyl)ethyl}benzamide (6k)**

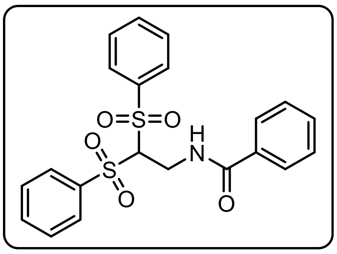

Synthesized according to the General Procedure D in DCM/MeOH (9:1, 0.2 M); colorless solid (38.7mg, 90%); **m.p.** 192–194 °C; **^{1}H NMR** (500 MHz, $CDCl_3$) δ 8.00 – 7.94 (m, 4H), 7.76 – 7.68 (m, 4H), 7.59 (t, *J* = 7.9 Hz, 4H), 7.53 (t, *J* = 7.4 Hz, 1H), 7.44 (t, *J* = 7.8 Hz, 2H), 7.11 (t, *J* = 6.1 Hz, 1H), 4.75 (t, *J* = 5.6 Hz, 1H), 4.25 (t, *J* = 5.9 Hz, 2H); **^{13}C NMR** (126 MHz, $CDCl_3$) δ 167.3, 137.9, 135.2, 133.4, 132.2, 129.5(7), 129.5(5), 128.8, 127.2, 81.5, 36.6; **IR** (cm^{-1}) 3279, 3086, 2034, 1633, 1556, 1307, 1145, 1076, 731, 685; **HRMS** (ESI) m/z calcd. for $C_{21}H_{20}NO_5S_2$ $[M+H]^+$: 430.0783, found: 430.0793.

5. Procedure for the gram-scale synthesis of 3a

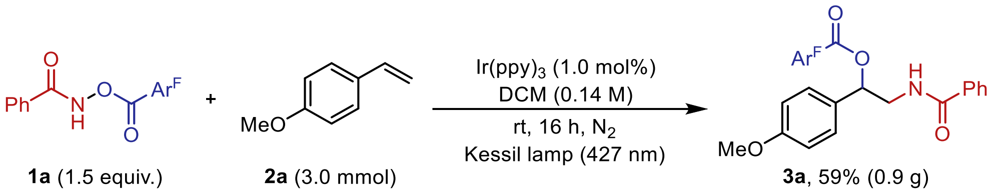

To an oven-dried 40 mL vial with a stir bar were added Ir(ppy)$_3$ (19.6 mg, 1.0 mol%), *N*-[{3,5-bis(trifluoromethyl)benzoyl}oxy]benzamide (**1a**, 1.7 g, 1.5 equiv.), 4-methoxystyrene (**2a**, 3.0 mmol) and anhydrous dichloromethane (21 mL, 0.14 M) under N_2 atmosphere. The vial was stirred at room temperature for 16 h under irradiation with a Kessil lamp (427 nm, maximum intensity), which was positioned 3~5 cm away from the reaction vial. Regular fans were equipped to maintain the temperature. The reaction mixture was poured into saturated $NaHCO_3$ aqueous solution (50 mL) and extracted with DCM (50 mL x 3). The combined organic layer was dried over anhydrous $MgSO_4$, filtered and evaporated under reduced pressure. Desired product **3a** was obtained in 59% yield (0.91 g) by silica gel column chromatography (*n*-hexane/EtOAc, 9:1 ~ 4:1).

6. Unsuccessful or low-yielding examples for 1,2-Amidooxygenation of alkenes using bifunctional *N*-benzoyloxyamides

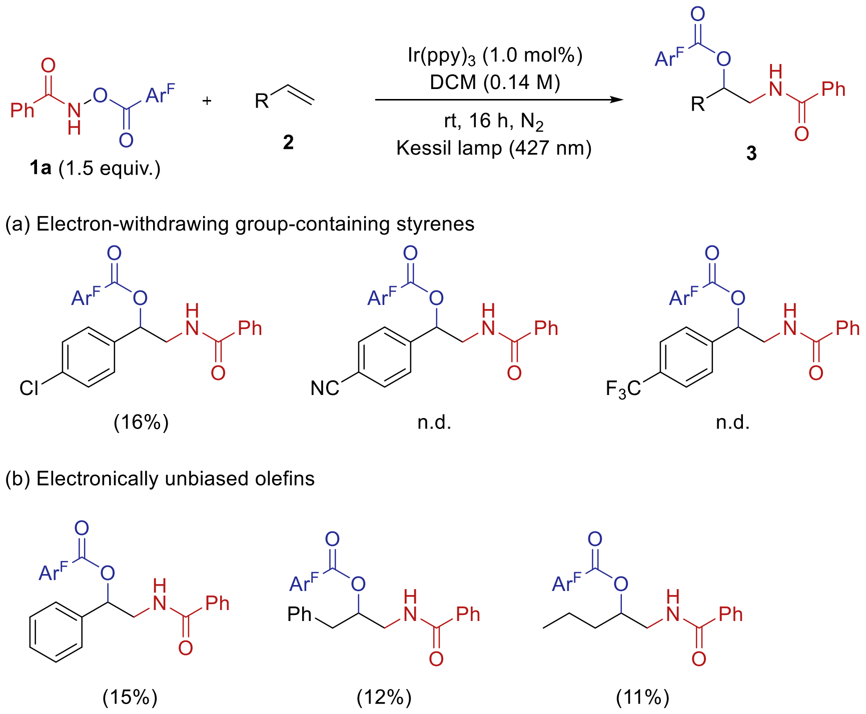

V. Procedures for the Experimental Mechanistic Studies

1. Cyclic Voltammetry (CV) analysis (Figure 1-a)

All electrochemical data were obtained on a Bio-Logic Science VSP potentiostat. Glassy carbon electrode, Pt wire counter electrode, and Ag pseudoreference electrode were used for the measurement to acquire the data in a scan rate of 200 mV/s. A sample was prepared by dissolving the bifunctional N–O reagent **1a** (0.01 M) in a solution of Bu_4NPF_6 (0.1 M) in MeCN (10 mL). As shown in Figure S1, **1a** shows an irreversible reduction peak. E_p was determined to be –1.54 V vs SCE. The cyclic voltammetry of **1a** was corrected through the cyclic voltammetry data of ferrocene in MeCN. The potential was measured with half-peak potential.

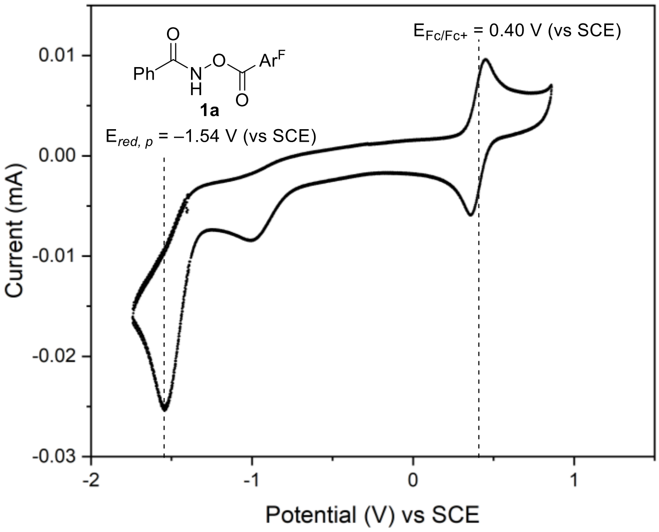

Figure S1. Cyclic Voltammogram of **1a**

2. Control experiment using 3a (Figure 1-c)

2.1. Control experiment with O-nucleophile

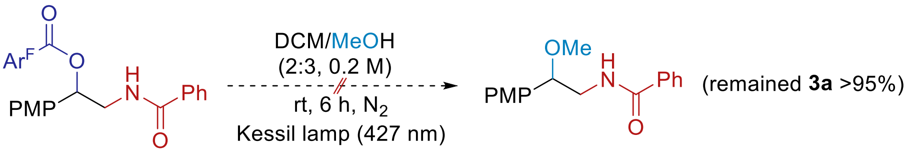

3a (0.04 mmol) **4d** (<5%)

To an oven-dried 1 mL vial with a stir bar were added 1,2-amidooxygenated product **3a** (0.04 mmol) in DCM/MeOH (2:3, 0.2 M) under N_2 atmosphere. The vial was stirred at room temperature for 6 h under irradiation with a Kessil lamp (427 nm, maximum intensity), which was positioned 3~5 cm away from the reaction vial. Regular fans were equipped to maintain the temperature. The solvent was removed under reduced pressure, and the result was analyzed by using ^{1}H NMR spectroscopy (dibromomethane as an internal standard in $CDCl_3$). The 1,2-amidooxygenation product **3a** remained predominantly unreacted with MeOH even under photoirradiation conditions, with only a minor amount of alcohol-substituted compound **4d** (<5%) formed.

2.2. Control experiment with N-nucleophiles

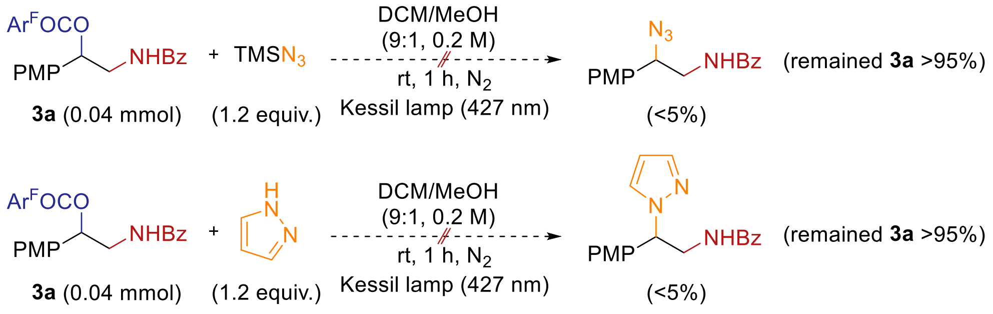

To an oven-dried 1 mL vial with a stir bar were added 1,2-amidooxygenated product **3a** (0.04 mmol), N-nucleophile (1.2 equiv.) in DCM (0.2 M) under N_2 atmosphere. The vial was stirred at room temperature for 1 h under irradiation with a Kessil lamp (427 nm, maximum intensity), which was positioned 3~5 cm away from the reaction vial. Regular fans were equipped to maintain the temperature. The solvent was removed under reduced pressure, and the result was analyzed by using ^{1}H NMR spectroscopy (dibromomethane as an internal standard in $CDCl_3$). The 1,2-amidooxygenation product **3a** remained intact, and no 1,2-diamination products were observed.

3. Radical trap experiment (Figure 1-d)

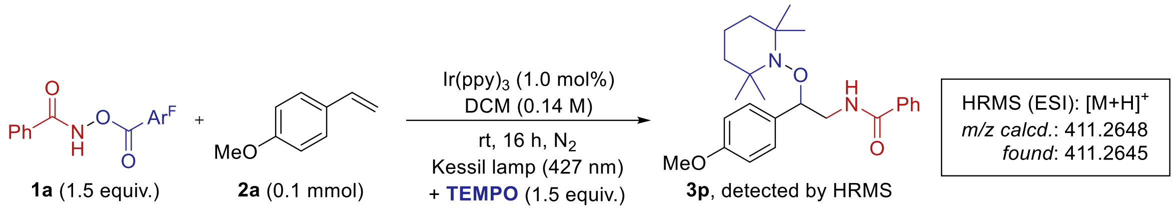

To an oven-dried 4 mL vial with a stir bar were added Ir photocatalyst (1.0 mol%), *N*-[{3,5-Bis(trifluoromethyl)benzoyl}oxy]benzamide (**1a**, 0.15 mmol, 1.5 equiv.), 4-methoxystyrene (**2a**, 0.1 mmol, 1.0 equiv.), TEMPO (2,2,6,6-tetramethylpiperidine 1-oxyl, 0.15 mmol, 1.5 equiv.), in anhydrous DCM (0.7 mL, 0.14 M) under N_2 atmosphere. The vial was stirred at room temperature for 16 h under irradiation with a Kessil lamp (427 nm, maximum intensity), which was positioned 3~5 cm away from the reaction vial. Regular fans were equipped to maintain the temperature. The solvent was removed under reduced pressure, and TEMPO-adduct **3p** was detected by HRMS.

***N*-[2-(4-Methoxyphenyl)-2-{(2,2,6,6-tetramethylpiperidin-1-yl)oxy}ethyl]benzamide (3p)**

HRMS (ESI) m/z calcd. for $C_{25}H_{35}N_2O_3$ $[M+H]^+$: 411.2648, found: 411.2645.

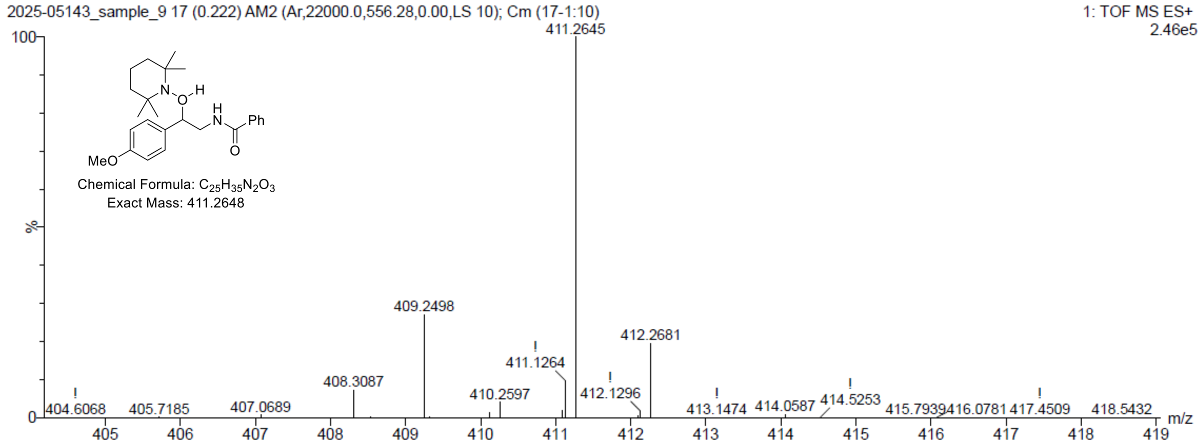

Figure S2. Detection of a TEMPO-adduct **3p** by ESI

4. Stern-Volmer Quenching Experiments

To track the phosphorescence quenching of Ir(ppy)$_3$, photoluminescence was measured on a Shimidazu RF-6000 Spectrofluorophotometer. The first sample was prepared by dissolving photocatalyst Ir(ppy)$_3$ and quencher **1e** in anhydrous DCM/MeOH (9:1). The solution was loaded into quartz cuvettes, and the concentration of Ir(ppy)$_3$ was 50 μM. The sample was excited at 420 nm, and emission intensity was detected at 517 nm. The second experiment was carried out similarly, using a solution of Ir(ppy)$_3$ and TMSN$_3$ in the same anhydrous DCM/MeOH (9:1). The third experiment was also carried out similarly, using a solution of Ir(ppy)$_3$ and 4-methoxystyrene in anhydrous DCM.

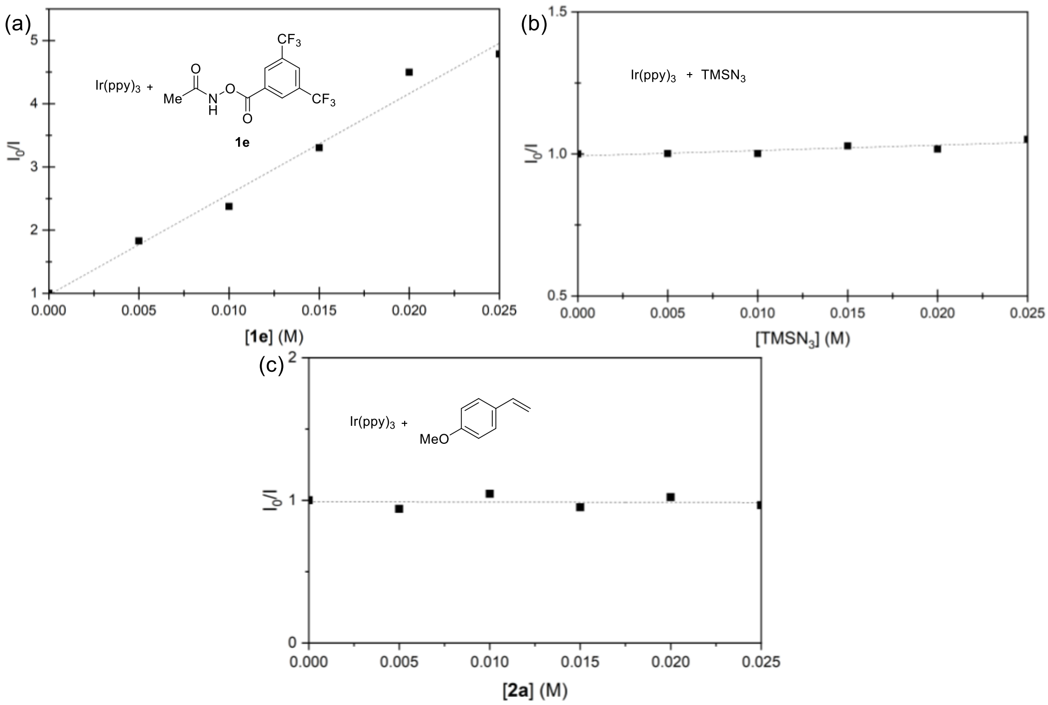

Figure S3. (a) Stern-Volmer plot of Ir(ppy)$_3$ with varied [**1e**]. (b) Stern-Volmer plot of Ir(ppy)$_3$ with varied [TMSN$_3$]. (c) Stern-Volmer plot of Ir(ppy)$_3$ with varied [**2a**].

5. Measurement of Reaction Quantum Yield

5.1. Determination of the photon flux

The photon flux of the LED lamp was determined by using standard ferrioxalate actinometry[18] following a modified literature procedure.[19,20]

A 0.018 M ferrioxalate solution was prepared by dissolving 178 mg of potassium ferrioxalate trihydrate and 84μL of sulfuric acid (95%) in 20 mL of water. This solution was stored in an amber vial in the dark. A buffer solution was prepared by dissolving 2.5 g of sodium acetate and 0.5 mL of sulfuric acid (95%) in 50 mL of water. These solutions were stored in dark condition.

Next, 3.5 mL of 0.018M ferrioxalate solution was transferred to an 8 mL vial with a stir bar in the dark. The vial was positioned 5 cm from a single Kessil LED (427 nm, maximum intensity) and irradiated for 15 seconds. After irradiation, 100 μL of the solution was transferred immediately to a foil-covered 10 mL volumetric flask containing 15 mg of 1,10-phenanthroline dissolved in 3.0 mL buffer solution. Then, water was added to make a total volume of 10 mL. The flask was shaked and stored in the dark for 20 min. Then 3.0 mL solution was transferred to a quartz cuvette (l = 1.0 mL), and the absorbance at λ = 510 nm was measured by UV-Vis spectrometer. The absorption of non-irradiated sample was also measured with the same process.

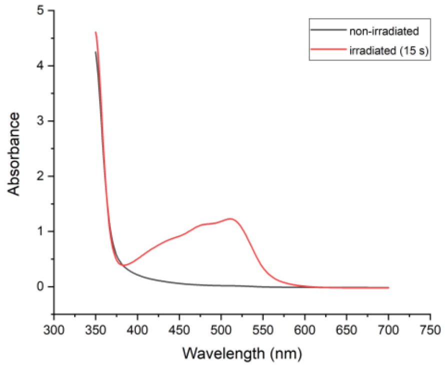

Figure S4. UV-Vis spectra of ferrioxalate/1,10-phenanthroline solutions

The number of moles of Fe(II) formed was calculated using:

$$\text{mol Fe(II)} = \frac{V_1 V_3 \Delta A(510\text{nm})}{V_2 l \varepsilon(510\text{nm})}$$

$$= \frac{(3.5\times10^{-3}\ \text{L})\times(1.0\times10^{-2}\ \text{L})\times1.210}{(1.0\times10^{-4}\ \text{L})\times(1.0\ \text{cm})\times(11100\ \text{L mol}^{-1}\text{cm}^{-1})} = 3.815\times10^{-5}\ \text{mol}$$

Where V_1 is the volume of the irradiated ferrioxalate (3.5 × 10^{-3} L), V_2 is the volume of the aliquot taken for the UV-Vis measurement (1.0 × 10^{-4} L), V_3 is the final volume after complexation with 1,10-phenanthroline (1.0 × 10^{-2} L), ΔA(510 nm) is the difference in absorbance at λ = 510 nm between the

irradiated and non-irradiated sample, l is the optical path length of the cuvette (1.0 cm), and ε (510 nm) is the molar absorptivity of the $Fe(phen)_3^{2+}$ complex at λ = 510 nm (11,100 L mol^{-1} cm^{-1}).

The photon flux was then computed as following:

$$\text{Photon flux} = \frac{\text{mol Fe(II)}}{\phi t f} = \frac{3.815\times10^{-5}\ \text{mol}}{1.11\times(15\ \text{s})\times0.926} = 2.474\times10^{-6}\ \text{einstein s}^{-1}$$

Where ϕ is the quantum yield of the ferrioxalate actinometer (1.11 at λ = 427 nm)[18], t is the time (15 s) and f is the fraction of absorbed light at λ = 427 nm, where f = 1 – 10^{-A}. The absorbance (A) of the 0.018 M ferrioxalate solution at λ = 427 nm was measured in a quartz cell was 1.129, therefore, f = 0.926.

5.2 Determination of the reaction quantum yield

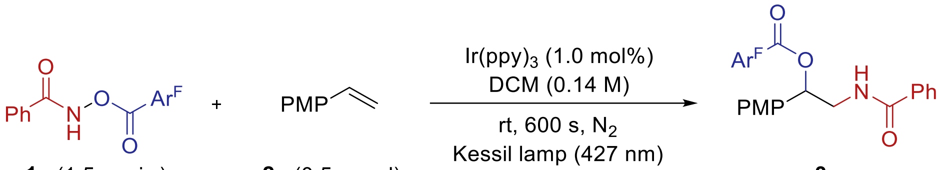

1a (1.5 equiv.) **2a** (0.5 mmol) **3a**

To an 8 mL vial with a stir bar were added substrate $Ir(ppy)_3$ (3.3 mg, 1 mol%), **1a** (1.5 equiv.), **2a** (0.5 mmol), and anhydrous dichloromethane (3.5 mL) under N_2 atmosphere. The vial was stirred at room temperature for 600 s under irradiation with a Kessil lamp (427 nm, maximum intensity) which was positioned 5 cm away from the vial. Regular fans were equipped to maintain the temperature. Upon completion, the solvent was removed under reduced pressure and the crude yield of 1,2-amidooxygenation product **3a** (10%, mole of product = 5.0 × 10^{-5}) was determined by using ^{1}H NMR spectroscopy (dibromomethane as an internal standard in $CDCl_3$).

The reaction quantum yield (Φ) was calculated using below equation:

$$\text{Quantum yield }(\Phi) = \frac{\text{mol product}}{\text{Photon flux}\times \text{t}\times f} = \frac{5.0\times10^{-5}\ \text{mol}}{(2.474\times10^{-6}\ \text{einstein s}^{-1})\times(600\ \text{s})\times 1} = 0.034$$

Where t is the time reaction (600 s) and f is the fraction of the light absorbed by the reaction mixture at λ = 427 nm, calculated as f = 1 – 10^{-A}. The absorbance (A) of reaction mixture at 427 nm was measured to be greater than 3, indicating that more than 0.999 of the incident light was absorbed. Therefore, f can be approximated as 1.

6. Carboradical trapping experiment via HAT (Figure 1-e)

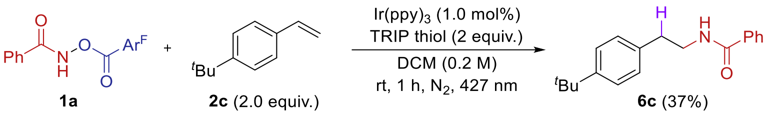

To an oven-dried 4 mL vial with a stir bar were added Ir(ppy)$_3$ (1.0 mol%), *N*-[{3,5-bis(trifluoromethyl)benzoyl}oxy]benzamide (**1a**, 0.1 mmol, 1.0 equiv.), 4-*tert*-butylstyrene (**2c**, 0.2 mmol, 2.0 equiv.), 2,4,6-triisopropylbenzenethiol (TRIP thiol, 2.0 equiv.) in DCM/MeOH (9:1, 0.2 M) under N_2 atmosphere. The vial was stirred at room temperature for 1 h under irradiation with a Kessil lamp (427 nm, maximum intensity), which was positioned 3~5 cm away from the reaction vial. Regular fans were equipped to maintain the temperature. Solvent was removed under reduced pressure and the product yield in crude mixture was determined by using ^{1}H NMR spectroscopy (dibromomethane as an internal standard in $CDCl_3$).

VI. Computational Studies

1. General details

All computations were carried out using density functional theory (DFT)[21] implemented in Gaussian 09 suite of program.[19] Geometry optimizations were performed in the gas phase with M06-2X[22] levels of theory and 6-31G** basis set. After geometry optimizations, the energies were re-evaluated with optimized structures under M06-2X level[22] and 6-311+G** basis set. Solvation energy corrections were carried out at the same level of the single point energy calculations using SMD[23] model (solvent = dichloromethane), where the solution phase electronic energies (E_{Sol}) were evaluated. Vibrational frequency calculations were conducted at the same level as the geometry optimizations, to derive the thermochemistry correction term (G − E) as well as to confirm the stationary points as either minima (no imaginary frequencies) or saddle points (one imaginary frequency) on the potential energy surface. Final solution phase Gibbs free energies (G_{Sol}) were computed as follows:

$$G_{Sol} = E_{Sol} + (G - E) \quad (1)$$

$$\Delta G_{Sol} = \Sigma G_{Sol} \text{ for products} - \Sigma G_{Sol} \text{ for reactants} \quad (2)$$

2. Calculation of redox potential

Standard one-electron redox potentials, E_0, are related to the total electron attachment energy in solution phase, ΔG^{EA}_{Sol}, by

$$\Delta G^{EA}_{Sol} = -nFE_0 \quad (1)$$

where F is the Faraday constant and n is the number of electrons (n = 1 for one-electron process). ΔG^{EA}_{Sol} was calculated as following:

$$\Delta G^{EA}_{Sol} = G_{Sol}[A(-1)] - G_{Sol}[A] \quad (2)$$

To compare E_0 with experimental values, we referenced the normal hydrogen electrode (NHE) calculated by Cramer (4.28 V).[23] So, the relative potential is computed using the formula:

$$E_0(NHE) = -\Delta G^{EA}_{Sol} - 4.28\ V \quad (3)$$

To convert the NHE potential to standard calomel electrode (SCE) potential, we subtracted 0.2412 V from the E_0(NHE).

$$E_0(SCE) = E_0(NHE) - 0.2412\ V \quad (4)$$

3. Energy components of all optimized geometries

Table S8. Computed energies of the optimized geometries

	**E(sol)** (SCF/TZ) [eV] SMD(dichloromethane)M06-2X/6-311+G**	**G-E** [eV] M06-2X/6-31G**	**G(sol)** [eV]
**1a**	-40668.133	4.859	-40663.274
**1a’**	-40670.741	4.734	-40666.008
**1a’-TS**	-40670.270	4.743	-40665.526
**A**	-10890.634	2.217	-10888.417
**B**	-29780.609	1.881	-29778.728
**2a**	-8424.499	2.813	-8421.686
**I**	-19317.033	5.765	-19311.269
**TS-I**	-19315.305	5.661	-19309.645
**II**	-19312.021	5.765	-19306.256
**3a**	-49081.603	8.451	-49073.152
**I’**	-19316.329	5.662	-19310.667
**TS-I’**	-19315.077	5.608	-19309.469
**II’**	-19312.354	5.854	-19306.500
**3a’**	-49095.205	8.569	-49086.636

4. Cartesian coordinates of all optimized geometries

1a

C	3.314285	-7.897675	-1.895865
O	2.508337	-8.735645	-2.222478
N	4.693509	-8.137162	-1.953606
O	5.022739	-9.485658	-1.939940
H	5.270561	-7.623283	-1.296061
C	4.846129	-10.064634	-0.716616
O	4.587681	-9.435595	0.274674
C	5.055145	-11.535163	-0.776196
C	5.030367	-12.233797	0.428630
C	5.214715	-13.611182	0.422645
C	5.423626	-14.291900	-0.771605
C	5.444621	-13.581189	-1.968340
C	5.259456	-12.204451	-1.983401
H	4.869295	-11.691810	1.354754
H	5.567500	-15.367283	-0.770928
H	5.263325	-11.658035	-2.919295
C	5.133159	-14.375877	1.715909
C	5.725557	-14.320034	-3.248740
F	5.606559	-13.654153	2.739018
F	5.833220	-15.515742	1.655167
F	3.868000	-14.700393	2.010403
F	7.041064	-14.513870	-3.415591
F	5.146970	-15.527948	-3.249058
F	5.280792	-13.647430	-4.315278
C	2.959165	-6.501164	-1.503239
C	1.876009	-5.903142	-2.149086
C	3.633528	-5.820106	-0.487717
C	1.493581	-4.612196	-1.807014
C	3.239640	-4.531700	-0.139609
C	2.178170	-3.924887	-0.805499
H	1.348657	-6.466034	-2.912112
H	4.427695	-6.307608	0.071474
H	0.658873	-4.142094	-2.316000
H	3.754296	-4.006794	0.658023
H	1.875432	-2.918455	-0.535419

1a'

C	3.304674	-7.659685	-2.258217
O	2.842365	-8.087452	-3.302288
N	4.264205	-8.263154	-1.516438
O	4.500568	-9.605440	-1.720738
H	4.449568	-8.038771	-0.538840
C	4.777611	-10.227006	-0.475792
O	4.789498	-9.523895	0.548578
C	5.033204	-11.608983	-0.605777
C	5.349828	-12.362089	0.569839
C	5.605768	-13.708053	0.486445
C	5.571212	-14.406694	-0.740368
C	5.256439	-13.659109	-1.894610
C	4.992382	-12.310832	-1.854437
H	5.382676	-11.844486	1.521294
H	5.772603	-15.469634	-0.792441
H	4.749925	-11.773869	-2.763303
C	5.956563	-14.485351	1.711416
C	5.209201	-14.395213	-3.193310
F	5.877260	-13.764752	2.843364
F	7.212431	-14.980243	1.659938
F	5.154310	-15.558451	1.880613
F	6.379086	-15.011072	-3.470559
F	4.279651	-15.374802	-3.189434
F	4.932059	-13.607803	-4.245355
C	2.918695	-6.302006	-1.721752
C	2.467478	-5.358170	-2.644711
C	2.940830	-5.981284	-0.362453
C	2.074120	-4.094284	-2.221064
C	2.539758	-4.717040	0.061605
C	2.113715	-3.769915	-0.865843
H	2.428701	-5.649525	-3.689214
H	3.237038	-6.723961	0.373840
H	1.732308	-3.361245	-2.945502
H	2.552543	-4.475578	1.119834
H	1.803391	-2.784239	-0.532318

1a'-TS

Imaginary Frequency = –925.17 cm^{-1}

C	3.930286	-8.175170	-2.588598
O	3.200369	-9.149088	-2.733404
N	5.247563	-8.174977	-3.009258
O	5.999373	-9.496510	-2.497069
H	5.784714	-7.441736	-2.553768
C	5.648969	-9.766389	-1.231749
O	5.579013	-8.892278	-0.359426
C	5.321066	-11.172481	-1.011222
C	5.285062	-11.669168	0.305812
C	5.038123	-13.015157	0.536869
C	4.803439	-13.902716	-0.511151
C	4.817351	-13.395168	-1.819290
C	5.074770	-12.062573	-2.076950
H	5.443670	-10.975278	1.123796
H	4.602413	-14.950920	-0.321479
H	5.068649	-11.671279	-3.087188
C	5.083994	-13.546592	1.935503
C	4.526361	-14.350344	-2.932683
F	4.250729	-14.590339	2.111274
F	4.755147	-12.621862	2.851326
F	6.312605	-13.991470	2.268676
F	5.379160	-15.397507	-2.927625
F	3.293763	-14.886031	-2.830152
F	4.609059	-13.786911	-4.145150
C	3.428988	-6.886997	-2.017414
C	2.134663	-6.476519	-2.354821
C	4.190556	-6.099426	-1.144141
C	1.628492	-5.275351	-1.872227
C	3.675541	-4.905182	-0.653860
C	2.399420	-4.480105	-1.025365
H	1.544162	-7.122829	-2.996526
H	5.151612	-6.474847	-0.802243
H	0.626886	-4.959422	-2.150479
H	4.265090	-4.309532	0.037232
H	2.002841	-3.543934	-0.643520

A

C	3.498929	-7.737244	-1.862871
O	2.918922	-8.435411	-2.687051
N	4.690105	-8.167709	-1.277189
H	4.720420	-9.182614	-1.430568
C	3.034479	-6.375761	-1.486855
C	1.828864	-5.913636	-2.021349
C	3.771167	-5.572963	-0.612403
C	1.361750	-4.650860	-1.682569
C	3.298366	-4.308804	-0.275443
C	2.096757	-3.848828	-0.809140
H	1.278656	-6.559077	-2.697961
H	4.708118	-5.943229	-0.211404
H	0.426366	-4.288906	-2.096132
H	3.868243	-3.681034	0.401354
H	1.730596	-2.861868	-0.544628

B

O	4.477145	-9.615573	-1.647724
C	4.762983	-10.130361	-0.549809
O	4.859625	-9.644228	0.593165
C	5.051916	-11.658798	-0.618789
C	5.390721	-12.367245	0.530308

C	5.651342	-13.735363	0.465466
C	5.577215	-14.419208	-0.744191
C	5.236918	-13.706551	-1.891442
C	4.976620	-12.339082	-1.831943
H	5.438245	-11.807770	1.460615
H	5.781955	-15.482089	-0.792815
H	4.714325	-11.756079	-2.710309
C	6.053138	-14.458892	1.712351
C	5.096953	-14.426767	-3.195811
F	5.242319	-14.176720	2.746053
F	7.297033	-14.133475	2.111626
F	6.040171	-15.797882	1.559413
F	5.740018	-15.611611	-3.199573
F	3.811348	-14.695309	-3.495560
F	5.582285	-13.714772	-4.225669

2a

C	2.018453	-7.427209	-0.146977
C	0.718442	-7.074537	-0.525907
C	2.649087	-6.670356	0.849391
C	0.070944	-5.989012	0.055483
C	2.004784	-5.586568	1.431467
C	0.713672	-5.239049	1.035457
H	0.214618	-7.658733	-1.291441
H	3.645903	-6.942333	1.182150
H	-0.936009	-5.730614	-0.256162
H	2.507976	-5.013273	2.203605
H	0.211463	-4.393505	1.494005
C	2.669489	-8.575657	-0.803238
C	3.963889	-8.889977	-0.748853
H	2.005295	-9.202751	-1.396181
H	4.684576	-8.292301	-0.199428
H	4.348303	-9.760339	-1.268126

I

C	2.965763	-8.091505	-2.114932
O	2.030511	-8.725827	-2.574983
N	4.205627	-8.637478	-1.936898
H	4.990347	-8.019564	-1.794900
C	2.818805	-6.654528	-1.702661
C	1.762415	-5.935945	-2.263934
C	3.665989	-6.034302	-0.781974
C	1.571340	-4.600566	-1.929875
C	3.469374	-4.698994	-0.442748
C	2.427021	-3.979660	-1.021552
H	1.104129	-6.449655	-2.956378
H	4.458182	-6.598031	-0.297724
H	0.753595	-4.042818	-2.374346
H	4.124029	-4.223208	0.279992
H	2.276077	-2.937803	-0.757843
C	4.488891	-9.998862	-2.359545
C	5.507764	-10.626031	-1.468743
C	6.458033	-11.602531	-1.859028
C	7.368598	-12.112718	-0.897471
C	6.552230	-12.111628	-3.179465
C	8.315592	-13.061542	-1.236479
C	7.504146	-13.062809	-3.508292
C	8.393255	-13.544342	-2.545734
H	7.310848	-11.741076	0.121902
H	5.865936	-11.762839	-3.943729
H	9.000146	-13.432371	-0.480230
H	7.554553	-13.437998	-4.525587
H	9.135624	-14.289235	-2.811191
H	3.529425	-10.530971	-2.322723
H	4.810233	-10.016136	-3.409126
H	5.470628	-10.336798	-0.421809

TS-I

Imaginary Frequency = −266.68 cm^{-1}

C	2.679749	-8.826349	-1.806965
O	2.411436	-9.299928	-2.903330
N	2.157557	-9.405522	-0.657042
H	2.711147	-9.110602	0.151492
C	3.600231	-7.640754	-1.696227
C	4.328825	-7.286156	-2.833981
C	3.777166	-6.916877	-0.513916
C	5.249135	-6.246849	-2.780694
C	4.687631	-5.866035	-0.465425
C	5.431937	-5.536816	-1.595539
H	4.169763	-7.857134	-3.741847
H	3.191176	-7.148804	0.370873
H	5.827002	-5.991061	-3.663096
H	4.814841	-5.303652	0.453782
H	6.149251	-4.723297	-1.553548
C	3.096694	-11.532911	-0.825568
C	4.370970	-11.143311	-0.587884
C	5.299828	-10.594626	-1.577154
C	6.416108	-9.870380	-1.132064
C	5.098501	-10.735873	-2.959254
C	7.296683	-9.289093	-2.034931
C	5.986605	-10.163849	-3.861373
C	7.084184	-9.435885	-3.404233
H	6.578153	-9.752940	-0.063786
H	4.243958	-11.293894	-3.326175
H	8.146639	-8.720401	-1.672377
H	5.819590	-10.284232	-4.926923
H	7.772925	-8.987166	-4.113001
H	2.488417	-11.945844	-0.029632
H	2.666961	-11.541148	-1.821219
H	4.737890	-11.165562	0.437573

II

C	3.239242	-8.050186	-1.975786
O	2.407379	-8.914925	-2.180254
N	4.595488	-8.361191	-2.080332
H	5.246777	-7.604564	-2.234065
C	2.903583	-6.651166	-1.608093
C	1.601031	-6.219843	-1.870734
C	3.824464	-5.784601	-1.011176
C	1.229034	-4.918090	-1.559932
C	3.445039	-4.485189	-0.695444
C	2.150919	-4.050980	-0.975964
H	0.900491	-6.916289	-2.318614
H	4.823178	-6.123858	-0.751012
H	0.220655	-4.579082	-1.769837
H	4.154131	-3.814252	-0.223284
H	1.858394	-3.035545	-0.730625
C	4.914766	-9.659078	-2.612026
C	5.677373	-10.486466	-1.668232
C	6.507972	-11.566240	-1.949233
C	7.059606	-12.288865	-0.848349
C	6.790009	-11.993770	-3.282744
C	7.848609	-13.397131	-1.070880
C	7.574469	-13.104102	-3.491092
C	8.100781	-13.799535	-2.388336
H	6.841953	-11.957175	0.162792
H	6.387313	-11.446610	-4.128180
H	8.268744	-13.952862	-0.241067
H	7.793839	-13.442210	-4.496968
H	8.723570	-14.671561	-2.564879
H	3.926806	-10.168421	-2.720322
H	5.353910	-9.623534	-3.614358
H	5.519581	-10.230449	-0.618983

3a

C	4.462049	-8.414537	-4.128553
O	3.630593	-9.309936	-4.180723
N	5.787019	-8.678334	-3.940671
H	6.414101	-7.941088	-3.655534
C	4.082587	-6.970118	-4.266618
C	2.747466	-6.636735	-4.034002
C	4.992550	-5.974992	-4.630230

C	2.330247	-5.315278	-4.137879
C	4.571957	-4.653111	-4.739817
C	3.243092	-4.321710	-4.487439
H	2.057487	-7.433112	-3.776436
H	6.022314	-6.228814	-4.864275
H	1.293151	-5.058354	-3.949020
H	5.279566	-3.883720	-5.030403
H	2.917234	-3.289953	-4.571415
C	6.233298	-10.037495	-3.739176
C	5.752757	-10.650728	-2.413238
C	6.461914	-11.943396	-2.088083
C	5.723580	-13.122454	-1.986887
C	7.849041	-11.987319	-1.908424
C	6.355167	-14.332158	-1.709849
C	8.477752	-13.194317	-1.621863
C	7.733512	-14.368597	-1.524210
H	4.646592	-13.091511	-2.127216
H	8.430848	-11.073808	-1.986013
H	5.769903	-15.242822	-1.635650
H	9.553136	-13.219690	-1.478492
H	8.228254	-15.309118	-1.304557
H	5.814763	-10.667712	-4.529420
H	7.318909	-10.057236	-3.810314
H	4.681329	-10.832364	-2.511443
O	5.792028	-9.734765	-1.285714
C	6.805253	-8.903881	-1.074884
O	7.782996	-8.779537	-1.782970
C	6.605252	-8.092271	0.167224
C	5.470891	-8.231301	0.966876
C	7.599751	-7.179558	0.509301
C	5.343707	-7.449524	2.108536
C	7.454706	-6.402475	1.653010
C	6.329480	-6.531146	2.458979
H	4.697916	-8.940066	0.694025
H	8.477800	-7.092627	-0.122266
H	6.220516	-5.922843	3.350335
C	8.505425	-5.377911	1.982184
F	8.320024	-4.249765	1.282930
F	8.486553	-5.050194	3.280190
F	9.731998	-5.826396	1.687595
C	4.155968	-7.629982	3.013862
F	3.803244	-6.473553	3.591239
F	3.094586	-8.099039	2.347734
F	4.423707	-8.496922	3.999914

==============================

I'

==============================

C	2.332137	-7.147612	-0.474681
C	1.075325	-7.600783	-0.883378
C	2.435886	-6.391336	0.691606
C	-0.058404	-7.310232	-0.134317
C	1.299760	-6.097216	1.443717
C	0.052583	-6.556820	1.033817
H	0.991793	-8.182005	-1.799201
H	3.410954	-6.035790	1.013231
H	-1.028871	-7.671166	-0.459211
H	1.391539	-5.508922	2.351122
H	-0.831453	-6.330035	1.620764
C	3.545099	-7.437954	-1.356826
C	4.843340	-7.432976	-0.617752
H	3.381197	-8.435930	-1.784611
H	5.653199	-6.794920	-0.944360
H	4.987398	-8.092199	0.228924
C	3.312764	-6.781447	-3.799285
O	2.850957	-5.938376	-4.548777
N	3.568347	-6.494637	-2.479085
H	3.277243	-5.550704	-2.249761
C	3.671093	-8.148614	-4.308240
C	2.906504	-8.656255	-5.360194
C	4.771893	-8.870542	-3.840143
C	3.215741	-9.892371	-5.915354
C	5.089453	-10.100934	-4.407994
C	4.307214	-10.616266	-5.438754
H	2.081624	-8.058612	-5.733772
H	5.387962	-8.462568	-3.043081
H	2.612753	-10.288526	-6.725827
H	5.950611	-10.654899	-4.048957
H	4.553663	-11.578270	-5.876639

==============================

TS-I'

Imaginary Frequency = –560.14 cm^{-1}

==============================

C	1.721369	-7.302964	-0.603781
C	0.426359	-7.138904	-0.106998
C	2.777203	-6.615631	0.004144
C	0.192415	-6.328017	1.000544
C	2.543503	-5.806930	1.108712
C	1.250835	-5.664538	1.613024
H	-0.399678	-7.658667	-0.585011
H	3.774425	-6.695050	-0.418492
H	-0.816988	-6.213717	1.381866
H	3.368099	-5.274033	1.570888
H	1.069953	-5.028722	2.473458
C	1.952676	-8.194911	-1.762224
C	3.027647	-9.039724	-1.840757
H	1.074830	-8.439834	-2.358339
H	3.855614	-8.961534	-1.144119
H	3.103970	-9.780247	-2.627597
C	2.426799	-7.211648	-4.408037
O	1.451434	-7.120330	-5.145893
N	2.480103	-6.630578	-3.148796
H	1.562121	-6.196442	-3.002592
C	3.661792	-7.940268	-4.835108
C	3.605889	-8.731156	-5.984224
C	4.853059	-7.845764	-4.110349
C	4.730162	-9.435329	-6.399816
C	5.978055	-8.548642	-4.530407
C	5.916040	-9.345434	-5.672007
H	2.671559	-8.776847	-6.534074
H	4.881682	-7.211081	-3.230653
H	4.685324	-10.053077	-7.290853
H	6.905402	-8.471428	-3.971953
H	6.794341	-9.893817	-5.997903

==============================

II'

==============================

C	1.701289	-7.301749	-0.386621
C	0.755274	-6.354635	-0.784986
C	2.664179	-6.967327	0.572164
C	0.765642	-5.082664	-0.221566
C	2.677549	-5.692374	1.124054
C	1.727062	-4.751229	0.729069
H	0.005951	-6.615053	-1.526959
H	3.399352	-7.693804	0.911558
H	0.023874	-4.352771	-0.525695
H	3.421136	-5.435732	1.870230
H	1.735119	-3.759661	1.168252
C	1.663061	-8.640884	-1.022391
C	2.373540	-9.830675	-0.525043
H	0.826539	-8.844671	-1.686564
H	2.967029	-9.767713	0.379590
H	1.994354	-10.807750	-0.805269
C	2.890719	-9.753832	-3.039792
O	2.167497	-10.699314	-3.137160
N	2.961856	-9.114401	-1.683499
H	3.769144	-8.521424	-1.491372
C	3.717677	-9.113454	-4.063772
C	3.970749	-9.859798	-5.222638
C	4.212978	-7.808288	-3.930708
C	4.742744	-9.307623	-6.234257
C	4.973191	-7.261067	-4.954738
C	5.244165	-8.013084	-6.098250
H	3.564724	-10.862343	-5.306108
H	3.974757	-7.191759	-3.067011
H	4.953089	-9.882525	-7.128800
H	5.349528	-6.248424	-4.866723
H	5.845538	-7.582890	-6.892020

==============================

3a'

==============================

C	1.287299	-6.811132	0.013813
C	-0.073680	-7.052755	0.202635
C	1.755815	-5.491947	0.013233
C	-0.960739	-5.996530	0.391492
C	0.865467	-4.437841	0.195956
C	-0.491875	-4.686193	0.385895
H	-0.442096	-8.075175	0.196443
H	2.817884	-5.290711	-0.105087
H	-2.017243	-6.197985	0.535376
H	1.236782	-3.418196	0.197243
H	-1.181621	-3.860834	0.529342
C	2.238585	-7.971868	-0.170369
C	3.143784	-8.196243	1.049403
H	1.668827	-8.902737	-0.295960
H	2.534422	-8.299539	1.948921
H	3.724320	-9.105679	0.886028
C	3.761304	-8.786969	-1.921736
O	3.823587	-9.887825	-1.393528
N	3.058322	-7.758522	-1.357961
H	2.834588	-6.952647	-1.923272
C	4.474458	-8.475115	-3.204451
C	4.938578	-9.556404	-3.953753
C	4.721822	-7.169497	-3.641169
C	5.610605	-9.337462	-5.151131
C	5.399063	-6.953905	-4.836663
C	5.833150	-8.037333	-5.597716
H	4.765714	-10.557455	-3.573189
H	4.435445	-6.315487	-3.032186
H	5.970618	-10.181140	-5.730333
H	5.597816	-5.940252	-5.168072
H	6.362429	-7.866249	-6.529412
O	5.261457	-5.749101	0.021100
C	5.053013	-6.844320	0.476912
O	3.982793	-7.062073	1.279611
C	5.986710	-7.995074	0.255819
C	6.151868	-9.006353	1.197783
C	7.005828	-10.070065	0.922179
C	7.678856	-10.141946	-0.290514
C	7.525278	-9.111807	-1.214790
C	6.706809	-8.026957	-0.938141
H	5.615797	-8.971156	2.141066
H	8.313489	-10.993234	-0.514886
H	6.596240	-7.218670	-1.651843
C	7.221140	-11.131826	1.964634
C	8.255616	-9.209900	-2.524936
F	6.113659	-11.335736	2.690672
F	8.193328	-10.785115	2.819723
F	7.574077	-12.299471	1.413817
F	9.583364	-9.252186	-2.339426
F	7.922587	-10.332656	-3.179431
F	7.988429	-8.173706	-3.325257

VII. ^{1}H, ^{13}C and ^{19}F NMR Spectra

N-Hydroxythiophene-3-carboxamide

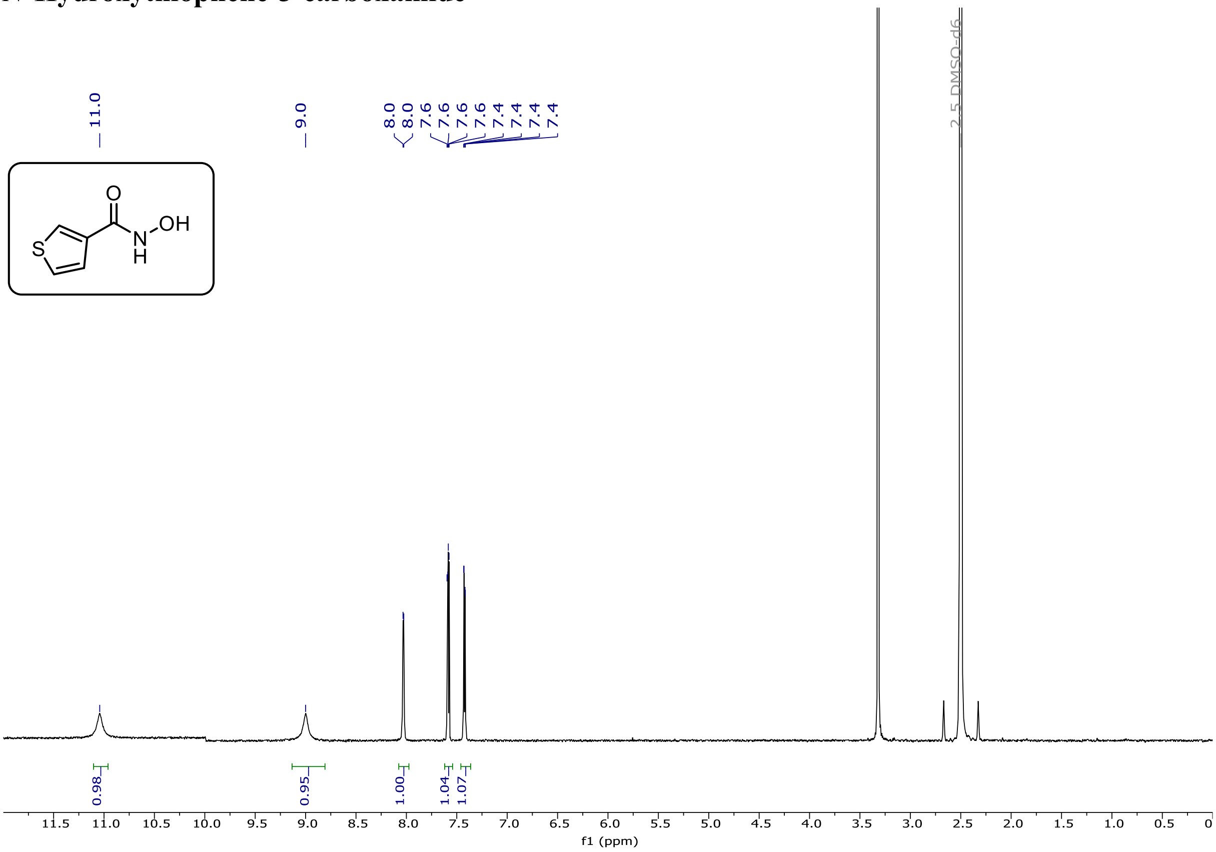

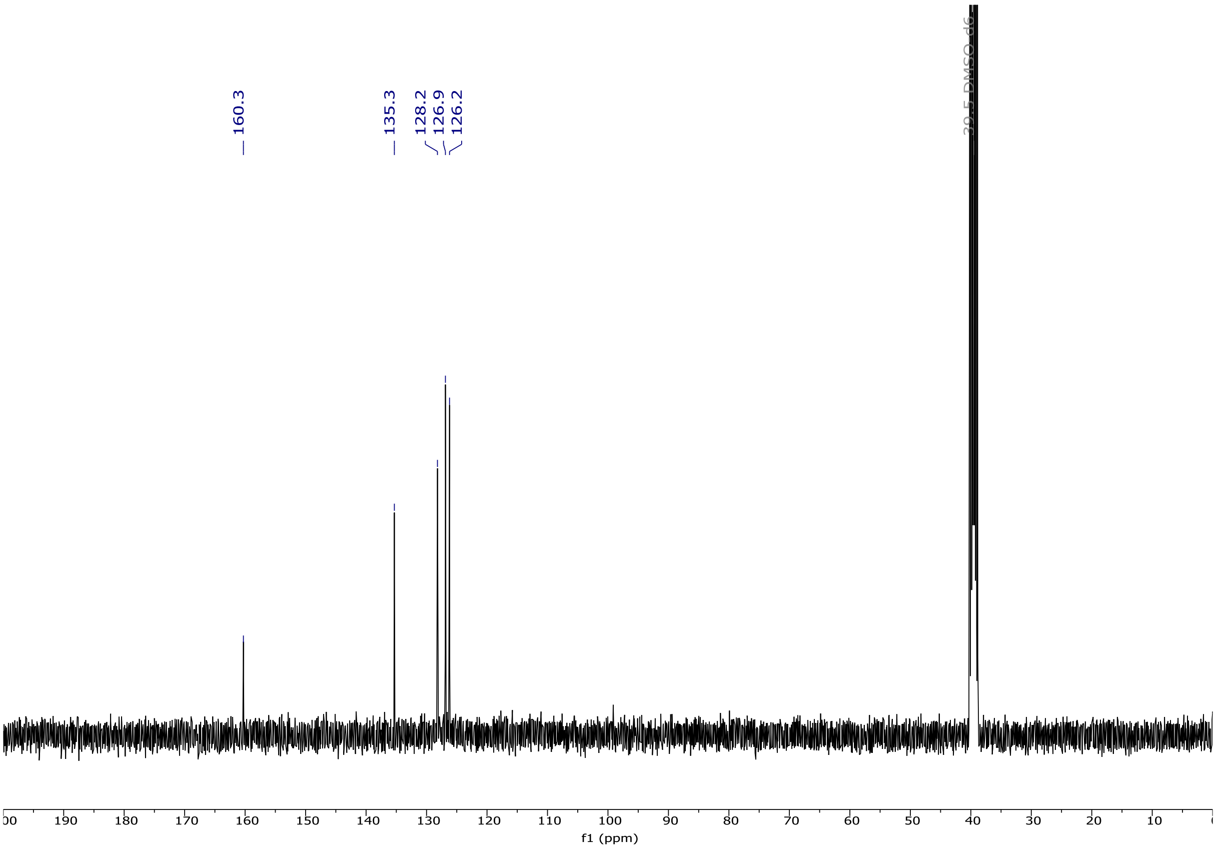

N-[{3,5-Bis(trifluoromethyl)benzoyl}oxy]-4-methylbenzamide

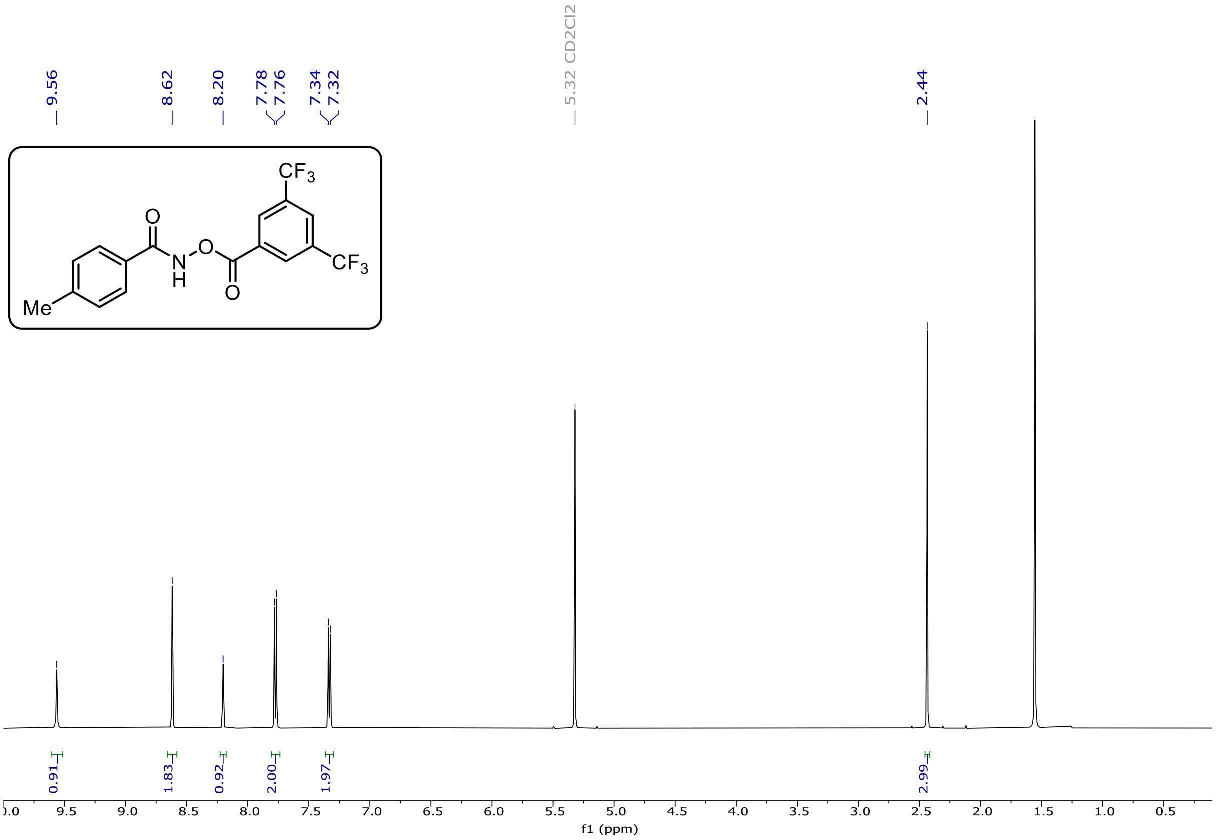

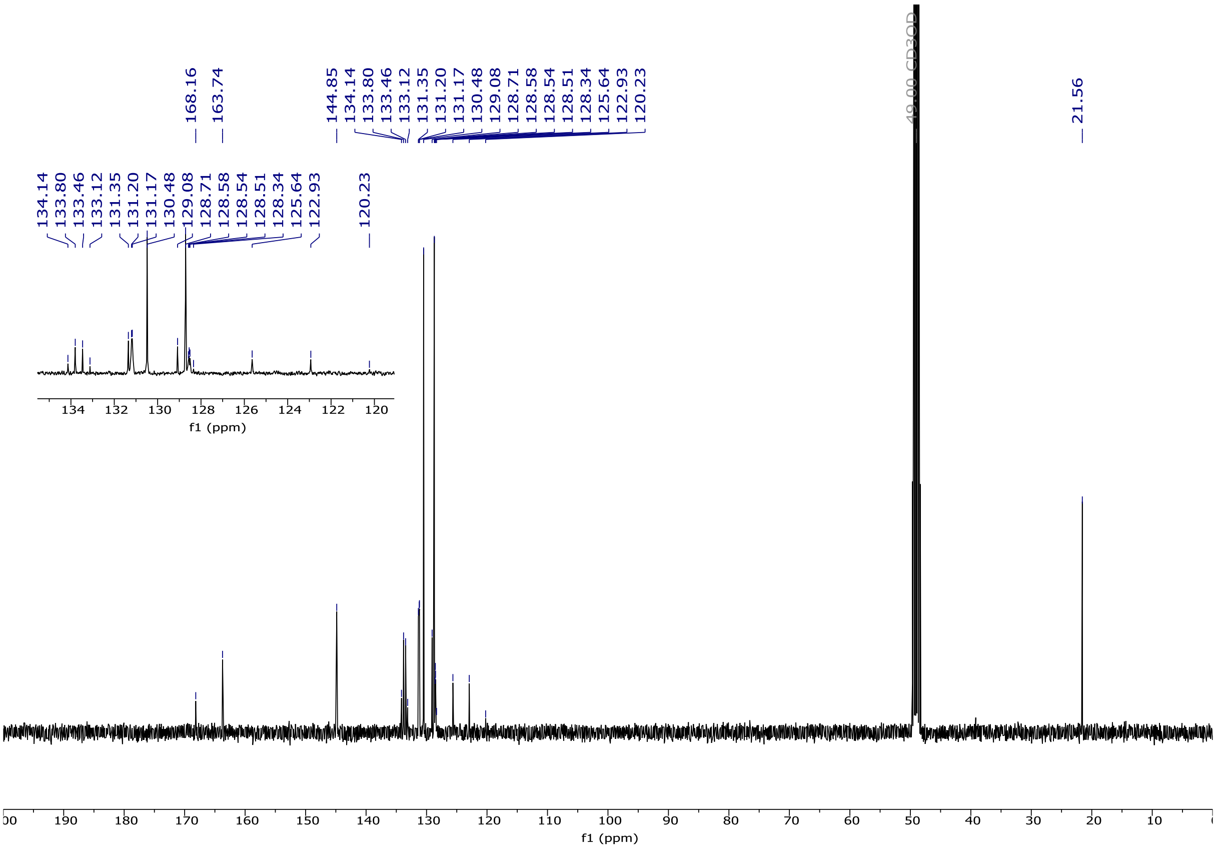

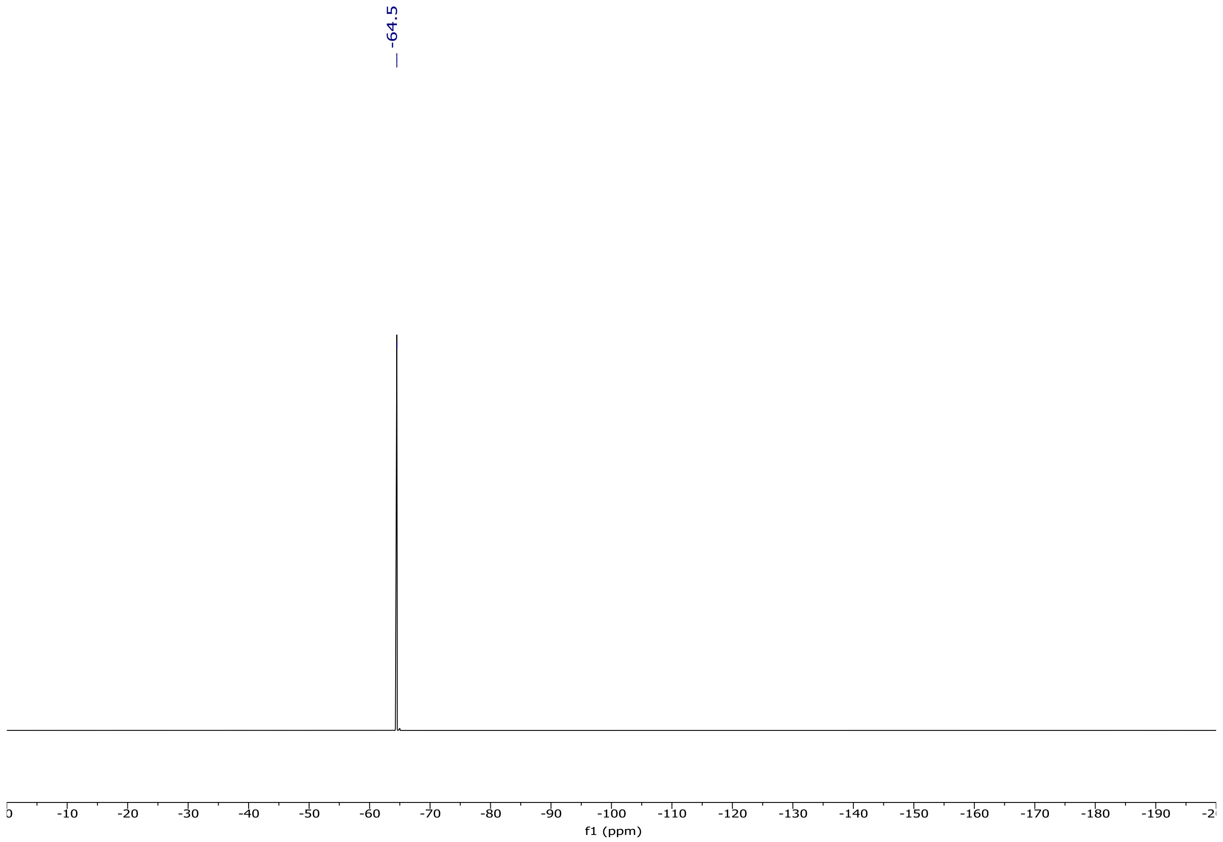
-64.5
0
-10
-20
-30
-40
-50
-60
-70
-80
-90
-100
-110
-120
-130
-140
-150
-160
-170
-180
-190
-2
f1 (ppm)

_N_-[{3,5-Bis(trifluoromethyl)benzoyl}oxy]-4-(_tert_-butyl)benzamide

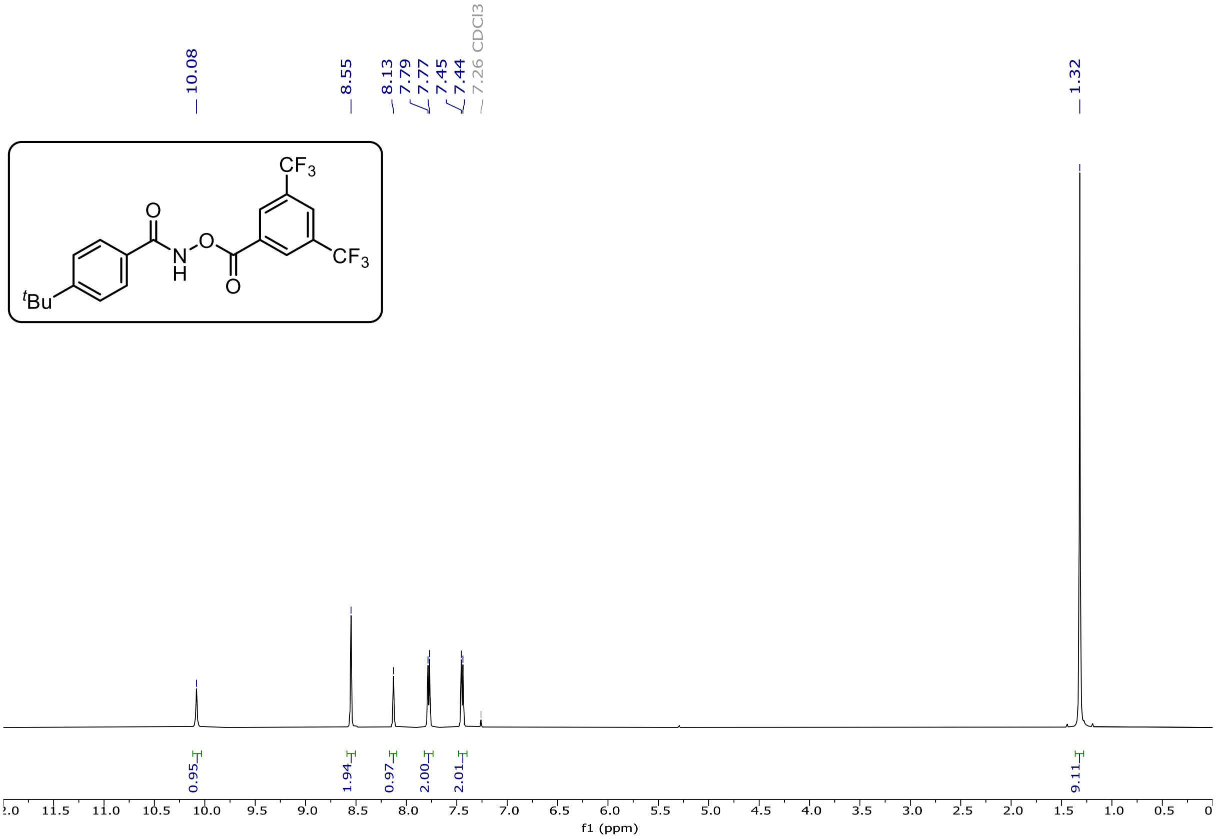

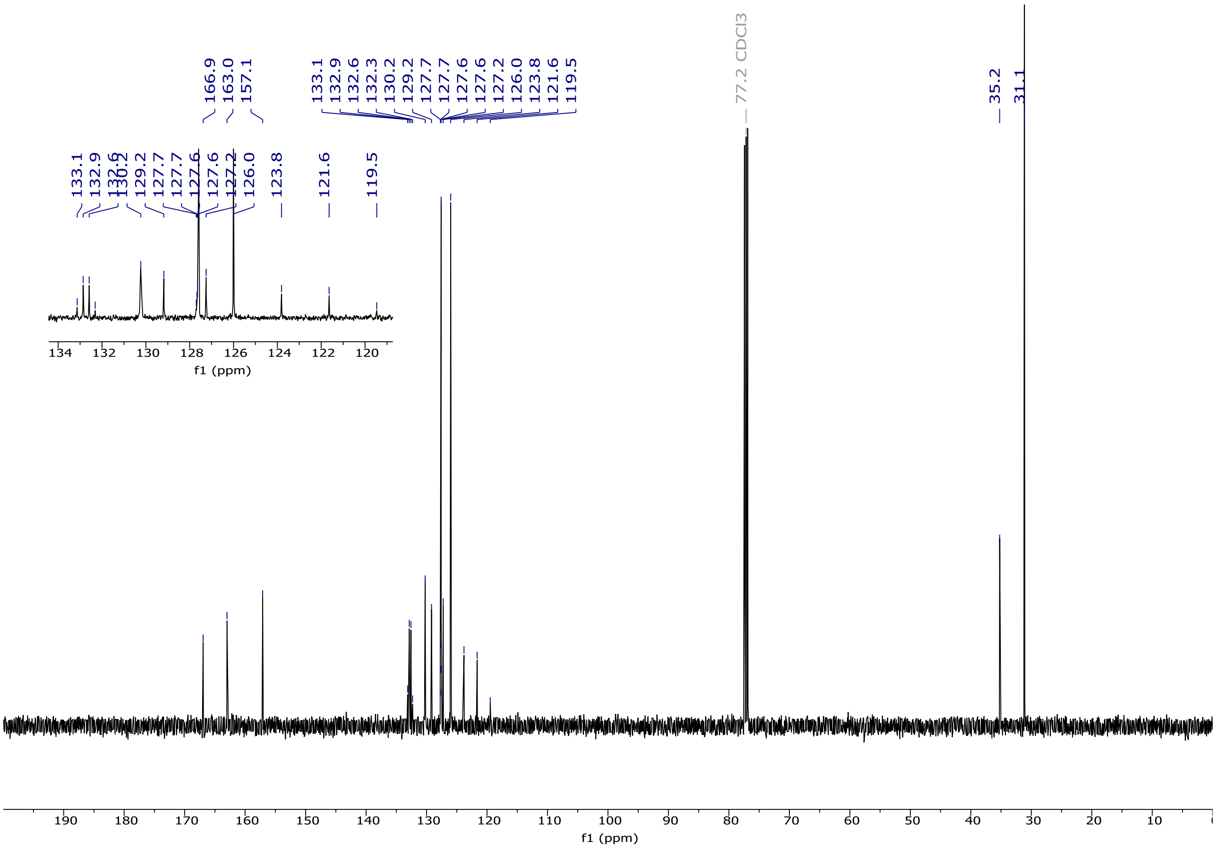

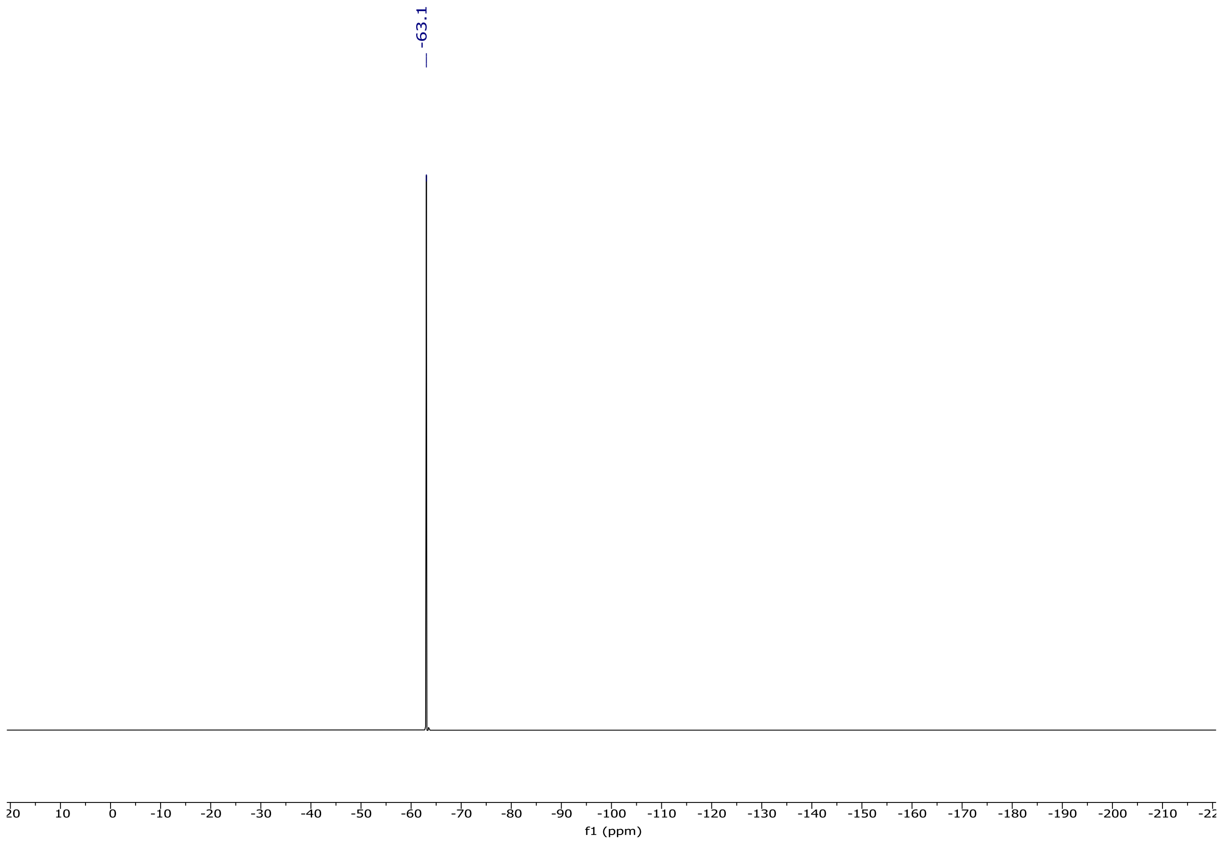
-63.1
20 10 0 -10 -20 -30 -40 -50 -60 -70 -80 -90 -100 -110 -120 -130 -140 -150 -160 -170 -180 -190 -200 -210
f1 (ppm)

N-[{3,5-Bis(trifluoromethyl)benzoyl}oxy]-4-chlorobenzamide

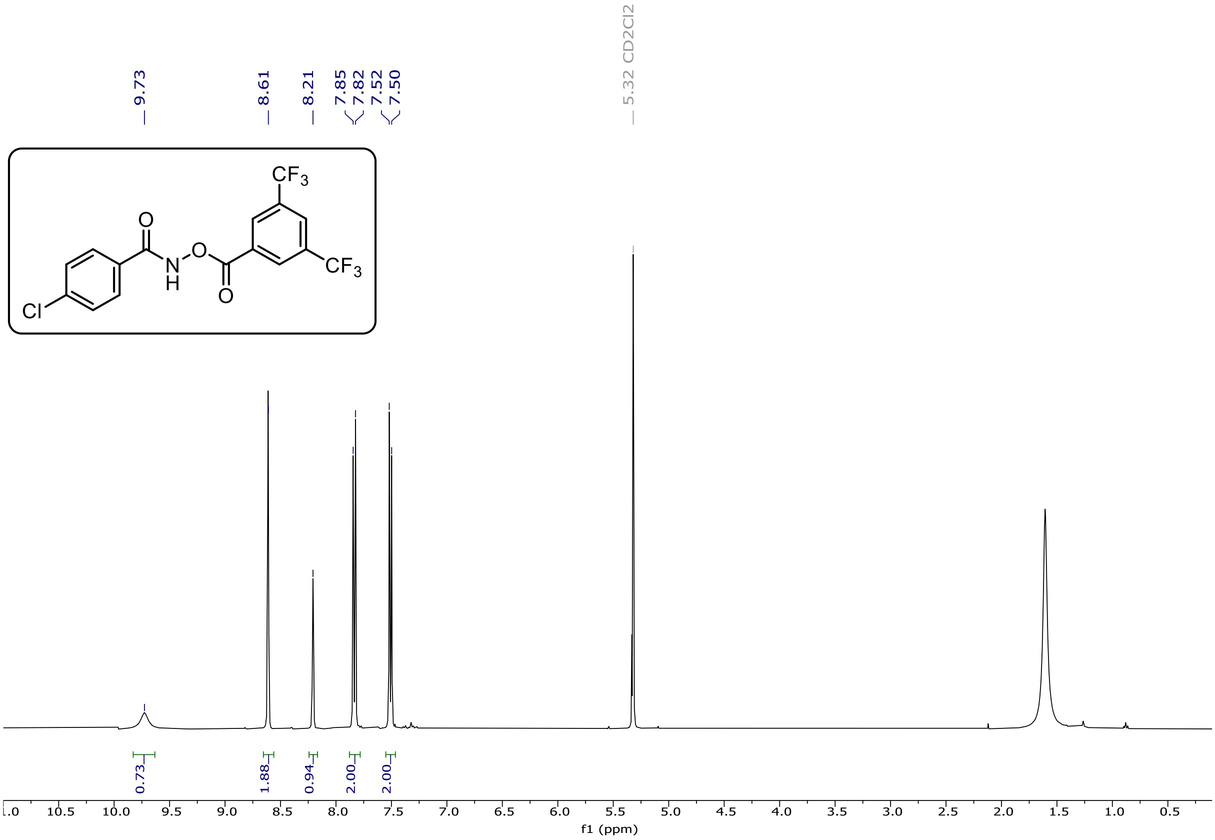

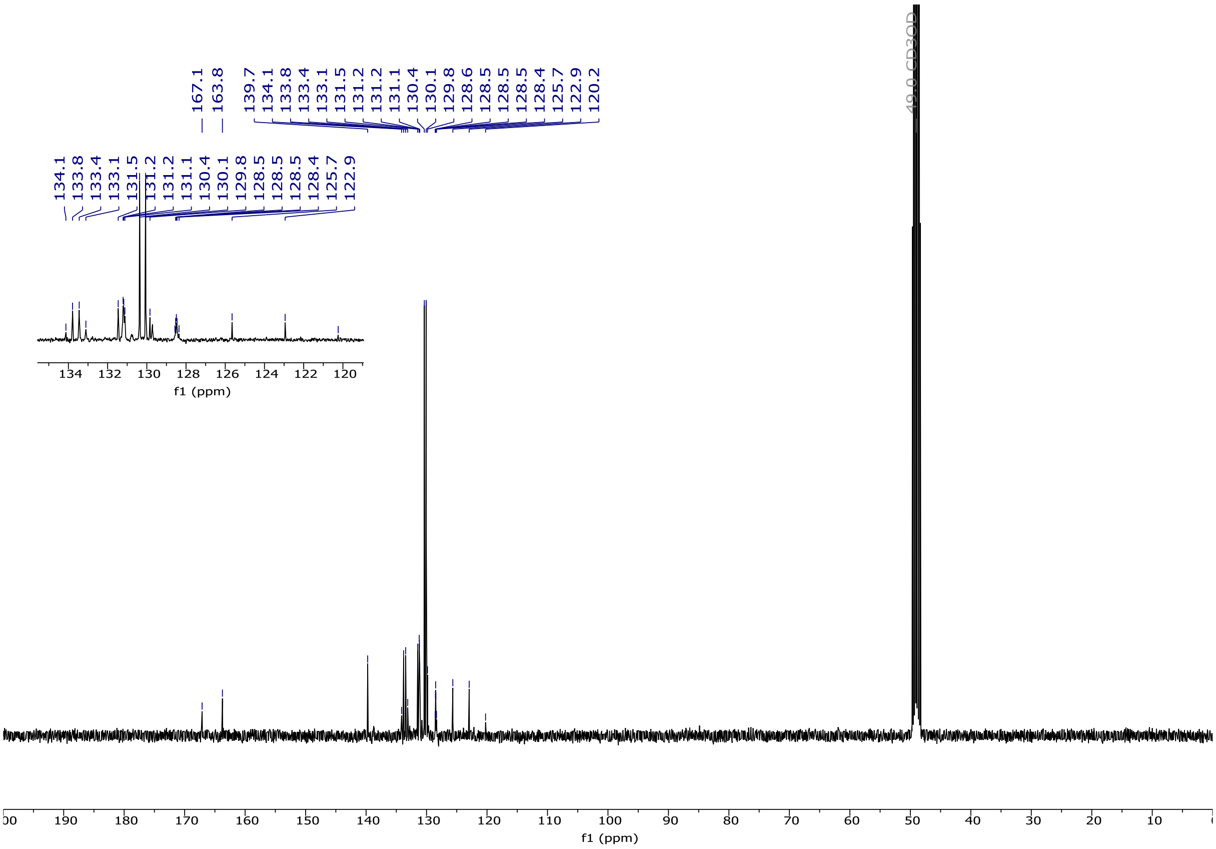

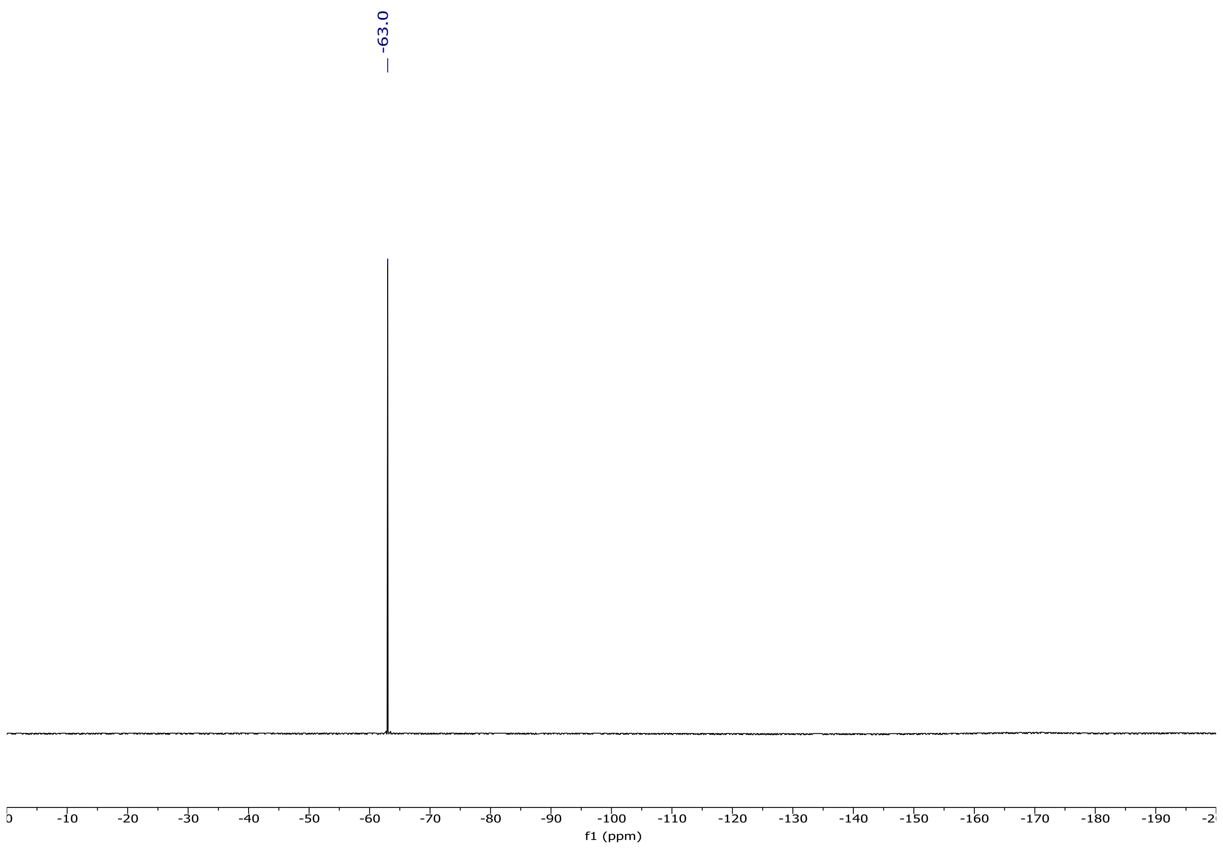
-63.0
f1 (ppm)

N-[{3,5-Bis(trifluoromethyl)benzoyl}oxy]-4-bromobenzamide

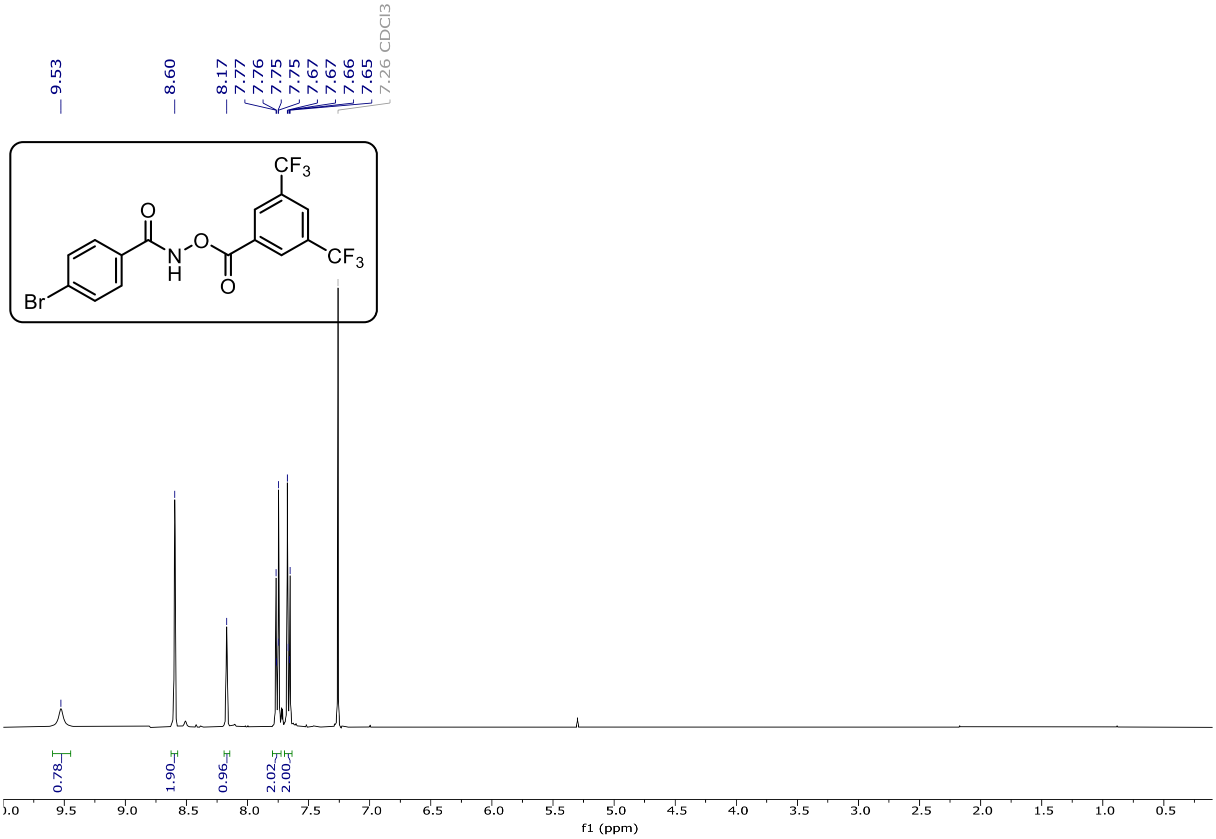

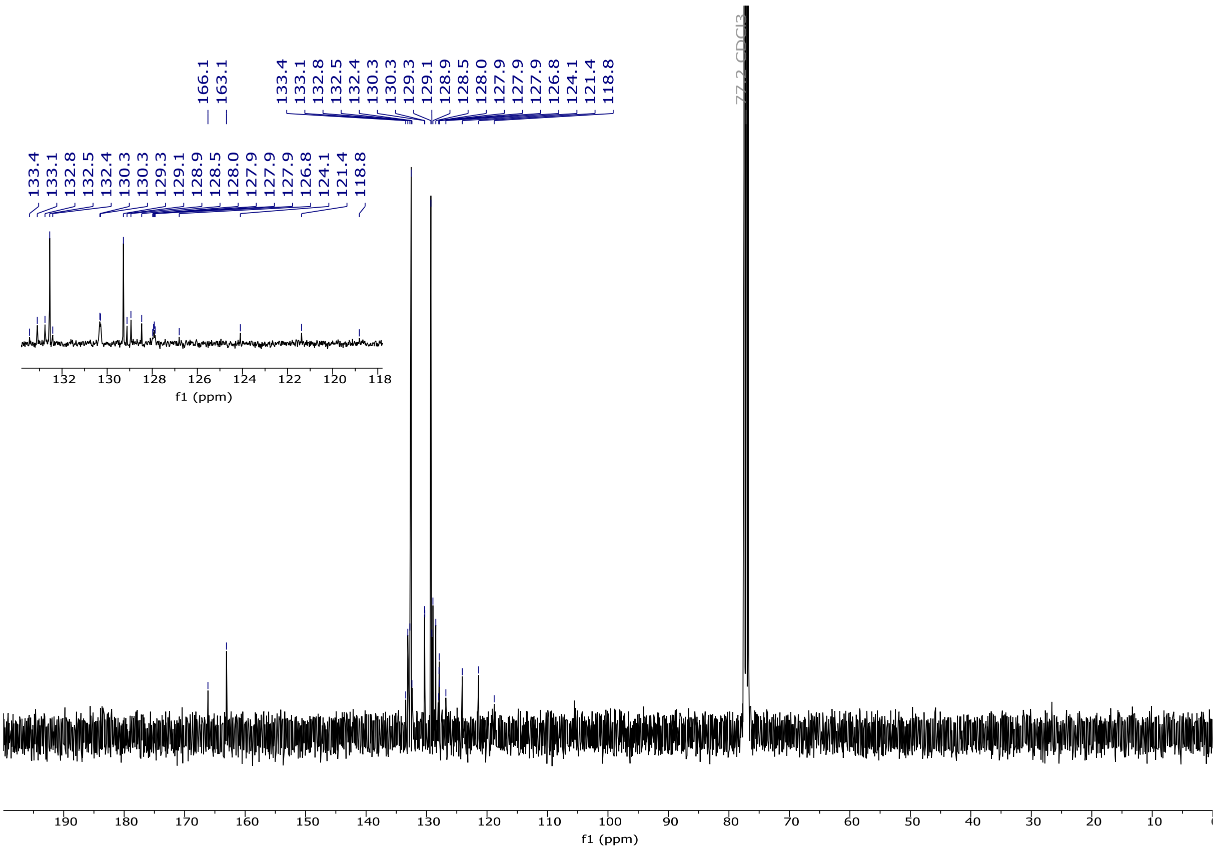

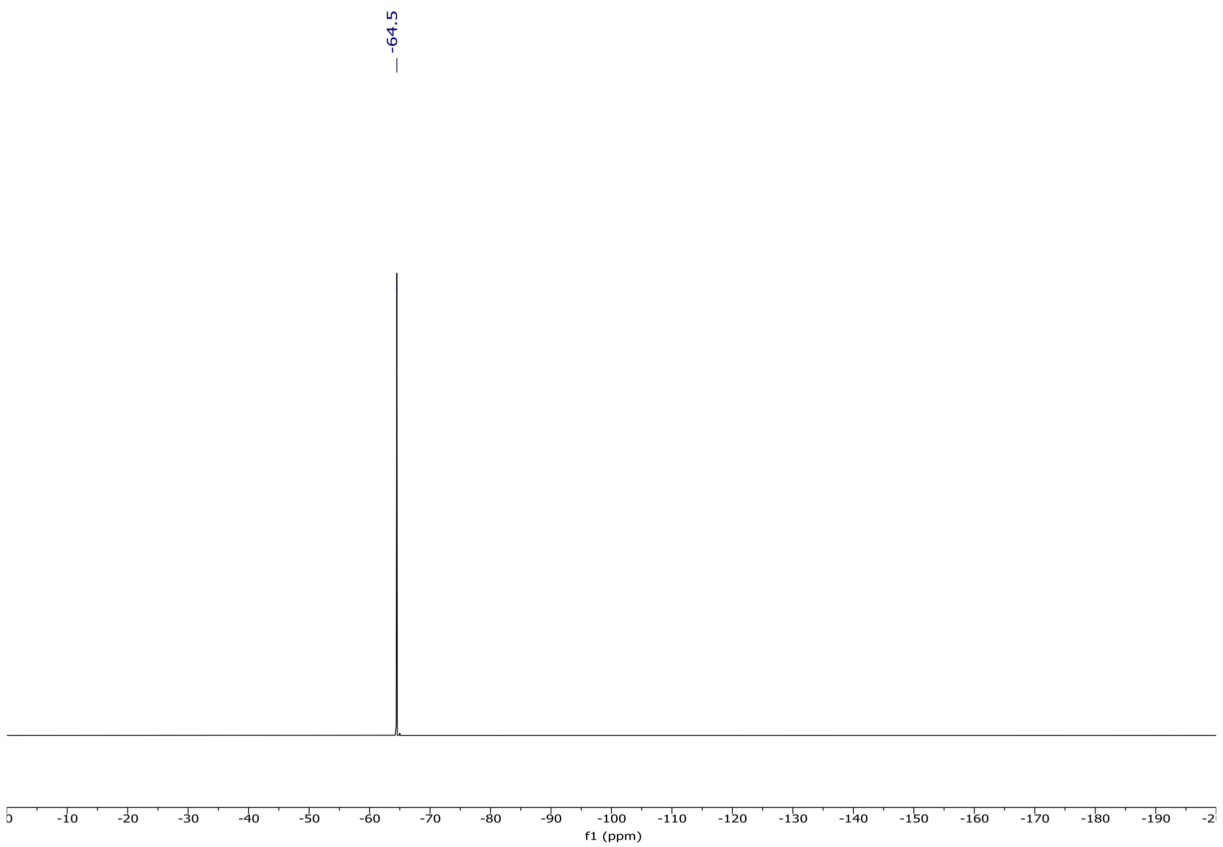
-64.5
f1 (ppm)

N-[{3,5-Bis(trifluoromethyl)benzoyl}oxy]cyclohexanecarboxamide

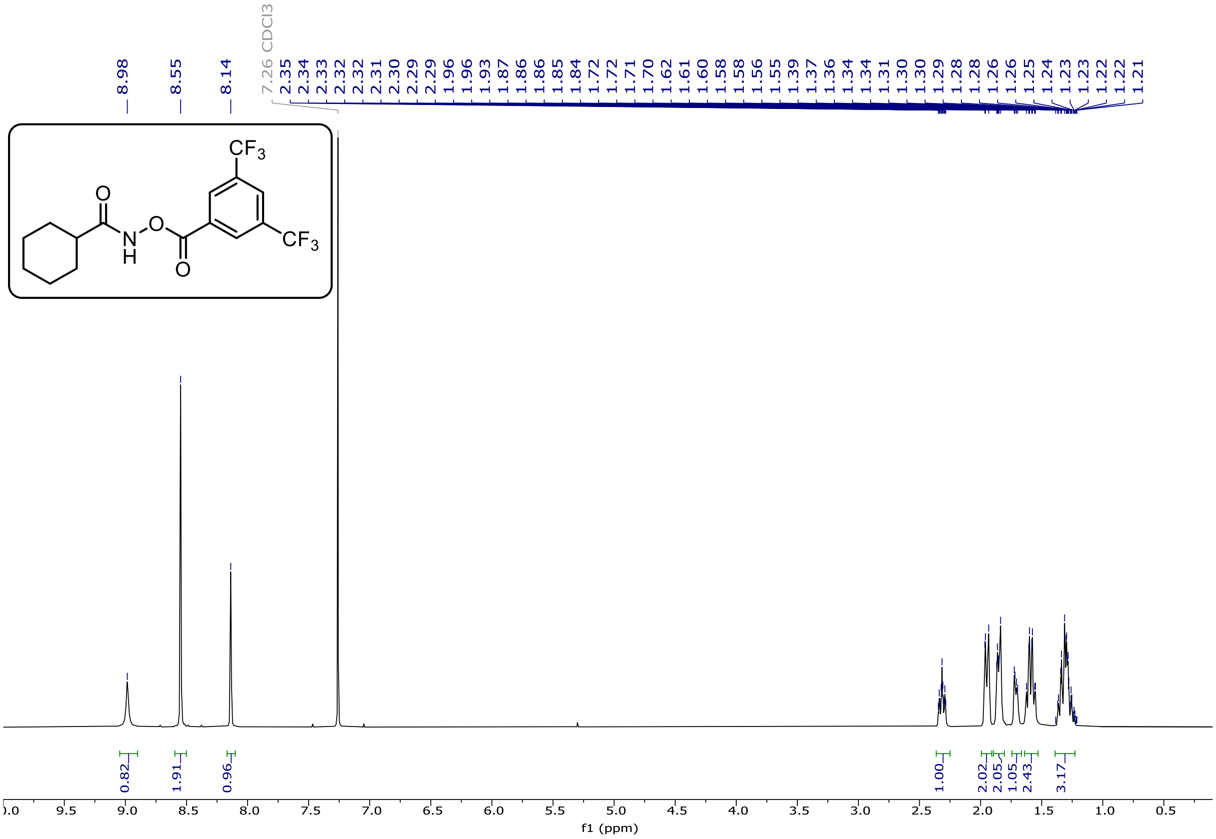

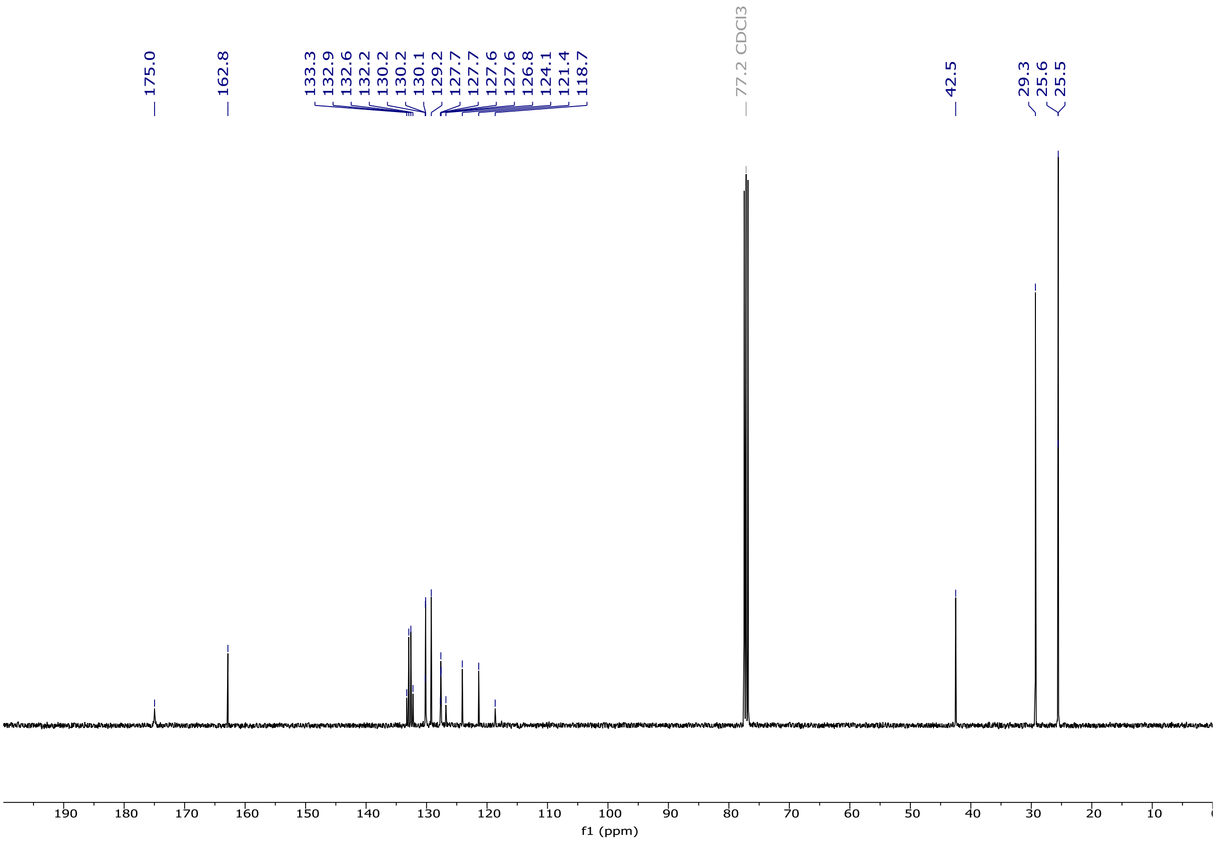

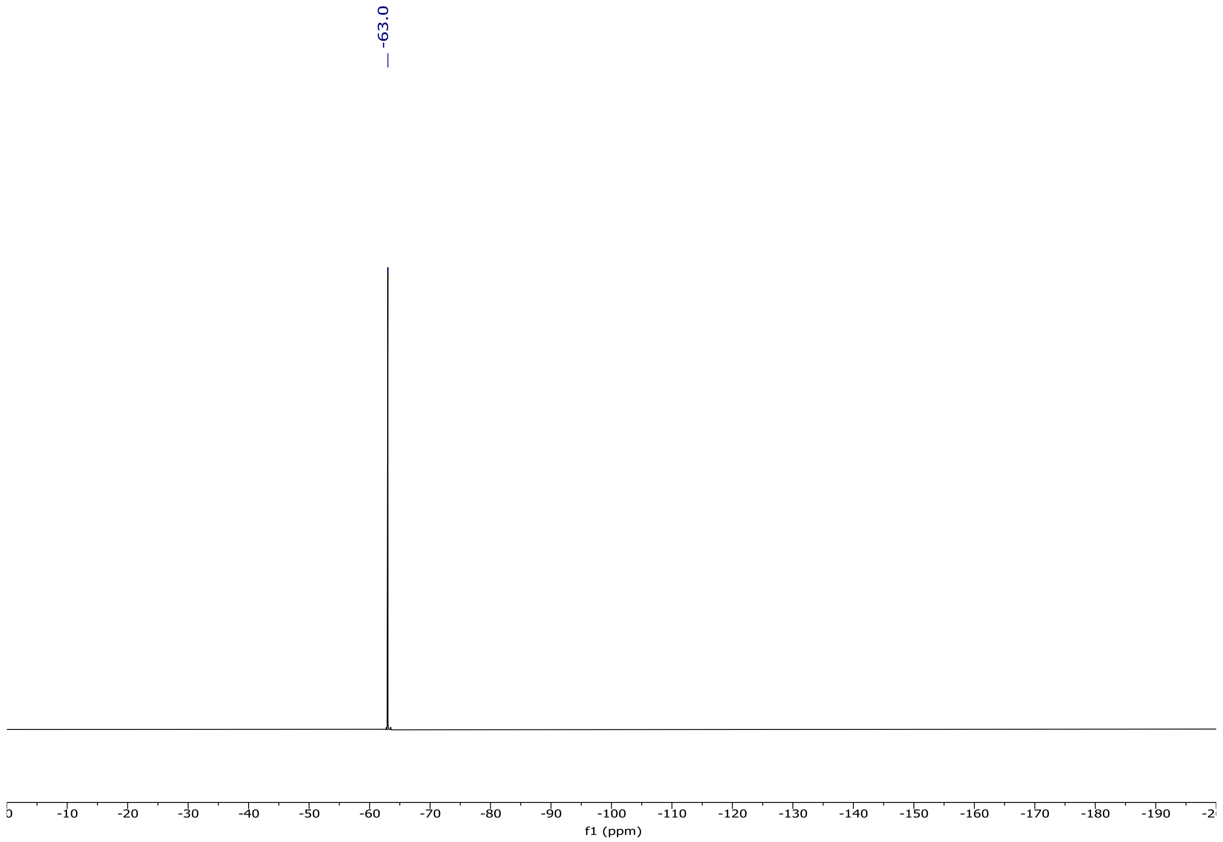

-63.0
0
-10
-20
-30
-40
-50
-60
-70
-80
-90
-100
-110
-120
-130
-140
-150
-160
-170
-180
-190
-2
f1 (ppm)

***N*-[{3,5-Bis(trifluoromethyl)benzoyl}oxy]thiophene-3-carboxamide**

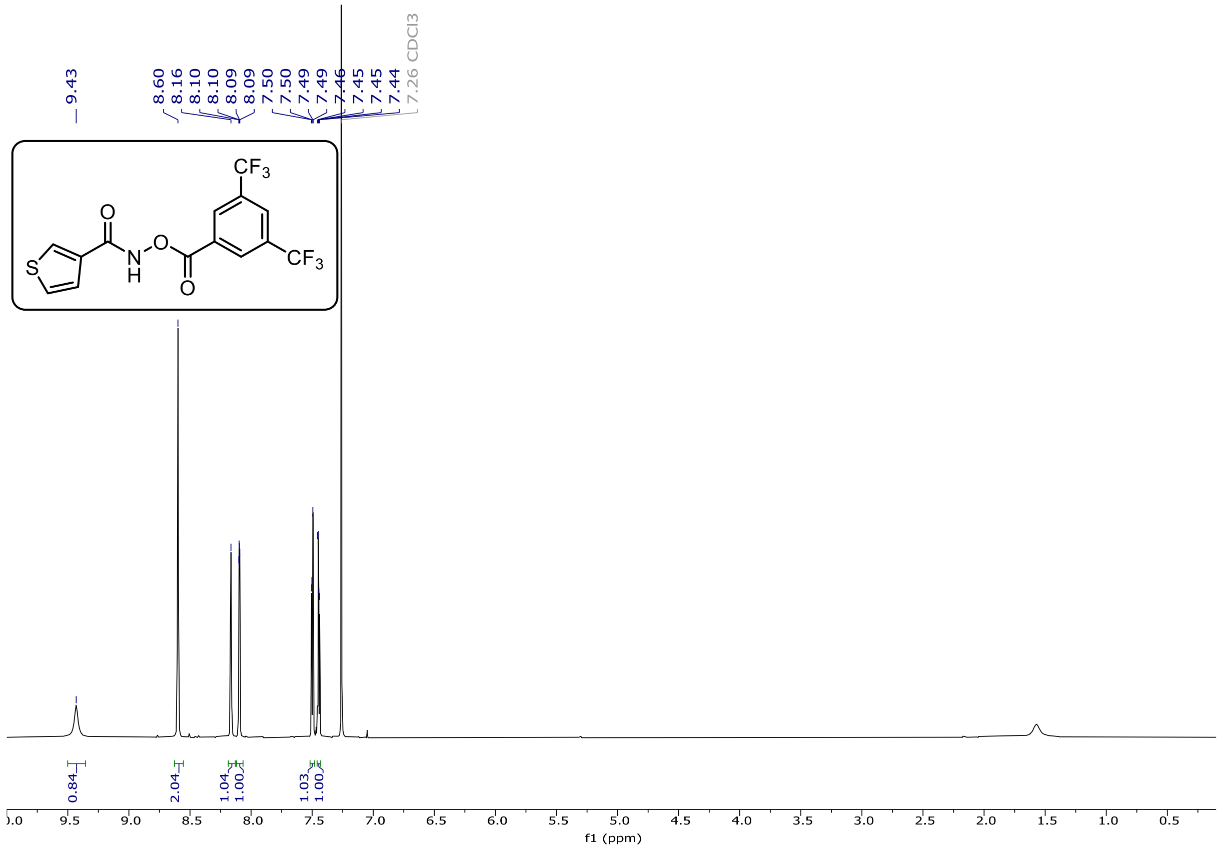

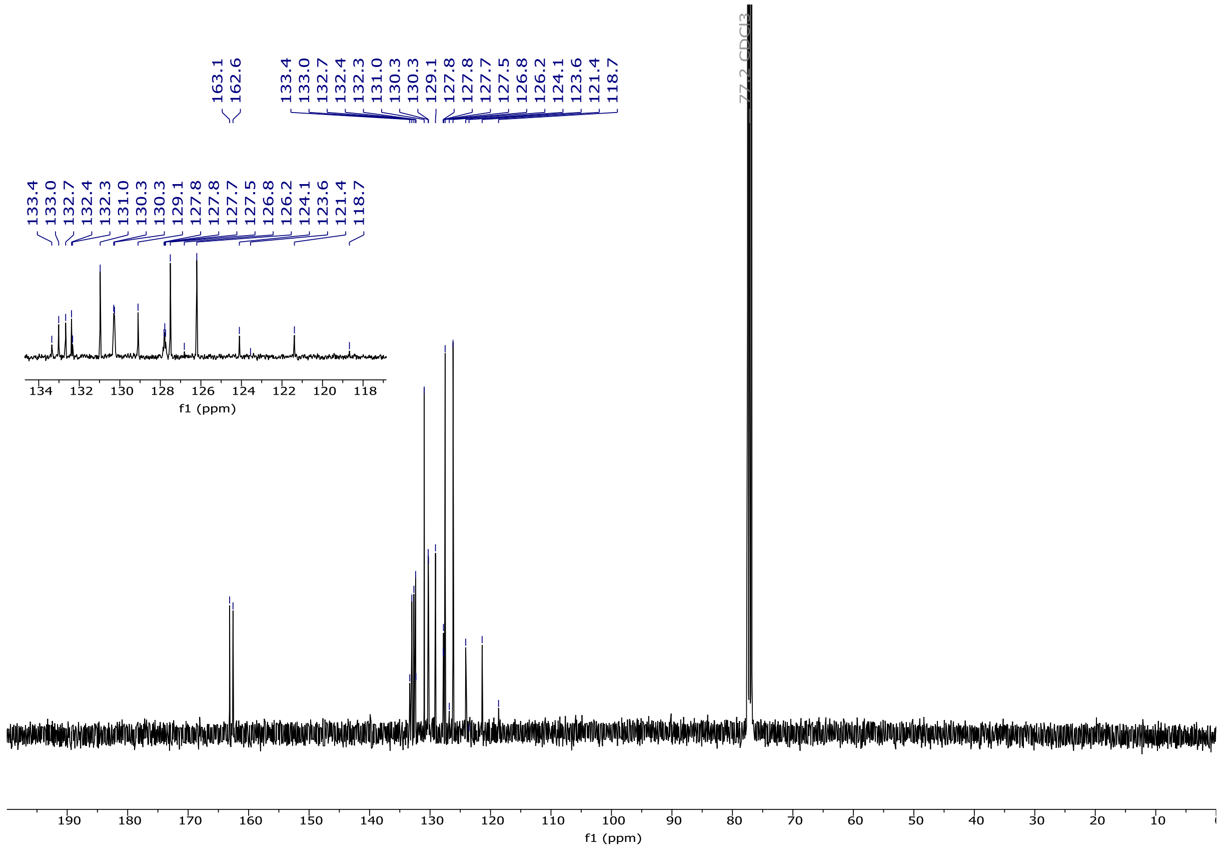

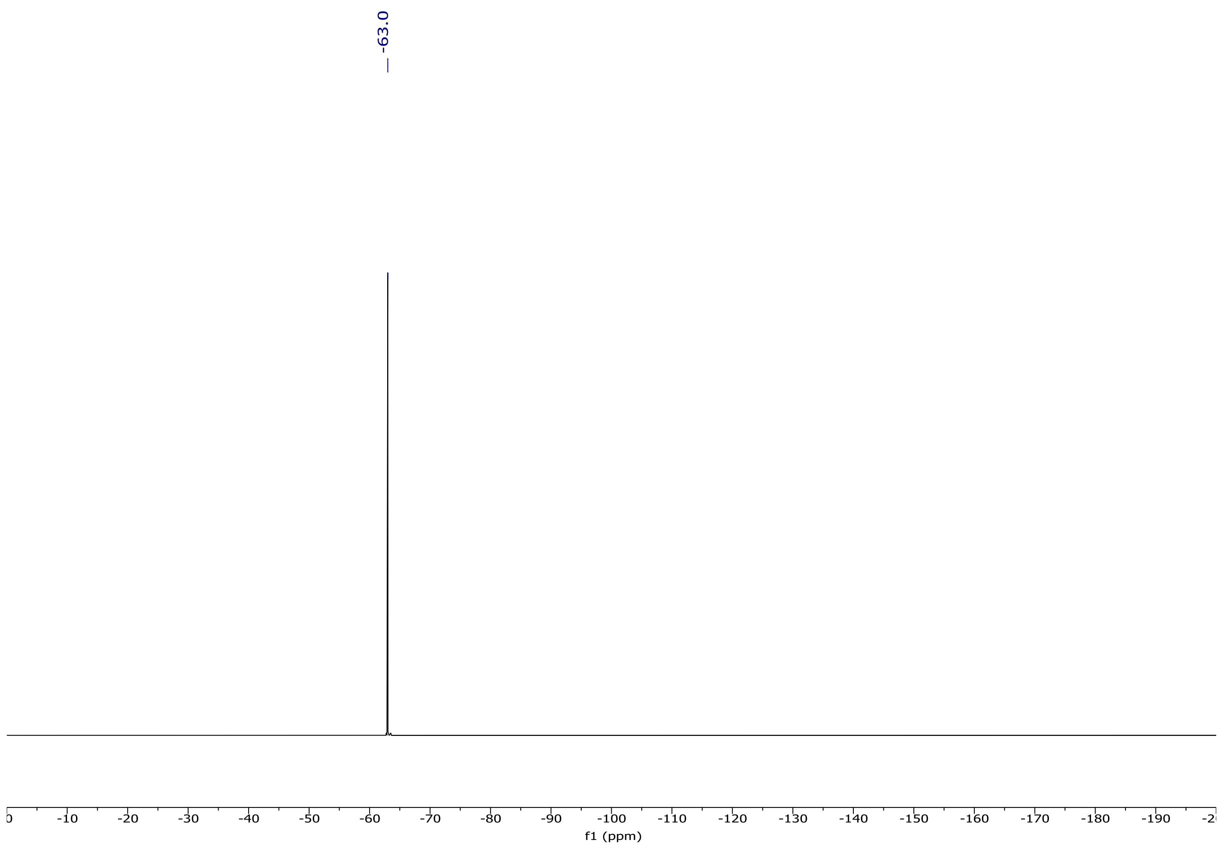

-63.0
0
-10
-20
-30
-40
-50
-60
-70
-80
-90
-100
-110
-120
-130
-140
-150
-160
-170
-180
-190
f1 (ppm)

N-[{3,5-bis(trifluoromethyl)benzoyl}oxy]-4-methoxybenzamide

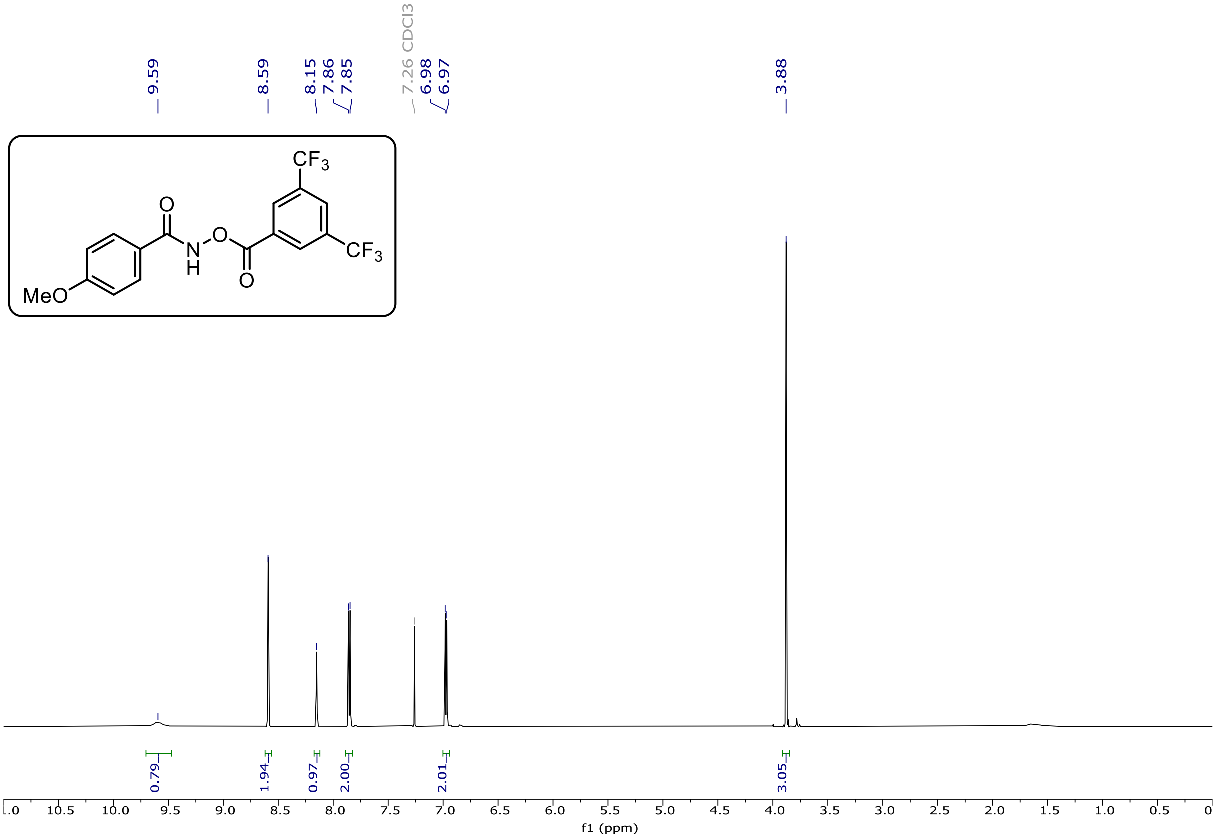

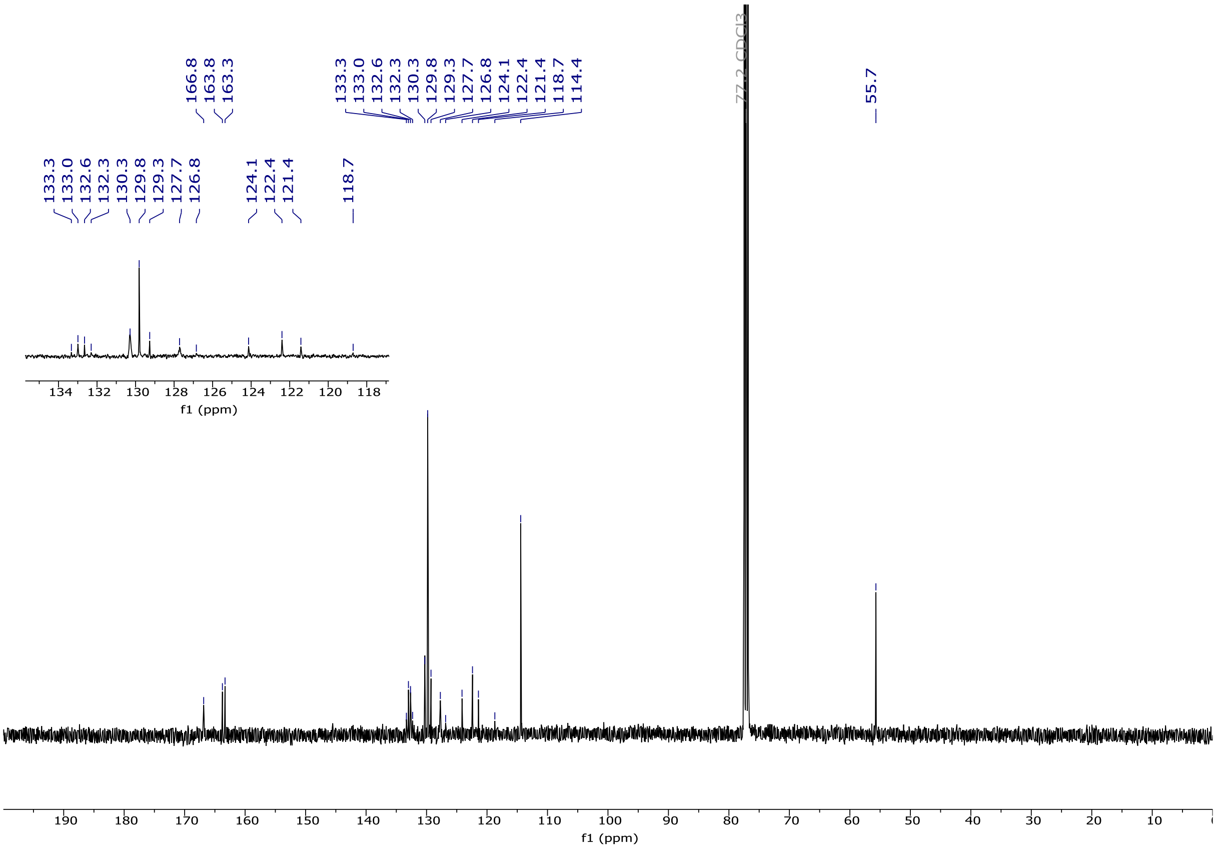

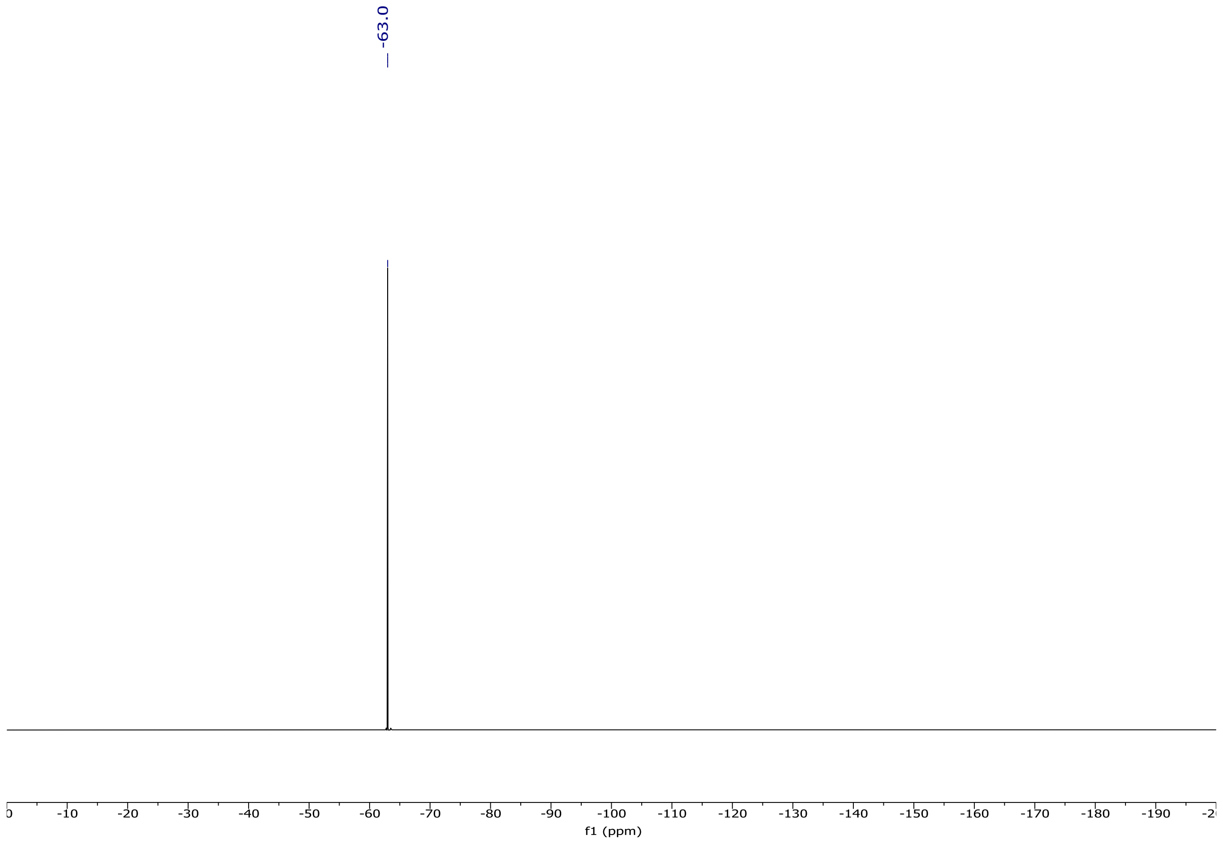
-63.0
0
-10
-20
-30
-40
-50
-60
-70
-80
-90
-100
-110
-120
-130
-140
-150
-160
-170
-180
-190
f1 (ppm)

***N*-[{3,5-Bis(trifluoromethyl)benzoyl}oxy]acetamide (1e)**

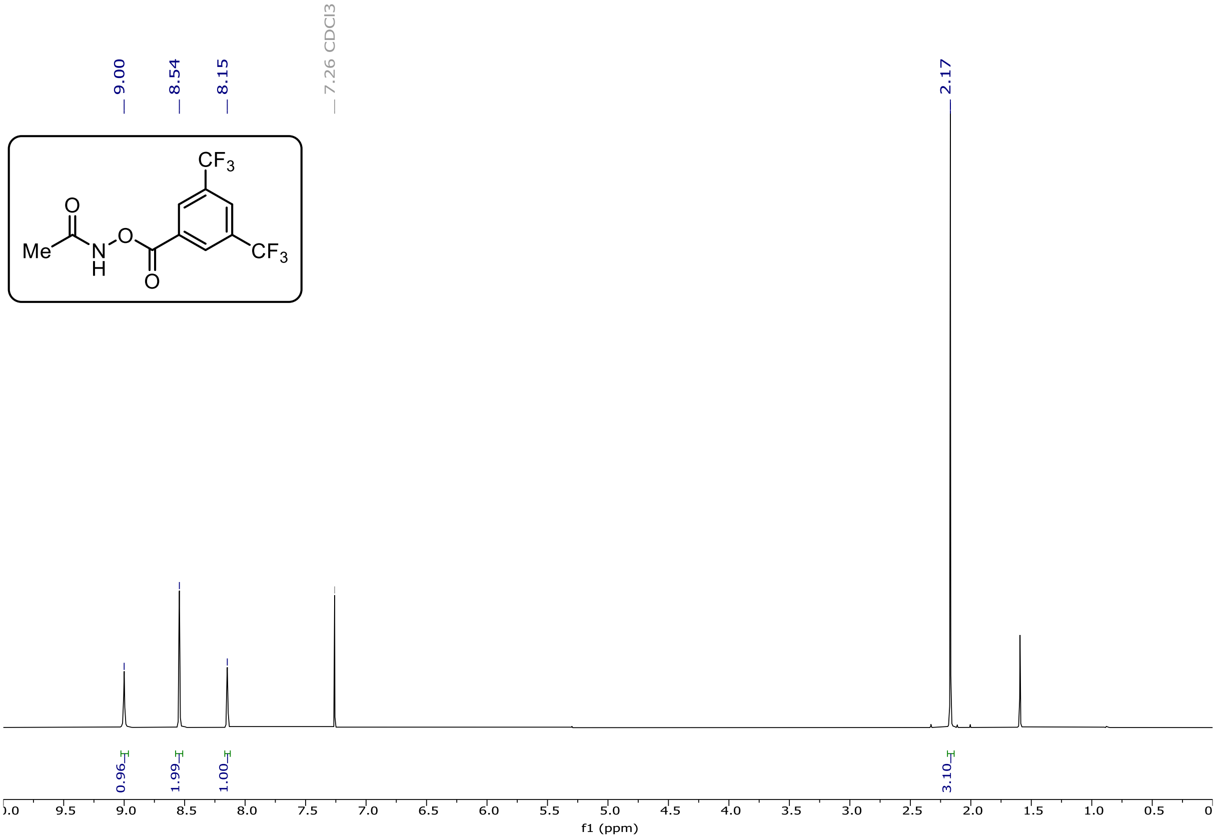

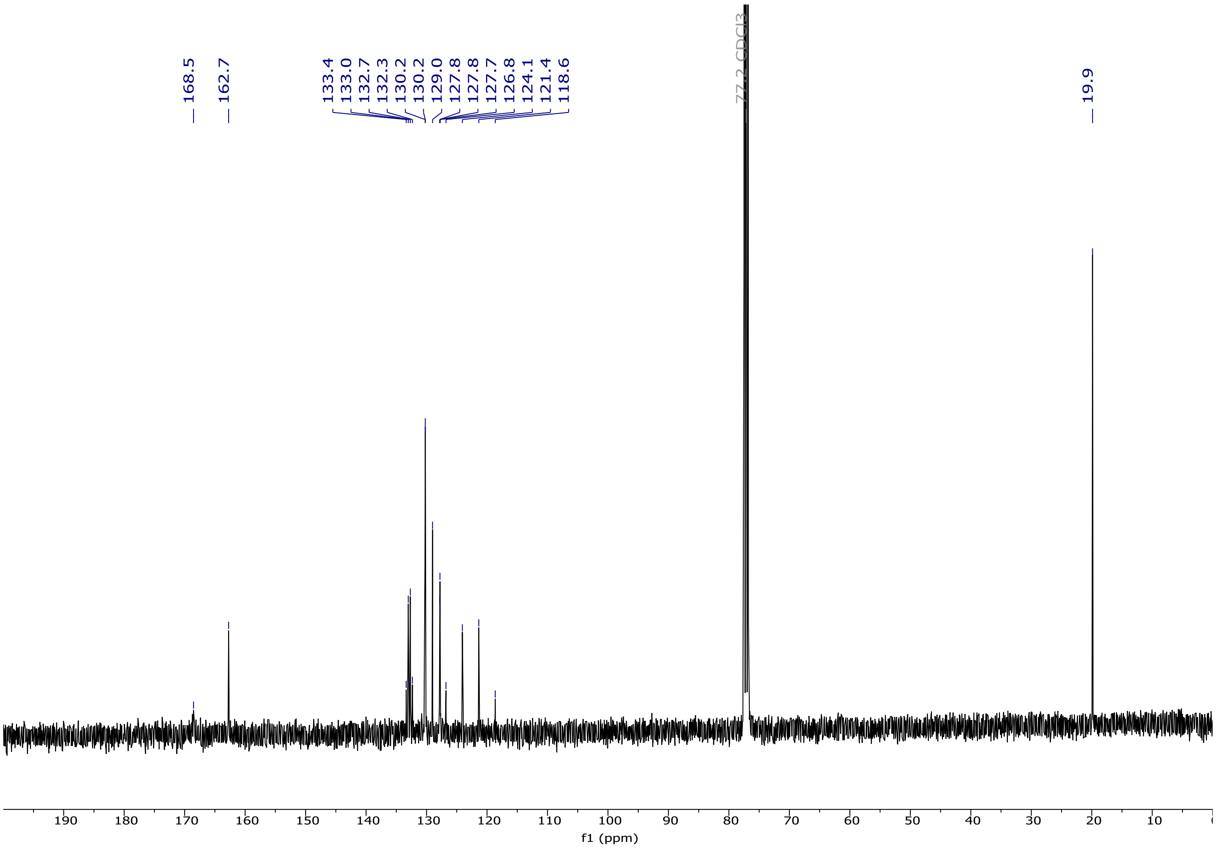

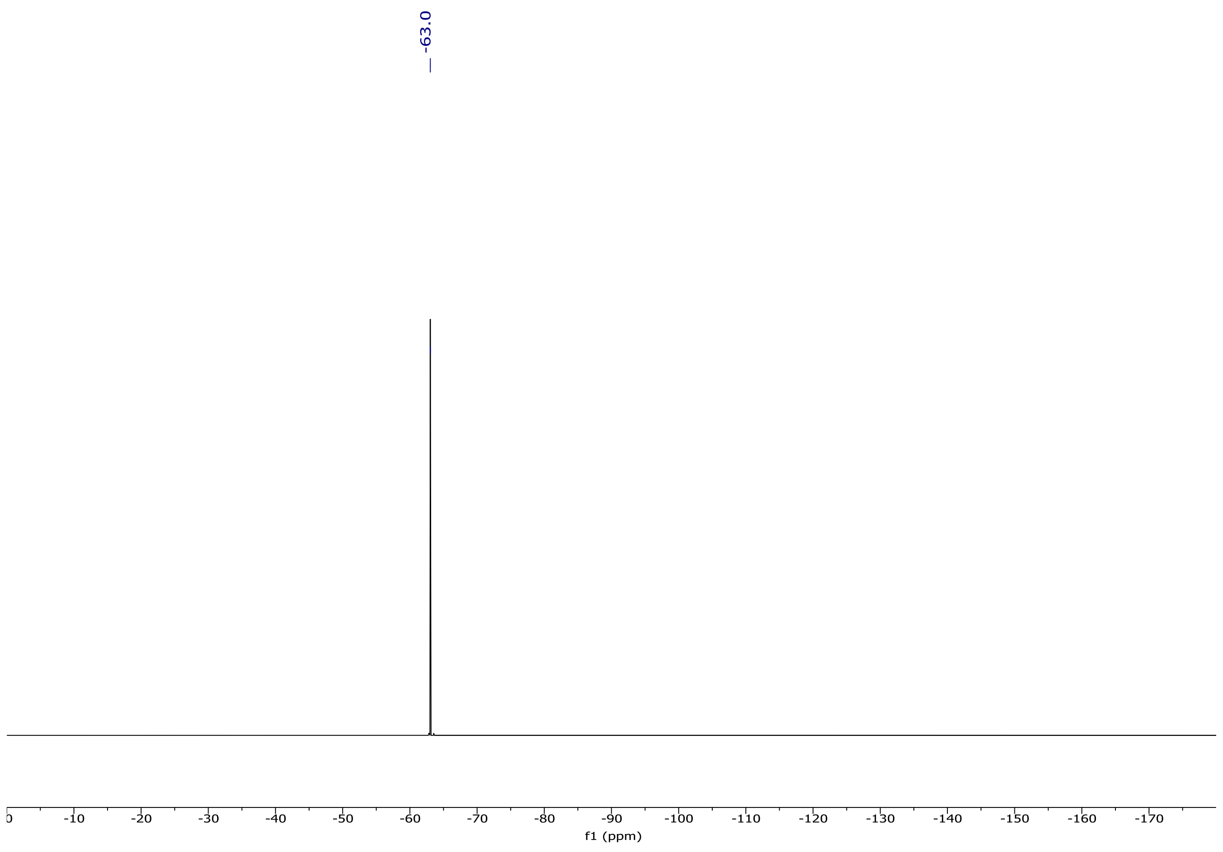
-63.0
0
-10
-20
-30
-40
-50
-60
-70
-80
-90
-100
-110
-120
-130
-140
-150
-160
-170
f1 (ppm)

N-(2,4-Dinitrophenoxy)benzamide (1d)

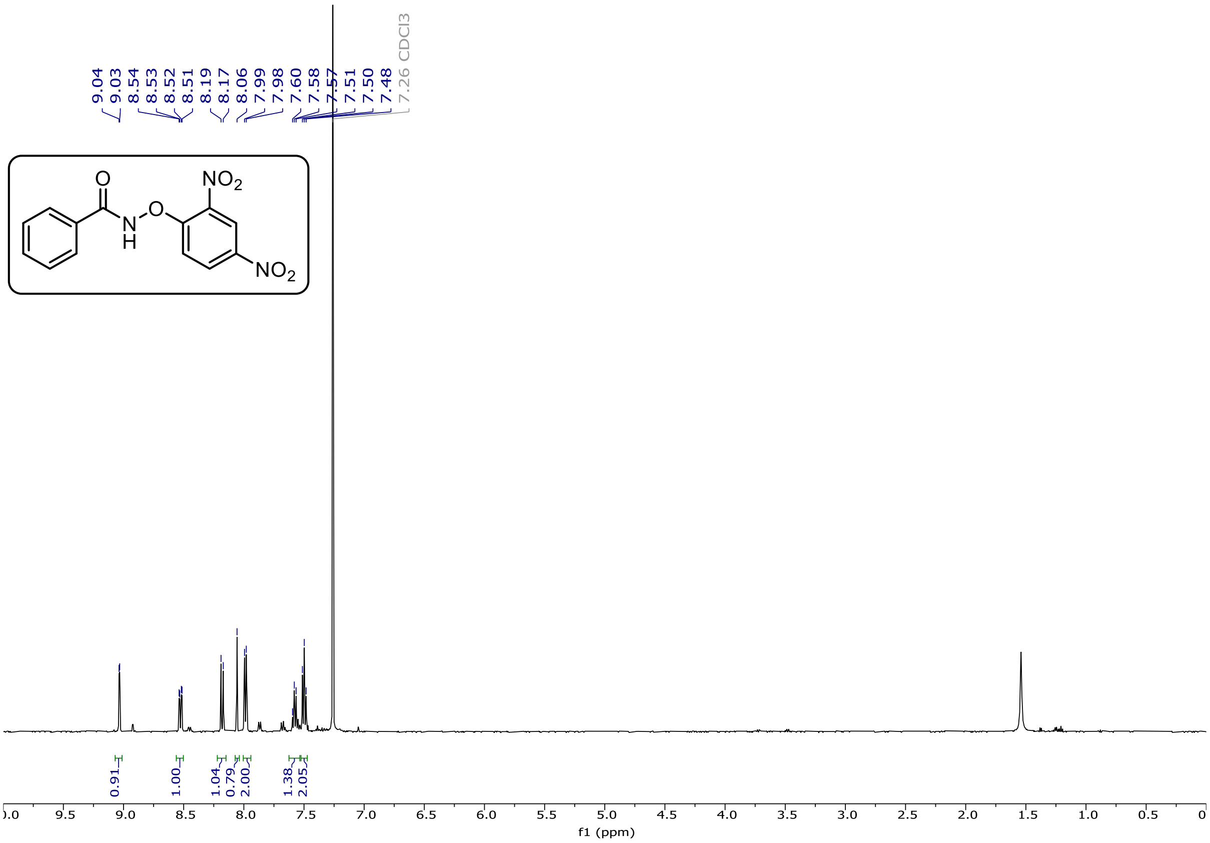

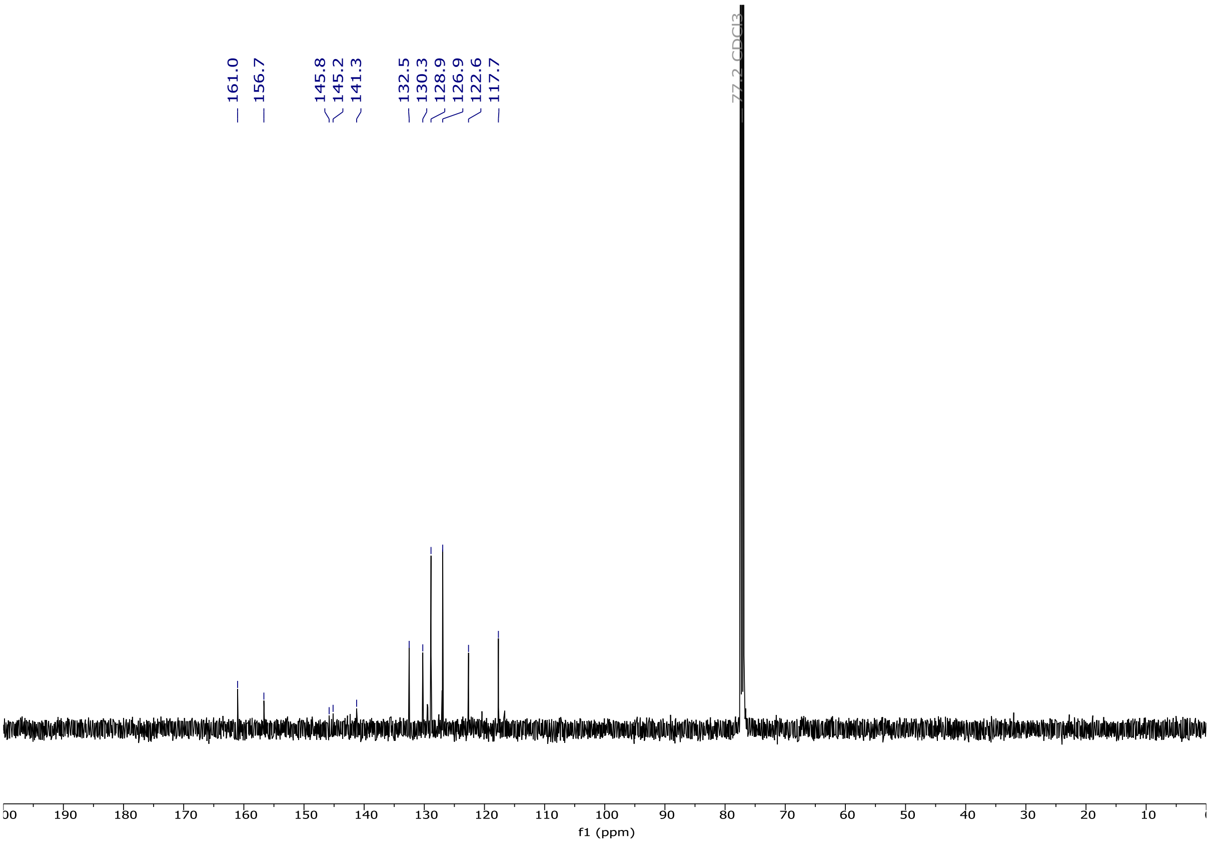

2-Benzamido-1-(4-methoxyphenyl)ethyl 3,5-bis(trifluoromethyl)benzoate (3a)

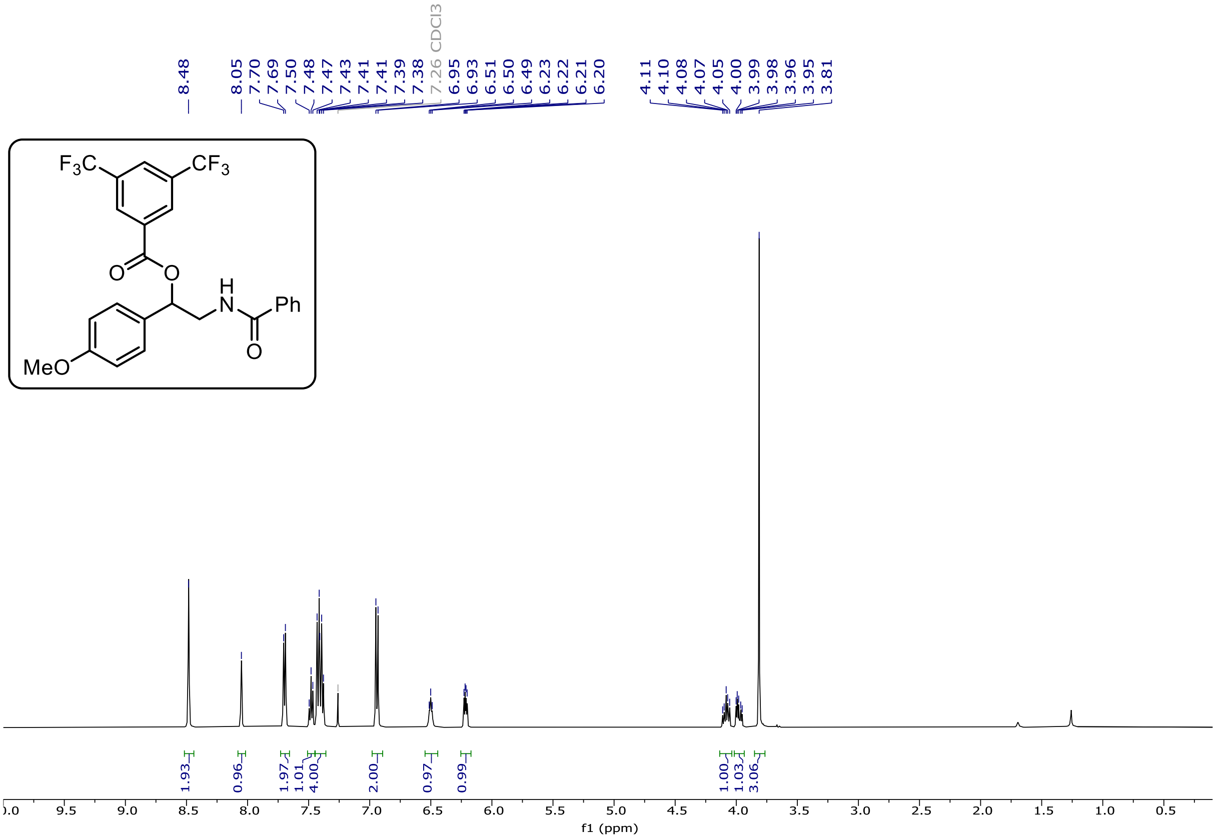

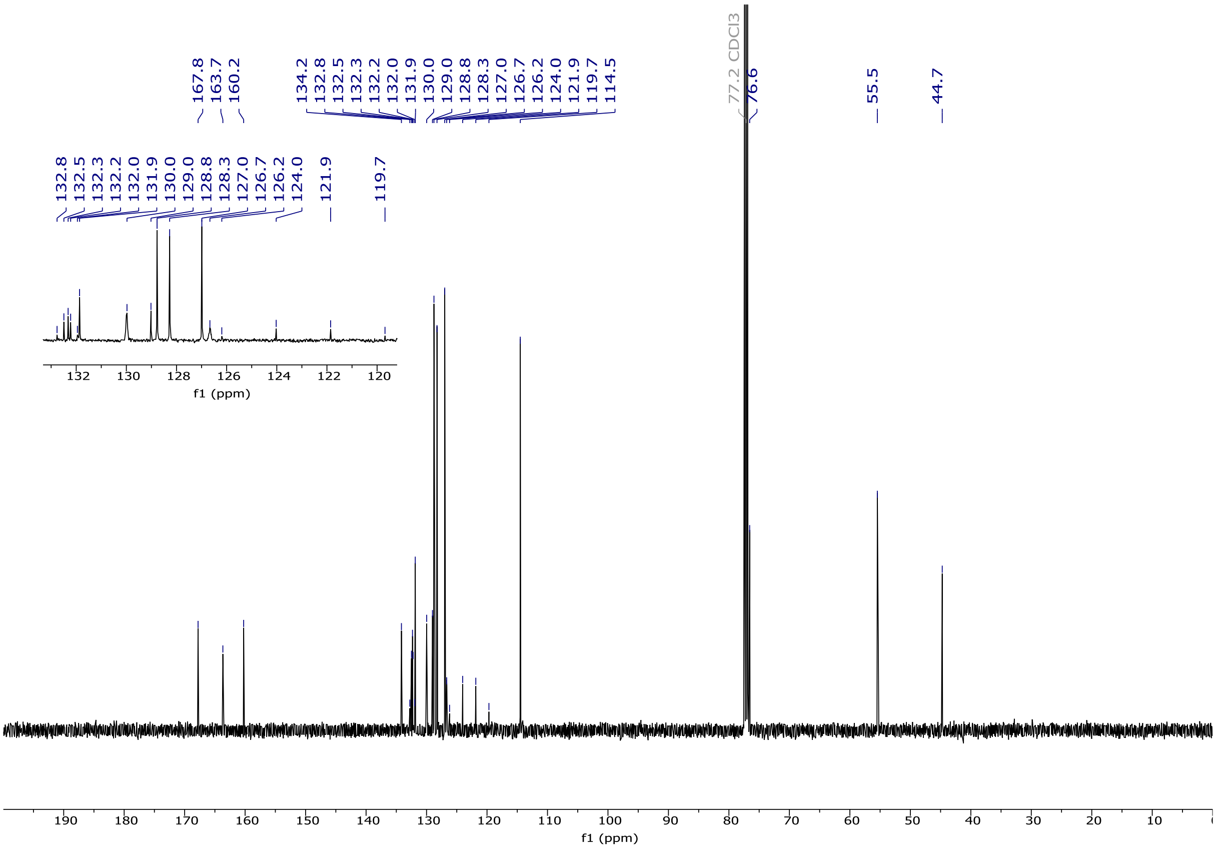

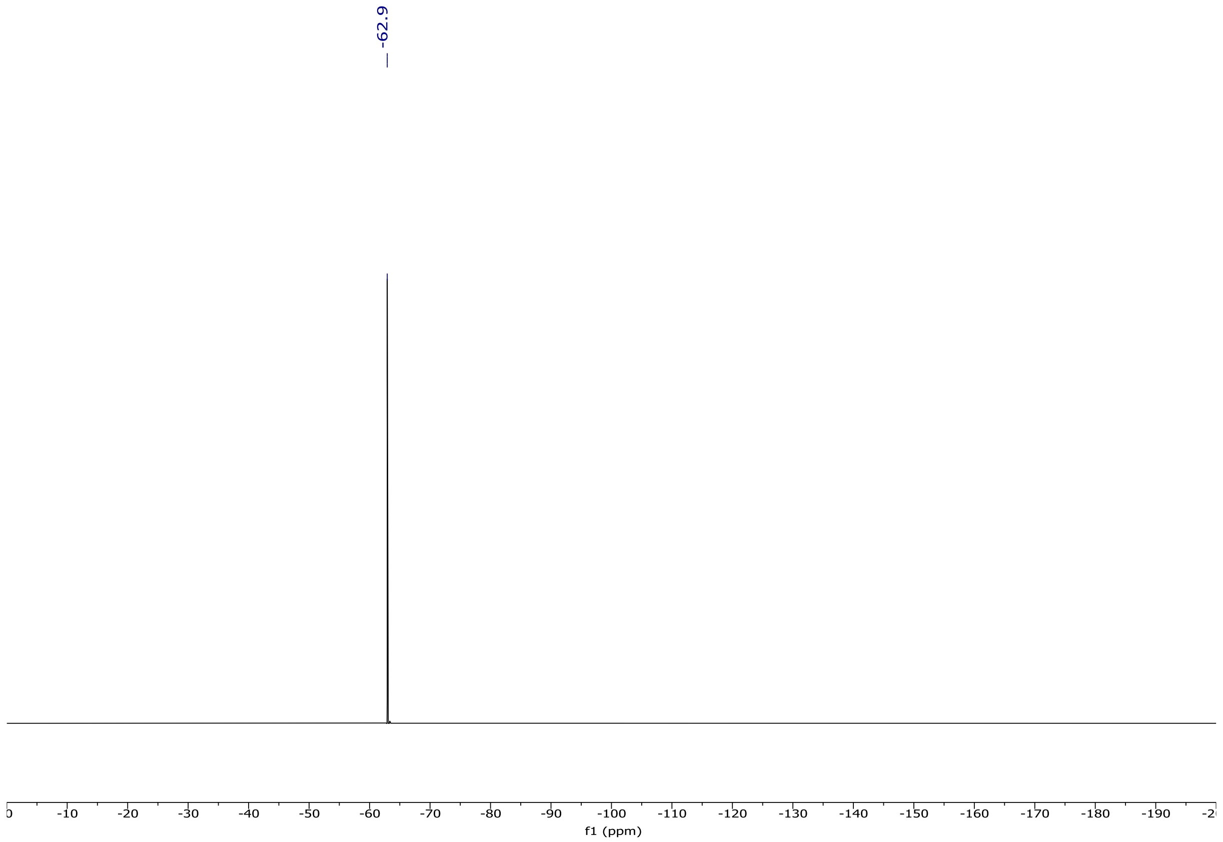
-62.9
0
-10
-20
-30
-40
-50
-60
-70
-80
-90
-100
-110
-120
-130
-140
-150
-160
-170
-180
-190
f1 (ppm)

2-Benzamido-1-(4-ethoxyphenyl)ethyl 3,5-bis(trifluoromethyl)benzoate (3b)

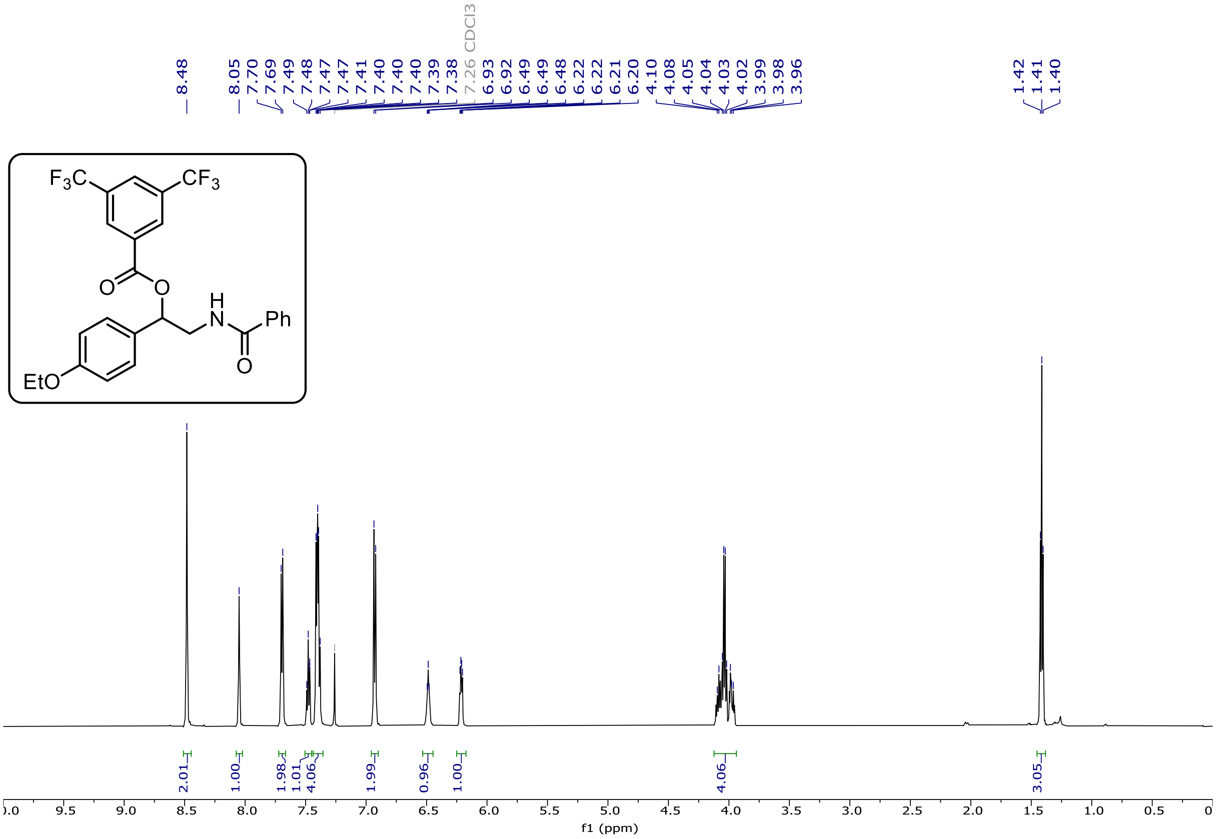

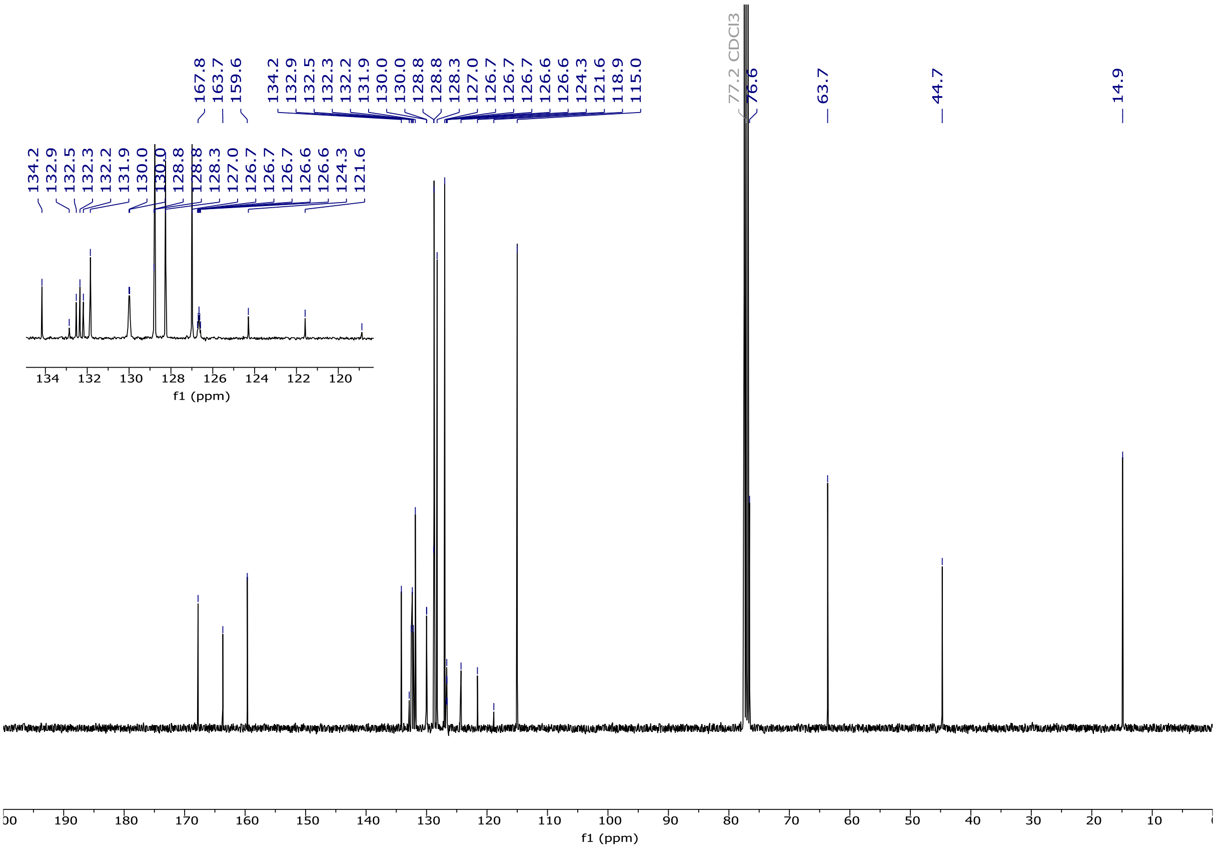

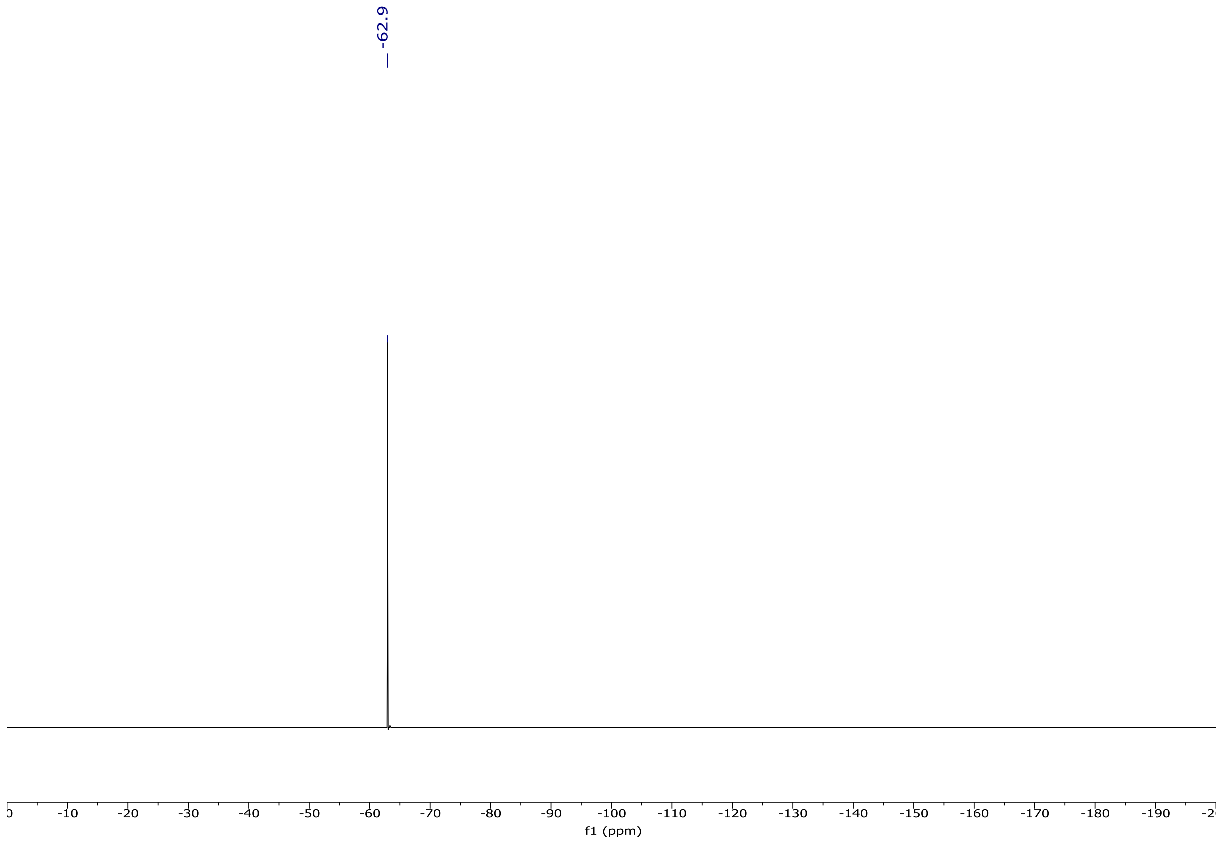
-62.9
f1 (ppm)

2-Benzamido-1-{4-(*tert*-butoxy)phenyl}ethyl 3,5-bis(trifluoromethyl)benzoate (3c)

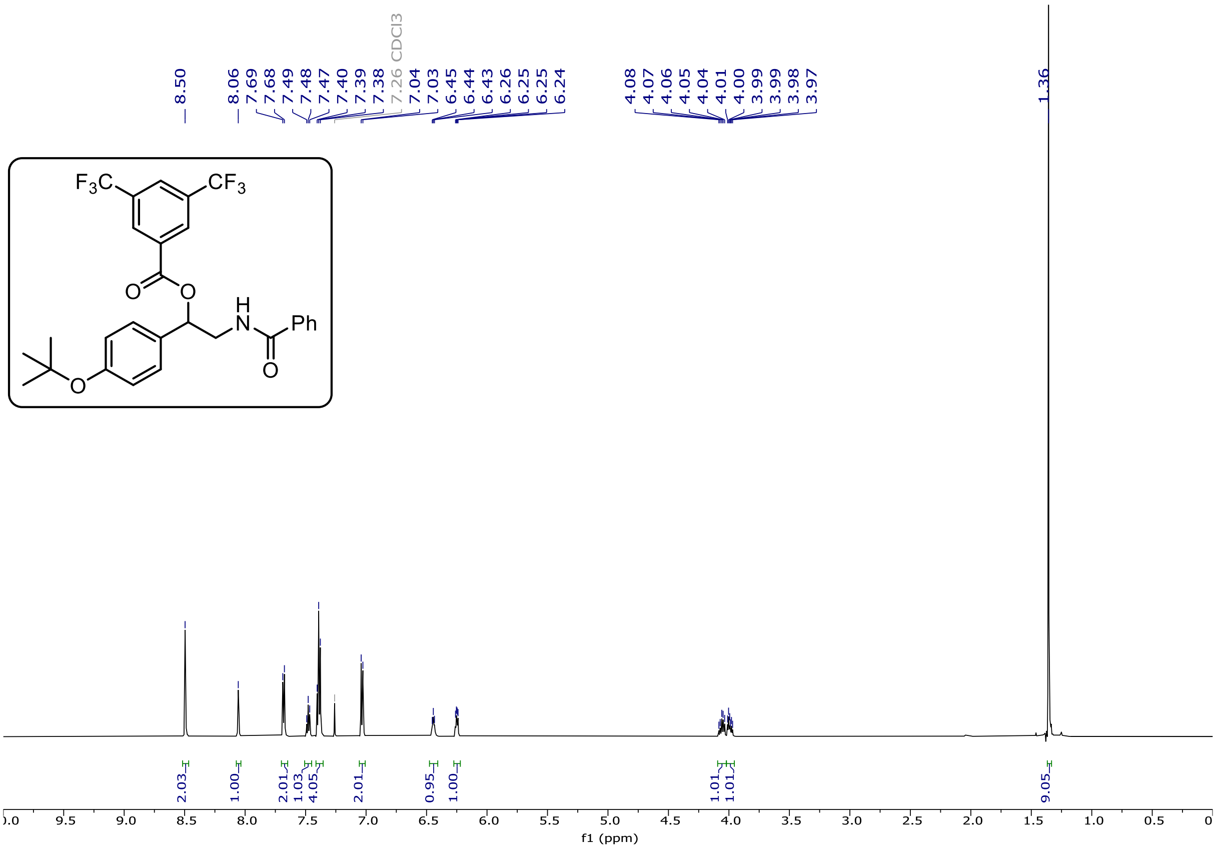

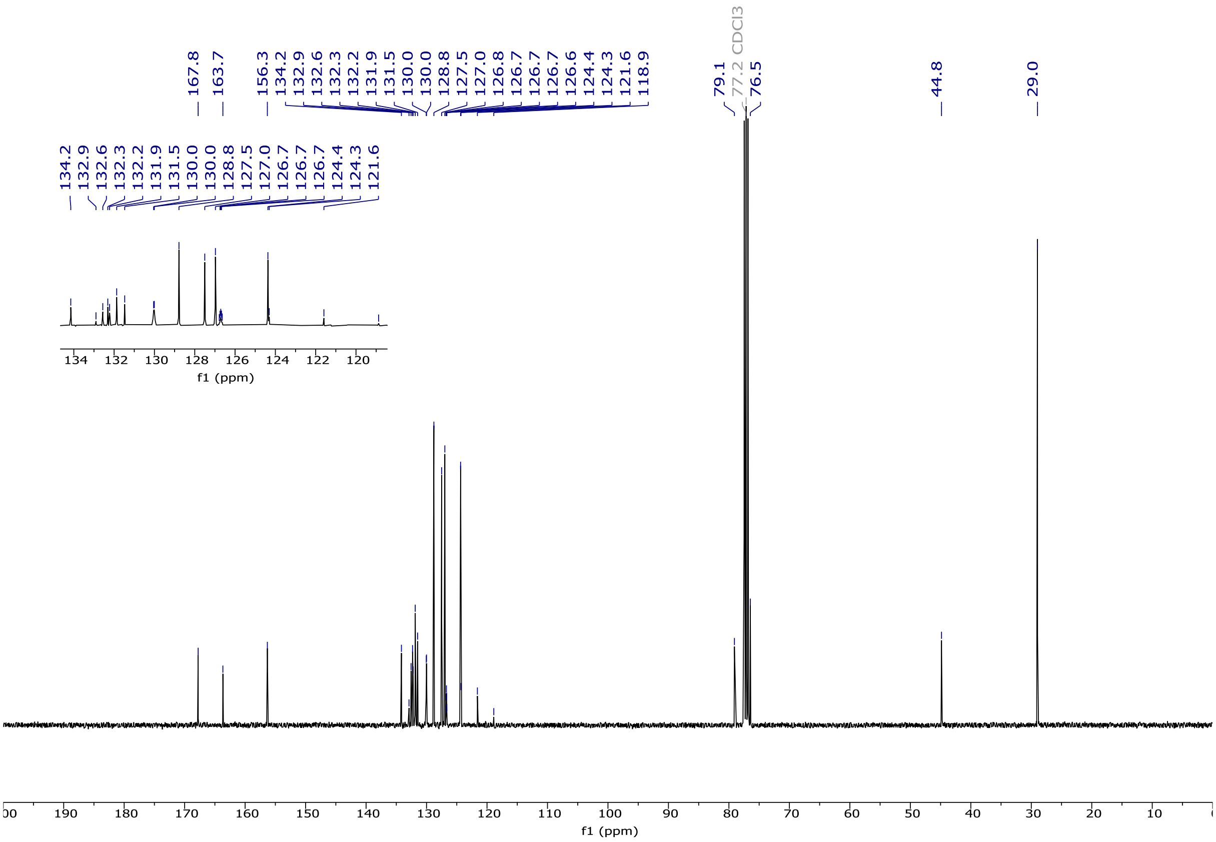

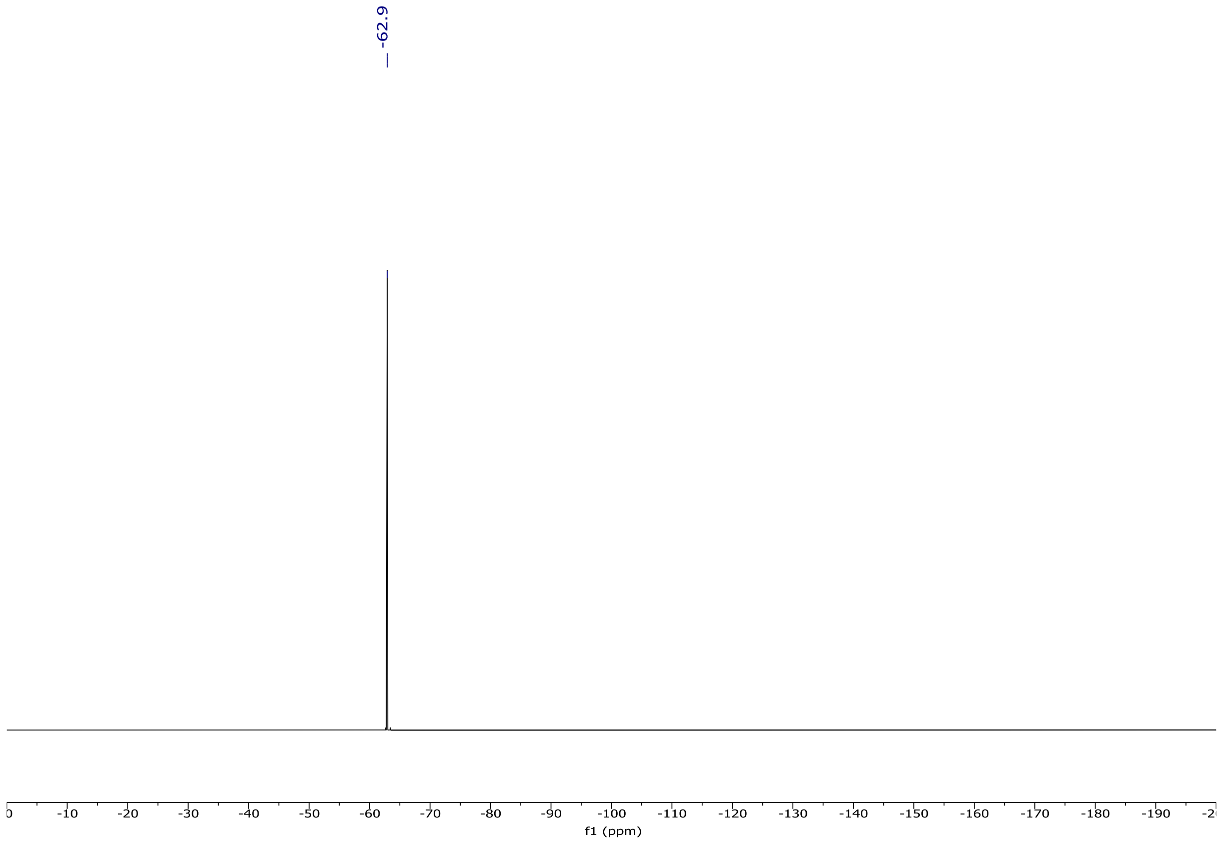
-62.9
0
-10
-20
-30
-40
-50
-60
-70
-80
-90
-100
-110
-120
-130
-140
-150
-160
-170
-180
-190
f1 (ppm)

2-Benzamido-1-(4-phenoxyphenyl)ethyl 3,5-bis(trifluoromethyl)benzoate (3d)

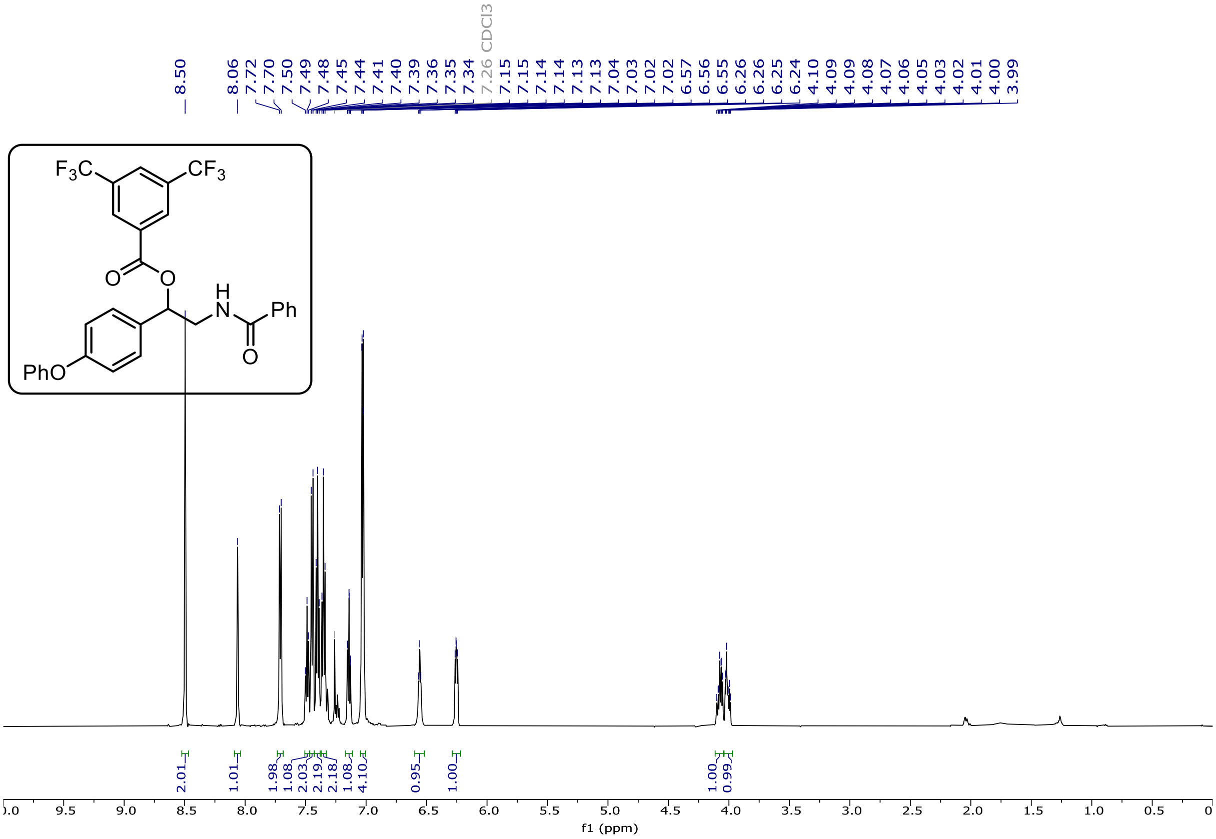

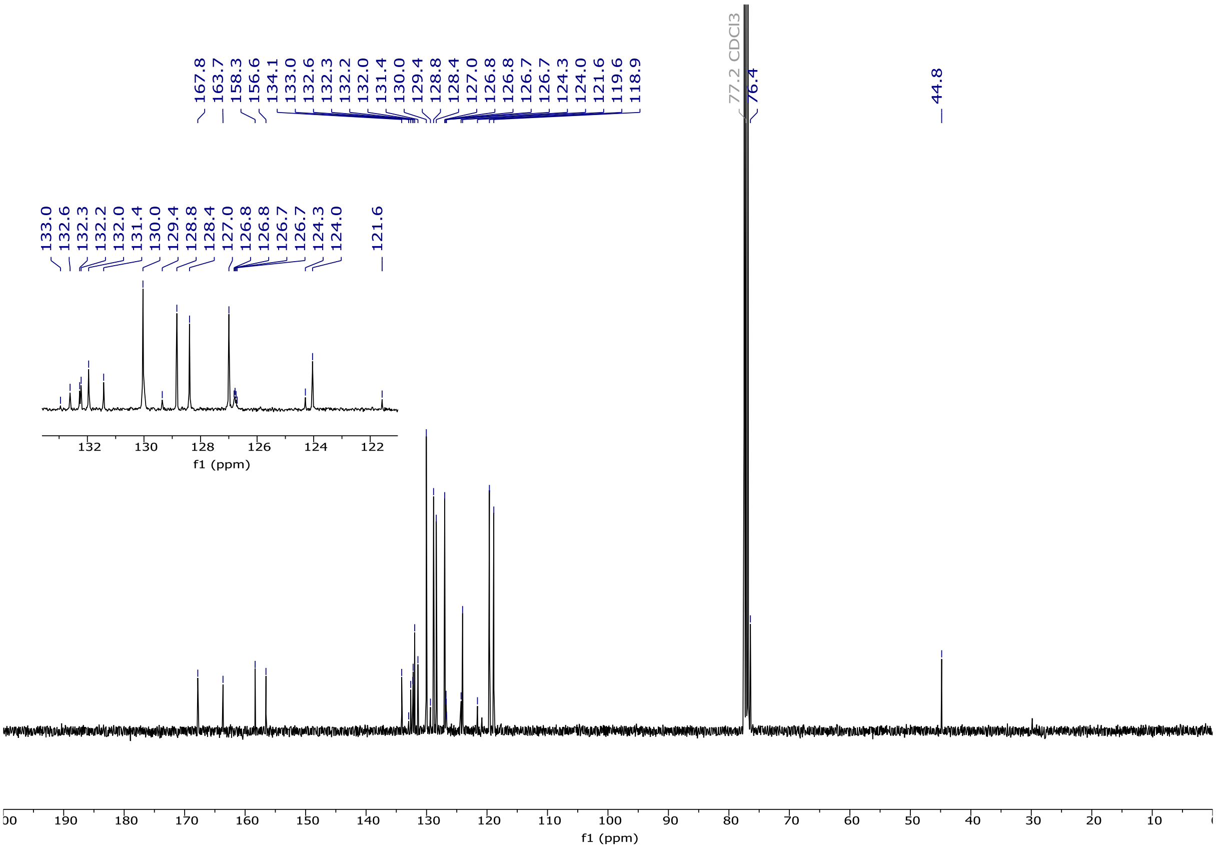

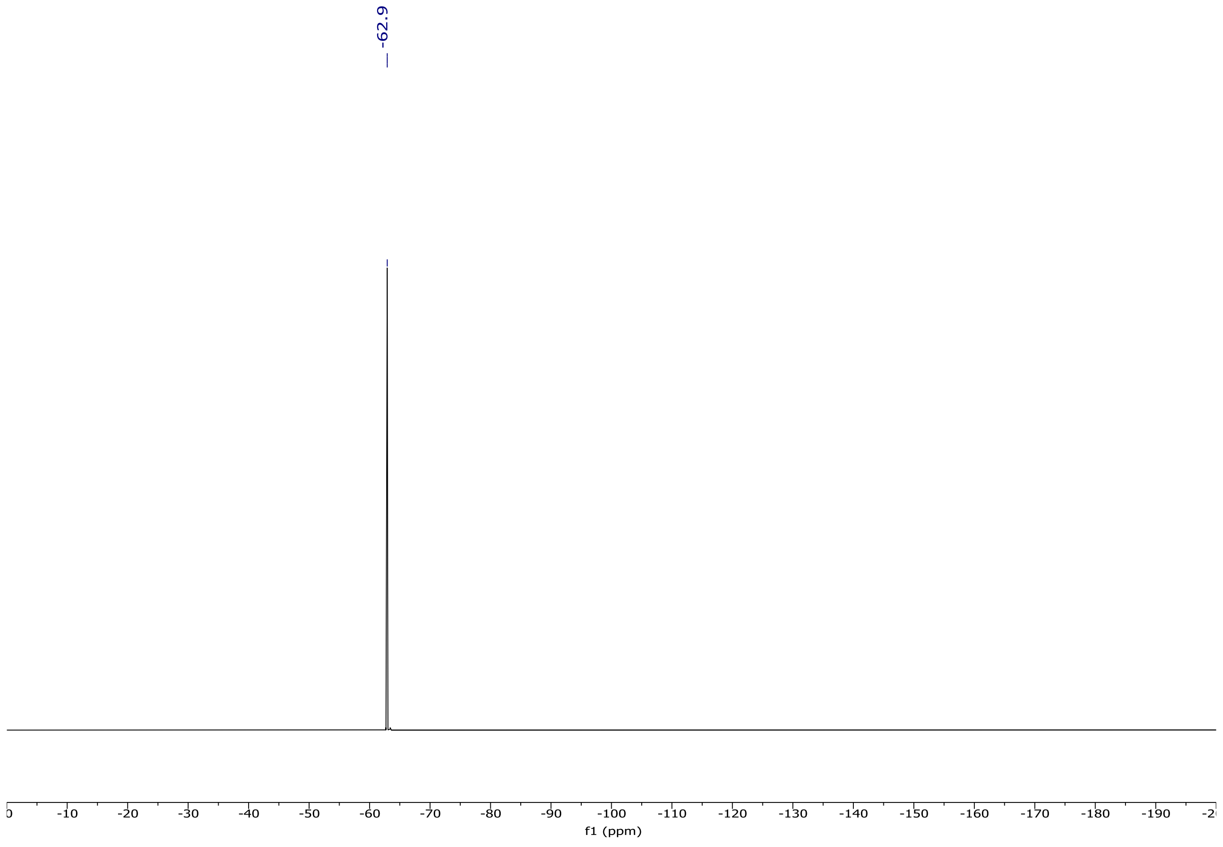
-62.9
0
-10
-20
-30
-40
-50
-60
-70
-80
-90
-100
-110
-120
-130
-140
-150
-160
-170
-180
-190
f1 (ppm)

2-Benzamido-1-(2-methoxyphenyl)ethyl 3,5-bis(trifluoromethyl)benzoate (3e)

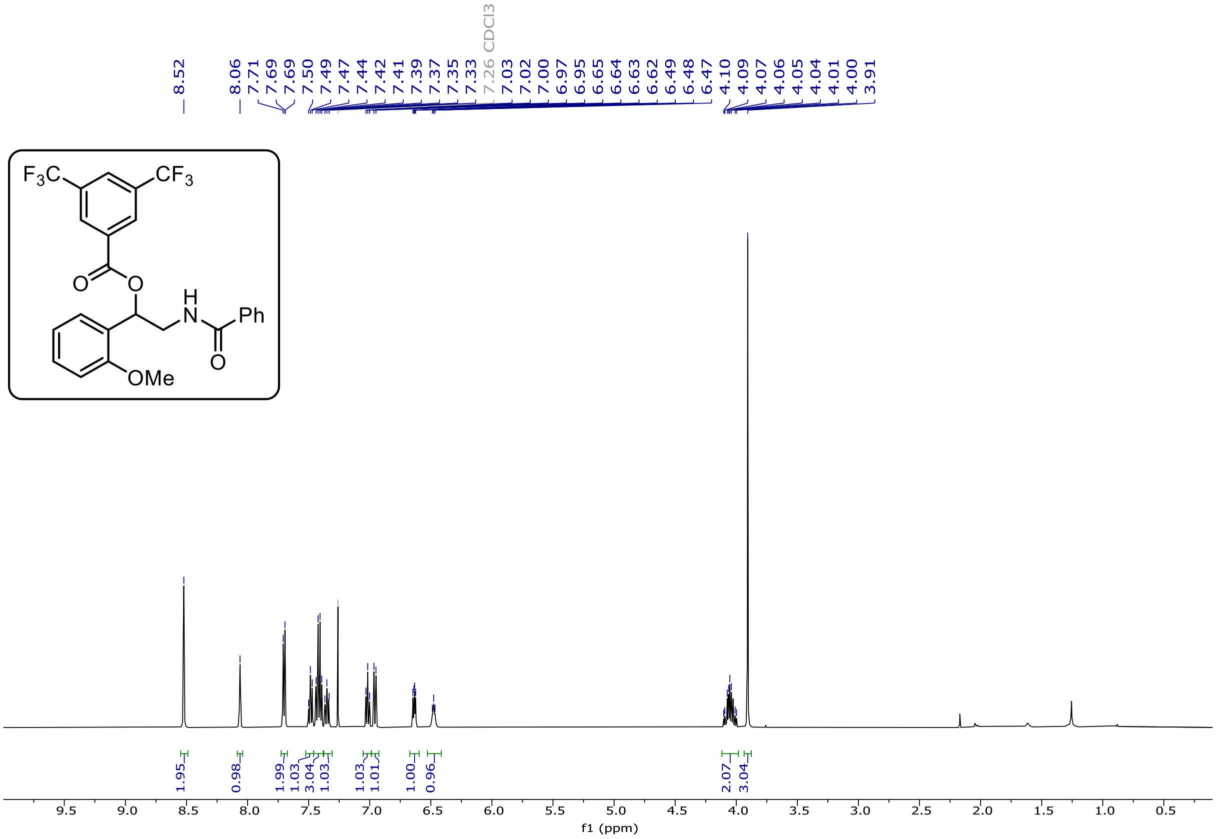

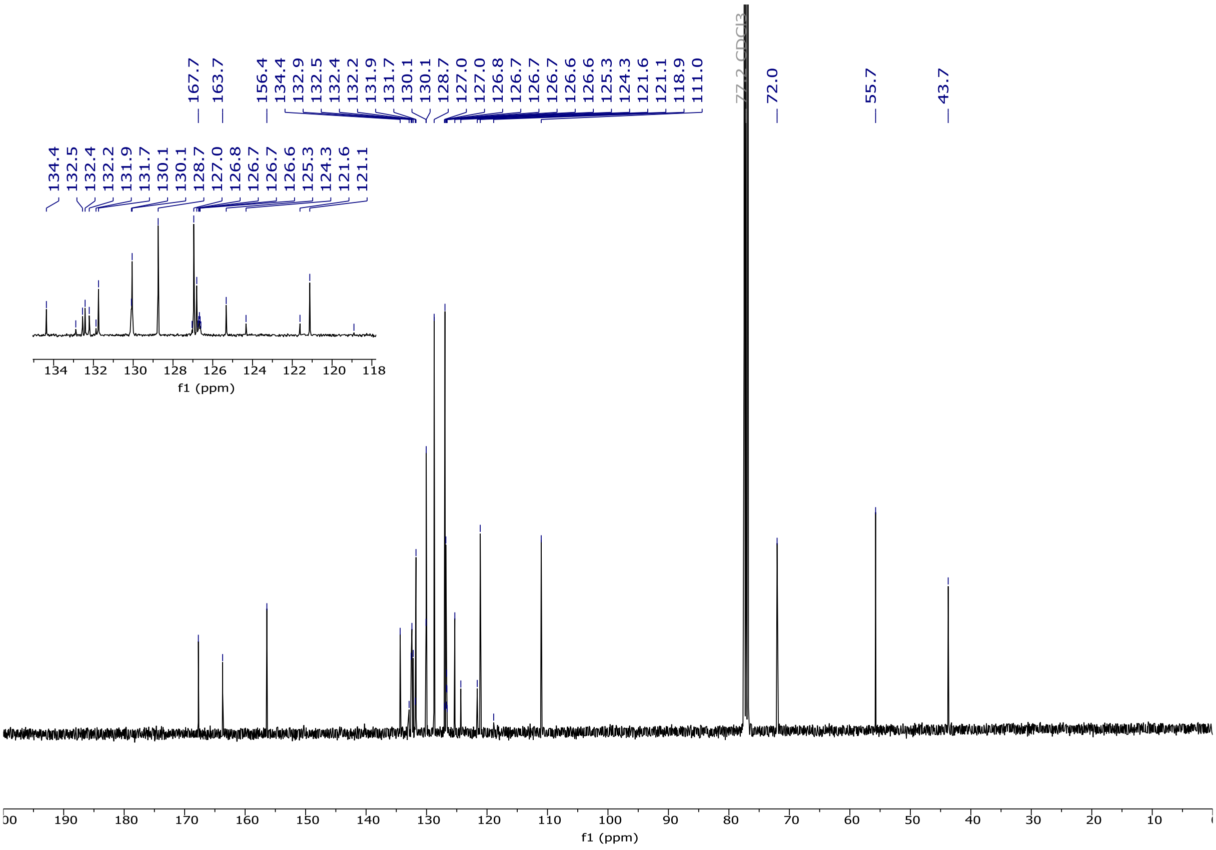

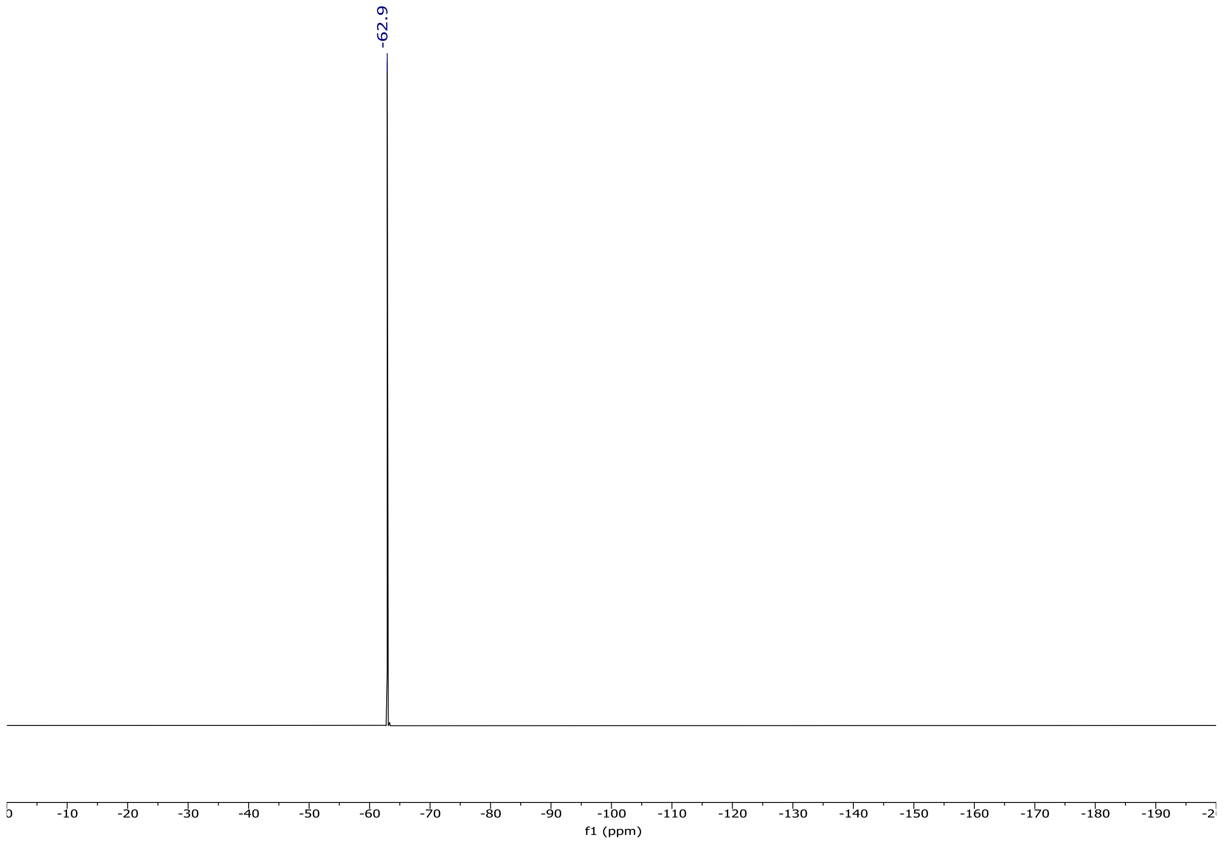
-62.9
f1 (ppm)

2-Benzamido-1-(3,4-dimethoxyphenyl)ethyl 3,5-bis(trifluoromethyl)benzoate (3f)

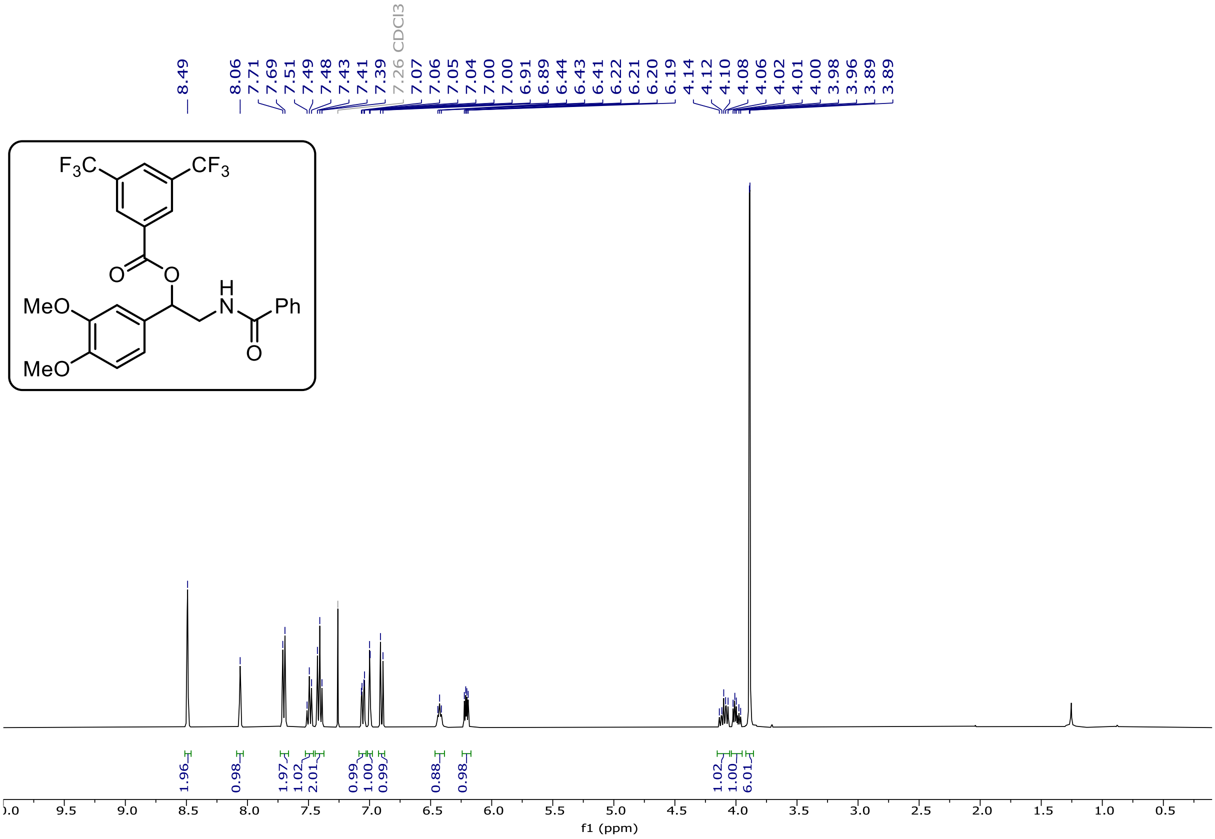

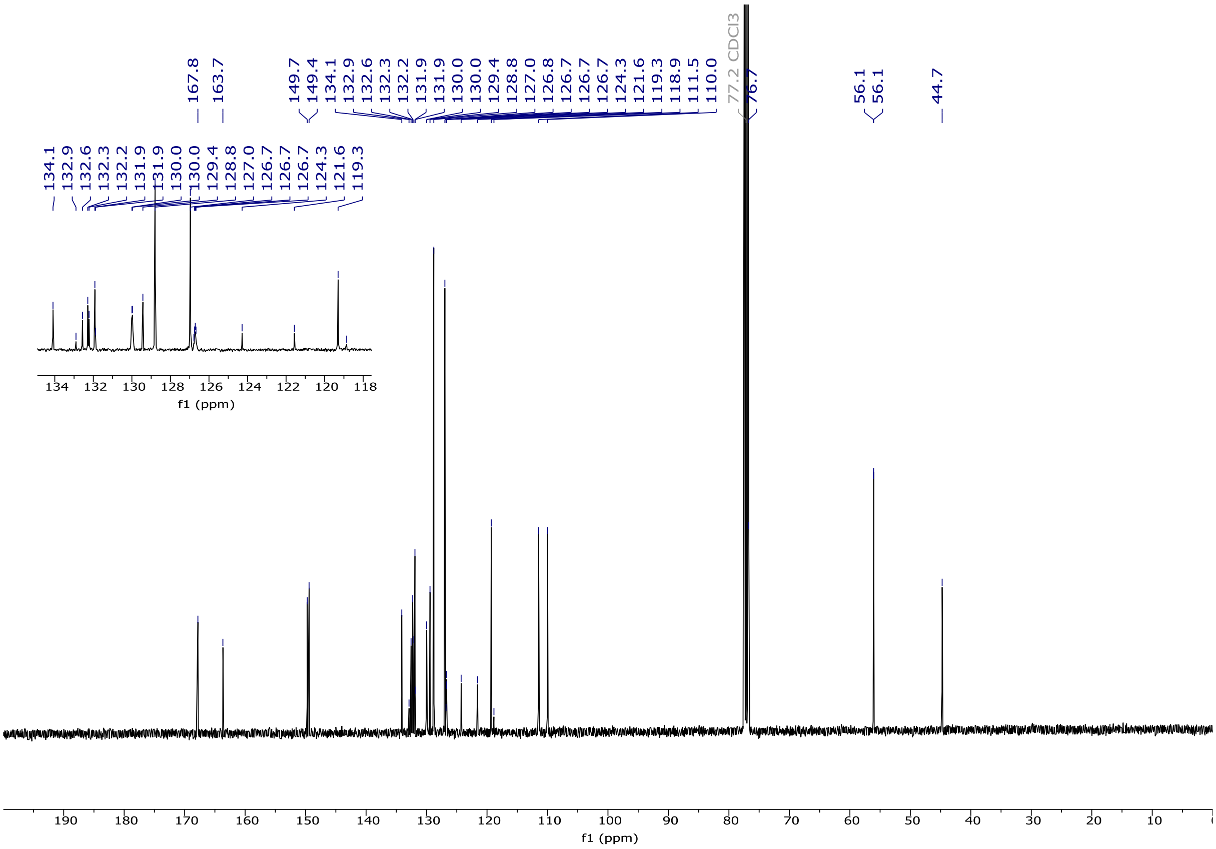

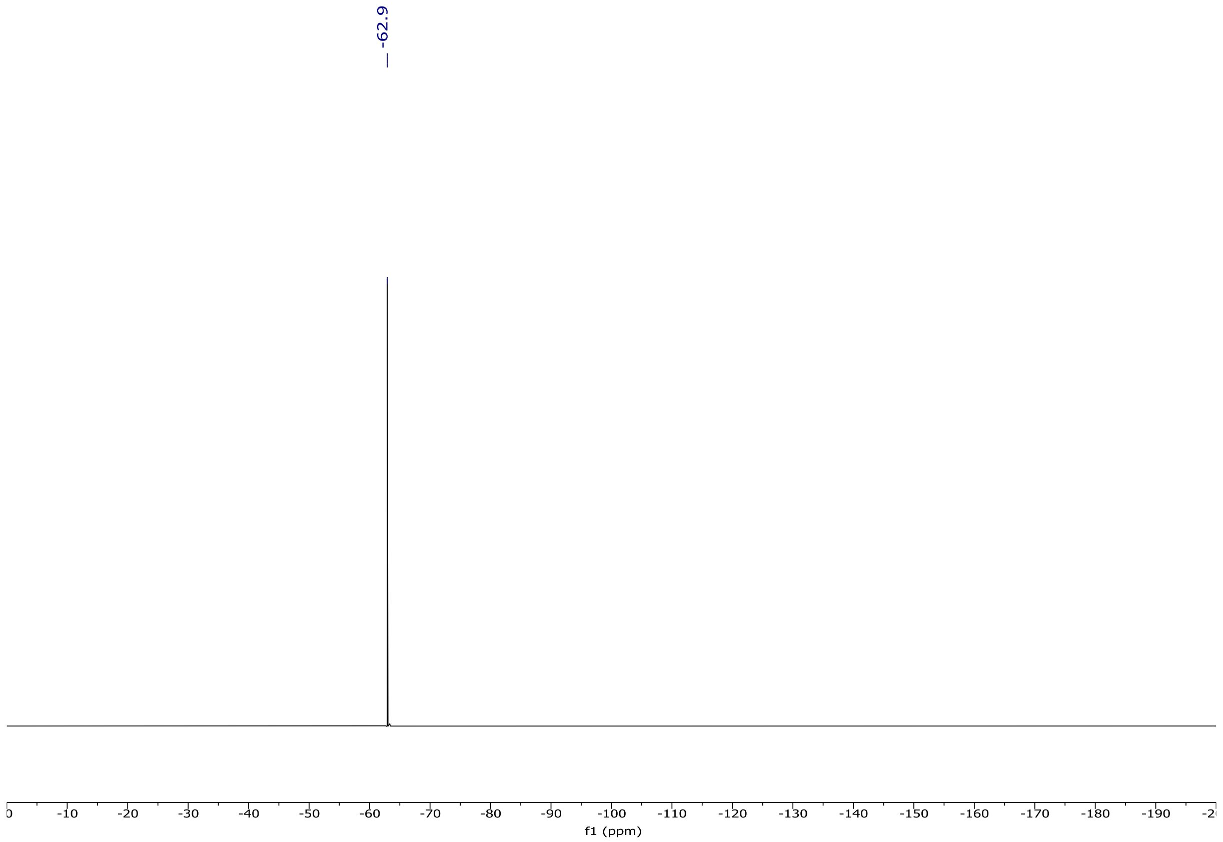
-62.9
f1 (ppm)

2-Benzamido-1-(benzo[*d*][1,3]dioxol-5-yl)ethyl 3,5-bis(trifluoromethyl)benzoate (3g)

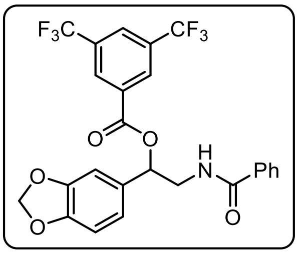

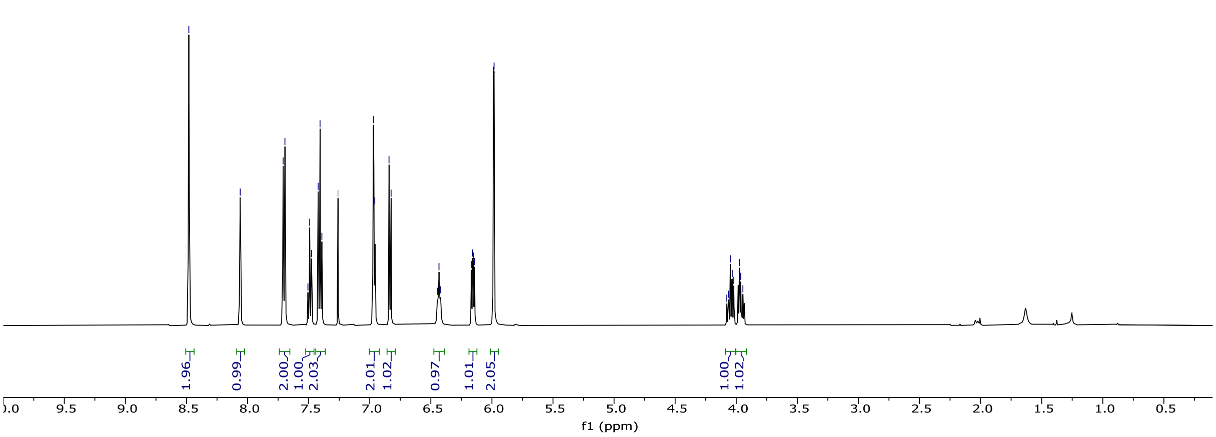

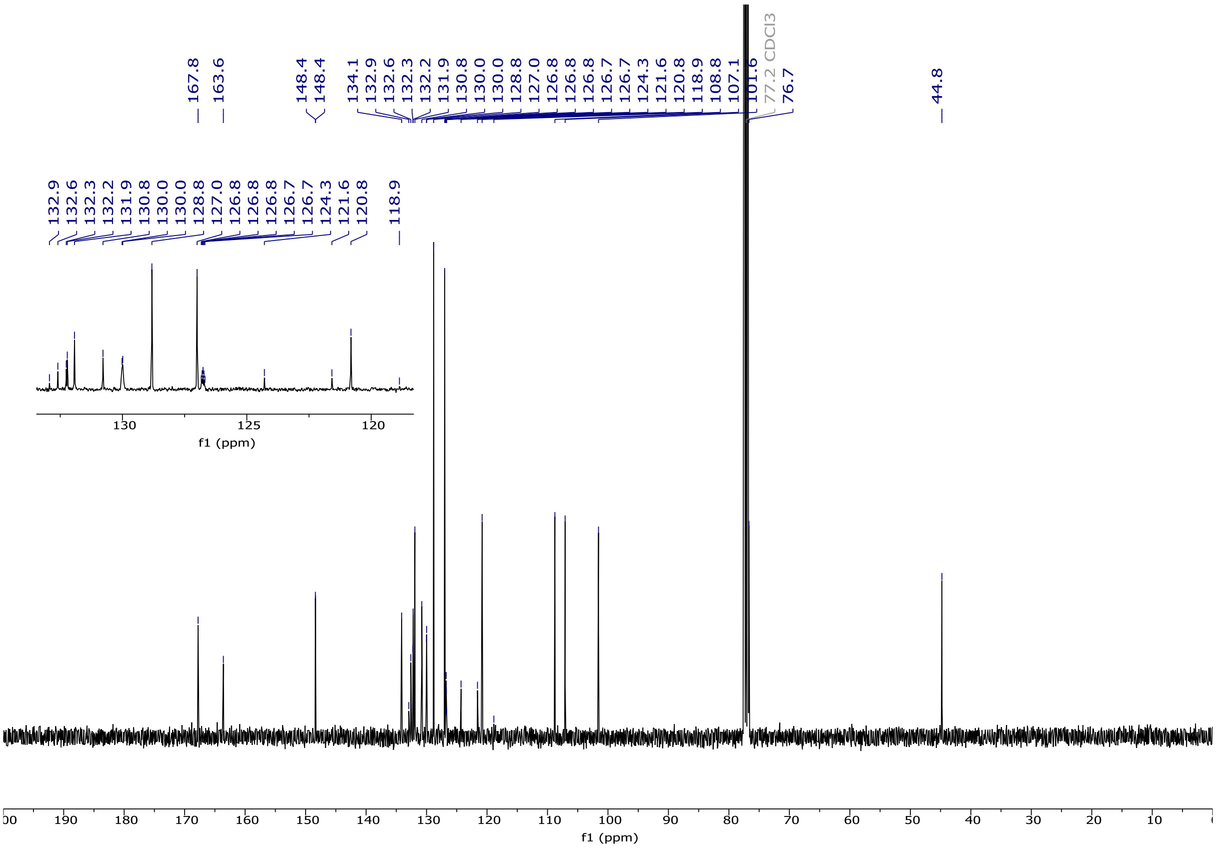

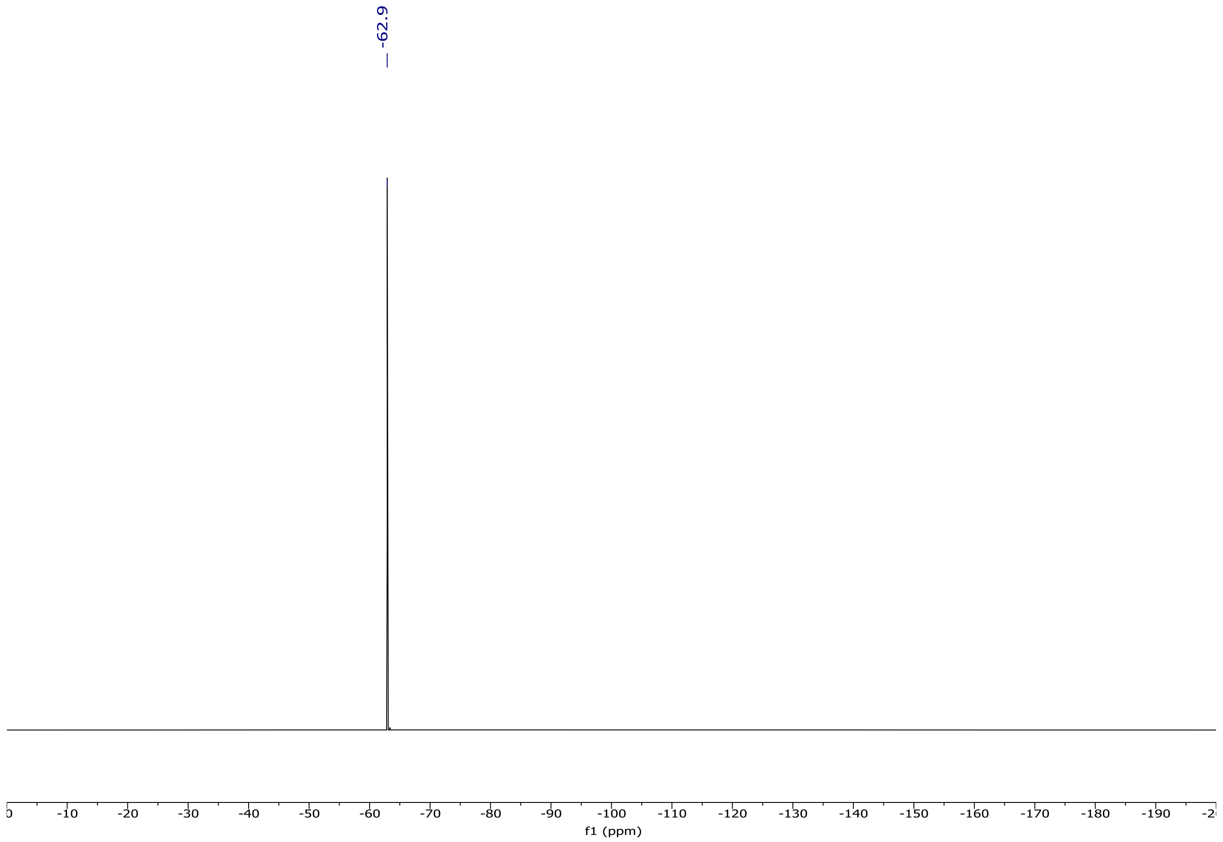
-62.9
0
-10
-20
-30
-40
-50
-60
-70
-80
-90
-100
-110
-120
-130
-140
-150
-160
-170
-180
-190
f1 (ppm)

2-Benzamido-1-(4-methoxyphenyl)propyl 3,5-bis(trifluoromethyl)benzoate (3h + 3h')

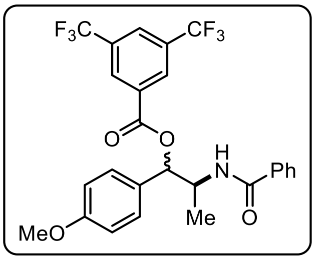

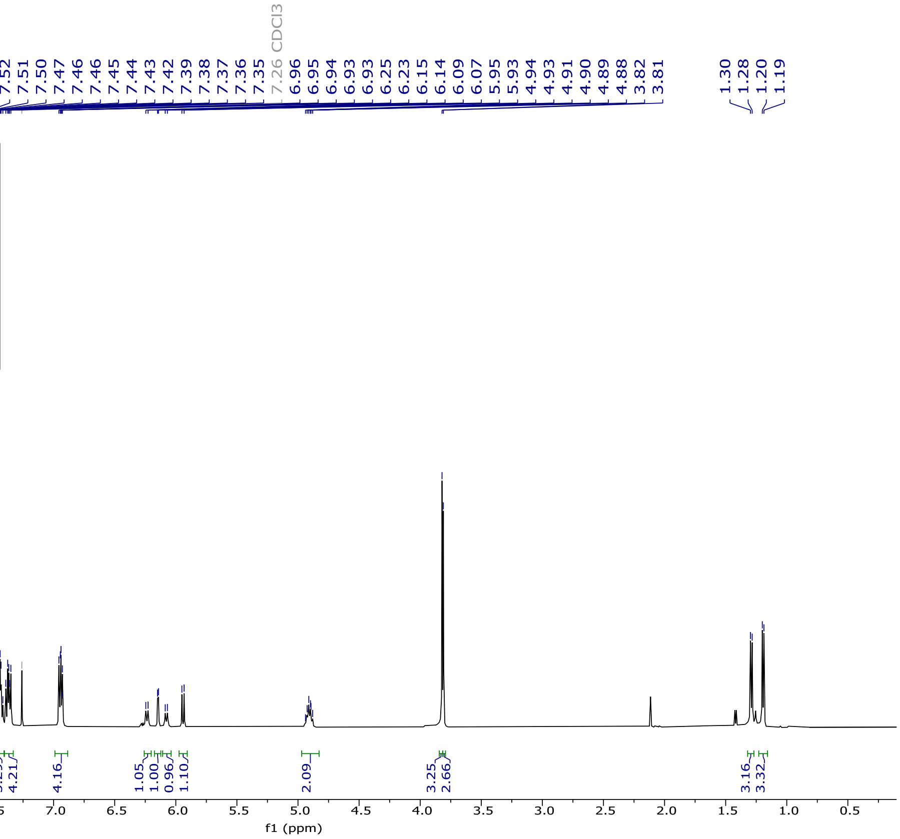

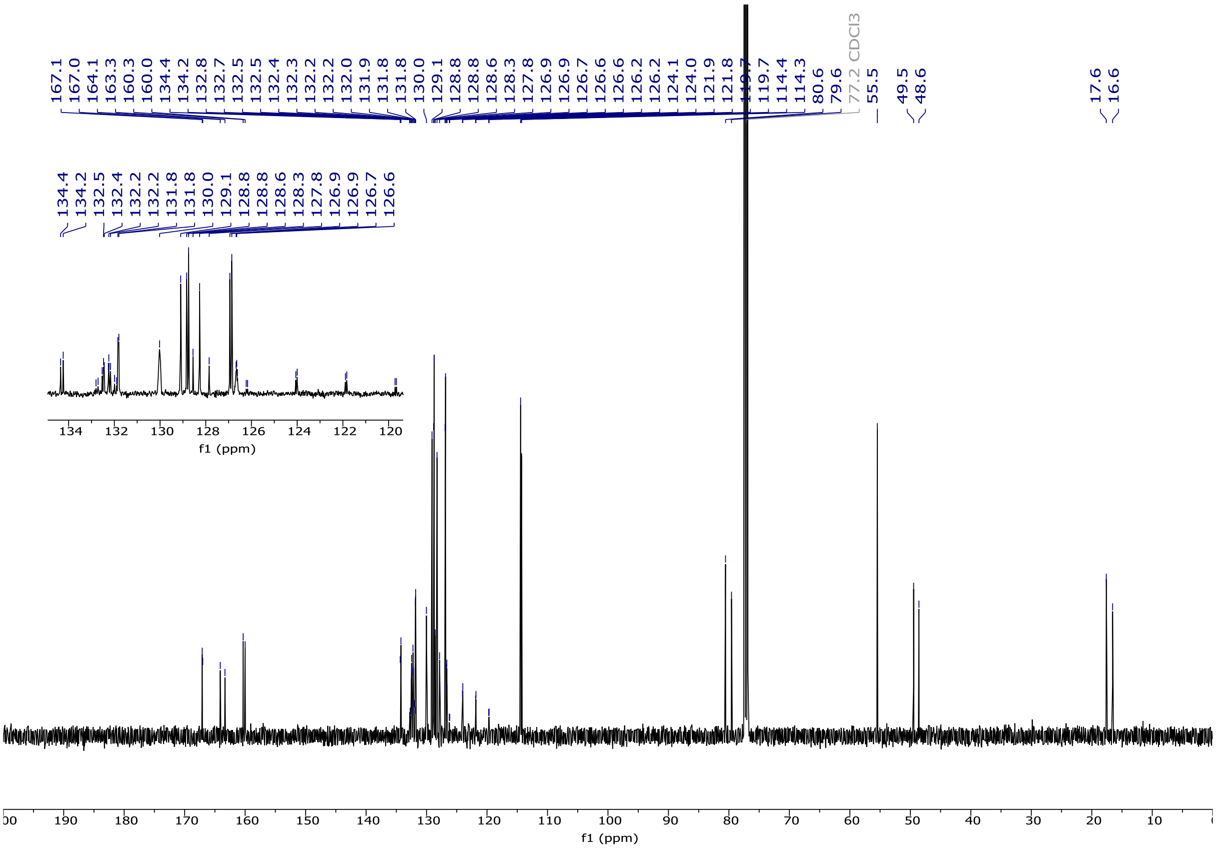

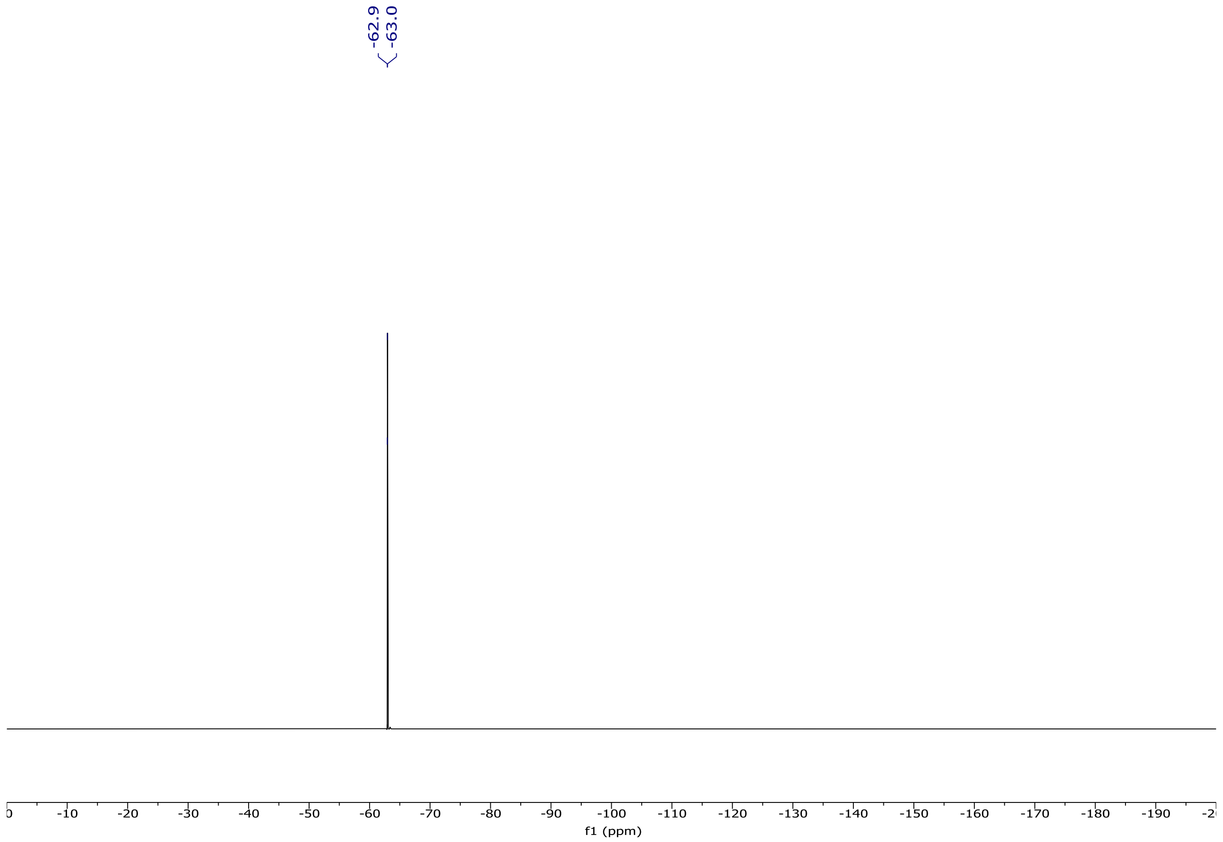
-62.9
-63.0
0
-10
-20
-30
-40
-50
-60
-70
-80
-90
-100
-110
-120
-130
-140
-150
-160
-170
-180
-190
-2
f1 (ppm)

2-Benzamido-1-(phenylthio)ethyl 3,5-bis(trifluoromethyl)benzoate (3i)

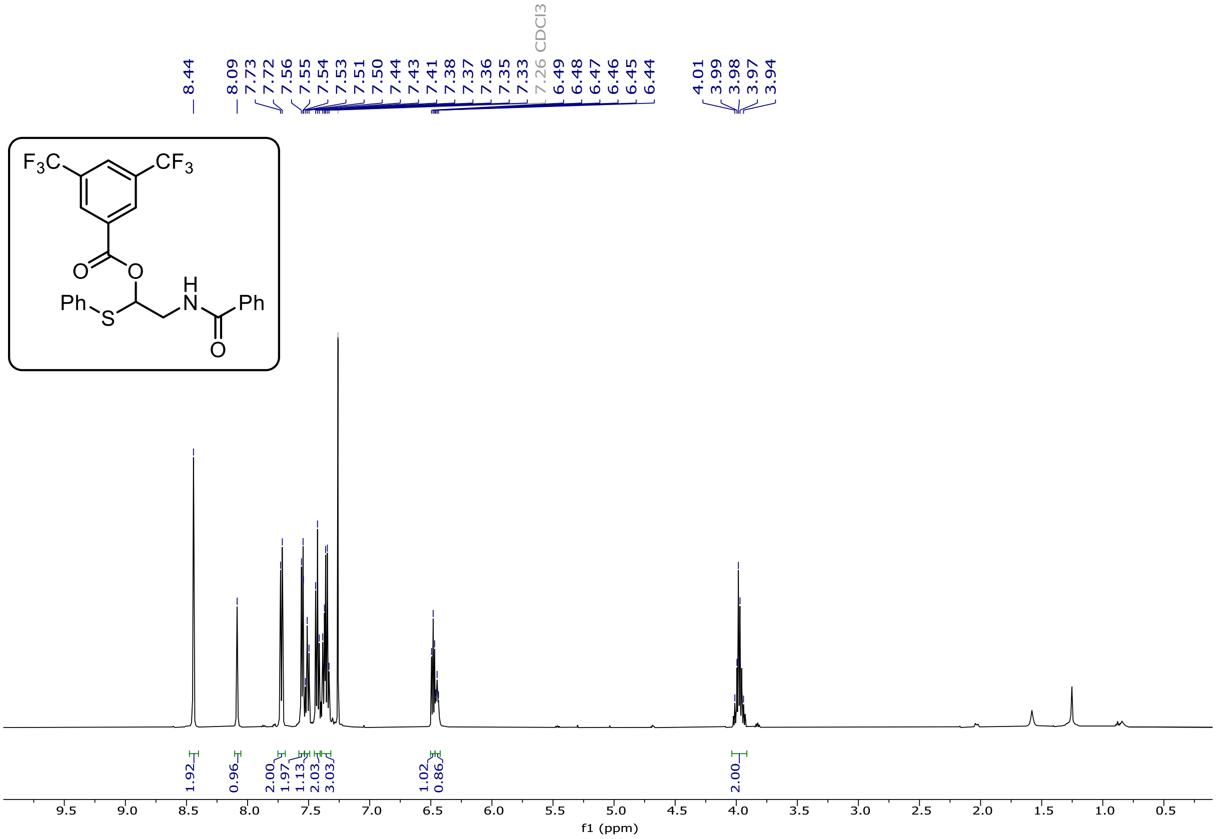

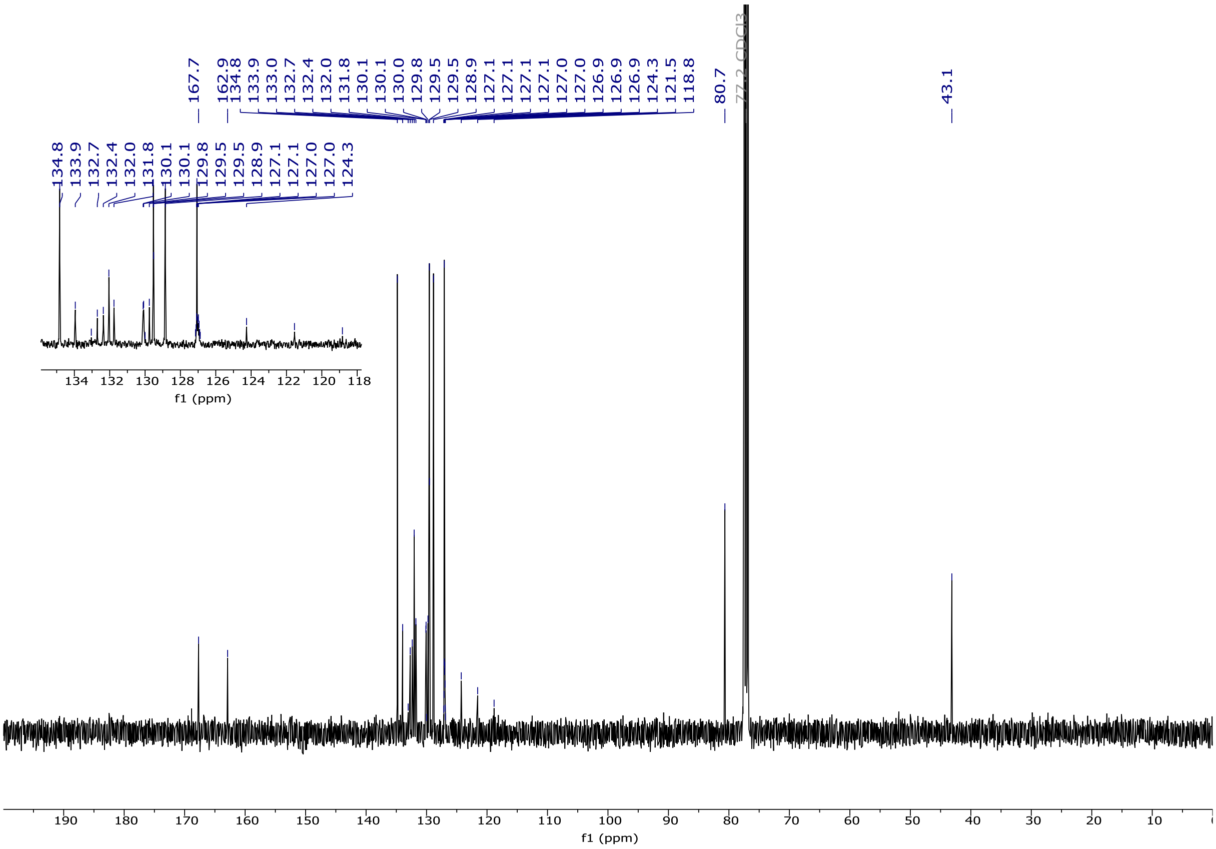

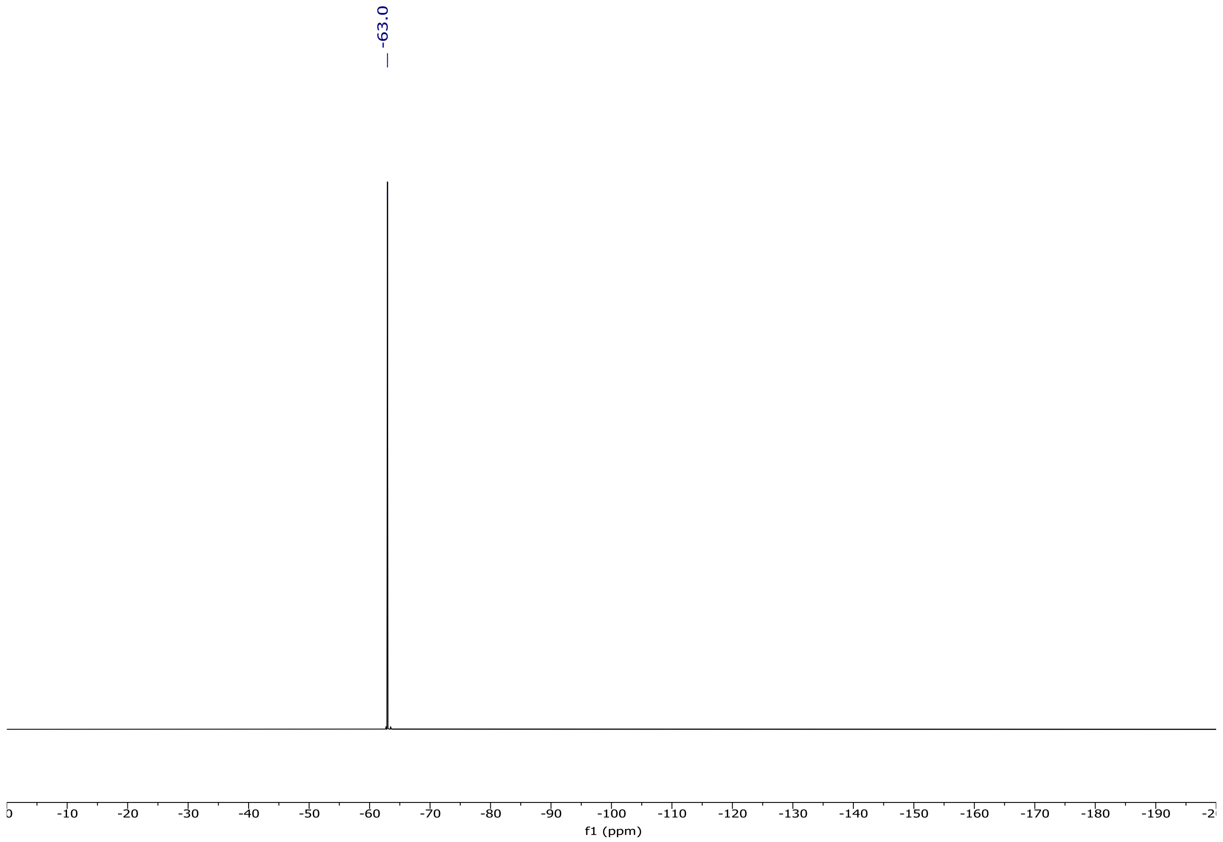

-63.0
0
-10
-20
-30
-40
-50
-60
-70
-80
-90
-100
-110
-120
-130
-140
-150
-160
-170
-180
-190
f1 (ppm)

1-(4-Methoxyphenyl)-2-(4-methylbenzamido)ethyl 3,5-bis(trifluoromethyl)benzoate (3j)

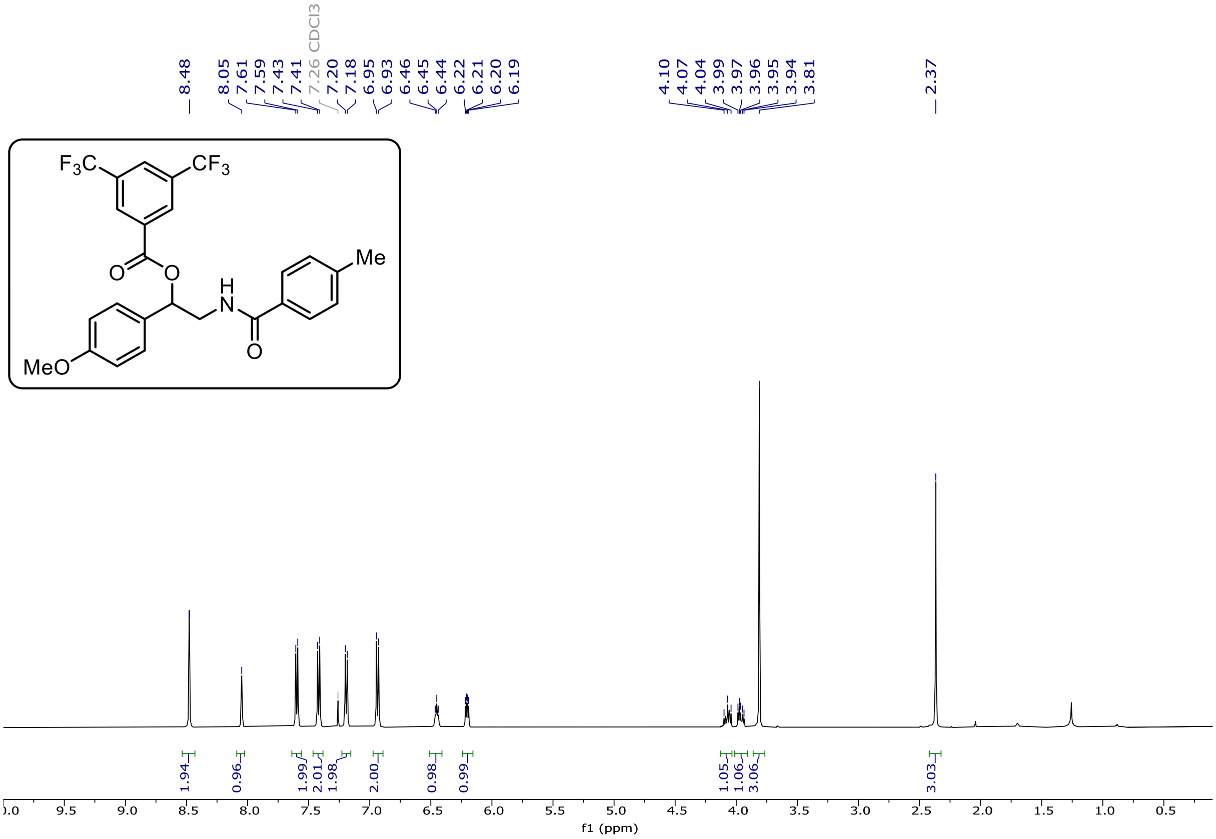

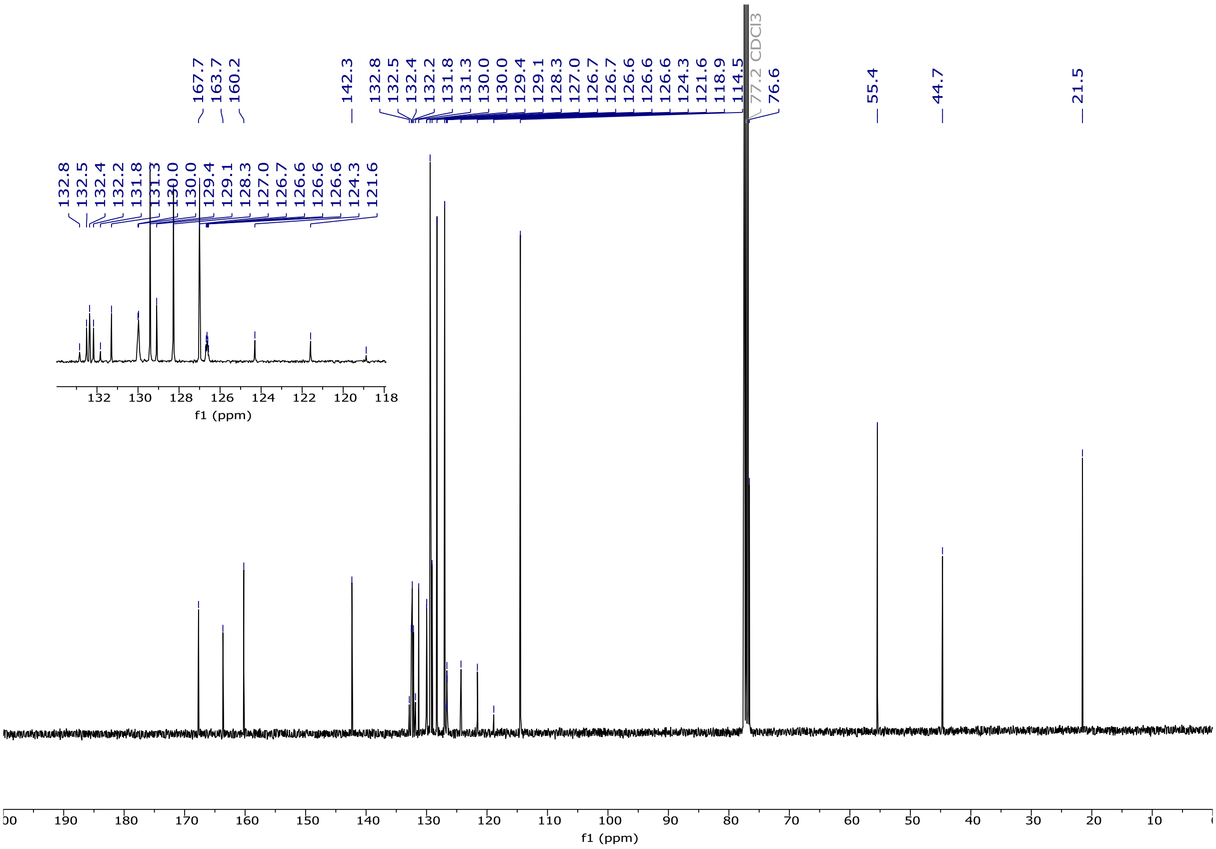

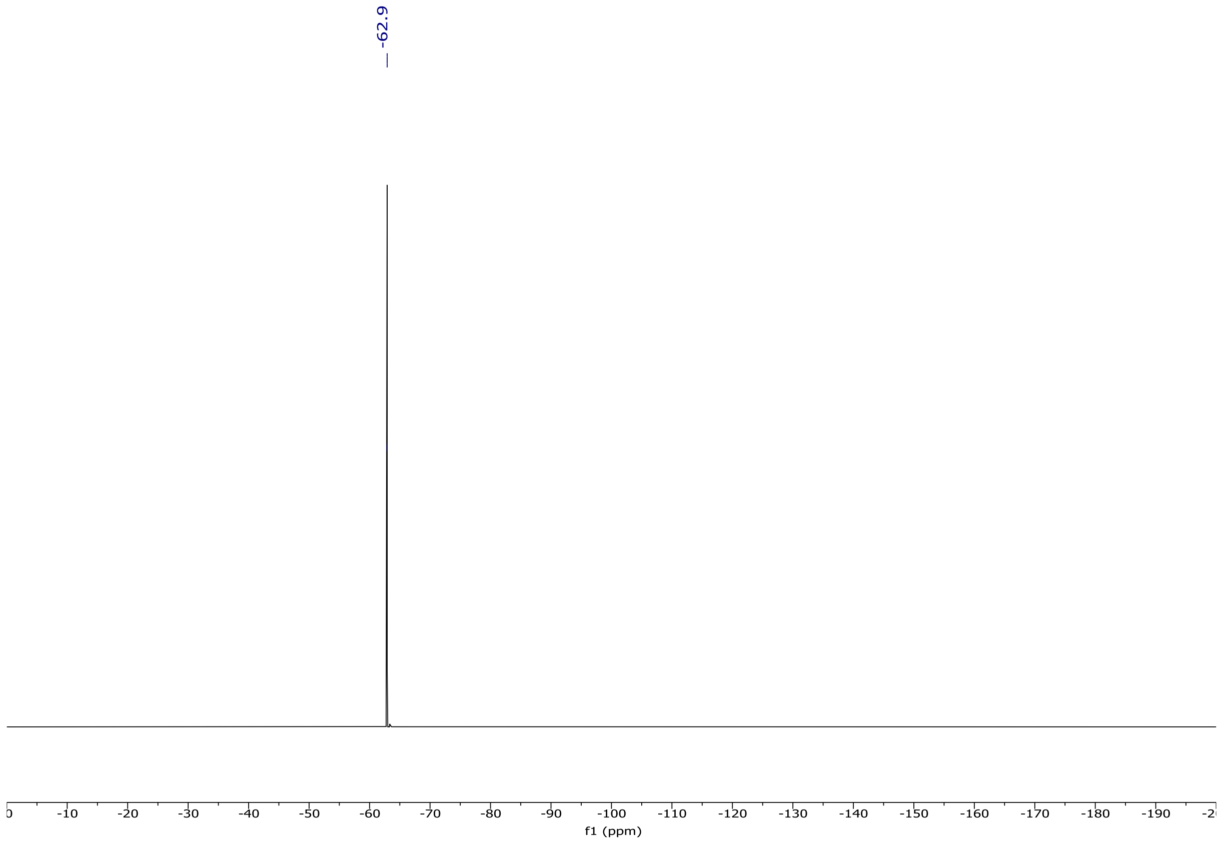
-62.9
-10
-20
-30
-40
-50
-60
-70
-80
-90
-100
-110
-120
-130
-140
-150
-160
-170
-180
-190
f1 (ppm)

2-{4-(*tert*-Butyl)benzamido}-1-(4-methoxyphenyl)ethyl 3,5-bis(trifluoromethyl)benzoate (3k)

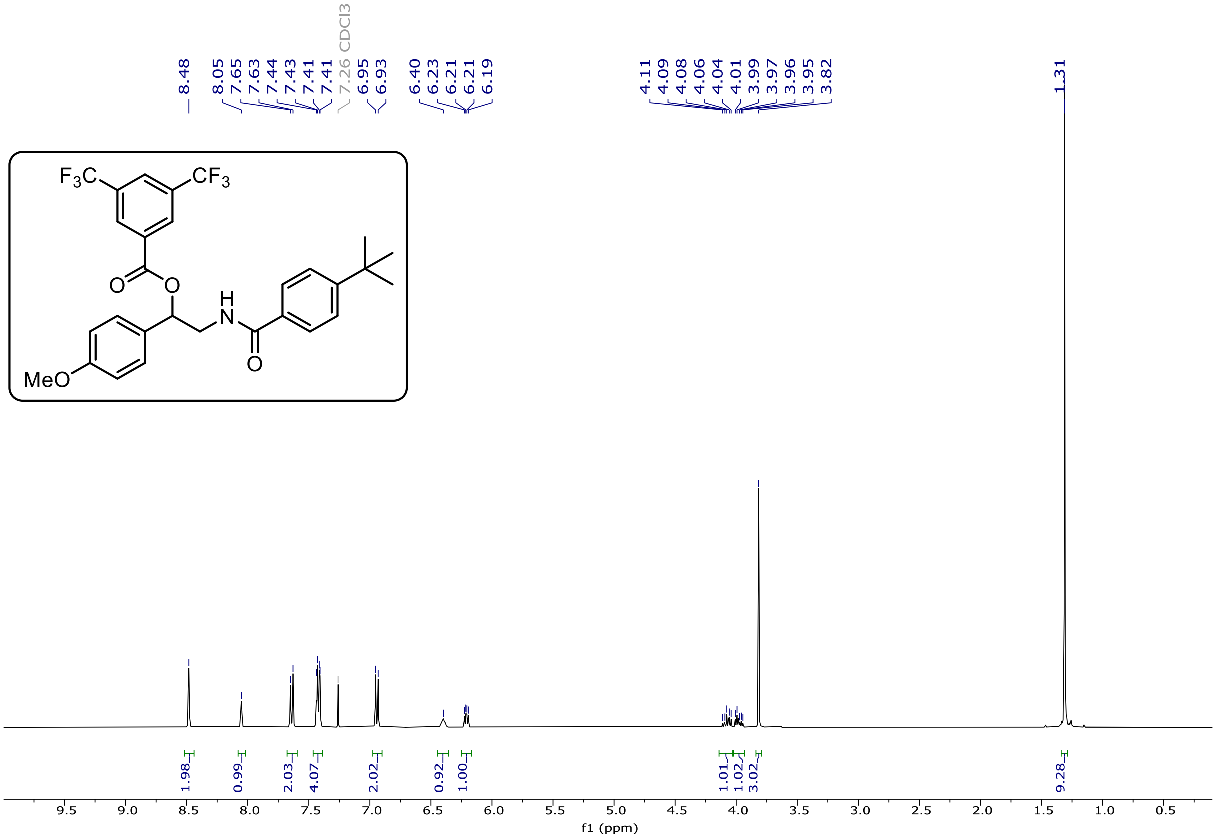

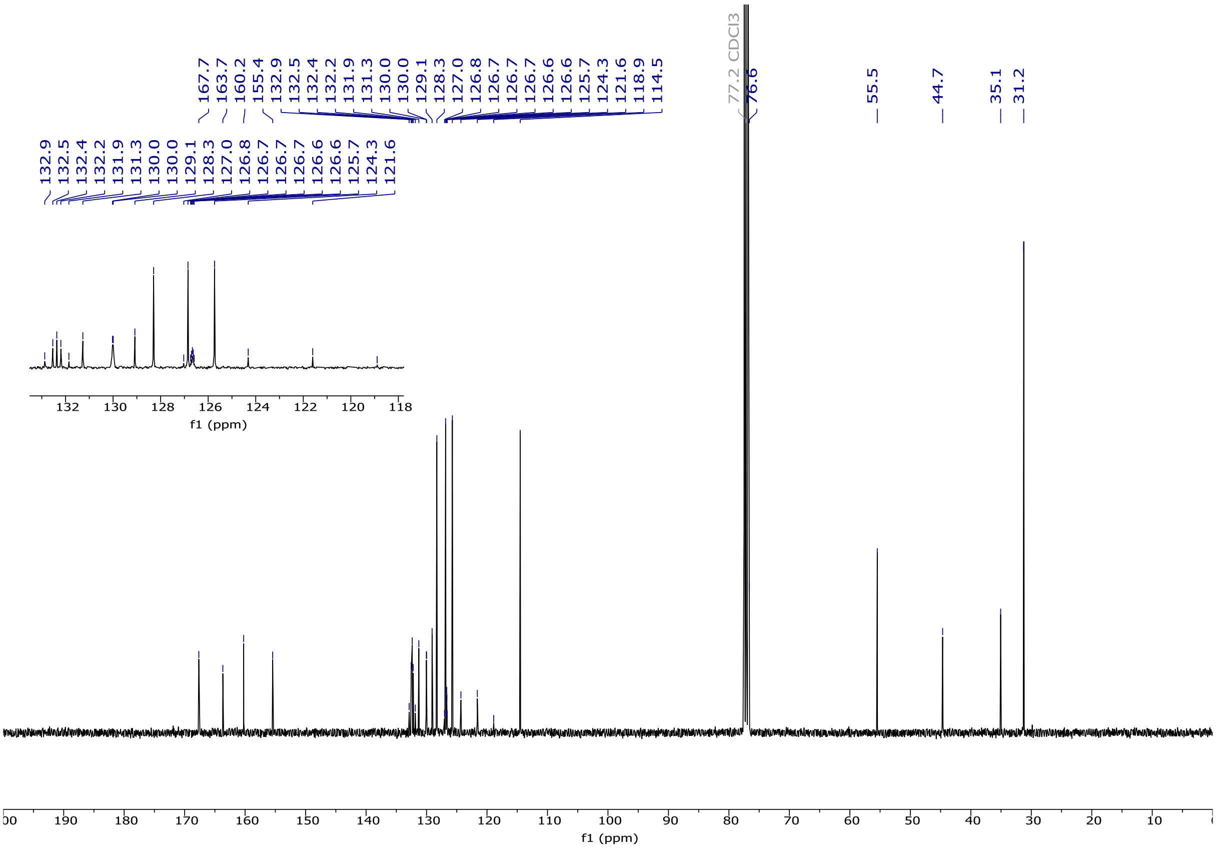

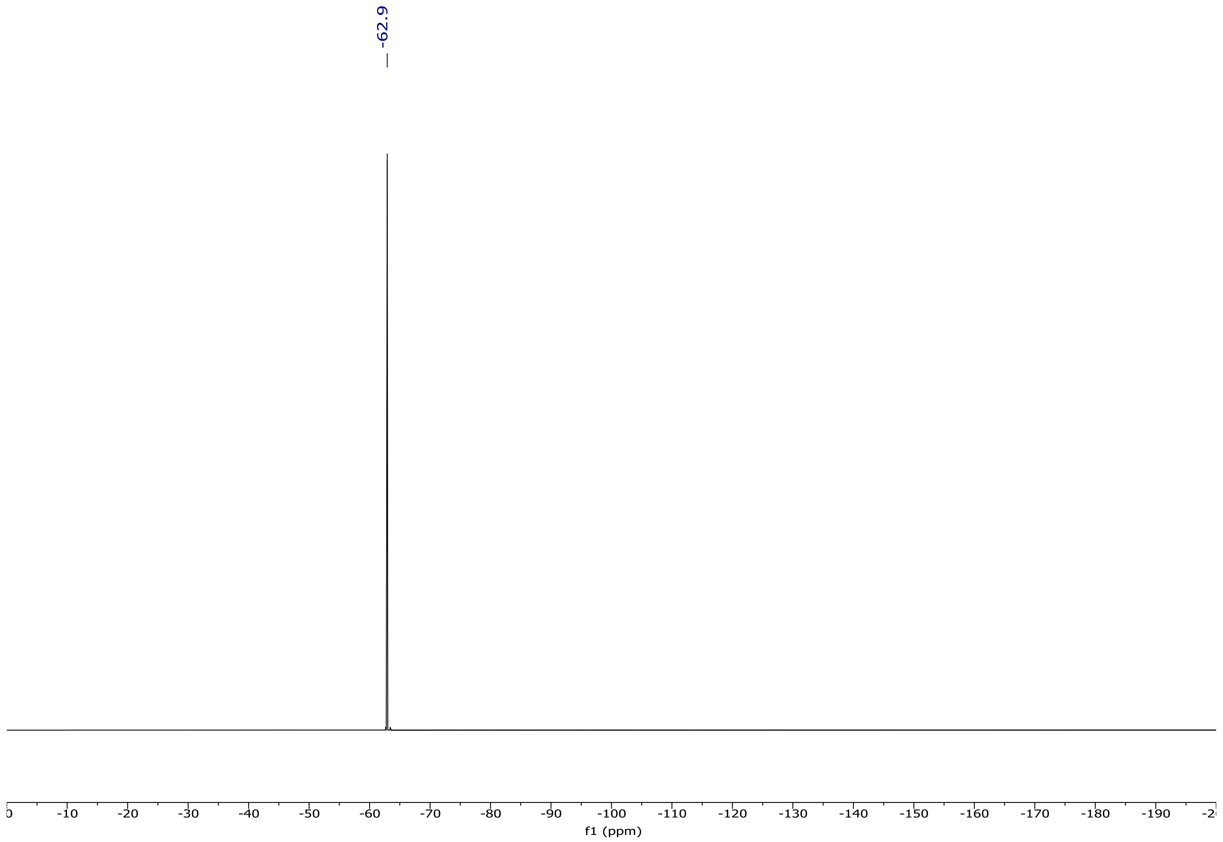

-62.9
0
-10
-20
-30
-40
-50
-60
-70
-80
-90
-100
-110
-120
-130
-140
-150
-160
-170
-180
-190
f1 (ppm)

2-(4-Chlorobenzamido)-1-(4-methoxyphenyl)ethyl 3,5-bis(trifluoromethyl)benzoate (3l)

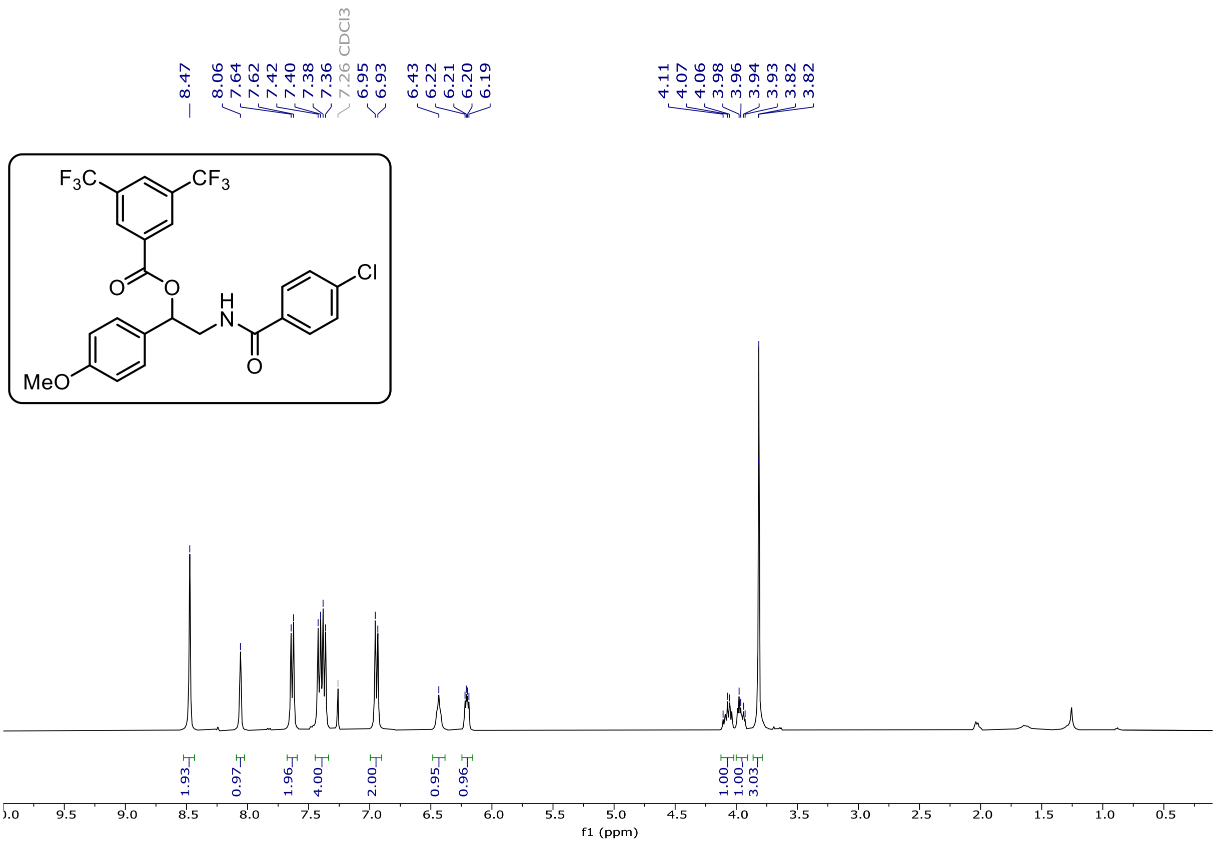

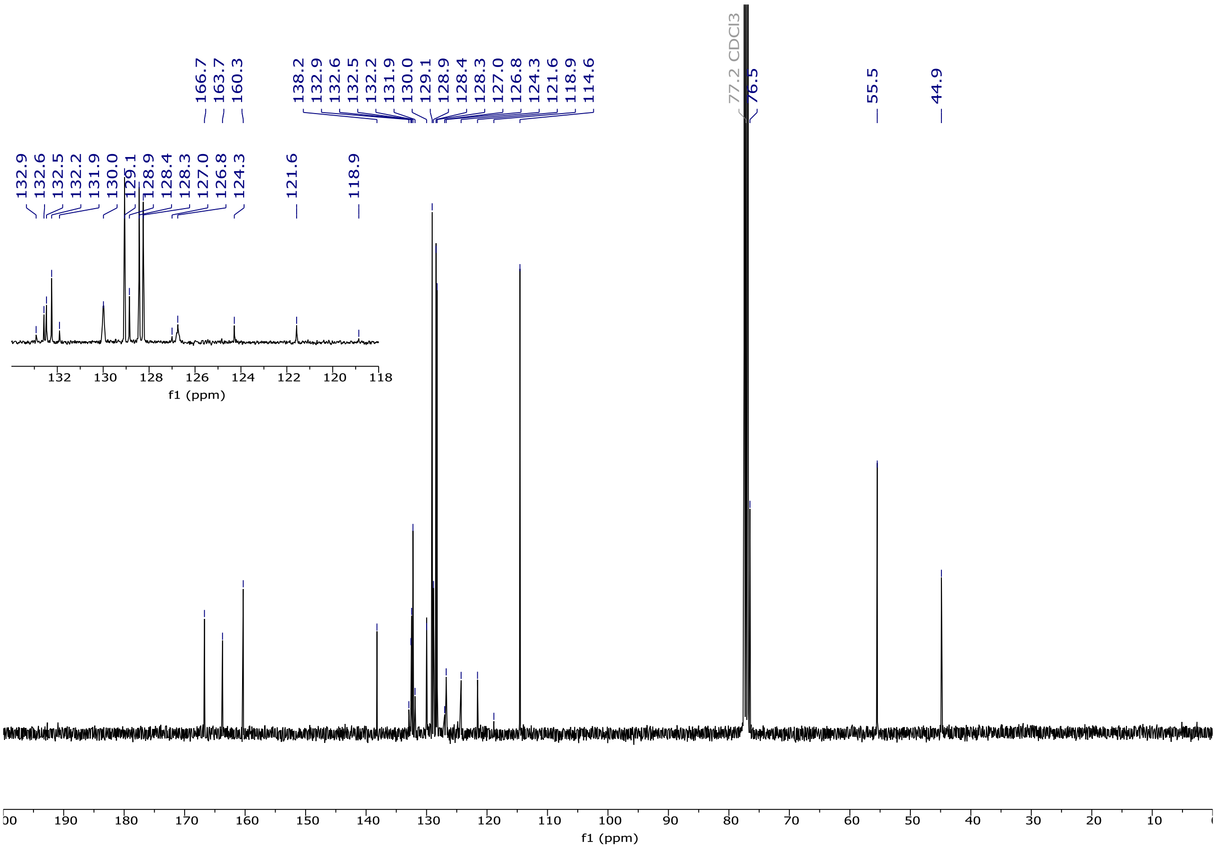

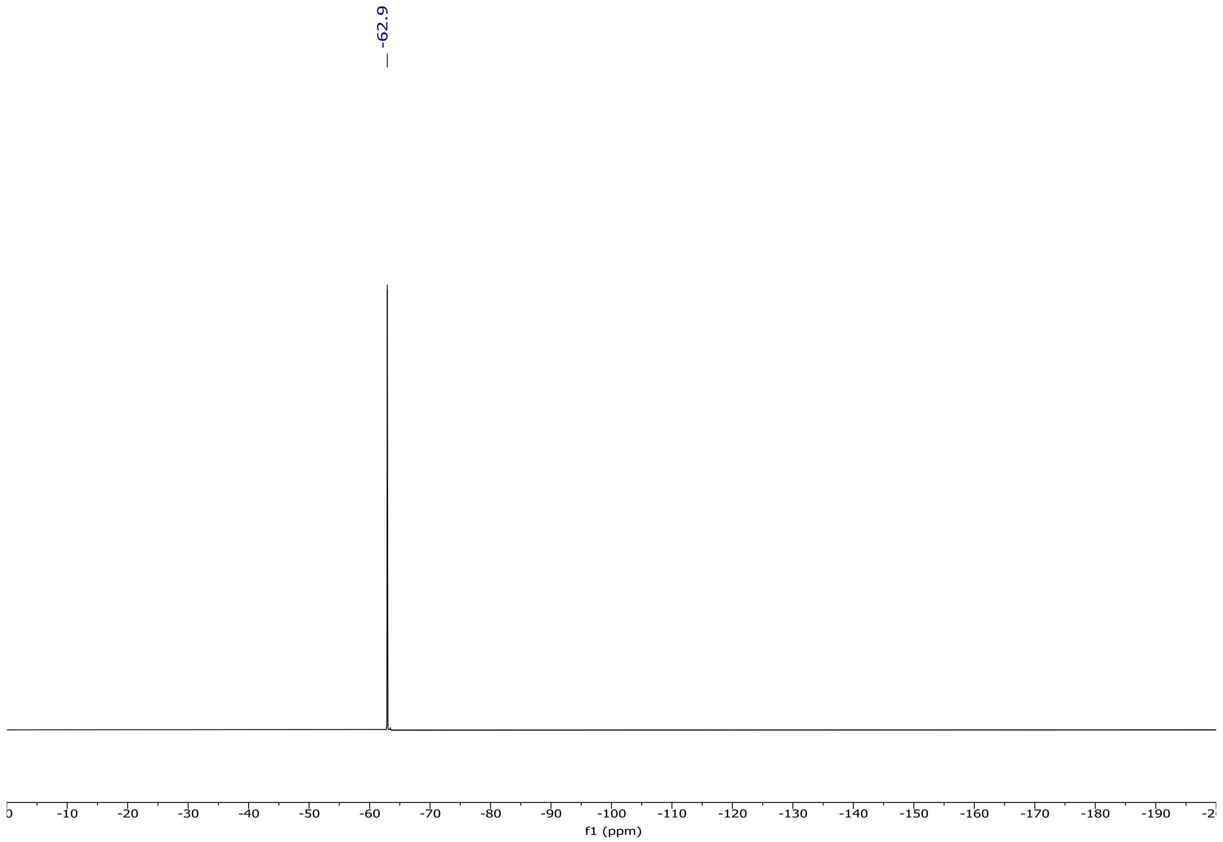
-62.9
0
-10
-20
-30
-40
-50
-60
-70
-80
-90
-100
-110
-120
-130
-140
-150
-160
-170
-180
-190
f1 (ppm)

2-(4-Bromobenzamido)-1-(4-methoxyphenyl)ethyl 3,5-bis(trifluoromethyl)benzoate (3m)

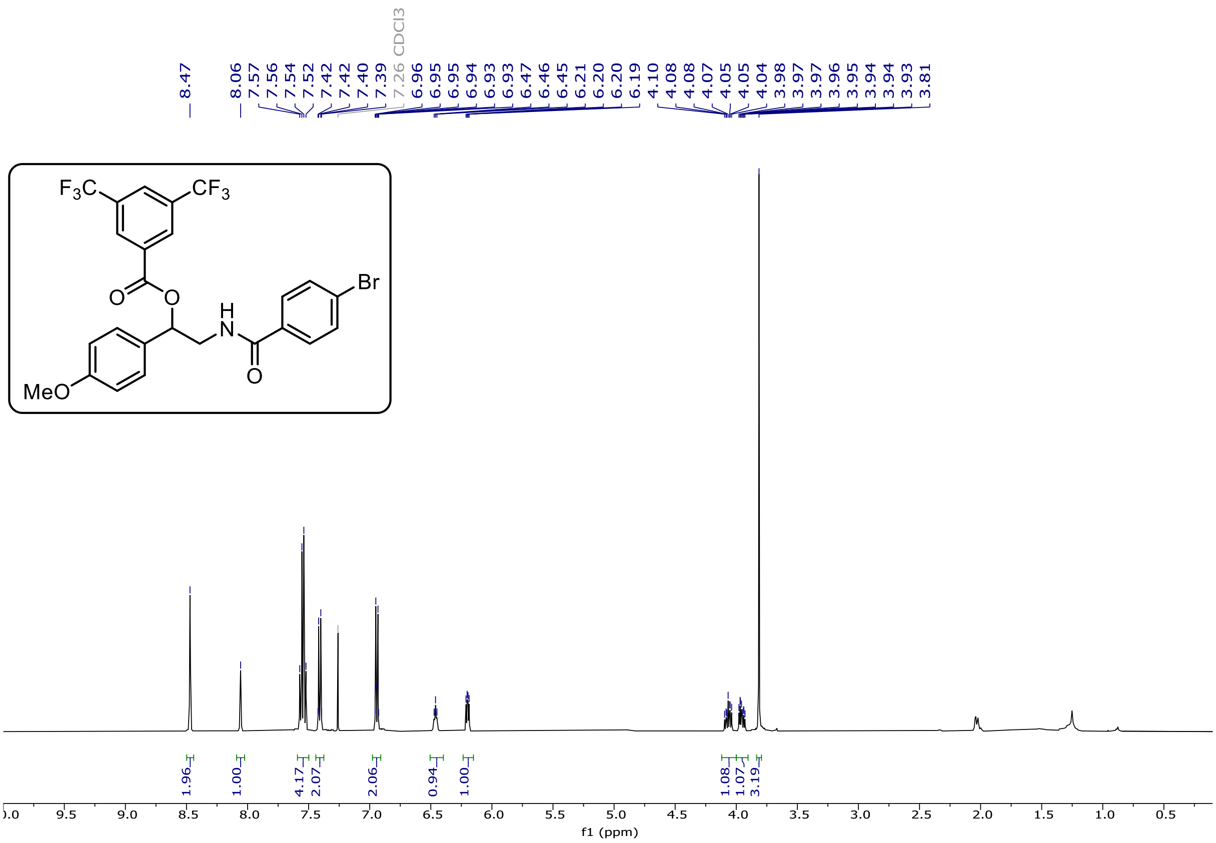

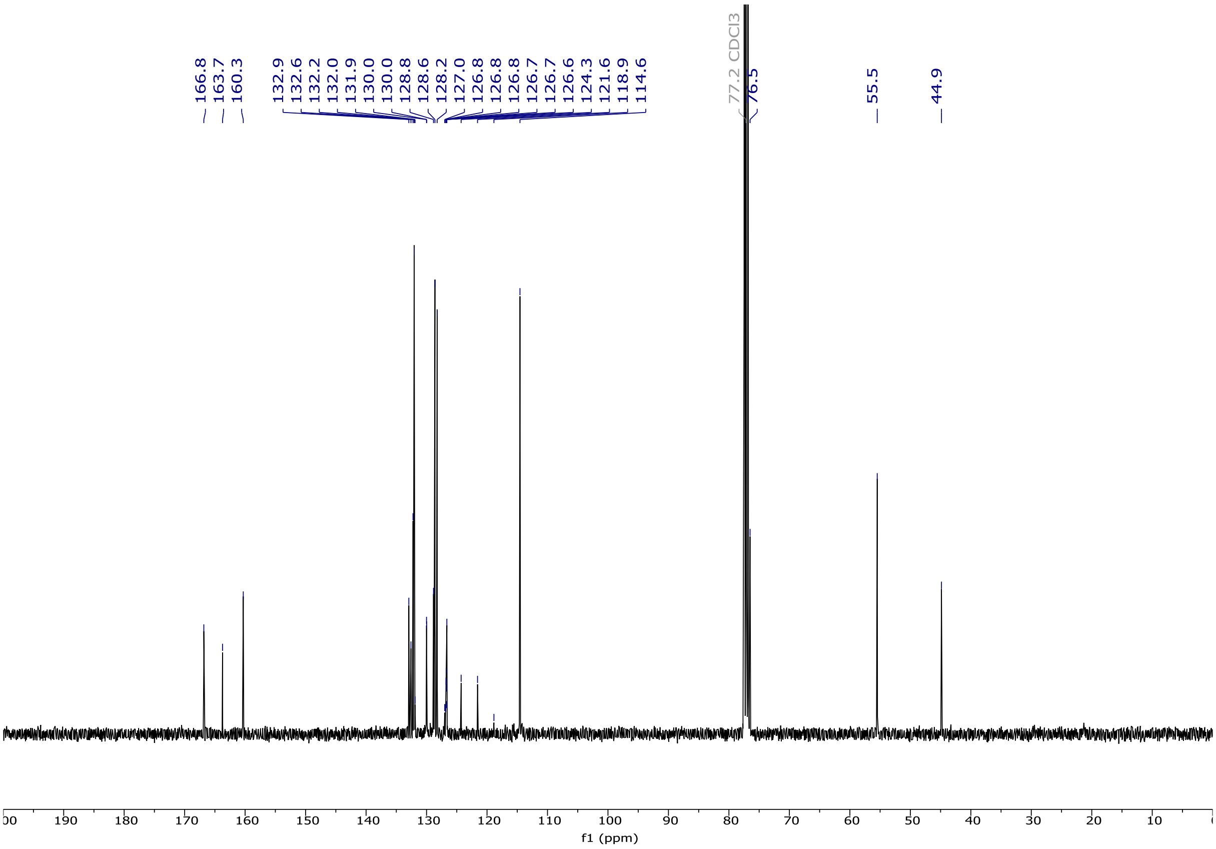

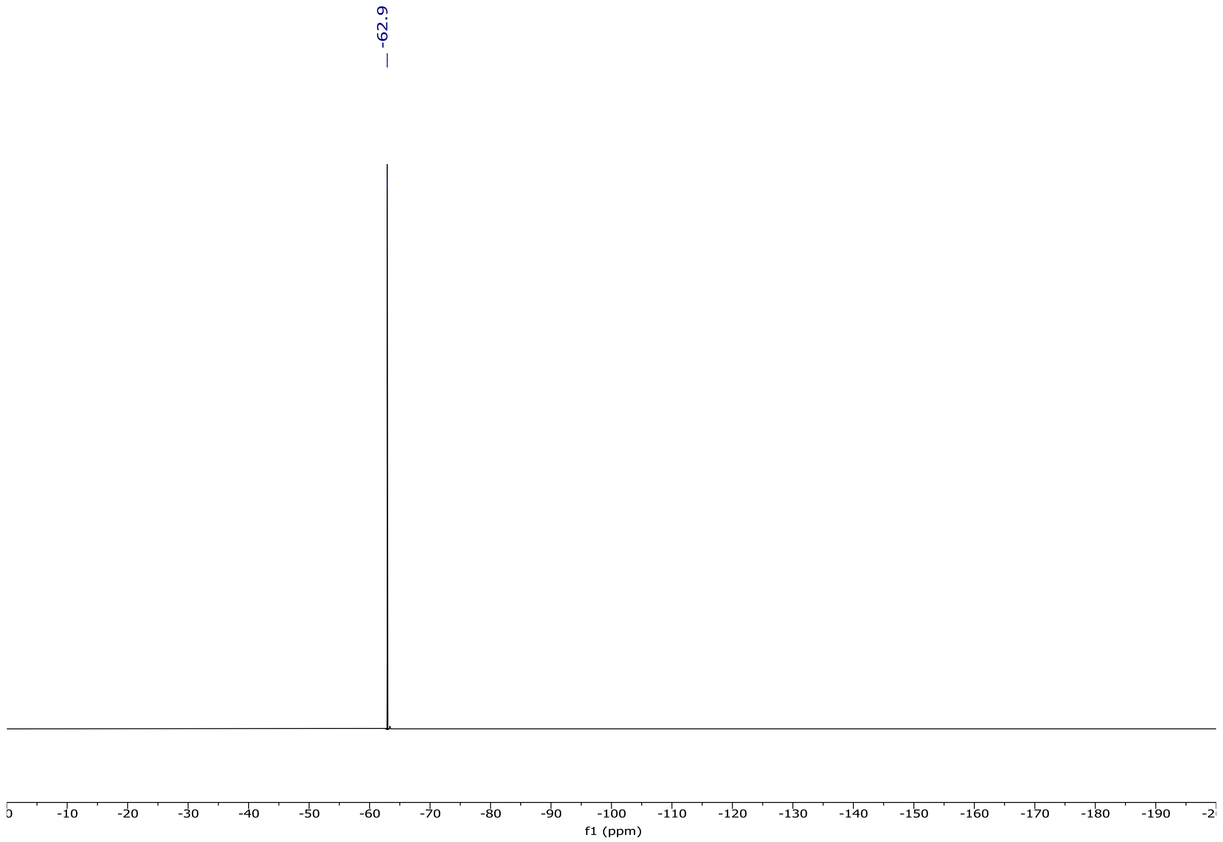

-62.9
0
-10
-20
-30
-40
-50
-60
-70
-80
-90
-100
-110
-120
-130
-140
-150
-160
-170
-180
-190
f1 (ppm)

1-(4-Methoxyphenyl)-2-(thiophene-3-carboxamido)ethyl 3,5-bis(trifluoromethyl)benzoate (3n)

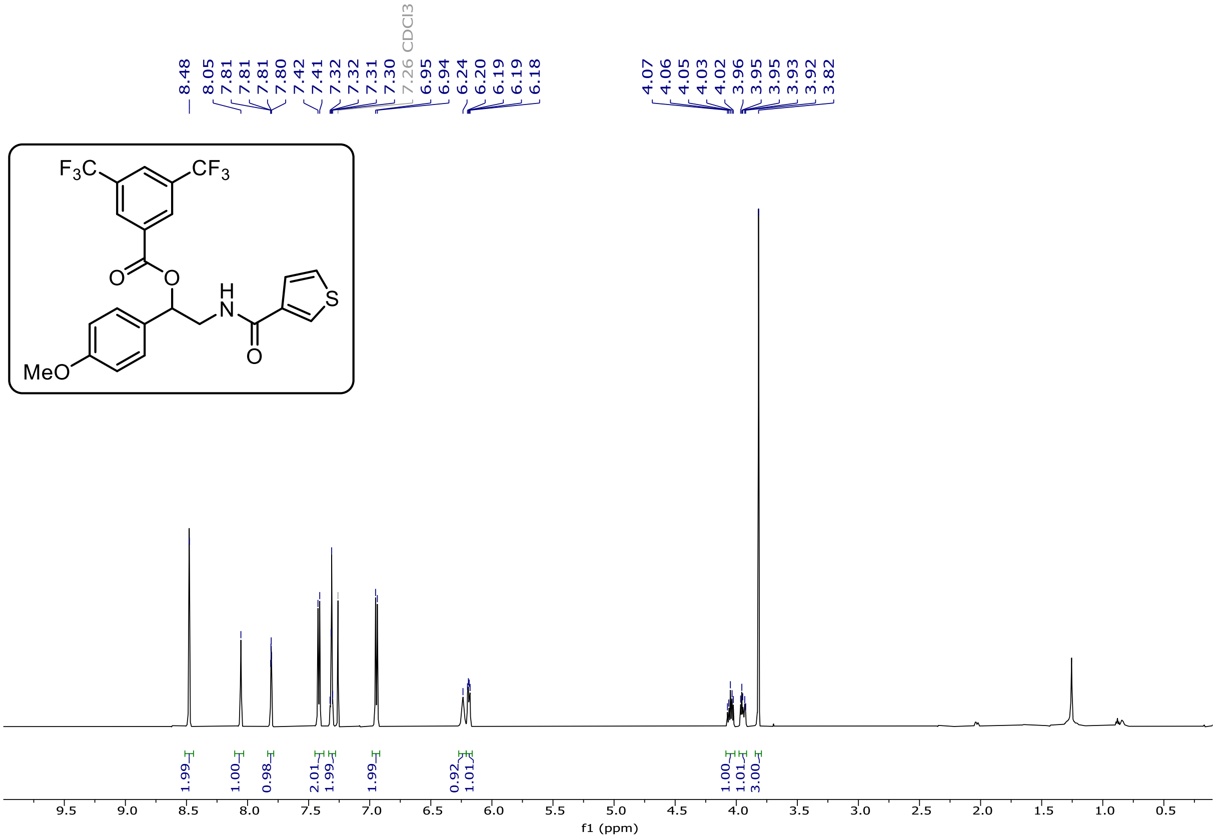

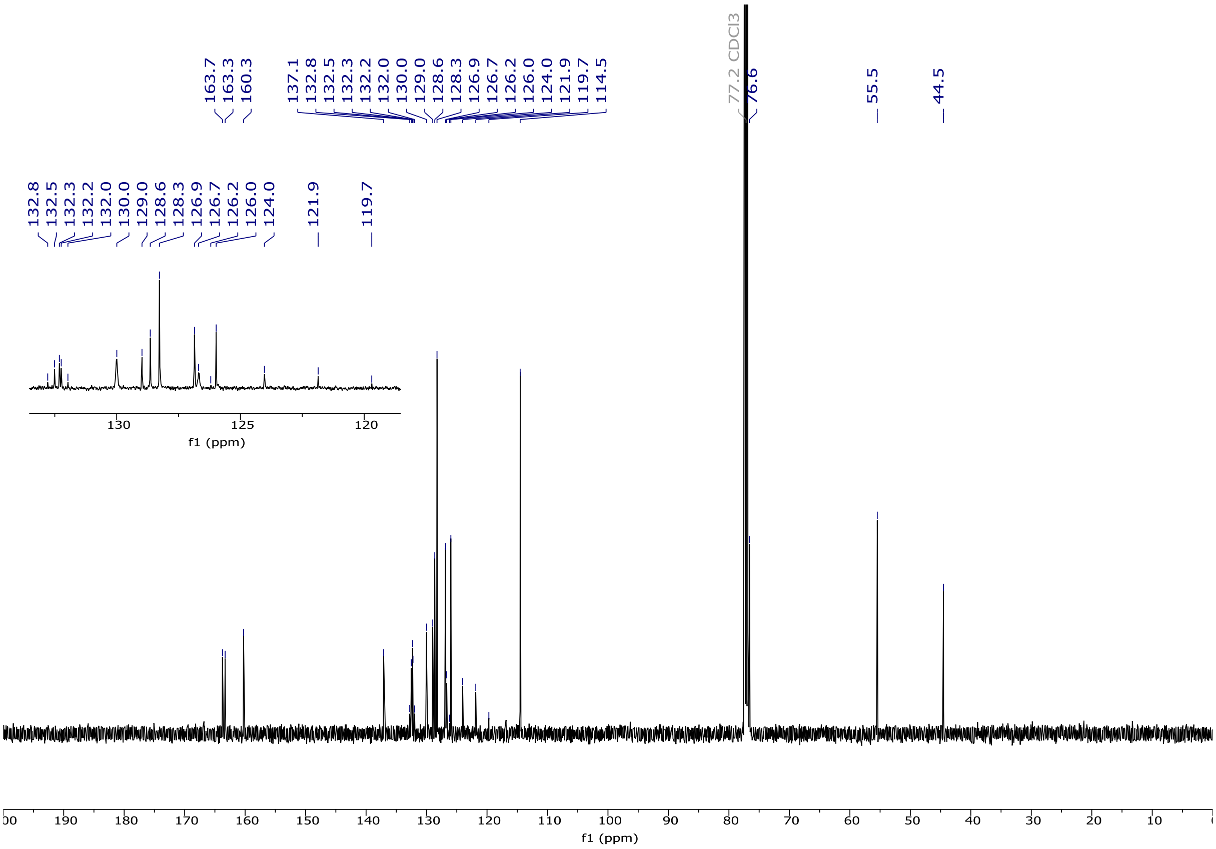

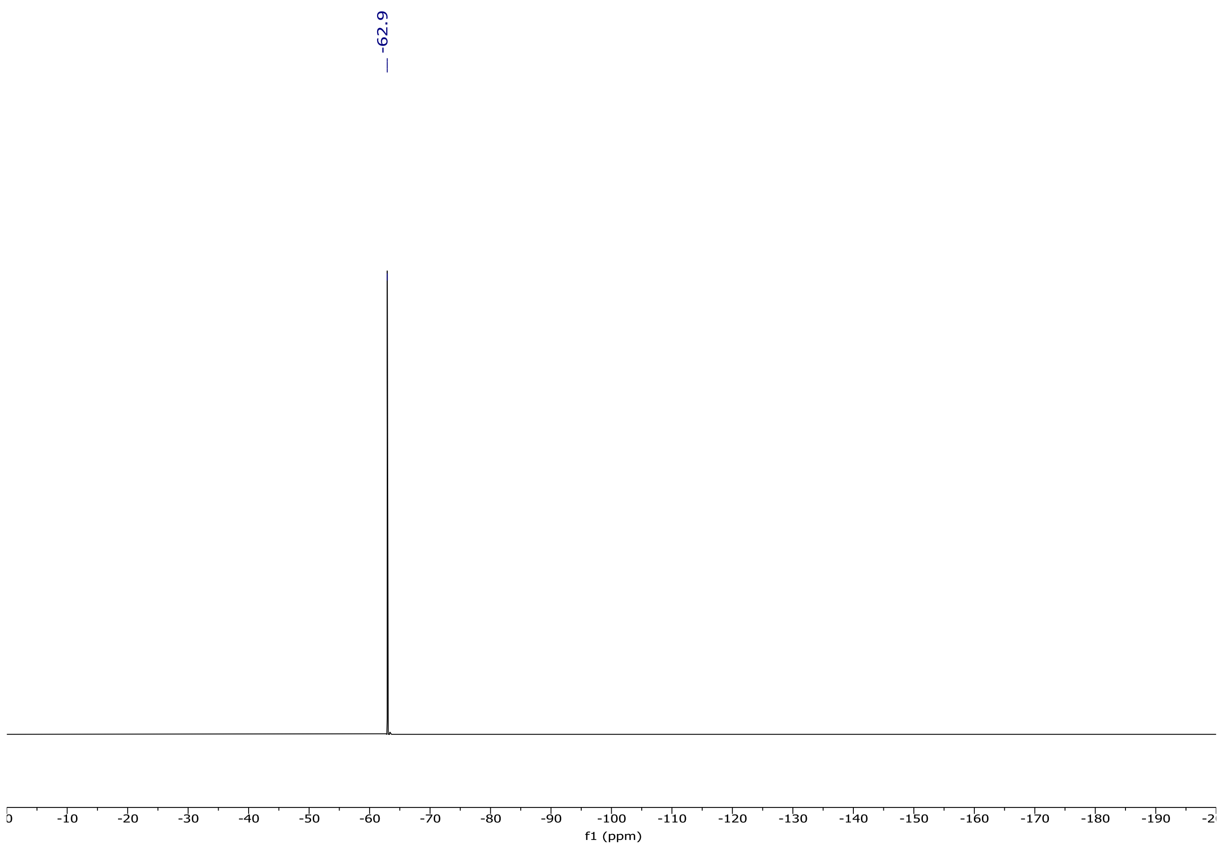
-62.9
0
-10
-20
-30
-40
-50
-60
-70
-80
-90
-100
-110
-120
-130
-140
-150
-160
-170
-180
-190
-2
f1 (ppm)

2-(Cyclohexanecarboxamido)-1-(4-methoxyphenyl)ethyl 3,5-bis(trifluoromethyl)benzoate (3o)

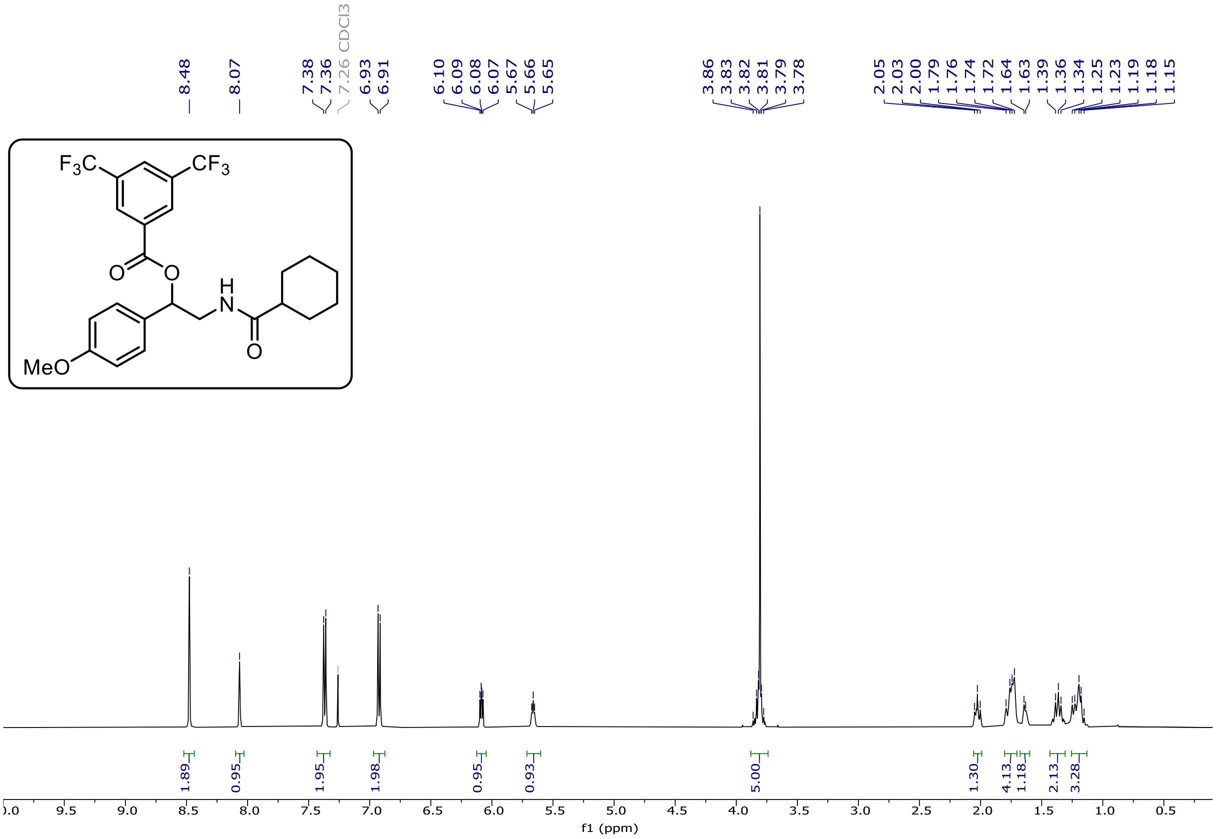

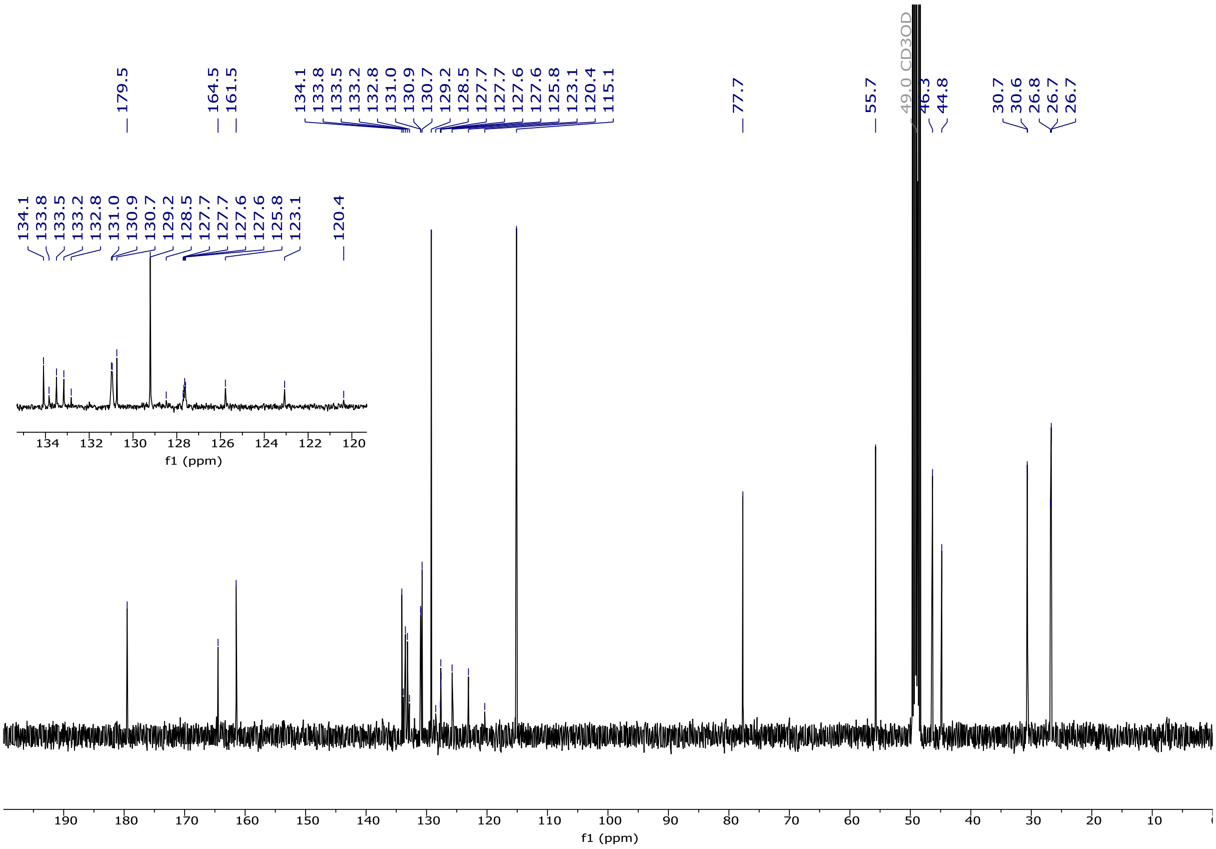

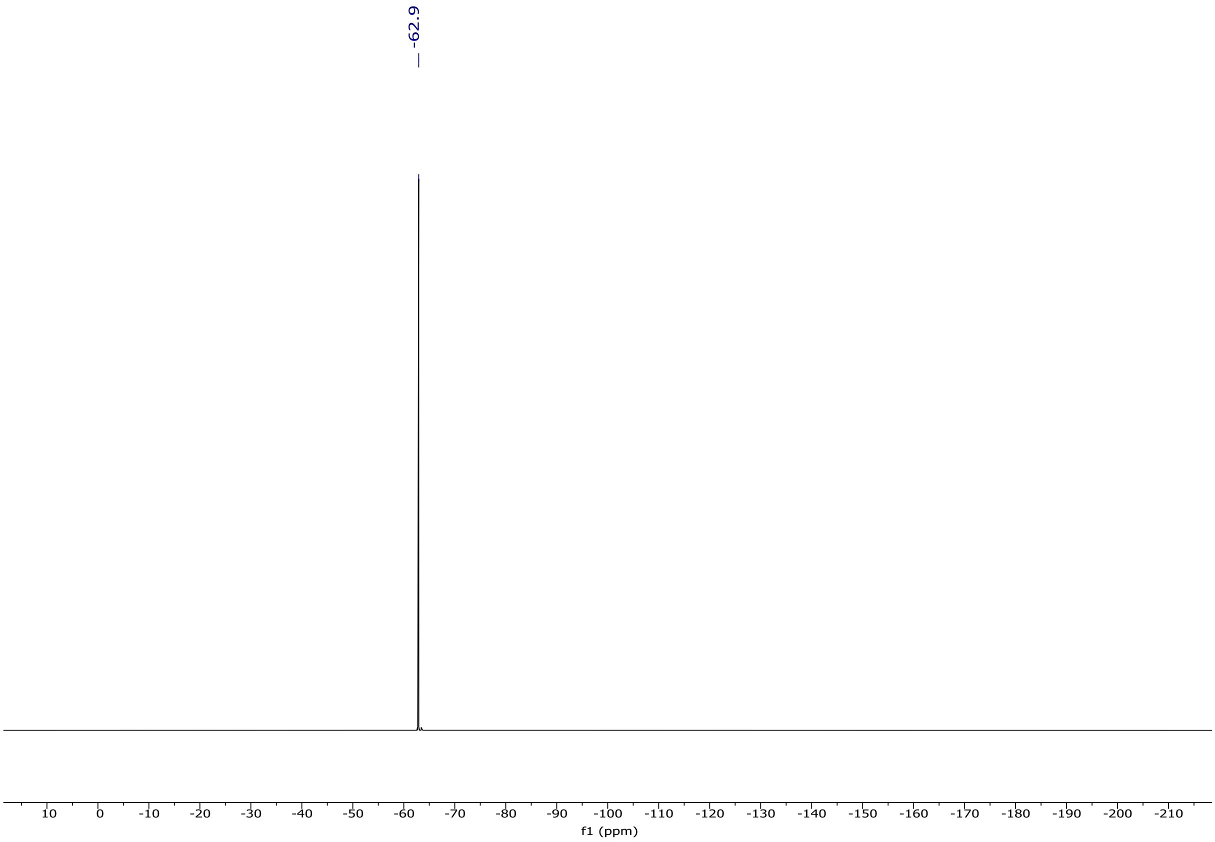
-62.9
10
0
-10
-20
-30
-40
-50
-60
-70
-80
-90
-100
-110
-120
-130
-140
-150
-160
-170
-180
-190
-200
-210
f1 (ppm)

N-(2-Methoxy-2-phenylethyl)benzamide (4a)

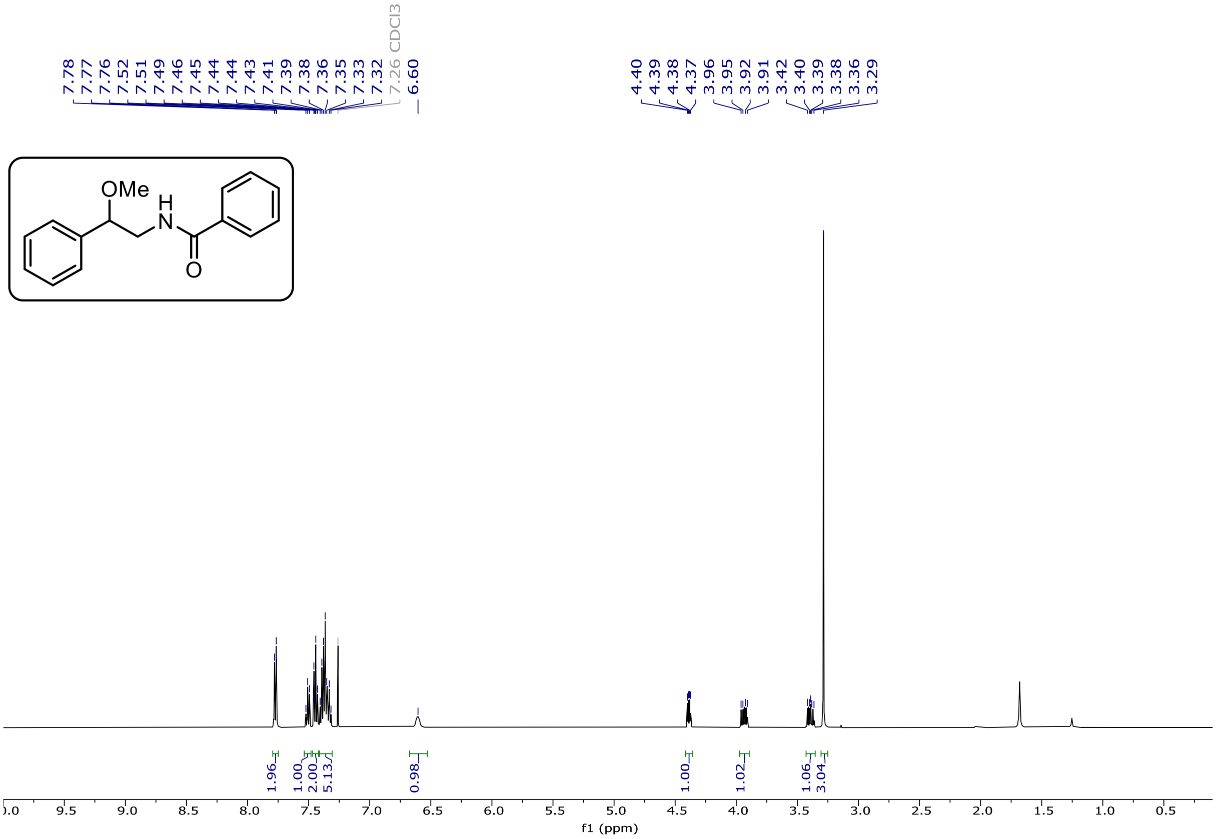

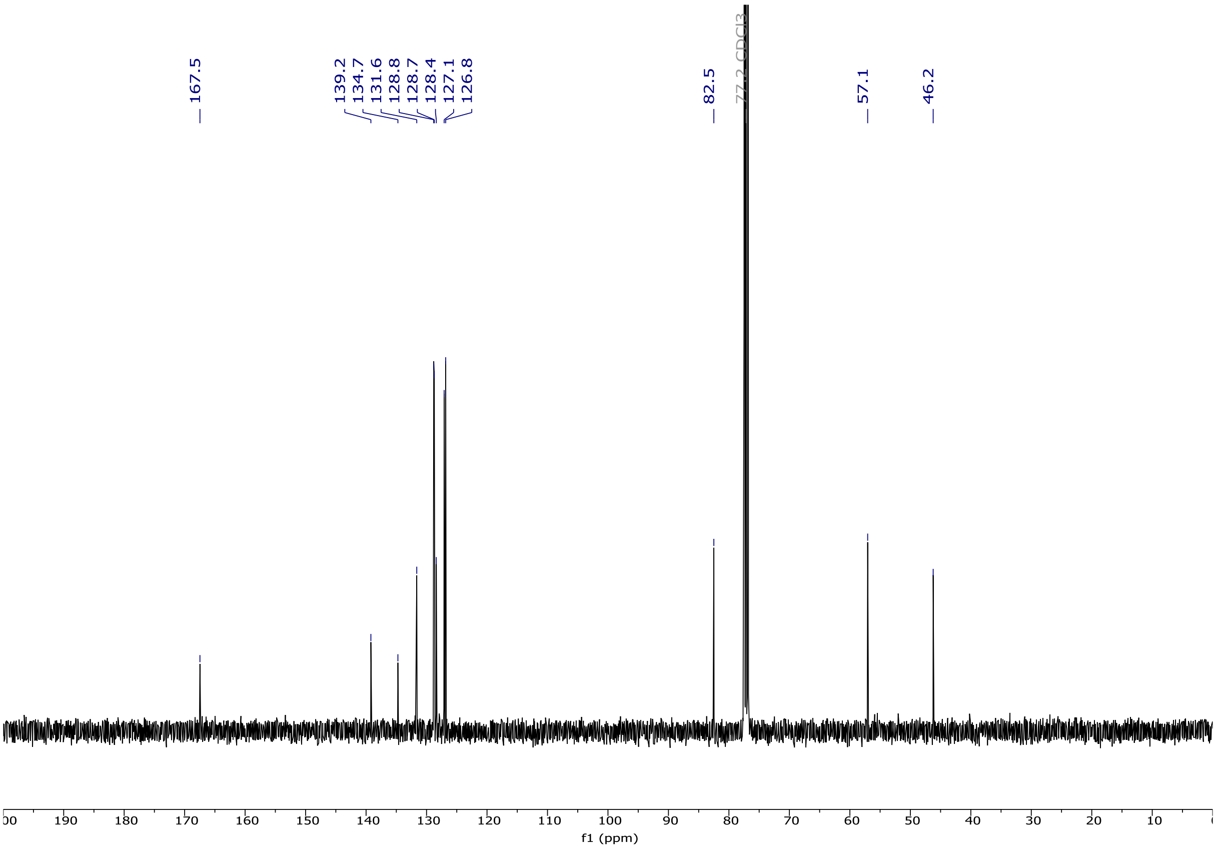

N-{2-Methoxy-2-(p-tolyl)ethyl}benzamide (4b)

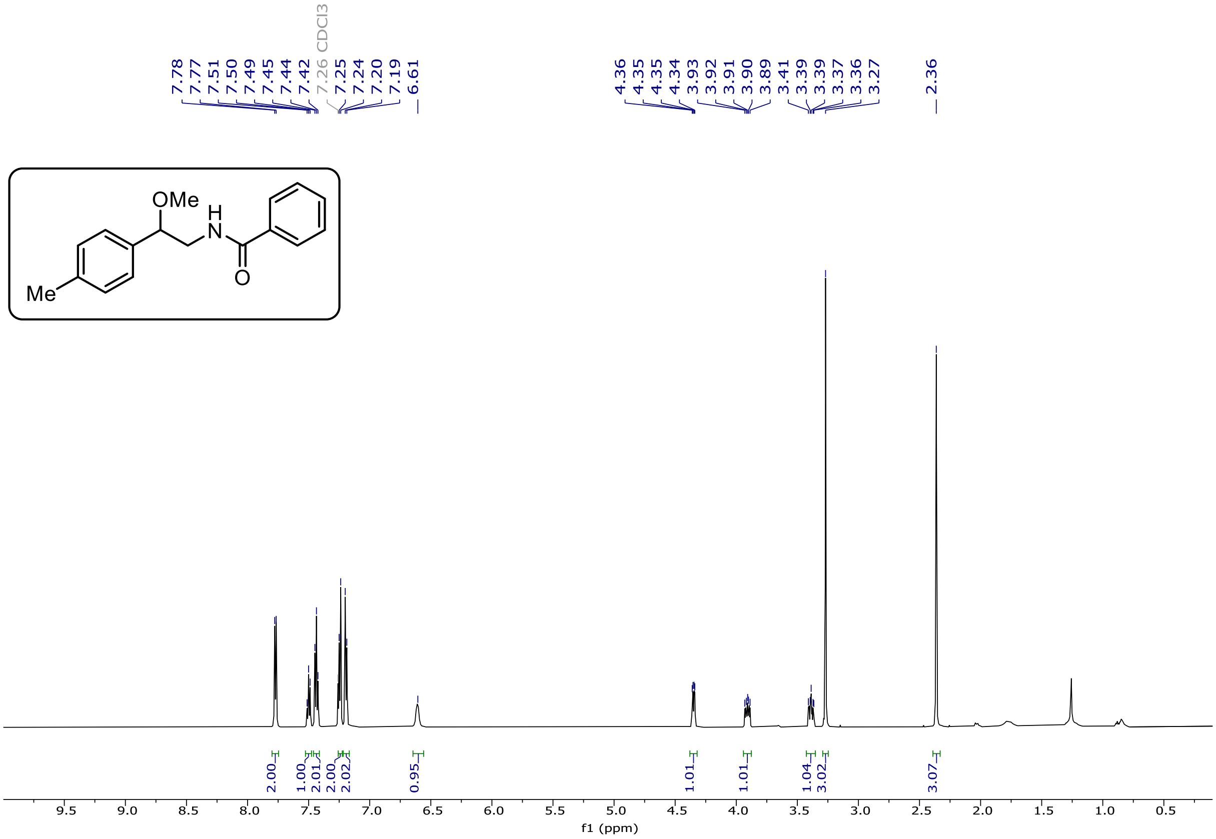

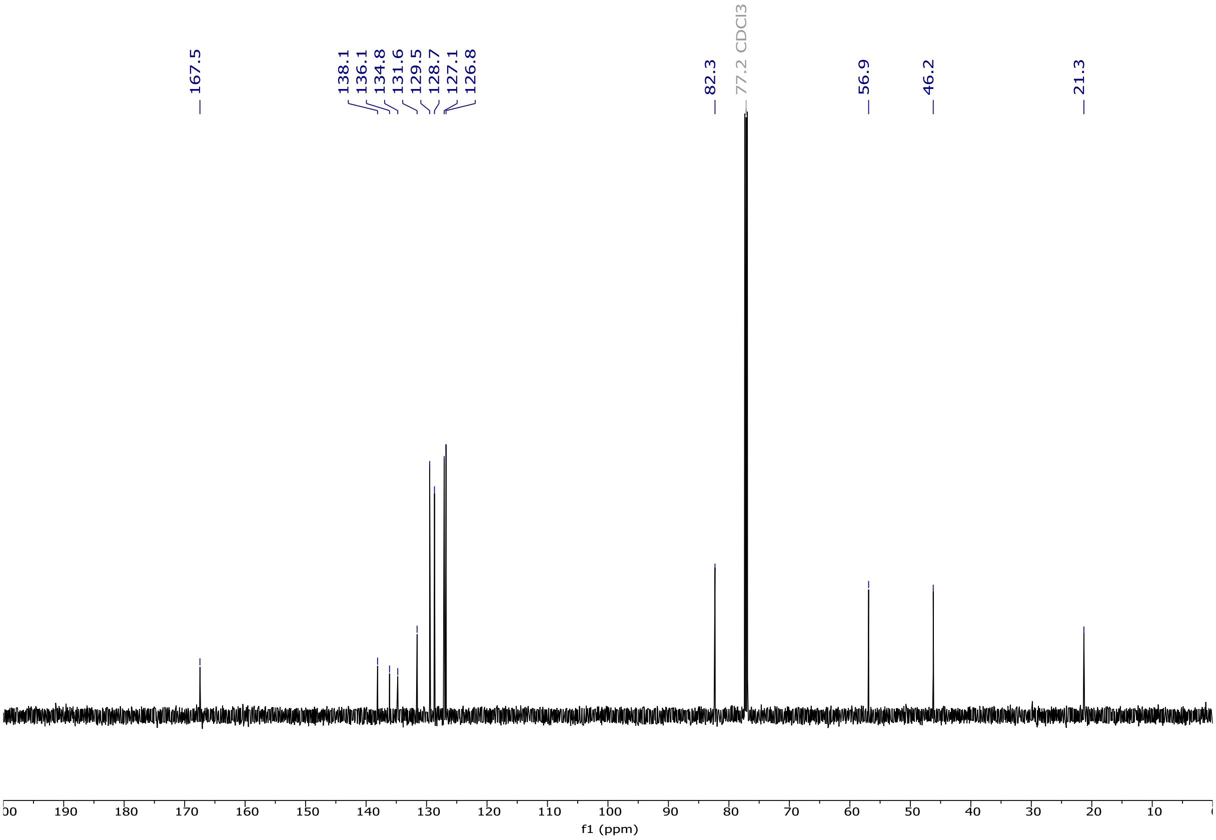

N-[2-{4-(*tert*-Butyl)phenyl}-2-methoxyethyl]benzamide (4c)

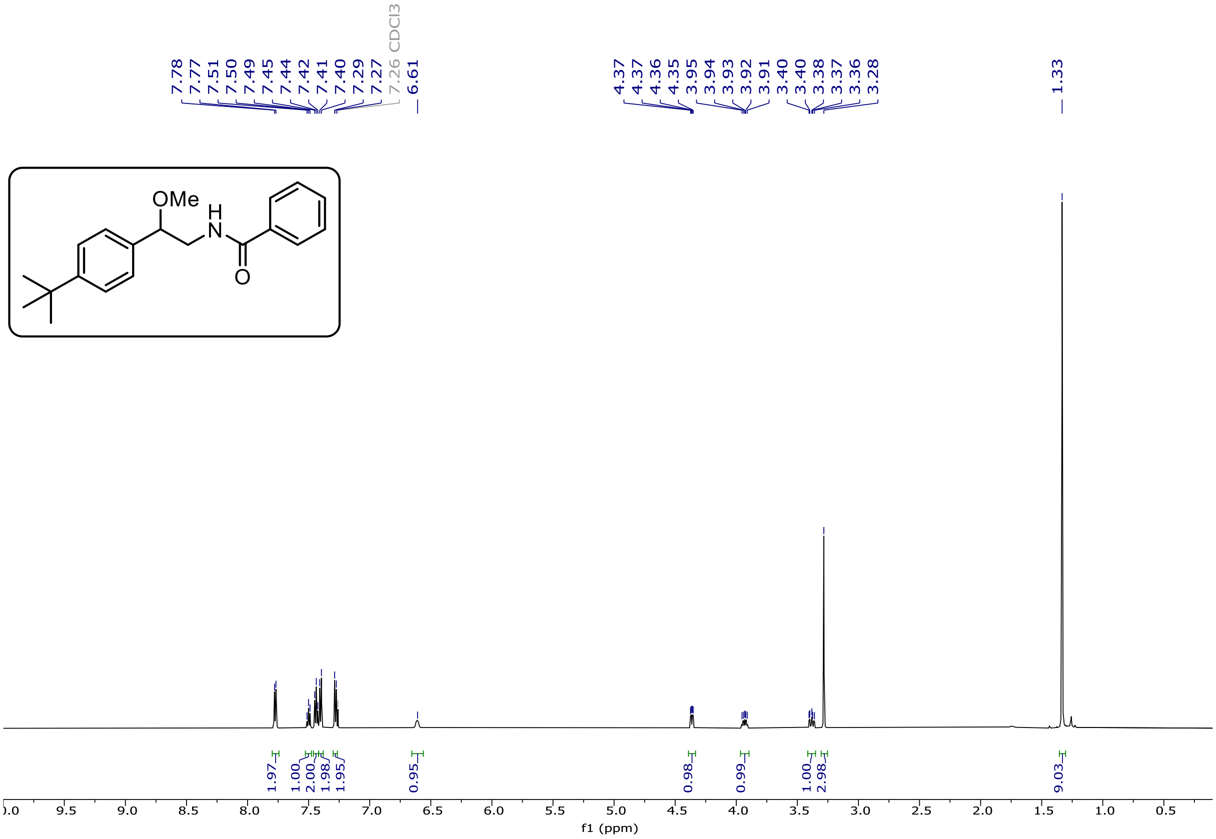

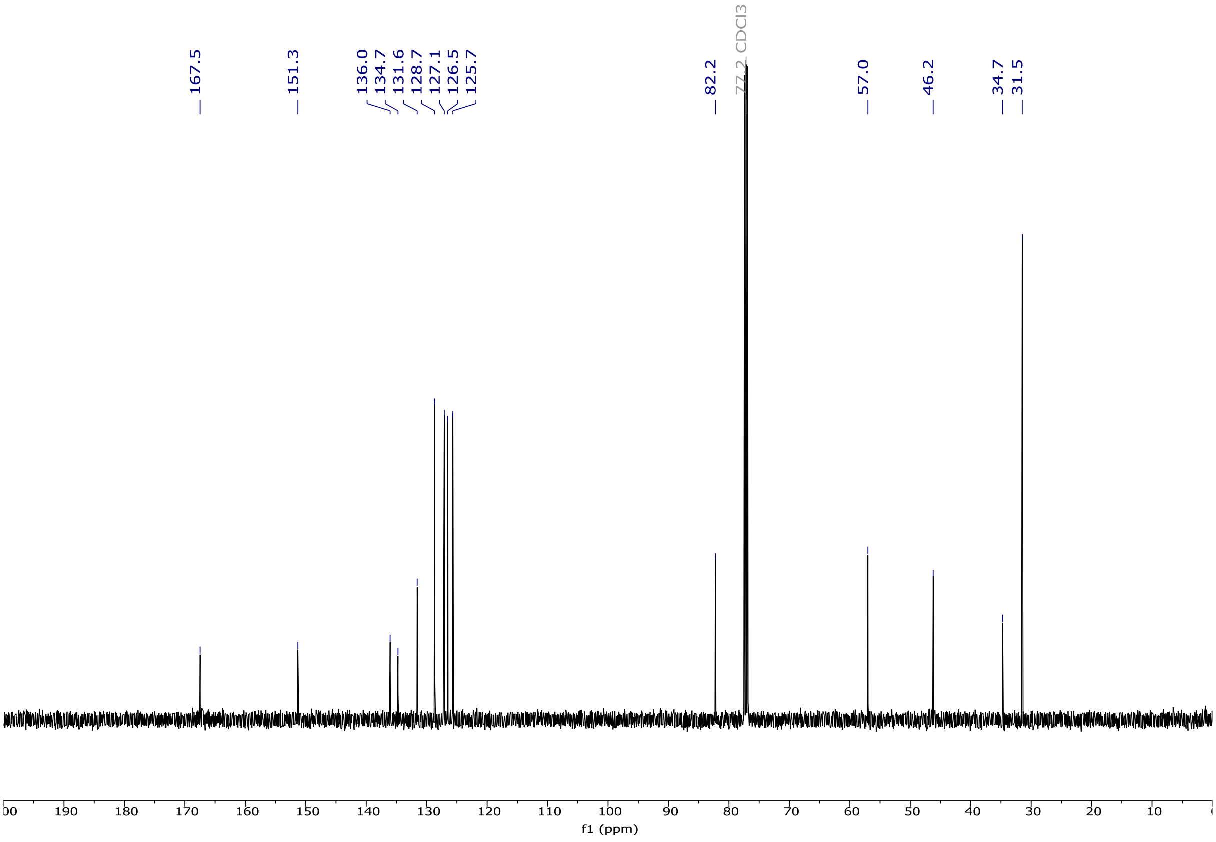

N-{2-Methoxy-2-(4-methoxyphenyl)ethyl}benzamide (4d)

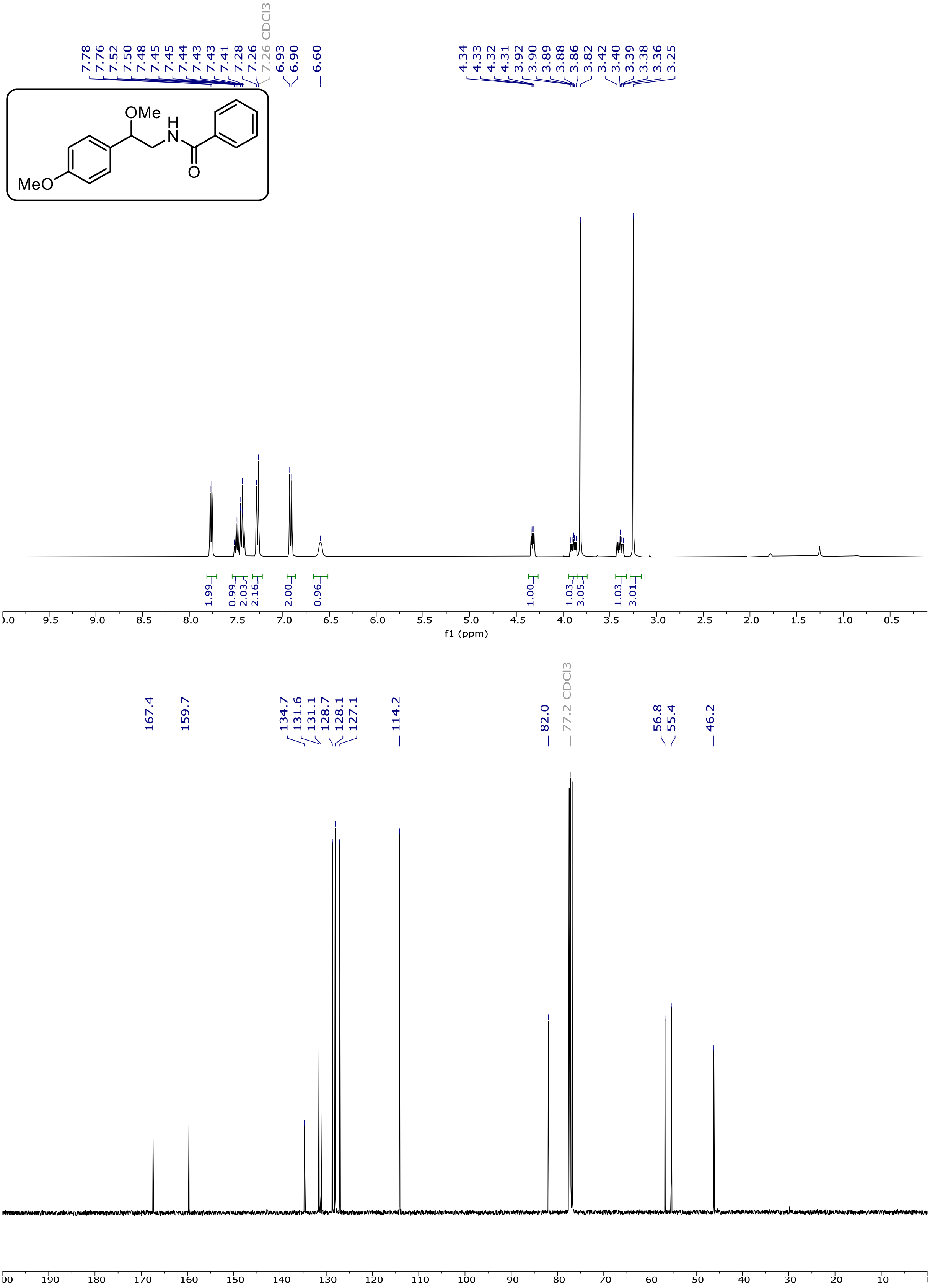

4-(2-Benzamido-1-methoxyethyl)phenyl acetate (4e)

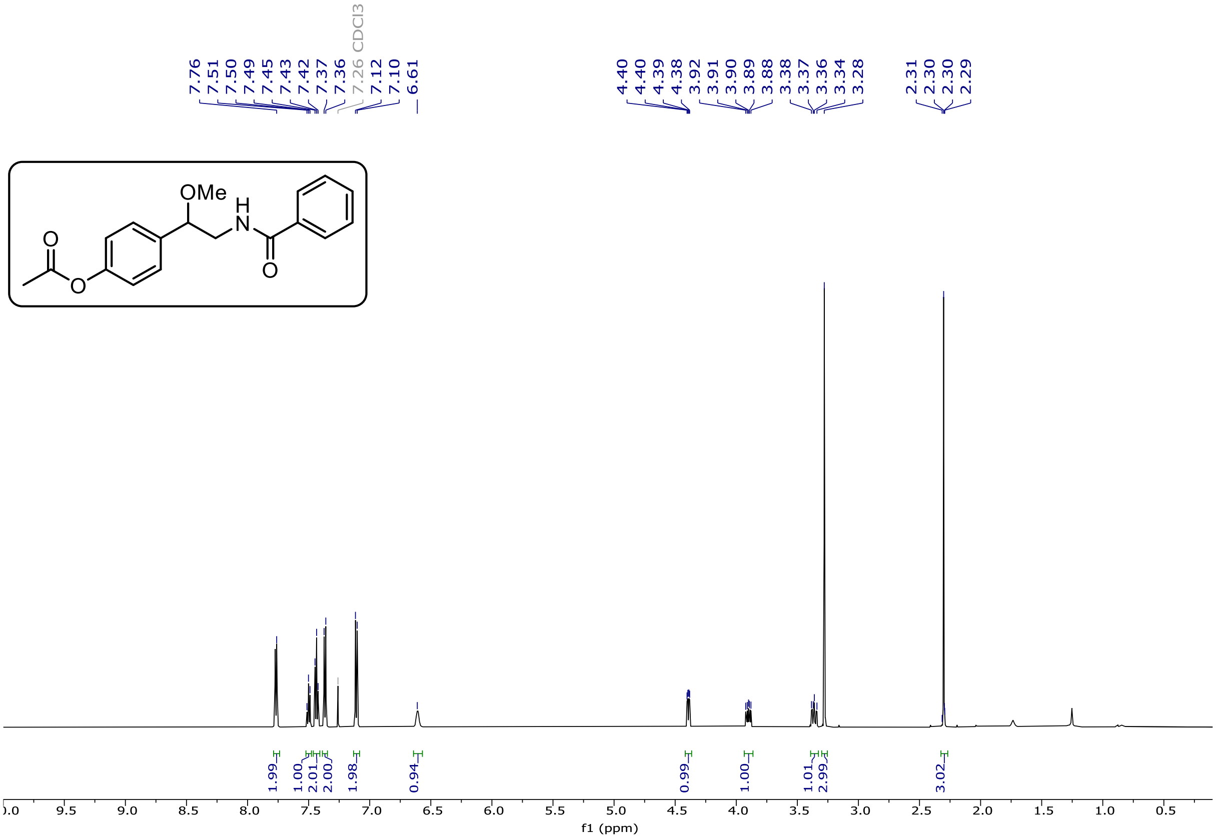

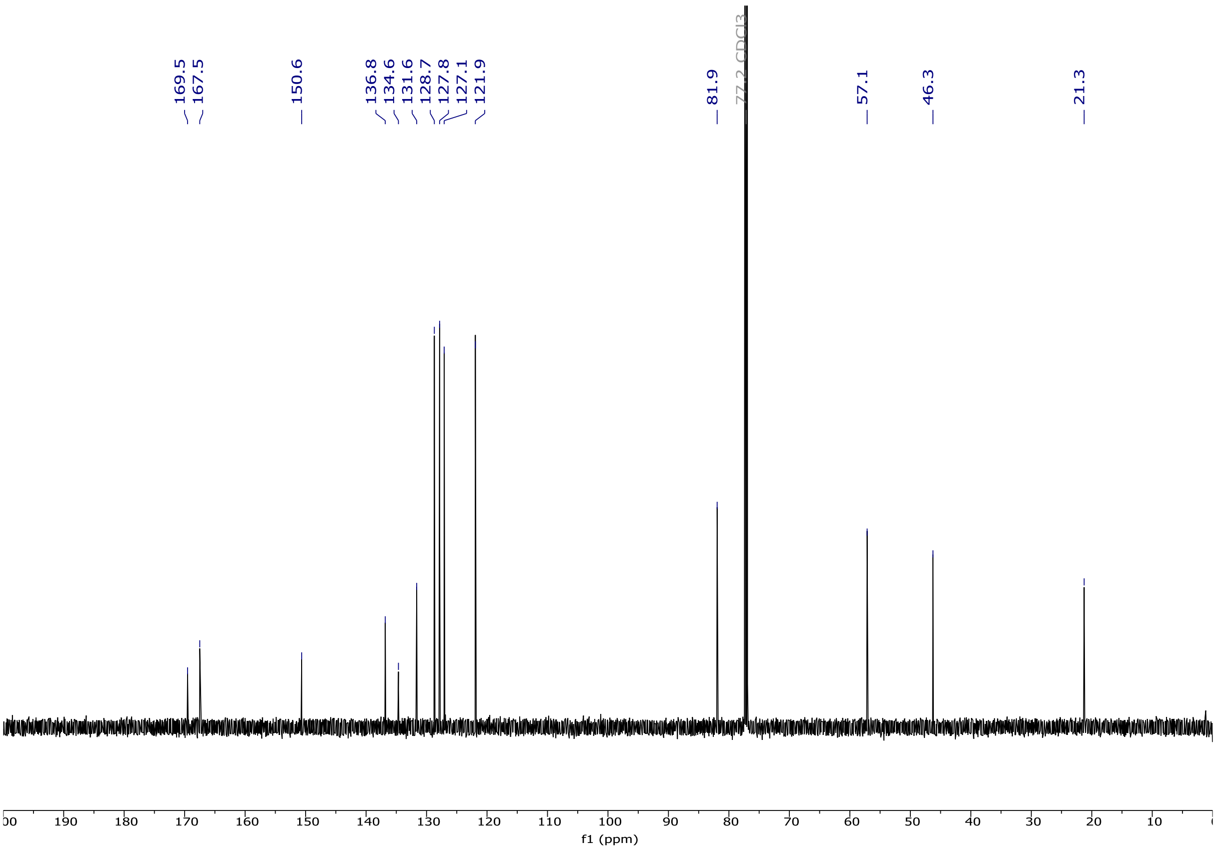

N-{2-(4-Fluorophenyl)-2-methoxyethyl}benzamide (4f)

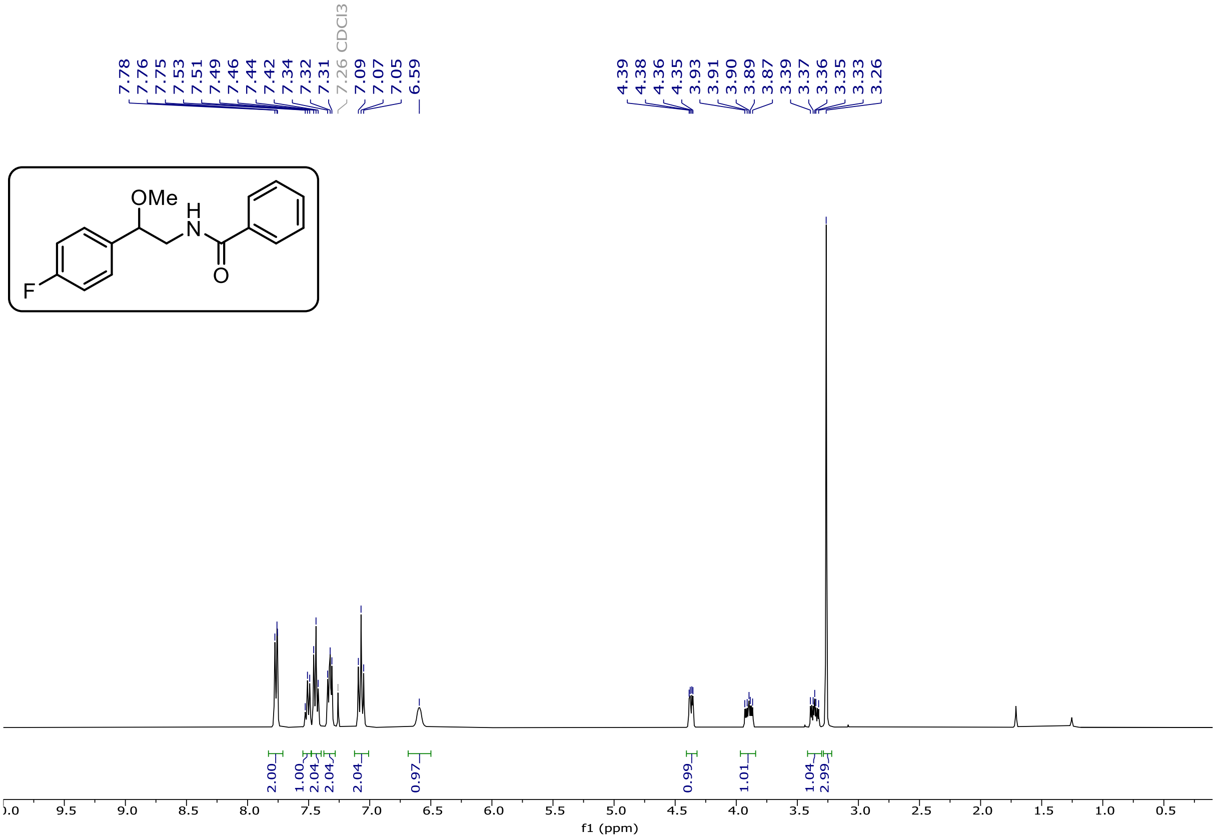

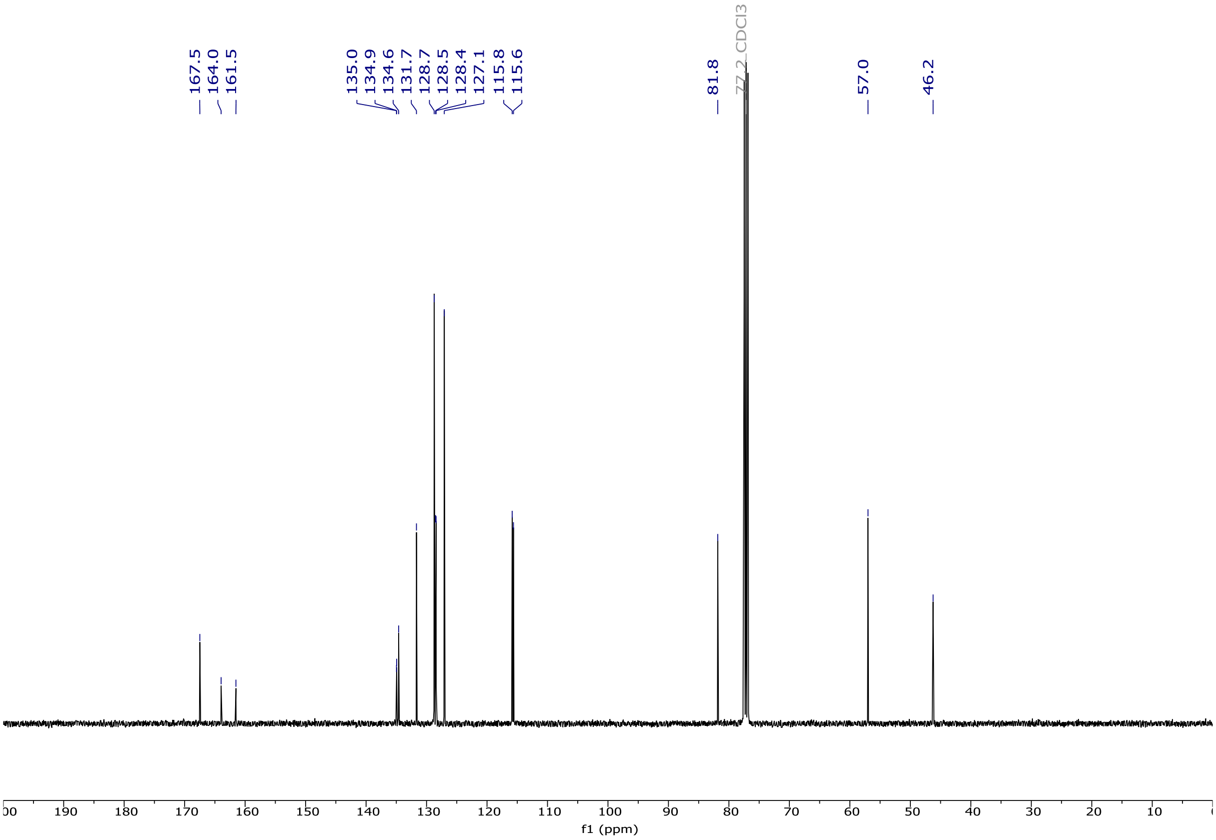

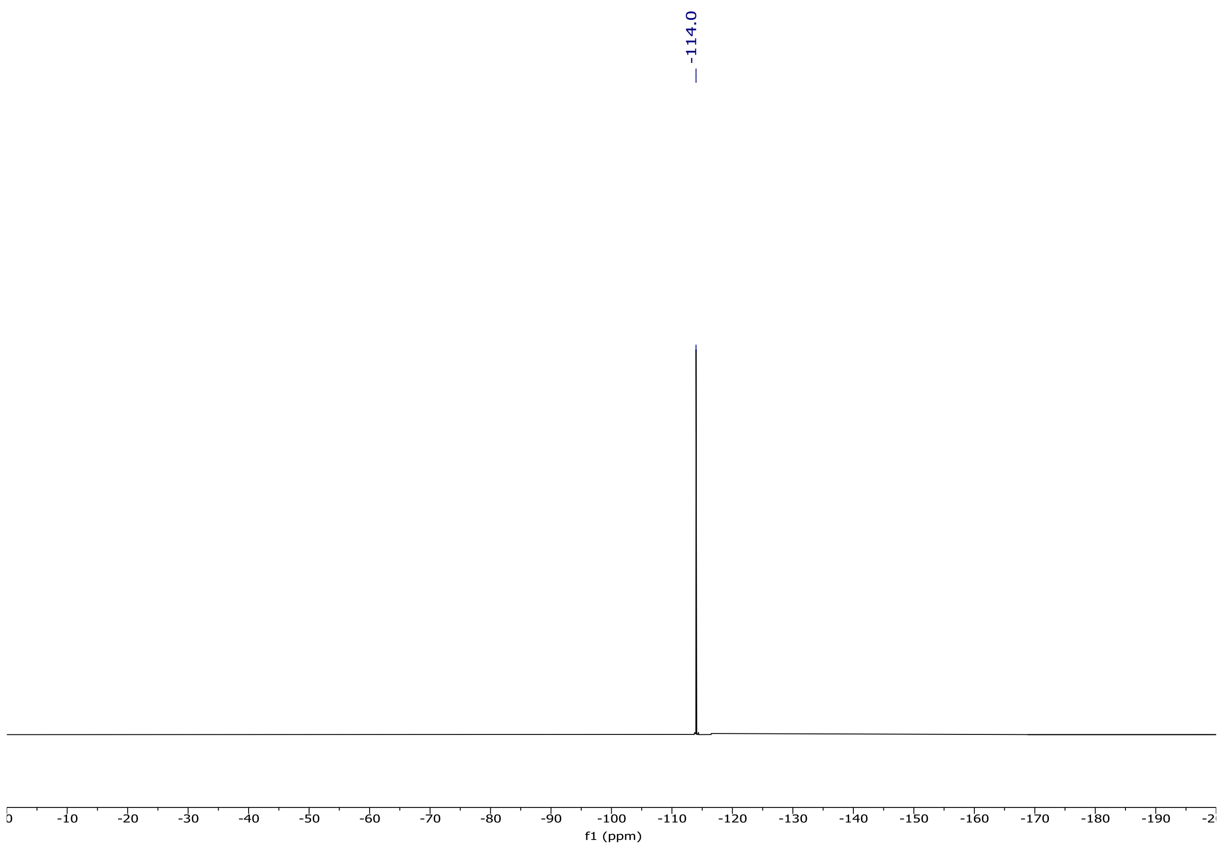
-114.0
0
-10
-20
-30
-40
-50
-60
-70
-80
-90
-100
-110
-120
-130
-140
-150
-160
-170
-180
-190
-2
f1 (ppm)

N-{2-Methoxy-2-(*o*-tolyl)ethyl}benzamide (4g)

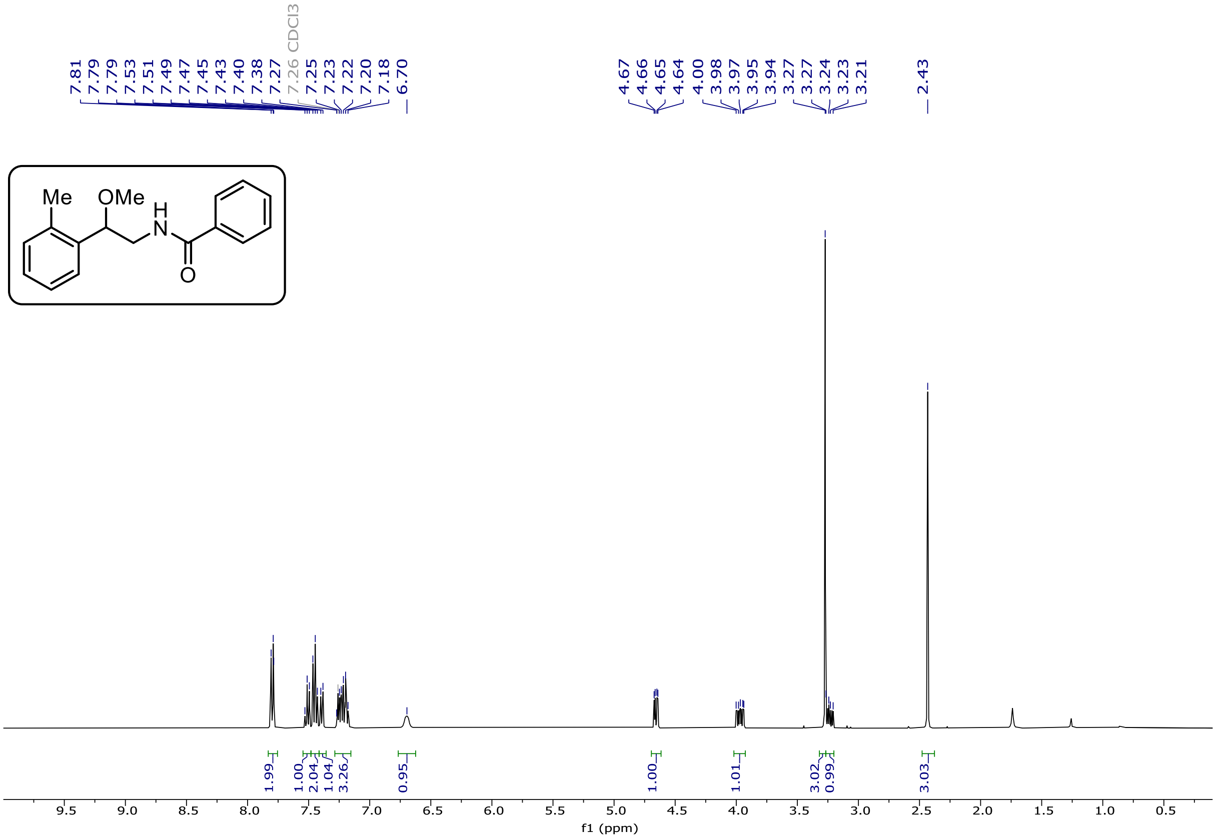

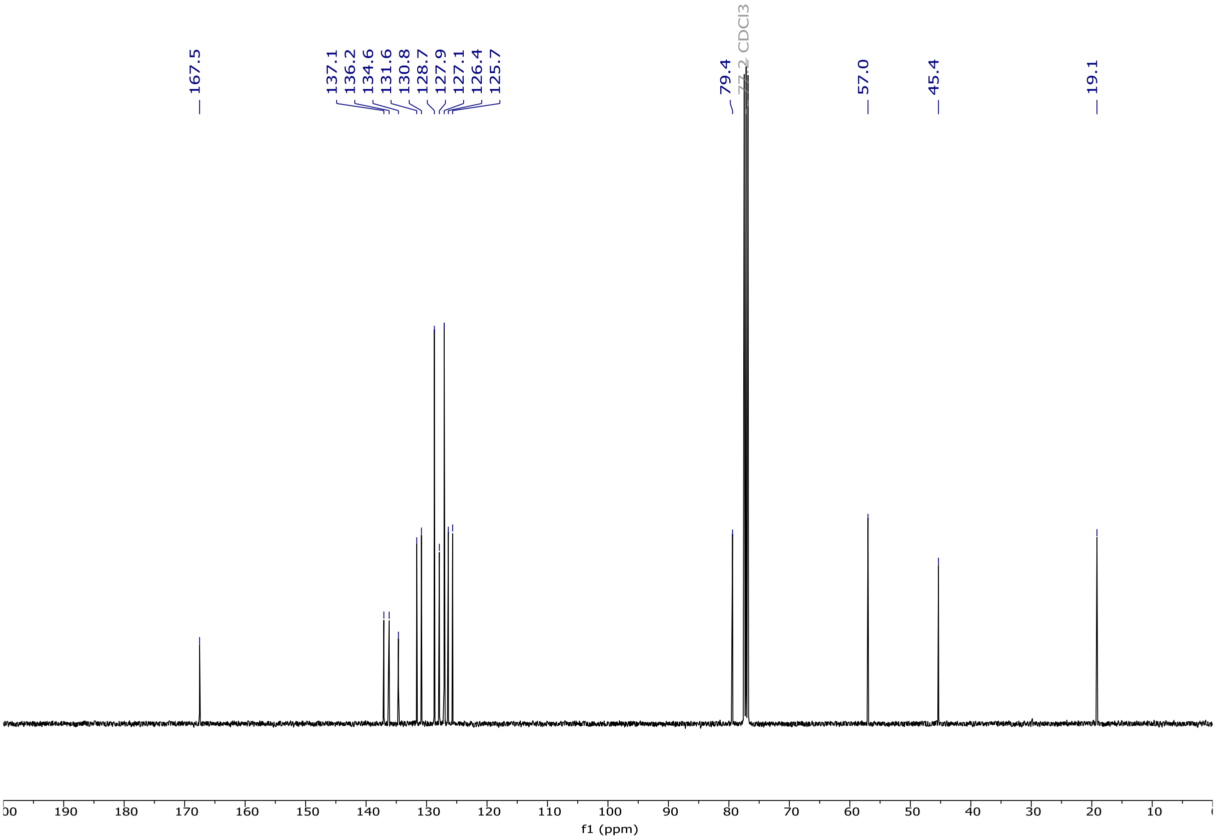

N−{2-(2-Bromophenyl)-2-methoxyethyl}benzamide (4h)

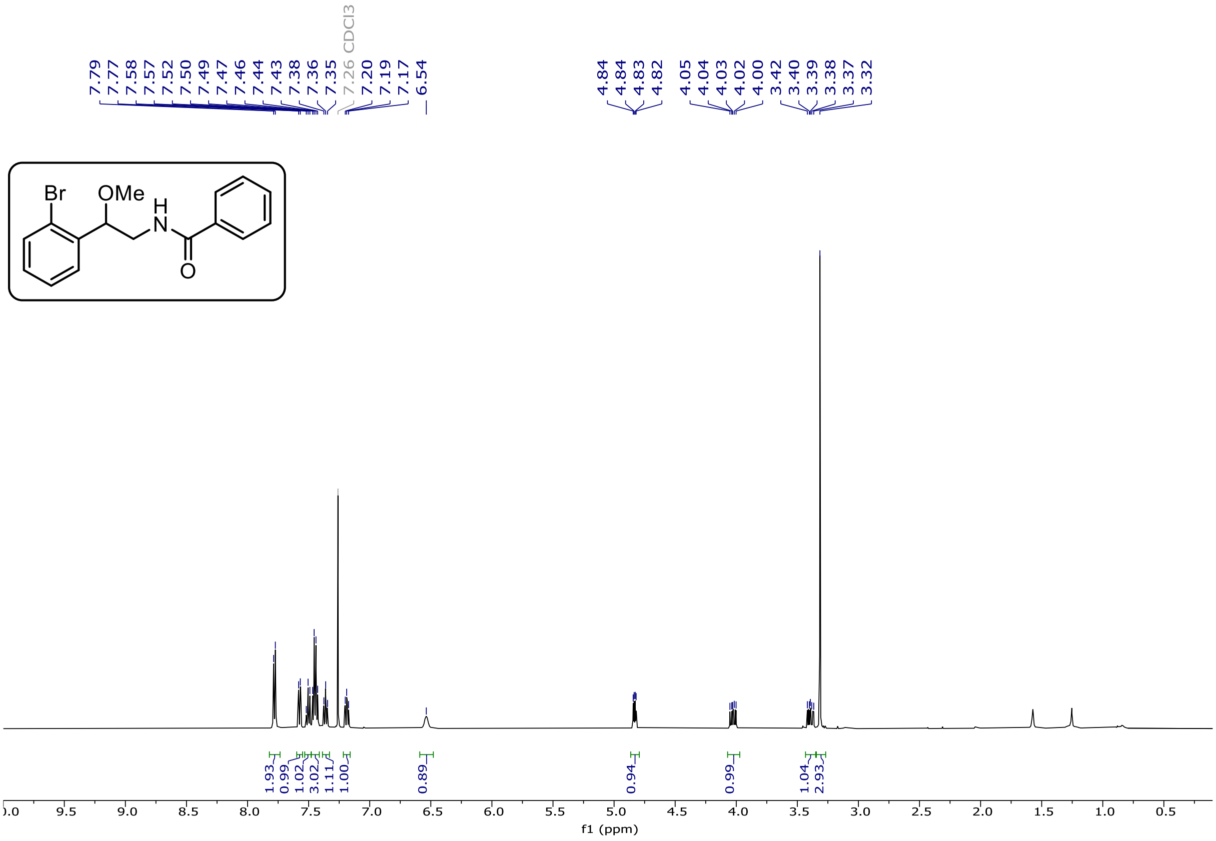

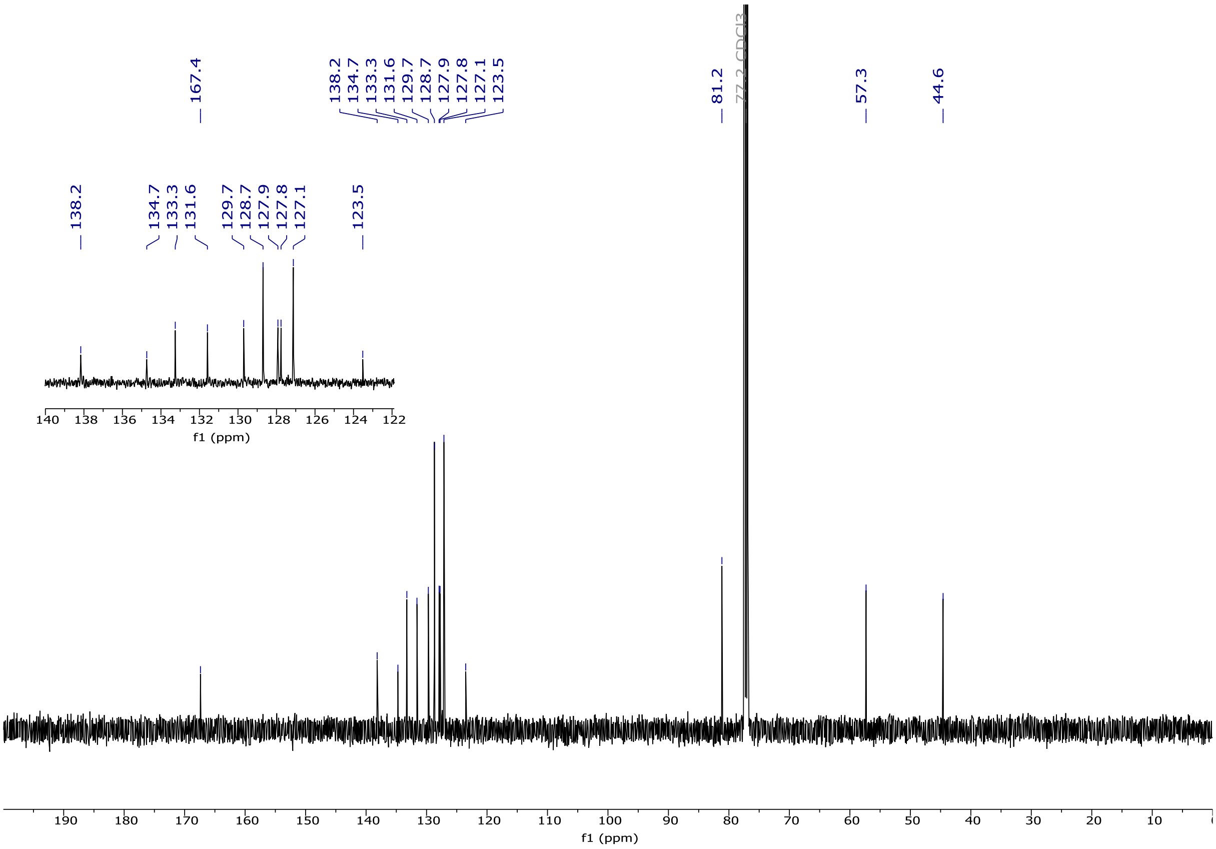

N-{2-Methoxy-2-(3-methoxyphenyl)ethyl}benzamide (4i)

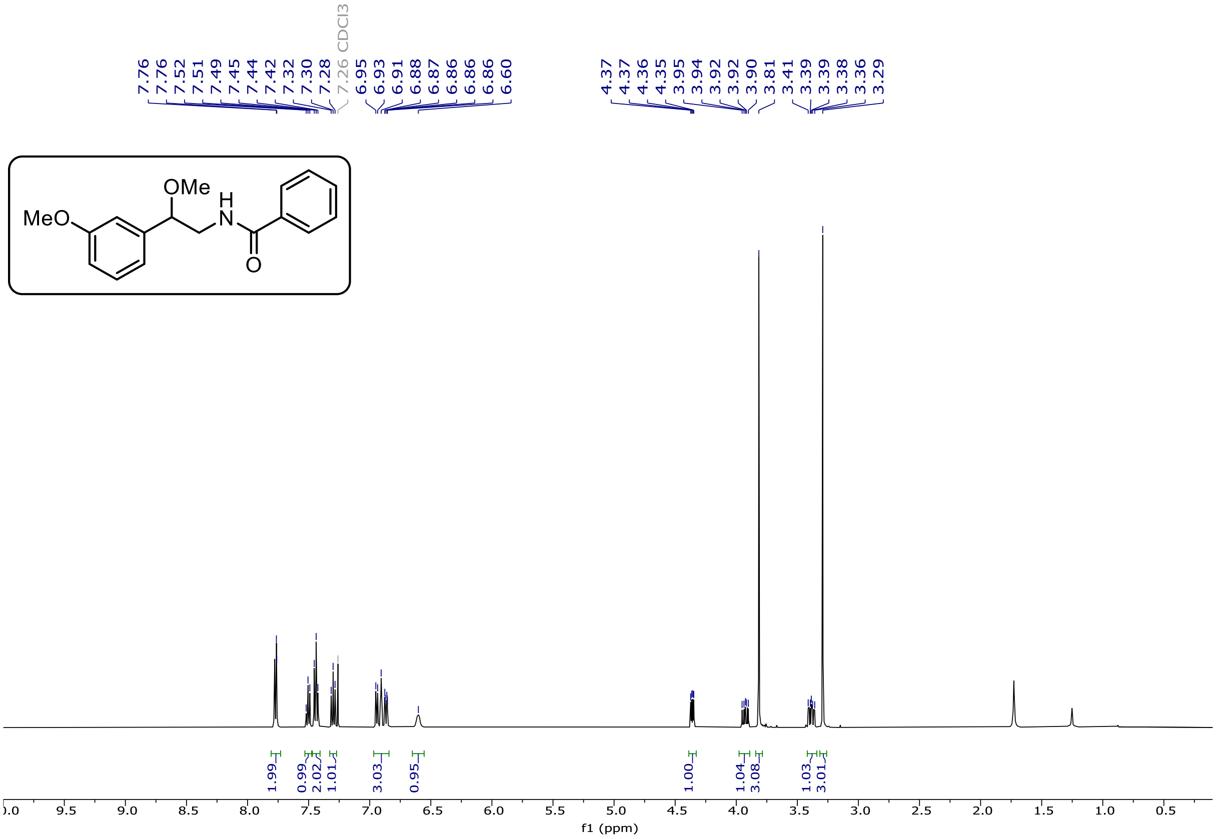

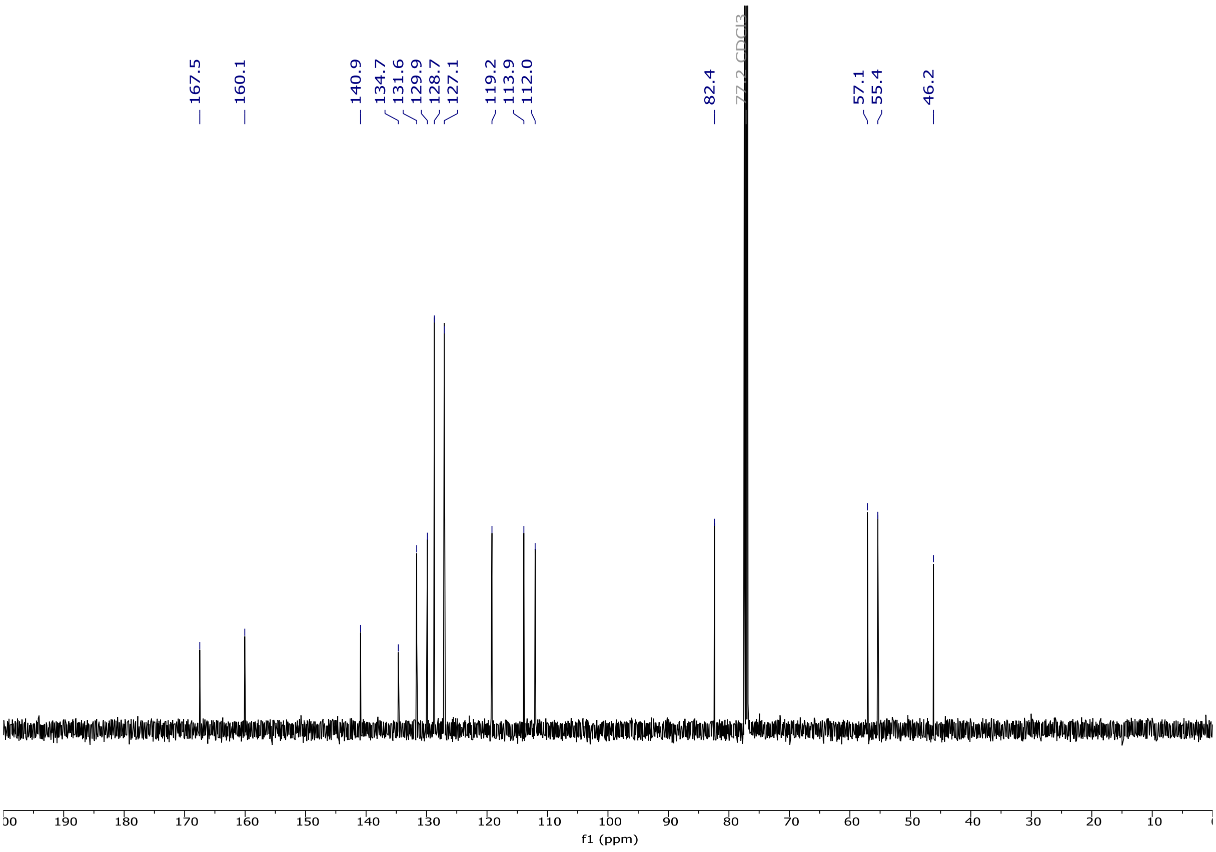

_N_-{2-Methoxy-2-(thiophen-2-yl)ethyl}benzamide (4j)

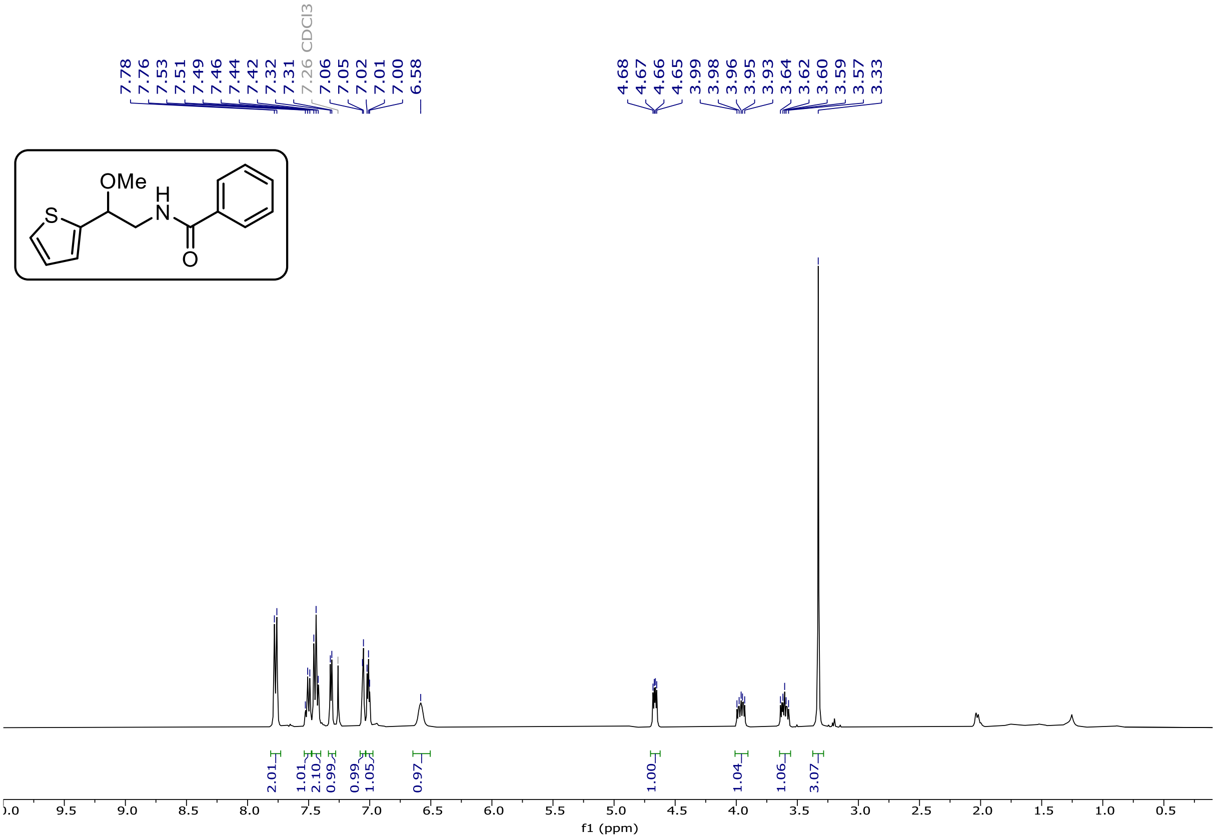

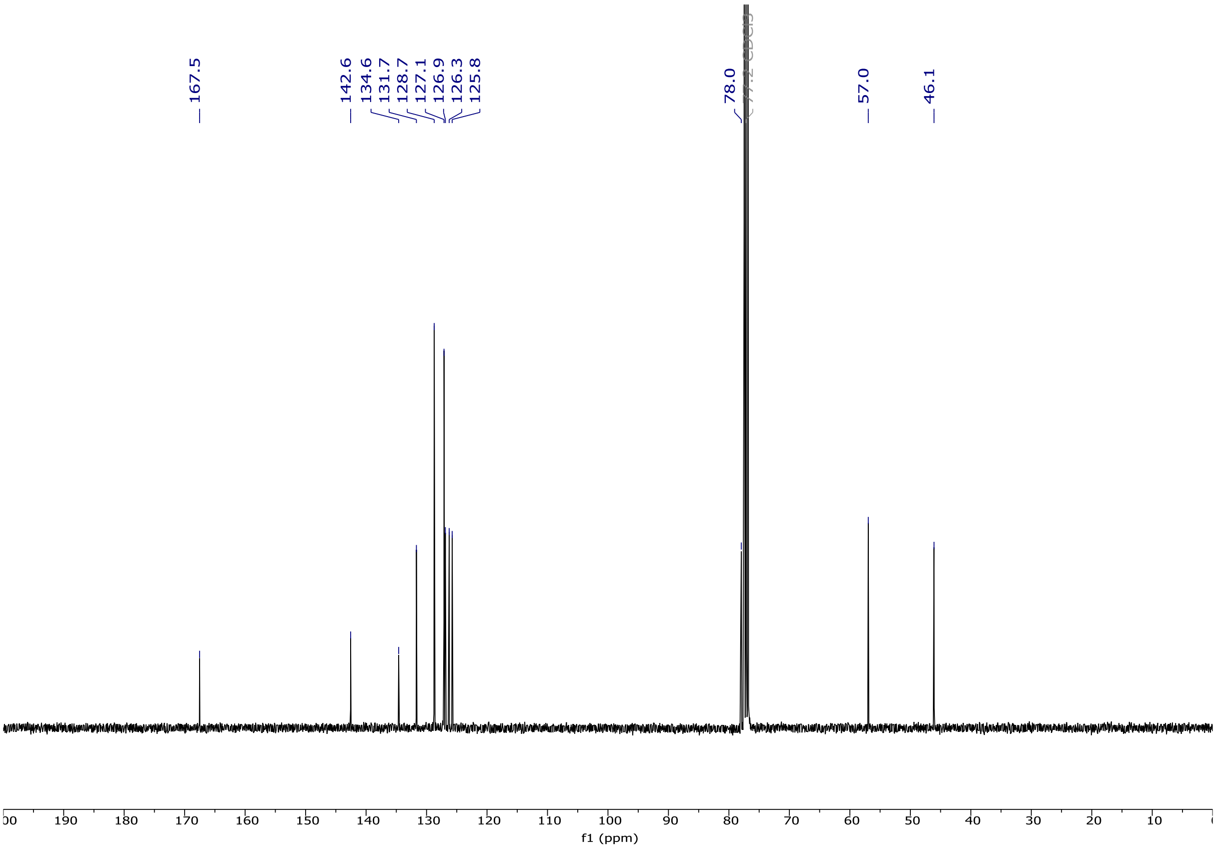

N-(2-Methoxy-2-phenylpropyl)benzamide (4k)

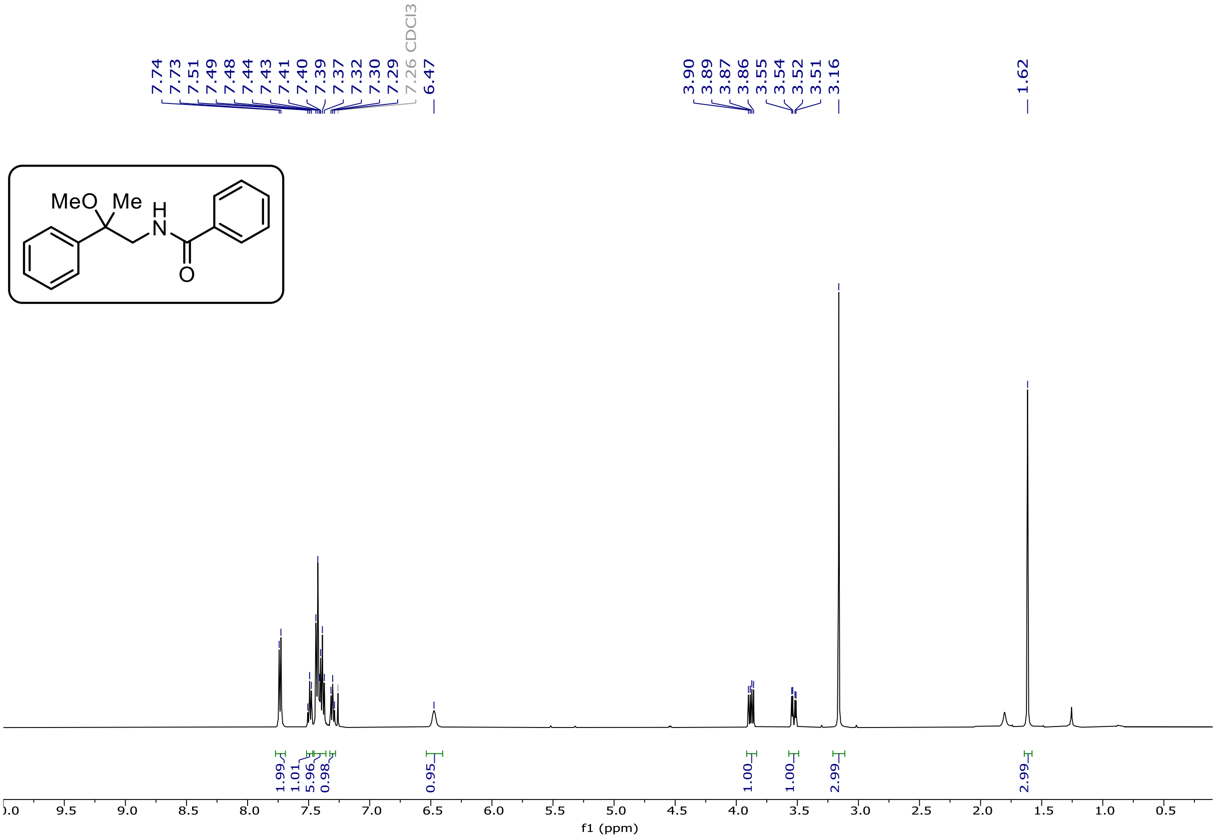

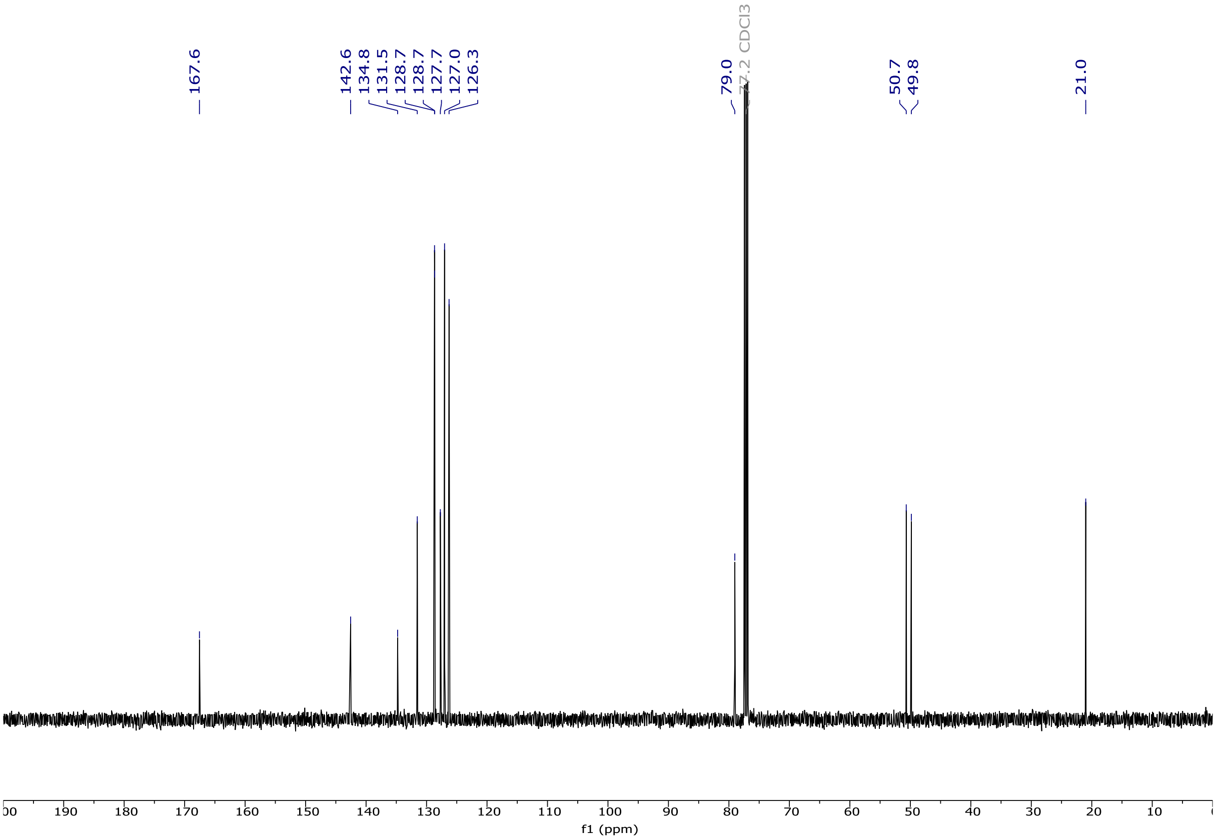

N-(2-Methoxy-2,2-diphenylethyl)benzamide (4l)

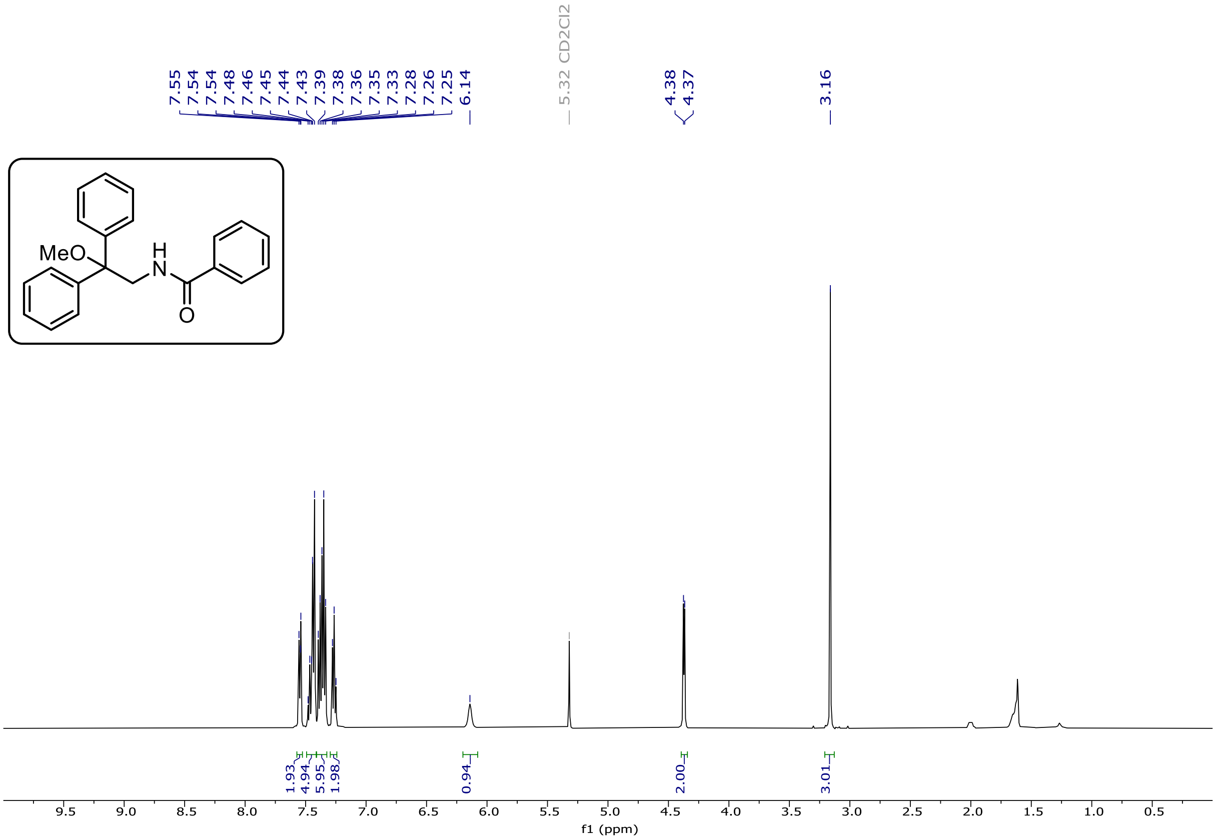

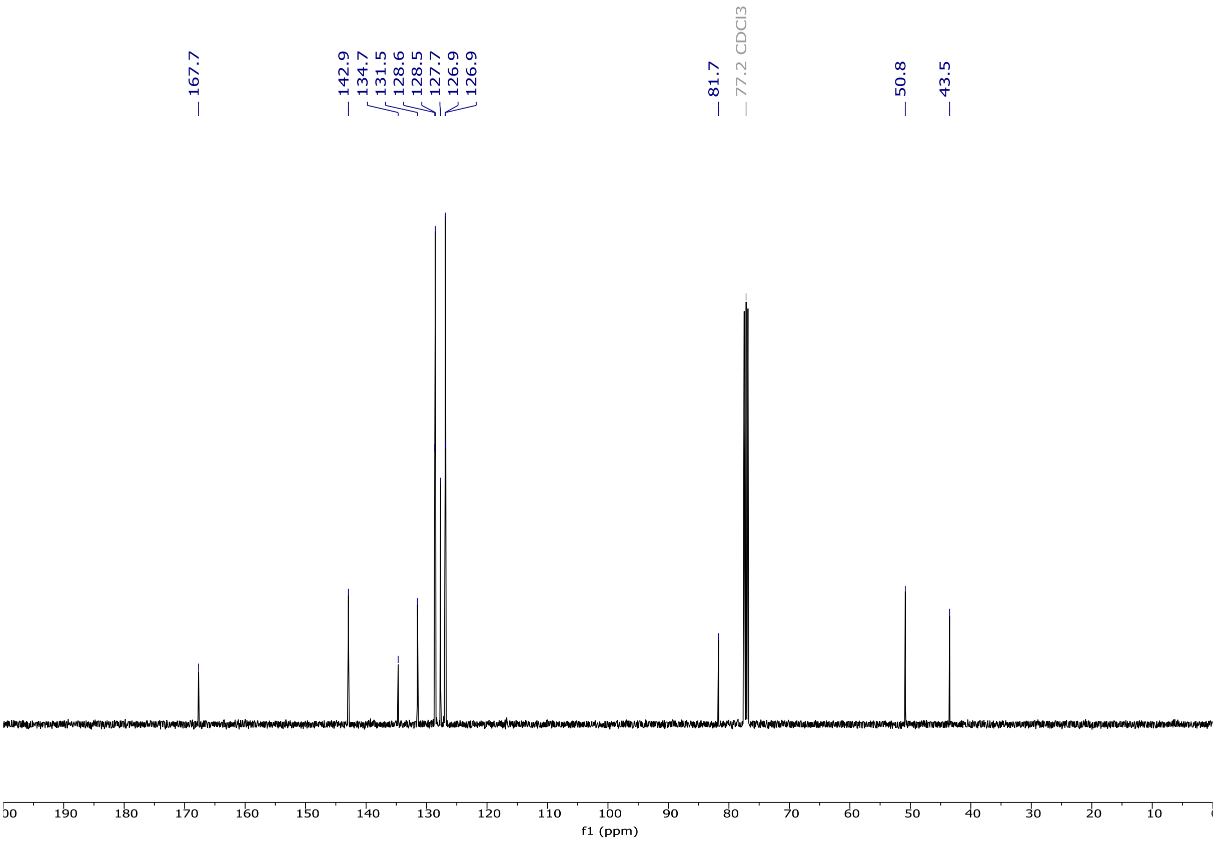

N-(1-Methoxy-1-phenylpropan-2-yl)benzamide (*erythro*-4m)

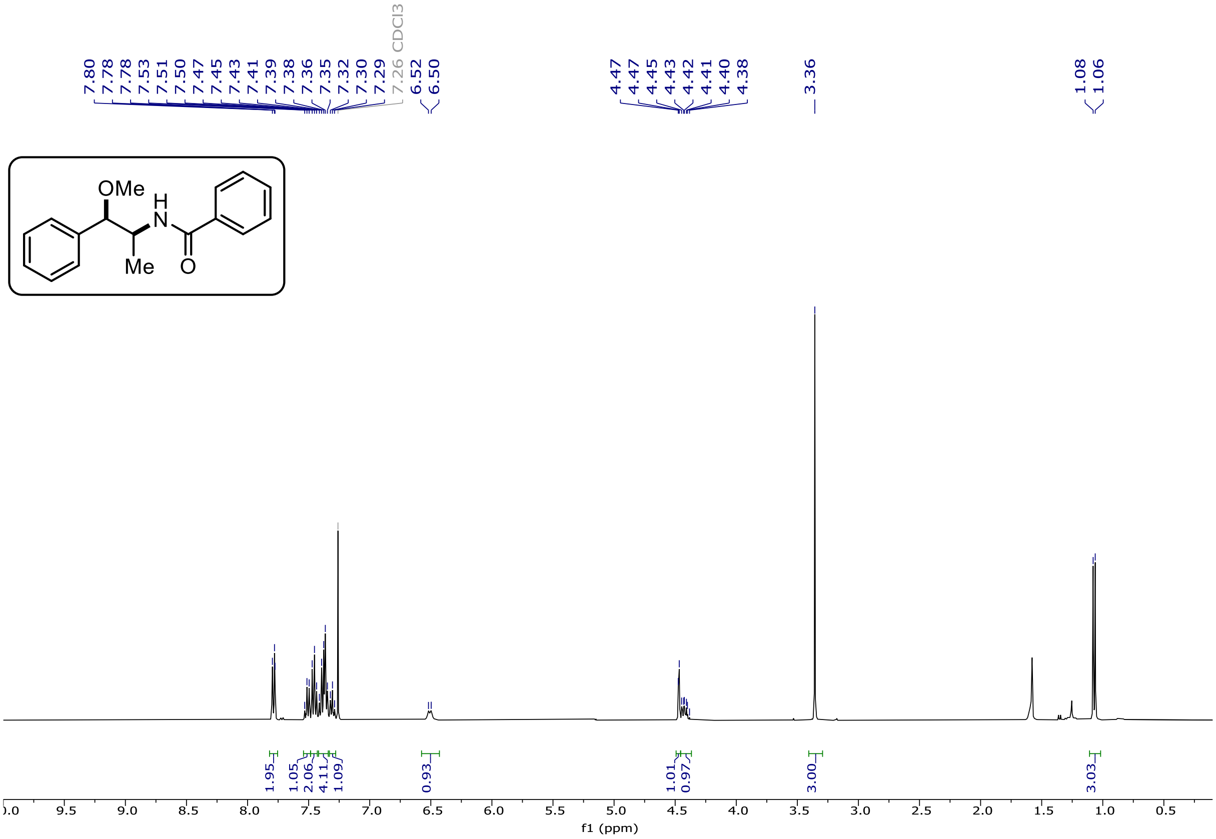

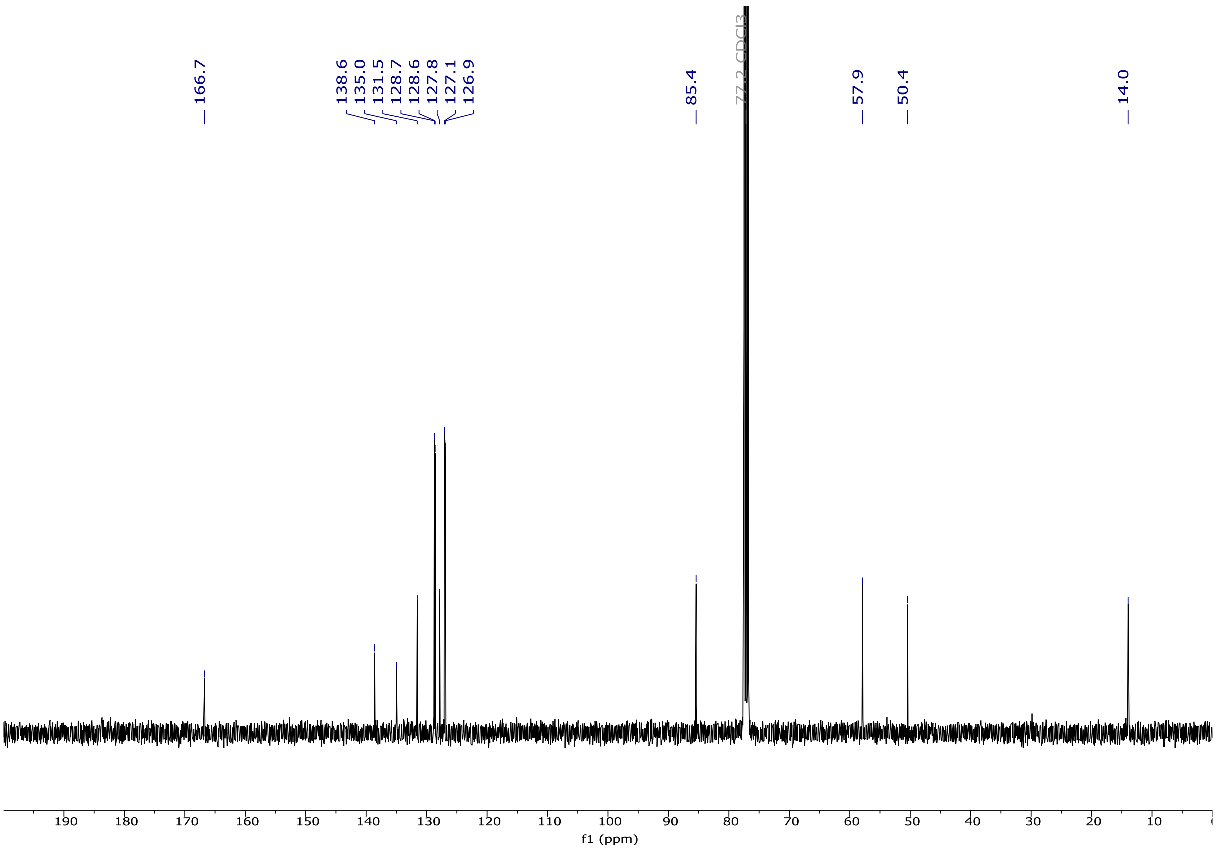

N-(2-Methoxy-1-phenylpropyl)benzamide (*threo*-4m')

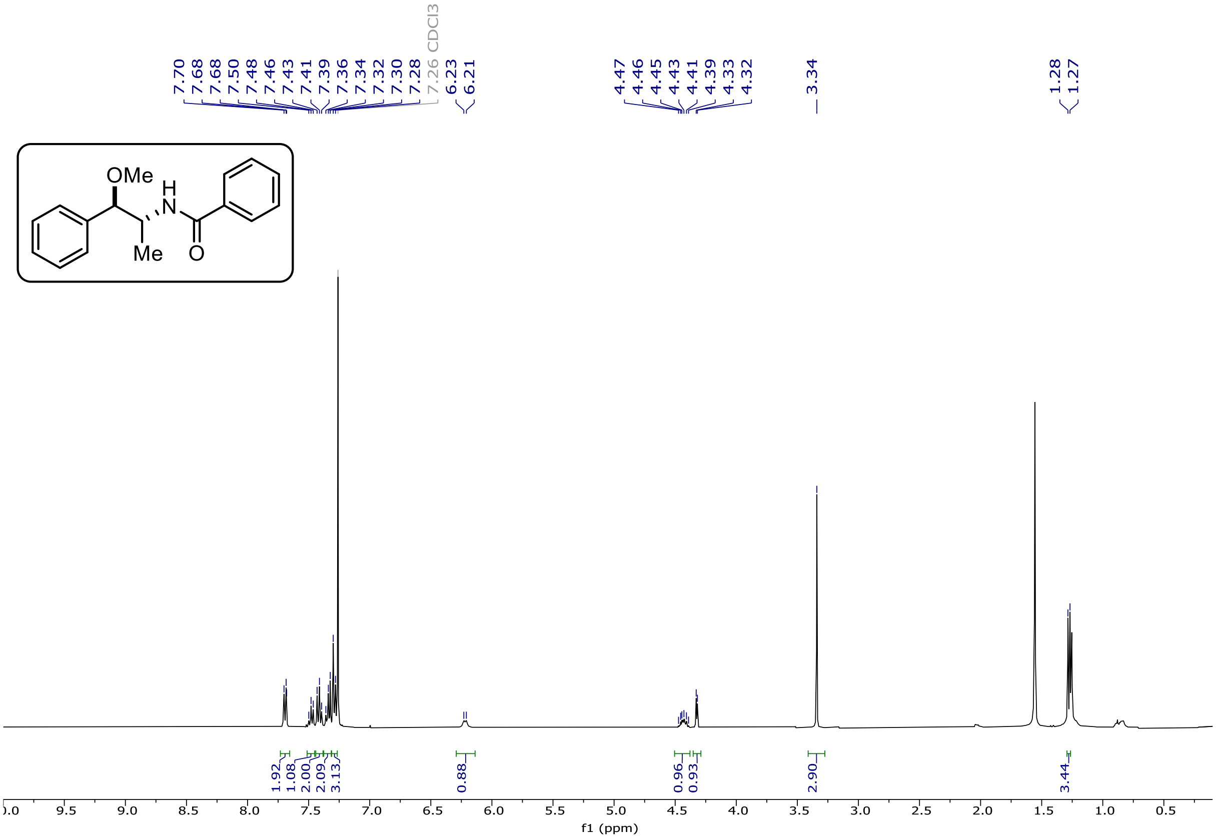

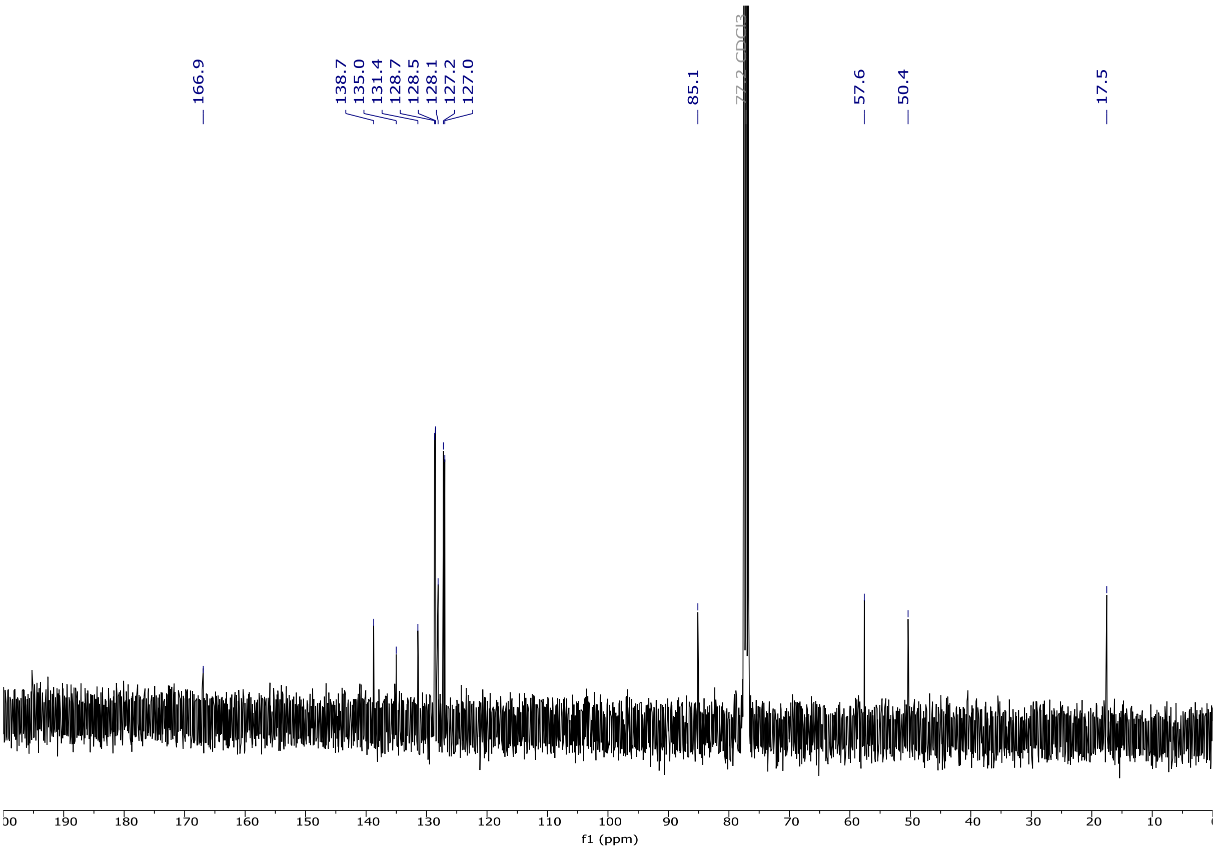

N-(2-Methoxy-2-phenylcyclohexyl)benzamide (4n + 4n')

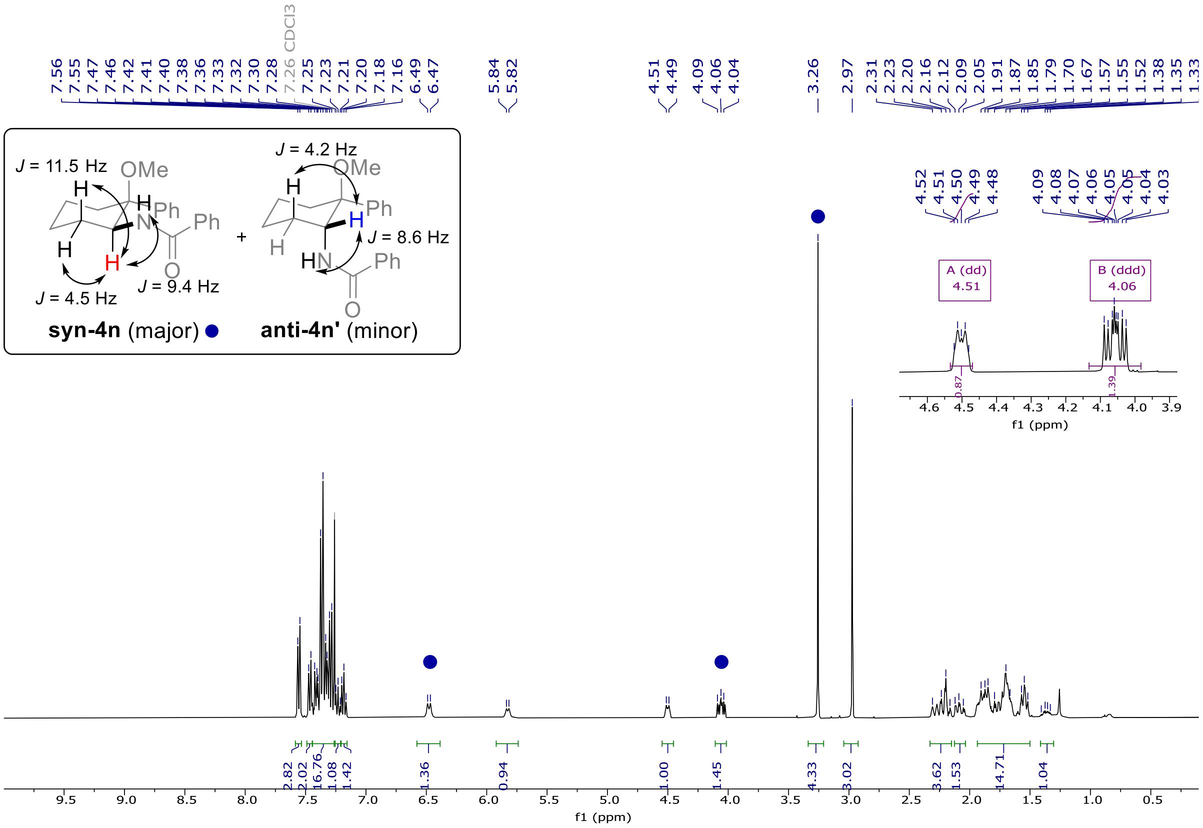

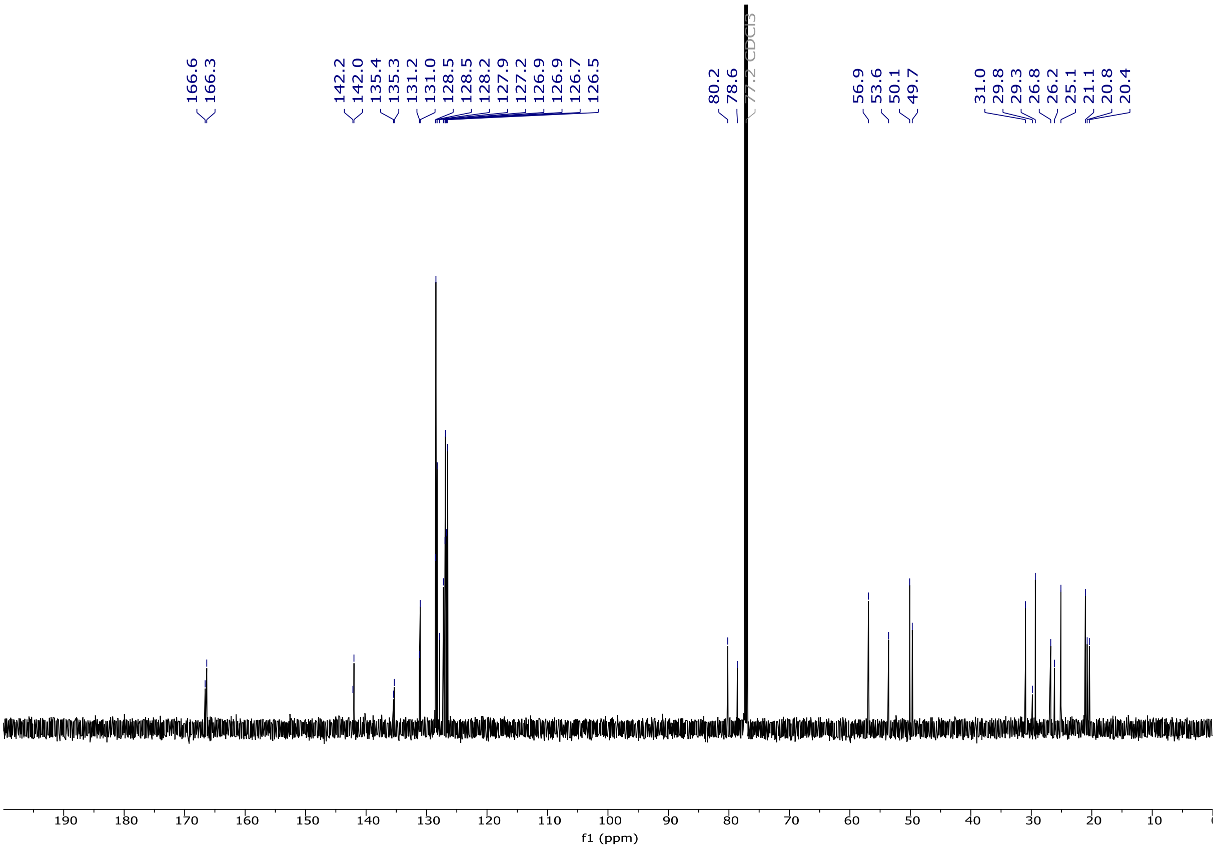

N-[2-Methoxy-2-{(8R,9S,13S,14S)-13-methyl-17-oxo-7,8,9,11,12,13,14,15,16,17-decahydro-6H-cyclopenta[a]phenanthren-3-yl}ethyl]benzamide (4o)

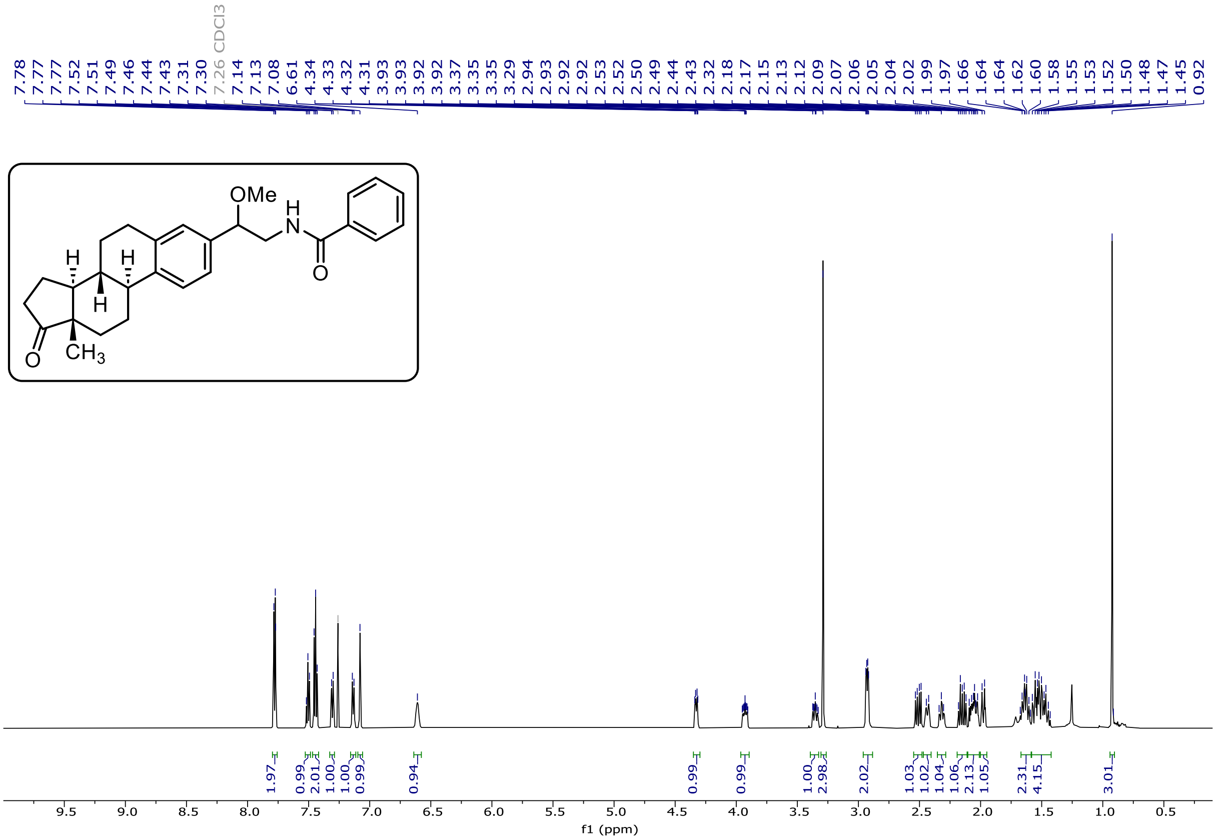

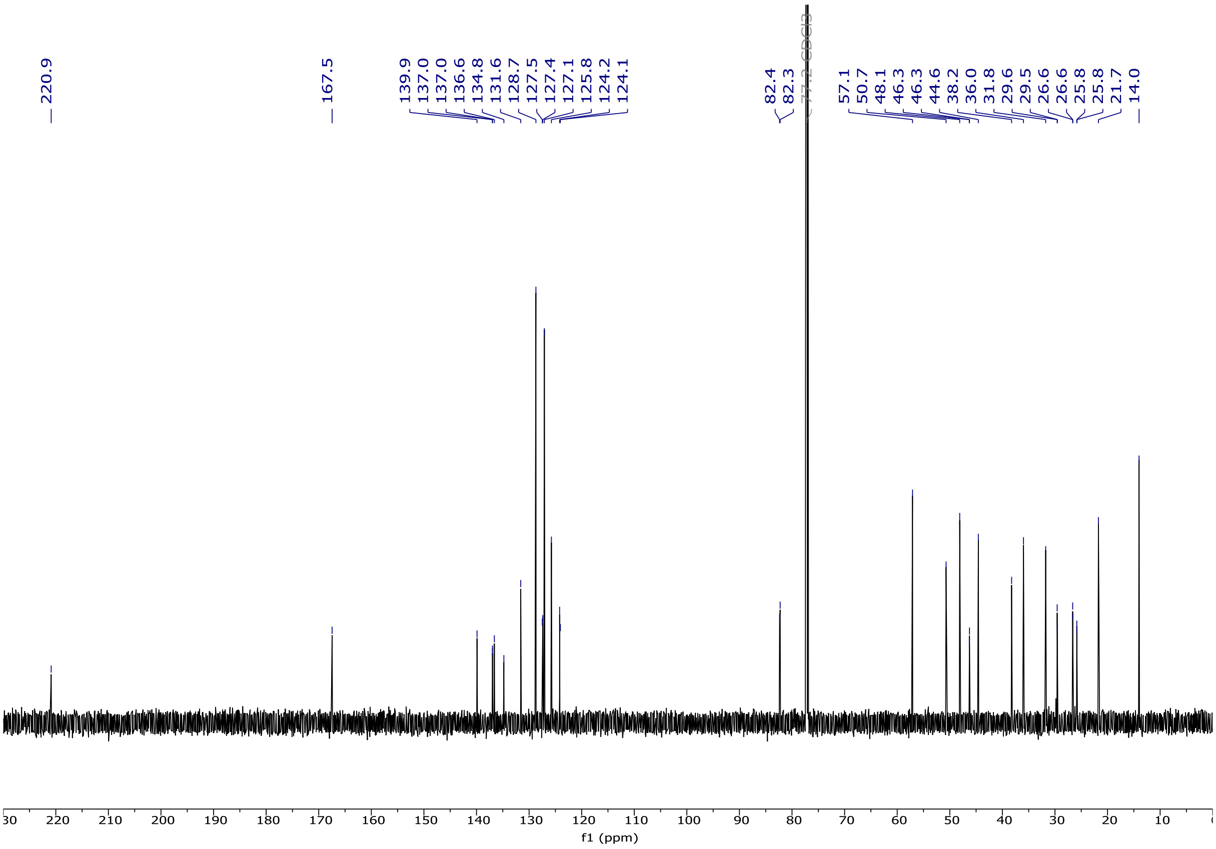

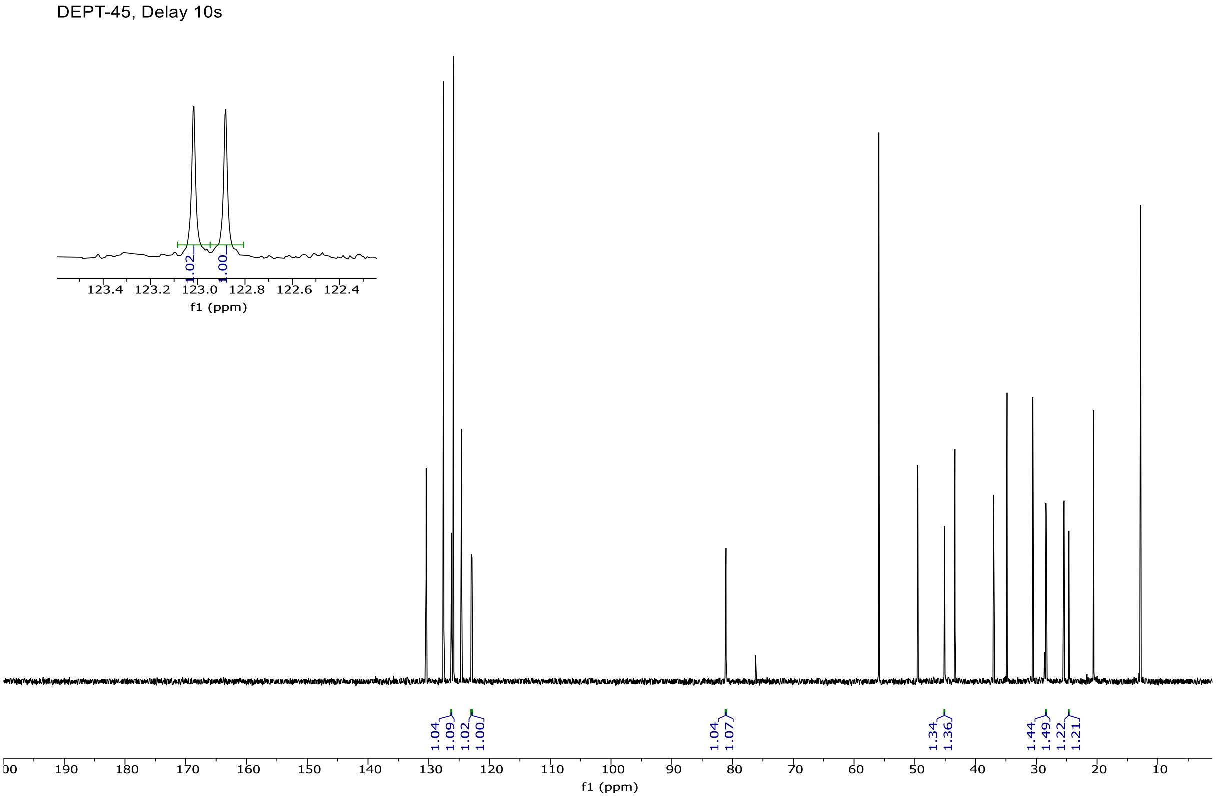

DEPT-45, Delay 10s
1.02
1.00
123.4 123.2 123.0 122.8 122.6 122.4
f1 (ppm)
1.04
1.09
1.02
1.00
1.04
1.07
1.34
1.36
1.44
1.49
1.22
1.21
f1 (ppm)

Methyl (2*S*)-3-{4-(2-benzamido-1-methoxyethyl)phenyl}-2-{(*tert*-butoxycarbonyl)amino} propanoate (4p)

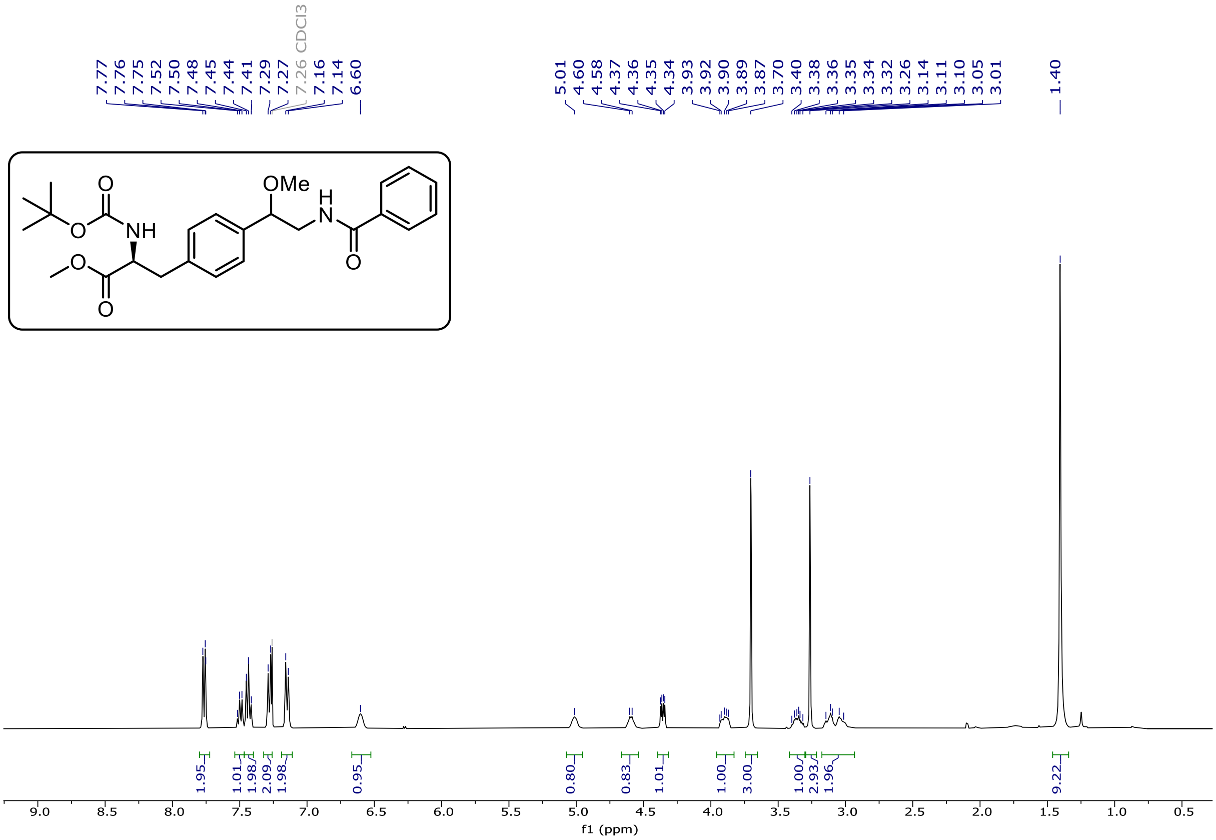

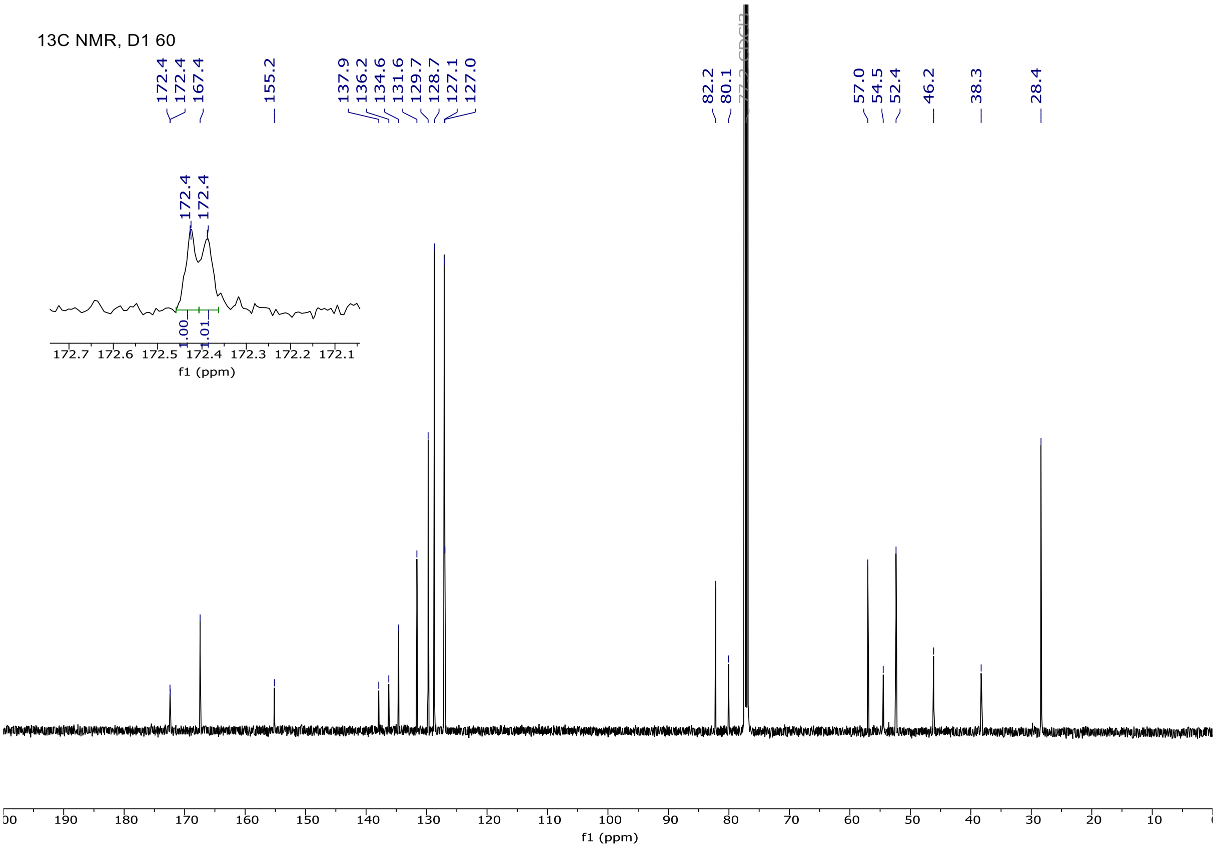

N-{2-Methoxy-2-(2-oxopyrrolidin-1-yl)ethyl}benzamide (4q)

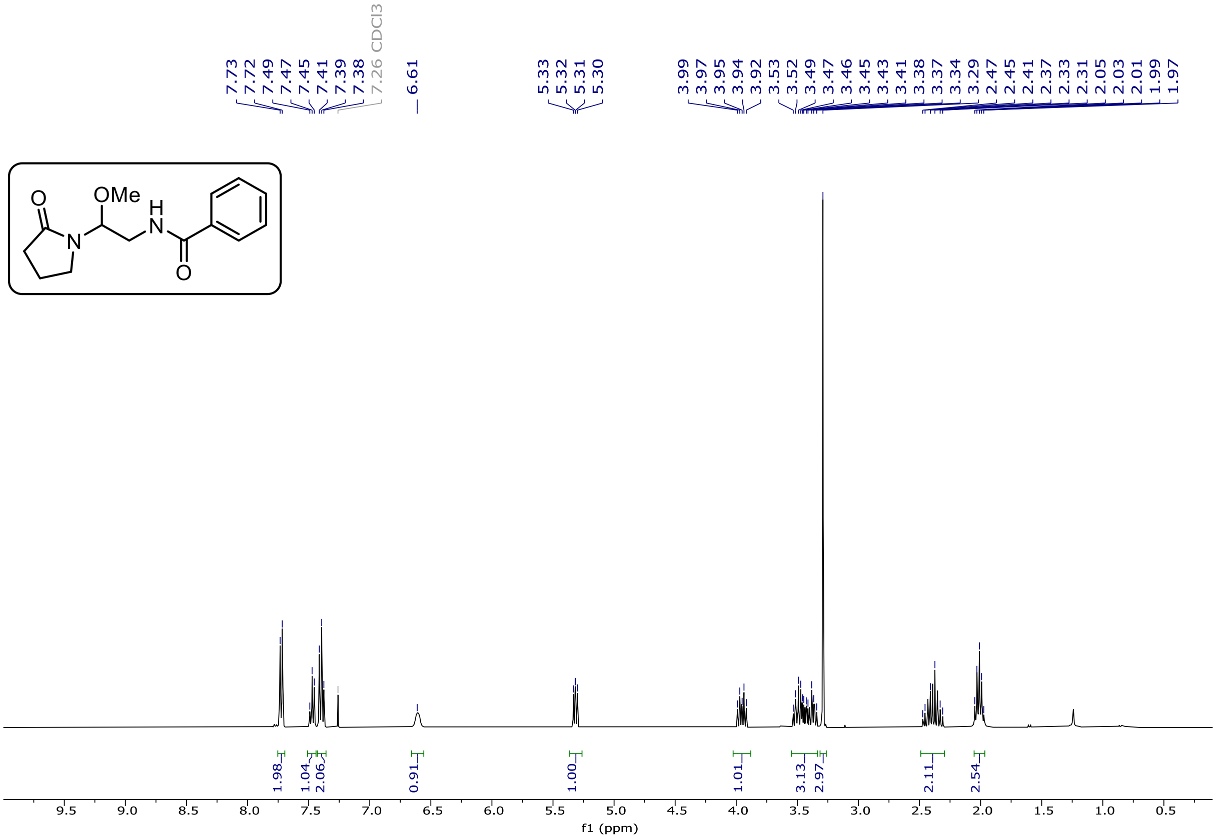

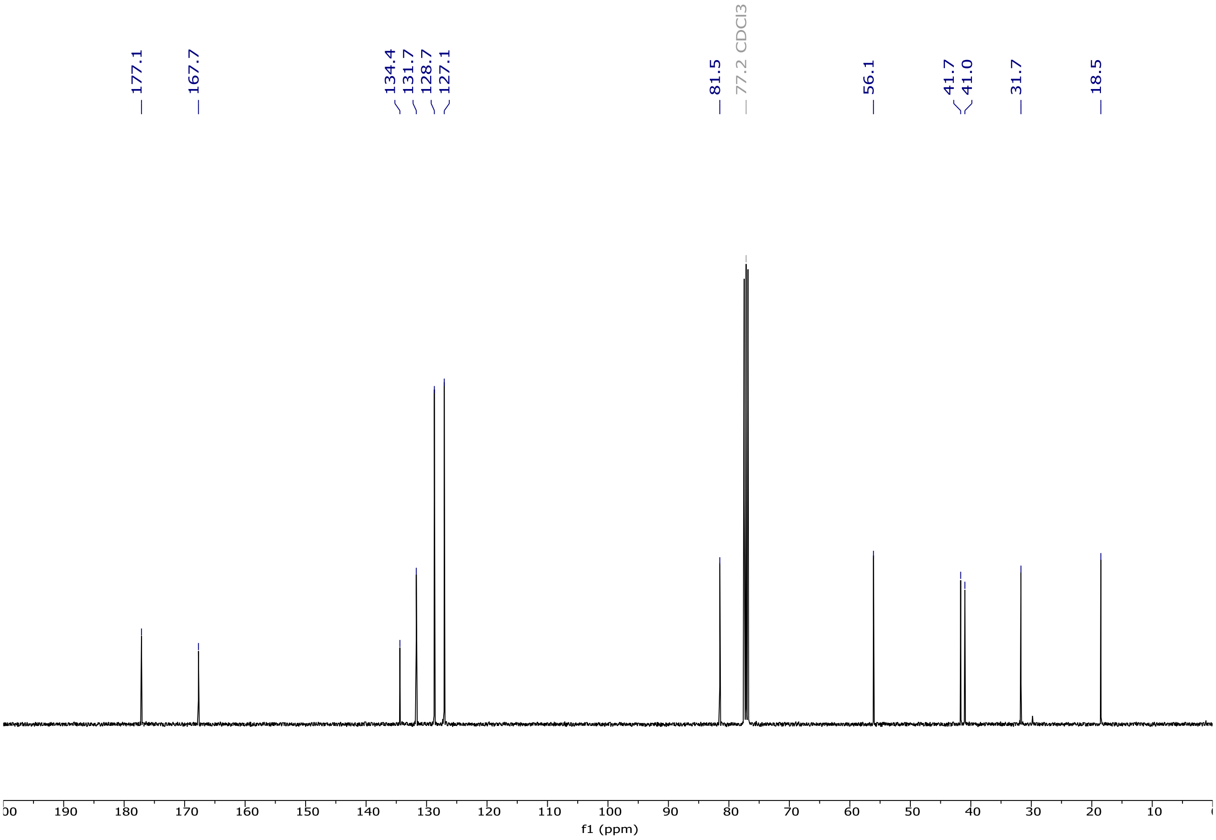

N-{2-Methoxy-2-(2-oxoazepan-1-yl)ethyl}benzamide (4r)

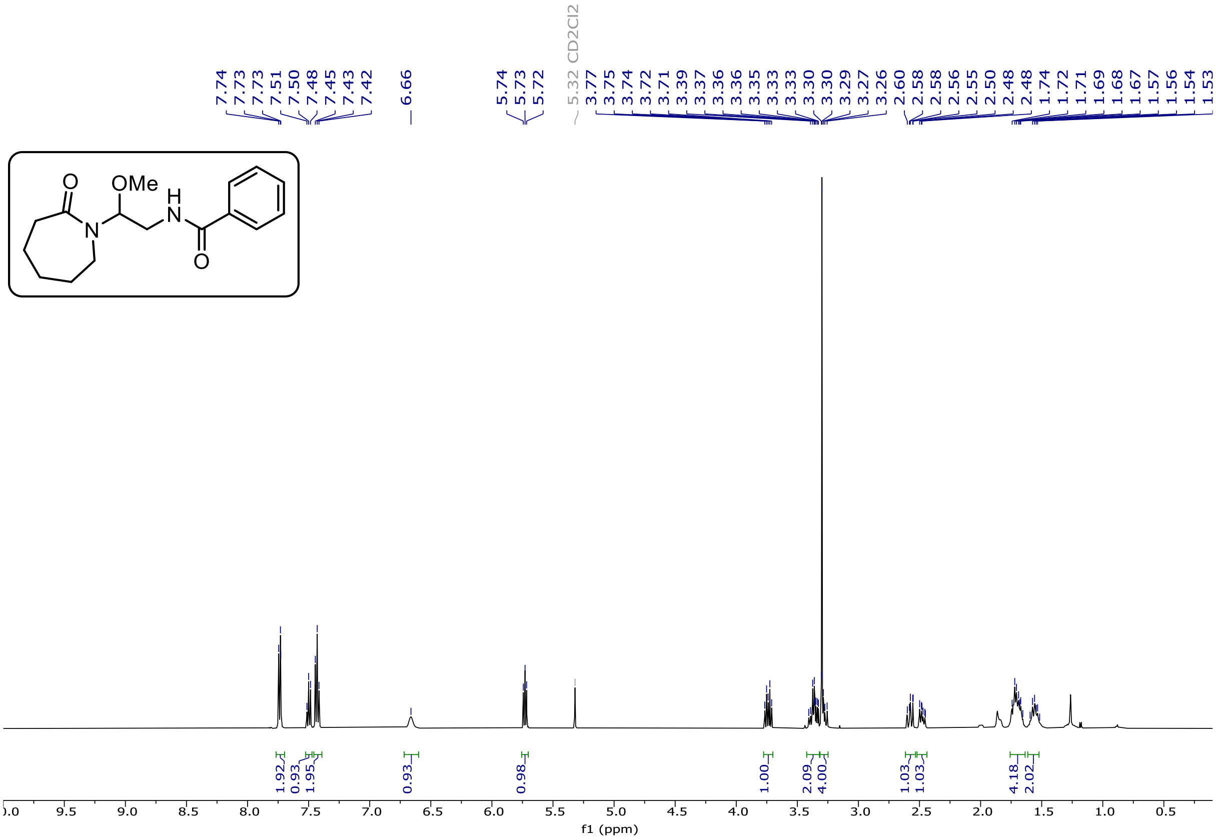

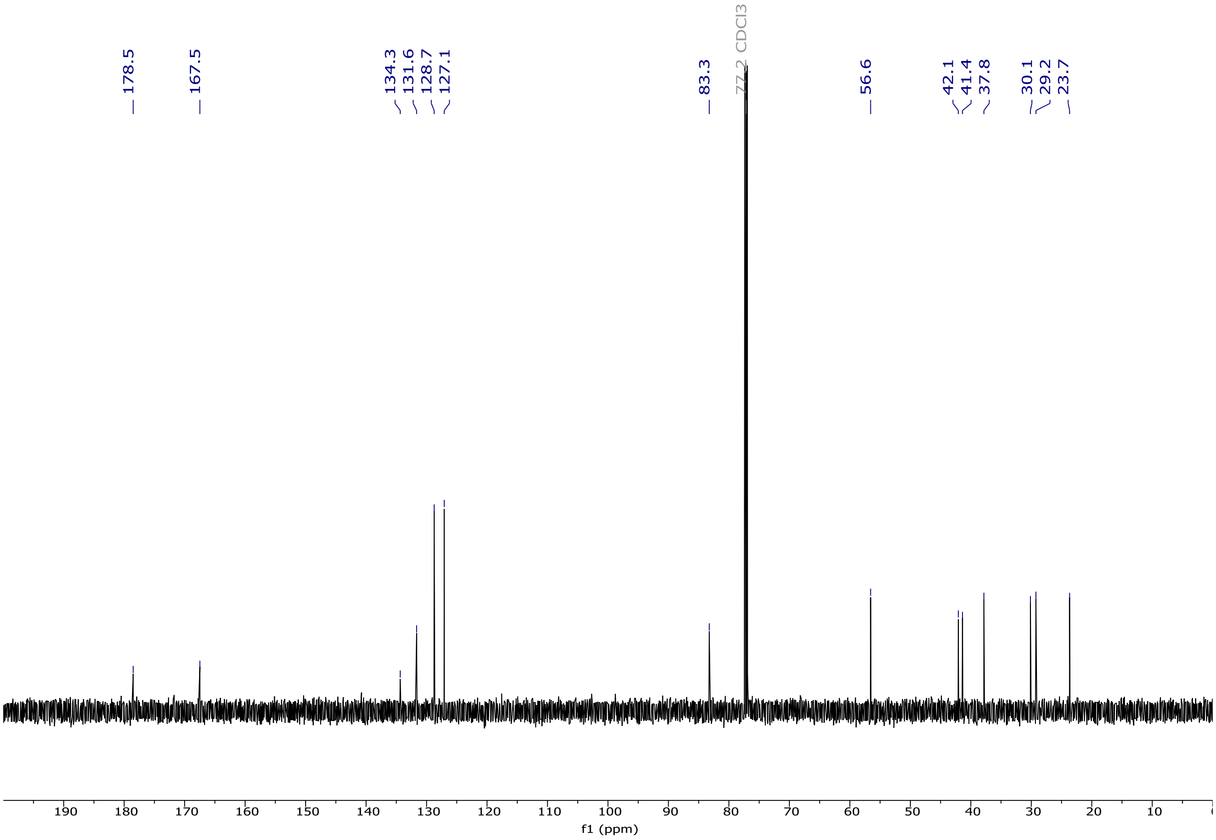

N-{1-Methoxy-1-(2-oxopyrrolidin-1-yl)propan-2-yl}benzamide (*threo*-4s)

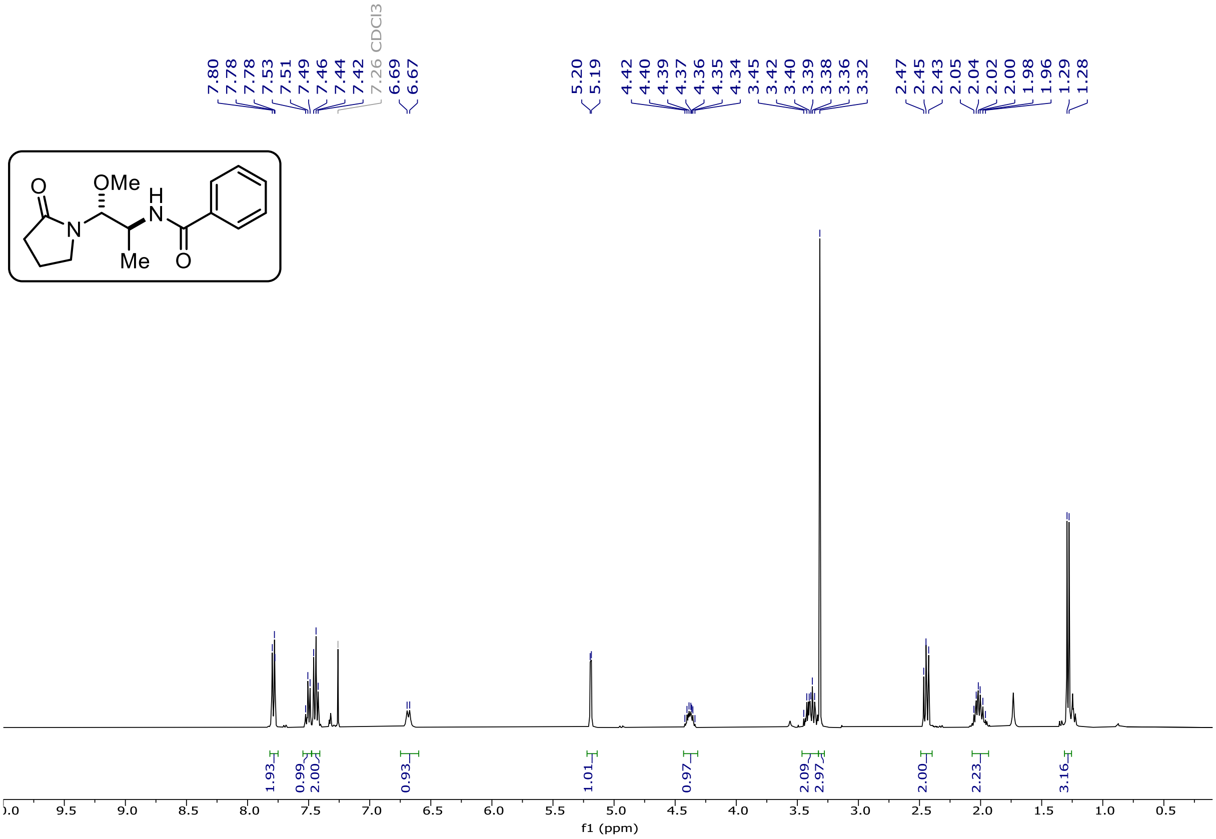

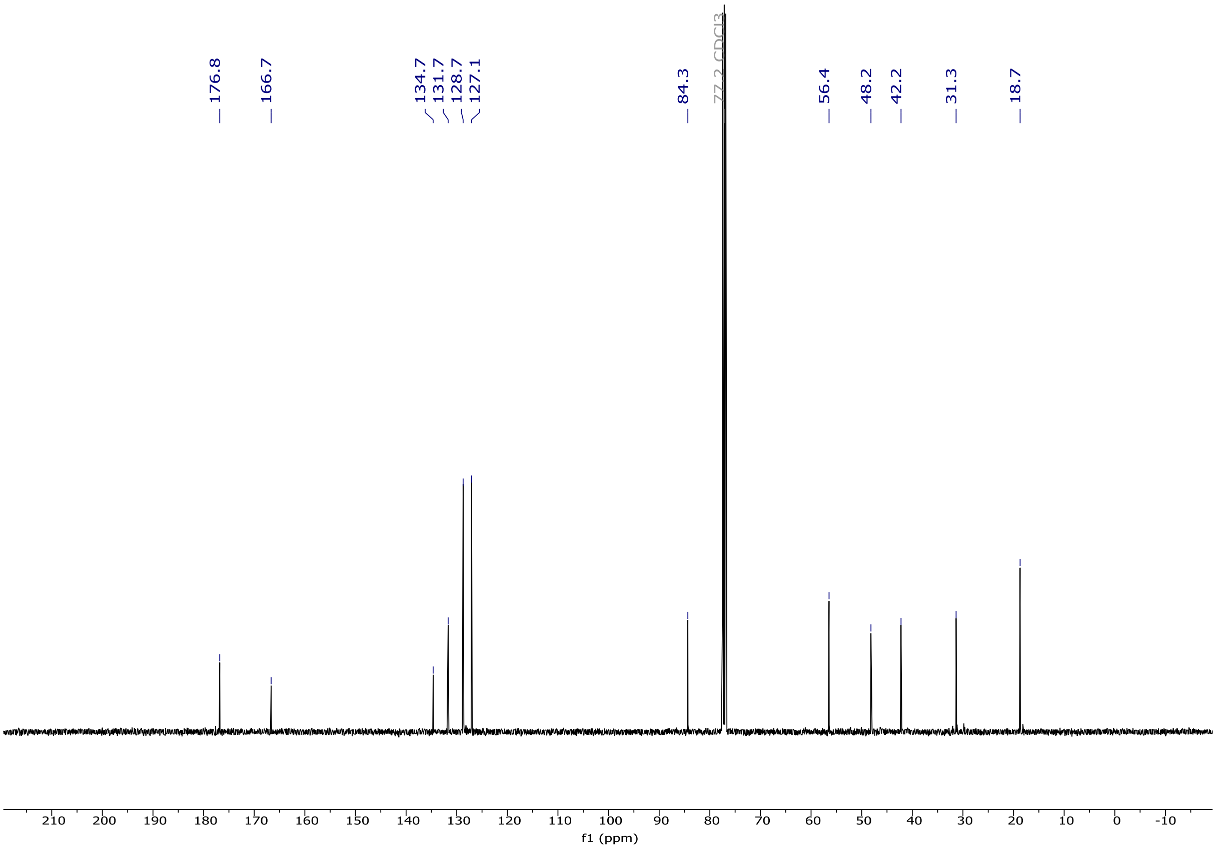

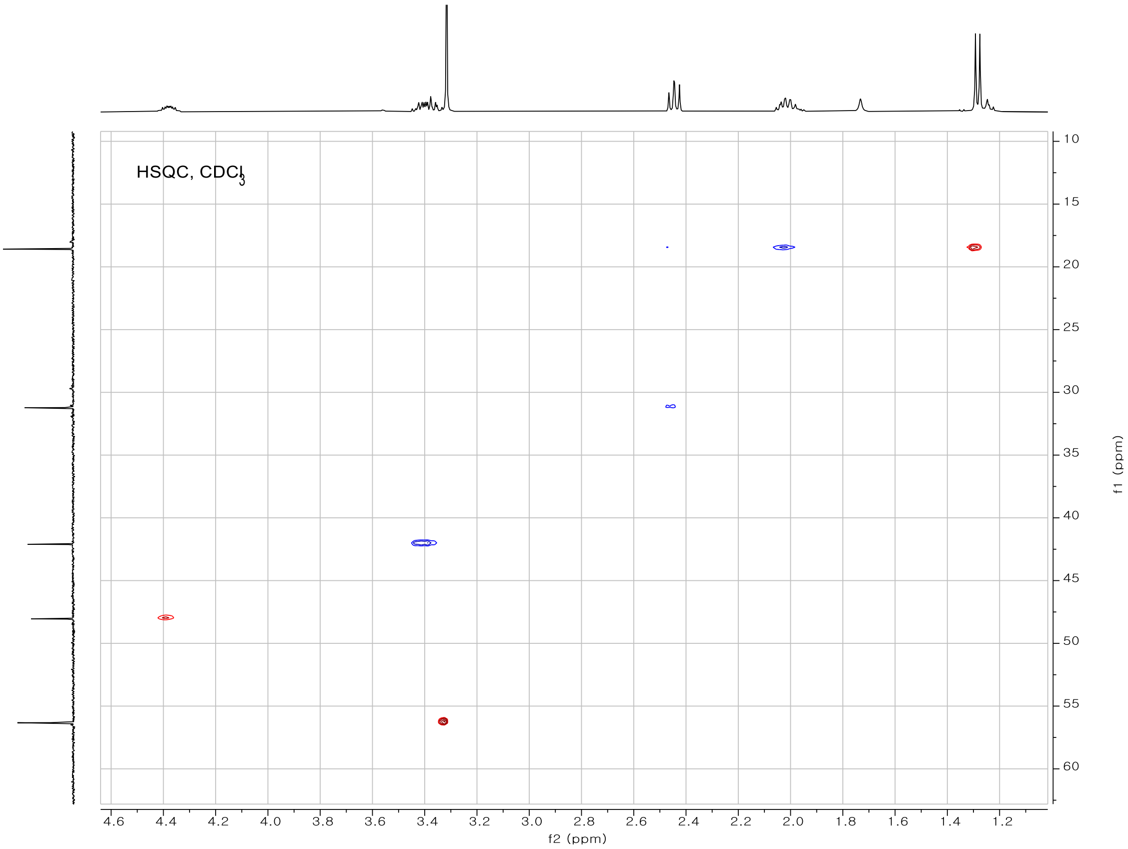
HSQC, CDCl3
f2 (ppm)
f1 (ppm)
4.6
4.4
4.2
4.0
3.8
3.6
3.4
3.2
3.0
2.8
2.6
2.4
2.2
2.0
1.8
1.6
1.4
1.2
10
15
20
25
30
35
40
45
50
55
60

_N_-(1-methoxy-1-(2-oxopyrrolidin-1-yl)propan-2-yl)benzamide (_erythro_-4s')

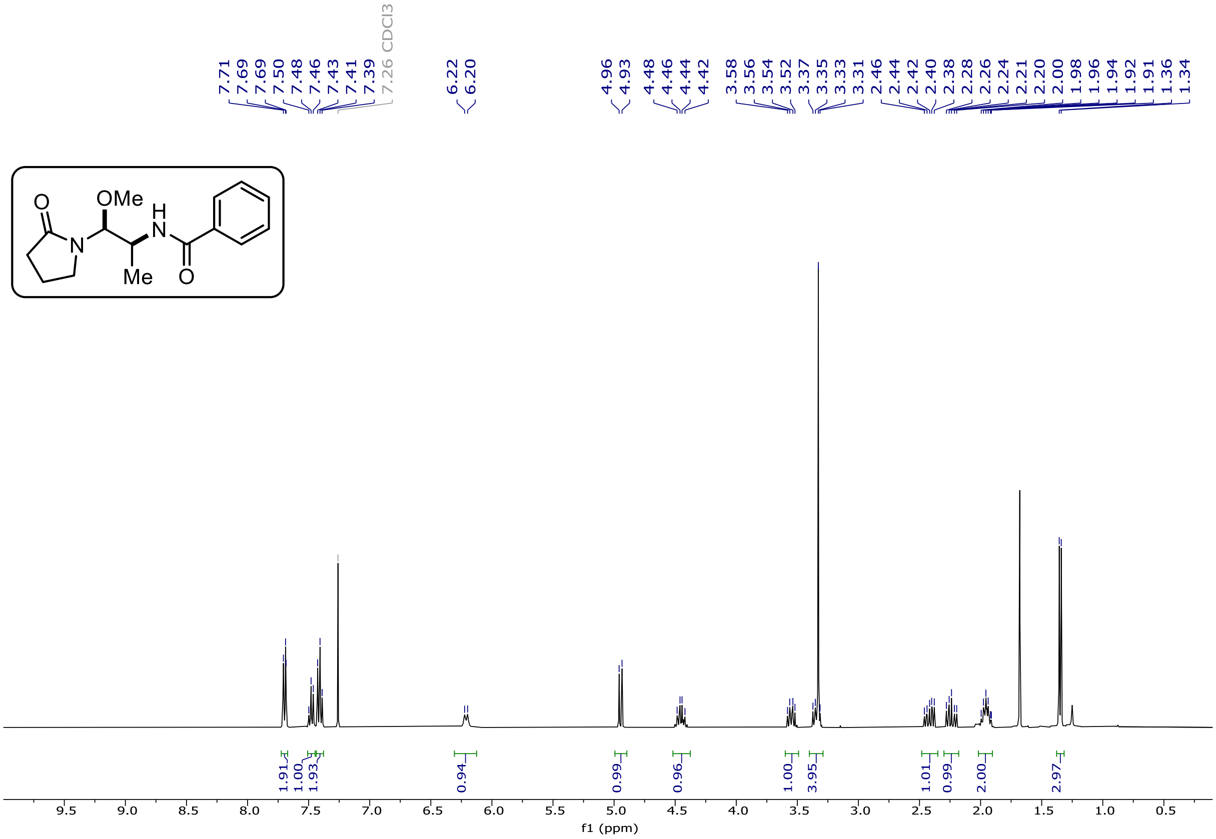

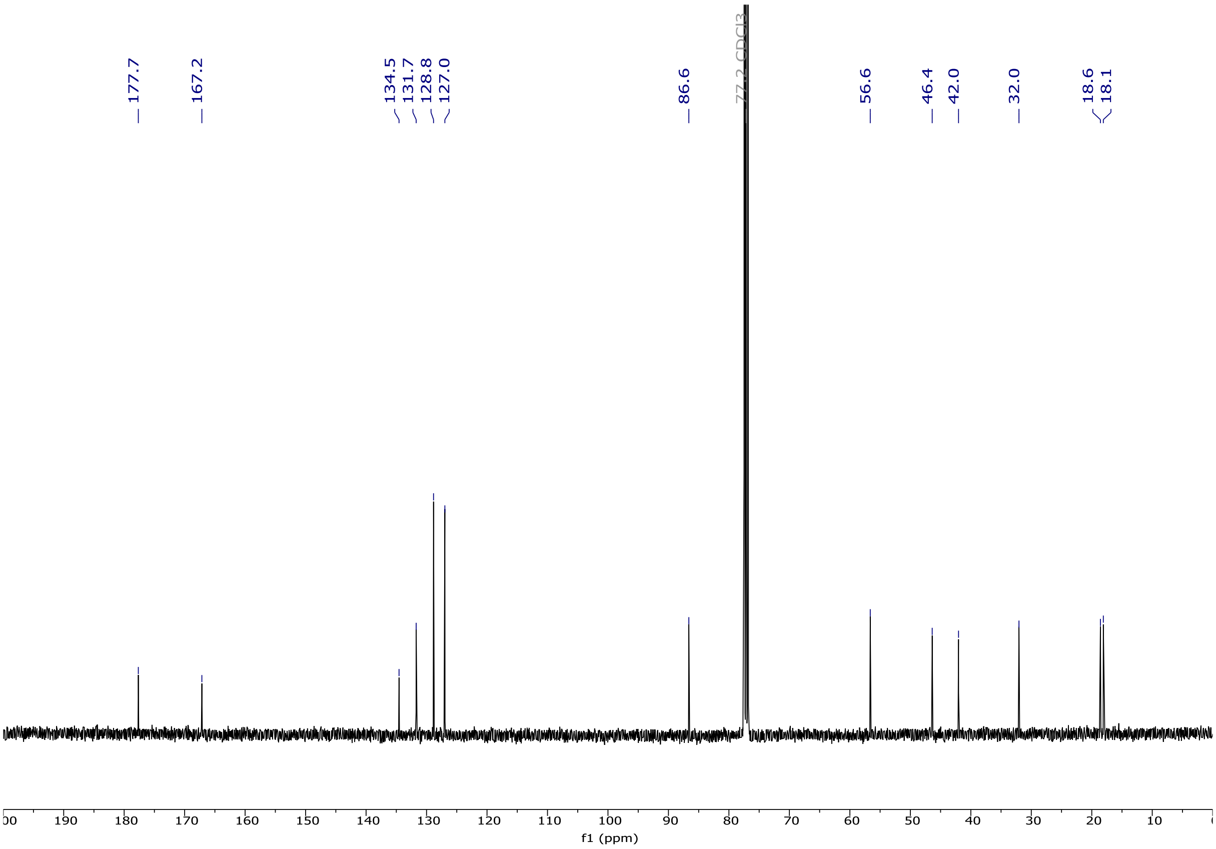

N-{2-(Ethylthio)-2-methoxyethyl}benzamide (4t)

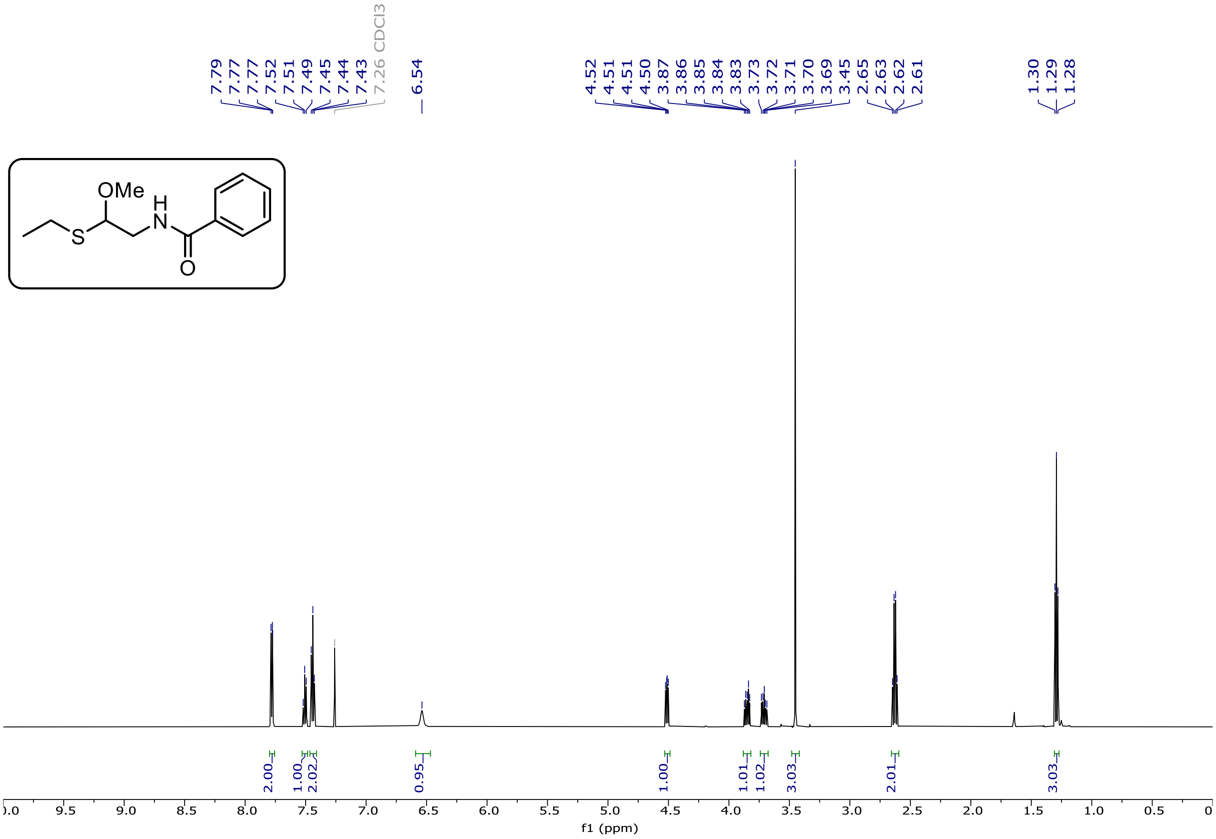

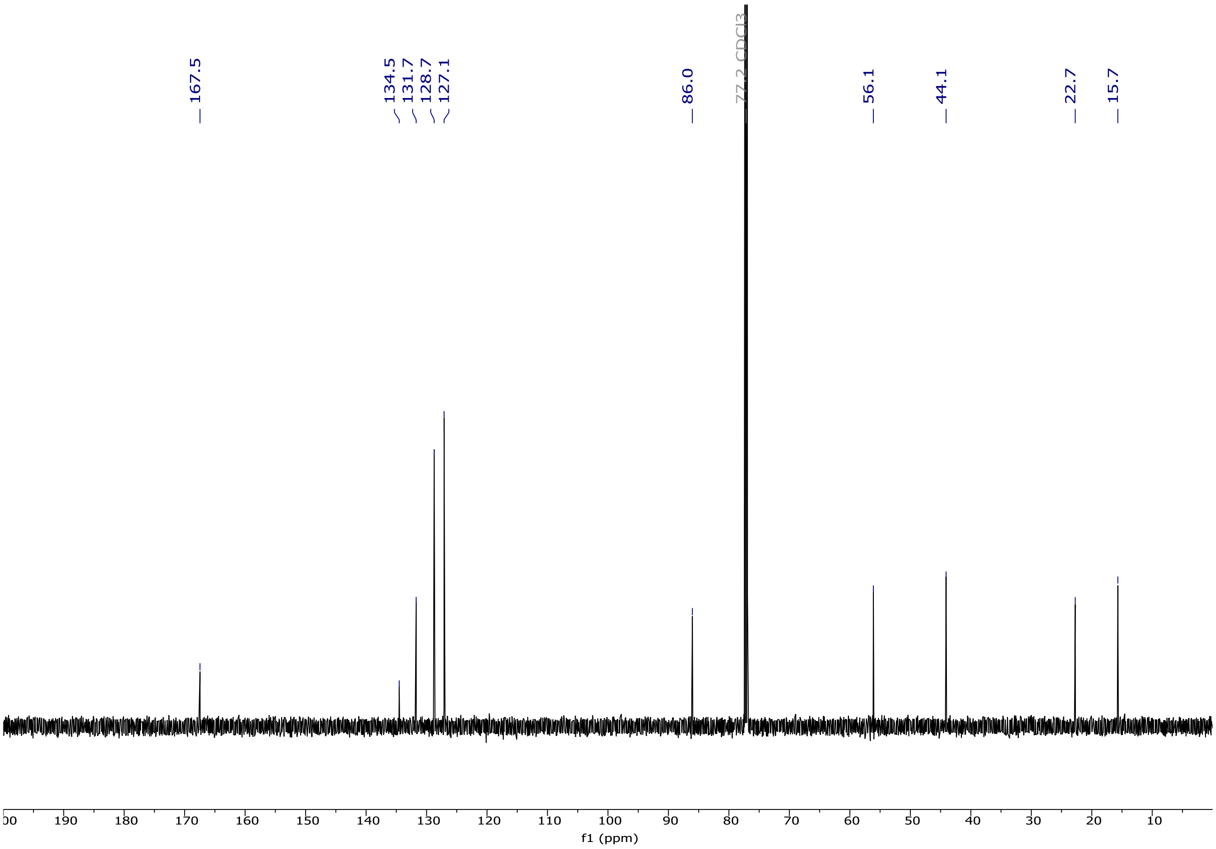

***N*-{2-Methoxy-2-(phenylthio)ethyl}benzamide (4u)**

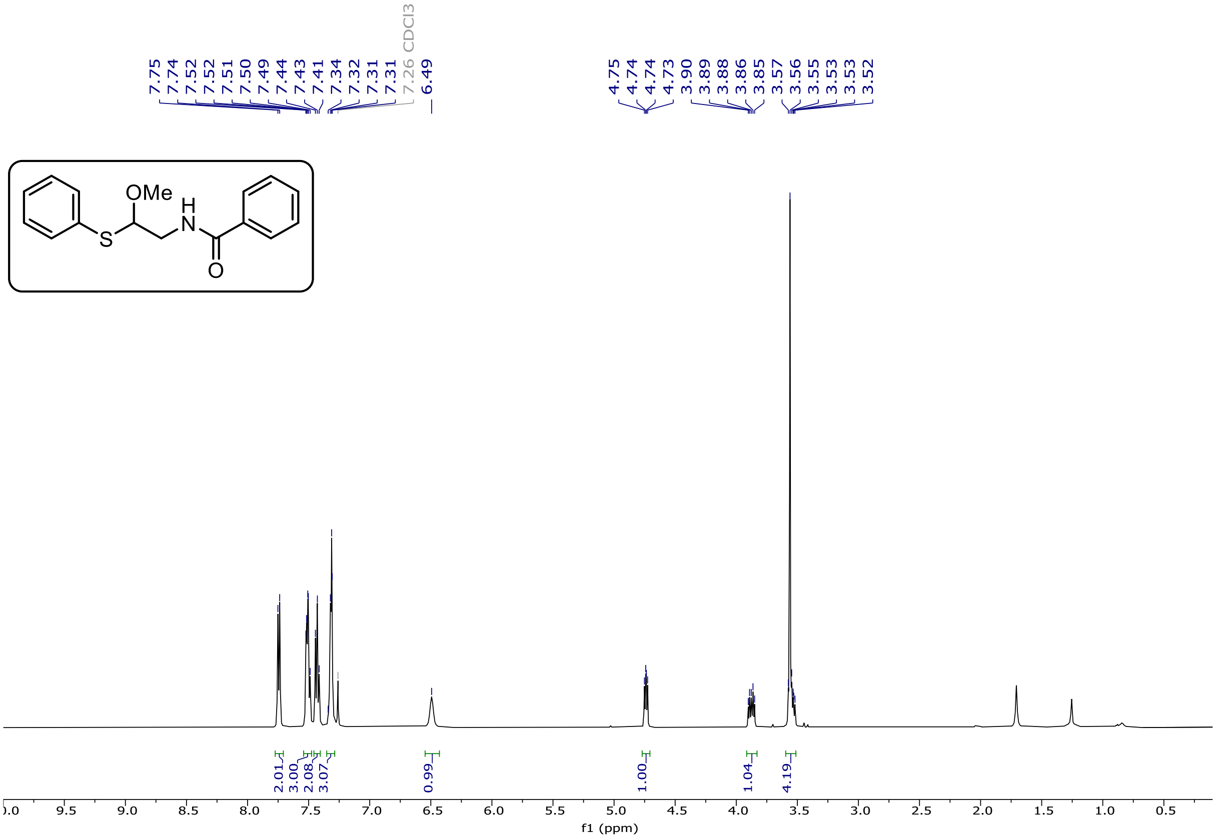

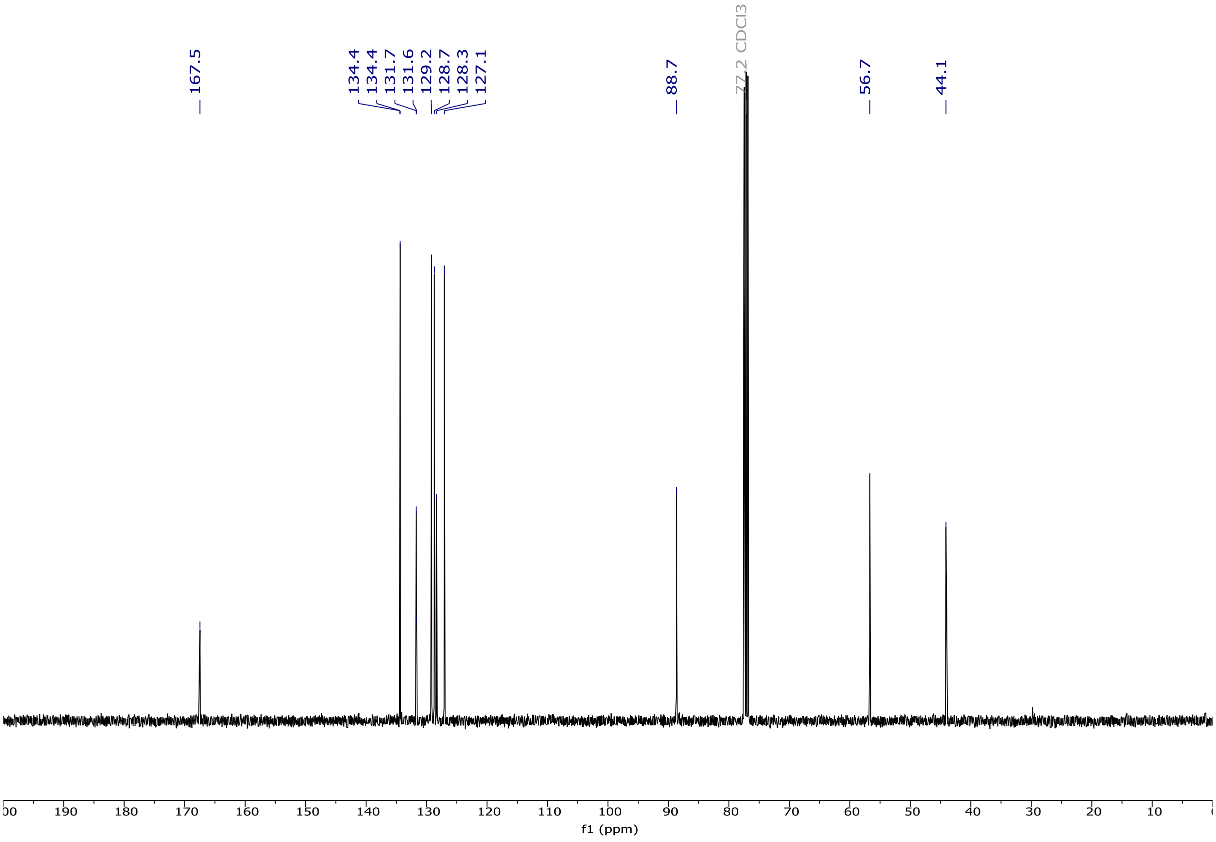

N-(2-Ethoxy-2-methoxyethyl)benzamide (4v)

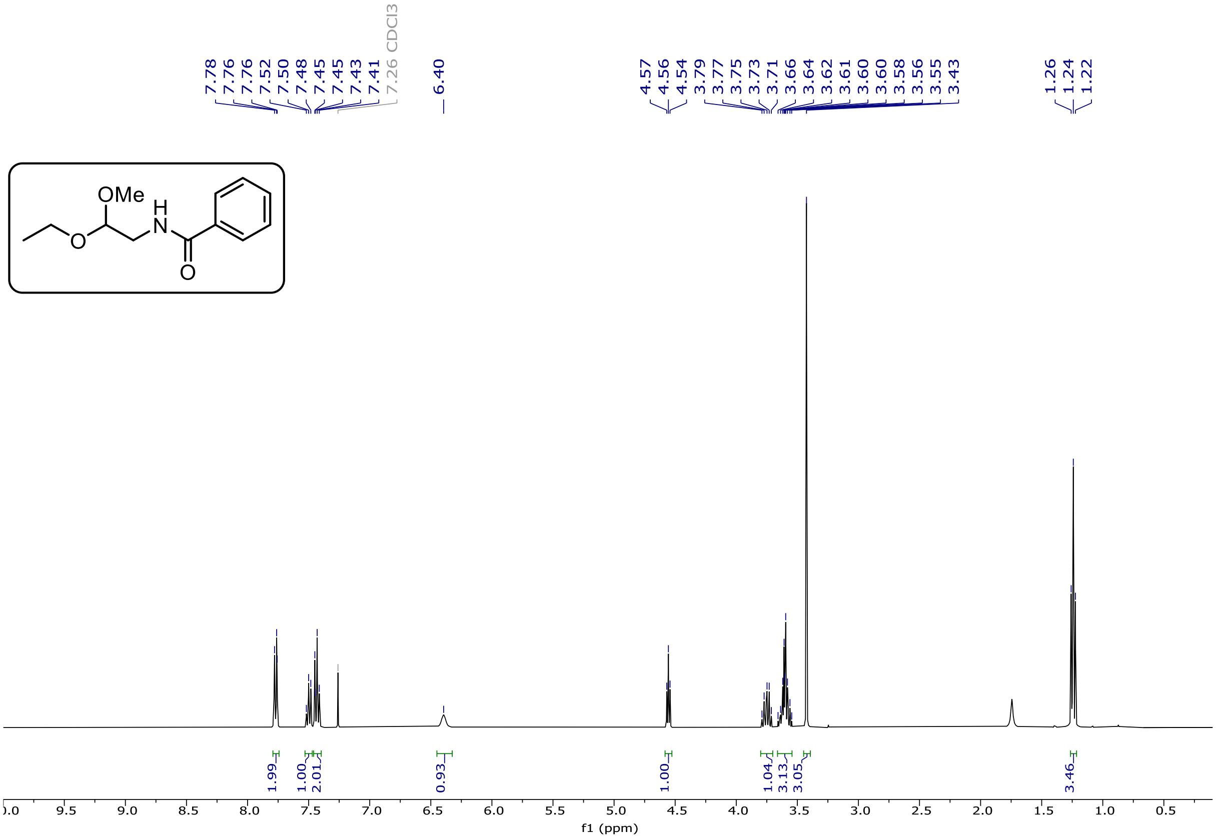

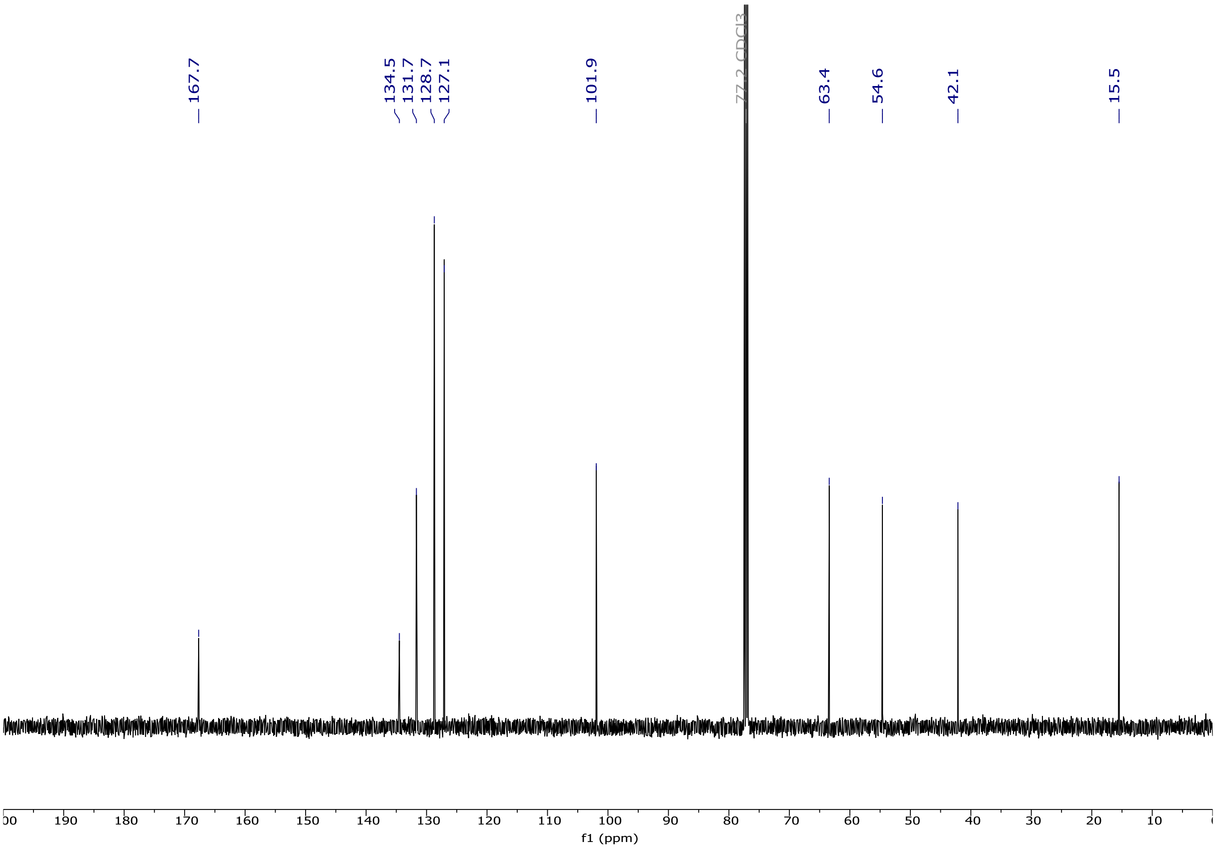

***N*-{2-(Cyclohexyloxy)-2-methoxyethyl}benzamide (4w)**

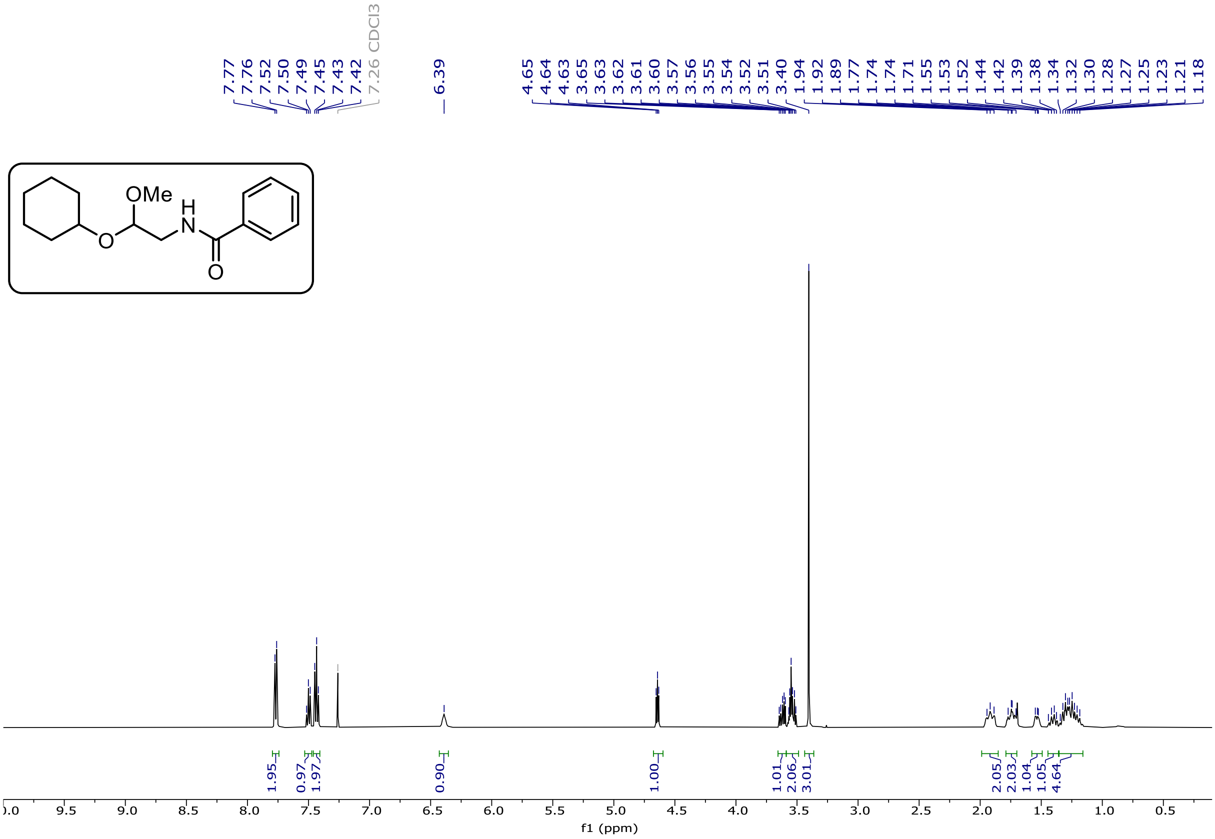

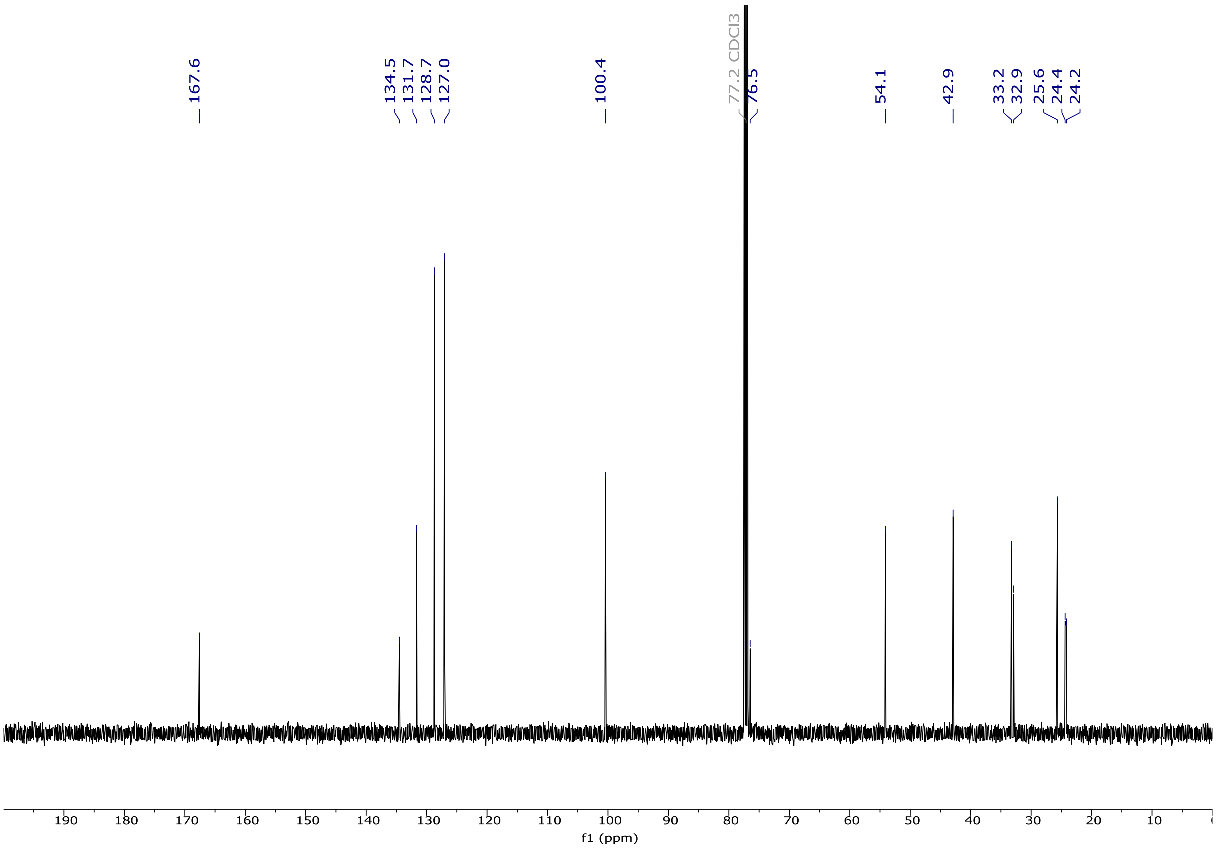

***N*-(2-Methoxy-2-phenoxyethyl)benzamide (4x)**

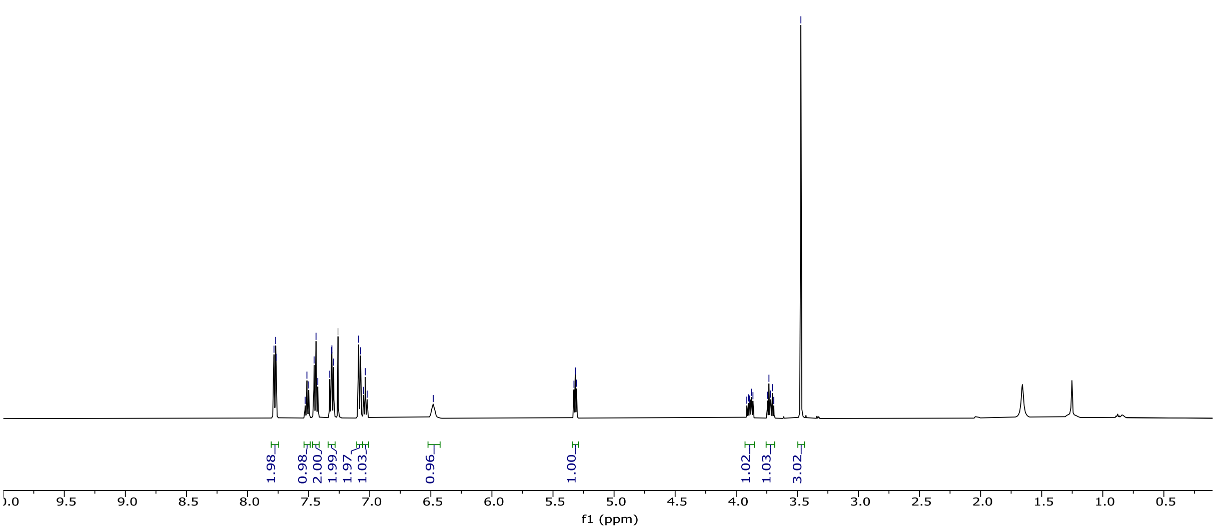

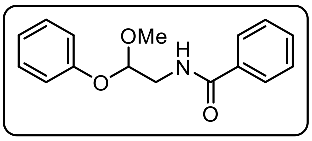

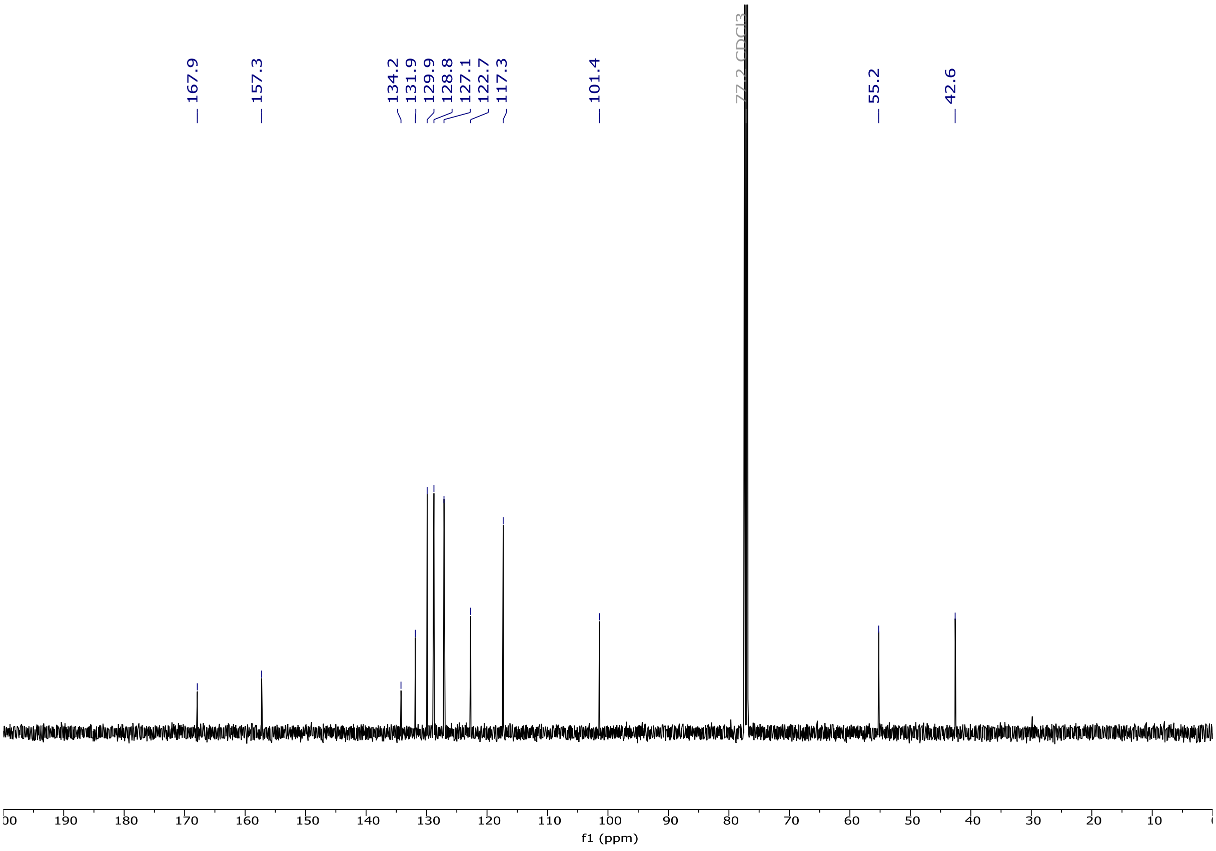

N-(2-Methoxytetrahydrofuran-3-yl)benzamide (4y+4y')

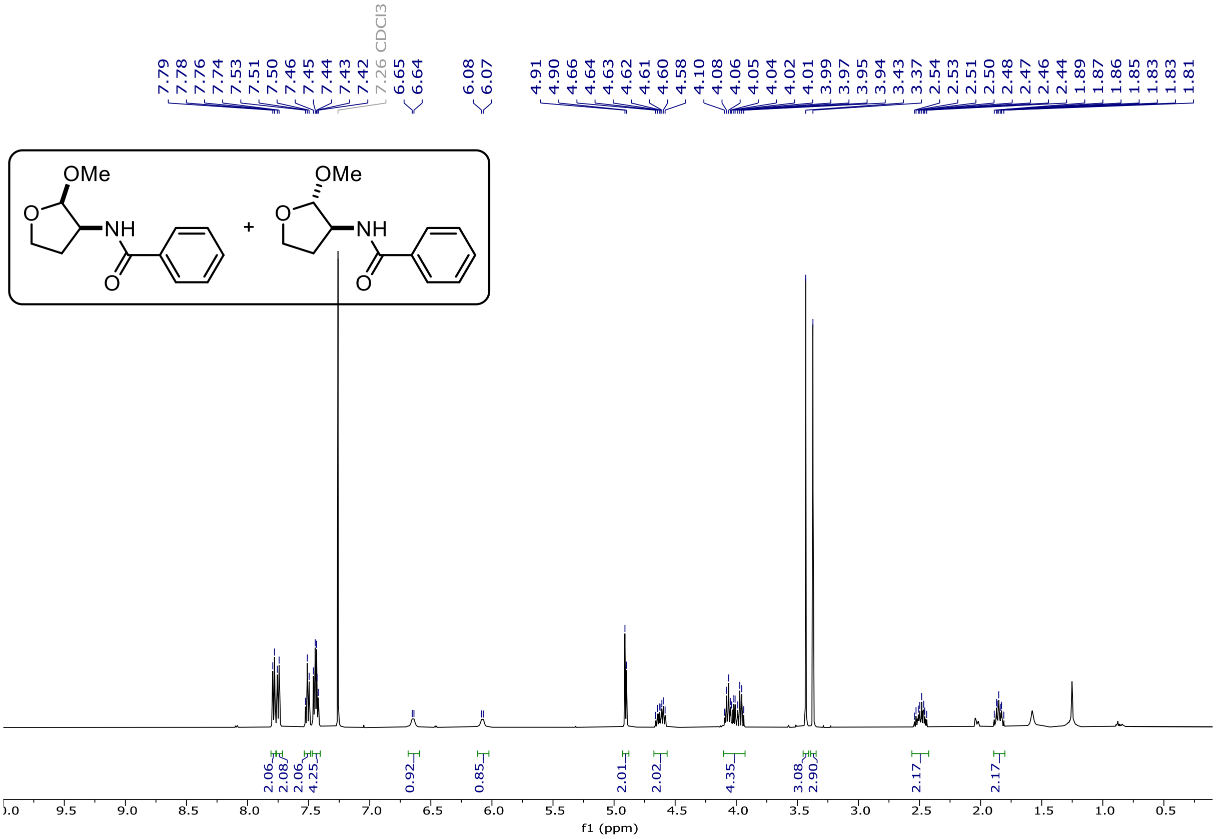

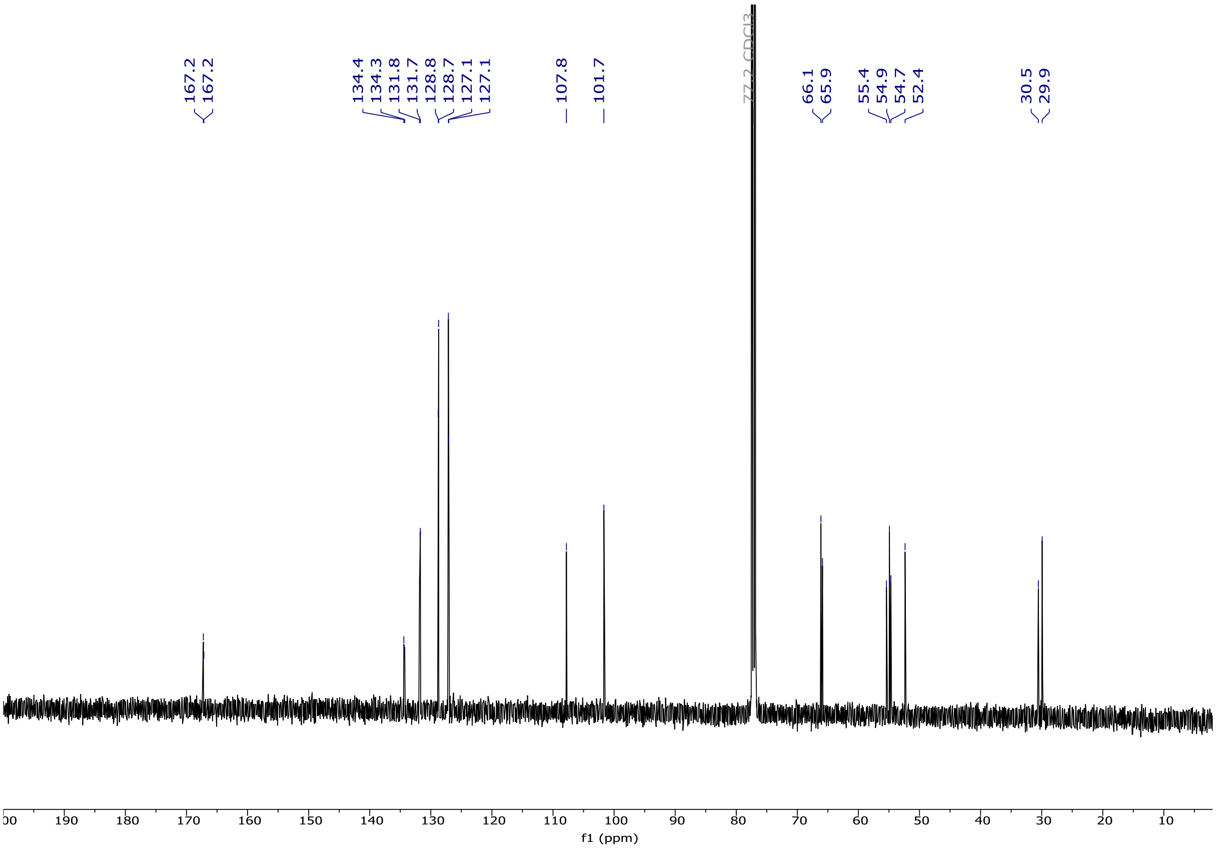

N-{2-Ethoxy-2-(2-oxopyrrolidin-1-yl)ethyl}benzamide (4z)

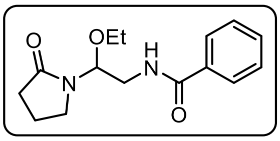

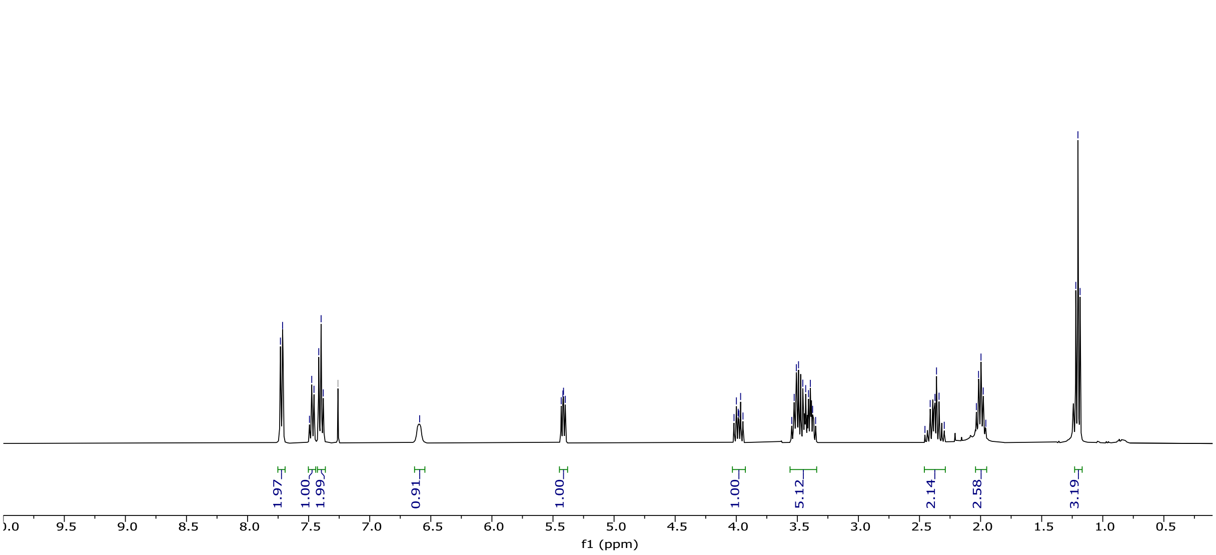

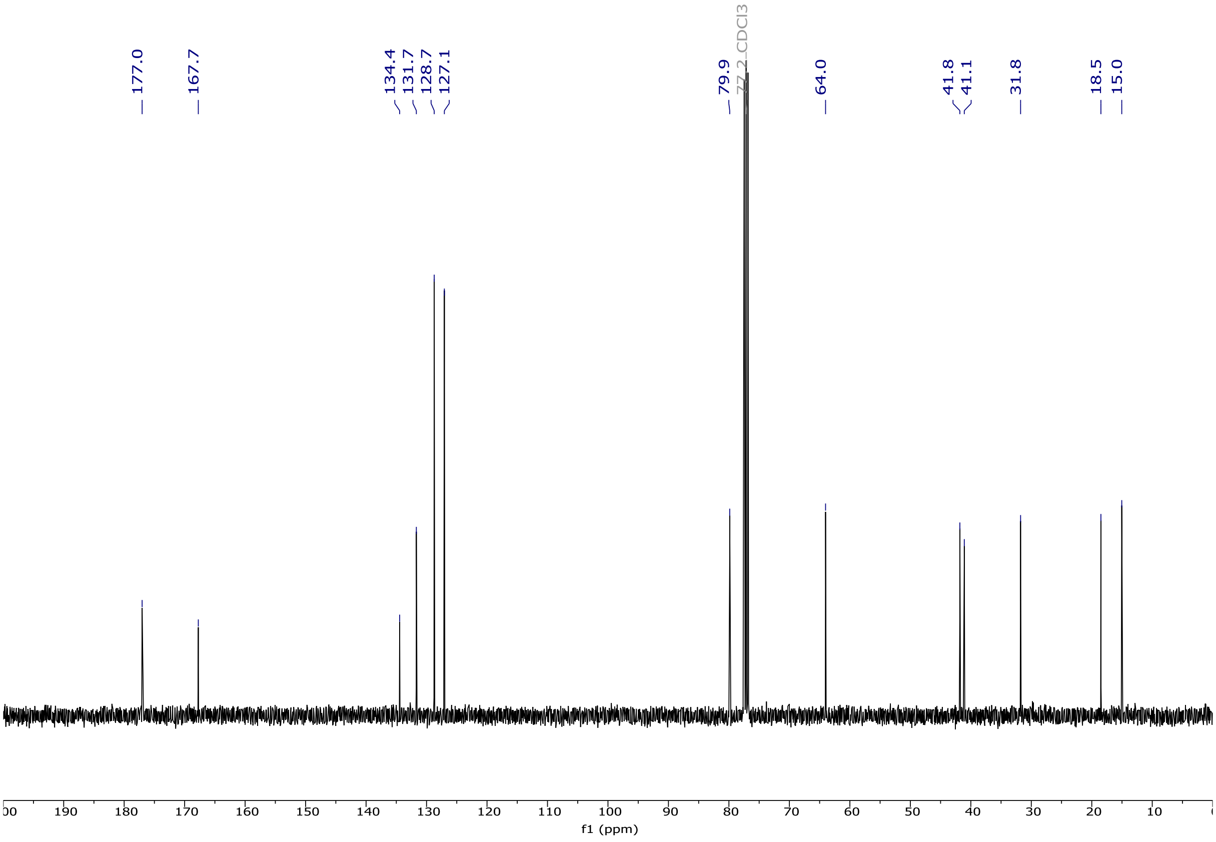

N-{2-Butoxy-2-(2-oxopyrrolidin-1-yl)ethyl}benzamide (4aa)

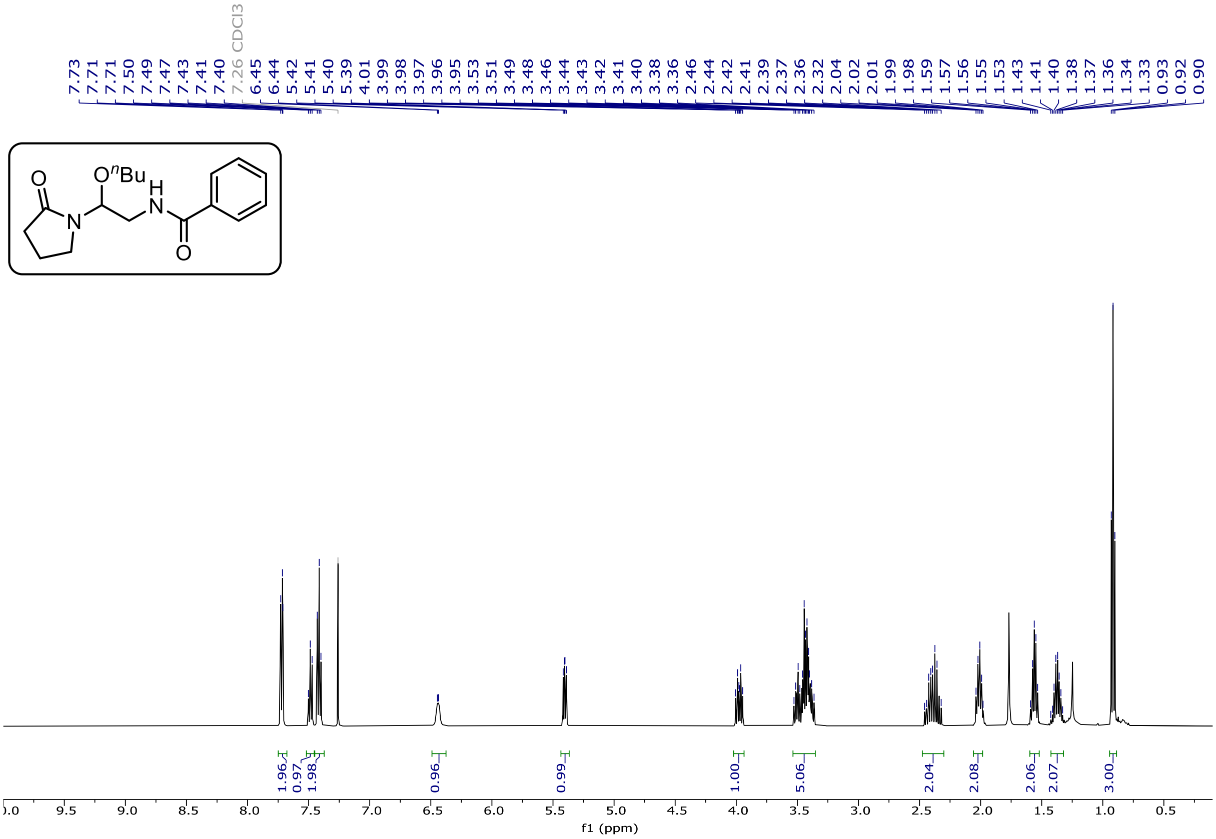

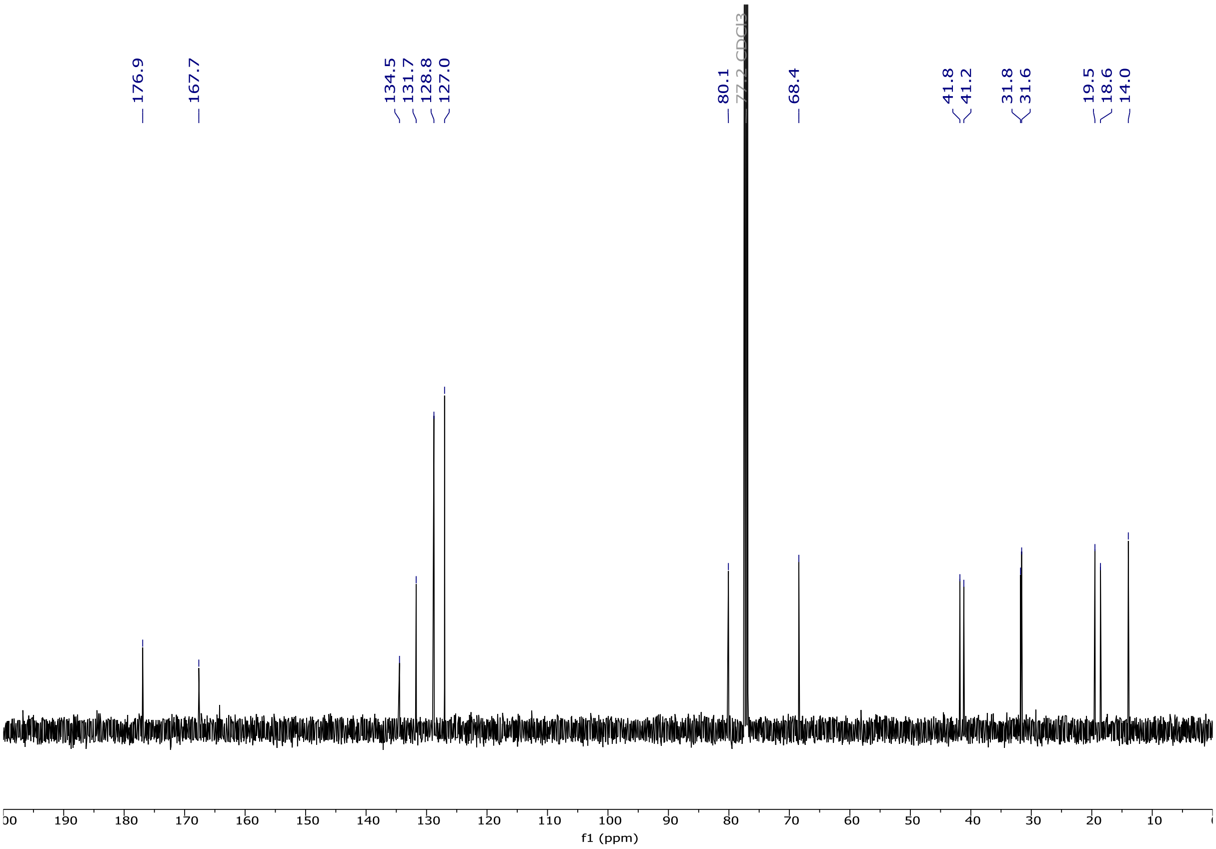

N-{2-Isopropoxy-2-(2-oxopyrrolidin-1-yl)ethyl}benzamide (4ab)

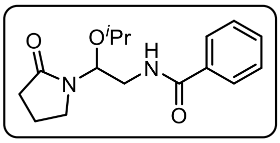

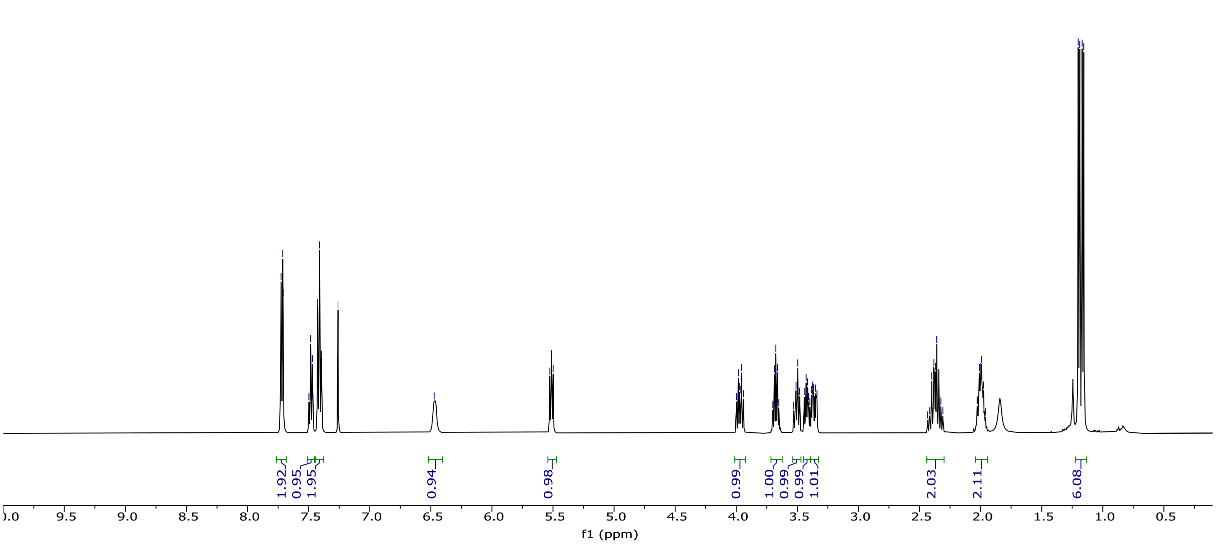

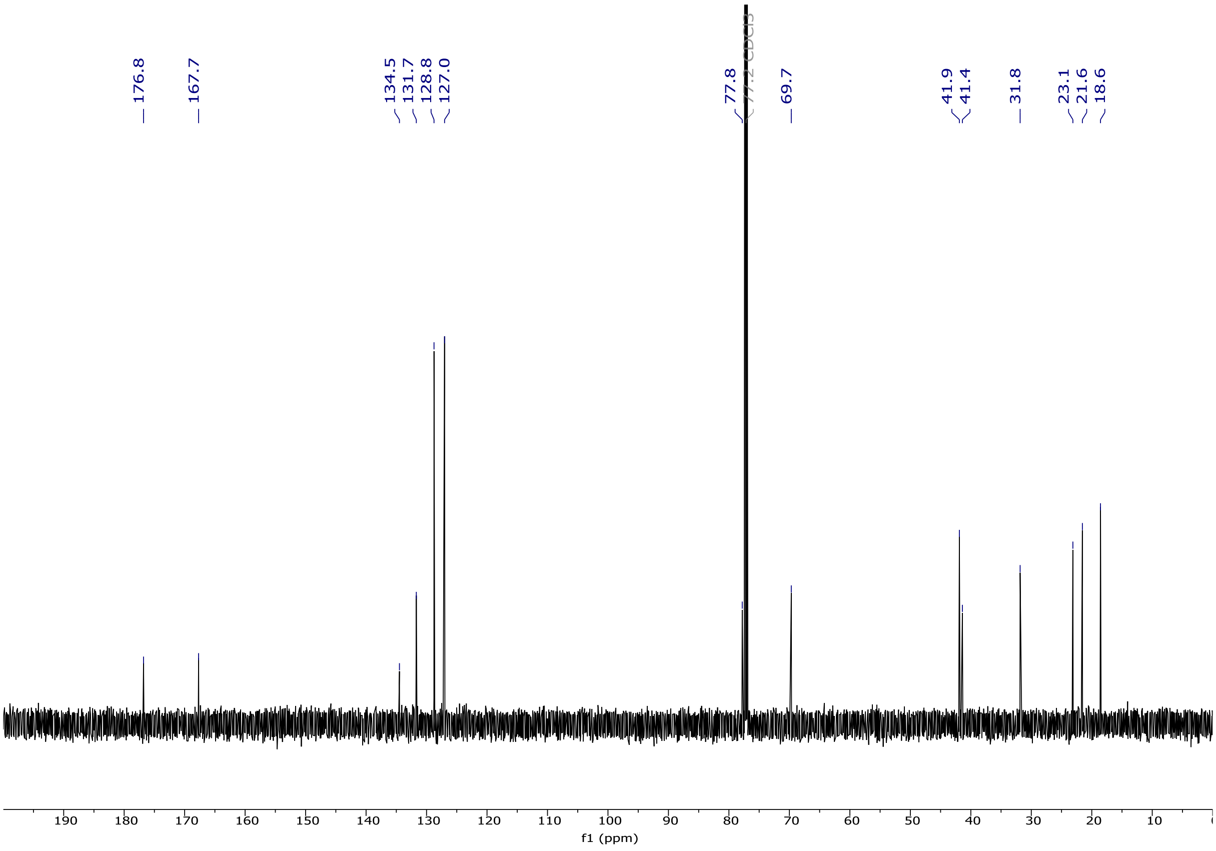

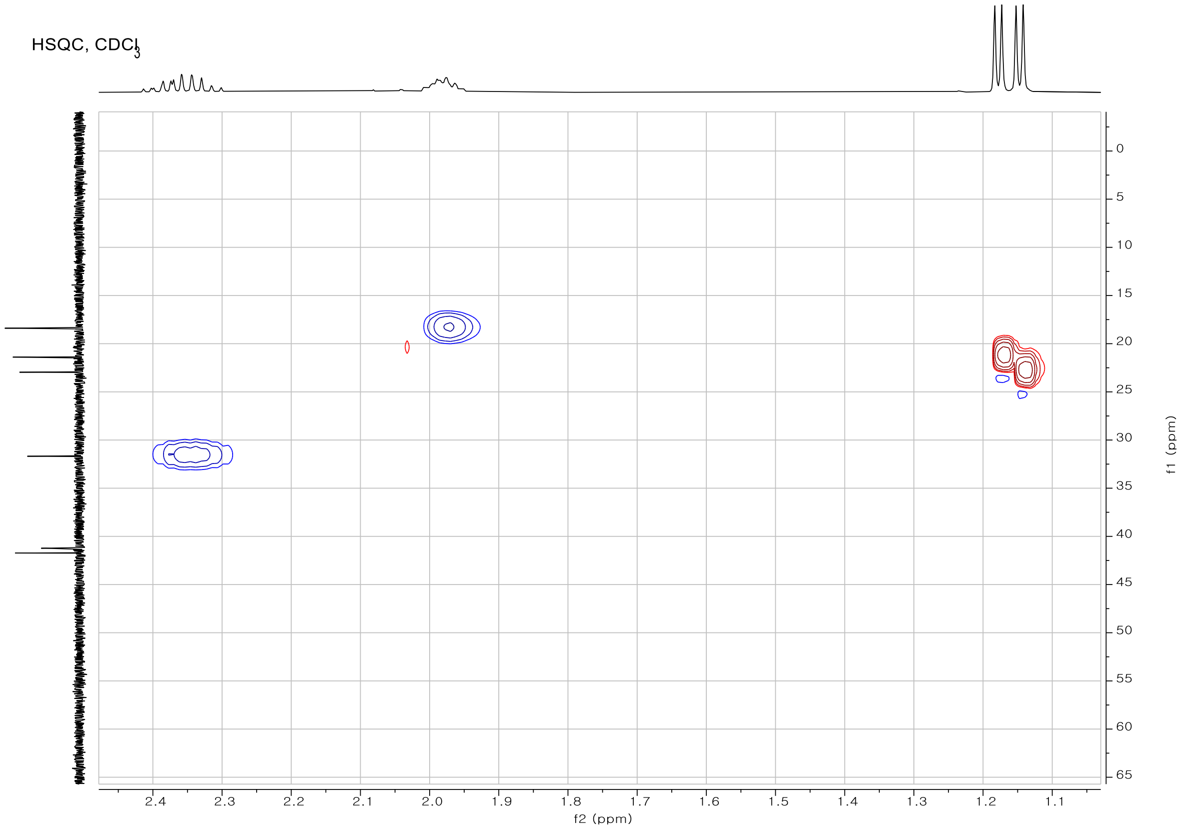

The two terminal methyl carbons of the isopropyl group appear as distinct signals in the ^{13}C NMR spectrum.

N-{2-(*tert*-Butoxy)-2-(2-oxopyrrolidin-1-yl)ethyl}benzamide (4ac)

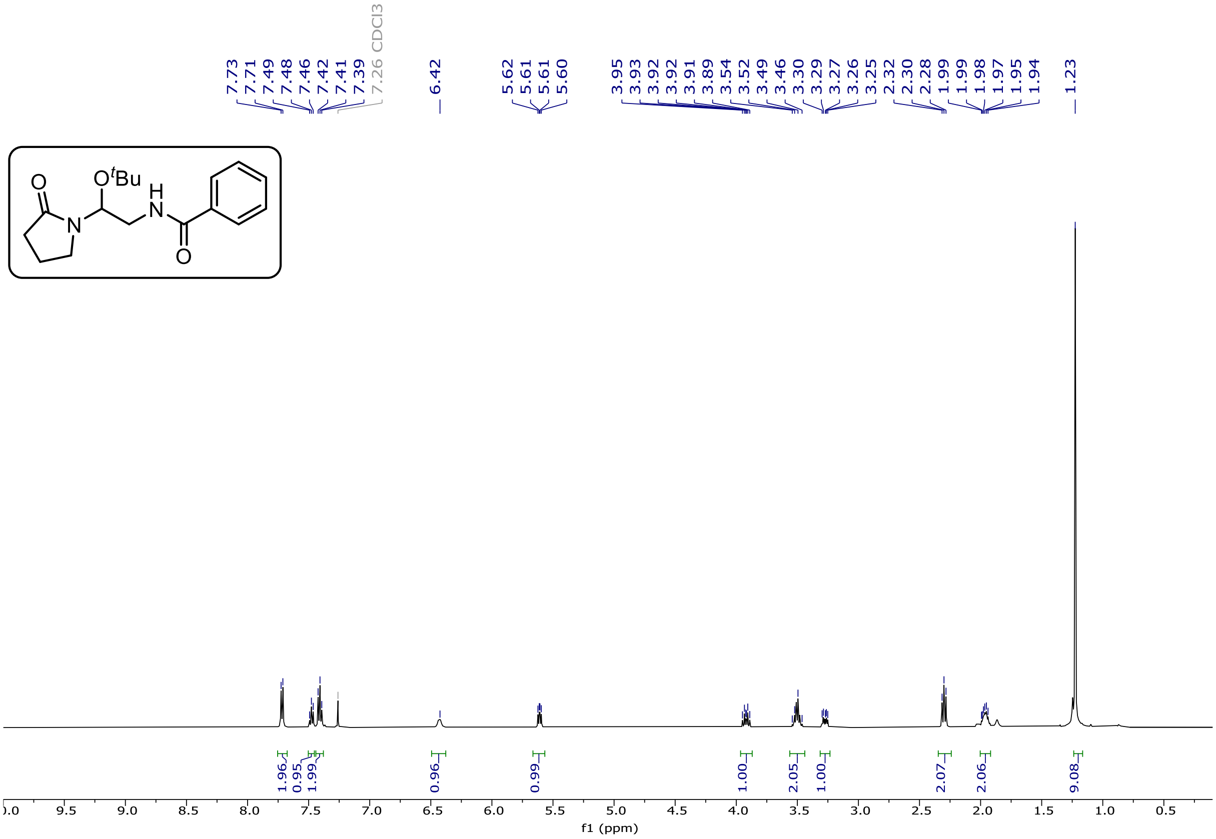

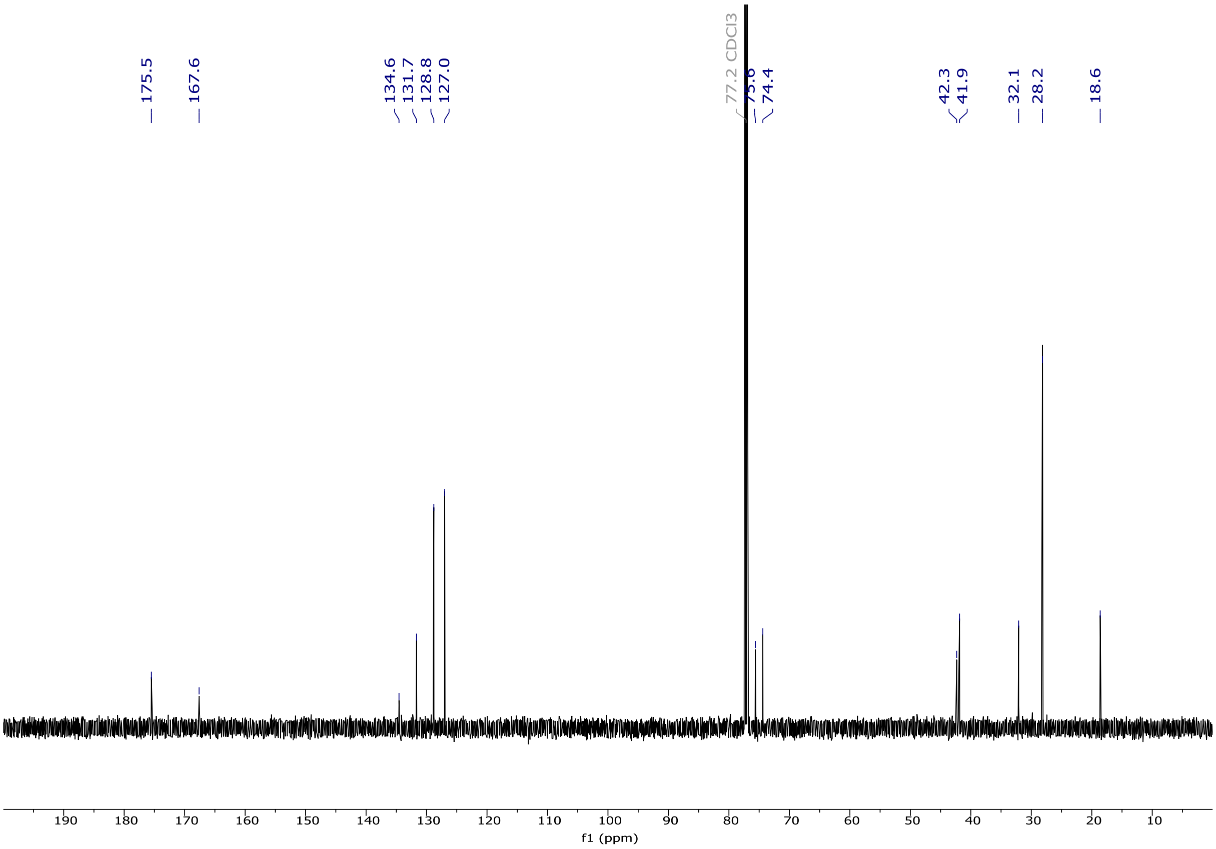

N-(2-Methoxy-2-phenylethyl)-4-methylbenzamide (4ad)

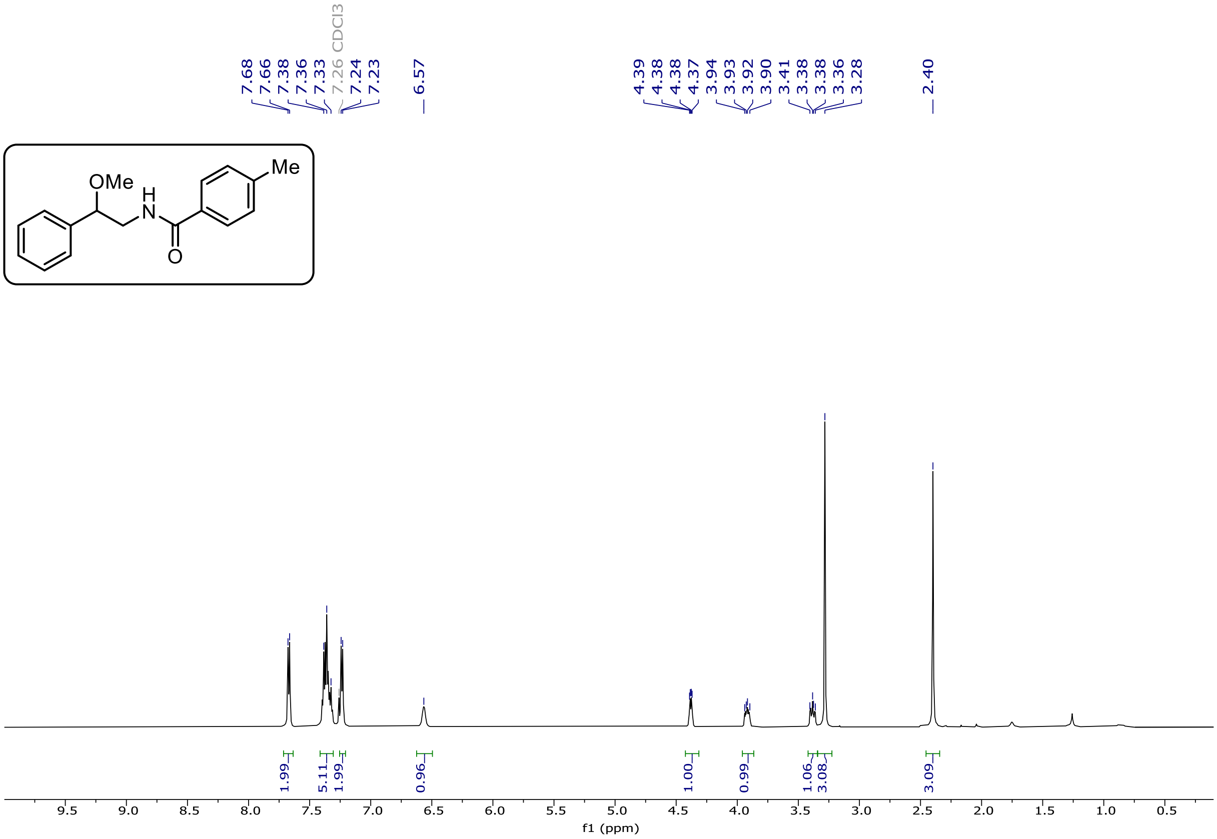

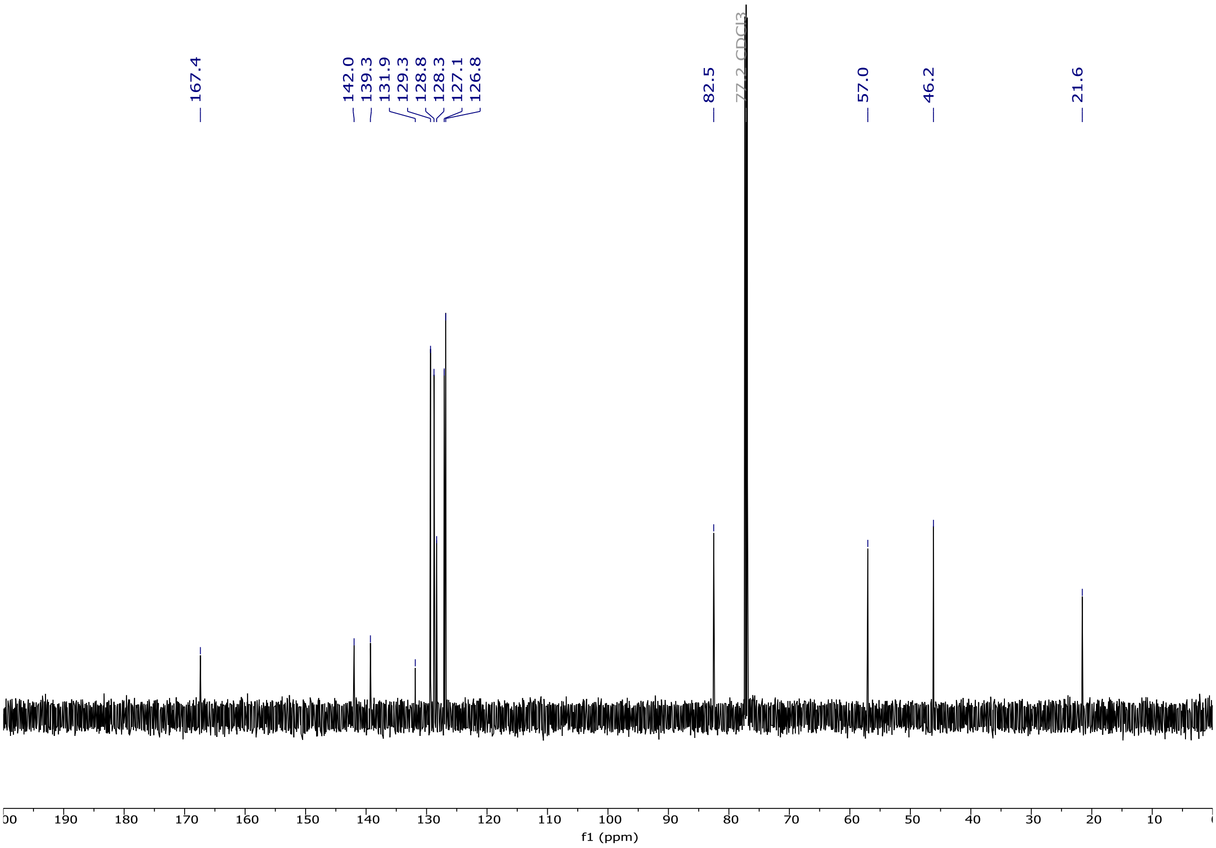

4-Chloro-*N*-(2-methoxy-2-phenylethyl)benzamide (4ae)

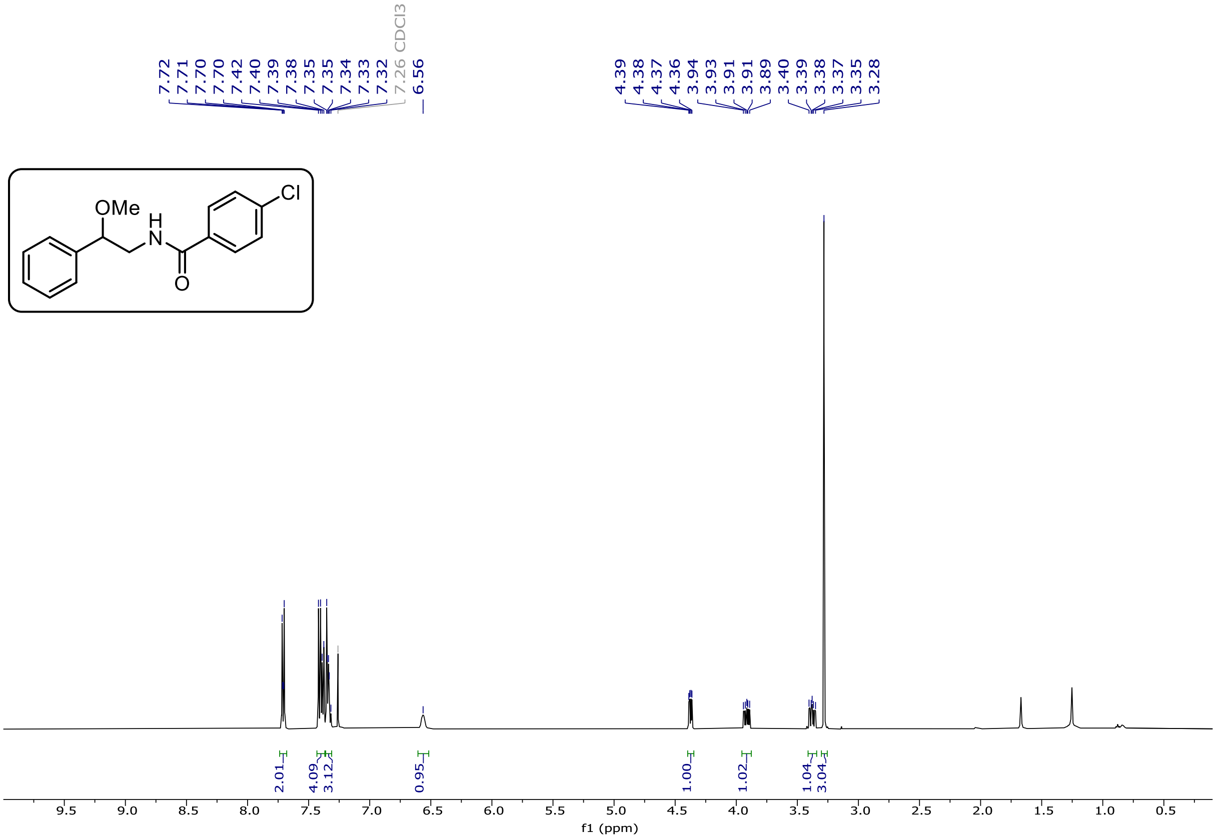

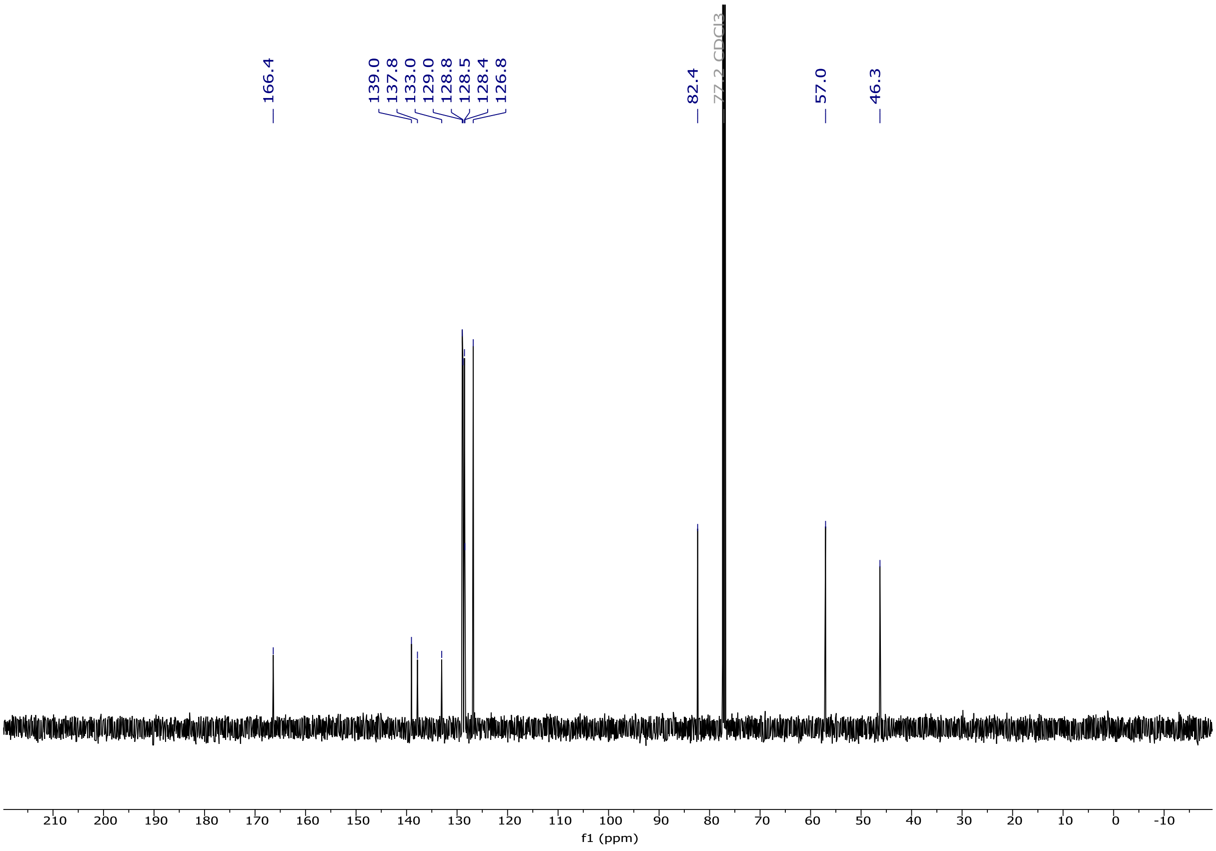

4-Methoxy-*N*-(2-methoxy-2-phenylethyl)benzamide (4af)

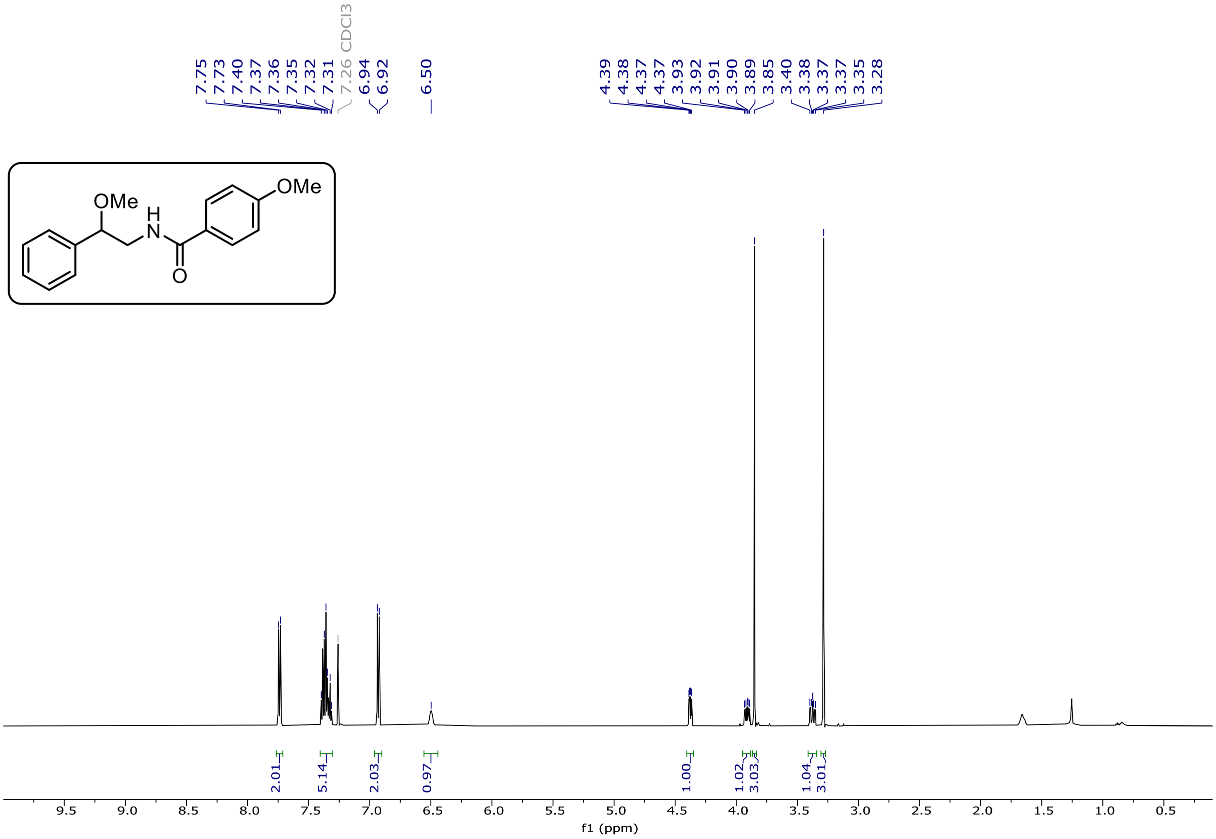

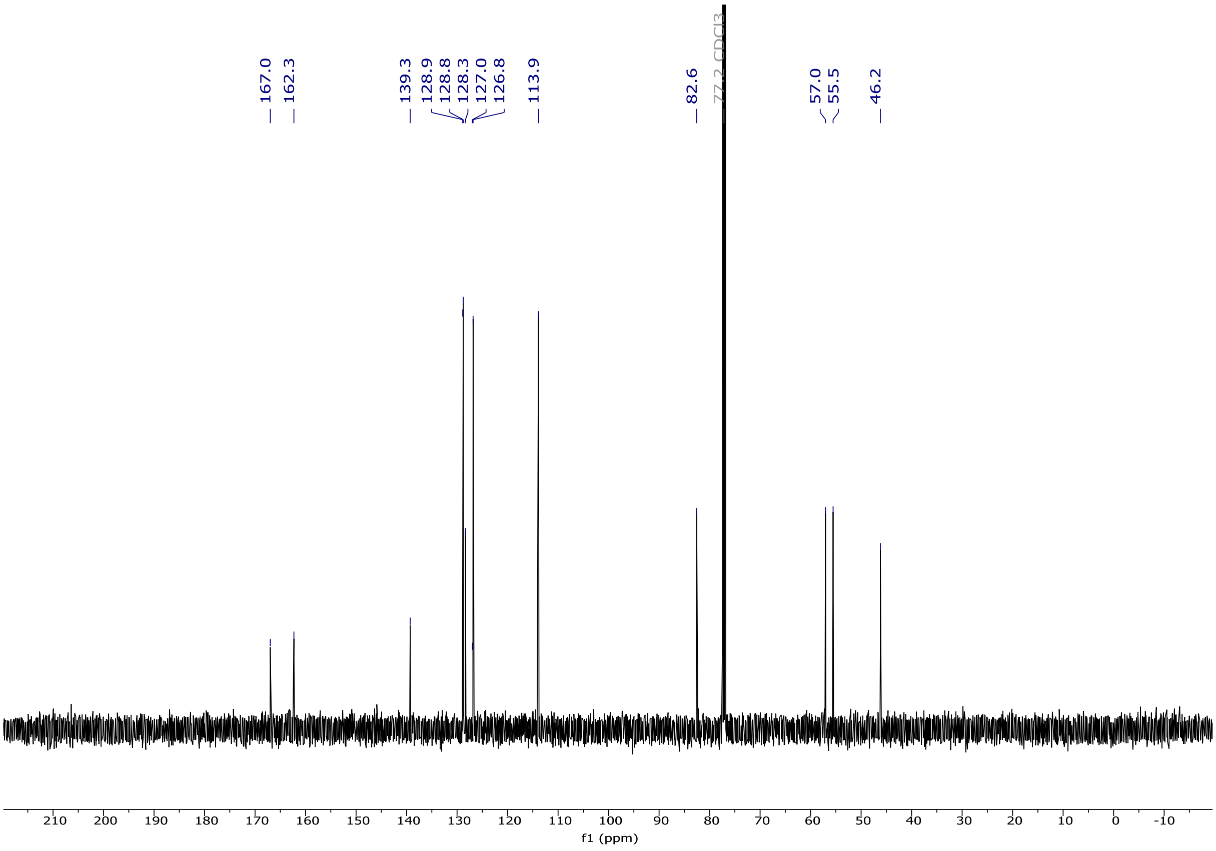

_N_-(2-Methoxy-2-phenylethyl)thiophene-3-carboxamide (4ag)

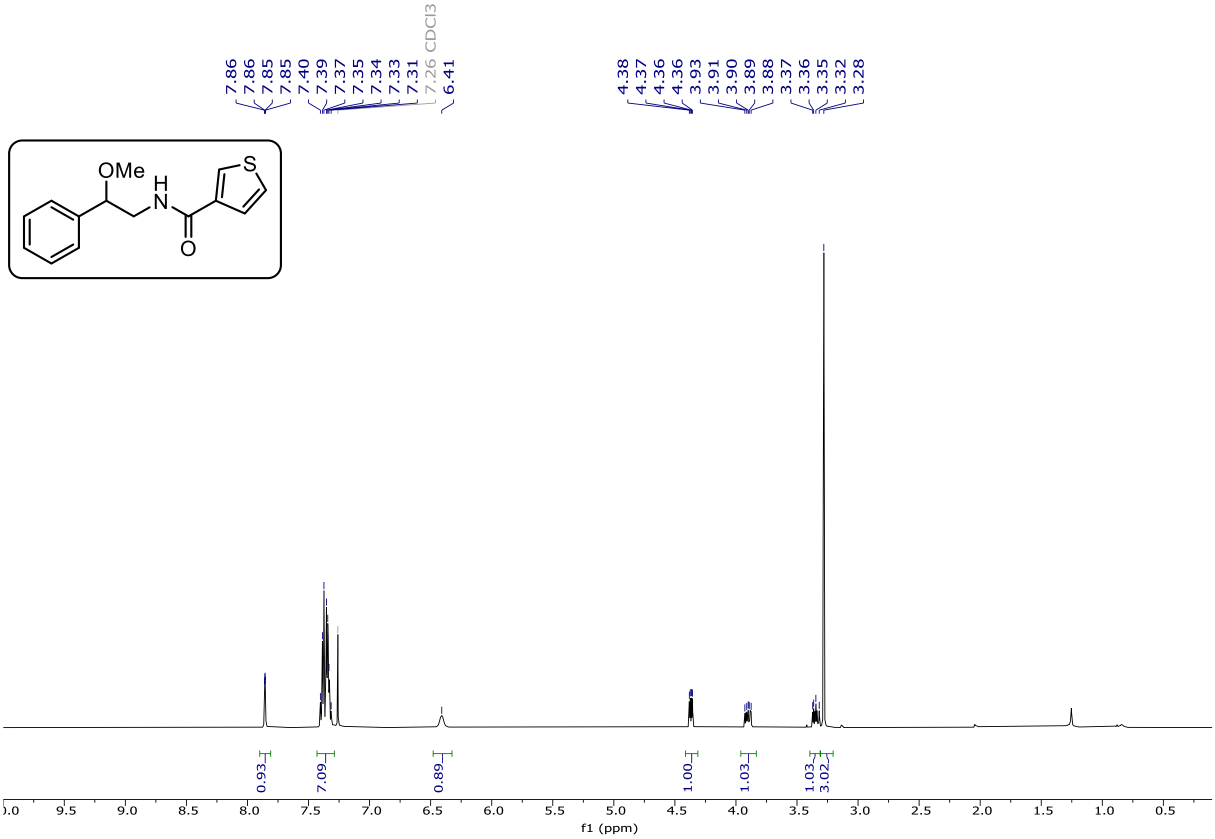

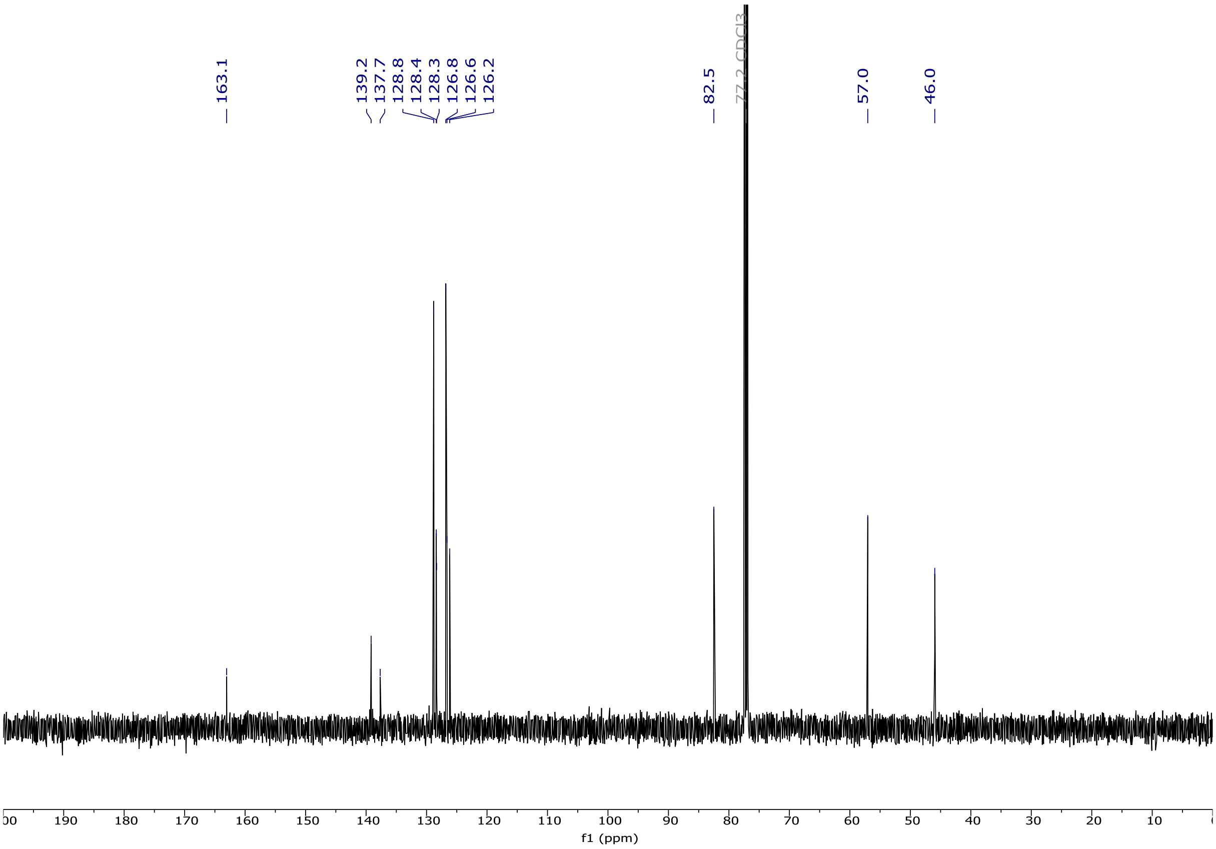

N-(2-Methoxy-2-phenylethyl)cyclohexanecarboxamide (4ah)

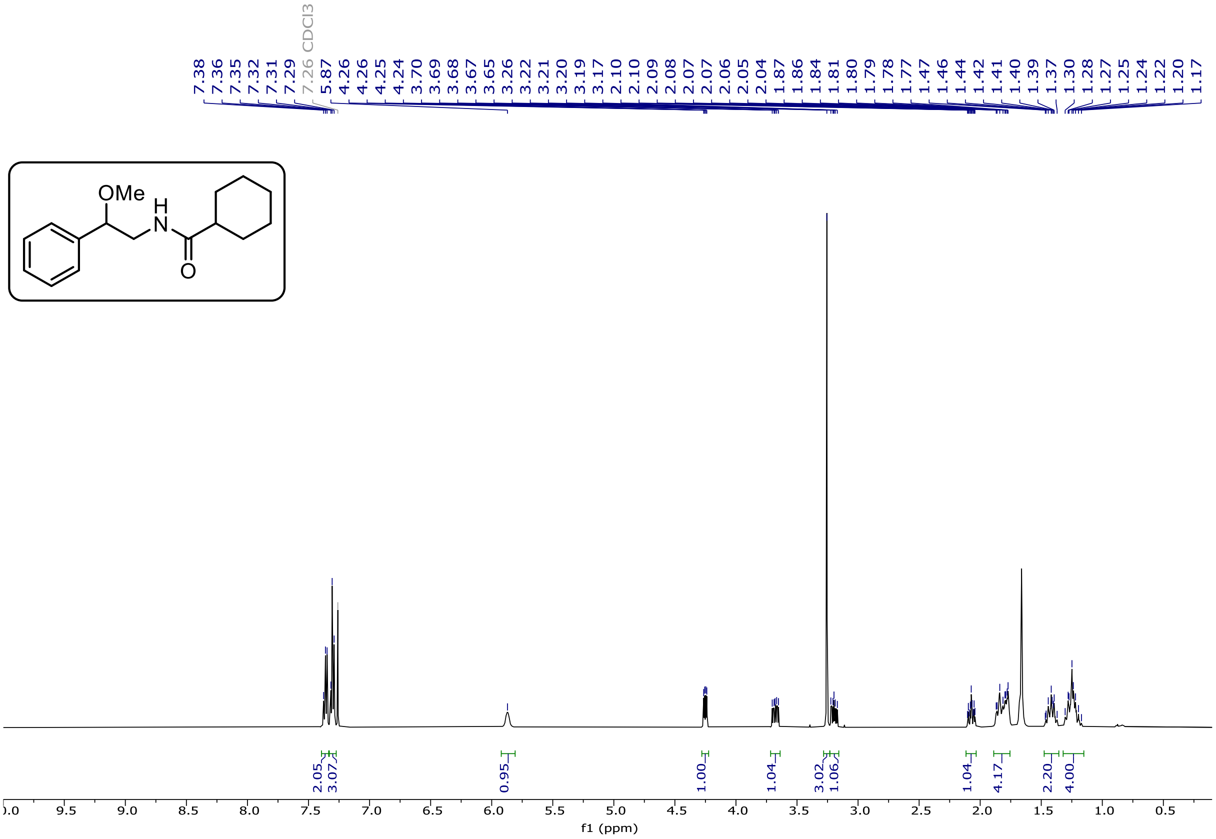

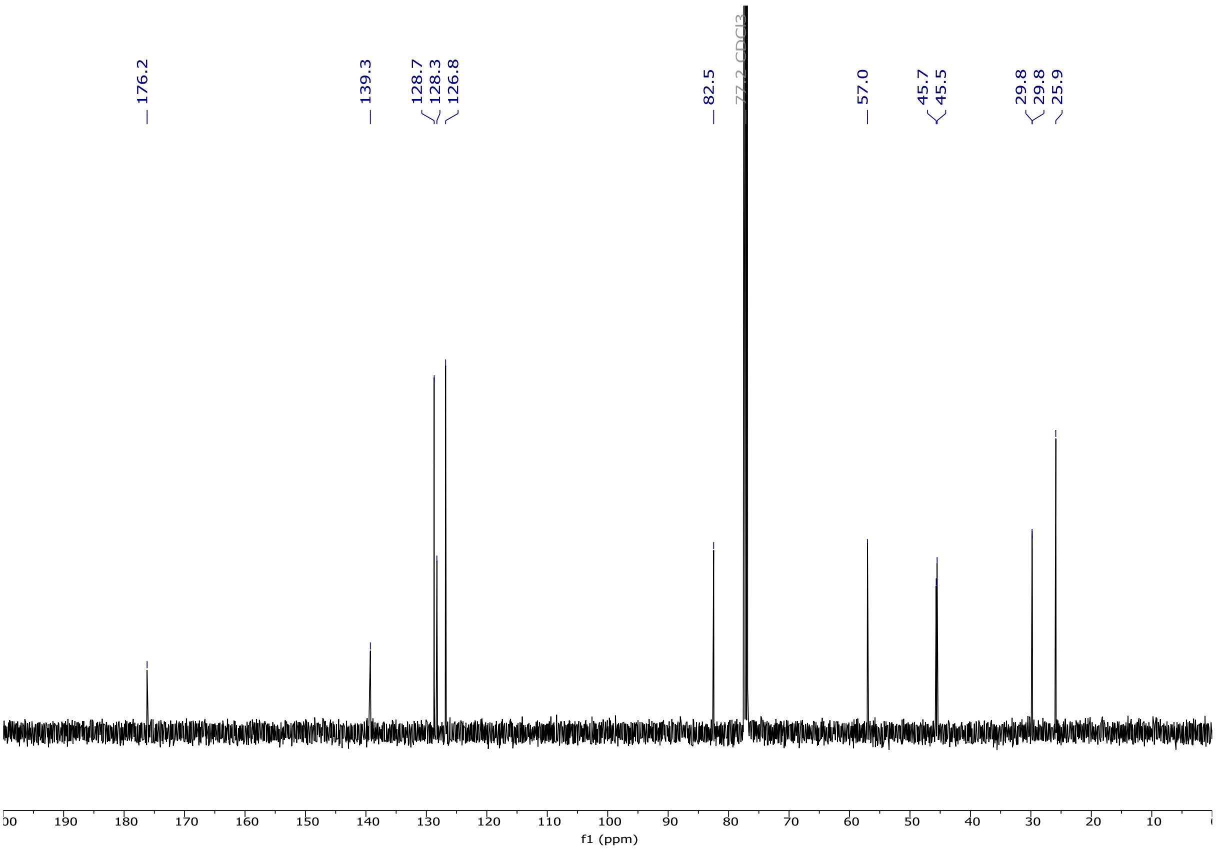

***N*-(2-Azido-2-phenylethyl)benzamide (5a)**

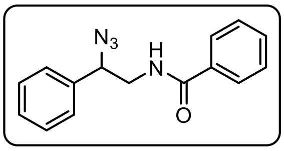

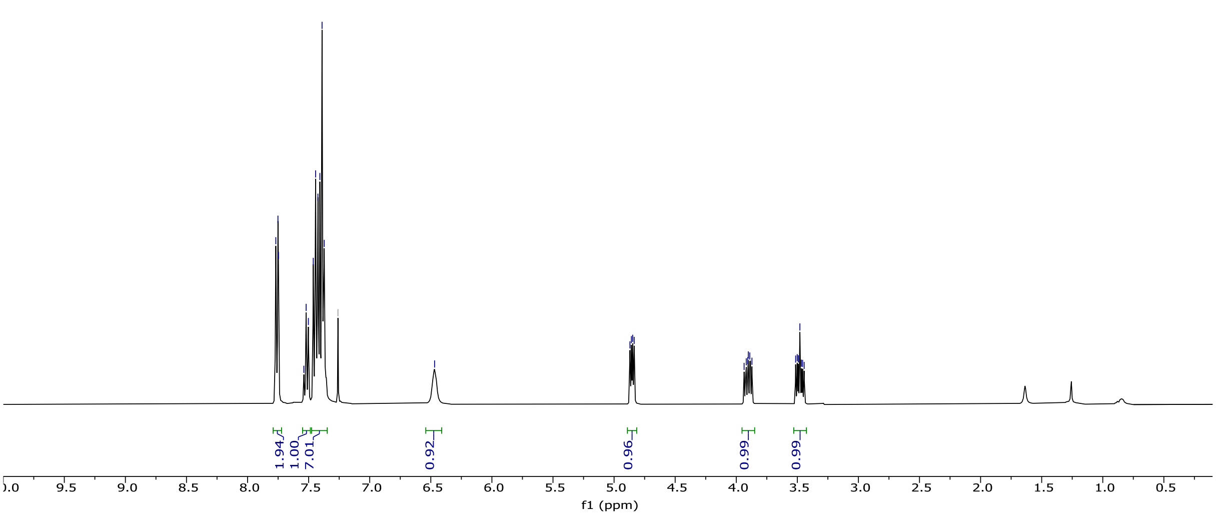

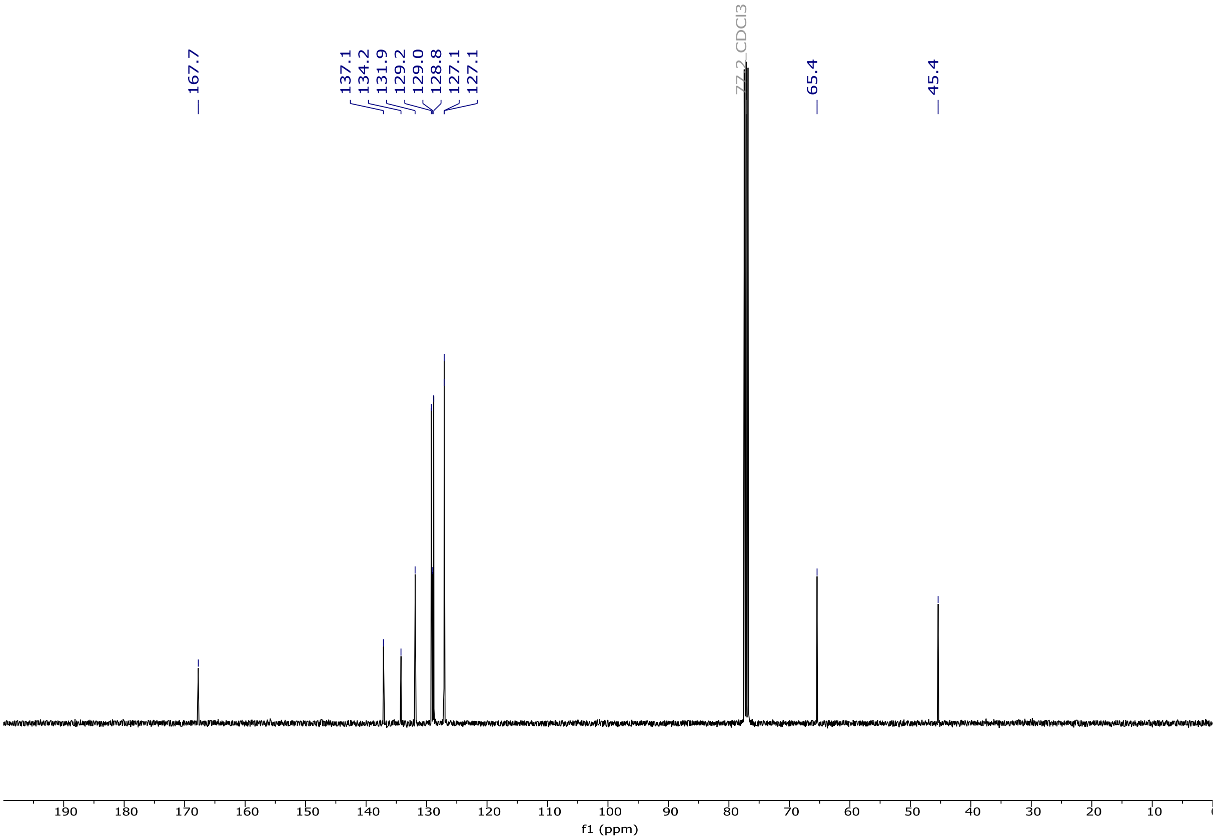

N-[2-Azido-2-{4-(tert-butyl)phenyl}ethyl]benzamide (5b)

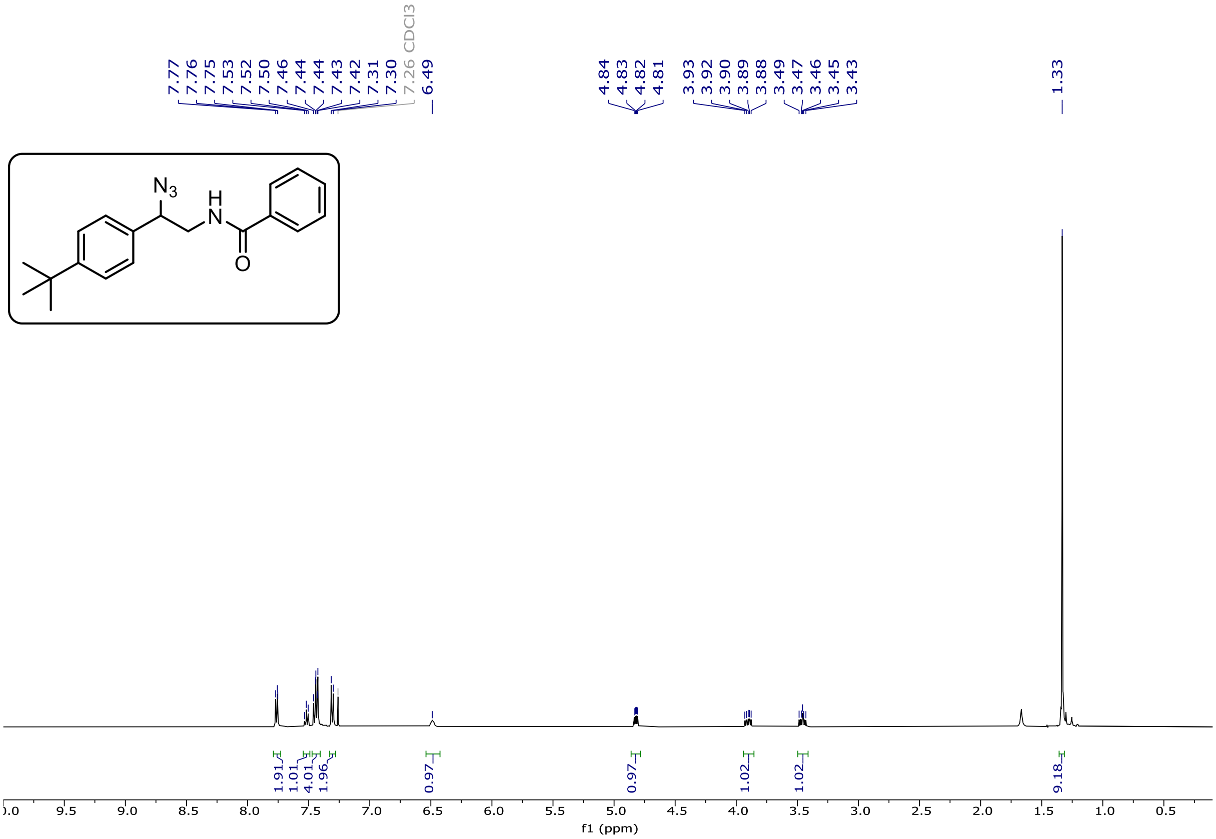

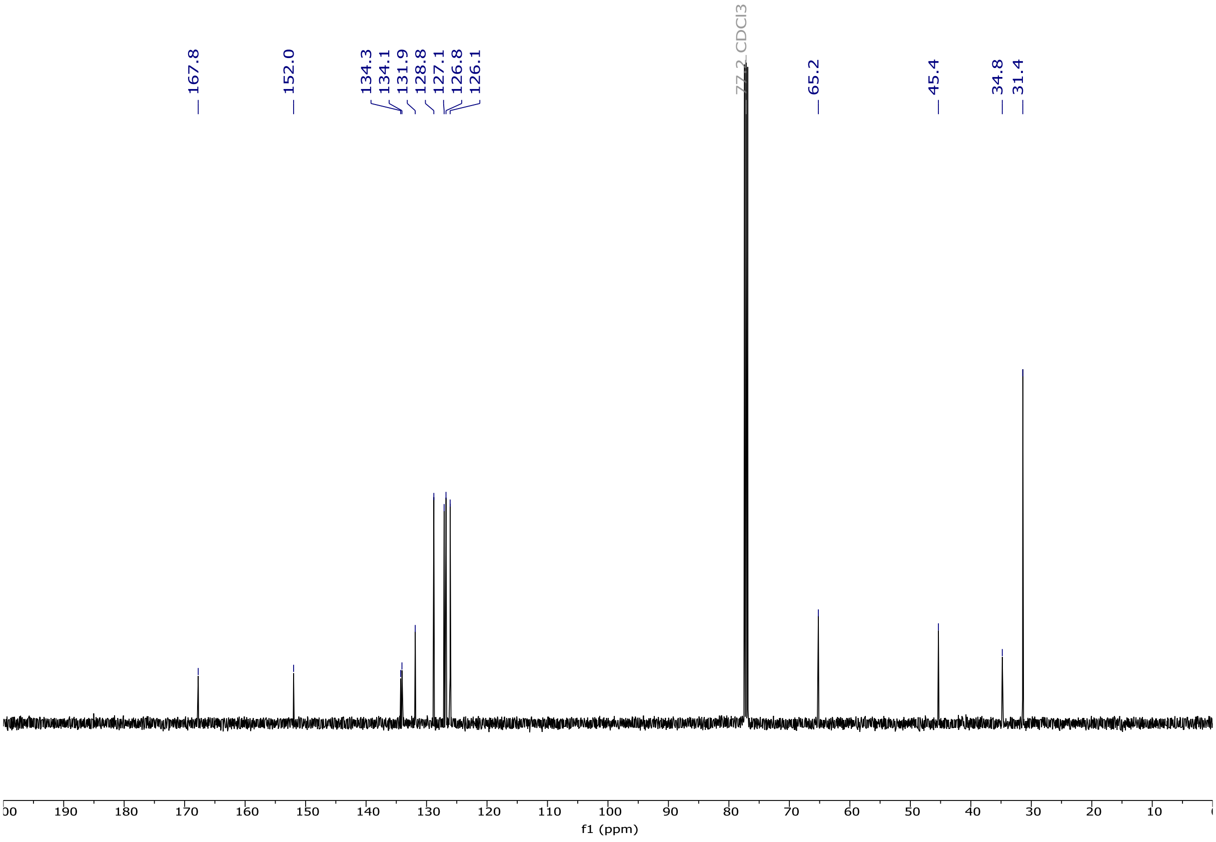

N-{2-Azido-2-(4-chlorophenyl)ethyl}benzamide (5c)

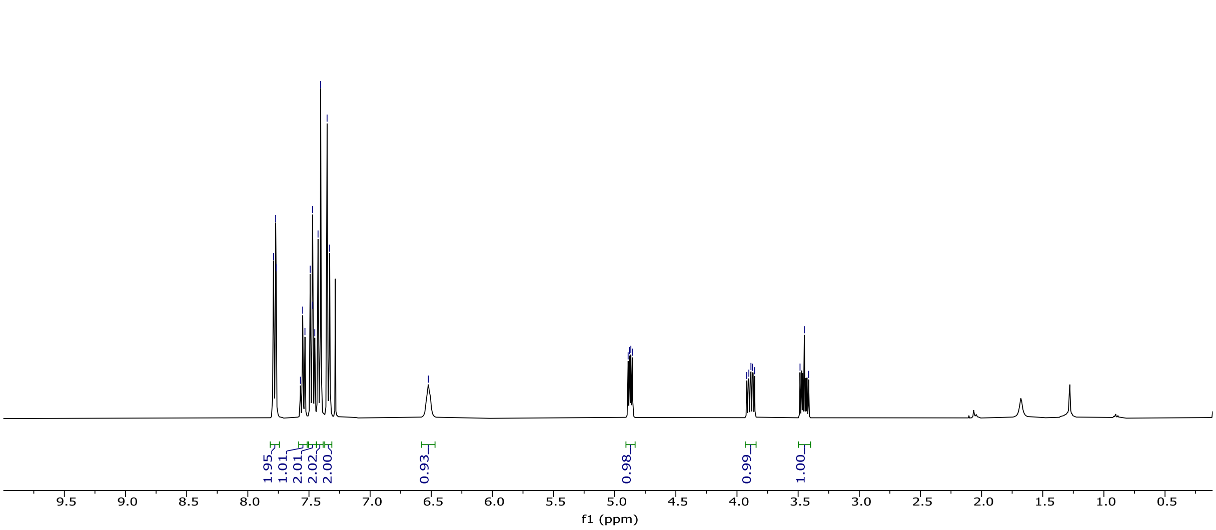

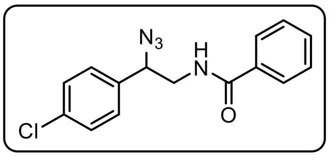

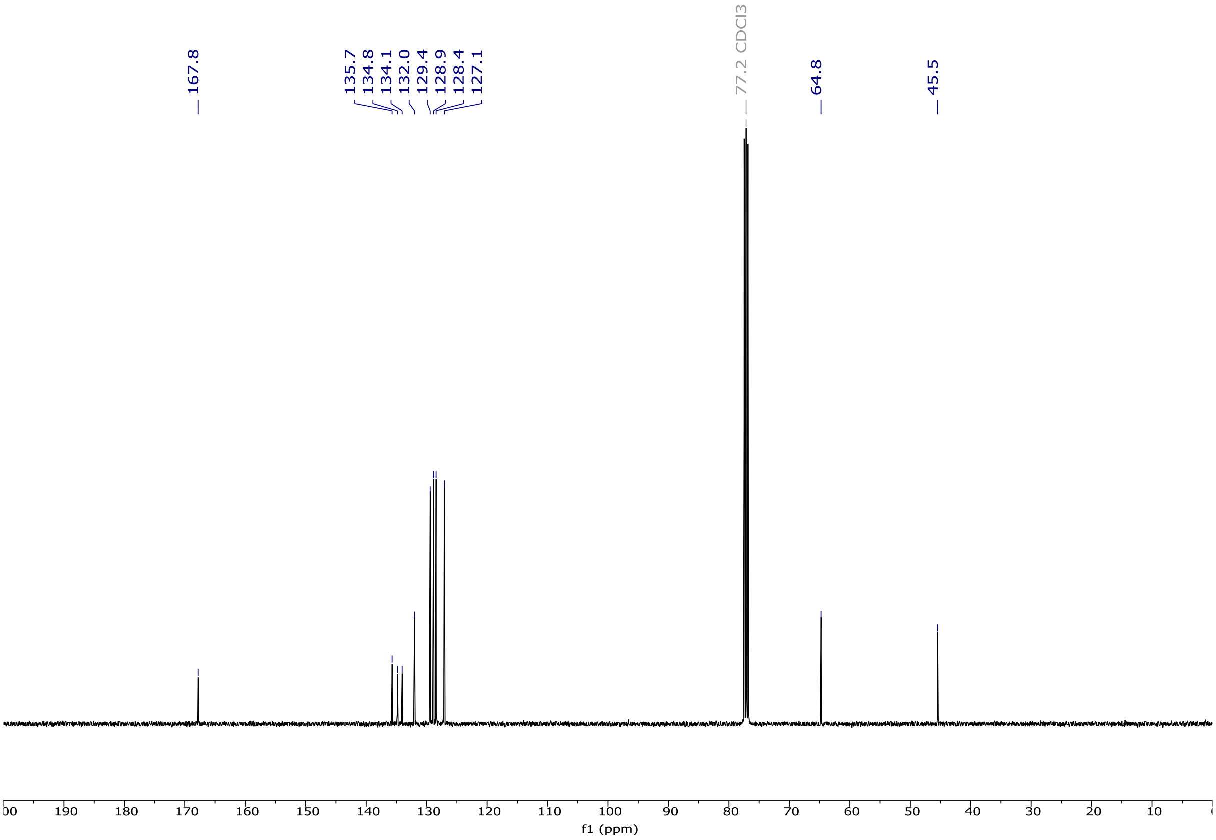

N-{2-Azido-2-(3-bromophenyl)ethyl}benzamide (5d)

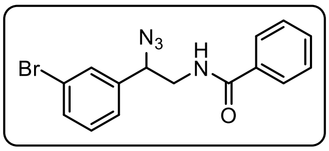

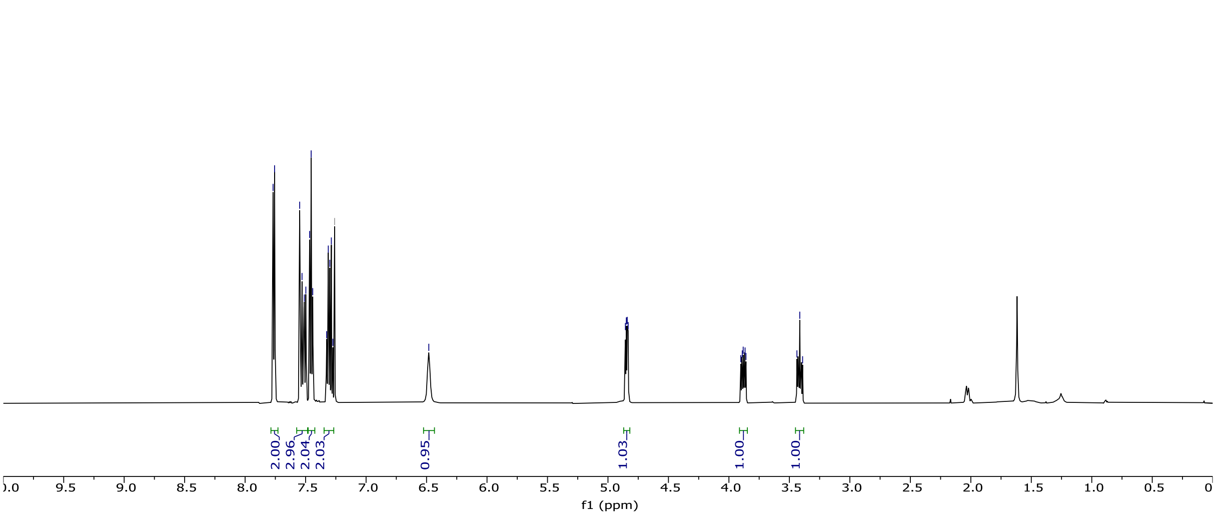

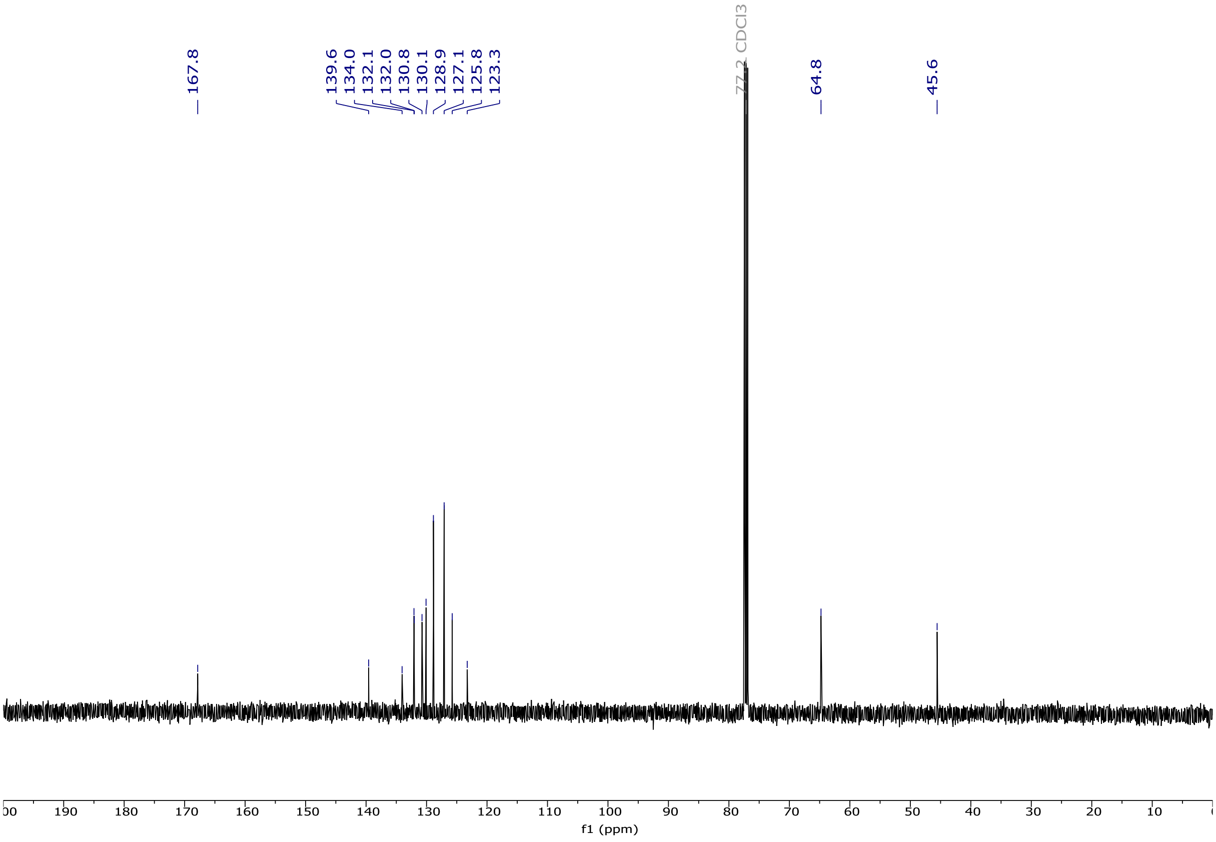

***N*- {2-Azido-2-(*o*-tolyl)ethyl}benzamide (5e)**

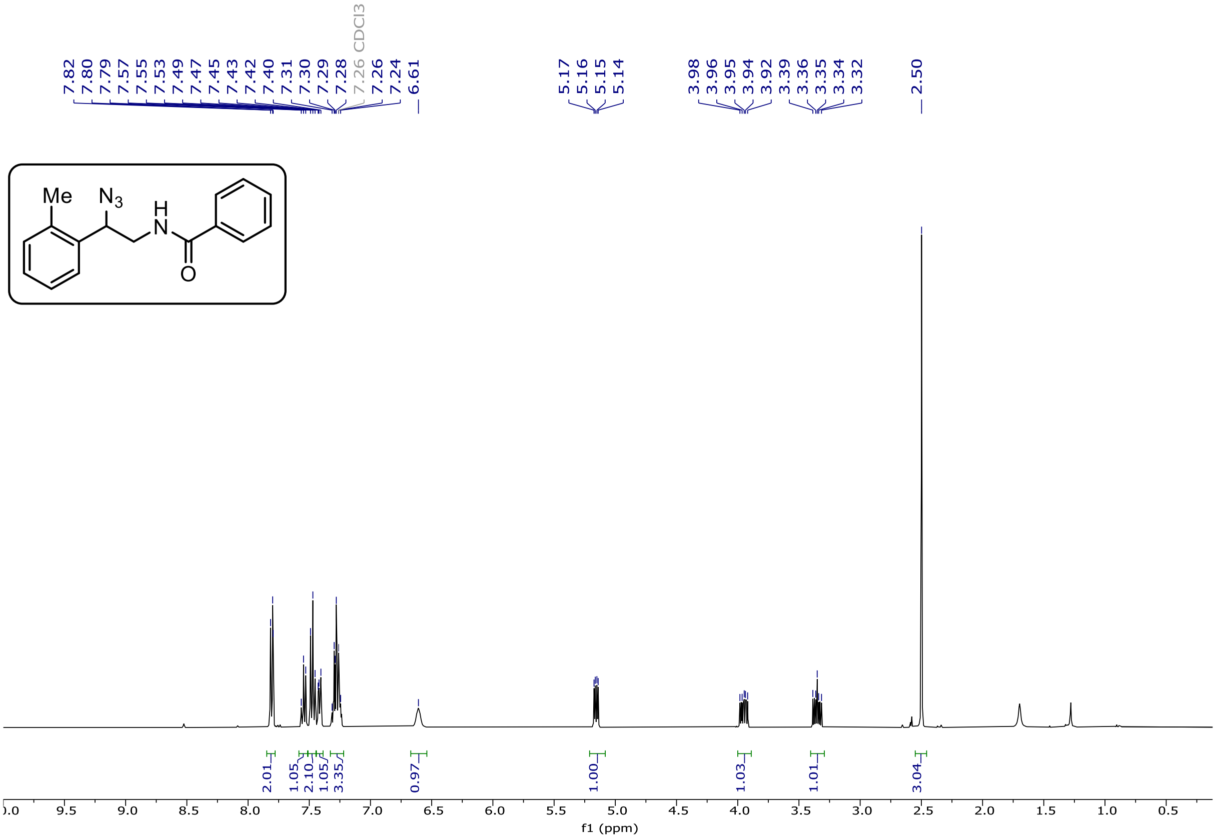

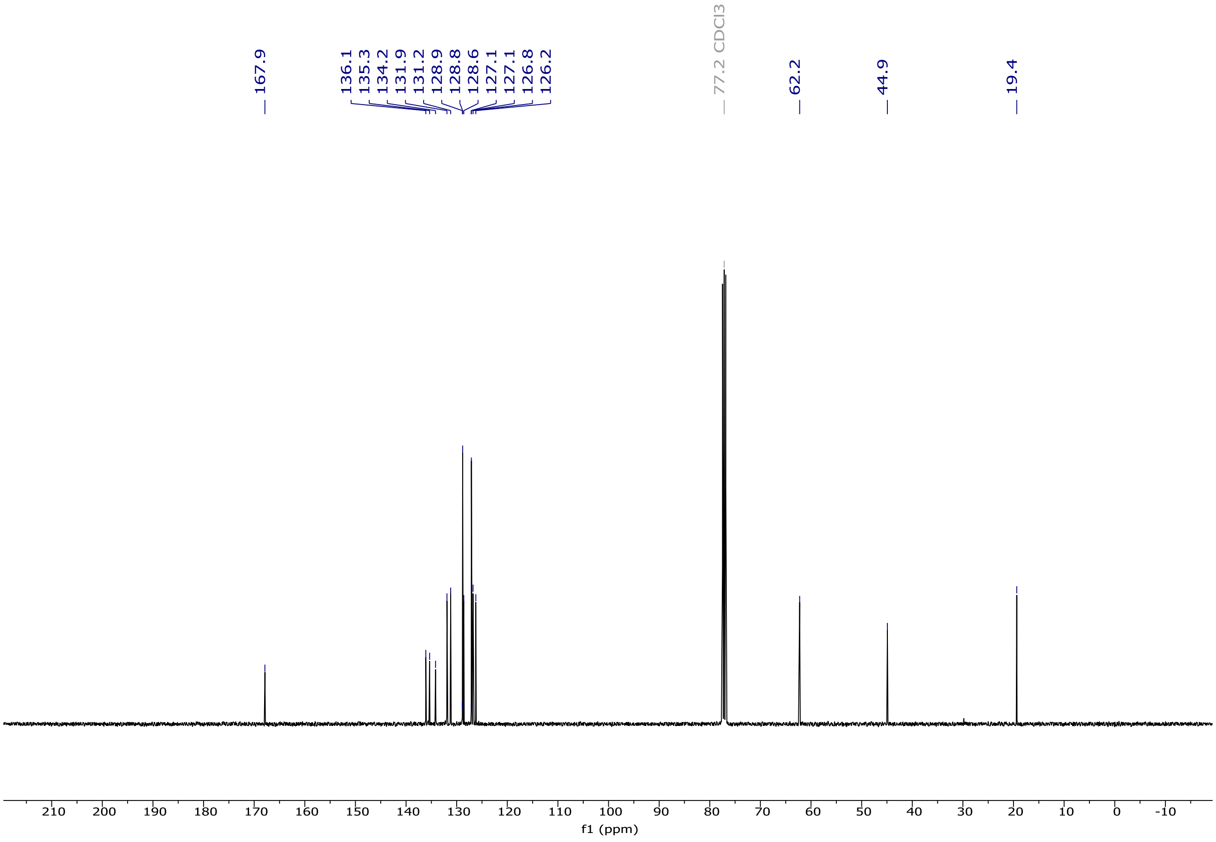

N-{2-Azido-2-(naphthalen-2-yl)ethyl}benzamide (5f)

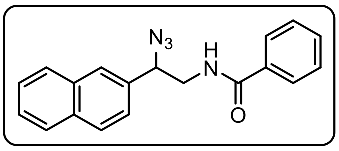

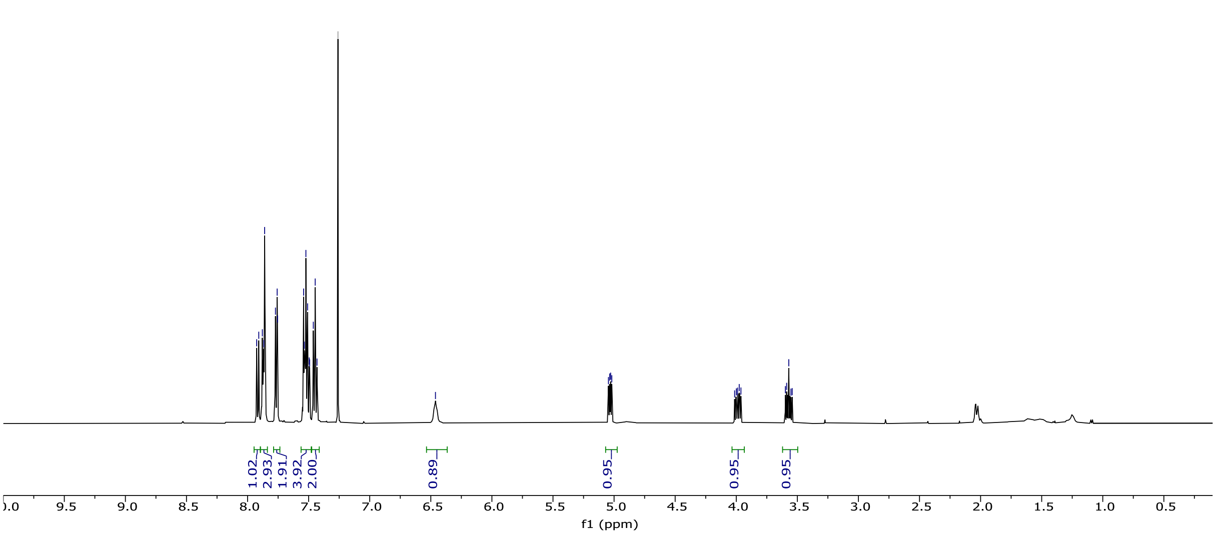

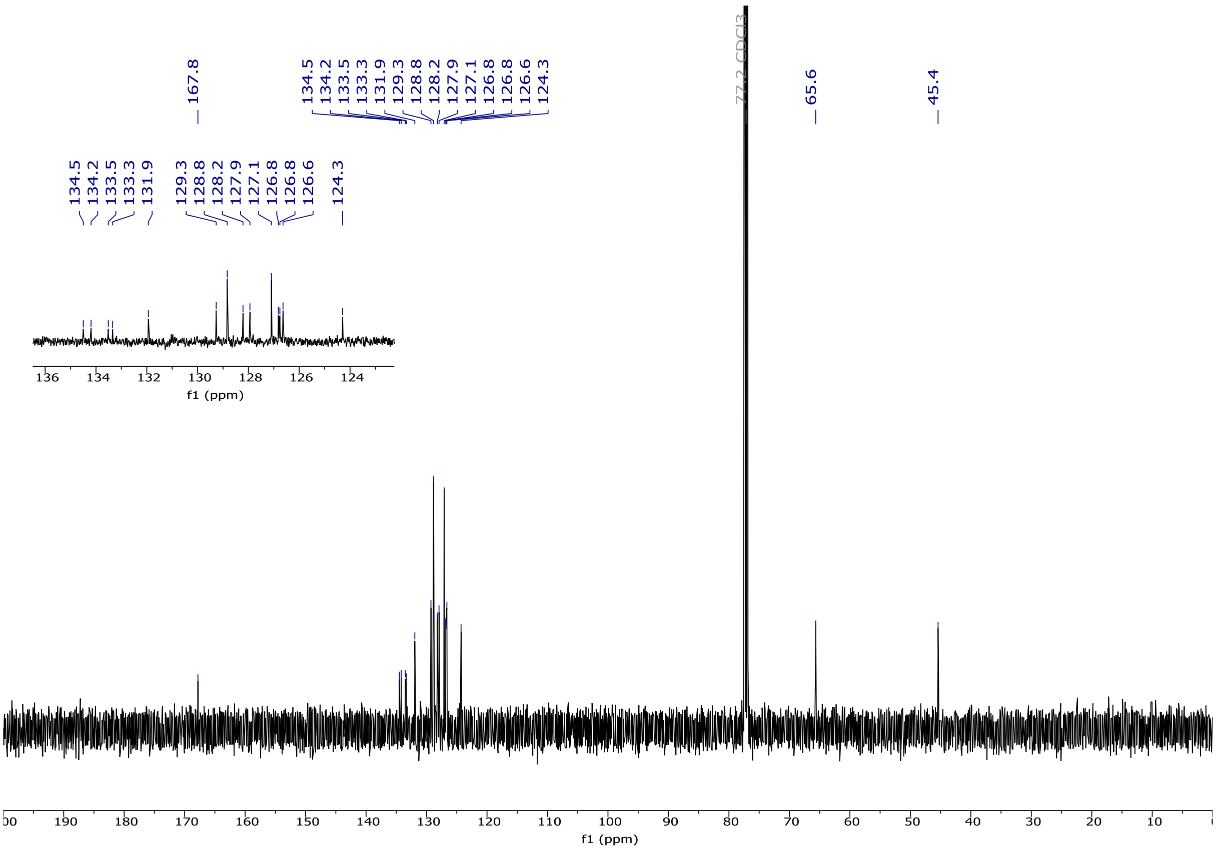

N-{2-Azido-2-(2-oxopyrrolidin-1-yl)ethyl}benzamide (5g)

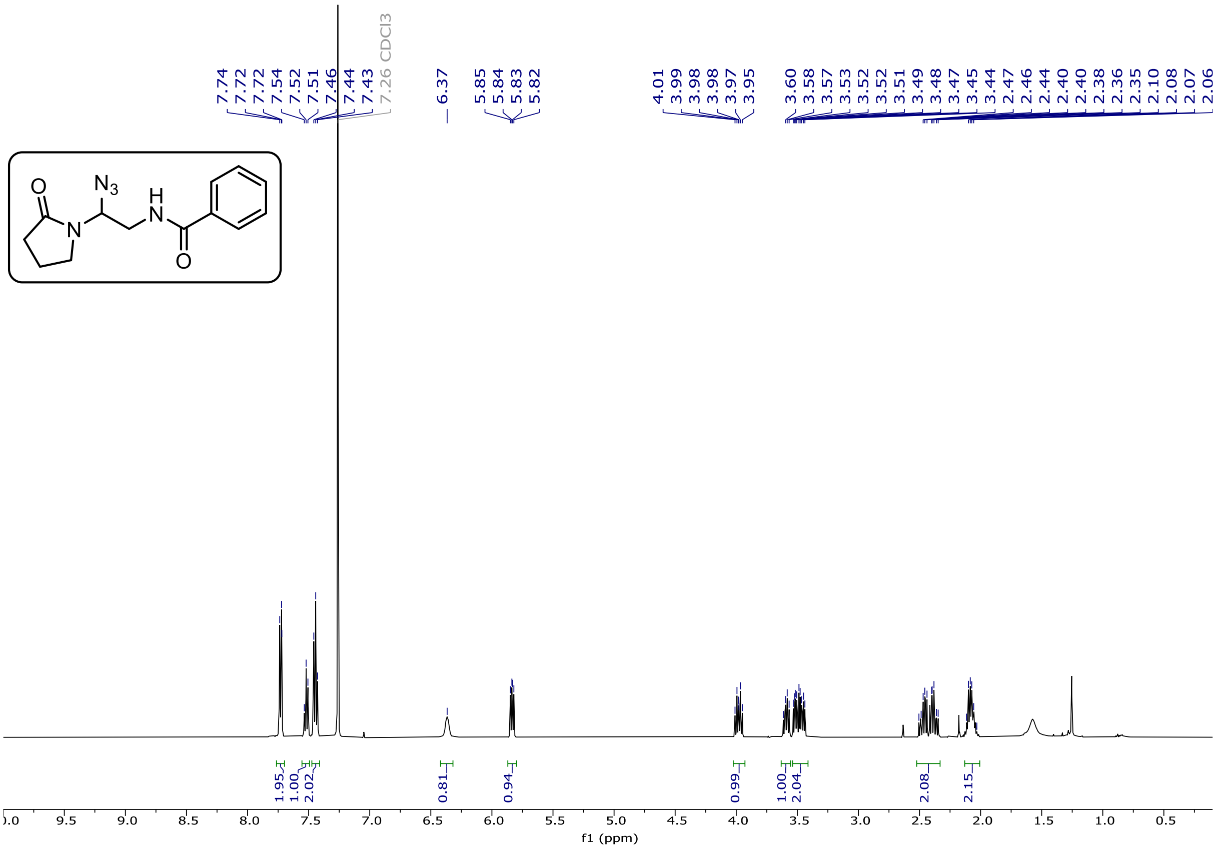

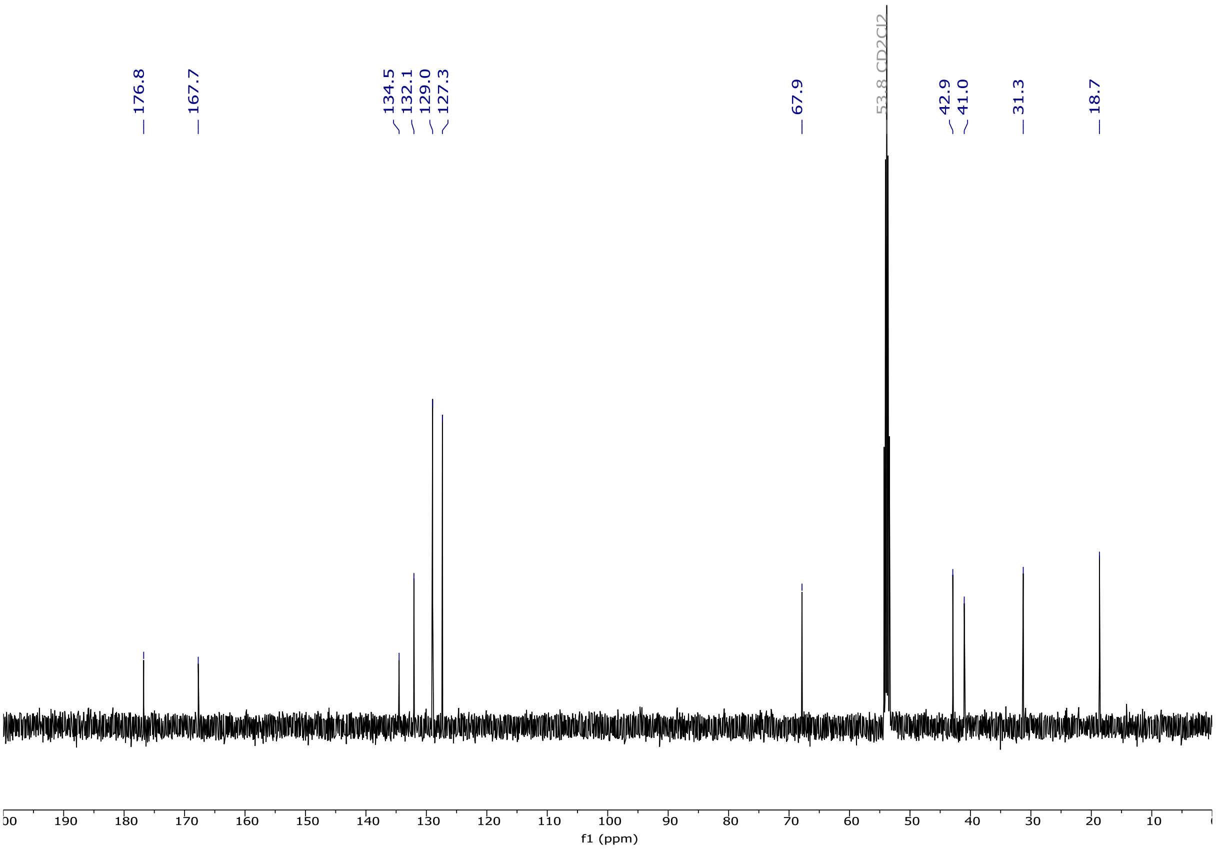

N-{2-(2-Oxopyrrolidin-1-yl)-2-(1H-pyrazol-1-yl)ethyl}benzamide (5h)

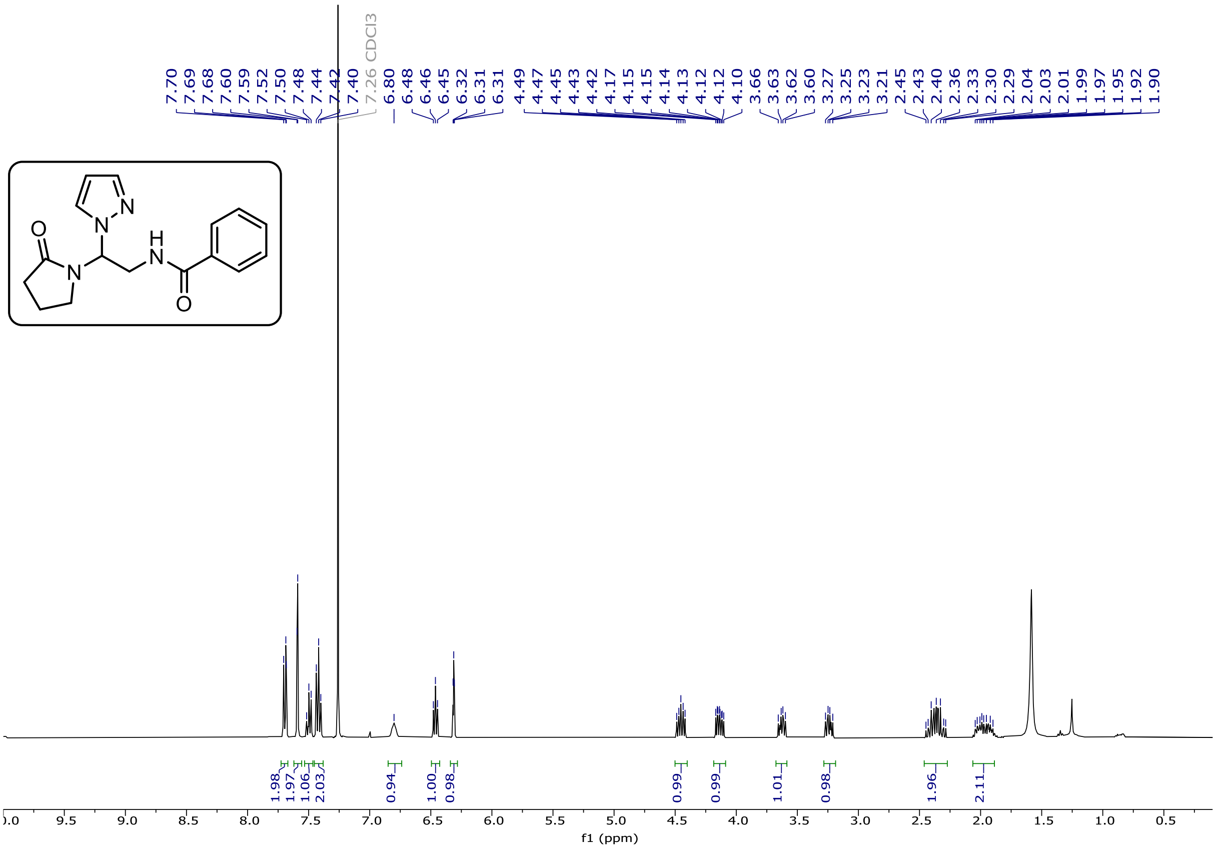

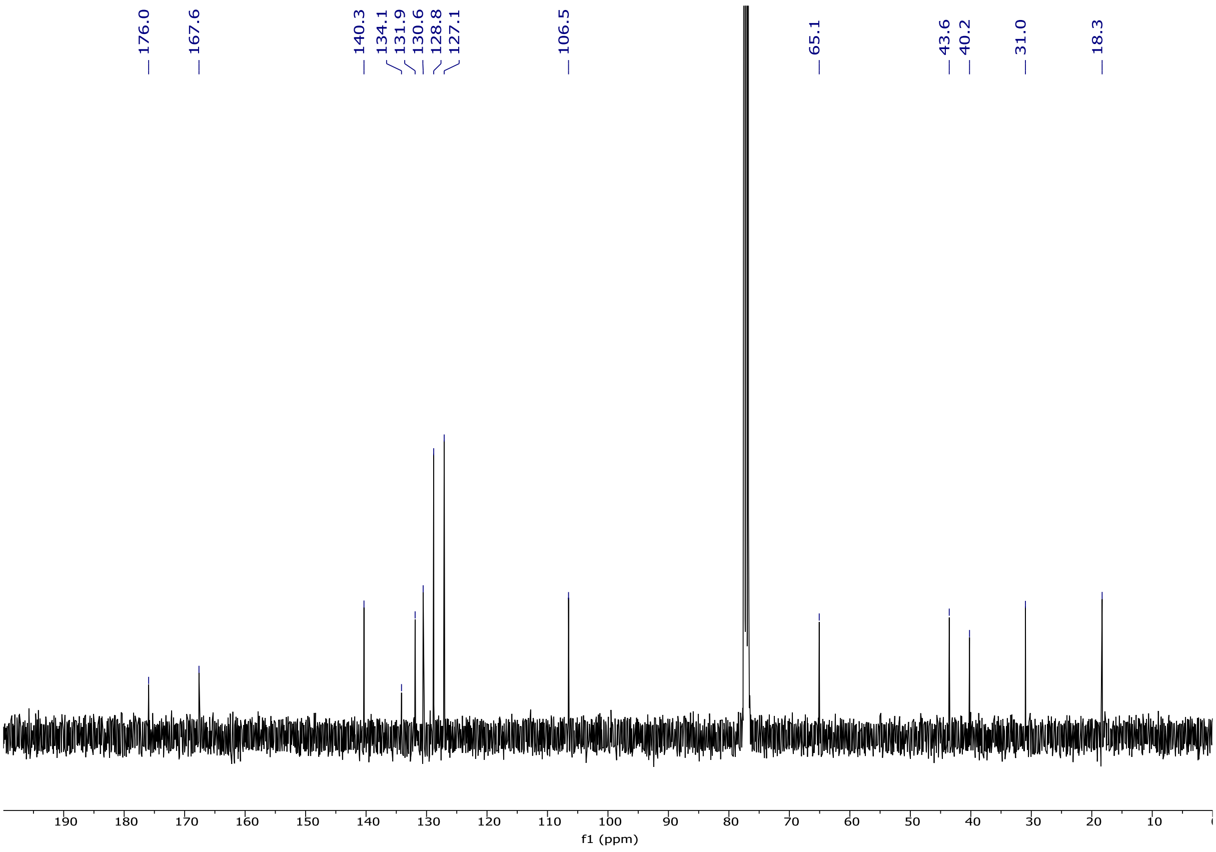

_N_-{2-(4-Iodo-1_H_-pyrazol-1-yl)-2-(2-oxopyrrolidin-1-yl)ethyl}benzamide (5i)

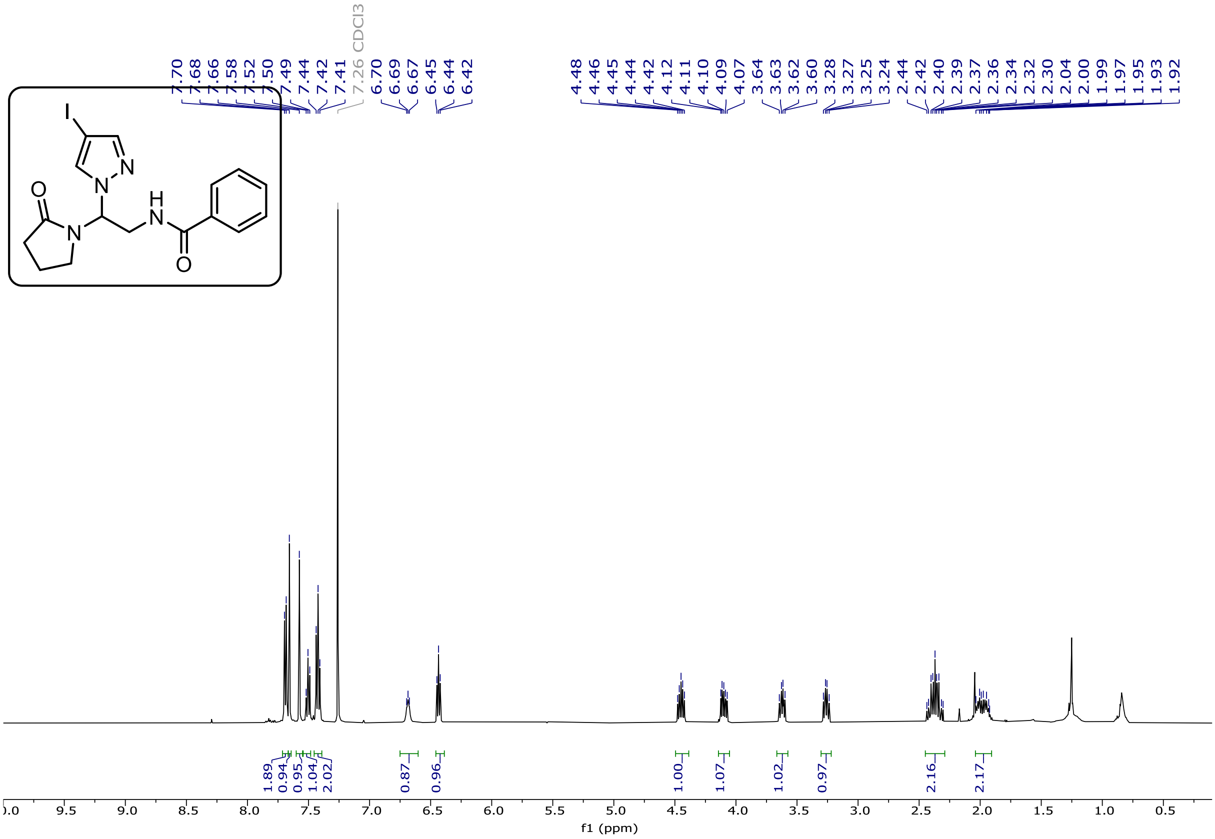

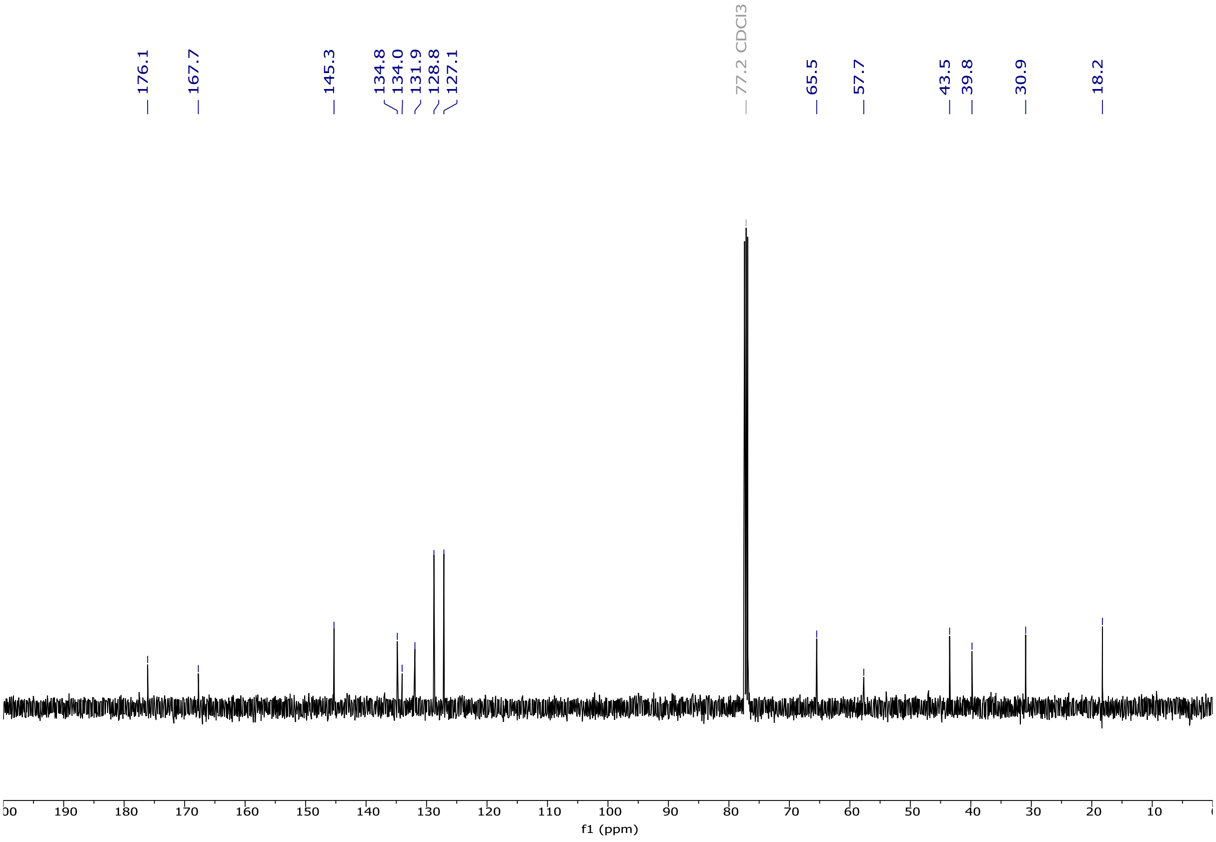

N-{2-(3,5-Dimethyl-1H-pyrazol-1-yl)-2-(2-oxopyrrolidin-1-yl)ethyl}benzamide (5j)

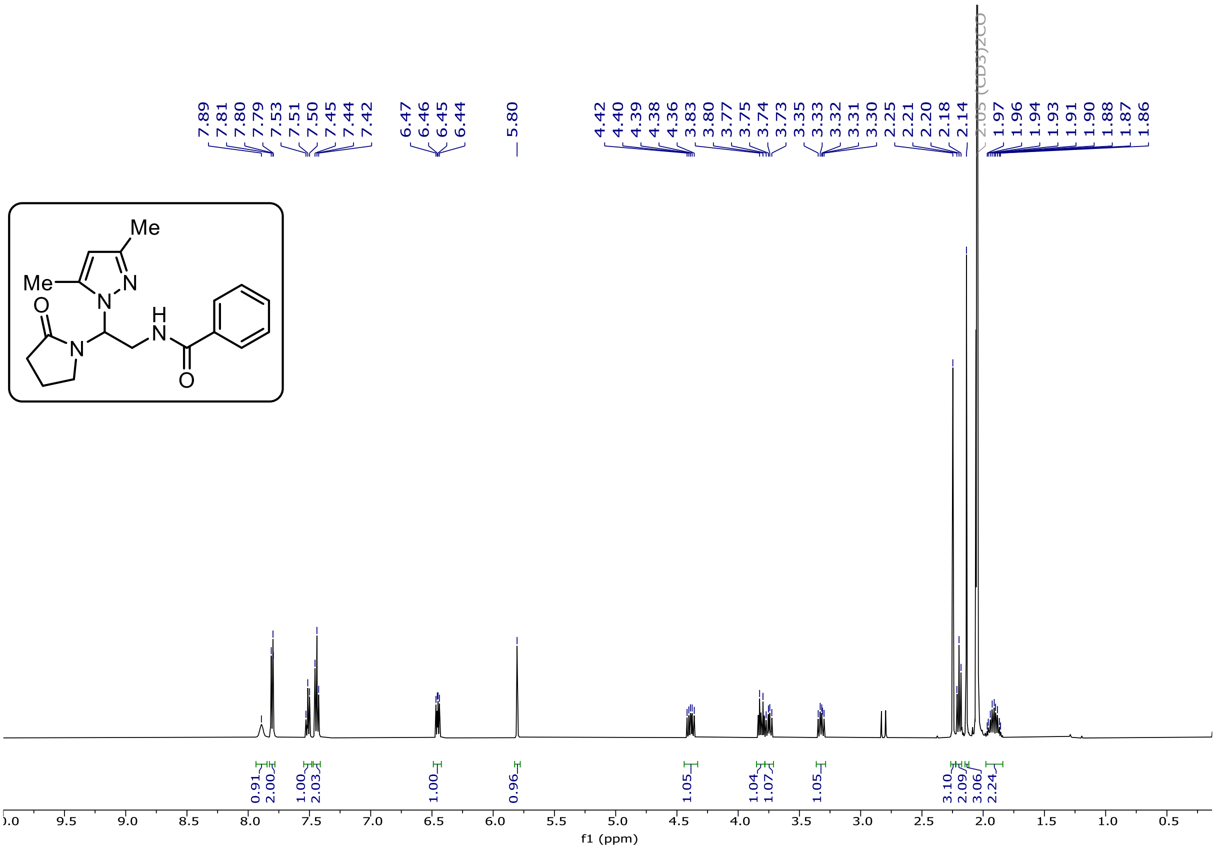

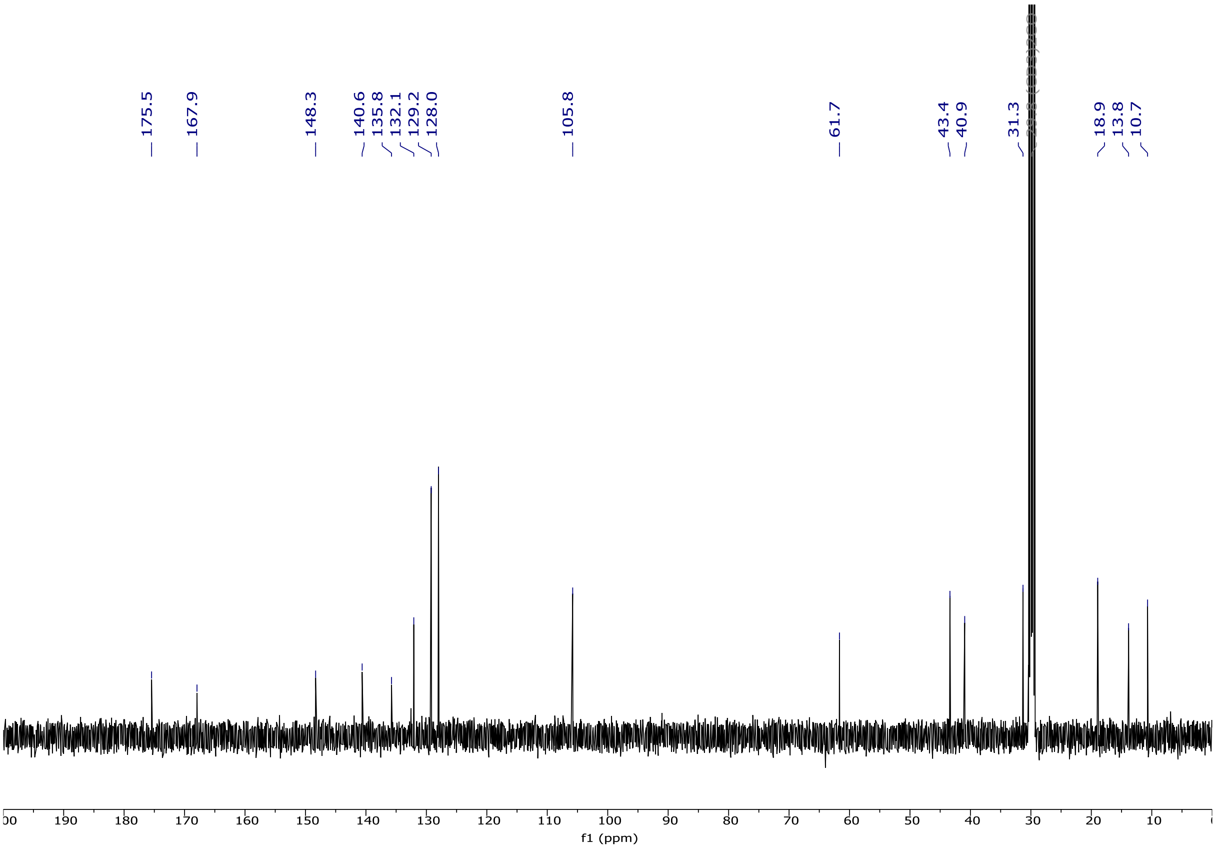

N-Phenethylbenzamide (6a)

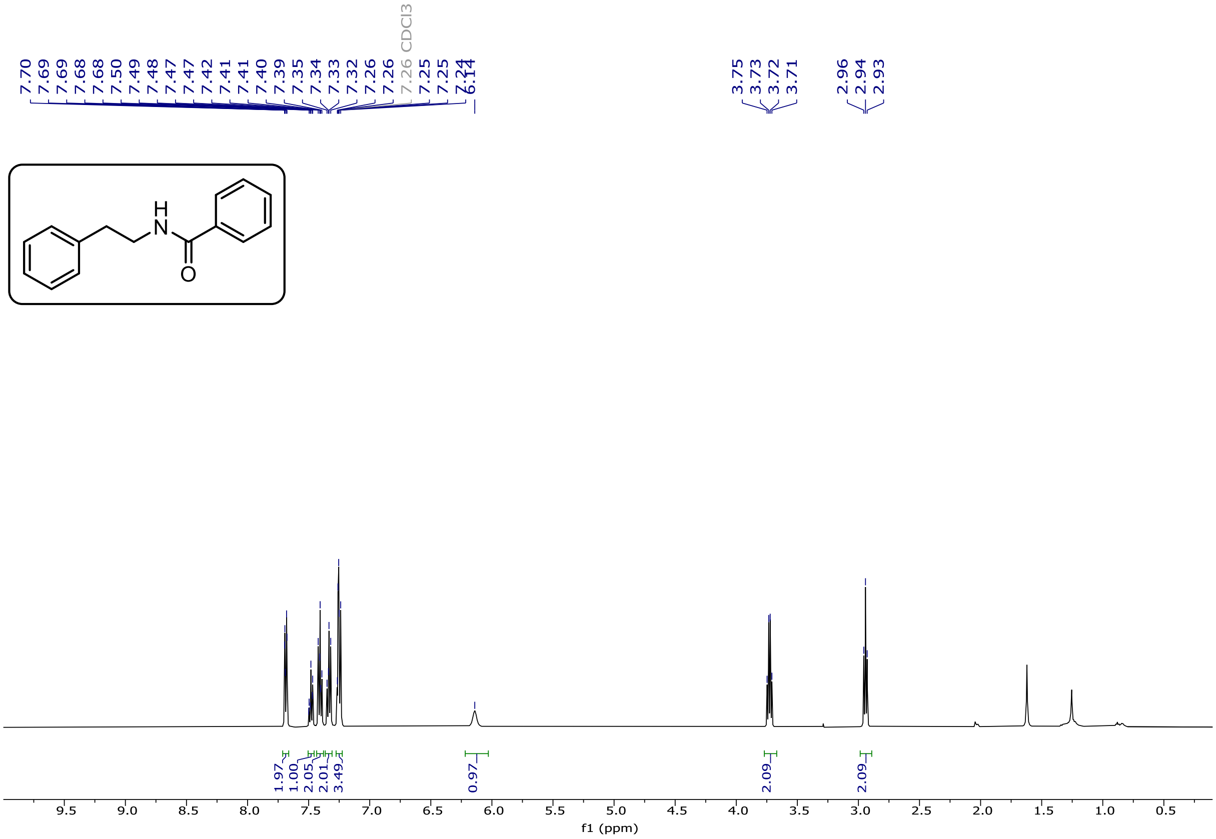

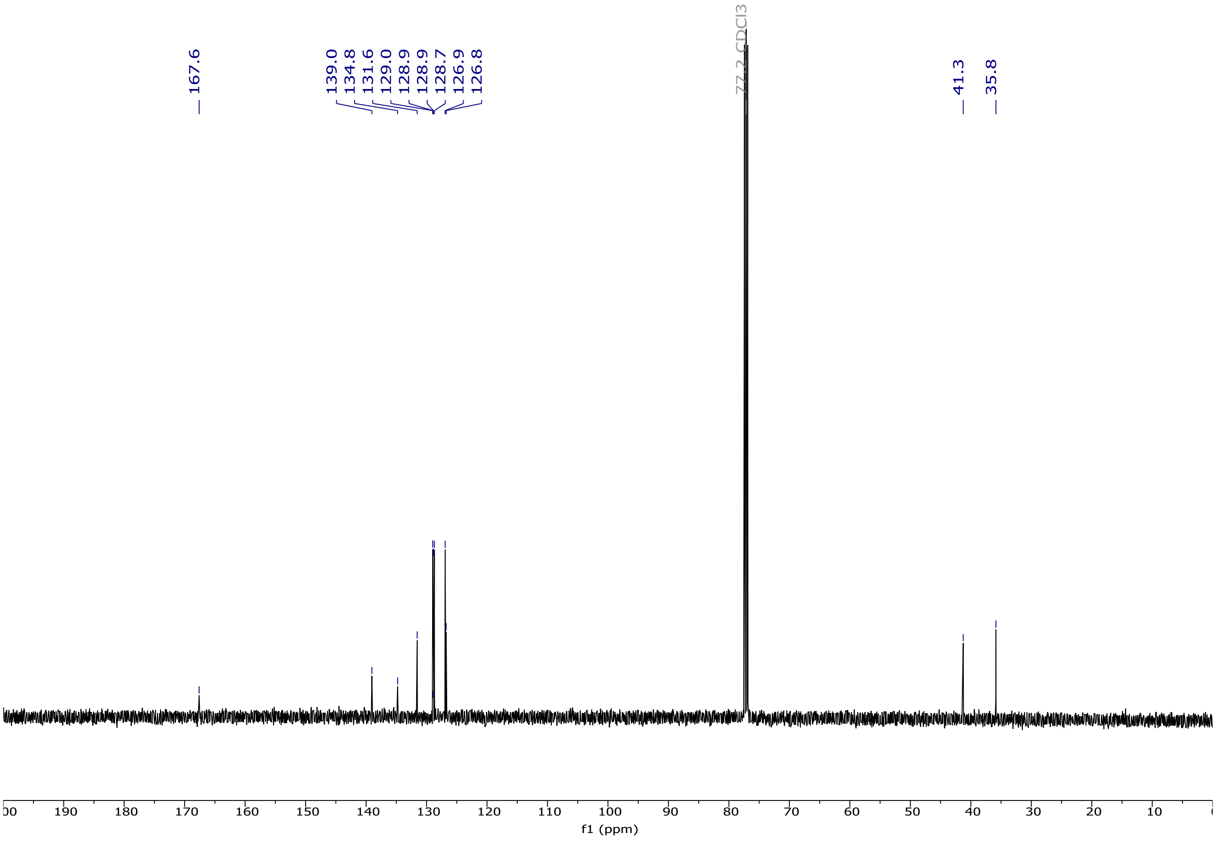

N-(4-Methylphenethyl)benzamide (6b)

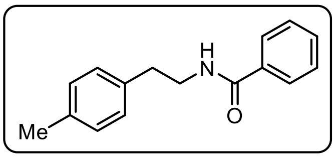

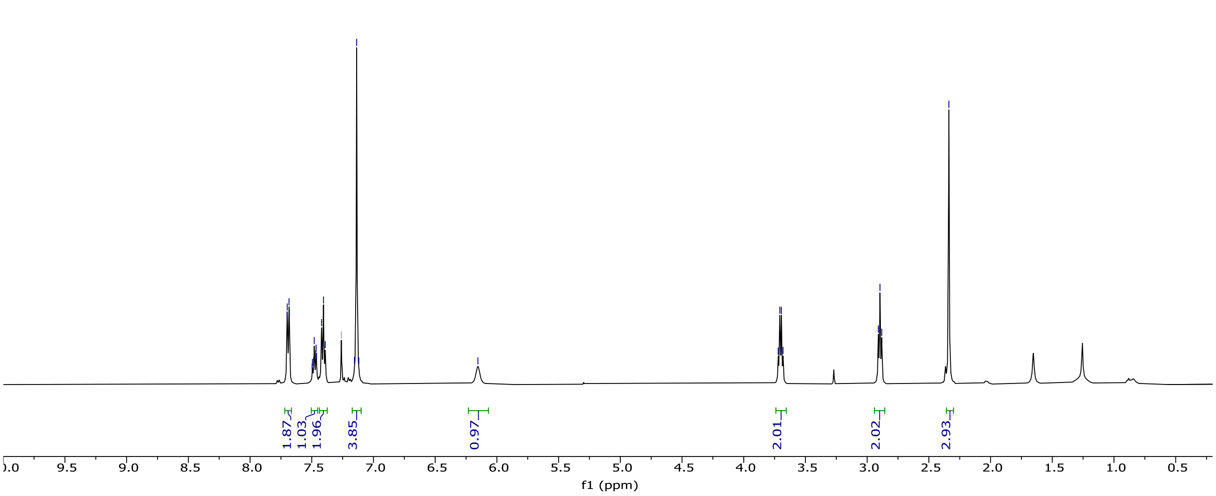

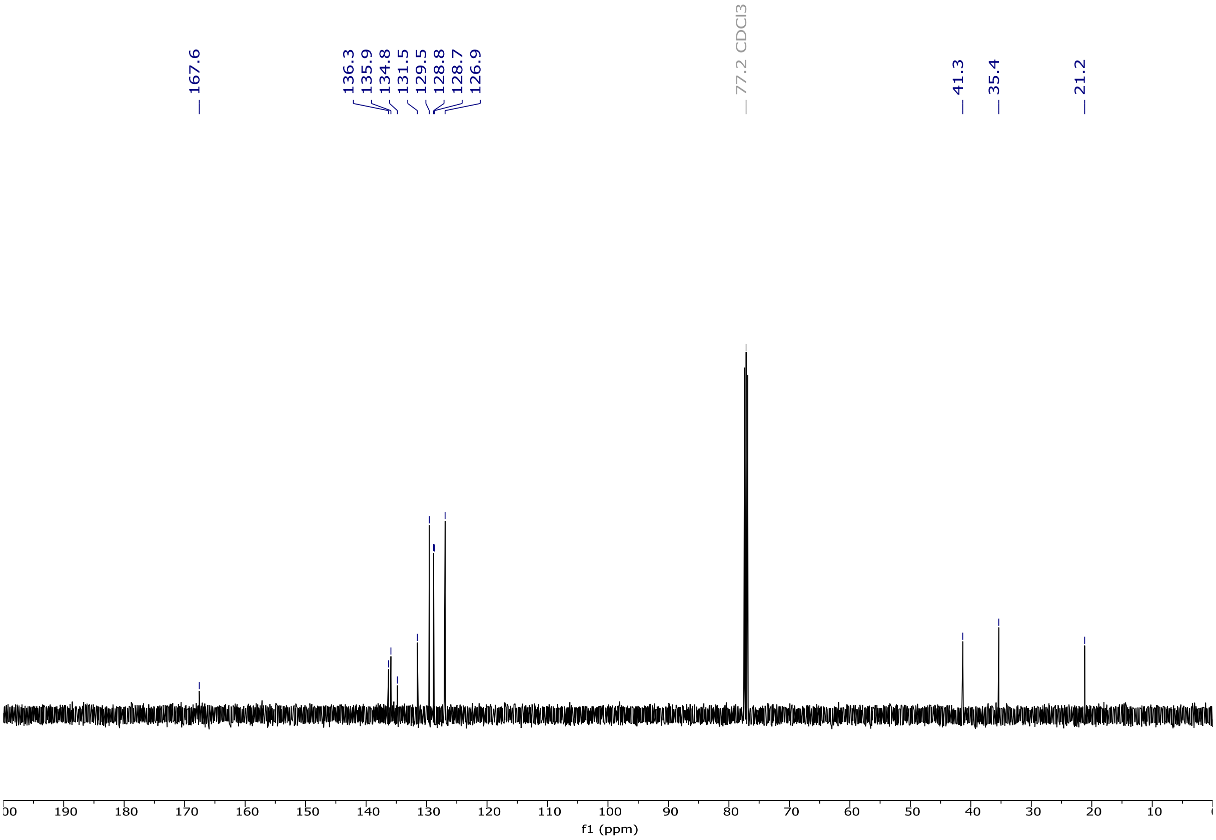

***N*-{4-(*tert*-Butyl)phenethyl}benzamide (6c)**

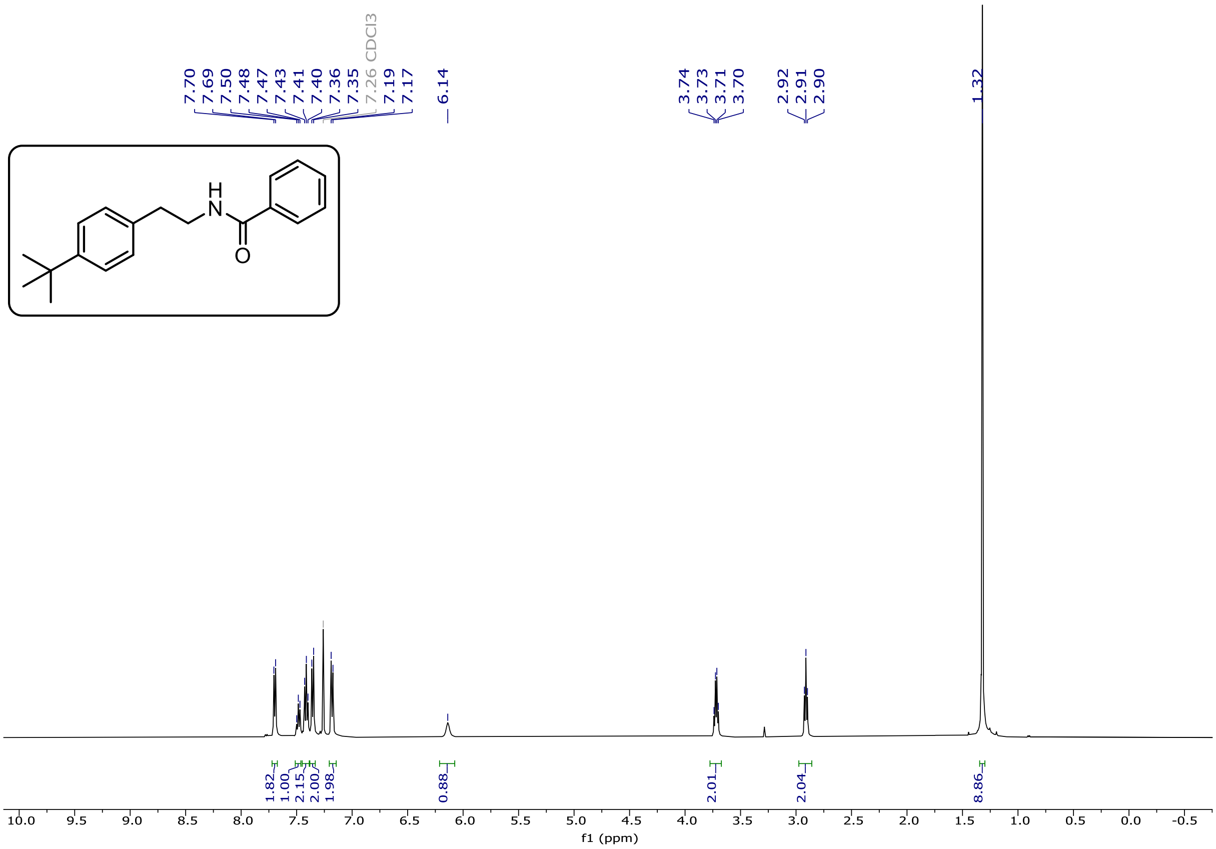

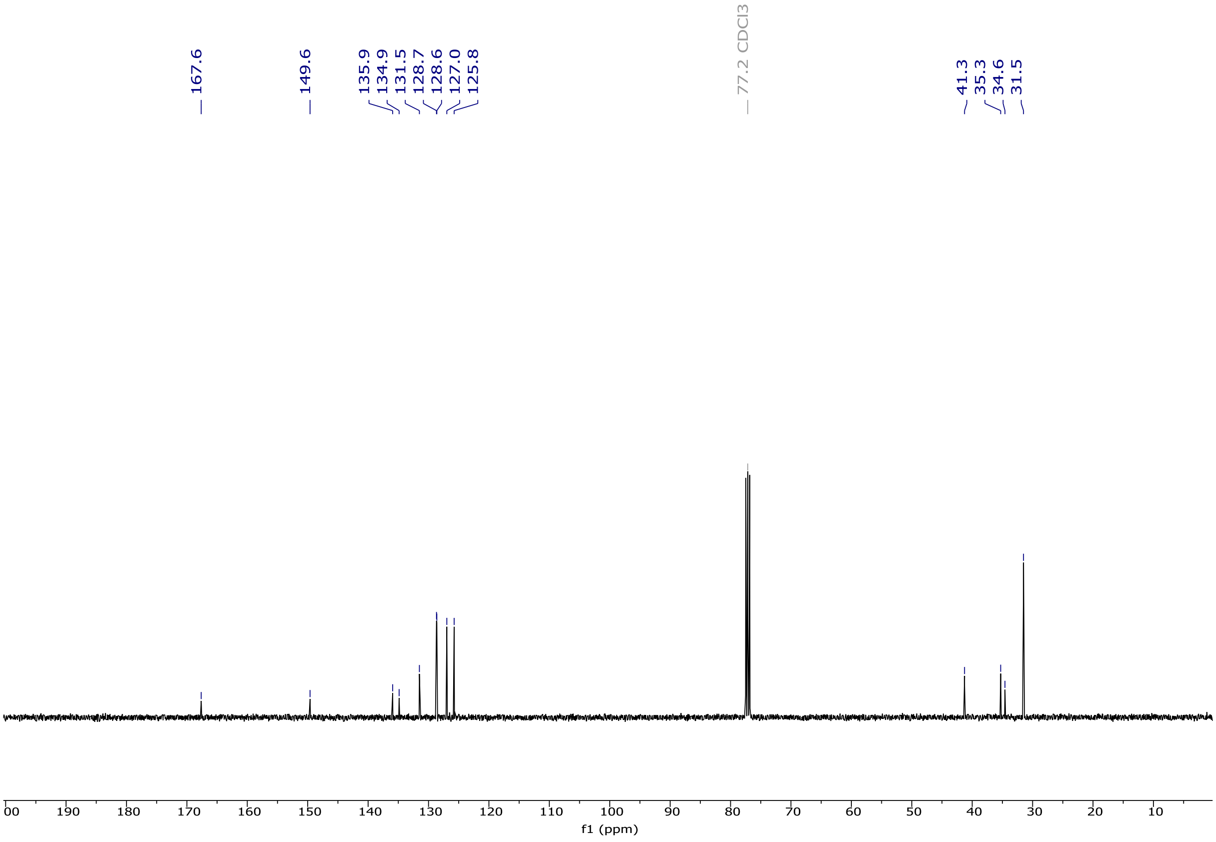

N-(4-Chlorophenethyl)benzamide (6d)

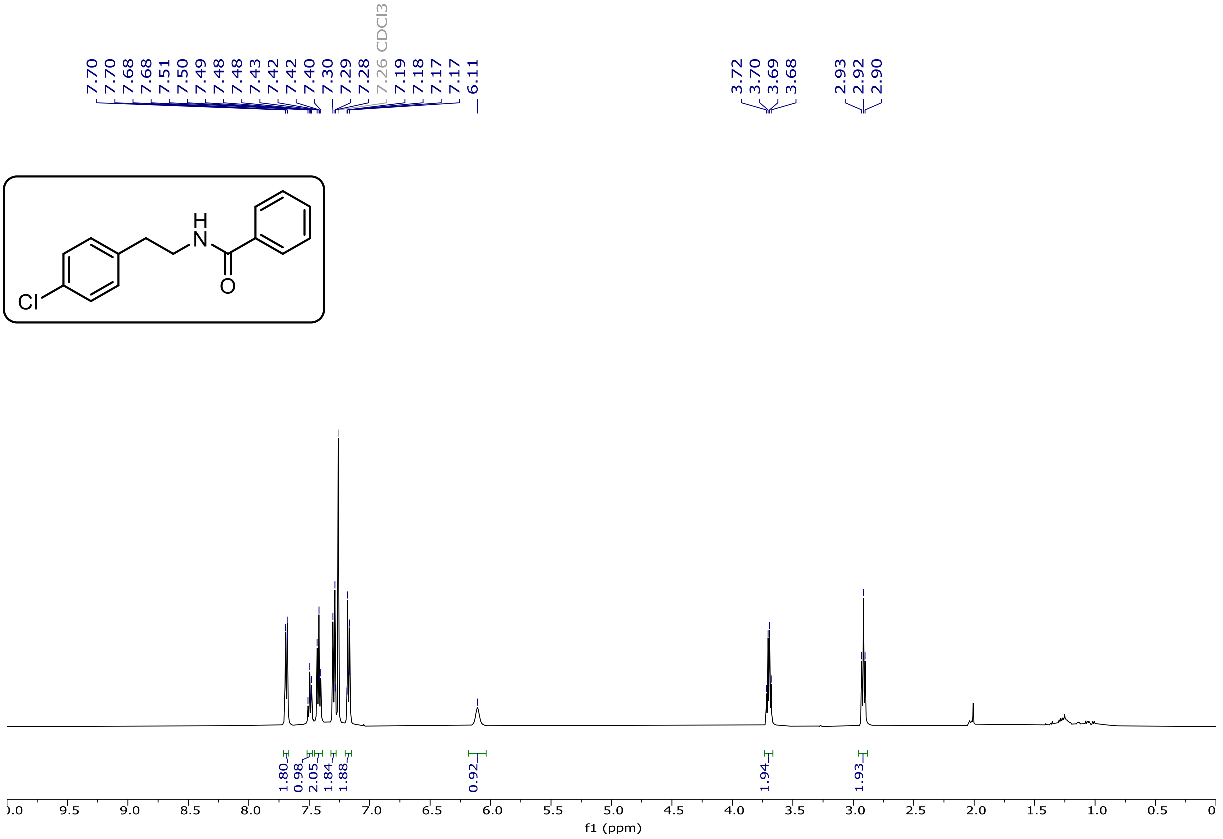

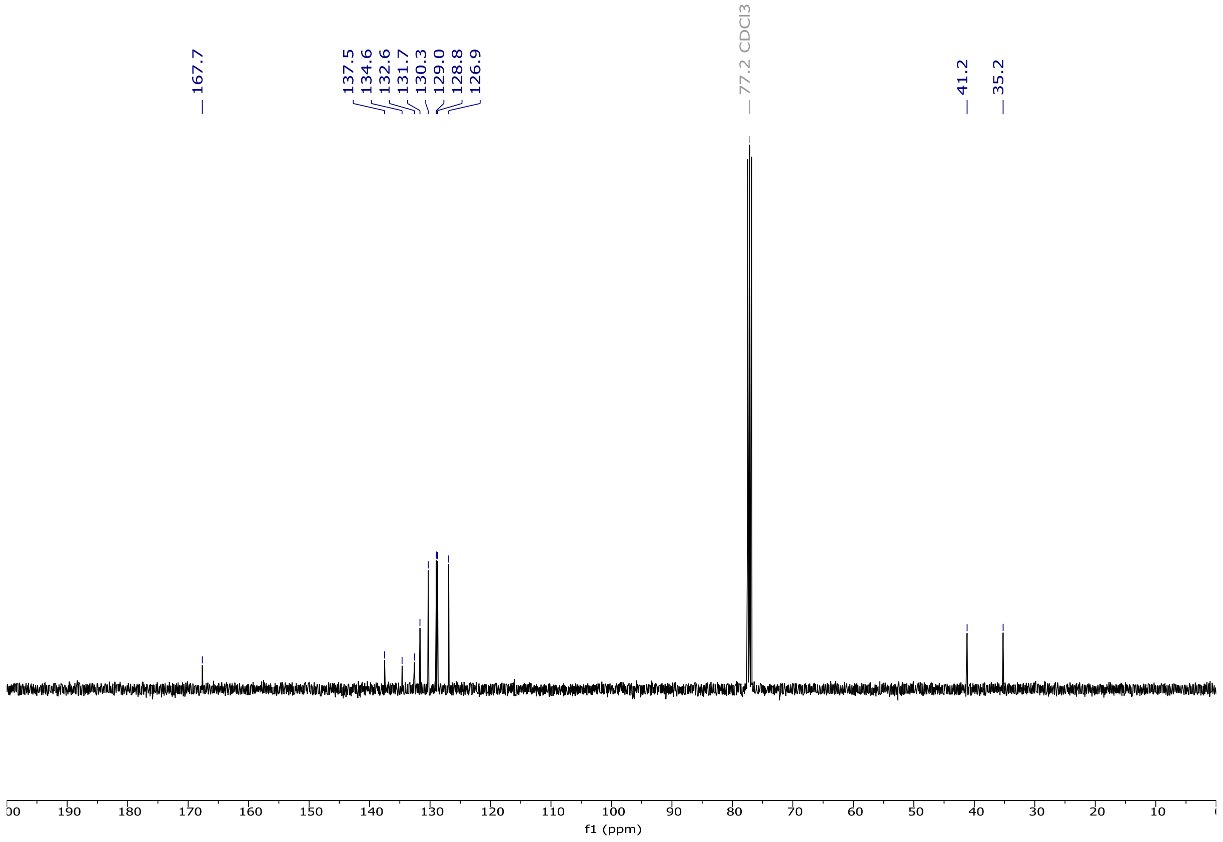

N-(4-Methoxyphenethyl)benzamide (6e)

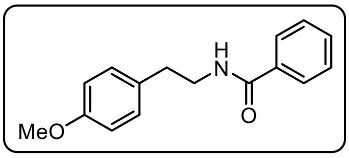

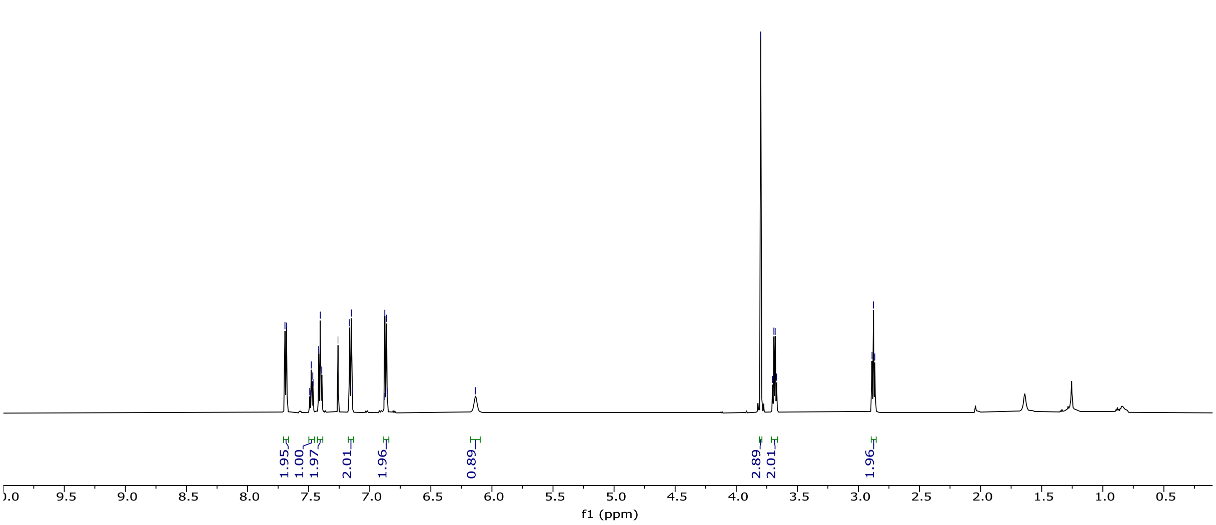

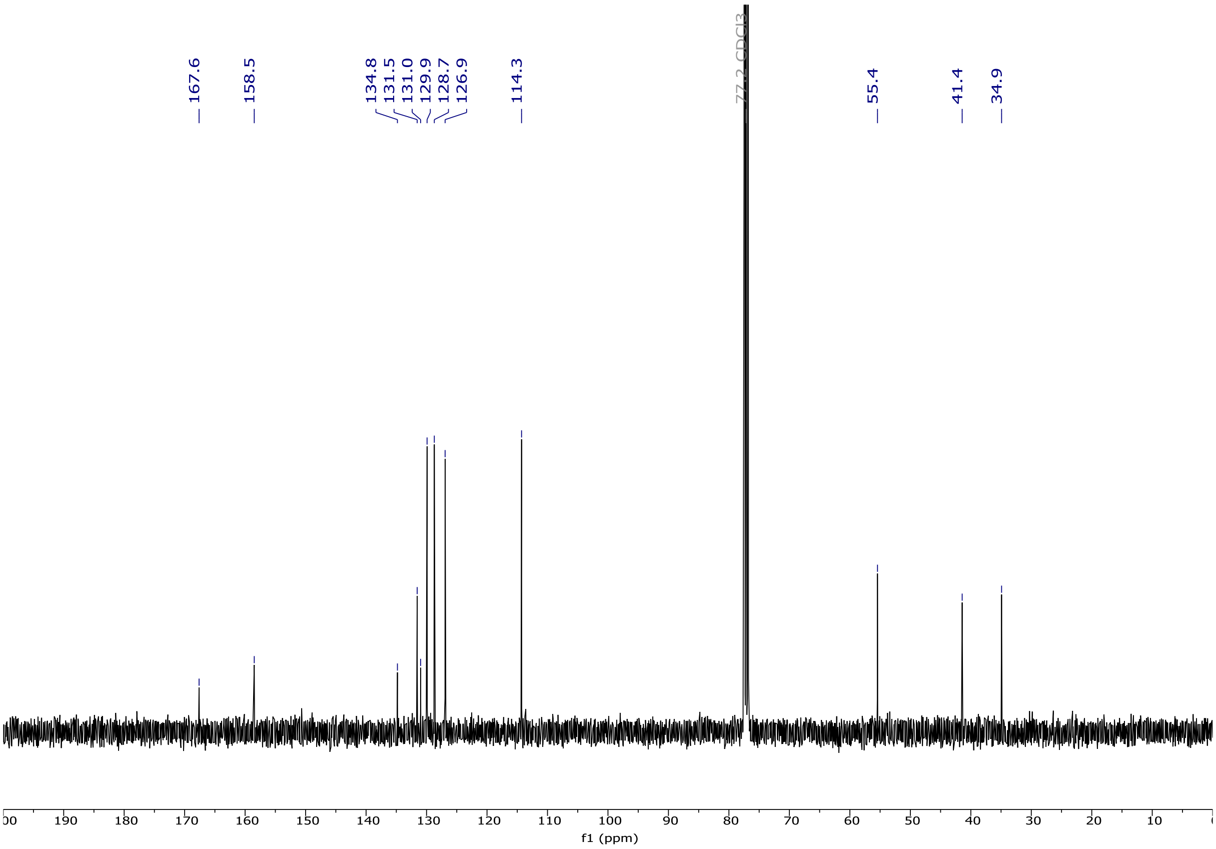

N-{2-(Benzo[*d*][1,3]dioxol-5-yl)ethyl}benzamide (6f)

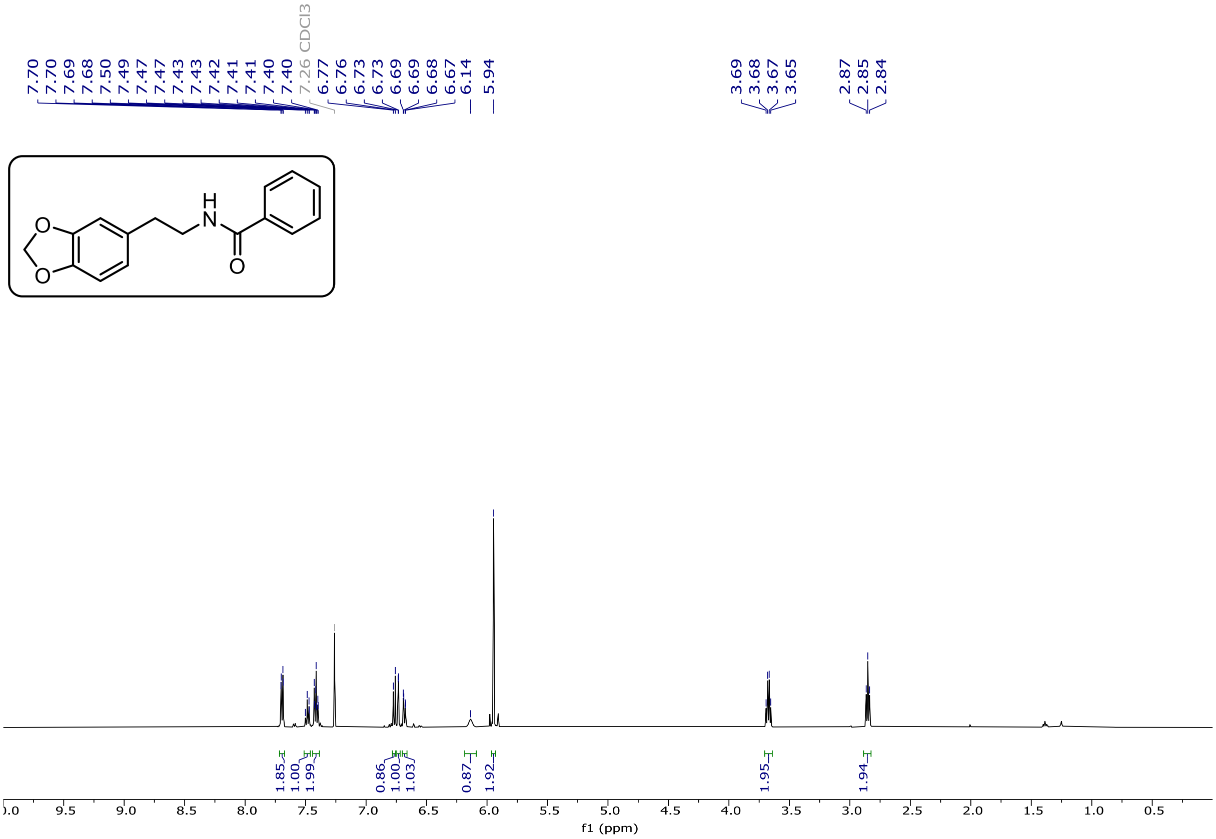

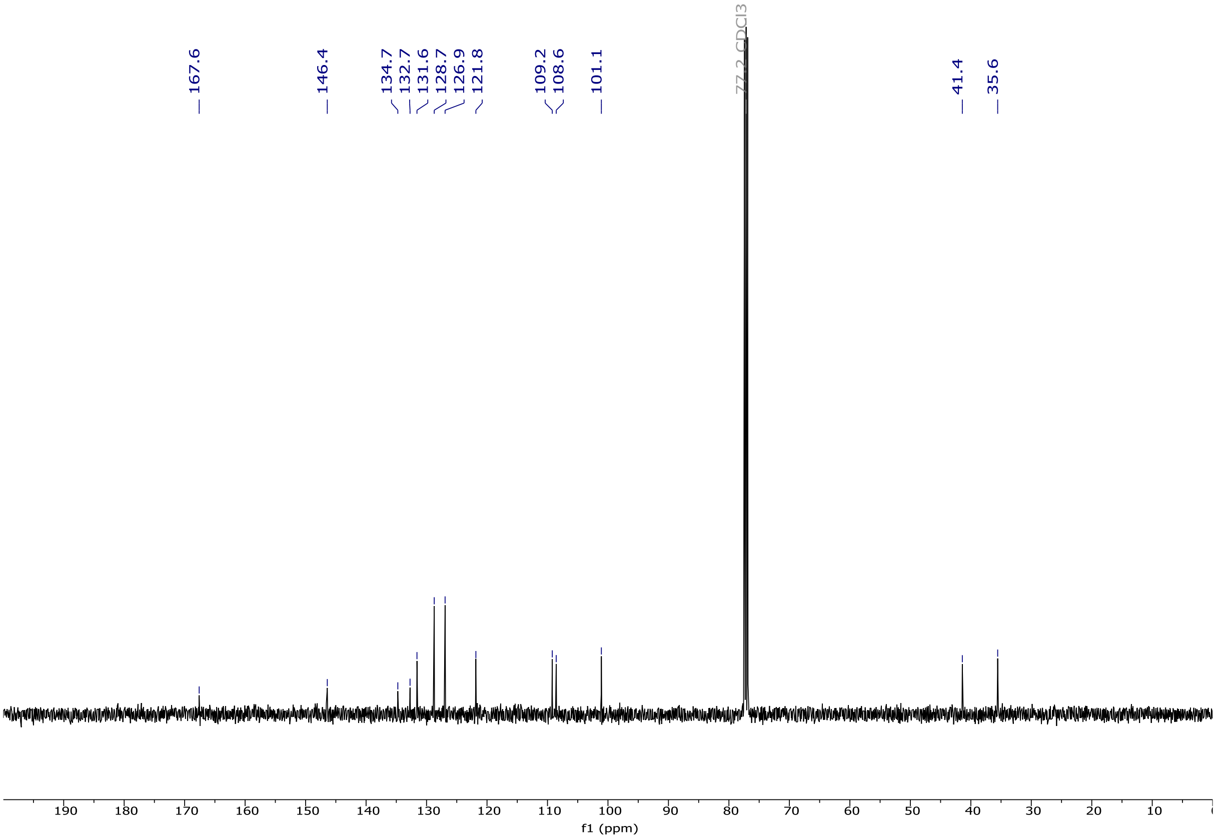

N-(2-Methylphenethyl)benzamide (6g)

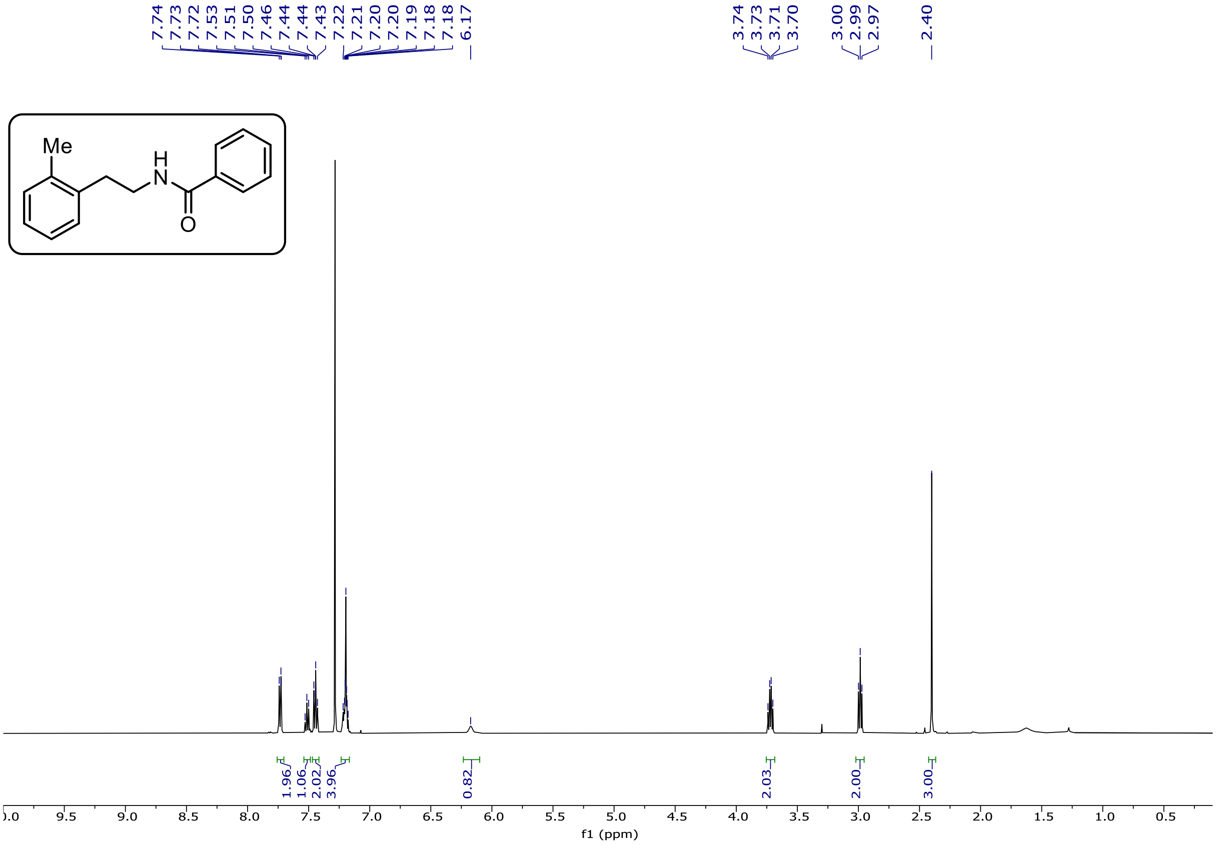

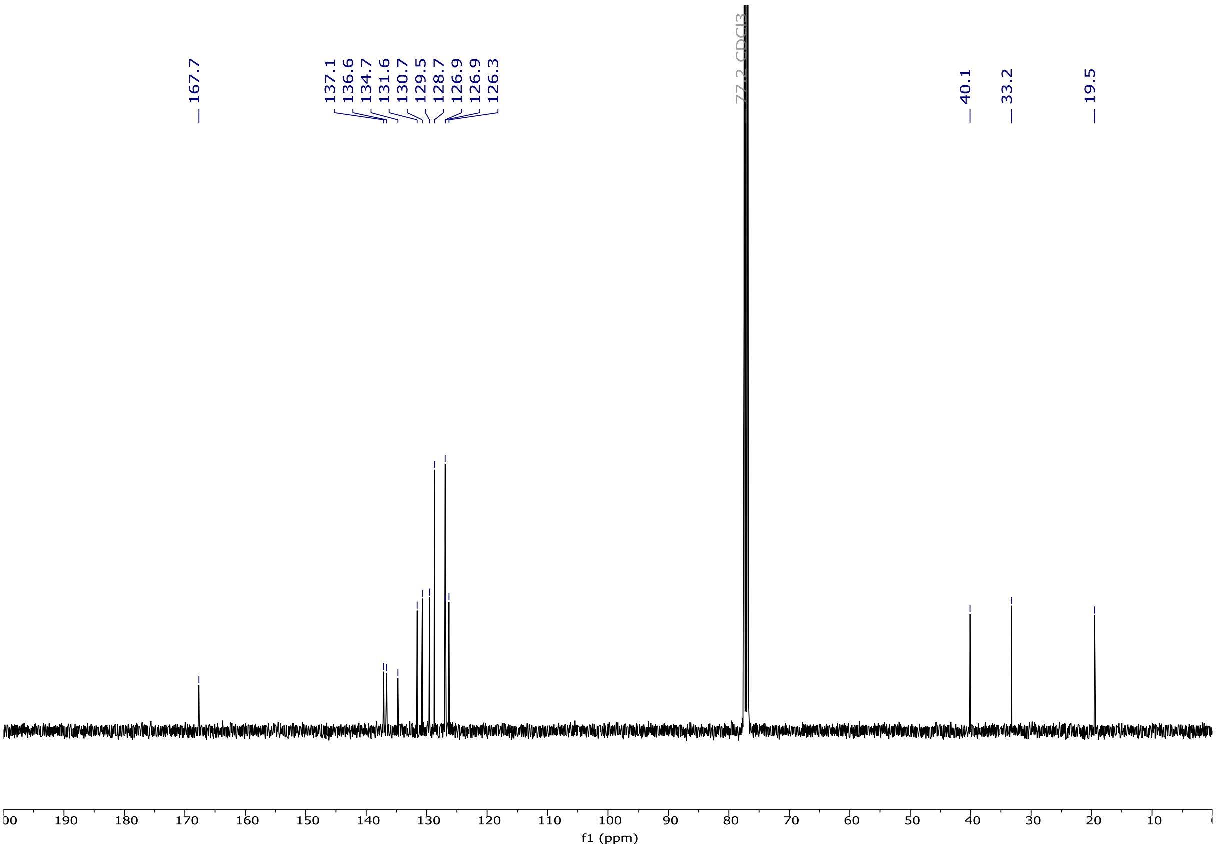

***N*-{1-(4-Methoxyphenyl)propan-2-yl}benzamide (6h)**

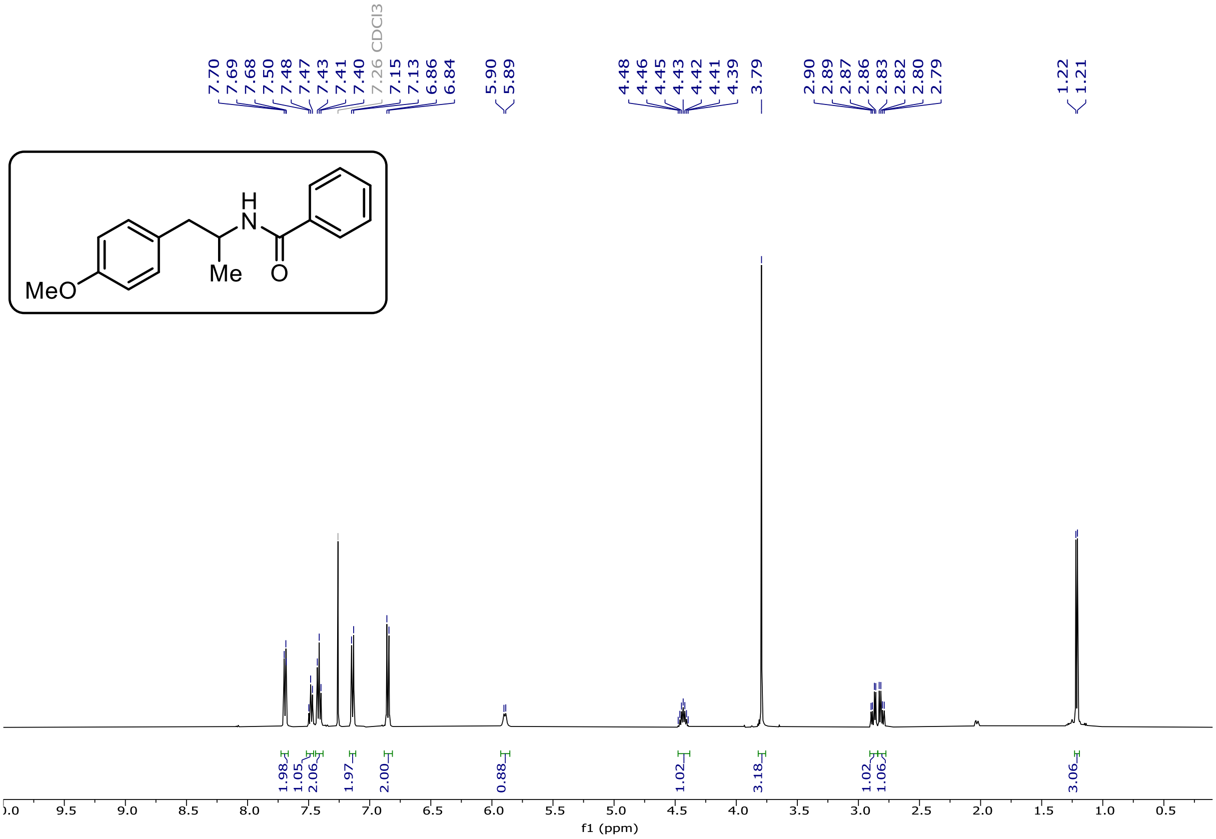

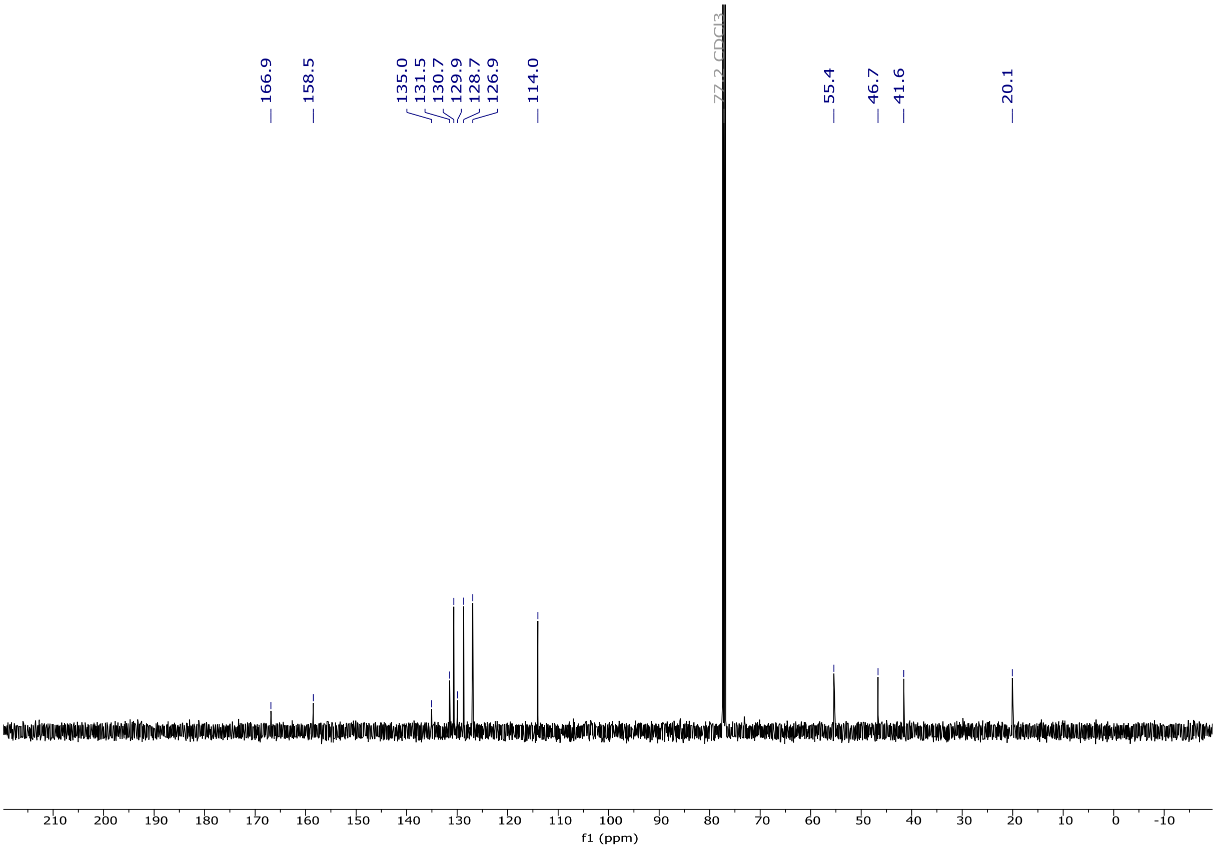

N-{2-(Ethylthio)ethyl}benzamide (6i)

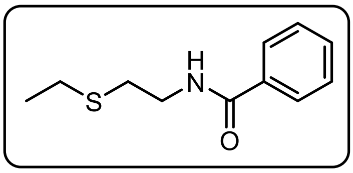

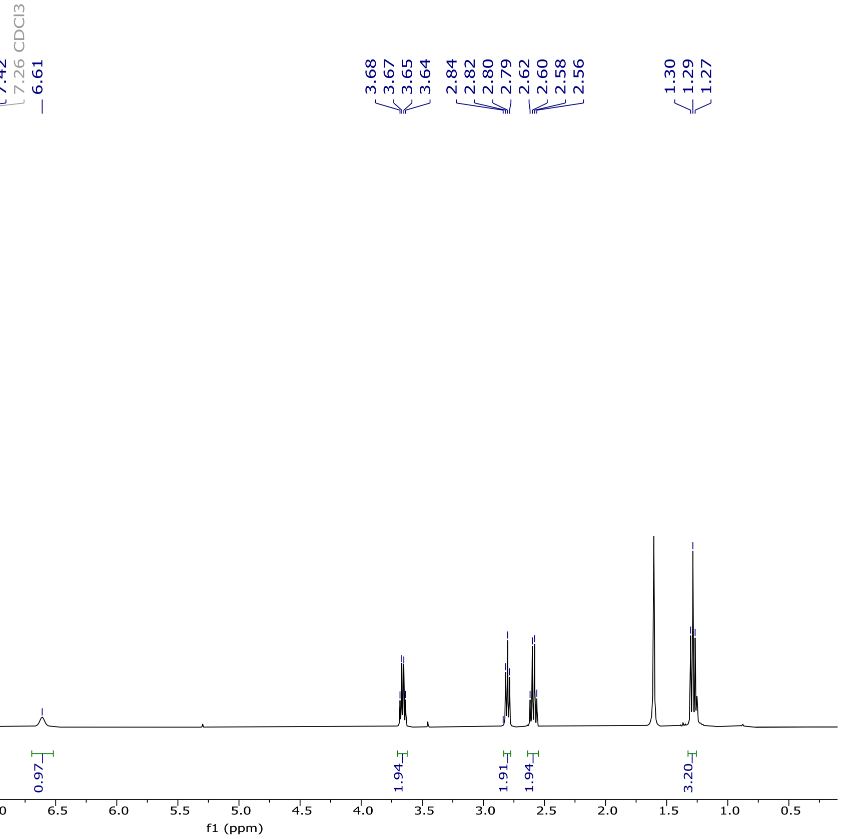

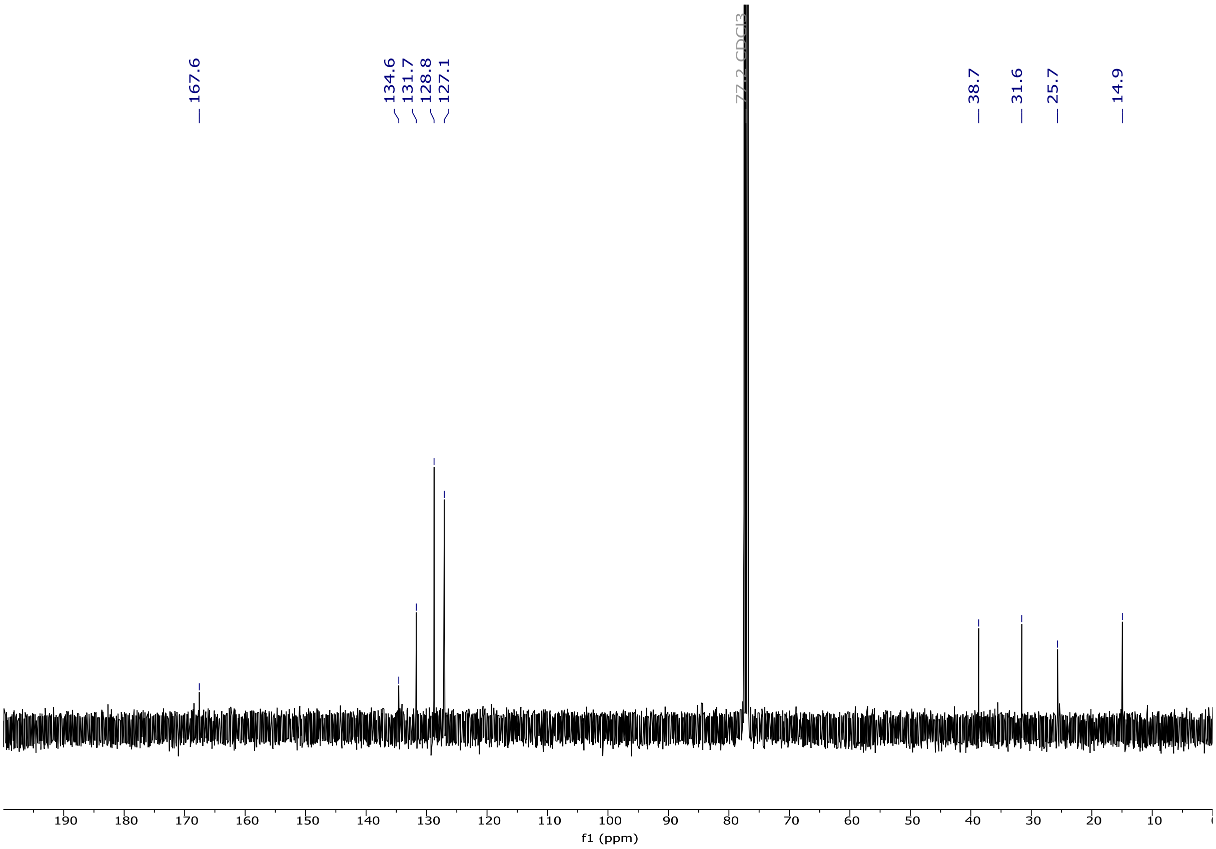

N-{2-(Phenylthio)ethyl}benzamide (6j)

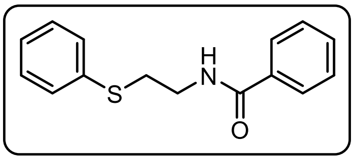

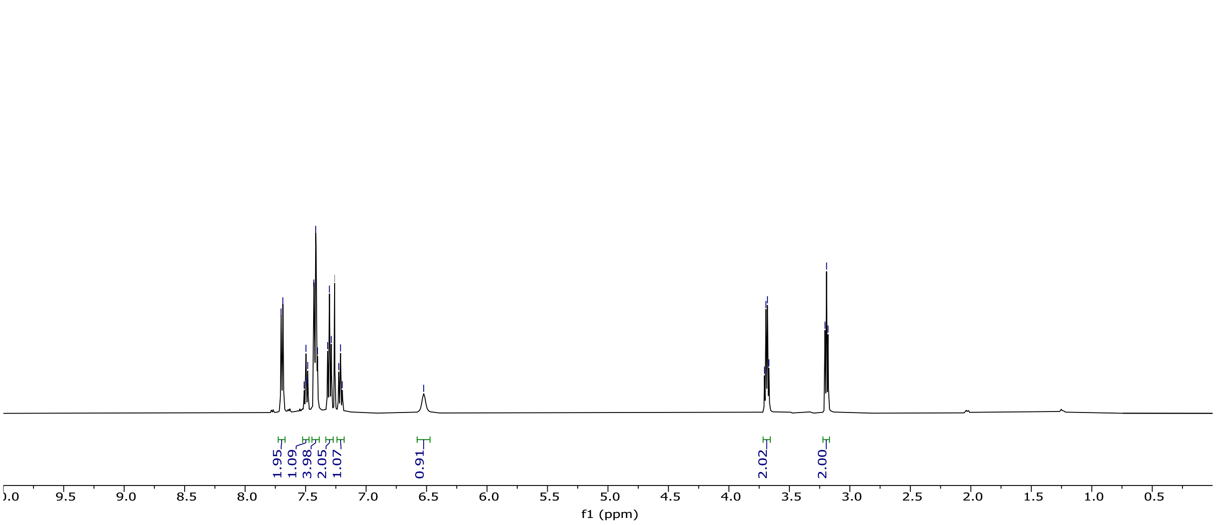

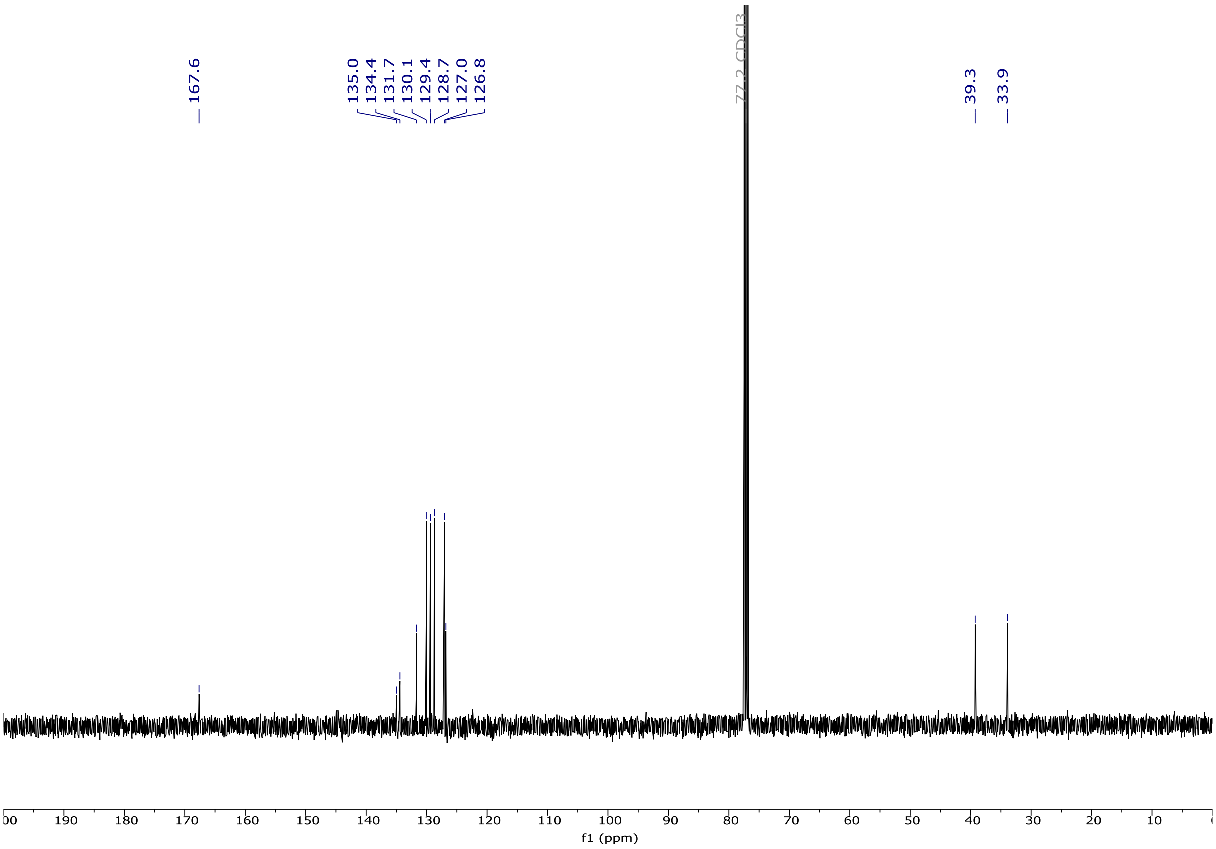

N-{2,2-Bis(phenylsulfonyl)ethyl}benzamide (6k)

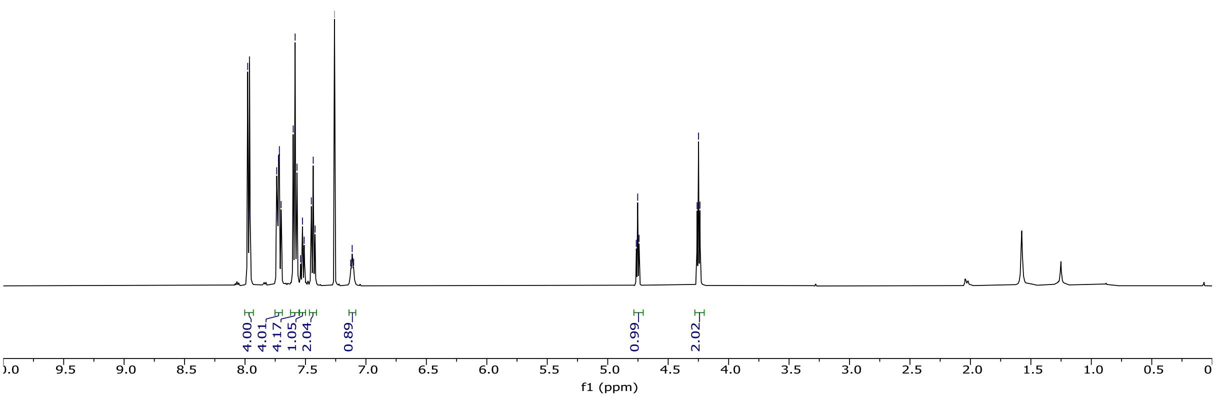

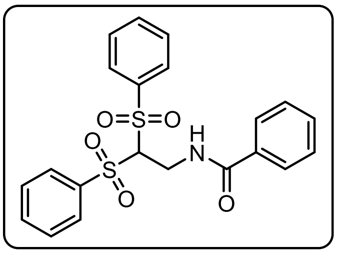

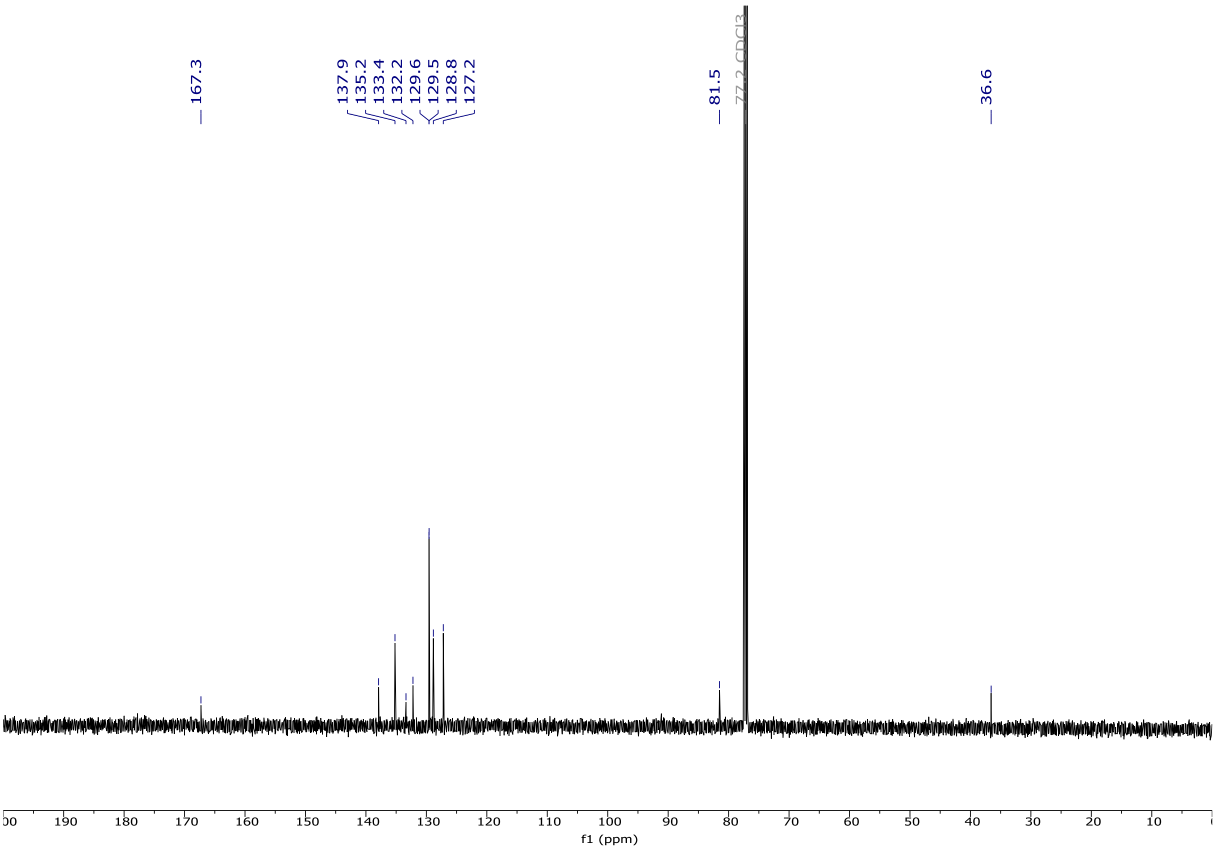

VIII. Crystallographic Data

Crystal data and structure refinement for major diastereomer of **4m** (CCDC 2428730)

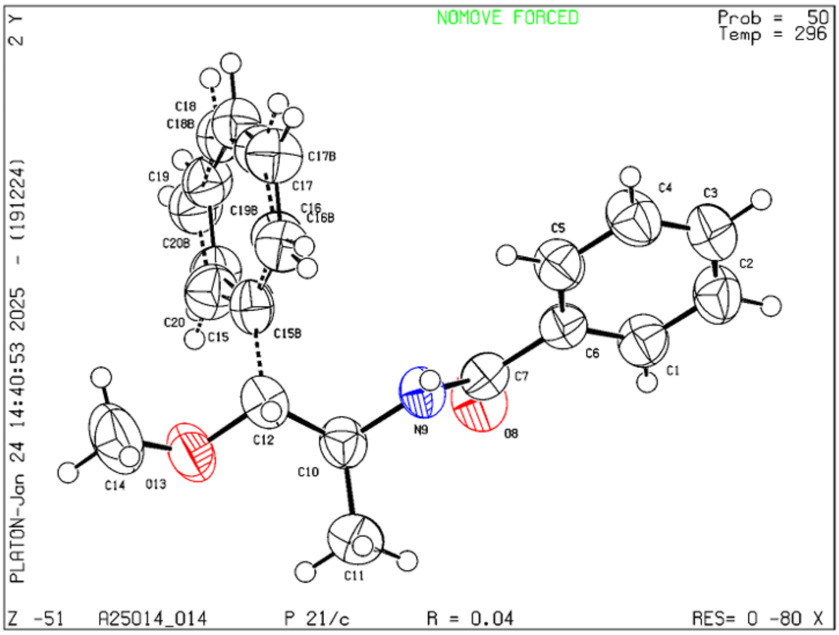

Empirical formula	$C_{17} H_{19} N O_2$	
Formula weight	269.33	
Temperature	296(2) K	
Wavelength	0.71073 Å	
Crystal system	Monoclinic	
Space group	$P2_1/c$	
Unit cell dimensions	a = 17.1827(8) Å	α = 90°
	b = 5.2241(2) Å	β = 99.1338(14)°
	c = 16.8261(7) Å	γ = 90°
Volume	1491.23(11) $Å^3$	
Z	4	
Density (calculated)	1.200 Mg/m^3	
Absorption coefficient	0.078 mm^{-1}	
F(000)	576	
Crystal size	0.162 x 0.025 x 0.023 mm^3	
Theta range for data collection	2.401 to 27.007°.	
Index ranges	–21<=h<=21, –6<=k<=6, –21<=l<=16	
Reflections collected	25417	
Independent reflections	3258 [R(int) = 0.1005]	
Completeness to theta = 25.242°	100.0 %	
Absorption correction	Semi-empirical from equivalents	
Max. and min. transmission	0.7455 and 0.7187	
Refinement method	Full-matrix least-squares on F^2	
Data / restraints / parameters	3258 / 252 / 223	
Goodness-of-fit on F^2	1.016	
Final R indices [I>2sigma(I)]	R1 = 0.0449, wR2 = 0.1135	
R indices (all data)	R1 = 0.0869, wR2 = 0.1360	
Largest diff. peak and hole	0.138 and –0.127 e·$Å^{-3}$	

Crystal data and structure refinement for major diastereomer of **4s** (CCDC 2428731)

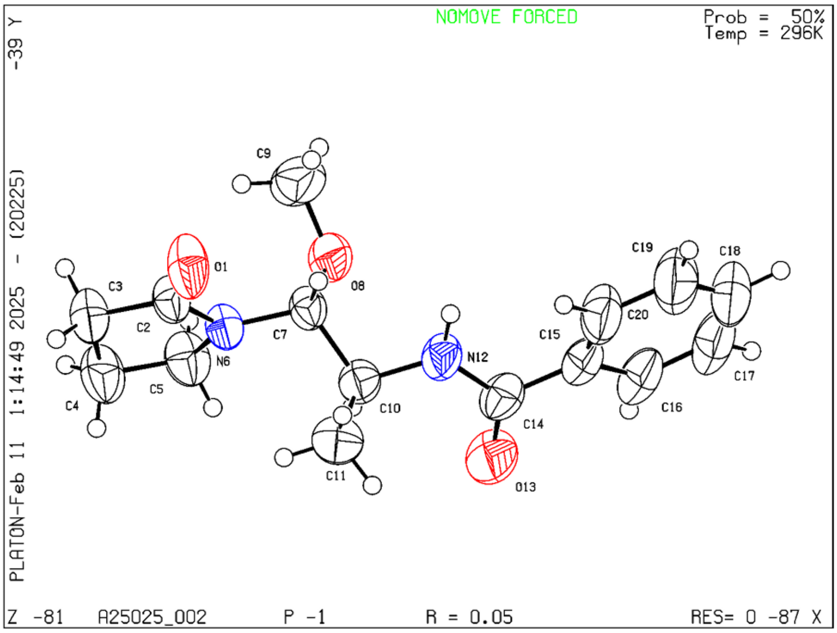

Empirical formula	$C_{15}H_{20}N_2O_3$	
Formula weight	276.33	
Temperature	296(2) K	
Wavelength	0.71073 Å	
Crystal system	Triclinic	
Space group	P-1	
Unit cell dimensions	a = 7.9210(2) Å	α = 96.7451(10)°
	b = 9.6187(3) Å	β = 103.5952(10)°
	c = 10.9640(3) Å	γ = 111.0918(11)°
Volume	738.50(4) $Å^3$	
Z	2	
Density (calculated)	1.243 Mg/m^3	
Absorption coefficient	0.087 mm^{-1}	
F(000)	296	
Crystal size	0.152 x 0.114 x 0.057 mm^3	
Theta range for data collection	2.328 to 27.984°.	
Index ranges	–10<=h<=10, –12<=k<=12, –14<=l<=14	
Reflections collected	25039	
Independent reflections	3565 [R(int) = 0.0372]	
Completeness to theta = 25.242°	99.9 %	
Absorption correction	Semi-empirical from equivalents	
Max. and min. transmission	0.7456 and 0.7130	
Refinement method	Full-matrix least-squares on F^2	
Data / restraints / parameters	3565 / 0 / 186	
Goodness-of-fit on F^2	1.064	
Final R indices [I>2sigma(I)]	R1 = 0.0516, wR2 = 0.1518	
R indices (all data)	R1 = 0.0668, wR2 = 0.1648	
Largest diff. peak and hole	0.198 and –0.161 e·$Å^{-3}$	

Crystal data and structure refinement of **5h** (CCDC 2428732)

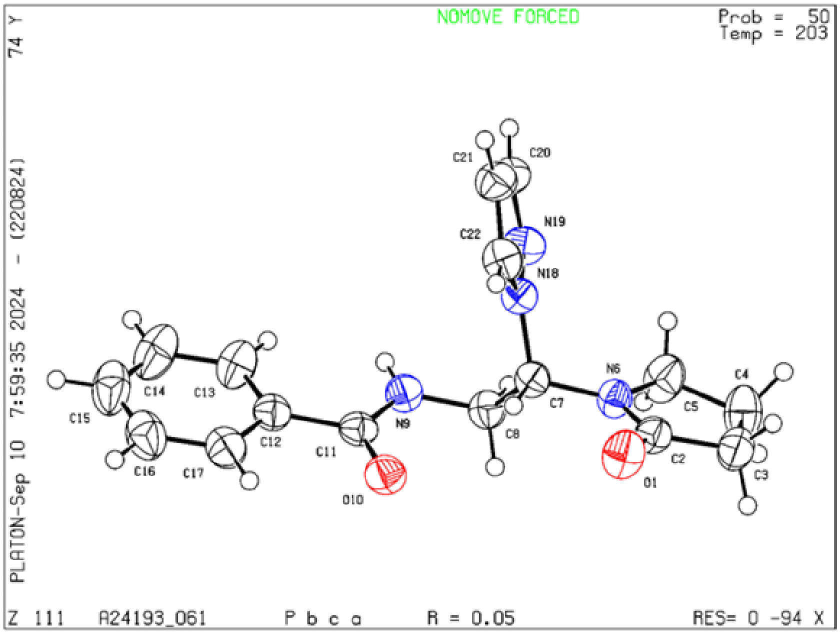

Empirical formula	$C_{16}H_{18}N_4O_2$	
Formula weight	298.34	
Temperature	203(2) K	
Wavelength	0.71073 Å	
Crystal system	Orthorhombic	
Space group	*Pbca*	
Unit cell dimensions	a = 10.6944(5) Å	α = 90°
	b = 16.3834(7) Å	β = 90°
	c = 17.2431(7) Å	γ = 90°
Volume	3021.2(2) $Å^3$	
Z	8	
Density (calculated)	1.312 Mg/m^3	
Absorption coefficient	0.090 mm^{-1}	
F(000)	1264	
Crystal size	0.167 x 0.047 x 0.022 mm^3	
Theta range for data collection	2.563 to 27.025°.	
Index ranges	–13<=h<=13, –20<=k<=20, –22<=l<=14	
Reflections collected	26408	
Independent reflections	3283 [R(int) = 0.1007]	
Completeness to theta = 25.242°	99.3 %	
Absorption correction	Semi-empirical from equivalents	
Max. and min. transmission	0.7455 and 0.7061	
Refinement method	Full-matrix least-squares on F^2	
Data / restraints / parameters	3283 / 0 / 205	
Goodness-of-fit on F^2	1.040	
Final R indices [I>2sigma(I)]	R1 = 0.0513, wR2 = 0.1226	
R indices (all data)	R1 = 0.0797, wR2 = 0.1447	
Largest diff. peak and hole	0.237 and –0.214 e·$Å^{-3}$	

Crystal data and structure refinement of **6h** (CCDC 2428736)

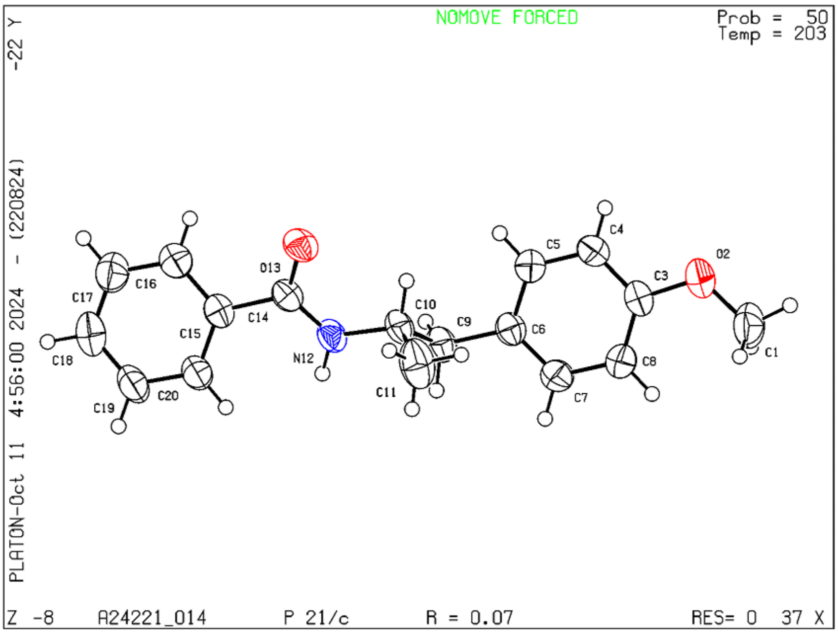

Empirical formula	$C_{17} H_{19} N O_2$	
Formula weight	269.33	
Temperature	203(2) K	
Wavelength	0.71073 Å	
Crystal system	Monoclinic	
Space group	$P2_1/c$	
Unit cell dimensions	a = 29.8217(15) Å	α = 90°
	b = 5.5779(3) Å	β = 97.386(3)°
	c = 9.1072(4) Å	γ = 90°
Volume	1502.34(13) $Å^3$	
Z	4	
Density (calculated)	1.191 Mg/m^3	
Absorption coefficient	0.078 mm^{-1}	
F(000)	576	
Crystal size	0.153 x 0.112 x 0.015 mm^3	
Theta range for data collection	2.755 to 26.964°.	
Index ranges	–37<=h<=38, –7<=k<=7, –11<=l<=11	
Reflections collected	16134	
Independent reflections	3255 [R(int) = 0.1072]	
Completeness to theta = 25.242°	99.6 %	
Absorption correction	Semi-empirical from equivalents	
Max. and min. transmission	0.7455 and 0.6577	
Refinement method	Full-matrix least-squares on F^2	
Data / restraints / parameters	3255 / 0 / 186	
Goodness-of-fit on F^2	1.085	
Final R indices [I>2sigma(I)]	R1 = 0.0675, wR2 = 0.1108	
R indices (all data)	R1 = 0.1321, wR2 = 0.1288	
Largest diff. peak and hole	0.134 and –0.176 e·$Å^{-3}$	

IX. References

[1] G. Dettori, S. Gaspa, A. Porcheddu, L. De Luca, *Adv. Synth. Catal.* **2014**, *356*, 2709–2713.
[2] G. Dettori, S. Gaspa, A. Porcheddu, L. De Luca, *Org. Biomol. Chem.* **2014**, *12*, 4582–4585.
[3] S. Y. Hong, Y. Park, Y. Hwang, Y. B. Kim, M.-H. Baik, S. Chang, *Science* **2018**, *359*, 1016–1021.
[4] S. Huh, S. Y. Hong, S. Chang, *Org. Lett.* **2019**, *21*, 2808–2812.
[5] H. Jung, H. Keum, J. Kweon, S. Chang, *J. Am. Chem. Soc.* **2020**, *142*, 5811–5818.
[6] Z. Zhang, Y. Yu, L. S. Liebeskind, *Org. Lett.* **2008**, *10*, 3005–3008.
[7] Y. Jiang, H. Li, H. Tang, Q. Zhang, H. Yang, Y. Pan, C. Zou, H. Zhang, P. J. Walsh, X. Yang, *Chem. Sci.* **2025**, *16*, 962–969.
[8] H. Zhou, L. Ge, J. Song, W. Jian, Y. Li, C. Li, H. Bao, *iScience* **2018**, *3*, 255–263.
[9] J. Xu, W. Qiu, X. Zhang, Z. Wu, Z. Zhang, K. Yang, Q. Song, *Angew. Chem., Int. Ed.* **2023**, *62*, e202313388.
[10] B. Chen, P. Cao, X. Yin, Y. Liao, L. Jiang, J. Ye, M. Wang, J. Liao, *ACS Catal.* **2017**, *7*, 2425–2429.
[11] S. Wang, J.-X. Zhang, T.-Y. Zhang, H. Meng, B.-H. Chen, W. Shu, *Nat. Commun.* **2021**, *12*, 2771.
[12] Y. Yi, H. Gholami, M. G. Morrow, B. Borhan, *Org. Biomol. Chem.* **2017**, *15*, 9570–9574.
[13] M. He, C. Shi, M. Luo, C. Yang, L. Guo, Y. Zhao, W. Xia, *J. Org. Chem.* **2024**, *89*, 1967–1979.
[14] A. Leggio, E. L. Belsito, M. L. Di Gioia, V. Leotta, E. Romio, C. Siciliano, A. Liguori, *Tetrahedron Lett.* **2015**, *56*, 199–202.
[15] Y. He, C. Du, J. Han, J. Han, C. Zhu, J. Xie, *Chin. J. Chem.* **2022**, *40*, 1546–1552.
[16] D. N. Garad, S. B. Mhaske, *Org. Lett.* **2016**, *18*, 3862–3865.
[17] J.-I. Matsuo, T. Kozai, H. Ishibashi, *Org. Lett.* **2006**, *8*, 6095–6098.
[18] T. Aubineau, J. Laurent, L. Olanier, A. Guérinot, *Chemistry Methods* **2023**, *3*, e202300002.
[19] Ł. Woźniak, J. J. Murphy, P. Melchiorre, *J. Am. Chem. Soc.* **2015**, *137*, 5678–5681.
[20] H. Keum, H. Jung, J. Jeong, D. Kim, S. Chang, *Angew. Chem., Int. Ed.* **2021**, *60*, 25235–25240.
[21] R. G. Parr, W. Yang, *Density-Functional Theory of Atoms and Molecules*, Oxford University Press, **1994**.
[22] Y. Zhao, D. G. Truhlar, *Theor. Chem. Acc.* **2008**, *120*, 215–241.
[23] A. V. Marenich, C. J. Cramer, D. G. Truhlar, *J. Phys. Chem. B* **2009**, *113*, 6378–6396.
