# Supplementary material for: Harnessing Bifunctional N‐Benzoyloxyamides for Photoredox Amidative Dual Functionalizations of Alkenes
Source: Angew Chem Int Ed Engl. 2025 Jun 16;64(32):e202506290. doi: 10.1002/anie.202506290 (PMC12322644; doi:10.1002/anie.202506290)

checkCIF/PLATON report

Structure factors have been supplied for datablock(s) A25014_014

THIS REPORT IS FOR GUIDANCE ONLY. IF USED AS PART OF A REVIEW PROCEDURE FOR PUBLICATION, IT SHOULD NOT REPLACE THE EXPERTISE OF AN EXPERIENCED CRYSTALLOGRAPHIC REFEREE.

No syntax errors found. CIF dictionary Interpreting this report

Datablock: A25014_014

```
Bond precision:   C-C = 0.0028 A           Wavelength=0.71073

Cell:            a=17.1827(8)     b=5.2241(2)          c=16.8261(7)
                 alpha=90         beta=99.1338(14)     gamma=90
Temperature:     296 K

                Calculated                  Reported
Volume          1491.23(11)                 1491.23(11)
Space group     P 21/c                      P 21/c
Hall group      -P 2ybc                     -P 2ybc
Moiety formula  C17 H19 N O2                C17 H19 N O2
Sum formula     C17 H19 N O2                C17 H19 N O2
Mr              269.33                      269.33
Dx,g cm-3       1.200                       1.200
Z               4                           4
Mu (mm-1)       0.078                       0.078
F000            576.0                       576.0
F000'           576.25
h,k,lmax        21,6,21                     21,6,21
Nref            3259                        3258
Tmin,Tmax       0.998,0.998                 0.719,0.746
Tmin'           0.987

Correction method= # Reported T Limits: Tmin=0.719 Tmax=0.746
AbsCorr = MULTI-SCAN

Data completeness= 1.000           Theta(max)= 27.007

R(reflections)= 0.0449( 1992)                        wR2(reflections)=
                                                     0.1360( 3258)
S = 1.016                  Npar= 223
```

The following ALERTS were generated. Each ALERT has the format
test-name_ALERT_alert-type_alert-level.
Click on the hyperlinks for more details of the test.

Alert level C

```
PLAT906_ALERT_3_C Large K Value in the Analysis of Variance ......     10.190 Check
```

Alert level G

```
PLAT003_ALERT_2_G Number of Uiso or U(i,j)  Restrained non-H-Atoms         12 Report
PLAT178_ALERT_4_G The CIF-Embedded .res File Contains SIMU Records          1 Report
PLAT186_ALERT_4_G The CIF-Embedded .res File Contains ISOR Records          1 Report
PLAT188_ALERT_3_G A Non-default SIMU Restraint Value has been used     0.0100 Report
PLAT301_ALERT_3_G Main Residue  Disorder ..............(Resd    1)        30% Note
PLAT793_ALERT_4_G Model has Chirality at C10         (Centro SpGr)          S Verify
PLAT793_ALERT_4_G Model has Chirality at C12         (Centro SpGr)          R Verify
PLAT811_ALERT_5_G No ADDSYM Analysis: Too Many Excluded Atoms ....          ! Info
PLAT860_ALERT_3_G Number of Least-Squares Restraints ............         252 Note
PLAT910_ALERT_3_G Missing # of FCF Reflection(s) Below Theta(Min).          1 Note
                1  0  0,
PLAT969_ALERT_5_G The 'Henn et al.' R-Factor-gap value ..........       2.776 Note
              Predicted wR2: Based on SigI**2  4.90 or SHELX Weight 13.38
PLAT978_ALERT_2_G Number C-C Bonds with Positive Residual Density.          1 Info
```

   0 **ALERT level A** = Most likely a serious problem - resolve or explain
   0 **ALERT level B** = A potentially serious problem, consider carefully
   1 **ALERT level C** = Check. Ensure it is not caused by an omission or oversight
  12 **ALERT level G** = General information/check it is not something unexpected

```
   0 ALERT type 1 CIF construction/syntax error, inconsistent or missing data
   2 ALERT type 2 Indicator that the structure model may be wrong or deficient
   5 ALERT type 3 Indicator that the structure quality may be low
   4 ALERT type 4 Improvement, methodology, query or suggestion
   2 ALERT type 5 Informative message, check
```

It is advisable to attempt to resolve as many as possible of the alerts in all categories. Often the minor alerts point to easily fixed oversights, errors and omissions in your CIF or refinement strategy, so attention to these fine details can be worthwhile. In order to resolve some of the more serious problems it may be necessary to carry out additional measurements or structure refinements. However, the purpose of your study may justify the reported deviations and the more serious of these should normally be commented upon in the discussion or experimental section of a paper or in the "special_details" fields of the CIF. checkCIF was carefully designed to identify outliers and unusual parameters, but every test has its limitations and alerts that are not important in a particular case may appear. Conversely, the absence of alerts does not guarantee there are no aspects of the results needing attention. It is up to the individual to critically assess their own results and, if necessary, seek expert advice.

Publication of your CIF in IUCr journals

A basic structural check has been run on your CIF. These basic checks will be run on all CIFs submitted for publication in IUCr journals (*Acta Crystallographica*, *Journal of Applied Crystallography*, *Journal of Synchrotron Radiation*); however, if you intend to submit to *Acta Crystallographica Section C* or *E* or *IUCrData*, you should make sure that full publication checks are run on the final version of your CIF prior to submission.

Publication of your CIF in other journals

Please refer to the *Notes for Authors* of the relevant journal for any special instructions relating to CIF submission.

PLATON version of 19/12/2024; check.def file version of 19/12/2024

Datablock A25014_014 - ellipsoid plot

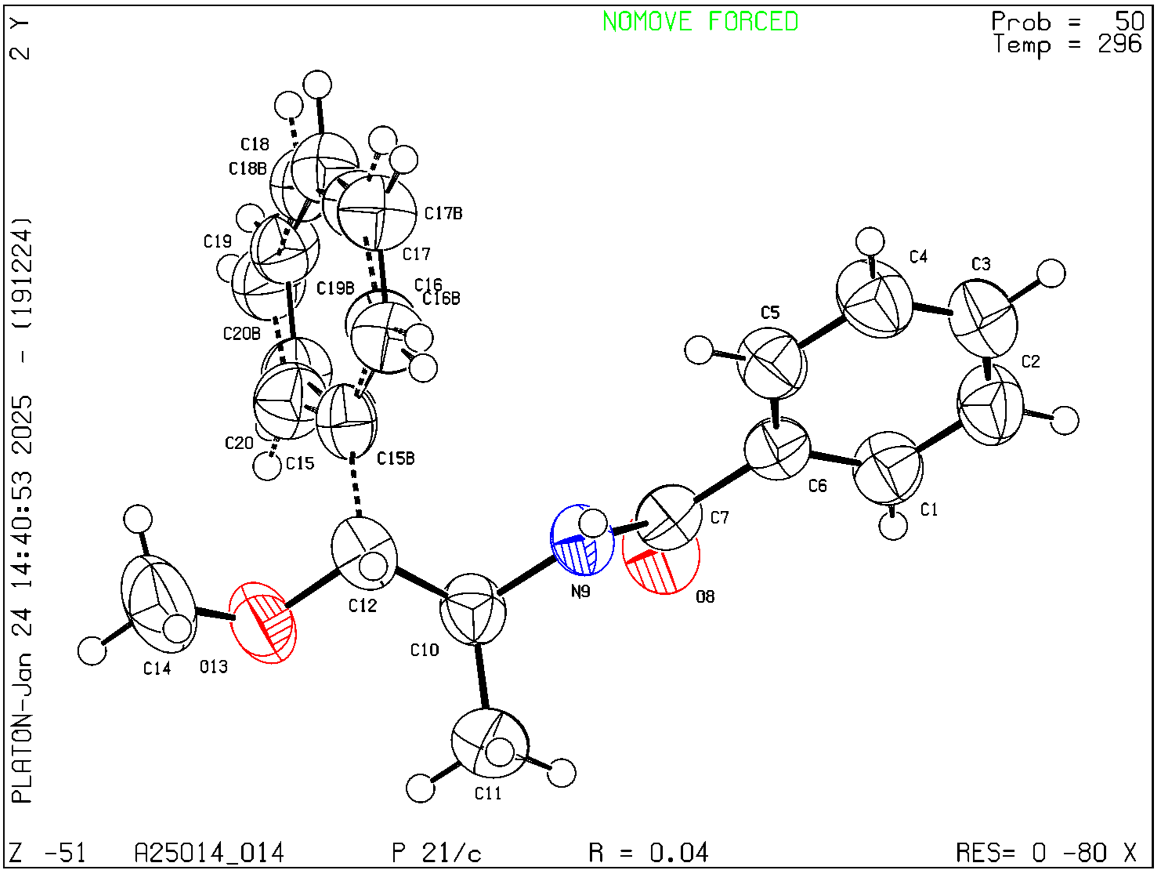

Supplement: Supplementary file 2 — Supporting Information [file ANIE-64-e202506290-s001.zip › 4m_checkcif.pdf]
