# Supplementary material for: Harnessing Bifunctional N‐Benzoyloxyamides for Photoredox Amidative Dual Functionalizations of Alkenes
Source: Angew Chem Int Ed Engl. 2025 Jun 16;64(32):e202506290. doi: 10.1002/anie.202506290 (PMC12322644; doi:10.1002/anie.202506290)

checkCIF/PLATON report

Structure factors have been supplied for datablock(s) A25025_002

THIS REPORT IS FOR GUIDANCE ONLY. IF USED AS PART OF A REVIEW PROCEDURE FOR PUBLICATION, IT SHOULD NOT REPLACE THE EXPERTISE OF AN EXPERIENCED CRYSTALLOGRAPHIC REFEREE.

No syntax errors found. CIF dictionary Interpreting this report

Datablock: A25025_002

Bond precision: C-C = 0.0031 A Wavelength=0.71073

Cell:	a=7.9210(2)	b=9.6187(3)	c=10.9640(3)
	alpha=96.7451(10)	beta=103.5952(10)	gamma=111.0918(11)
Temperature:	296 K		

	Calculated	Reported
Volume	738.50(4)	738.50(4)
Space group	P -1	P -1
Hall group	-P 1	-P 1
Moiety formula	C15 H20 N2 O3	C15 H20 N2 O3
Sum formula	C15 H20 N2 O3	C15 H20 N2 O3
Mr	276.33	276.33
Dx,g cm-3	1.243	1.243
Z	2	2
Mu (mm-1)	0.087	0.087
F000	296.0	296.0
F000'	296.14	
h,k,lmax	10,12,14	10,12,14
Nref	3575	3565
Tmin,Tmax	0.988,0.995	0.713,0.746
Tmin'	0.987	

Correction method= # Reported T Limits: Tmin=0.713 Tmax=0.746
AbsCorr = MULTI-SCAN

Data completeness= 0.997 Theta(max)= 27.984

R(reflections)= 0.0516(2616) wR2(reflections)= 0.1648(3565)

S = 1.064 Npar= 186

The following ALERTS were generated. Each ALERT has the format

test-name_ALERT_alert-type_alert-level.

Click on the hyperlinks for more details of the test.

Alert level C

```
PLAT241_ALERT_2_C High   'MainMol' Ueq as Compared to Neighbors of        C19 Check
PLAT906_ALERT_3_C Large K Value in the Analysis of Variance ......      3.109 Check
```

Alert level G

```
PLAT793_ALERT_4_G Model has Chirality at C7          (Centro SpGr)          R Verify
PLAT793_ALERT_4_G Model has Chirality at C10         (Centro SpGr)          S Verify
PLAT910_ALERT_3_G Missing # of FCF Reflection(s) Below Theta(Min).          1 Note
                0  0  1,
PLAT912_ALERT_4_G Missing # of FCF Reflections Above STh/L=  0.600          8 Note
PLAT969_ALERT_5_G The 'Henn et al.' R-Factor-gap value ..........       6.165 Note
              Predicted wR2: Based on SigI**2  2.67 or SHELX Weight 15.49
PLAT978_ALERT_2_G Number C-C Bonds with Positive Residual Density.          5 Info
PLAT992_ALERT_5_G Repd & Actual _reflns_number_gt Values Differ by          9 Check
```

0 **ALERT level A** = Most likely a serious problem - resolve or explain
0 **ALERT level B** = A potentially serious problem, consider carefully
2 **ALERT level C** = Check. Ensure it is not caused by an omission or oversight
7 **ALERT level G** = General information/check it is not something unexpected

0 ALERT type 1 CIF construction/syntax error, inconsistent or missing data
2 ALERT type 2 Indicator that the structure model may be wrong or deficient
2 ALERT type 3 Indicator that the structure quality may be low
3 ALERT type 4 Improvement, methodology, query or suggestion
2 ALERT type 5 Informative message, check

It is advisable to attempt to resolve as many as possible of the alerts in all categories. Often the minor alerts point to easily fixed oversights, errors and omissions in your CIF or refinement strategy, so attention to these fine details can be worthwhile. In order to resolve some of the more serious problems it may be necessary to carry out additional measurements or structure refinements. However, the purpose of your study may justify the reported deviations and the more serious of these should normally be commented upon in the discussion or experimental section of a paper or in the "special_details" fields of the CIF. checkCIF was carefully designed to identify outliers and unusual parameters, but every test has its limitations and alerts that are not important in a particular case may appear. Conversely, the absence of alerts does not guarantee there are no aspects of the results needing attention. It is up to the individual to critically assess their own results and, if necessary, seek expert advice.

Publication of your CIF in IUCr journals

A basic structural check has been run on your CIF. These basic checks will be run on all CIFs submitted for publication in IUCr journals (*Acta Crystallographica*, *Journal of Applied Crystallography*, *Journal of Synchrotron Radiation*); however, if you intend to submit to *Acta Crystallographica Section C* or *E* or *IUCrData*, you should make sure that full publication checks are run on the final version of your CIF prior to submission.

Publication of your CIF in other journals

Please refer to the *Notes for Authors* of the relevant journal for any special instructions relating to CIF submission.

PLATON version of 02/02/2025; check.def file version of 02/02/2025

Datablock A25025_002 - ellipsoid plot

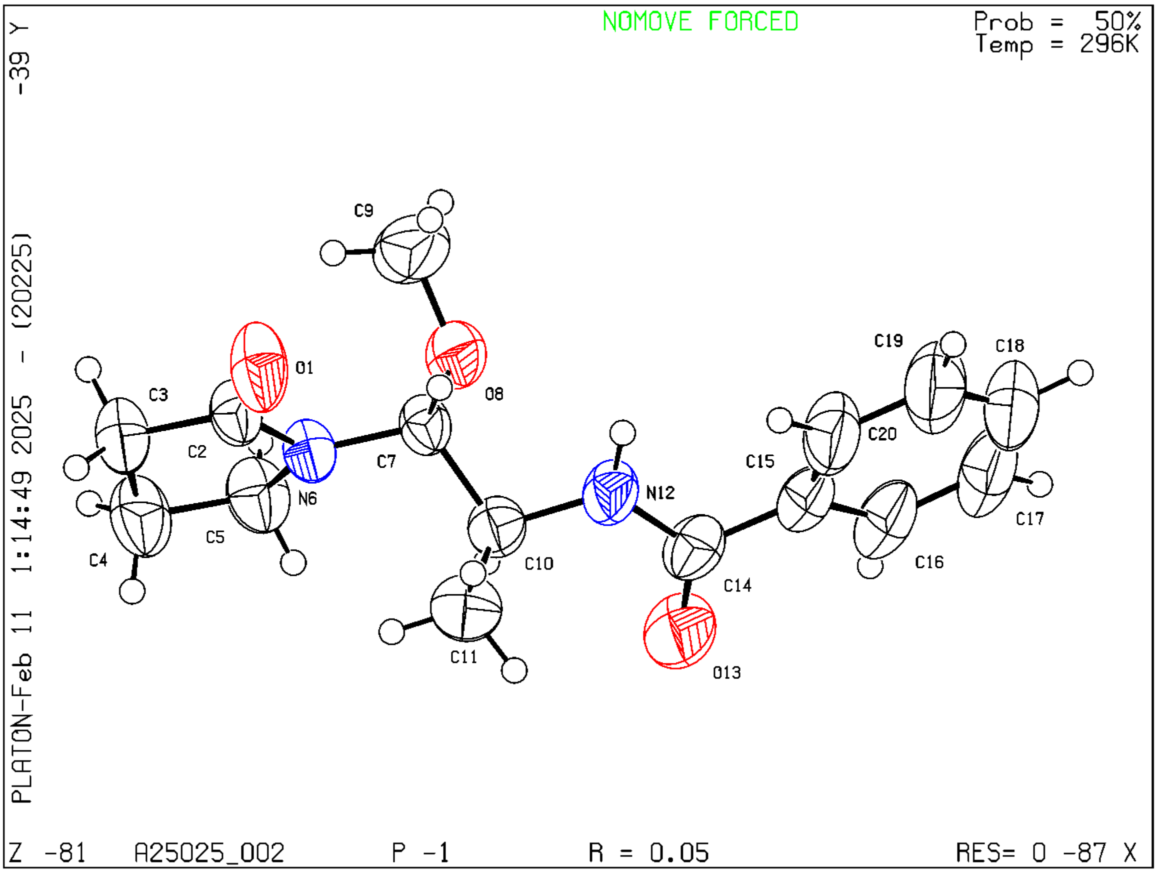

Supplement: Supplementary file 2 — Supporting Information [file ANIE-64-e202506290-s001.zip › 4s_checkcif.pdf]
