# Supplementary material for: Harnessing Bifunctional N‐Benzoyloxyamides for Photoredox Amidative Dual Functionalizations of Alkenes
Source: Angew Chem Int Ed Engl. 2025 Jun 16;64(32):e202506290. doi: 10.1002/anie.202506290 (PMC12322644; doi:10.1002/anie.202506290)

checkCIF/PLATON report

Structure factors have been supplied for datablock(s) A24193_061

THIS REPORT IS FOR GUIDANCE ONLY. IF USED AS PART OF A REVIEW PROCEDURE FOR PUBLICATION, IT SHOULD NOT REPLACE THE EXPERTISE OF AN EXPERIENCED CRYSTALLOGRAPHIC REFEREE.

No syntax errors found.   CIF dictionary   Interpreting this report

Datablock: A24193_061

```
Bond precision:   C-C = 0.0032 A          Wavelength=0.71073

Cell:              a=10.6944(5)      b=16.3834(7)      c=17.2431(7)
                   alpha=90          beta=90           gamma=90
Temperature:       203 K

                Calculated                     Reported
Volume          3021.2(2)                      3021.2(2)
Space group     P b c a                        P b c a
Hall group      -P 2ac 2ab                     -P 2ac 2ab
Moiety formula  C16 H18 N4 O2                  C16 H18 N4 O2
Sum formula     C16 H18 N4 O2                  C16 H18 N4 O2
Mr              298.34                         298.34
Dx,g cm-3       1.312                          1.312
Z               8                              8
Mu (mm-1)       0.090                          0.090
F000            1264.0                         1264.0
F000'           1264.50
h,k,lmax        13,20,22                       13,20,22
Nref            3305                           3283
Tmin,Tmax       0.995,0.998                    0.706,0.746
Tmin'           0.985

Correction method= # Reported T Limits: Tmin=0.706 Tmax=0.746
AbsCorr = MULTI-SCAN

Data completeness= 0.993          Theta(max)= 27.025

                                                    wR2(reflections)=
R(reflections)= 0.0513( 2337)                       0.1447( 3283)

S = 1.040                    Npar= 205
```

The following ALERTS were generated. Each ALERT has the format
test-name_ALERT_alert-type_alert-level.
Click on the hyperlinks for more details of the test.

Alert level C

```
PLAT906_ALERT_3_C Large K Value in the Analysis of Variance ......      7.557 Check
PLAT911_ALERT_3_C Missing FCF Refl Between Thmin & STh/L=    0.600         16 Report
                2  0  0,  12  2  0,  12  3  0,  12  4  0,  12  6  0,   6 13  0,
               12  1  1,  12  5  1,  11  8  1,  11 10  1,   9 13  1,  10  8  2,
               10  7  3,   9 12  3,  11  7  4,  10  2  6,
```

Alert level G

```
PLAT793_ALERT_4_G Model has Chirality at C7          (Centro SpGr)          S Verify
PLAT910_ALERT_3_G Missing # of FCF Reflection(s) Below Theta(Min).          2 Note
                0  2  0,   0  0  2,
PLAT912_ALERT_4_G Missing # of FCF Reflections Above STh/L=  0.600          4 Note
PLAT913_ALERT_3_G Missing # of Very Strong Reflections in FCF ....          1 Note
                2  0  0,
PLAT933_ALERT_2_G Number of HKL-OMIT Records in Embedded .res File          4 Note
               12  6  0,  12  5  1,  12  1  1,  11 10  1,
PLAT969_ALERT_5_G The 'Henn et al.' R-Factor-gap value ..........      3.245 Note
              Predicted wR2: Based on SigI**2  4.46 or SHELX Weight 13.92
PLAT978_ALERT_2_G Number C-C Bonds with Positive Residual Density.          4 Info
```

0 **ALERT level A** = Most likely a serious problem - resolve or explain
0 **ALERT level B** = A potentially serious problem, consider carefully
2 **ALERT level C** = Check. Ensure it is not caused by an omission or oversight
7 **ALERT level G** = General information/check it is not something unexpected

0 ALERT type 1 CIF construction/syntax error, inconsistent or missing data
2 ALERT type 2 Indicator that the structure model may be wrong or deficient
4 ALERT type 3 Indicator that the structure quality may be low
2 ALERT type 4 Improvement, methodology, query or suggestion
1 ALERT type 5 Informative message, check

It is advisable to attempt to resolve as many as possible of the alerts in all categories. Often the minor alerts point to easily fixed oversights, errors and omissions in your CIF or refinement strategy, so attention to these fine details can be worthwhile. In order to resolve some of the more serious problems it may be necessary to carry out additional measurements or structure refinements. However, the purpose of your study may justify the reported deviations and the more serious of these should normally be commented upon in the discussion or experimental section of a paper or in the "special_details" fields of the CIF. checkCIF was carefully designed to identify outliers and unusual parameters, but every test has its limitations and alerts that are not important in a particular case may appear. Conversely, the absence of alerts does not guarantee there are no aspects of the results needing attention. It is up to the individual to critically assess their own results and, if necessary, seek expert advice.

Publication of your CIF in IUCr journals

A basic structural check has been run on your CIF. These basic checks will be run on all CIFs submitted for publication in IUCr journals (*Acta Crystallographica*, *Journal of Applied Crystallography*, *Journal of Synchrotron Radiation*); however, if you intend to submit to *Acta Crystallographica Section C* or *E* or *IUCrData*, you should make sure that full publication checks are run on the final version of your CIF prior to submission.

Publication of your CIF in other journals

Please refer to the *Notes for Authors* of the relevant journal for any special instructions relating to CIF submission.

PLATON version of 22/08/2024; check.def file version of 21/08/2024

Datablock A24193_061 - ellipsoid plot

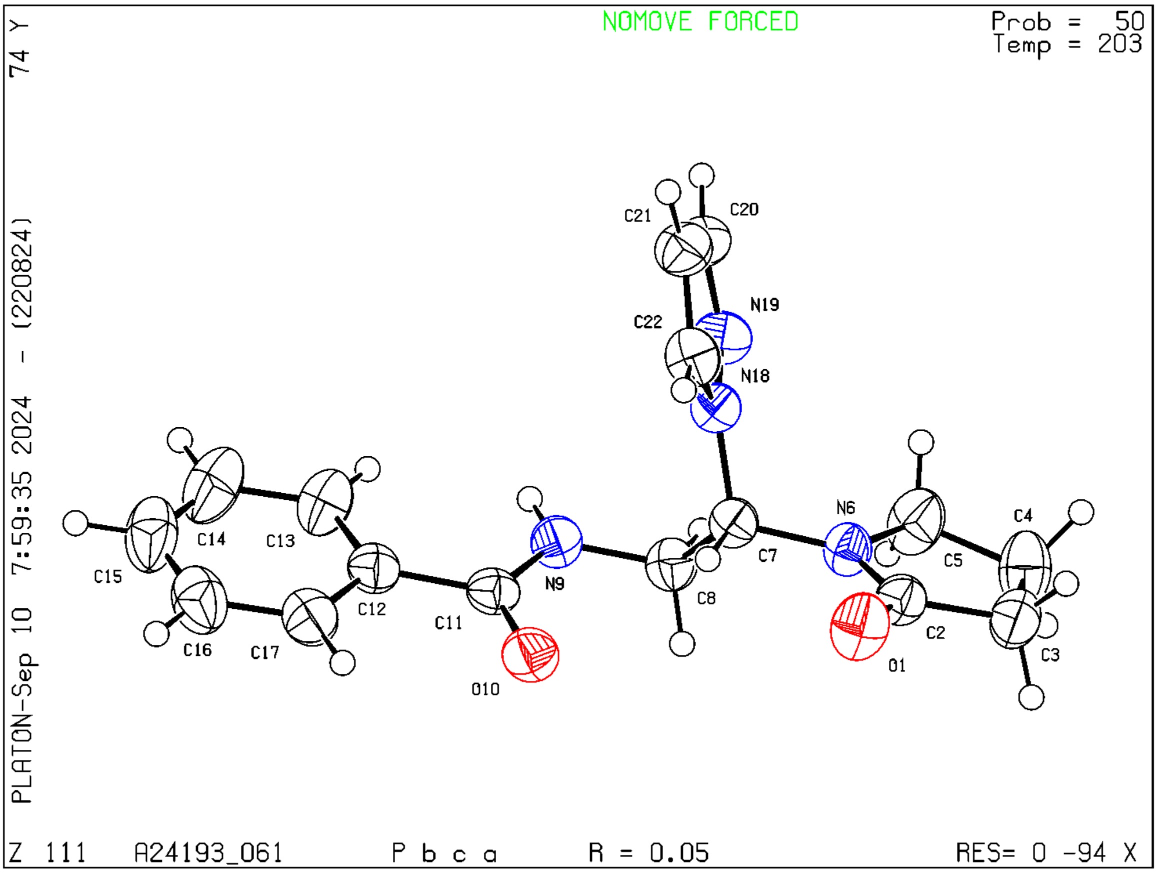

Supplement: Supplementary file 2 — Supporting Information [file ANIE-64-e202506290-s001.zip › 5h_checkcif.pdf]
