# Supplementary material for: Harnessing Bifunctional N‐Benzoyloxyamides for Photoredox Amidative Dual Functionalizations of Alkenes
Source: Angew Chem Int Ed Engl. 2025 Jun 16;64(32):e202506290. doi: 10.1002/anie.202506290 (PMC12322644; doi:10.1002/anie.202506290)

checkCIF/PLATON report

Structure factors have been supplied for datablock(s) A24221_014

THIS REPORT IS FOR GUIDANCE ONLY. IF USED AS PART OF A REVIEW PROCEDURE FOR PUBLICATION, IT SHOULD NOT REPLACE THE EXPERTISE OF AN EXPERIENCED CRYSTALLOGRAPHIC REFEREE.

No syntax errors found. CIF dictionary Interpreting this report

Datablock: A24221_014

Bond precision:	C-C = 0.0037 A		Wavelength=0.71073
Cell:	a=29.8217(15)	b=5.5779(3)	c=9.1072(4)
	alpha=90	beta=97.386(3)	gamma=90
Temperature:	203 K		

	Calculated	Reported
Volume	1502.34(13)	1502.34(13)
Space group	P 21/c	P 21/c
Hall group	-P 2ybc	-P 2ybc
Moiety formula	C17 H19 N O2	C17 H19 N O2
Sum formula	C17 H19 N O2	C17 H19 N O2
Mr	269.33	269.33
Dx,g cm-3	1.191	1.191
Z	4	4
Mu (mm-1)	0.078	0.078
F000	576.0	576.0
F000’	576.25	
h,k,lmax	38,7,11	38,7,11
Nref	3273	3255
Tmin,Tmax	0.990,0.999	0.658,0.746
Tmin’	0.988	

Correction method= # Reported T Limits: Tmin=0.658 Tmax=0.746
AbsCorr = MULTI-SCAN

Data completeness= 0.995 Theta(max)= 26.964

R(reflections)= 0.0675(1866) wR2(reflections)= 0.1288(3255)

S = 1.085 Npar= 186

The following ALERTS were generated. Each ALERT has the format
test-name_ALERT_alert-type_alert-level.
Click on the hyperlinks for more details of the test.

Alert level C

```
PLAT906_ALERT_3_C Large K Value in the Analysis of Variance ......     31.881 Check
PLAT906_ALERT_3_C Large K Value in the Analysis of Variance ......      3.609 Check
PLAT906_ALERT_3_C Large K Value in the Analysis of Variance ......      2.393 Check
PLAT911_ALERT_3_C Missing FCF Refl Between Thmin & STh/L=    0.600          7 Report
              -24  4  1,  -9  3  3, -30  0  4,  25  2  4,  19  3  6,  20  0  6,
               18  3  7,
```

Alert level G

```
PLAT793_ALERT_4_G Model has Chirality at C10         (Centro SpGr)          S Verify
PLAT910_ALERT_3_G Missing # of FCF Reflection(s) Below Theta(Min).          3 Note
                1  0  0,   2  0  0,   3  0  0,
PLAT912_ALERT_4_G Missing # of FCF Reflections Above STh/L=  0.600          8 Note
PLAT933_ALERT_2_G Number of HKL-OMIT Records in Embedded .res File          2 Note
               -9  3  3,  11  6  4,
PLAT969_ALERT_5_G The 'Henn et al.' R-Factor-gap value ..........      2.066 Note
              Predicted wR2: Based on SigI**2  6.23 or SHELX Weight 11.87
PLAT978_ALERT_2_G Number C-C Bonds with Positive Residual Density.          0 Info
```

0 **ALERT level A** = Most likely a serious problem - resolve or explain
0 **ALERT level B** = A potentially serious problem, consider carefully
4 **ALERT level C** = Check. Ensure it is not caused by an omission or oversight
6 **ALERT level G** = General information/check it is not something unexpected

0 ALERT type 1 CIF construction/syntax error, inconsistent or missing data
2 ALERT type 2 Indicator that the structure model may be wrong or deficient
5 ALERT type 3 Indicator that the structure quality may be low
2 ALERT type 4 Improvement, methodology, query or suggestion
1 ALERT type 5 Informative message, check

It is advisable to attempt to resolve as many as possible of the alerts in all categories. Often the minor alerts point to easily fixed oversights, errors and omissions in your CIF or refinement strategy, so attention to these fine details can be worthwhile. In order to resolve some of the more serious problems it may be necessary to carry out additional measurements or structure refinements. However, the purpose of your study may justify the reported deviations and the more serious of these should normally be commented upon in the discussion or experimental section of a paper or in the "special_details" fields of the CIF. checkCIF was carefully designed to identify outliers and unusual parameters, but every test has its limitations and alerts that are not important in a particular case may appear. Conversely, the absence of alerts does not guarantee there are no aspects of the results needing attention. It is up to the individual to critically assess their own results and, if necessary, seek expert advice.

Publication of your CIF in IUCr journals

A basic structural check has been run on your CIF. These basic checks will be run on all CIFs submitted for publication in IUCr journals (*Acta Crystallographica*, *Journal of Applied Crystallography*, *Journal of Synchrotron Radiation*); however, if you intend to submit to *Acta Crystallographica Section C* or *E* or *IUCrData*, you should make sure that full publication checks are run on the final version of your CIF prior to submission.

Publication of your CIF in other journals

Please refer to the *Notes for Authors* of the relevant journal for any special instructions relating to CIF submission.

PLATON version of 22/08/2024; check.def file version of 21/08/2024

Datablock A24221_014 - ellipsoid plot

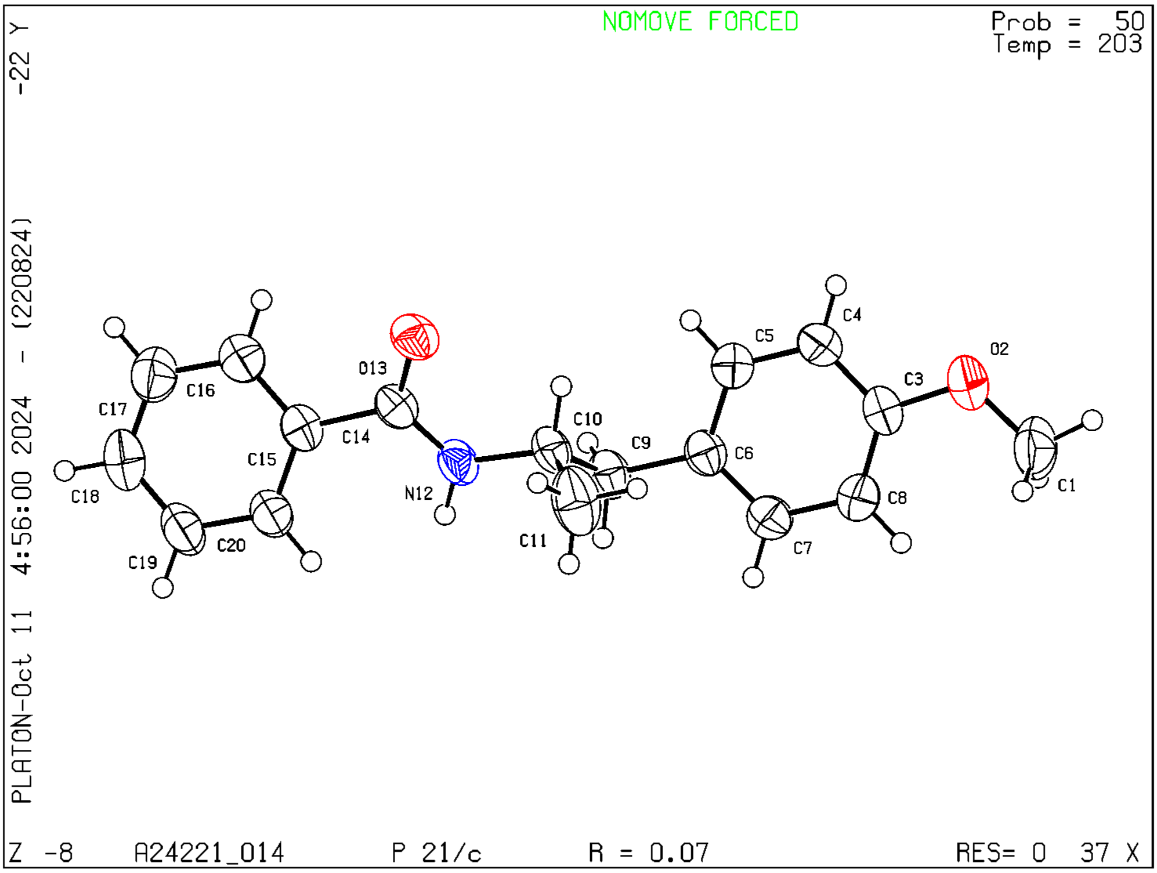

Supplement: Supplementary file 2 — Supporting Information [file ANIE-64-e202506290-s001.zip › 6h_checkcif.pdf]
